# Mapping the evolution of fertility support policies in China: A content and instrumental analysis

**DOI:** 10.1371/journal.pone.0332137

**Published:** 2025-10-09

**Authors:** Ying Yue, Xiaojun Yang, Jie Ma

**Affiliations:** 1 School of Public Policy and Administration, Xi’an Jiaotong University, Xi’an, China; 2 Jinhe Center of Economic Research, Xi’an Jiaotong University, Xi’an, China; The Islamia University of Bahawalpur Pakistan, PAKISTAN

## Abstract

Fertility support policies encompass measures providing economic, time, and childcare assistance to families with children. In China, these policies have continuously evolved in response to demographic shifts and changing socioeconomic environments. This study analyzes 226 policy texts across four stages using content analysis to examine two important dimensions—policy content and policy instruments. The results demonstrate that services and other forms of support outnumber time and financial provisions, reflecting a gradual diversification of policy content. Mandatory instruments are used more frequently than hybrid and voluntary ones, though the overall application of instruments is balanced. Different types of policy content employ distinct policy instruments. Moreover, the evolution of these policies reflects a process of adaptation to changing social contexts, the evolving role of women, and improved efficacy. However, current policies still face challenges including insufficient use of voluntary and hybrid instruments and inadequate attention to gender equality. To enhance fertility support, we recommend diversifying the use of instruments away from mandatory ones towards hybrid and voluntary options, ensuring comprehensive and inclusive policy content, and improving coordination across policy tools.

## 1. Introduction

Globally, low fertility rates have emerged as a significant challenge, affecting population structure, economic growth, and social sustainability. China has experienced a continuous decline in fertility in recent years, with its natural population growth rate dropping to −0.99‰ in 2024. This ultra-low fertility rate has prompted the Chinese government to adjust its fertility support policies to address demographic shifts [1]. Fertility support refers to assistance provided by various sectors of society to families raising children and adolescents, including time, financial, service, and employment support. Fertility support policies are public measures designed to ensure effective delivery of such assistance [2].

China’s population objectives have shifted significantly over time, driving corresponding adaptations in its fertility support policies. Early policies, aligned with the goal of “promoting population growth,” provided basic support, primarily focused on maternal time protection. During the era of “birth rate control”, notably the one-child policy [3], fertility support policies were introduced but largely shaped by restrictive measures, focusing on protecting maternal health. In response to today’s ultra-low fertility, the objective shifted to “enhancing birth rates and optimizing population structure,” leading to relaxed birth restrictions and the introduction of broader, more proactive support policies. Despite these efforts, fertility rates have continued to decline [4], falling to below 1.1 in 2024. Persistent low fertility, coupled with rapid population aging, highlights the critical need to understand and improve the effectiveness of fertility support policies.

The evolution of fertility support policies in response to shifting population objectives raises crucial questions: What stages has these fertility support policies undergone? What has been the main focus of policy content (i.e., the substantive support measures, such as financial subsidies, childcare services, or leave entitlements) at each stage? To what extent are the policy instruments (i.e., the implementation mechanisms, categorized as mandatory, hybrid or voluntary) mandatory? How have these features contributed to, or failed to reverse, the persistent decline in fertility rates? And how can fertility policies be improved? Answering these questions can deepen our understanding of how China’s fertility support policies have developed and functioned, providing guidance for future reforms. However, existing research primarily emphasizes on drawing lessons from international experiences [5] or analyzing the current domestic system [6], and analyses of policy evolution are predominantly qualitative [7]. There is a lack of systematic, quantitative analysis examining the interactions between specific policy content and instrument types over time. Filling this gap can help shed light on the limited effectiveness of existing fertility support policies on raising fertility rates.

This study employs a novel quantitative content analysis approach to examine the evolution of China’s fertility support policies. Using 226 national-level policy texts issued between 1949–2023, we develop a two-dimensional analytical framework [8] to examine policy instruments and policy content, as well as the interaction between the two aspects. Policy content captures the focus of a policy, while policy instrument represents the method of implementation. Instruments are typically categorized as mandatory, hybrid, or voluntary, based on the degree of government involvement [9]. This approach reveals the structural characteristics and evolutionary trajectory of the policy system, clarifies the underlying mechanisms of policy development, and provides valuable insights for improving fertility support policies [10].

## 2. Literature review

### 2.1. Family policies and fertility support in developed countries

Family policy refers to social measures enacted by governments to support family stability and functions [11]. Today, family policies cover the entire family lifecycle and focus on several key areas: (1) marriage and family; (2) fertility, parenting, and child services; (3) work-life balance; and (4) eldercare and caregiving. Among these, fertility support specifically targets assistance for families before and after childbirth. The term “fertility support” is rarely used explicitly in developed countries; it is typically considered part of broader family policy. This includes measures providing economic, time, and childcare support to families with children [12].

Family policies originated in late-nineteenth and early-twentieth century France and Sweden in response to the demographic changes and the effects of industrialization on families. In 1970s, many European countries adjusted family policies to support working families, including single-parent and low-income households, in response to social changes [13]. Fertility support policies specifically began in 19th-century Europe with the aim to protect women’s health. In 1877, Switzerland became the first country to grant pregnant women up to eight weeks of paid leave. Subsequently, Germany, Austria, Belgium, the Netherlands, and Sweden enacted legislation providing similar maternity leave to female workers [14].

Today, fertility support policies in many developed countries include three main components: financial, time-based, and service support. First, financial support policies include systems such as Japan’s comprehensive child allowance with annual payment adjustments [15] and the United States’ refundable tax credits, which benefit all families with children, including those without taxable income [16]. Second, countries such as Sweden, the United Kingdom, Norway, and Japan provide extensive leave [17]. Finally, childcare services are a critical element: Sweden provides an affordable public preschool system that guarantees placement for children over one within three to four months, with parental costs capped at about 8% of income [18], while South Korea provides universal free childcare services [19].

Developed countries have accumulated substantial practical experience in implementing fertility support policies with diverse objectives [20]. These goals include poverty reduction, income maintenance, child-rearing subsidies, promoting employment, advancing gender equality, fostering early childhood development, and encouraging higher birth rate. However, these policies differ significantly in scope, implementation, and underlying values across regions.

Nordic welfare states represent the most comprehensive model. They provide approximately one year of paid parental leave [21], universal and affordable public childcare [22], and paternity quotas in parental leave [23]. These measures not only provide effective fertility support, but paternity leave and accessible childcare also counteract the “mother as primary caregiver” paradigm, promoting equitable caregiving distribution. Moreover, extended leave allows parents to spend more time with their children, which is linked to lower high school dropout rates and improved future earnings [24].

Central European countries provide moderate financial support for families. Following Nordic examples, they prioritize the provision of parental leave and childcare services. For instance, Belgium and France have long offered public childcare [25], which contributes positively to fertility rates [26].

Anglo-Saxon countries, such as the United States, adopt a more minimalist approach. The U.S. provides only 12 weeks of unpaid parental leave under the 1993 Family and Medical Leave Act, and lacks adequate public childcare services [27]. Despite these limitations, even minimal support has shown positive effects on infant health [28] and fertility rates [29].

In contrast, Southern European, Eastern European, and East Asian countries emphasize traditional family structures in their fertility support systems. Although fertility support policies have been introduced and sometimes improve fertility [30], their effectiveness is constrained by gender norms. Parental leave uptake remains low among men, while extended maternity leave increases women’s career interruption risks. Moreover, families often face limited access to formal childcare [20]. These conditions reinforce traditional gender roles and perpetuates employment discrimination against women [31].

Overall, fertility support policies across regions reflect two distinct value orientations. The first is the “market-oriented” approach, which aims to reduce caregiving burden on families. Nordic countries such as Sweden and Finland exemplify this approach through extensive paid parental leave and universal childcare services. This strategy promotes more equal caregiving roles between parents and reduces welfare dependency through market-based solutions and public services [32,33]. The second is a “family-oriented” approach, which reinforces traditional caregiving roles. It assumes women as primary caregivers and supports men as breadwinners, primarily through maternity leave and parental benefits [34].

These contrasting models of fertility support policies corresponds to varying stages of policy development: Nordic policies present a mature stage, Central European policies are moderately developed, while East Asia policies remain in early development. Fundamentally, these differences stem from distinct institutional relationships between policy content and instruments, and from different roles of government, market, and family in sharing the cost of childbearing.

### 2.2. Chinese fertility support policies

The policy objectives of developed countries primarily focus on ensuring the welfare of women and children, with increased fertility rates viewed as a positive byproduct [35]. In this study, we adopt a similar perspective, prioritizing maternal and child welfare. Based on existing definitions [2], we define fertility support policies as encompassing all measures implemented in China since 1949 that support childbirth and parenting. These include maternity leave, reproductive health care, maternal health check-ups, labor protections for women, and more. Specifically, “fertility support policies” in this study cover both the family planning-era services aimed at safeguarding maternal-child health and the post-two-child era measures such as childcare designed to encourage childbirth.

China’s fertility support policies were first introduced in the early 1950s. In 1951, maternity leave of 56 days was established to protect the health of working women. The leave gradually increased to 90 days in 1988, then to 98 days in 2012. To encourage fathers’ childcare involvement and improve family well-being, paternity leave was piloted at the province level since 1980s, culminating in national standardization in 2016. Following the implementation of the two-child policy, a broad array of support measures emerged, including time, economic, service assistance, among other policy instruments.

However, notable gaps remain in current policy design. Maternity leave extends up to 188 days while paternity leave is limited to 30 days, reinforcing mothers’ caregiving role and contributing to women’s career interruptions, income loss, and reduced advancement opportunities. Furthermore, men’s low uptake of paternity leave driven by workplace stigma or job security concerns further entrenches traditional “breadwinner” norms [20]. Additionally, the high cost of childcare services often pushes women into flexible or part-time work, intensifying the motherhood penalty [31].

Existing research on China’s fertility support policies reveals three prominent trends. First, scholars often categorize policies from family-based perspective [36] or life-course approaches [6]. While these analyses address key aspects of fertility-related measures, they rarely explore the detailed evolution of fertility support policies over time. Second, most research relies on qualitative methods to analyze characteristics in specific stages, often relying on subjective interpretations, with limited use of quantitative tools to trace the evolutionary patterns. Third, studies often focus on a single policy dimension such as instruments, content, or goals, without in-depth analyses of how these components interact. For instance, some examine the alignment between population goals and fertility policies [7], track shifts in population objectives [37], and comprehensively analyze fertility support within wider family welfare framework [38]. The structural design and operational mechanisms of these policies remain underexplored.

Based on the current frameworks examining dimensions such as policy instruments, content, stages, objectives, and efficacy [10], we view public policy as a rational government response to competing interests, aiming to maximize social benefits. Aligning policy instruments with content—tailored to specific developmental stages—is key to effective policy design [39]. Understanding the co-evolution of policy instruments and policy content is essential for analyzing policy change. Instrument-content interactions offers multi-level insights into how governments pursue policy objectives through implementation strategies and substantive interventions across policy stages. However, existing research lacks quantitative examination of their longitudinal development. This paper uses content analysis to quantitatively trace the interaction between policy instruments and content over time, addressing a notable empirical gap in the literature.

This study contributes to research on fertility support policies in two ways. First, it analyzes the characteristics and coordination of policy content and instruments in Chinese fertility support policies. Second, it demonstrates how these elements interact across different policy stages, offering a new perspective on policy mechanisms and evolution. By analyzing these dynamics across China’s policy stages and incorporating models from the developed countries’, this study provides guidance for optimizing fertility support policies.

## 3. Analytical framework, methodology, and data

### 3.1. Analytical framework

This study examines interactions between policy content and instruments. It employs a two-dimensional analytical framework structured along two axes: policy content on the X-axis) and policy instruments on the Y-axis. The framework proposes three propositions to help explain the evolution of fertility support policies.

Proposition 1: Policy content reflects the stage-specific priorities of fertility support policies. Tracking changes in content can reveal shifts in underlying policy objectives [10].

Proposition 2: Policy instruments refer to the means by which governmental implements policy [39]. Initially, fertility support policies relied primarily on mandatory instruments, such as administrative orders setting maternity leave or penalties for prohibited labor practices. Over time, a more balanced use of mandatory, hybrid, and voluntary instruments emerged, including regulations, subsidies, and public-private partnerships to enhance childcare services.

**Fig 1:**
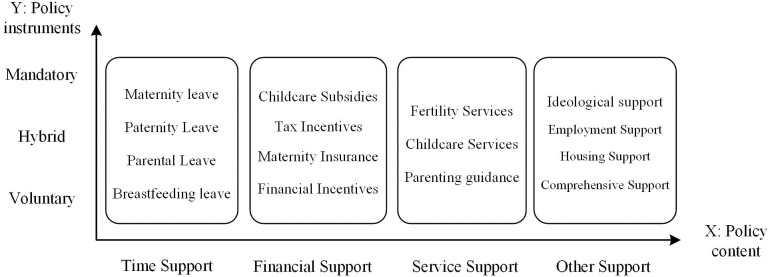
Two-dimensional analysis framework of fertility support policy.

Proposition 3: The evolution of fertility support policies is shaped by the interactions between instrument and content. Improving coordination between the two can enhance the overall policy effectiveness. The analytical framework is illustrated in Fig 1.

#### 3.1.1. X: Policy content.

Fertility support policies in China include maternity and paternity leave, health check-ups for pregnant and postpartum women, labor protections for women, childcare services, tax deductible for children, and child subsidies, among others. Using systematic text coding, we classify these policies into four primary categories and fifteen subcategories: time support, financial support, service support, and other support (Table 1). These categories represent key fertility decision factors—time costs, economic pressures, care resources, and social environment. This classification ensures clear policy boundaries to avoid overlap, and aligns with an established analytical framework for fertility support policies [2]. These categories represent the policy content dimension (X-axis) of our analytical framework.

**Table 1:** Policy content dimension index.

Policy content		Content description	Code
Time Support	Maternity leave	Ensure the safety of women’s maternity and provide adequate time for recovery post-delivery	111
	Paternity Leave	Men take care of his wives and children	112
	Parental Leave	Parents take care of the baby together	113
	Breastfeeding leave	Guarantee designated breastfeeding time	114
Financial Support	Childcare Subsidies	Subsidies for families with many children	121
	Tax Incentives	Tax deductions for children’s education and childcare expenses	122
	Maternity Insurance	Maternity medical expenses, maternity allowance	123
	Financial Incentives	Extend financial support to enterprises engaged in maternal and infant care and childcare services	124
Service Support	Fertility Services	Deliver maternal and infant safety services throughout the reproductive process	131
	Childcare Services	Care and development services for infants and young children	132
	Parenting guidance	Childcare guidance services for families	133
Other Support	Ideological support	Advocate recognition of childbirth’s value	141
	Employment Support	Protect the legal rights of working parents	142
	Housing Support	Home purchase subsidies and rental discounts	143
	Comprehensive Support	Providing fertility support in various ways	144

#### 3.1.2. Y: Policy instruments.

Policy instruments are the tools through which governments translate policy goals into concrete actions. Public policies are designed to motivate specific behaviors, and selecting appropriate instruments is essential for achieving the goal [40]. Scholars have developed several criteria to classify policy instruments: the locus of intervention (demand-, supply-, environment-driven) [41], the type of resource they use (organizational, authoritative, financial, or informational) [42], and the degree of government involvement (mandatory, hybrid, or voluntary) [9].

In the context of fertility support, policy instruments include mandating companies to provide 98 days of maternity leave, providing financial subsidies and tax incentives to develop community childcare services, and improving the quality of childcare by offering relevant programs in higher education and vocational schools. These instruments vary in their degree of government enforcement—some are compulsory, while others are suggestive—which results in different levels of policy effectiveness.

Given that the effectiveness of fertility policies depends heavily on enforcement capacity, this study adopts Howlett’s classification of policy instruments, mandatory, hybrid, and voluntary types, as the Y-dimension of our analytical framework [9]. This framework reveals the degree of mandatory enforcement in policy implementation, ranging from mandatory to voluntary participation. In contrast, classifications based on the locus of intervention (e.g., supply or demand) or resource type (e.g., financial or authority) are unable to distinguish the degree of policy enforcement effectively.

Mandatory policy instruments include administrative orders, institutional measures, regulations, supervision and management, and penalties. Hybrid policy instruments include financial subsidies, tax relief, policy support, and advocacy activities. Voluntary policy instruments involve collaboration with market entities, private capital, educational institutions, social organizations, and community networks (Table 2).

**Table 2:** Indicators of policy instrument dimension.

Policy instruments		Instruments description	Code
Mandatory	Administrative orders	Orders and Requirements for Fertility Support	211
	Institutional measures	Improvement of policies and institutions related to fertility support	212
	Regulations	Definition of duties of responsible parties	213
	Supervision and management	Supervision and management of the fertility support program	214
	Penalties	Penalties for violating relevant policies and regulations	215
Hybrid	Financial subsidies	Financial subsidies to enterprises or individuals who support childbirth	221
	Tax relief	Tax and fee concessions for enterprises or individuals that support childbirth	222
	Policy support	Policy support for enterprises or individuals that support childbirth	223
	Advocacy activities	Creating a supportive environment for childbirth through advocacy activities	224
Voluntary	Relying on market institutions	Relying on various organizations, enterprises, and private capital to support childbirth	231
	Relying on education institutions	Relying on education and research institutions to support childbirth	232
	Relying on social organizations	Relying on social organizations to support childbirth	233
	Relying on Community	Relying on community networks to support fertility	234

### 3.2. Methods and data

This article uses content analysis to examine policy texts. Content analysis systematically quantifies and analyzes explicit textual content using specific, replicable coding rules [43]. This approach minimizes subjectivity and ambiguity in qualitative analyses, clarifying policy contexts and trends.

#### 3.2.1. Data collection.

We selected national-level fertility support policy issued between October 1949 and January 2023. These include government initiatives aimed at encouraging childbirth, such as childcare subsidies and services, as well as reproductive health policies from the family planning era designed to protect women’s well-being and children’s health.

Policy texts were sourced from the Peking University Law Database. Collection followed these steps:

First, we conducted pilot searches with keywords in the “policy content” dimension, including “maternity leave,” “wages during maternity leave,” “paternity leave,” “parental leave,” “lactation leave,” “care leave,” “pregnancy,” “infants,” “childcare,” “early childhood education,” “parenting guidance,” “birth subsidies,” “medical expenses for childbirth,” “maternity insurance,” “delivery,” and “gestation.” The pilot search revealed that some of the retrieved texts were only loosely related to fertility support.

Second, we refined the keywords to “maternity leave,” “reward leave,” “paternity leave,” “maternity insurance,” “childcare subsidies,” “childcare services,” “female employee protection,” “maternal,” “infant healthcare,” and “maternity medical expenses.” We also supplemented searches using State Council and ministry websites.

Finally, five graduate students screened out irrelevant texts, resulting in 226 valid policy documents. We prioritized authoritative document types such as notification, law, opinions, resolution, decision, regulations and plans, and excluded responses/ approvals.

#### 3.2.2. Text encoding.

The policy texts were manually coded following this process: First, each content units of the 226 policy texts was assigned a unique ID in the format “policy number-code number.” Individual statements within the texts served as the unit of analysis. All fertility-related statements were coded to avoid omissions. Next, we applied a two-dimensional framework to code each content unit by both content and instrument dimensions [44]. For policy content, we categorized it into four types: time support, financial support, service support, and other support. Each statement was read and coded accordingly. Policy instruments were classified into three types: mandatory, hybrid, and voluntary based on the level of government involvement described in the statement.

We implemented the following process to ensure the consistency of manual coding: (1) Five graduate students underwent a one-week training to standardize their understanding of the coding methodology. (2) Coders were organized into five virtual groups of three members each. Through systematic rotation, each coder participated in multiple groups. (3) All coders independently coded the same five texts in a trial round, followed by group discussions to reach consensus. (4) During the formal coding process, two coders independently coded each text, with a third coder reviewing for consistency. (5) Within this formal coding phase, to assess inter-coder reliability, we calculated Cohen’s Kappa coefficient based on the initial independent coding assignments by the first two coders for each text. This achieved a Kappa coefficient of 0.865, indicating a high degree of agreement. [45]. For texts where the initial two coders disagreed, the third coder intervened to facilitate discussion and reach a final consensus.

Following this procedure, the 226 policy texts produced 962 content units. For example, in Table 3, the policy titled “Guiding Opinions on Improving Supportive Measures for Active Childbirth” was assigned the policy number “44.” The statement “Fees of public childcare institutions are set by local governments, and supervision of fees of universal childcare institutions is strengthened” was the second content unit of this policy text; and thus its sequence number was “2.” The policy content fell under “service support,” specifically “childcare services,” thus resulting in a policy content code of “132”. The policy employed a “supervision and management” tool categorized under “mandatory,” leading to an instrument code of “214”.

**Table 3:** Example of policy analysis unit coding process.

Policy No.	Policy name	Policy analysis unit	Code No.	Content code	Instrument code
44	Guiding Opinions on Improving Supportive Measures for Active Childbirth	Fees of public childcare institutions are set by local governments, and supervision of fees of universal childcare institutions is strengthened.	2	132	214
99	Special Regulations on the Labor Protection of Female Employees	Female employees are entitled to 98 days of maternity leave, of which 15 days may be taken antepartum; in the event of obstructed labor, an additional 15 days of maternity leave are granted; for multiple births, an additional 15 days of maternity leave are awarded for each additional infant.	5	111	211

Another example is the policy “Special Regulations on the Labor Protection of Female Employees” with a policy number “99”. The statement “Female employees are entitled to 98 days of maternity leave, of which 15 days may be taken antepartum; in the event of obstructed labor, an additional 15 days of maternity leave are granted; for multiple births, an additional 15 days of maternity leave are awarded for each additional infant” was the fifth content unit with a sequence number of “5”. The policy content fell under “time support,” specifically “maternity leave,” resulting in a content code of “111.” The policy employed an “administrative orders” instrument categorized under “mandatory,” leading to an instrument code of “211.”

## 4. Results

### 4.1. Overall analysis of fertility support policies

The number of fertility support policies in China has fluctuated across different policy periods between 1949 and 2023. In the early years of nation-building, the Chinese government introduced a concise yet foundational set of regulations to quickly establish a fertility support framework. In 1978, “family planning” was formally included in the Constitution, followed by successive policies on eugenics and maternal health, with a gradual increase in policy issuance during this period. Since 2015, the implementation of the “two-child” policy marked a shift toward relaxing fertility restrictions and actively promoting childbirth through various governmental measures, resulting in a substantial increase in policy output.

As shown in Table 4, under the policy content dimension, service support accounts for the largest share of fertility support policies (50.42%), followed by other support (25.99%), financial support (16.74%), and time support (6.68%). Scholars have traditionally viewed China’s fertility support policies as predominantly emphasizing time and financial support [36]. This finding show that the content has diversified significantly, now encompassing service, employment, ideological, and housing support measures.

**Table 4:** Overview of fertility support policies (Quantity/Proportion).

	Mandatory	Hybrid	Voluntary	Total
Time Support	62/6.44%	4/0.42%	0/0%	66/6.68%
Financial Support	132/13.72%	26/2.7%	3/0.31%	161/16.74%
Service Support	227/23.6%	138/14.35%	120/12.47%	485/50.42%
Other Support	123/12.79%	112/11.64%	15/1.56%	250/25.99%
Total	544/56.55%	280/29.11%	138/14.35%	962/100%

Under the policy instruments dimension, most fertility policies use mandatory instruments (56.55%), followed by hybrid instruments (29.11%), and voluntary instruments (14.35%). The dominance of mandatory instruments not only reflects the government’s prioritization of fertility support and its directive role in implementation, ensuring compliance through legal and administrative means. For example, the “Regulations on the Labor Protection of Female Employees” (1988) state: “During the breastfeeding period, female employees cannot be assigned to third-level physical labor as defined by the state. Their working hours must not be extended.” This provision effectively protects the health of nursing mothers. Meanwhile, hybrid and voluntary policy instruments enhance flexibility and promote social participation.

The distribution of policy content and instruments combinations show that “service support-mandatory” accounts for the highest proportion (23.6%). Across all four policy content categories, mandatory instruments predominate, especially in time support, where hybrid and voluntary instruments are relatively lacking. For example, the “Special Provisions on Labor Protection for Female Employees” (2012) state: “Employers shall arrange one-hour breastfeeding breaks for female employees during their breastfeeding period within the daily working hours.” However, the effectiveness of breastfeeding Leave policies depends on enterprise-level implementation. Imposing corporate responsibilities without complementary cost-sharing policies may hinder execution at the organizational level.

Similarly, service support policies, such as those for childcare services, are closely tied to the market and still rely heavily on mandatory instruments. For instance, “Guiding Opinions on Improving Supportive Measures for Active Childbirth” (2022) states: “fees of public childcare institutions are set by local governments, and supervision of fees of universal childcare institutions is strengthened.” While such price controls can lower costs, they may also reduce providers’ incentives for quality improvement, potentially failing to meet diverse consumer needs.

### 4.2. Evolutionary milestones of fertility support policies

This section combines policy paradigm analysis and punctuated-equilibrium theory to examine the evolution of fertility support policy content and instruments in China. Policy paradigm analysis, rooted in Peter Hall’s pioneering work in social policy, provides a framework to understand how policy ideas, goals, tools, and problem definitions evolve. Hall identifies three levels of change: adjustments in tool settings, alterations in the tools themselves, and fundamental transformations in overarching goals and conceptual frameworks [46]. Changes at the third level signify a paradigm shift, reflecting deep transformation in social policy. This framework allows us to assess the extent and direction of changes in fertility support policies by examining their objectives, content, and instruments.

Punctuated-equilibrium theory, adapted from evolutionary biology of the alternating patterns of long-term stability and short-term dramatic changes in species evolution, explains the nonlinear characteristics of policy change—specifically the phenomenon of prolonged stability interspersed with occasional, significant transformations [47]. In this study, the punctuated-equilibrium framework reveals the interactions between conflicts and institutions during policy shifts and clarifies the conditions under which disruptive changes in fertility support policies occur.

Within this integrated framework, the overall policy objectives function as key indicators of paradigm shifts—any change signifies a transformation at Hall’s third level [48]. Meanwhile, punctuated-equilibrium theory attributes policy discontinuity to the emergence of new “conflicts” arising from shifting historical contexts. In the periods of lacking such conflicts, policy goals remain stable, and adjustments to fertility support content and instruments reflect incremental equilibrium adjustments. Conversely, when new conflicts arise, shifts in overarching goals drive fundamental changes to policy content and instruments, marking discontinuous punctuated change.

Since the founding of the People’s Republic of China in 1949, significant shifts in population trends, family size, and family structure have directly shaped the country’s fertility support policies. This paper integrates policy paradigm analysis with punctuated-equilibrium theory, focusing on the landmark events in China’s population development. By employing these pivotal events as benchmarks, it summarizes the distinctive characteristics of fertility support policies across various stages. This section analyzes “when fertility support policies change,” while the next section will address “how they change” and “why they change.”

**Fig 2:**
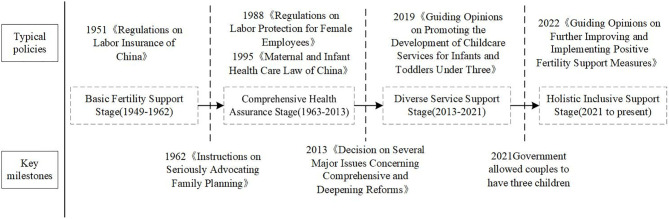
Milestones and stages in the evolution of fertility support policies.

China’s fertility support policies can be divided into four stages: the Basic Fertility Support Stage (1949–1962); the Comprehensive Health Assurance Stage (1963–2013); the Diverse Service Support Stage (2013–2021); and the Holistic Inclusive Support Stage (2021 to present), as shown in Fig 2.

The Basic Fertility Support Stage (1949–1962): This early stage reflected the government’s aim to encourage childbirth in order to revive the post-war economy. The primary population objective was to “promote population growth”. Policies were still in their formative stages, and the state adopted basic measures to support women of childbearing age. A key policy from this stage was the “Labor Insurance Regulations” (1951). This regulation not only protected women’s rights to maternity leave and health—stating that “if a woman is unable to work after her maternity leave, she shall be treated according to illness regulations upon medical verification”—but also established maternity benefits: “maternity allowances shall be disbursed from the labor insurance fund.” During this period, China’s population grew from 540 million (1949) to 673 million (1962). Family structures were predominantly multigenerational extended families with large sizes.

The Comprehensive Health Assurance Stage (1963–2013): As the pressure on social resources caused by rapid population growth became increasingly pronounced, the 1962 directive “On Seriously Advocating Family Planning” marked the start of family planning era in China. Although initially limited in scope, family planning was strengthened over the following decades, and by 1982, the one-child policy was formally added to the Constitution. The population objective shifted to “birth control.” This shift did not eliminate fertility support; rather, the state focused on maternal and child health, improving public health literacy and access to services [2]. Key policies included “Regulations on the Labor Protection of Female Employees” (1988) and “Implementation Measures of the Maternal and Child Health Care Law” (1995). The “Implementation Measures of the Maternal and Child Health Care Law” mandated services such as prenatal care, sterilized delivery, newborn resuscitation, postpartum visits, breastfeeding instruction, and health education. During this period, China’s population exhibited a substantial increase, initially at a rapid rate and subsequently at a more gradual one. Population growth slowed from 692 million (1963) to 1.143 billion (1990) and then to 1.367 billion (2013). Family size has gradually shrunk, with nuclear families (parents and unmarried children) becoming the norm.

The Diverse Service Support Stage (2013–2021): During this stage, China faced declining fertility alongside the remaining demographic dividend. In 2013, couples where one partner was an only child were allowed to have two children; by 2015, the policy expanded to all couples. The population goals shifted to “mitigating aging and the decline of the working-age population while optimizing population structure.” The government emphasized service-based support for fertility. A key policy in 2019, the Guiding Opinions on Promoting the Development of Childcare Services for Infants Under Three Years Old, encouraged market-based childcare services. It required local governments to plan for land use for childcare centers, supported workplace childcare facilities, and encouraged kindergartens to offer nursery classes. Population growth slowed further, rising from 1.367 billion (2013) to 1.413 billion (2021). Meanwhile, fertility rates declined, and nuclear/single-person households, delayed marriage, and postponed childbearing became more common.

The Holistic Inclusive Support Stage (2021 to present): The persistently low fertility rate and accelerating population ageing has prompted government to adopted more comprehensive and inclusive fertility support measures. In 2021, the Political Bureau of the Central Committee of the Communist Party of China implemented the three-child policy. In 2022, the Guiding Opinions on Further Improving and Implementing Proactive Birth Support Measures issued by the National Health Commission, the National Development and Reform Commission, and the Publicity Department of the Central Committee became a key landmark fertility support policy of this period. This policy aims to create a women-friendly, life-cycle approach by optimizing childcare services, enhancing maternity insurance, and eliminating employment discrimination. During this period, China’s natural population growth rate turned negative for three consecutive years. The population has fallen from 1.413 billion in 2021 to 1.408 billion in 2024. Over 20% of the population is now aged 60 or above. Trends in non-marriage, delayed marriage, and postponed childbirth further weaken the reproductive function of families.

### 4.3. Characteristics of the evolution of the fertility support policy

Research on policy change should address not only the transformations but also the underlying driving forces [47]. This section explains “how” and “why” fertility support policies have evolved by examining elements within policy texts—namely, policy content and instruments. Understanding the distinct policy stages is essential for tracing the evolution of fertility support policies. By analyzing the content, instruments, and their interactions across stages, we gain insight into the internal logic of fertility support policies and can better predict future trends.

#### 4.3.1. Evolution of policy content.

The evolution of policy content across the four stages indicates changes in the government’s objectives and priorities. In total, the 962 fertility support policy units span four main categories and 15 subcategories, as shown in Table 5.

**Table 5:** Evolution of fertility support policy content.

Policy content		1949-1962	1963-2013	2013-2021	2021 to present
Time Support	Maternity leave	8	21	8	5
	Paternity Leave	0	2	5	1
	Parental Leave	0	1	1	1
	Breastfeeding leave	0	10	2	1
	Total Time Support	8	34	16	8
Financial Support	Childcare Subsidies	5	8	3	2
	Tax Incentives	0	1	0	9
	Maternity Insurance	2	69	48	12
	Financial Incentives	0	1	0	1
	Total Financial Support	7	79	51	24
Service Support	Fertility Services	0	74	72	45
	Childcare Services	0	13	178	88
	Parenting guidance	0	0	12	3
	Total Service Support	0	87	262	136
Other Support	Ideological support	0	37	19	13
	Employment Support	2	49	17	27
	Housing Support	0	0	1	5
	Comprehensive Support	1	20	40	19
	Total Other Support	3	106	77	64
Total		18	306	406	302

The goal of the Basic Fertility Support Stage (1949–1962) was to quickly establish foundational policies for the newly formed People’s Republic of China. Thus, the policy content primarily focused on time and financial support, including maternity leave (8) and childbirth subsidies (5). For instance, the “Labor Insurance Regulations” (1953) stated: “female employees are entitled to a total of fifty-six days of maternity leave, during which their salaries will continue to be paid,” and “the costs of medical examinations and childbirth are borne by the enterprise administration or the employer.” This aligns with initial international models focusing on maternity leave, job protection, and wage compensation [5]. Though limited in scope, these policies effectively addressed the need for rapid policy implementation and aligned with global standards.

The policy priorities of the Comprehensive Health Assurance Stage (1963–2013) shifted to reproductive health and population quality. Therefore, the main focuses were on maternal and infant health (74), maternity insurance (69), and employment support (49). Few policies addressed paternity leave, tax incentives, or childcare services. Key objectives also included promoting gender equality in labor protection. For example, the “Law on the Protection of Women’s Rights and Interests” (1992) stated: “No unit may dismiss female employees or unilaterally terminate labor contracts due to marriage, pregnancy, maternity leave, or breastfeeding.” Additionally, the “Regulations on the Health Care of Female Employees” (1993) prohibited employers from assigning night shifts or overtime work to female employees with infants younger than one year.

During the Diverse Service Support Stage (2013–2021), the government expanded fertility policies to support the “two-child” policy. Policy content included providing childcare services (178), fertility services (72), and comprehensive support (40). Parental leave and housing support remained scarce, and tax incentives had yet to emerge. The goal was to optimize population structure, improve maternal and infant health, protect women’s rights, and promote gender equality, to create a support system for families of childbearing age. For example, the “Guiding Opinions on Further Enhancing Youth Marriage and Love Affairs” (2017) stated, “Protect reproductive rights of young women and ensure material support and leave during pregnancy, childbirth, and breastfeeding stages.”

In the Holistic Inclusive Support Stage (2021 to present), the government has introduced comprehensive fertility support measures to promote the three-child policy. The policy content primarily focuses providing childcare services (88), fertility services (45), and employment support (27) for families of childbearing age. Policies now address the entire process of conception, parenting, and upbringing. Special emphasis is placed on protecting women’s employment rights, fostering a fertility-friendly work environment, and supporting both parents in sharing family responsibilities. For example, the “Outline for the Development of Women and Children in China” (2021) calls for, “exploring the implementation of parental leave” and “encouraging employers to establish facilities such as breastfeeding rooms.”

An analysis of the evolution of policy content across the four stages shows that policies related to time support were introduced early, with a robust set established during the Basic Fertility Support Stage (1949–1962) and subsequently strengthened in later stages. In contrast, service support policies surged during the Diverse Service Support Stage (2013–2021), along with a slight rise in other supportive measures. Overall, the range of policy content has evolved from single-focus to diversified, reflecting a shift from “family-oriented” to “market-oriented” policy values.

#### 4.3.2. Evolution of policy instruments.

The evolution of policy instruments across the four stages reflects changing levels of government involvement. Overall, the 962 units of fertility support policies include three major categories and thirteen subcategories of policy instruments, as shown in Table 6.

**Table 6:** Evolution of fertility support policy instruments.

Policy instruments		1949-1962	1963-2013	2013-2021	2021 to present
Mandatory	Administrative orders	11	61	45	18
	Institutional measures	0	39	82	41
	Regulations	4	61	55	24
	Supervision and management	0	11	20	15
	Penalties	0	40	9	8
	Total Mandatory	15	212	211	106
Hybrid	Financial subsidies	2	7	7	14
	Tax relief	0	1	4	12
	Policy support	0	32	28	19
	Advocacy activities	1	31	79	43
	Total Hybrid	3	71	118	88
Voluntary	Relying on market institutions	0	18	51	29
	Relying on education institutions	0	2	7	6
	Relying on social organizations	0	2	1	1
	Relying on Community	0	1	18	2
	Total Voluntary	0	23	77	38
Total		18	306	406	232

During the Basic Fertility Support Stage (1949–1962), the government relied heavily on mandatory instruments (15), with a few hybrid instruments (3) and no voluntary instruments. These mandatory instruments included administrative orders (11), regulations (4), financial subsidies (2), and advocacy activities (1). The dominant use of mandatory instruments reflects the government’s high level of involvement in fertility support policies. At this time, China was operating under a “planned economy,” making mandatory policy instruments the optimal choice.

During the Comprehensive Health Assurance Stage (1963–2013), mandatory policy instruments remained dominant (212), while hybrid instruments (71) and voluntary instruments (23) started to emerge. Specifically, instruments included administrative orders (61), regulations (61), penalties (40), institutional measures (39), policy support (32), and advocacy activities (31). For example, the “Law on the Protection of Women’s Rights and Interests” (2005) required local governments to assist impoverished women and illustrated the use of a hybrid instrument. The post-1978 reforms enhanced the role of the market, reducing reliance on mandates.

During the Diverse Service Support Stage (2013–2021), the use of mandatory (211), hybrid (118), and voluntary (77) instruments became more balanced, further reducing government mandatory involvement. Specifically, key instruments included institutional measures (82), advocacy activities (79), regulations (55), market-based mechanisms (51), and administrative orders (45). For instance, the “Guiding Opinions on Promoting Childcare Services for Children Under Three” (2019) encouraged public-private partnerships in improving childcare facilities in areas with high employment density, which demonstrates the use of a voluntary instrument. However, although voluntary and hybrid policy instruments gained significance, mandatory instruments still had the highest proportion.

In the Holistic Inclusive Support Stage (2021 to present), the systematic use of mandatory (106), hybrid (88), and voluntary (38) policy instruments continue to develop. Primary tools include advocacy activities (43), institutional measures (41), reliance on market institutions (29), and regulations (24). Compared to the previous stage, voluntary and hybrid instruments have grown, promoting collaborative engagement among government, market, and societal actors. For example, the “Guiding Opinions on Improving Supportive Measures for Active Childbirth” (2022) states “employers can implement flexible working hours and remote work to assist employees caring for their children” which is an example of the voluntary policy instrument.

The analysis of policy instruments across four stages shows that China used different instruments to achieve demographic goals. Mandatory instruments remained predominant in all stages, followed by hybrid instruments, with voluntary instruments least utilized. The proportion of mandatory instruments has steadily declined over time, reflecting a shift toward a more balanced approach.

#### 4.3.3. Evolution of content- instruments interaction.

**Fig 3:**
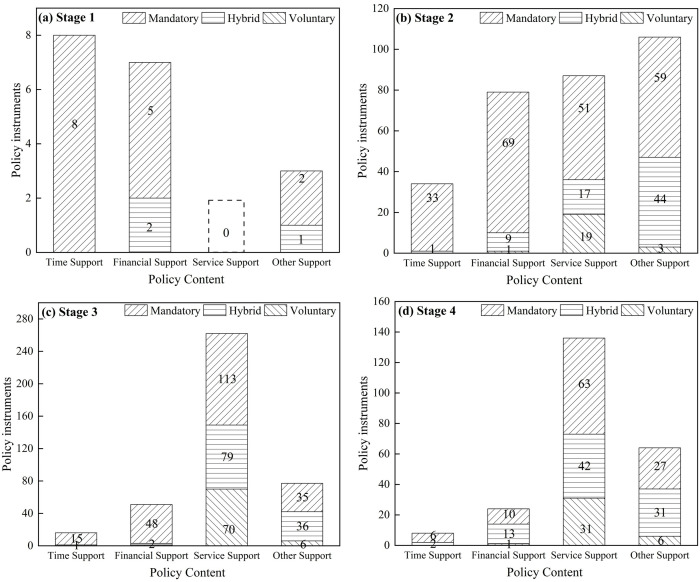
Interaction between policy content and policy instruments across different stages.

Analyzing the interaction between policy content and instruments reveals deeper structural patterns. A two-dimensional framework analysis of 962 policy units shows that across four categories of policy content—time, financial, service, and others—a variety of instruments (mandatory, hybrid, and voluntary) are used, though patterns varied by stage (see Fig 3).

In the Basic Fertility Support Stage (1949–1962), content relied almost exclusively on mandatory instruments. In the Comprehensive Health Assurance Stage (1963–2013), both mandatory and hybrid instruments were widely used with different policy contents, leading to a significant increase in policy complexity. During the Diverse Service Support Stage (2013–2021), mandatory, hybrid, and voluntary instruments interacted with a wider range of contents, reflecting a diversification of content and instrument interactions and growing market involvement. In the Holistic Inclusive Support Stage (2021 to present), interactions become more systematic and balanced, with a focus on the entire process of “fertility, parenting, and education”. The target audience also expanded from “women” to “families”, as reflected in the introduction of housing support.

The application of hybrid policy instruments for financial and other supports has increased significantly since the Comprehensive Health Assurance Stage. Additionally, the use of hybrid and voluntary instruments for service support has risen after the Diverse Service Support Stage. Taking time support as an example, maternity leave in 1951, stipulating “fifty-six days of leave with full salary,” is an administrative order. In contrast, the 2021 policy merely proposed “supporting pilot parental leave for qualified regions” as a general form of policy support. This evolution from mandatory to hybrid instruments in leave provision reflects decreasing government control.

## 5. Discussion

A quantitative analysis of fertility support policy texts reveals that Chinese fertility support policy has gradually evolved in response to historical contexts, shifting policy goals and changing economic and social structures. Our findings provide valuable insights into the key characteristics of this evolution.

### 5.1. Policies have evolved to fit the historical context

Our quantitative analysis identifies major shifts in China’s fertility support policies across different population policy eras. These policies have gradually expanded from basic time support to multidimensional support. Early fertility support policies under the “promote population growth” phase focused narrowly on maternity leave, akin to basic supports in Anglo-Saxon countries [27]. During the “birth-control” era, support remained limited, primarily safeguarding maternal health. Following the emergence of ultra-low fertility after 2015, policies broadened to include childcare services, financial subsidies, and parental leave, aligning more closely with Nordic/Central European models [22,49]. However, gaps remain particularly in paternity leave, childcare provision, and parenting support, where China still lags behind advanced economies.

Regarding policy instruments, the proportion of mandatory instruments has steadily decreased across stages. Mandatory instruments dominated during the planned economy eras. Since 2015, the use of hybrid tools (e.g., tax incentives) and voluntary tools (e.g., market-based services) has increased, reflecting a shift toward market engagement and broader societal participation. Despite the progress, hybrid/voluntary tool adoption especially in service delivery (e.g., public-private childcare partnerships) remains markedly lower compared to Northern/Central Europe countries.

### 5.2. Interaction combinations for enhancing policy effectiveness

Effectiveness of fertility policies depends on the interaction between policy content and instruments interact. As demographic goals change over time, the scope of fertility support policies has expanded. Traditional mandatory instruments such as orders and penalties have become less effective. International evidence suggests that tools engaging market and social actors are more successful in raising fertility [18]. Hybrid tools—such as tax breaks or childcare allowances—can mobilize market resources, support families, and reduce dependence on direct government aid [36].

However, hybrid instruments remain underused in China’s fertility support policies. For example, childcare services are often provided solely by either public or private institutions, restricting innovation and resource integration. Improving integration of policy content and delivery mechanisms has strong potential to increase overall policy impact.

### 5.3. Emphasis on gender equality and market-oriented childcare

The evolving values of Chinese fertility support policies reflects adaptation to women’s needs. Deeply rooted Confucian traditions have long shaped gender roles in China. Today, persistent inequality in both the workplace and household is a key factor behind declining fertility. Policies have shifted from a “family-oriented” approach, which emphasizes traditional caregiving roles, to a “market-oriented” approach seeking to alleviate caregiving burdens.

For decades, China’s fertility support policies have assumed that women are the primary caregivers and focused on extending maternity leave. After the two-child policy, the government introduced various supportive measures, but these have not effectively encouraged childbearing [3]. Domestic and international evidence [31] suggests that extending maternity leave without broader support reinforces motherhood penalties by increasing costs for employers and harming women’s career prospects. Meanwhile, limited paternity leave discourages father involvement, and inadequate childcare services place an outsized burdens on women, reinforcing traditional norms. Current “family-oriented” policies demonstrate significant gender blindness, falling short of modern working women’s needs.

Post-three-child policy implementation, China has expanded childcare services to reduce familial care and begun promoting paternity leave. This reflects an emerging alignment with Nordic-style, market-based strategies for reducing family caregiving burdens and promoting more equitable parenting [32,33]. With rising gender equality awareness and delayed retirement in China, this “market-oriented” approach better suits the needs of modern working women. It also offers a path to disrupting the cycle of motherhood penalties and low fertility intentions. However, current policies remain heavily focused on women, and measures encouraging male participation in childcare such as paternity leave and parental leave was introduced late and remains limited. Moreover, childcare policy lacks practical enforceability, with minimal use of financial subsidies and tax incentives to boost service availability and quality.

## 6. Conclusion and policy implications

### 6.1. Conclusion

This study employs content analysis to construct an analytical framework for understanding the evolution of China’s fertility support policies across two dimensions: policy content and policy instruments. The analysis covers 226 policies documents across four historical stages.

The findings reveal that services and other forms of support outnumber time and financial policies, indicating a gradual diversification of policy content. Furthermore, the frequency of mandatory policy instruments surpasses that of hybrid and voluntary types, though the use of hybrid and voluntary tools has increased, showing a trend toward more balanced utilization. Distinct structural differences exist in the use of policy instruments across different content areas; notably, the number of hybrid policy instruments utilized for financial and other supports significantly increases after the Comprehensive Health Assurance Stage. Similarly, the use of hybrid and voluntary instruments for service support rises markedly following the Diverse Service Support Stage.

The evolution of fertility support policies reflects China’s adaptation to changing demographic conditions and shifting gender roles, thereby enhancing policy efficacy. Through an analysis of this evolution combined with the experiences of developed countries, the study offers evidence-based recommendations for optimizing China’s fertility support framework.

### 6.2. Policy implications

Firstly, it is essential to increase the use of hybrid and voluntary instruments to enhance policy adaptability. The research findings indicate that, although mandatory instruments are still prevalent, the proportion is gradually decreasing. Hybrid and voluntary instruments are better suited to today’s market-driven economy and diverse family needs. These tools blend state guidance with market flexibility [50], helping to mobilize resources efficiently. For instance, as economic constraints remain a critical barrier to fertility for many families in China, international practices [51] such as tax breaks, cash subsidies and housing incentives could be adapted to the local context to improve affordability and encourage childbearing.

Secondly, parental leave and especially paternity leave policies should be further enhanced to promote gender equality. Research findings indicate that current parental leave provisions are limited in scope and insufficient in length, thereby failing to effectively alleviate women’s childcare burdens. The division of childcare responsibilities between parents is a significant factor influencing fertility rates [52]. Extending leave periods and encouraging male involvement in childcare can reduce women’s dual pressures from work and family and enhance childbearing willingness. The government should legislate clear standards for leave duration, enforce compliance, and promote cultural change toward shared parenting. For example, aligning paternity and parental leave policies with international norms and introducing penalties for non-compliance—would reinforce gender equality in both the policy framework and its implementation [23].

Thirdly, promoting the socialization and marketization of childcare services is essential to enhance both quality and accessibility. At present, childcare providers are either governmental or private with little collaboration between the two. While government-run childcare institutions are more affordable, they often lack flexible management, which can limit service quality and the ability to meet diverse needs. In contrast, market-based services tend to be more expensive and vary in quality. A more balanced, market-oriented and socially inclusive approach can expand supply and better meet family needs. The government should actively encourage the involvement of enterprises, communities, and non-profit organizations in the provision of childcare services using voluntary instruments, particularly in some regions with higher demand. At the same time, expanding hybrid tools like subsidies and tax incentives would reduce provider risks and cost burdens, making childcare services more sustainable, diverse, and widely available.

## Supporting information

S1 Appendix 226 original policy documents. (ZIP)

S2 Appendix Entire coded document. (XLSX)
