# Supplementary material for: Mapping the evolution of fertility support policies in China: A content and instrumental analysis
Source: PLoS One. 2025 Oct 9;20(10):e0332137. doi: 10.1371/journal.pone.0332137 (PMC12510515; doi:10.1371/journal.pone.0332137)
Supplement: S1 Appendix — (ZIP) [file pone.0332137.s001.zip › S1 Appendix. 226 original policy documents/103-全国人民代表大会财政经济委员会关于第十一届全国人民代表大会第五次会议主席团交付审议的代表提出的议案审议结果的报告(FBM-CLI-1-187201).docx]

全国人民代表大会财政经济委员会关于第十一届全国人民代表大会第五次会议主席团交付审议的代表提出的议案审议结果的报告

发布部门： 全国人大财政经济委员会

发布日期：2012.10.23

实施日期：2012.10.23

时效性： 现行有效

效力级别： 工作文件

法规类别： 人大议事

全国人民代表大会财政经济委员会关于第十一届全国人民代表大会第五次会议主席团交付审议的代表提出的议案审议结果的报告

（2012年10月26日第十一届全国人民代表大会常务委员会第二十九次会议通过）

全国人民代表大会常务委员会：

第十一届全国人民代表大会第五次会议主席团交付财经委员会审议的代表提出的议案123件，其中要求制定法律的61件、修改法律的60件、开展执法检查的1件、开展立法后评估的1件，将内容相同或相近的合并后，共有71个立法、执法检查及立法后评估项目。议案的内容涵盖宏观调控、市场监管、金融财税、社会保障、劳动关系等方面。

财经委员会高度重视代表议案办理工作，制定了具体的工作方案，对代表议案进行了认真研究，并邀请国务院19个部门负责议案办理工作的同志商议议案答复工作，同时委托相关部门提出议案初步处理意见。财经委员会还就代表关注的部分议案，如建议制定航天法等组成了专题调研组，进行立法论证。在议案办理过程中，通过向领衔代表发函征求意见或当面交换意见、电话沟通等方式，认真听取代表的意见。9月27日，财经委员会召开第70次全体会议，对议案处理意见进行了审议。现将审议结果报告如下：

一、15件议案提出的3个立法项目已提请全国人大常委会审议

1.关于修改劳动合同法的议案8件。

2.关于制定旅游法的议案6件。

3.关于制定资产评估法的议案1件。

二、25件议案提出的9个立法项目已列入十一届全国人大常委会立法规划或2012年立法计划，建议起草单位加快工作进程，尽快报请全国人大常委会审议

要求修改法律的议案19件

4.关于修改消费者权益保护法的议案4件。

5.关于修改广告法的议案6件。

6.关于修改商标法的议案6件。

7.关于修改税收征收管理法的议案2件。

8.关于修改城市房地产管理法的议案1件。

要求制定法律的议案6件

9.关于制定期货法的议案1件。

10.关于制定电信法的议案1件。

11.关于制定行业协会法的议案1件。

12.关于制定住房保障法的议案3件。

三、26件议案提出的16个立法项目确有立法必要，建议有关部门加快立法调研论证工作，争取列入新一届全国人大常委会立法规划

要求修改法律的议案15件

13.关于修改电力法的议案3件。

14.关于修改安全生产法的议案3件。

15.关于修改计量法的议案1件。

16.关于修改会计法的议案1件。

17.关于修改票据法的议案1件。

18.关于修改产品质量法的议案1件。

19.关于修改中小企业促进法的议案1件。

20.关于修改民用航空法的议案1件。

21.关于修改证券法的议案1件。

22.关于修改反不正当竞争法的议案1件。

23.关于修改拍卖法的议案1件。

要求制定法律的议案11件

24.关于制定航天法的议案4件。

25.关于制定物流法的议案1件。

26.关于制定电子政务法的议案1件。

27.关于制定物业管理法的议案3件。

28.关于制定网络交易法、电子商务法的议案2件。

四、55件议案提出的41个立法项目，建议通过现行法律法规的贯彻实施，制定和修改有关法律、行政法规等解决议案所提问题，或对立法开展调研论证

（一）对13件议案提出的9个立法项目，相关法律、行政法规或有关规章已有规定，建议通过加强有关法律法规的实施力度，解决议案所提问题

29.关于修改公司法的议案5件。议案所提问题在土地管理法、城市房地产管理法、公司登记条例等有关法律法规已有明确规定，或可通过制定公司章程解决。

30.关于全国人大常委会就提高我国自主创新能力形成专项决议的议案1件。国家高度重视自主创新能力建设，出台了科学技术进步法、促进科技成果转化法等一系列法律法规和政策措施，建议有关部门认真贯彻法律法规，不断完善政策措施，加大工作力度，继续推进自主创新能力的提高。

31.关于制定民间投资促进法的议案1件。2012年政府工作报告和国务院批转的《2012年深化经济体制改革重点工作意见》中，明确要求有关部门2012年上半年务必出台有关民间投资具体实施政策，建议有关部门抓紧落实，解决议案所提问题。

32.关于修改保险法的议案1件。中国保监会认为，议案所提问题保险法已有规定或可通过订立保险合同加以约定。财经委员会建议中国保监会等有关部门细化完善相关规定，加大监管力度，解决议案所提问题。

33.关于制定进城务工人员权益保护法的议案1件。有关法律、法规和国务院的有关规定对农民工依法享有的权利作了规定，建议人力资源和社会保障部通过贯彻落实有关法律法规和政策，解决农民工在社会保险、工资保障、职业技能培训等保障农民工权益的问题。

34.关于制定教育投入保障法的议案1件。我国教育法、义务教育法等法律对经费投入问题作了规定，建议有关部门通过严格落实现有法律规定，解决议案所提问题。

35.关于制定无障碍建设法的议案1件。《无障碍环境建设条例》已于2012年8月1日正式实施，建议在贯彻实施的基础上，总结经验，待条件成熟时，将条例上升为法律。

36.关于制定小企业金融服务法的议案1件。建议中国人民银行、中国银监会等部门在进一步贯彻落实中小企业促进法的基础上，加大对小微企业的金融扶持力度。

37.关于制定高温行业劳动保护法的议案1件。2012年6月29日，国家安监总局、卫生部、人力资源和社会保障部、全国总工会联合发布了新的《防暑降温措施管理办法》，议案所涉及的问题在办法中已有明确规定。

（二）对25件议案提出的18个立法项目，建议通过制定和修改其他法律或行政法规等解决议案所提问题

38.关于制定金融消费者权益保护法的议案1件。建议通过制定《存款保险条例》、《银行卡条例》、《征信业管理条例》等法规，为相关领域消费者权益保护提供法律保障。

39.关于修改建筑法的议案2件。建议通过制定《建筑市场管理条例》和修改《民用建筑节能管理条例》解决议案所提问题。

40.关于修改台湾同胞投资保护法的议案1件。建议通过修订实施细则解决该法实施中存在的问题。

41.关于修改价格法的议案2件。议案所提建议有些已由相关行政法规、规章或规范性文件作了明确规定，有些可以通过制定配套的法规和规章来解决。

42.关于制定财政转移支付法的议案1件。国务院已将制定《财政转移支付管理暂行条例》列入立法工作计划，鉴于当前制定法律时机和条件还不够成熟，建议先行制定条例解决议案所提问题。

43.关于制定电子支付法的议案1件。建议通过制定《电子支付指引》、《银行卡条例》等有关法规、规章解决议案所提问题。

44.关于制定信息化促进法的议案1件。建议通过制定完善信息安全条例等相关法规、规章，解决议案所提的问题。

45.关于制定家政服务法的议案1件。商务部正在研究制定家政服务业管理办法。建议先通过制定行政法规和规章解决议案所提问题，待条件成熟时，再推进家政服务业立法。

46.关于制定社会信用法的议案1件。建议通过制定《征信业管理条例》、《个人征信信息保护暂行规定》等法规规章解决议案所提问题。

47.关于制定民间借贷法的议案4件。对偶发性民间借贷，应主要依据现有民事、合同等法律予以规范。对小额贷款公司等非存款类放贷组织，建议通过制定相关行政法规予以规范，及时解决议案所提问题。

48.关于制定住宅法的议案1件。住房保障法和城市房地产管理法（修订）已列入十一届全国人大常委会立法规划。建议通过住房保障立法和修改城市房地产管理法解决议案所提问题。

49.关于制定社会保障法的议案2件。我国目前在社会保险、社会救助、特殊群体权益保护等方面已制定了相关法律法规，同时正在稳步推进社会保障法律体系的建立和完善。建议在修改相关法律和制定行政法规的过程中研究吸收代表建议，待条件成熟时，推进社会保障立法。

50.关于制定房屋拆迁法的议案1件。国务院已制定了《国有土地上房屋征收与拆迁补偿条例》，正在研究修订土地管理法，并制定《农民集体所有土地征收补偿安置条例》。建议尽快出台相关行政法规，及时解决议案所提问题。

51.关于制定城市公共交通保障法的议案1件。国务院相关部门正在积极制定《城市公共交通条例》，条例草案已公开征求社会意见，议案所提建议在草案中已有体现。

52.关于制定担保公司监督管理法的议案1件。建议有关部门认真贯彻执行并适时修改完善《融资性担保公司管理暂行办法》，解决好融资性担保存在的问题，同时，对将非融资性担保业务纳入监管的必要性和可行性进行深入研究，结合融资性担保监管经验，提出制定统一的担保行业法律制度的意见。

53.关于制定农村信用法的议案1件。中国人民银行正在制定《征信业管理条例》，其中有关内容涉及农村信用体系建设。

54.关于制定潜水法的议案1件。国务院有关部门非常重视潜水领域的立法工作，建议先通过制定行政法规解决议案所提问题，2008年潜水条例草案已报送国务院，草案的主要内容与议案所提方案基本相同。

55.关于制定工资法的议案2件。建议通过制定企业和机关事业单位工资管理的行政法规，解决议案所提问题。

（三）对17件议案提出的14个立法项目，建议进一步调研论证，同时通过研究制定相关政策，改进工作，解决议案所提问题

56.关于修改社会保险法的议案2件。社会保险法2011年7月1日才正式实施，是否将工伤保险和生育保险纳入基本医疗保险以及确定统一的基本养老费数额，尚需研究论证。

57.关于制定反暴利法的议案1件。建议国家发改委对议案提出的问题和建议进行认真研究，并加强对重点行业的反暴利监管。

58.关于制定经济法的议案1件。我国经济立法采用分类单项立法的方式，实践证明，这种方式适应了经济体制改革和经济社会发展的需要。制定经济法，实现经济法的法典化还需要更多的实践探索。

59.关于修改政府采购法第二十二条的议案1件。政府采购法第二十二条第一款规定的是供应商参加政府采购活动应当具备的基本条件，并未对中小企业参加政府采购进行限制。建议财政部在制定政府采购具体实施办法时认真研究代表的意见，进一步明确中小企业参加政府采购的政策，解决议案提出的问题。

60.关于制定陆地边境地区发展扶持促进法的议案1件。目前国务院已出台兴边富民行动规划，加大了对边境地区的支持力度，建议有关部门认真落实国务院有关政策措施，解决议案所提问题。

61.关于修改企业破产法的议案1件。建议有关方面进一步研究论证代表意见，完善相关司法解释，解决议案所提问题。

62.关于制定公共服务基本法的议案1件。我国初步形成了由单行法律、法规和规章组成的公共服务制度框架，为满足公民基本公共服务需求提供了有效的法律保障。基本公共服务涉及许多方面，不同领域公共服务的对象、标准、模式差异很大，各地区之间保障水平很不平衡，制定统一的公共服务基本法有待研究论证。

63.关于制定集体经济组织法的议案1件。国家对经济组织的划分标准是按照企业投资方式与责任形式，目前大多数城镇和农村集体经济组织的设立与变更均已根据不同情况分别适用公司法、合伙企业法以及农民专业合作社法等。目前是否就集体经济组织进行单项立法，尚需研究。

64.关于修改就业促进法的议案1件。人力资源和社会保障部正在认真研究人力资源市场上存在的年龄歧视等问题，拟及时提出相关建议。建议国务院有关部门研究代表提出的意见，不断完善促进就业的政策措施。

65.关于制定中小企业信用担保法的议案1件。建议中国银监会研究完善中小企业信用担保机制，认真研究吸收议案所提建议。

66.关于制定航空法的议案3件。鉴于相关部门和单位对立法主体、空域管理体制等问题存在较大争议，目前难以制定统一的航空法。建议有关部门和单位深入研究议案所提问题，探索完善我国航空管理制度。

67.关于完善债券市场立法的议案1件。目前债券市场和监管体制改革尚在进一步探索中，立法的时机还不够成熟，财经委员会将认真研究代表意见，根据今后金融市场的改革发展情况适时提出相关立法建议。

68.关于制定互联网/物联网法的议案1件。制定全面规范互联网的基本法，时机尚不成熟；对物联网应用宜采取逐步立法的方式加以解决，建议统筹考虑互联网、物联网领域的实践情况和立法需求。

69.关于制定公物管理法的议案1件。对公物的占有、使用、收益和处分的权利，物权法作了明确具体的规定。目前公物的种类和范围在理论上还存在较大争议，如何统一立法，需要作进一步探索。

五、2件议案提出的1项执法检查和1项立法后评估的建议，建议全国人大常委会在今后研究制定工作计划时统筹安排

70.关于开展车辆保险有关法律法规执法检查的议案1件。建议中国保监会等有关部门严格执法，加强对车险理赔的监督检查。

71.关于开展信托法立法后评估的议案1件。建议有关部门和单位加快信托法配套法规建设，统筹安排立法后评估工作。

以上报告，请审议。

全国人民代表大会财政经济委员会

2012年10月23日

附件：

全国人民代表大会财政经济委员会关于第十一届全国人民代表大会

第五次会议主席团交付审议的代表提出的议案的审议意见

一、15件议案提出的3个立法项目已提请全国人大常委会审议

1．王荣华等30名代表、欧真志等33名代表、秦希燕等32名代表、刘玲等31名代表(第 20号、第168号、第325号、第461号议案)提出，劳动合同法实施以来，各地劳务派遣用工迅猛发展，劳务派遣制度被滥用，造成同工不同酬、劳务派遣管理不完善、异地派遣劳动者社会保险权益保障不明确、被派遣劳动者权益维护不到位等问题，建议修改劳动合同法有关劳务派遣的规定，进一步规范劳务派遣行为。姜健等31名代表、任沁新等31名代表、于文等30名代表、左延安等30名代表(第75号、第299号、第315号、第437号议案)提出，在企业管理实践中发现，劳动合同法部分条款过度向劳动者倾斜，主体“对等”关系失衡，造成企业的管理成本增加和劳动者随意解除劳动合同，同时存在着企业规避法律、劳动者自身维权意识淡薄、政府监督管理不到位等问题，建议修改劳动合同法有关劳动者单方解除劳动合同、同工同酬的认定、企业解除劳动合同的条件、无固定期限劳动合同等规定。人力资源和社会保障部认为，劳动合同法在完善劳动合同制度、明确劳动合同双方权利义务、保护劳动者合法权益、构建和谐劳动关系等方面发挥了重要作用，实施中也出现了一些新问题。违约金、经济补偿、劳动者单方解除劳动合同等问题劳动合同法已作了明确规定，劳动合同法的立法意图是解决实践中存在的劳动合同短期化和用人单位利用优势地位限制劳动者流动的问题，平衡社会负担和用人单位负担，经济补偿年限的计算等问题需进一步明确，建议通过司法解释予以明确；连续两次签订固定期限劳动合同的认定、劳务派遣是否适用无固定期限劳动合同等问题，建议立法机关作出明确规范；强化支付令的规定属于民事诉讼法的内容，建议在修改民事诉讼法时予以研究；同工同酬的认定，该部正在进一步完善有关定义，解决具体操作问题。 2012年6月，十一届全国人大常委会第二十七次会议对劳动合同法修正案草案进行了初次审议。

2．姜健等31名代表、许世辉等31名代表、周晓光等30名代表、刘庆宁等31名代表、周洪宇等30名代表、戴雅萍等34名代表(第79号、第114号、第207号、第240号、第344号、第 463号议案)建议加快制定旅游法，促进旅游业发展、保护旅游者和旅游经营者的合法权益、规范旅游市场秩序、提高旅游服务质量、正确处理旅游资源保护与开发的关系、明确旅游主管部门的职责、加强旅游管理等。财经委员会十分重视旅游立法工作，于2009年12月牵头，组织国家发改委、国务院法制办、国家旅游局等23个部门和有关专家成立了旅游法起草组。2012年3月14日，财经委员会全体会议审议通过了旅游法草案。议案所提意见和建议，大部分在草案中已有所体现。2012年8月，十一届全国人大常委会第二十八次会议对旅游法草案进行了初审。

3．左延安等30名代表(第438号议案)提出，商标资产的价值评估在企业资产重组、股份制改造等资本运营过程中具有重要意义，但目前我国缺乏有关商标评估的法律法规，商标评估机构不规范，商标评估方法不科学、不统一，法律责任不明确，使被评估企业的利益受损，造成不公平的资产交易和竞争，应加强商标评估立法，制定统一的法律规范。国家工商总局认为，为适应加入世贸组织的需要，该局于2011年废止了《企业商标管理若干规定》和《商标评估机构管理暂行办法》。2012年2月，十一届全国人大常委会第二十五次会议初审了资产评估法草案。议案所提商标专用权评估业务、评估机构和评估人员的法律责任等问题在资产评估法草案中已有体现。

二、25件议案提出的9个立法项目已列入十一届全国人大常委会立法规划或2012年立法工作计划，建议起草单位加快工作进程，尽快报请全国人大常委会审议

要求修改法律的议案19件

4．徐景龙等31名代表、应名洪等30名代表、南存辉等30名代表、莫照兰等30名代表 (第6号、第117号、第255号、第385号议案)提出，随着社会经济的快速发展以及新型消费关系、消费形式不断涌现，消费者权益受到侵害的情况日益严重，消费者权益保护法已经明显不能适应当前客观形势的需要，迫切需要修改。国家工商总局认为，消费者权益保护法(修订)已列入十一届全国人大常委会立法规划，2009年该局牵头启动消费者权益保护法的修订工作，2010年将修订草案送审稿报送国务院。财经委员会建议有关部门和单位加快起草工作进度。

5．徐景龙等31名代表、姜健等31名代表、郑捷等30名代表、韦飞燕等30名代表、刘庆宁等31名代表、左延安等30名代表(第55号、第77号、第160号、第237号、第243号、第439号议案)提出，随着市场经济快速发展，广告发布明显增多，利用广告从事违法经营活动日趋严重，虚假广告泛滥，广告法的部分内容及执法工作已不能完全适应规范广告活动、维护广告市场秩序、保护消费者利益、弘扬社会良好风尚的要求，建议修改广告法。国家工商总局认为， 2004年该局启动了广告法的修订工作，并于 2009年将修订草案送审稿报送国务院，议案关注的问题在修订送审稿中有较为充分的体现。广告法(修订)已列入十一届全国人大常委会立法规划，财经委员会建议有关部门抓紧工作，尽快提请审议。

6．姜健等31名代表、戴仲川等30名代表、周晓光等30名代表、邵峰晶等31名代表、俞学文等30名代表、左延安等30名代表(第78号、第115号、第208号、第386号、第396号、第 436号议案)提出，商标法中有关商标申请在先原则、保护注册商标专用权、驰名商标认定条件、商标审查规则等一系列规定已不适应新形势的需要，应及时修改。国家工商总局认为，2003年该局启动了商标法第三次修改工作，2009年将修订草案送审稿报送国务院，2011年国务院法制办向社会公开征求意见。议案提出的有关驰名商标、恶意抢注、简化异议程序、自行改变注册商标等问题在修订草案中有较为充分的体现。商标法(修订)已列入十一届全国人大常委会立法规划，财经委员会建议尽快完善修订草案，争取早日提请审议。

7．周晓光等30名代表、蔡奇等30名代表 (第206号、第257号议案)建议修改税收征收管理法。国家税务总局认为，议案提出的关于明确税收登记的法律地位、扩大税务登记的适用范围、完善税收登记注销制度、建立涉税信息共享机制、加快信息化建设、明确电子申报资料法律效力、确立纳税评估法律地位、明确有关部门报送涉税信息法律义务等建议，对完善税收征管制度具有重要意义，将在起草税收征收管理法修正案草案时研究吸收。税收征收管理法(修订)已列入十一届全国人大常委会立法规划，财经委员会建议认真研究议案所提建议，加快起草工作，争取早日提请审议。

8．杜波等30名代表(第366号议案)提出，城市房地产管理法滞后于房地产业的发展，建议修改。住房和城乡建设部认为，城市房地产管理法(修订)已列入十一届全国人大常委会立法规划，目前已形成城市房地产管理法修订草案稿，议案所提问题在草案中已有所体现。财经委员会建议有关部门加强协调，争取早日提请审议。

要求制定法律的议案6件

9．朱玉辰等30名代表(第24号议案)提出，随着我国期货市场的日益壮大，现有期货市场规则体系缺乏基础法律支撑和指导的弊端日益显现，已不能完全满足市场的深层次发展需要，尤其是不能满足金融衍生品市场的发展需要，建议制定期货法。期货法已列入十一届全国人大常委会立法规划，财经委员会会同中国证监会等部门开展起草工作，形成了草案稿，将在总结股指期货实施经验的基础上，进一步修改完善后提请审议。

10．徐景龙等31名代表(第58号议案)提出，电信条例不能满足行业的实际需求，法律效力较弱，规定过于原则，缺乏明确的违规处罚细则，建议制定电信法。工业和信息化部认为，原信息产业部承担了电信法的起草工作，并于 2004年7月将电信法草案送审稿报送国务院。议案提出的电信市场准入、互连互通、电信普遍服务、三网融合、电信市场竞争监管、用户信息保护、电信监管体系等方面的具体立法建议，对于完善电信法草案具有很好的参考价值。电信法已列入十一届全国人大常委会立法规划，财经委员会建议有关部门结合议案内容，尽快完善草案，争取早日提请审议。

11．王晶等30名代表(第276号议案)提出，我国各地、各行业陆续出现了很多行业性组织，但缺乏统一规范行业协会的法律，影响了行业协会健康发展，容易造成行业协会行为不规范、结构不合理等问题，建议尽快出台行业协会法。民政部认为，我国已基本形成了覆盖国民经济各个门类的行业协会体系，行业协会在经济社会发展中发挥着越来越重要的作用，但也存在着布局结构不尽合理、职能定位不明确、作用发挥不明显等问题，应加强相应立法。行业协会商会法已列入十一届全国人大常委会立法规划，财经委员会建议有关部门认真研究代表建议，加快立法工作进程。

12．蔡奇等30名代表、刘卫星等30名代表、金志国等30名代表(第259号、第300号、第388号议案)提出，我国住房保障体系不够完善，保障对象不够明确，保障方式不够统一，保障责任不够落实，收入核定和资格认定困难，退出机制不够完善，需要加强住房保障立法。住房和城乡建设部认为，议案对我国目前住房保障现状及立法基础的分析十分中肯，现阶段开展住房保障相关立法确有其必要性。住房保障法已列入十一届全国人大常委会立法规划，财经委员会建议有关部门认真研究代表建议，加快相关立法工作进程。

三、26件议案提出的16个立法项目确有立法必要，建议有关部门加快立法调研论证工作，争取列入新一届全国人大常委会立法规划

要求修改法律的议案15件

13．褚君浩等30名代表、李新炎等31名代表、南存辉等30名代表(第19号、第41号、第252号议案)提出，随着国内经济发展及整个社会用电需求和结构的变化，以及新能源和可再生能源供应的迅猛发展，电力法中部分条款不适应新型发电技术发展的需要，为了推广可再生能源的应用，建议修改电力法。国家能源局认为，议案所提建议对修改电力法具有重要参考价值，该局将积极推进电力法修改工作，同时通过开展试点项目建设、促进低电压等级电网开放，为修订电力法积累经验。财经委员会建议有关部门认真研究代表建议，积极开展调研论证，争取将其列入全国人大常委会立法规划。

14．戎光道等31名代表、杨庚宇等32名代表、蔡奇等31名代表(第22号、第227号、第 258号议案)建议修改安全生产法，完善安全生产方针、建立健全安全生产管理体系、明确政府职责、建立政府安全生产业绩考核指标体系、加大安全生产投入保障、明确注册安全工程师的法律地位、推行安全生产责任强制保险、加大处罚力度等。国家安监总局认为，国务院高度重视安全生产法的修订工作，该局已将修订草案送审稿报送国务院。财经委员会建议有关部门认真研究吸收代表建议，加快安全生产法修订工作进程。

15．章联生等30名代表(第39号议案)建议修改计量法与WTO规则不符合的部分条款。国家质检总局认为，随着社会主义市场经济体制的建立和科学技术的发展，计量法部分规定已不能满足实际需要，确有修改必要。该局于2000年启动了修订工作，于2005年和2010年两次将修订草案送审稿报送国务院。议案关于实行计量检定和计量校准并行的量传溯源方式、对进口计量器具开展型式批准、加强对商品量的监管等建议，将在下一步立法工作中加以完善。财经委员会同意国家质检总局的意见，建议相关部门认真研究议案所提建议，积极开展法律修订工作，争取将其列入全国人大常委会立法规划。

16．徐景龙等31名代表(第59号议案)提出，会计法规定的违法行为情节轻重不分，行政处罚自由裁量权过大，单位负责人的法律责任缺失，建议修改。财政部认为，会计法修订已列入该部立法工作计划，正积极开展调研，建议将会计法修订列入全国人大常委会立法规划。财经委员会同意财政部的意见。

17．么志义等32名代表(第122号议案)提出，近年来空白背书引发的法律纠纷案件逐年上升，票据法原则上只承认记名背书，没有明确规定空白背书的法律效力，建议修改票据法。中国人民银行认为，票据法确需修改，对于代表所提意见，将在研究修改过程中综合权衡考虑。财经委员会同意中国人民银行的意见，建议进一步研究论证，适时提出立法建议。

18．南存辉等30名代表(第253号议案)提出，某些行业的中国国家标准低于发达国家标准，一些外国品牌对华销售产品时，采取“双重标准”，损害了中国消费者合法权益，建议修改产品质量法，规定缺陷产品召回制度，并明确规定进口商品应明示该产品所适用的标准。国家质检总局赞成在产品质量法的修订中增加产品召回制度的规定；对于“双重标准”的问题，可通过提高我国标准水平、采用国外先进标准等方式予以解决，建议在产品质量法修订中进一步完善产品标识管理。财经委员会同意质检总局的意见，建议抓紧开展法律修订准备工作，争取列入全国人大常委会立法规划。

19．金颖颖等30名代表(第256号议案)提出，中小企业促进法已不能完全适应目前经济形势变化与支持中小企业继续发展的需要，有必要对其进行修订。财经委员会认为，中小企业发展还面临着相当的困难和挑战，有必要通过修改中小企业促进法，鼓励创新，促进创业，创造就业机会，建议将其列入全国人大常委会立法规划。

20．吴江林等30名代表(第279号议案)提出，目前我国民航航班延误、托运行李遗失事件频频发生，缺乏投诉渠道和受理部门，旅客索赔无门，建议修改民用航空法关于航班延误、行李遗失等相关规定。中国民用航空局认为，近年来该局结合行业发展，已启动民用航空法的修改工作，研究完善承运人损害赔偿责任制度。财经委员会同意中国民用航空局的意见，建议抓紧法律修订工作。

21．欧阳泽华等31名代表(第303号议案)提出，目前证券法与市场改革发展实际不相适应，特别是“十二五”规划确定的加快转变经济发展方式、加强法治政府建设、提高对外开放水平、创新社会管理方式等目标和任务，对资本市场法治建设包括资本市场功能定位、运行机制和监管执法等方面提出了更高要求，建议修订证券法。财经委员会认为，议案所提建议值得认真研究，有必要对证券法进行修改，建议将证券法修订列入全国人大常委会立法规划。

22．杨伟程等33名代表(第367号议案)提出，反不正当竞争法缺乏对新出现的不正当竞争行为的规定，现有的一些规定较为笼统、缺乏操作性，难以适应市场发展的需要，应抓紧修订完善反不正当竞争法。国家工商总局认为，为更好地维护公平竞争市场秩序、促进统一开放竞争有序的现代市场体系的建立，修改反不正当竞争法十分必要。2003年该局进行修订工作，于 2008年将修订草案送审稿报送国务院，议案所提不正当竞争行为的一般性条款、细化与补充不正当竞争行为的种类、利用网络的不正当竞争行为等建议在草案中已有较充分的体现。财经委员会建议抓紧草案修订工作，争取列入全国人大常委会立法规划。

23．王法亮等30名代表(第383号议案)提出，近年来，我国拍卖行业发展迅猛，拍卖企业迅速增加，年成交额成十倍增长，同时存在“拍假”“假拍”盛行、信息披露不透明、政府部门监管缺位、诚信规则缺失等问题，建议修改拍卖法。商务部认为，议案所提意见合理，今后将在修改完善拍卖法时认真研究。财经委员会同意商务部的意见，建议抓紧拍卖法修订准备工作，争取列入全国人大常委会立法规划。

要求制定法律的议案11件

24．徐景龙等30名代表、李朋德等31名代表、胡浩等31名代表、彭小枫等35名代表(第 62号、第127号、第132号、第489号议案)提出，我国航天事业自1956年创建以来，取得了一系列辉煌成就，走出了具有中国特色的自主创新发展道路。但我国航天立法工作严重滞后，目前只有少数单行法规和规定，存在法律效力等级较低、立法空白点多、可操作性较差等问题，制约了航天事业的健康发展，建议尽快制定航天法。国防科工局认为航天法立法十分必要和紧迫，加快航天立法是维护国家安全和外空权益的需要、是保障航天活动规范有序开展的需要、是切实履行国际义务和有序开展国际合作的需要、是推动航天事业可持续性发展的需要。建议全国人大发挥主导作用，积极推动立法工作。解放军总装备部认为，该部十分重视航天装备立法工作，已形成了一系列规章制度，为航天立法奠定了基础，下一步将积极参加立法工作。财经委员会同意以上意见，并已启动立法调研论证工作，在立法必要性和一些重要问题上与有关单位已达成共识，建议将该法列入全国人大常委会立法规划。

25．朱慧秋等30名代表(第67号议案)提出，我国物流法律的不完善已经成为现代物流业健康、快速和可持续发展的瓶颈，建议制定物流法。国家发改委认为，制定物流法很有必要，将从现有法律法规及规章入手，对物流业的产业概念和边界、管理体制、准入条件、鼓励政策、以及与现行有关法律法规的衔接问题，进行深入研究论证，会同有关部门先行开展前期立法调研，建议将物流法列入全国人大常委会立法规划。财经委员会同意国家发改委的意见。

26．戴仲川等30名代表(第113号议案)提出，我国传统政务运行环境和模式由现实世界延伸到虚拟空间，电子政务建设取得了很大的成绩，现行法律难以适应电子政务的发展，存在着电子政务网络重复建设、结构不合理、业务系统水平低、应用和服务领域窄、信息资源开发利用滞后，互联互通不畅、标准不统一、有安全隐患等问题，建议制定电子政务法。国务院办公厅认为，随着电子政务的快速发展，我国电子政务立法工作不断加强，颁布了电子签名法、政府信息公开条例等法律法规，地方政府出台了一批地方性电子政务规章规定，但现阶段我国电子政务的发展仍存在体制机制的深层次问题，确需研究制定电子政务法。国务院有关部门已开展了立法前期调研，形成了初步研究成果，立法的条件日趋成熟，建议将电子政务法纳入人大立法计划。财经委员会同意国务院办公厅意见，建议将该法列入全国人大常委会立法规划。

27．许金和等31名代表、南存辉等30名代表、任玉奇等30名代表(第161号、第254号、第338号议案)提出，目前物业管理不规范，矛盾纠纷多发，房地产开发商、业主、物业管理企业之间的责权利不明确，建议制定物业管理法。住房和城乡建设部认为，议案所提问题在实践中确实存在，制约了物业管理行业的健康发展和物业管理服务水平的提高。该部将在贯彻落实物业管理条例的基础上，全面系统地梳理物业服务中存在的问题，适时提出将物业管理条例上升为法律的建议。财经委员会建议将该法列入全国人大常委会立法规划。

28．周晓光等30名代表、周晓光等30名代表(第209号、第212号议案)提出，目前，我国尚缺乏一部专门针对网络交易的法律，现有规定比较分散，已制定的相关法规、规章也存在效力层级不高、体系不清、内容不全等突出问题，不能适应电子商务日益发展的现实需要。建议制定网络交易法和电子商务法。商务部认为，电子商务可以涵盖网络交易内容，出台电子商务法有利于电子商务持续健康发展，规范网络交易行为，更好地保护消费者权益和知识产权，有利于防范交易风险和明确各部门职责，建议将其纳入全国人大常委会立法规划并尽快启动立法工作。财经委员会同意商务部的意见。

四、55件议案提出的41个立法项目，建议通过现行法律法规的贯彻实施，制定和修改有关法律、行政法规等解决议案所提问题，或对立法开展调研论证

(一)对13件议案提出的9个立法项目，相关法律、行政法规或有关规章已有规定，建议通过加强有关法律法规的实施力度，解决议案所提问题

29．海南代表团、赖鞍山等30名代表、王文京等31名代表、易昕等36名代表、杨天夫等 30名代表(第3号、第112号、第136号、第 156号、第232号议案)提出，随着社会经济发展环境的变化，公司法的部分条款已经不适应新形势的需要，建议修改公司法，增加股权转让和恶意兼并的规定，明确股东出资不实的连带责任和优先股制度，规范公司登记和年检。国务院法制办认为，涉及土地使用权的股权转让属于法律执行和具体监管问题，土地管理法、城市房地产管理法等有关法律法规已有明确规定；股东优先购买权的行使应以自愿协商为原则，也可以在公司章程中作出规定；股权转让时股东优先购买权在公司法中已有规定，一定程度上可防范恶意兼并，对公司职工代表大会恶意兼并否决权问题，有待进一步研究；股东出资不实的连带责任在公司法中已有明确规定，该办工会同有关部门研究延长发起人股份禁售期的问题；公司法虽未系统规定优先股制度，但允许公司章程另行规定；各地工商部门查询工商档案做法不一问题，可由国家工商总局根据公司登记条例制定统一的规定加以解决，财经委员会同意国务院法制办的意见。

30．史贵禄等30名代表(第126号议案)提出，我国自主创新力还不强，高科技产业的商业模式、服务方式和技术大都来自于国外，这种状况对我国创新经济发展十分不利，迫切需要制定国家自主创新法，建议全国人大常委会就提高我国自主创新能力形成专项决议。科技部认为，改革开放以来，我国颁布实施了科学技术进步法、促进科技成果转化法、科学技术普及法等一系列促进创新的法律，企业所得税法、政府采购法等相关法律中也规定了促进保障创新的措施，各地方也相应制定了旨在促进创新的科技进步条例和其他地方性法规。科技部建议深化科技体制改革，加快创新体系建设，根据经济社会发展需要，不断修订和完善自主创新法律制度。财经委员会同意科技部的意见，建议有关部门认真贯彻法律法规，不断完善政策措施，加大工作力度，继续推进自主创新能力的提高。

31．周晓光等30名代表(第210号议案)提出，我国民间投资已成为促进经济增长、调整产业结构、繁荣城乡市场、扩大社会就业的重要力量，尽管国家放宽了对民间资本进入行业和领域的限制，然而一些地方政府监管有余、服务不足，加之部分地方政府信用约束不够、政策不稳定或不连续，使得一些进入垄断行业的民间资本处境尴尬，甚至不得不被迫退出，建议制定民间投资促进法。国家发改委认为，国家高度重视通过政策法规手段促进民间投资健康发展，但民间投资发展仍面临一些难题，议案提出的准入难问题尚未得到根本解决。2012年政府工作报告和国务院批转的《2012年深化经济体制改革重点工作意见》中，明确要求各部门2012年上半年务必出台有关民间投资实施细则，相关部门已按要求，报送了详细工作计划。国家发改委将继续推动出台民间投资发展实施细则并督促落实，在总结实践经验基础上，积极开展调研，听取各方意见，深入研究各国经验和做法，提高政策措施的针对性、科学性和系统性，为民间投资发展营造更好的法制环境。财经委员会同意国家发改委意见。

32．刘庆宁等31名代表(第239号议案)提出，保险法第十四条关于保险合同生效时间和第六十条被保险人代位求偿权的规定在实务中容易产生纠纷，建议修改保险法。中国保监会认为，第十四条中保险费的交付和保险责任开始的时间，可以特别约定，对先交付保险费、后签发保险单其间发生保险事故的保险责任，应根据投保单或订立合同时的约定来认定；第六十条中，保险事故发生后，被保险人有权向保险人请求保险赔偿，保险人赔偿后，对第三者行使代位求偿权。财经委员会建议中国保监会等有关部门细化完善相关规定，加大监管力度，解决议案所提问题。

33．刘卫星等31名代表(第294号议案)提出，目前我国缺少对进城务工人员在城市的就业、子女上学、养老统筹、医疗保险、保障住房等合法权益的法律保护，建议制定进城务工人员权益保护法。人力资源和社会保障部认为，有关法律法规和国务院规定对农民工依法享有的权利作了规定，该部将通过贯彻落实有关法律法规和政策，解决农民工社会保险、工资保障、职业技能培训等保障农民工权益的问题，同时积极配合有关部门研究解决农民工子女教育、组织制度等问题。财经委员会同意人力资源和社会保障部的意见。

34．周洪宇等30名代表(第343号议案)提出，我国教育经费总量不足、投入不均、投入渠道不畅，建议制定教育投入保障法，合理划分政府的教育投入责任、完善各级教育预算决策程序、优化教育投入结构、拓宽教育经费来源渠道、对教育投入实行严格考核和问责等。财政部认为，我国教育法、义务教育法等法律，对经费投入问题作了规定，在目前财税体制改革深入推进的情况下，单独制定教育投入保障法存在较多困难，建议进一步做好现行法律法规的贯彻落实。财经委员会同意财政部的意见，建议严格依法办事，解决议案关注的问题。

35．袁敬华等31名代表(第355号议案)提出，我国大部分城市的道路、公共建筑、居住小区、公共交通未进行无障碍改造，一些新建设施不符合无障碍规范，已建设施管理亟待加强，信息交流无障碍设施还较为薄弱，全社会无障碍环境尚未形成，建议制定无障碍建设法。中国残疾人联合会认为，国务院已审议通过了《无障碍建设条例》，并于今年8月1日起正式实施，建议在贯彻实施的基础上，总结经验，待条件成熟时，将条例上升为法律。财经委员会同意上述意见。

36．程惠芳等36名代表(第398号议案)提出，需要制定小企业金融服务法，从根本上解决小型微型企业“融资难”、“融资贵”问题。中国人民银行认为，中小企业尤其是小微企业是社会主义市场经济体系的重要组成部分，但长期影响和制约中小企业发展的融资难问题仍没有得到根本解决，中小企业金融服务法律法规仍不健全，有必要修改中小企业促进法或制定小企业金融服务法，为小企业融资提供法律保障。财经委员会同意中国人民银行的意见，建议有关部门在进一步贯彻实施中小企业促进法的基础上，加大对小微企业的金融扶持力度。

37．傅企平等33名代表(第399号议案)提出，《防暑降温措施暂行办法》已不能适应形势发展的需要，建议制定高温行业劳动保护法，明确高温保护的对象和时间，规定高温期间施工的劳动保护措施和高温作业的补贴标准以及高温作业主体单位的责任等。国家安监总局认为，该局和卫生部、人力资源社会保障部、全国总工会等四部门已着手修订《防暑降温措施暂行办法》，并已起草出修订征求意见稿，拟以四部门名义联合印发，待时机成熟后，建议国务院再制定行政法规。财经委员会同意国家安全监管总局的意见。2012年6月29日，国家安全监管总局、卫生部、人力资源社会保障部、全国总工会联合发布了《防暑降温措施管理办法》，《防暑降温措施暂行办法》废止。

(二)对25件议案提出的18个立法项目，建议通过制定和修改其他法律或行政法规等解决议案所提问题

38．林荫茂等30名代表(第21号议案)提出，当前侵害金融消费者权益的现象多发频发，现有的消费者权益保护法等法律法规对金融消费者权益保护不足，建议制定金融消费者权益保护法。中国人民银行认为，金融消费者权益保护涉及人民银行、银监会、证监会、保监会等多个部门，与多部法律存在交叉，实践经验也有待积累，制定专门法律的条件尚不成熟。该行将着力推动《存款保险条例》、《银行卡条例》、《征信业管理条例》等法规出台，为相关领域消费者权益保护提供法律保障。财经委员会同意中国人民银行的意见，建议抓紧制定相关法规、规章，及时解决议案所提问题。

39．徐征等30名代表、周晓光等30名代表 (第23号、第205号议案)提出，建筑法已不能适应规范建设活动和建筑市场秩序的需要，缺乏对低碳建筑的规范，需要进行修改。住房和城乡建设部认为，对议案所提的建筑市场和低碳建筑问题，将通过起草《建筑市场管理条例》和修改《民用建筑节能管理条例》解决。财经委员会同意住房和城乡建设部的意见。

40．张秀娟等30名代表(第35号议案)提出，台湾同胞投资保护法已不能适应新形势发展的需要，建议修改。商务部认为，代表议案中的具体意见，准确反映了新形势发展的需要，也有前瞻性，具有很高的参考价值。商务部高度重视台胞投资保护工作，已经启动修改台湾同胞投资保护法实施细则的研究工作，将加强调研，总结经验，积极与有关部门沟通，争取尽快启动修订工作。财经委员会同意商务部的意见。

41．徐景龙等31名代表、赖鞍山等33名代表(第56号、第111号议案)提出，价格法存在与WTO规则相冲突、表述不恰当、内容不完善等问题，建议修改价格法，进一步规定与基本价格制度相适应的定价形式、把“规定限价”纳入政府指导价范畴、明确市县价格主管部门价格管理权限、对中介服务收费作出更灵活规定、明确行业组织价格自律的定位、明确价格听证会消费者代表构成比例和聘请办法要求、细化法律责任等。国家发改委认为，议案所提建议，有些已由相关法规规章或规范性文件作了明确规定，有些需要在修改价格法时认真研究，建议先就个别项目出台配套的法规和规章，再适时修订相关行政法规、规章和规范性文件。财经委员会同意国家发改委的意见。

42．徐景龙等31名代表(第57号议案)建议制定财政转移支付法，明确财政转移支付的概念和分类，确定财政转移支付的主要目标，科学划分中央和地方政府的事权，确定各级政府之间的收入分配，确定财政转移支付拨款的程序和拨款额的计算方式，建立转移支付管理监督体系。财政部赞同议案提出的完善财政转移支付法律制度的意见，认为当前立法时机和条件还不够成熟，宜先行制定《财政转移支付管理暂行条例》，国务院已将该条例列入立法工作计划。财经委员会同意财政部的意见。

43．徐龙等33名代表(第73号议案)提出，近年来我国电子支付和电子商务快速发展，但电子支付相关规定法律层级较低，无法明确相关主体权利义务关系，为保障支付交易安全，促进电子商务的健康发展，需要制定电子支付法。中国人民银行认为，完善电子支付立法，有利于加强电子支付领域监管，但目前出台法律的条件尚不成熟。为维护支付服务的正常运营和金融体系稳定，该行正在开展《电子支付指引》、《银行卡条例》等起草和准备工作。财经委员会同意中国人民银行的意见。

44．王东洲等36名代表(第180号议案)提出，当前信息化的发展面临着规划不统一、资源开发不足、产业自主创新能力不足、安全问题突出、管理体制和机制改革滞后等问题，建议制定信息化促进法。工业和信息化部认为，国务院高度重视信息化工作，制定出台了一系列方针政策，对信息化发展的各项任务作出了系统安排。该部正起草信息安全条例等相关行政法规和规章，积极推进信息化立法进程。财经委员会建议通过制定完善相关法规、规章，解决议案所提的问题。

45．周晓光等30名代表(第203号议案)提出，随着我国经济快速发展、人民生活水平大幅提高、人口的老龄化、家庭规模的小型化、计划生育政策的实施，对家政服务的需求越来越大，但家政服务人员劳动就业和社会保障等方面的法律法规不健全，家政市场管理混乱，家政服务人员缺乏培训，家政服务纠纷处理机制不完善，建议制定家政服务法。商务部认为，《国务院关于加快发展服务业的若干意见》、《国务院办公厅关于加快发展服务业若干政策措施的实施意见》、《国务院办公厅关于发展家庭服务业的指导意见》等文件的出台，为家政服务业加快发展奠定了良好的政策基础，目前该部正在研究制定家政服务业管理办法。财经委员会建议先通过制定行政法规和规章解决议案所提问题，待条件成熟时，再推进家政服务业立法。

46．周晓光等30名代表(第204号议案)提出，当前社会诚信缺失，严重破坏市场环境和社会秩序，需要制定社会信用法。中国人民银行认为，现阶段社会各界对信用信息的概念仍有不同理解，在保护个人隐私、企业商业秘密和扩大信用公开等方面仍存较大争议，制定统一的社会信用法时机尚不成熟。目前，该行正在推动制定《征信业管理条例》、《个人征信信息保护暂行规定》等法规规章。财经委员会同意中国人民银行的意见。

47．周晓光等30名代表、杨成涛等30名代表、刘卫星等30名代表、刘玲等31名代表(第 211号、第251号、第298号、第464号议案)提出，我国民间借贷市场规模很大，但长期游离于正规金融监管之外，缺乏相应法律法规的引导、规范和监管，风险不易监控，易滋生非法集资、洗钱犯罪等问题，需要制定民间借贷法。中国人民银行认为，对小额贷款公司等非存款类放贷组织，目前该行工会同有关部门起草相关行政法规；对不以放贷为常业的偶发性民间借贷，则可以主要依据现有民事、合同等法律予以规范。财经委员会同意中国人民银行的意见，建议抓紧制定相关法规，及时解决议案所提问题。

48．郑功成等31名代表(第231号议案)提出，住宅是人民生活的必需品，居住权是人民的基本权益，健康的住房保障制度与理性的房地产市场都迫切需要制定住宅法。住房和城乡建设部认为，随着住房制度改革和市场经济发展，有必要加强住宅专项立法，议案所提内容可以通过制定住房保障法和修改城市房地产管理法来解决。住房保障法和城市房地产管理法(修订)已列入十一届全国人大常委会立法规划，立法工作正在进行当中。财经委员会同意住房和城乡建设部的意见。

49．章联生等33名代表、邵峰晶等31名代表(第274号、第387号议案)提出，我国没有一部统领各种社会保障方面的法律，现有社会保障法律体系涵盖领域窄，覆盖面比较小，建议制定社会保障法。人力资源和社会保障部认为，在社会保险方面，我国已制定了社会保险法和相关配套行政法规；在社会救助方面，制定了城市生活无着的流浪乞讨人员救助管理办法、法律援助条例、自然灾害救助条例、城市居民最低生活保障条例等；在保护特殊群体权益方面，制定了残疾人保障法、未成年人保护法、妇女权益保障法、老年人权益保障法、预防未成年人犯罪法等法律。老年人权益保障法正在修订中；社会救助法(草案)经国务院常务会议两次审议；基本医疗保险条例、失业保险条例(修订)等已列入国务院2012年立法工作计划，正在稳步推进。下一步，将积极研究并推进社会保障法的立法进程，财经委员会同意人力资源和社会保障部的意见。

50．王晶等30名代表(第277号议案)提出，随着城镇化建设发展，房屋拆迁矛盾日益突显，物权法及有关法律法规的规定操作性不强，建议制定房屋拆迁法。住房和城乡建设部认为，根据城市房地产管理法，国务院已制定了《国有土地上房屋征收与拆迁补偿条例》；集体土地上房屋征收适用土地管理法及其实施条例，目前国务院正在研究修订土地管理法，并制定《农民集体所有土地征收补偿安置条例》，规范集体土地房屋征收补偿活动。财经委员会同意住房和城乡建设部的意见，建议尽快制定完善相关法律法规，及时解决议案所提问题。

51．刘卫星等30名代表(第293号议案)提出，我国城市公共交通发展存在拥堵现象严重、规划布局不合理、基础设施供给不足、信息网络系统发展滞后、财政公交支出比例偏低等问题，建议制定城市公共交通保障法。交通运输部认为，加快城市公共交通立法进程、促进城市公共交通优先发展、缓解城市交通拥堵十分必要和迫切，但直接出台城市公共交通保障法的难度较大，该部正积极配合国务院法制办制定《城市公共交通条例》，条例草案已公开征求社会意见。财经委员会建议在条例草案的修改完善工作中认真吸收代表意见，解决议案所提问题。

52．刘卫星等30名代表(第295号议案)提出，《融资性担保公司管理暂行办法》人为地将担保公司分为融资性担保和非融资性担保，将非融资性的担保公司排除在监管之外，当前担保公司超范围经营甚至非法经营现象突出，扰乱金融秩序，影响宏观调控，冲击实体经济，加剧了中小企业融资难和融资贵；建议制定担保公司监督管理法，明确担保公司的形式、设立、变更、终止和经营范围，设立经营规则和内部合规控制，规定监督管理。中国银监会征求国家发改委、工业和信息化部、财政部、商务部、工商总局、国务院法制办的意见后认为，担保机构是专业经营担保业务的中介机构，融资性担保业务更具有高杠杆、高风险的特点，要求机构具备一定实力和信用等条件，具备风险识别、风险控制等专业技术和能力，满足风险控制体系、业务标准和专业人员等一系列要求；《融资性担保公司管理暂行办法》旨在对融资性担保公司进行全面规范，在规范机构行为、促进机构发展方面发挥了重要作用；建议有关部门牵头就担保机构立法问题进行研究论证。财经委员会建议有关部门认真贯彻执行并适时修改完善《融资性担保公司管理暂行办法》，解决好融资性担保存在的问题，同时对将非融资性担保业务纳入监管的必要性和可行性进行深入研究，结合融资性担保监管经验，提出制定统一的担保行业法律制度的意见。

53．金硕仁等30名代表(第309号议案)提出，农村信用体系是我国信用体系建设的薄弱环节，缺乏统一和有针对性的法律规范与约束，建议制定农村信用法。中国人民银行认为，农村信用立法需要和我国社会信用体系整体法律制度建设统筹考虑，立法周期长，难以解决农村信用体系对立法的迫切需求。正在起草的征信业管理条例草案将对农村信用体系建设起到积极促进作用。财经委员会同意中国人民银行的意见。

54．许振超等34名代表(第352号议案)提出，潜水是高危特殊行业，我国潜水立法尚属空白，缺少潜水及水下作业技术规则，无法建立与发达国家潜水从业资格资质对等互认机制，潜水人员的健康与人身安全得不到根本保障，建议制定潜水法。交通运输部非常重视潜水领域的立法工作，认为可以先通过制定行政法规解决议案所提问题，2002年该部启动了潜水条例草案的起草工作。2008年将条例草案报送国务院，草案的主要内容与议案所提立法方案基本相同。财经委员会同意交通运输部的意见。

55．袁敬华等30名代表、俞学文等30名代表(第384号、第397号议案)提出，当前我国工资收入分配领域存在着分配机制不健全、分配秩序不规范、同工不同酬、地区和行业之间收入差距过大的突出问题，建议制定工资法。人力资源和社会保障部认为，工资收入分配作为重要民生内容，应予立法规范，制定工资法是深化工资收入分配制度改革、规范工资收入分配行为、促进社会公平的重要制度保障。该部正在收集相关资料、开展调研，为起草企业工资管理方面的行政法规做好准备工作；公务员法就公务员工资问题作了原则规定，正在抓紧研究制定配套法规；同时抓紧研究制定深化事业单位收入分配制度改革的工作方案。待上述关于企业和机关事业单位工资方面的法规出台并取得实践经验后，将适时启动工资法起草工作。财经委员会同意人力资源和社会保障部的意见。

(三)对17件议案提出的14个立法项目，建议进一步调研论证，同时通过研究制定相关政策，改进工作，解决议案所提问题

56．黄志明等31名代表(第96号议案)提出，基本医疗保险、工伤保险和生育保险还没有实现全面的规范与统一，给实际操作带来了诸多不便，影响了社会保障体制的完善，建议修改社会保险法，将工伤保险和生育保险险费的缴纳及其相关法律条款纳入基本医疗保险。张雅英等 30名代表(第408号议案)提出，社会保险法根据用人单位的工资总额按比例缴纳基本养老保险费的规定，使用人单位提高员工工资的成本和风险大幅度增加，极大地影响了用人单位提高工资的意愿和能力，同时会造成退休后待遇不平等，建议修改社会保险法第十二条，规定国家或各省、自治区、直辖市每年确定全国或地区统一的基本养老费数额。人力资源和社会保障部认为，代表所提意见值得高度重视，鉴于社会保险法2011年7月1日才正式实施，在加大法律实施力度的同时，对工伤保险和生育保险纳入基本医疗保险的建议深入研究论证；确定统一的基本养老费数额的建议，会影响社会保险统筹互济原则的实现和现有法律法规体系。财经委员会同意人力资源和社会保障部的意见。

57．史贵禄等30名代表(第128号议案)提出，现实生活中的暴利现象屡见不鲜，《制止牟取暴利的暂行规定》已暂行十几年，很多内容难以操作，收效甚微，建议制定反暴利法，规范暴利行为的认定标准，处罚方法，并通过立法把利润确定在一个合理的幅度以内。国家发改委认为，在市场经济条件下，暴利往往是欺诈、垄断、哄抬价格等违法行为的结果，建议做好立法之前的各项准备工作，加强调研，了解企业的实际生产经营情况，收集各行业和领域的有关统计数据，处理好发展市场经济和政府适度干预的关系，加强对重点行业的反暴利监管。财经委员会建议相关部门对议案提出的问题和建议认真进行研究，并通过完善政策法规，切实加强监管解决议案所提问题。

58．黄河等30名代表(第129号议案)提出，经济法是中国特色社会主义法律体系中的重要法律部门，目前我国已有六十部具体的经济法律，但没有一部统领、协调这些具体经济法律制度的基本经济法，建议制定经济法。财经委员会认为，从八届全国人大开始，我国按照建立社会主义市场经济体制的要求，为适应经济体制改革和经济社会发展的需要，加快各项经济立法，采用了分类单项立法的方式，实践证明，这种方式是行之有效的。制定经济法，实现经济法的法典化还需要更多的实践探索。

59．莫小莎等30名代表(第238号议案)提出，政府采购法第二十二条对中小企业参加政府采购进行了限制，使得国家对中小企业政策的落实大打折扣，建议修改政府采购法。财政部认为，政府采购法第二十二条第一款规定的是供应商参加政府采购活动应当具备的基本条件，立法本意并未对中小企业参加政府采购进行限制。对议案反映的问题，该部将加强法律的贯彻执行，有针对性地研究完善相关制度规范。财经委员会建议有关部门认真研究代表提出的意见，不断完善政策法规，进一步明确中小企业参加政府采购的政策，解决议案所提问题。

60．刘庆宁等31名代表(第241号议案)提出，由于历史、地理、自然等方面的原因，边境地区与内地相比，经济社会发展存在较大差距，边境地区贫困人口依然达总人口40％以上，发展面临的特殊困难很多，维护稳定的任务十分繁重，关心和支持陆地边境地区加快发展关系到民族团结、边疆稳定和边防巩固，关系到全面建设小康社会和构建社会主义和谐社会的进程，关系到国家的长治久安，建议制定陆地边境地区发展扶持促进法。国家发改委认为，议案所提意见非常中肯，对于促进边境地区发展的立法，建立健全相关法律体系，具有重要的指导意义。2011年国务院办公厅印发了《兴边富民行动规划 (2011-2015年)》，重点强调要加大对边境地区的支持力度，该委将在总结经验和专题调研的基础上，积极研究有关立法问题。财经委员会建议有关部门认真研究代表意见，落实国务院有关政策措施，解决议案所提问题。

61．齐奇等31名代表(第250号议案)提出，我国民事诉讼法和相关法律，对于民事执行程序和破产程序衔接的规定过于原则，缺乏刚性约束和可操作性的规范，影响了企业破产法的实施，建议修改企业破产法等法律，财经委员会认为，企业破产法实施以来，对规范企业破产行为，全面保护各方当事人利益，维护社会主义市场经济秩序，发挥了重要作用，同时在司法实践中也出现了一些新问题和新矛盾，需要完善相关法律制度，建议有关方面进一步研究论证代表意见，完善相关司法解释，解决议案所提问题。

62．朱雪琴等30名代表(第339号议案)建议制定公共服务基本法，界定公共服务的基本类型、公共服务的提供主体，规定公共服务评估制度等。国务院法制办认为，我国初步形成了由单行法律、法规和规章组成的公共服务制度框架，为满足公民基本公共服务需求提供了有效的法律保障。基本公共服务涉及许多方面，不同领域公共服务的对象、标准、模式差异很大，各地区之间保障水平很不平衡，制定统一的公共服务基本法有待研究论证。财经委员会建议有关部门认真研究代表意见，不断完善相关政策，改进工作，解决议案所提问题。

63．袁敬华等30名代表(第363号议案)提出，集体经济是我国宪法规定的公有制形式之一，建议制定集体经济组织法。财经委员会认为，随着我国社会主义市场经济体制的建立，国家对经济组织的划分标准也从原来的所有制、行业等属性转变为企业投资方式与责任形式，从而形成了公司、合伙企业、个人独资企业、农民专业合作社等经济组织形式并且已分别立法，大多数城镇和农村集体经济组织的设立与变更均已根据不同情况分别适用公司法、合伙企业法以及农民专业合作社法等。我国经济体制改革仍在进一步深化中，能否探索出新的集体经济组织形式，如何单独立法，都需要作进一步探索。

64．康凤英等30名代表(第365号议案)提出，就业促进法没有明确规定如何保障尚未达到职工法定退休年龄的中年劳动者的平等劳动权利，导致企事业单位用工时产生年龄歧视，建议修改就业促进法。人力资源和社会保障部认为，就业问题的解决有赖于劳动者个人、家庭、企业与政府多个主体承担起相应的责任，将会同有关部门对议案所提建议开展专门调研，深入研究人力资源市场上存在的年龄歧视等问题，及时提出政策建议。财经委员会同意人力资源和社会保障部意见。

65．郑杰等30名代表(第433号议案)提出，担保法等相关法律对中小企业信用担保机构规定不够全面，中小企业信用担保机构的法律地位、行业定位不明确，建议制定中小企业信用担保法。中国银监会认为，融资性担保业务监管部际联席会议认真研究了议案所提建议，并征求了有关部门意见，大多数部门对制定中小企业信用担保法的必要性持不同意见，仅对中小企业信用担保进行立法有待进一步研究。财经委员会同意中国银监会的意见，建议进一步研究完善中小企业信用担保机制，在推动相关工作中认真研究吸收议案所提建议。

66．徐景龙等31名代表、张涛等31名代表、杨国海等47名代表(第434号、第435号、第488号议案)提出，我国军用航空和民用航空分别立法、缺乏协调；对警务、海关、海洋监察以及其他非军事国家航空活动管理无法可依。为处理涉外航空纠纷，推动我国航空管理体制改革，规范国内所有航空事项，协调航空与其他相关领域的活动，迫切需要加快国家航空整体立法。中国民用航空局认为，在民航法制定前，曾着手制定航空法，后因各方意见不一致，才改为出台民航法。目前，国家空管委已经草拟了航空法，但由于有关部门对立法主体、空域管理体制等问题存在较大争议，立法工作尚在论证中。财经委员会建议有关部门和单位深入研究议案所提问题，积极探索完善我国航空管理制度。

67．左红等30名代表(第462号议案)提出，由于我国债券市场没有建立统一、明确的多层次法律体系，使得我国债券市场在统一准入条件、信息披露标准、资信评级要求、投资者保护要求等方面都未达成一致，多头监管使得各类债券在发行审核程序、发行标准、信息披露等方面宽严不一、监管效率低下，建议完善我国债券市场立法。财经委员会认为，议案提出的通过完善债券法律体系和债券交易机制、建立合格机构投资者制度、加强债券市场制度保障等建议，对推动我国债券市场发展具有积极的意义。鉴于目前债券市场和监管体制改革尚在进一步探索中，立法的时机还不够成熟，财经委员会将认真研究议案的有关建议，并根据今后金融市场的改革发展情况适时提出相关立法建议。

68．杨震等31名代表(第465号议案)提出，信息通信网络已成为社会的主要基础设施，同时物联网的法律规定基本空白，但在管理上存在政出多门、法律层级低等问题，建议制定互联网／物联网法或制定有关物联网应用的法规。工业和信息化部认为，制定全面规范互联网的基本法，时机尚不成熟；对物联网应用宜采取逐步立法的方式加以解决，建议统筹考虑互联网、物联网领域的实践情况和立法需求。财经委员会同意工业和信息化部的意见。

69．邹萍等34名代表(第475号议案)提出，目前对于服务于公共利益的公物还缺乏专门法律进行规范，致使公物质量差、过度商业化、公共用途受到损害以及公物使用人权利救济等问题得不到有效解决，建议制定公物管理法通则。财经委员会认为，国家、集体、私人都可以作为权利人，对特定的物享有直接支配和排他的权利，任何单位和个人不得侵犯。议案中提出的“公物”概念，即具有公共用途的公共财产，可以理解为国有和集体财产，范围包括矿藏、水流、海域、森林、山岭、草原、荒地、滩涂、野生动植物等自然资源，铁路、公路、电力设施、电信设施和油气管道等基础设施，以及国防资产和文物，国家机关和国家举办的事业单位的财产等等。国家和集体对于上述财产，如何行使占有、使用、收益和处分的权利，物权法都作了明确具体的规定。此外，由于公物的种类和范围在理论上还存在较大争议，在目前条件下，还难以对公物管理进行统一立法，一些问题有待作进一步探讨研究。

五、2件议案提出的1项执法检查和1项立法后评估的建议，建议全国人大常委会在研究制定今后工作计划时统筹安排

70．周晓峰等30名代表(第389号议案)提出，当前在车险理赔过程中无责不赔、高保低赔、拖延赔偿时间、代位求偿不力、定损标准不一等问题比较突出，建议全国人大常委会对保险法等有关法律的执行情况进行检查，并在此基础上启动车辆保险法的立法工作。中国保监会认为，该会高度重视车险理赔的问题，针对议案所提的问题将采取积极的措施加以改进。财经委员会建议有关部门严格执法，加强对车险理赔的监督检查。

71．刘沧龙等38名代表(第422号议案)建议开展信托法的立法后评估工作，以信托登记、公益信托、信托财产独立性三项制度为重点，加快推进信托法配套法规规章建设。中国银监会认为，该会2006年即组织研究信托登记制度问题，由于涉及诸多相关登记部门且无法达成一致，因此暂时搁置；该会也与民政部等相关部门多次沟通公益信托制度问题，共同研究起草公益信托管理办法，目前已完成初稿。财经委员会建议有关部门和单位加快信托法配套法规建设，统筹安排立法后评估工作。
