# Supplementary material for: Mapping the evolution of fertility support policies in China: A content and instrumental analysis
Source: PLoS One. 2025 Oct 9;20(10):e0332137. doi: 10.1371/journal.pone.0332137 (PMC12510515; doi:10.1371/journal.pone.0332137)
Supplement: S1 Appendix — (ZIP) [file pone.0332137.s001.zip › S1 Appendix. 226 original policy documents/107-全国人民代表大会教育科学文化卫生委员会关于第十二届全国人民代表大会第五次会议主席团交付审议的代表提出的议案审议结果的报告(FBM-CLI-1-307433).docx]

全国人民代表大会教育科学文化卫生委员会关于第十二届全国人民代表大会第五次会议主席团交付审议的代表提出的议案审议结果的报告

发布部门： 全国人大教育科学文化卫生委员会

发布日期：2017.12.24

实施日期：2017.12.24

时效性： 现行有效

效力级别： 工作文件

法规类别： 人大议事

全国人民代表大会教育科学文化卫生委员会关于第十二届全国人民代表大会第五次会议主席团交付审议的代表提出的议案审议结果的报告

全国人民代表大会常务委员会：

第十二届全国人民代表大会第五次会议主席团交付教育科学文化卫生委员会（以下简称我委）审议的代表提出的议案共66件，其中立法方面的议案61件，监督方面的议案5件。61件立法议案中，教育方面24件、科技方面2件、文化方面11件、人口卫生体育方面24件，共涉及37个立法项目；5件监督方面的议案涉及3个执法检查项目。

为做好本届最后一年的议案办理工作，我委按照全国人大常委会有关要求部署，不松劲、不懈怠，在以往工作基础上，不断探索和改进议案办理工作，提高议案办理质量和实效，精心做好今年的代表议案办理工作。一是召开主任委员办公会议研究部署相关工作，制定工作方案，确定专人负责。二是召开议案办理工作座谈会，有针对性地听取国务院13个有关部门同志的意见和建议。三是在议案办理过程中，继续坚持把代表议案办理工作与立法和监督工作相结合。四是不断加强与议案领衔代表的联系沟通。五是到代表驻地与代表面对面开展议案办理工作。

一、5件议案提出的3项监督项目，其中1项已由全国人大常委会组织实施；2项尚未组织实施。

1项已实施的监督项目

关于开展药品管理法执法检查的议案3件。

在2017年6月召开的十二届全国人大常委会第二十八次会议上，听取和审议了王晨副委员长兼秘书长所作的关于检查药品管理法实施情况的报告。

2项尚未实施的监督项目

（1）关于开展促进科技成果转化法执法检查的议案1件。

议案就该法中有关科技成果评估方法及国有资产管理等内容提出异议，希望开展执法检查。我委在2017年开展了有针对性的专题调研3次。建议国务院有关部委要进一步加大对该法贯彻落实，切实解决问题。

（2）关于提请全国人大常委会开展教育法执法检查的议案1件。

代表在议案中提出的问题非常重要，我委建议，全国人大常委会在对教育法、义务教育法、教师法、未成年人保护法实施情况进行监督时认真研究以推动问题的解决。

二、2件议案提出的1个立法项目已由全国人大常委会审议通过

关于制定公共图书馆法的议案2件。

第十二届全国人大常委会第三十次会议通过了《中华人民共和国公共图书馆法》。

三、19 件议案提出的6个立法项目，已列入十二届全国人大常委会立法规划或年度立法工作计划，适时提请全国人大常委会审议

1．关于尽快修改职业教育法的议案2件；

2．关于制定学前教育法的议案7件；

3．关于修改专利法第六条的议案1件；

4．关于修改文物保护法的议案3件；

5．关于制定基本医疗卫生法（国民健康法）的议案4件；

6．关于修订药品管理法的议案2件。

四、16件议案提出的9个立法项目确有立法必要，建议有关部门认真研究论证，加快调研起草工作，条件成熟时提请全国人大常委会审议

1．关于制定家庭教育法的议案3件；

2．关于制定终身教育法的议案1件；

3．关于制定继续教育法的议案1件；

4．关于修改教师法的议案2件；

5．关于加快修订学位条例的议案1件；

6．修改档案法的议案4件；

7．关于制定执业药师法（药师法）的议案2件；

8．关于修改执业医师法的议案1件；

9．关于修订体育法的议案1件。

五、24件议案提出的21个立法项目，有的在相关法律法规中已有规定，有的可通过加强执法监督、制定相关政策或规章解决议案所提问题，还有的待立法条件成熟后再纳入立法工作计划

（一）4件议案提出的4个立法项目已有相关法律法规，可通过加大执法监督力度、或制定政策解决议案所提问题

1．关于修订科普法的议案1件。

我委建议，有关部门和组织应结合代表议案内容，进一步加大对现行科普法的贯彻实施力度。

2．关于制定古都保护法的议案、关于尽快制定国家历史文化名城名镇保护法的议案共2件。

我委将继续督促有关部门进一步加强执法，并在修改文物保护法和相关监督工作中认真研究吸纳代表们的意见和建议，积极推进历史文化名城等文化遗产和古都的保护工作。

3．关于制定心理健康促进法的议案1件。

我委建议，国务院有关部门要进一步贯彻落实精神卫生法的有关规定，加强沟通协调，完善政策。

（二）9件议案提出的8个立法项目，相关部门正在研究制定修改相关法律法规

1．关于制定考试法的议案1件。

我委认为，代表们在议案中所提建议很有参考性，希望教育部在制定国家教育考试条例时认真研究采纳。

2．关于制定高等院校招生与考试法的议案1件。

我委认为，代表议案关注高考公平问题，提出的建议非常重要，我们将在相关立法过程中予以借鉴吸收。

3．关于制定烟害防治法的议案、关于制定公共场所禁烟法的议案共2件。

我委建议，国务院相关部门要深入论证议案所提出的重点难点问题，协调各方意见，进一步凝聚共识，加快立法进程。我委将继续督促推动全面控烟工作取得新进展。

4．关于制定互联网药品交易法的议案1件。

我委建议，国务院相关部门要认真研究吸纳代表议案中提出的意见和建议，加强立法研究，争取尽早将药品管理法修改草案提请全国人大常委会审议，并在工作中完善相关制度，切实保障人民群众用药安全。

5．关于加快儿童用药立法的议案1件。

我委建议，国务院相关部门应加快立法进程，积极研究吸纳代表所提意见和建议，不断完善儿童用药保障制度。

6．关于制定急救医疗服务法的议案1件。

我委建议，国务院相关方面应认真研究代表议案提出的问题和建议，抓紧调研，加快法规起草进程，同时继续抓好政策完善和落实工作，为加快推进我国医疗急救事业全面、协调、可持续发展提供法律保障。

7．关于修改母婴保健法的议案2件。

我委建议，国务院相关部门要认真研究代表议案提出的问题，认真贯彻妇幼卫生工作方针，加快妇幼卫生健康服务体系建设，针对法律实施和管理工作中遇到的问题，统筹修订完善相关法规规章，理顺体制，规范服务，为母婴保健法以后的修订工作积累经验。

（三）11件议案提出的9个立法项目，建议有关部门认真调研论证

1．关于制定互联网教育法的议案1件。

我委认为，互联网教育的实施主体、教育目标、服务对象复杂多样，其规范和管理的专业性、技术性较强。与其他教育法律比较起来，互联网教育法涉及面更广、涉及主体种类更多、法律关系更加复杂，对于各部门间的协同配合、资源统筹等机制要求更为迫切，超出了现有教育管理体制的管理范围。我委将与有关部委就互联网教育发展应用中的许多问题进一步加强探索研究。

2．关于修改义务教育法的议案3件和关于修改教育法个别条款的议案1件。

这四件议案所提出的在现阶段将学前教育或高中教育纳入义务教育。我委认为这属于国家基础教育的基本制度设计。建议国务院有关方面加快研究，提出可行性操作方案。

3．关于制定抗菌药管理法的议案1件。

我委建议，国务院相关部门应认真研究吸纳代表议案提出的问题和一些具体管理建议，加大相关规章规定的落实，加强管理和督查，适时研究制定相关管理法规。

4．关于肿瘤防治法立法的议案1件。

我委建议，国务院相关部门要认真贯彻落实慢性病防治的相关规划，深入推进癌症防治体系建设，进一步完善相关配套规章，适时开展肿瘤防治立法论证工作。我委在推进基本医疗卫生与健康促进法等相关法律立法过程中，认真研究了代表议案提出的具体建议，充分考虑癌症等重大疾病防控工作，以适当方式予以规定。

5．关于建立反医疗暴力法的议案1件。

我委建议，国务院相关部门应认真研究吸纳代表议案提出的意见和建议，及时制定完善有关法规制度，建立完善医疗纠纷预防和处理的长效机制，进一步加强医疗机构的医德医风建设，不断提高医疗服务质量，坚决打击涉医违法犯罪活动，积极维护正常的医疗秩序，持续加强宣传教育力度，努力营造尊医重卫的社会氛围。

6．关于制定遗体捐献法的议案1件。

我委建议，国务院相关部门要认真研究代表议案提出的意见和建议，逐步完善相关的配套规章，积累有关遗体捐献的管理经验，探索建立适合我国国情的遗体捐献管理制度，适时研究制定遗体捐献法律法规。

7．关于修订完善食品安全法的议案1件。

我委建议，国务院及相关部门要认真研究代表议案反映的问题和意见，完善配套法规规章，待时机成熟时再修改食品安全法。

8．关于修改人口与计划生育法个别章节的议案1件。

我委建议，国务院相关部门应认真研究代表议案提出的问题，加强与相关方面的沟通协调，坚持人口与发展综合决策，坚持计划生育基本国策，进一步完善计划生育政策和社会保障、税收、住房、教育等相关政策，促进人口长期均衡发展。同时，根据人口形势发展情况，修改完善人口与计划生育法。

上述66件议案的具体审议意见，详见附件。

以上报告，请审议。

附件：1．全国人民代表大会教育科学文化卫生委员会关于第十二届全国人民代表大会第五次会议主席团交付审议的代表提出的议案的审议意见

2．十二届五次会议议案审议结果报告有关数据统计

全国人民代表大会教育科学文化卫生委员会

2017年12月24日

附件1

全国人民代表大会教育科学文化卫生委员会关于第十二届全国人民代表大会第五次会议主席团交付审议的代表提出的议案的审议意见

一、5件议案提出的3项监督项目，其中1项已由全国人大常委会组织实施；2项尚未组织实施。

1项已实施的监督项目

高广生、姜健、袁敬华等91名代表提出开展《药品管理法》执法检查的议案3件（第45号、277号、288号）。议案建议全国人大常委会对药品管理法实施情况开展执法检查。按照张德江委员长批示精神，2017年3月至5月，全国人大常委会对药品管理法实施情况进行了执法检查。王晨副委员长兼秘书长在第十二届全国人大常委会第二十八次会议上作了关于检查药品管理法实施情况的报告。

2项尚未实施的监督项目

1．朱志远等31名代表提出关于开展促进科技成果转化法执法检查的议案1件（第82号）。议案建议全国人大常委会对促进科技成果转化法实施情况开展执法检查，重点关注科技成果评估定价环节与促进科技成果转化法有关规定衔接问题。2016年7月至8月，全国人大常委会对促进科技成果转化法实施情况开展了执法检查。王晨副委员长兼秘书长在十二届全国人大常委会第二十四次会议作了关于检查促进科技成果转化法实施情况的报告。会后，审议意见连同执法检查报告一并送国务院研究处理。按照监督法的有关规定，2017年4月召开的全国人大常委会第二十七次会议，书面审议了国务院提出的研究处理报告。

为进一步做好后续工作，增强执法检查实效，按照王晨副委员长兼秘书长指示要求，我委继续开展执法检查后续跟踪调研工作。先后赴湖北省、北京市、黑龙江省对促进科技成果转化法贯彻实施情况进行跟踪调研。教科文卫委员会在执法检查后续跟踪调研中，重点关注了法律实施过程中科技成果评估定价环节与促进科技成果转化法有关规定衔接问题，并在调研报告中提出了具体意见，建议有关部门加强研究，健全符合科技创新规律的无形资产管理制度。

2．花蓓等31位代表提出关于开展教育法执法检查的议案1件（第81号）。议案指出，近年来，性侵害未成年人犯罪案件频频发生，引起家长恐慌和社会各界的关注，损害了教师职业和教育行业的形象和声誉。在办案过程中发现，发案学校在聘用教师及其他工作人员时存在把关不严、管理不善的问题，在司法机关和教育行政部门之间也存在着刑事司法和行政执法的信息通报和机制衔接不畅的问题。议案建议全国人大常委会针对这些问题对教育法的施行情况开展执法检查。代表在议案中提出的问题非常重要，我委建议，全国人大常委会适时对教育法、义务教育法、教师法、未成年人保护法实施情况进行监督时加大研究，以推动问题解决。

二、2件议案提出的1个立法项目已由全国人大常委会审议通过

姜健等30名代表提出关于尽快制定公共图书馆法的议案1件（第270号），蒋婉求等34名代表提出关于制定图书馆法的议案1件（第176号）。议案提出，用法律来保障和促进公共图书馆事业健康有序发展，具有必要性和紧迫性；制定公共图书馆法，要明确经费保障机制，科学规划和建设公共图书馆设施，重视对少儿图书馆的设置；在对公共图书馆立法的同时，要重视对学校图书馆、专业图书馆等其他类型图书馆的法律保障。

制定公共图书馆法是十二届全国人大常委会立法规划中的第一类项目，我委一直高度重视该法的立法工作，近年来赴多地进行立法调研，并与有关部门就重点问题多次进行研讨。2017年6月1日接到全国人大常委会办公厅转来的《国务院关于提请审议〈中华人民共和国公共图书馆法（草案）〉的议案》，同年6月5日召开全委会对《中华人民共和国公共图书馆法（草案）》（以下简称草案）进行了审议。6月23日，第十二届全国人大常委会第二十八次会议对草案进行了初审，之后根据常委会组成人员的意见进行了反复修改，认真研究并吸纳了议案中的内容。第十二届全国人大常委会第三十次会议通过了《中华人民共和国公共图书馆法》。

三、19件议案提出的6个立法项目，已列入十二届全国人大常委会立法规划或年度立法工作计划，适时提请全国人大常委会审议

1．赵国红、姜健等61名代表提出关于尽快修改职业教育法的议案2件（第125、272号）。议案指出，1996年实施的职业教育法，是我国职业教育发展史上的重要里程碑，为推动职业教育改革与发展起了巨大作用。但随着我国经济发展进入新常态、职业教育已经不能完全适应当前改革发展的要求，存在结构不尽合理、质量有待提高、办学条件不足、体制机制不畅等突出问题。需要通过修订职业教育法，从根本上为加快发展现代职业教育，实现职业教育现代化提供法律保障。

全国人大及其教科文卫委员会高度重视职业教育法修订工作。2013年，职业教育法（修改）列入十二届全国人大常委会立法规划。2014年，教科文卫委员会赴黑龙江、贵州、吉林等省开展修订职业教育法立法调研，积极推动法律修订进程。2015年，全国人大常委会对职业教育法实施情况开展了全覆盖的执法检查。2016年、2017年，教科文卫委员会分别赴重庆、新疆、吉林就执法检查整改落实情况进行后续跟踪调研，以进一步推动法律贯彻实施。国务院有关部门高度重视职业教育法的修订工作，2016年，教育部以党中央、国务院领导指示精神，国务院有关决定以及全国人大执法检查报告为依据，在职业教育改革发展实践基础上，集中修订和补充重要条文，形成职业教育法修订草案，并征求了国务院各部门及地方的意见。目前，教育部已经形成了修正案（草案），并与人力资源和社会保障部就草案内容进行积极协商。下一步，教科文卫委员会将继续加强与有关部门的沟通联系，推动职业教育法修订草案尽快提请全国人大常委会审议。

2．郝萍、白红战、姜健、张淑琴、陈爱莲、庞丽娟、王家娟等219名代表提出关于尽快制定学前教育法的议案7件（第61、142、279、282、343、349、443号）。议案肯定了我国学前教育事业改革发展取得的重大进展，各地在探索实践中也积累了许多成功经验。但学前教育仍是我国教育体系中最为薄弱的环节，存在着发展不均衡、资源总体短缺、合格师资严重缺乏、管理体制不健全等突出问题。学前教育作为国民教育和社会公共服务体系的重要组成部分，社会各界对其立法的重要性、迫切性已达成共识。

全国人大及其教科文卫委员会高度重视学前教育立法工作。2013年以来，教科文卫委员会先后赴天津、安徽、云南、吉林、四川开展一系列调查研究，总结梳理各地学前教育发展和立法状况，分析研判学前教育立法面临的主要困难和问题。2015年12月，全国人大常委会审议通过教育法修正案，增加关于学前教育的专门规定。2016年，把全国人大代表提出的24份有关大力发展学前教育的建议列为重点督办建议。教育部也做了大量工作，先后修订幼儿园工作规程，出台关于幼儿园建设、编制、卫生保健等方面的具体政策，并已初步形成学前教育法专家建议稿。教科文卫委员会将督促起草部门认真研究采纳代表建议提出的意见，及时沟通了解工作进展和重点难点问题，加快推动学前教育立法进程。

3．徐建群等62名代表提出关于修改专利法的议案1件（第190号）。议案建议，全国人大常委会通过改革试点授权在全面创新改革八大区域内暂停适用专利法第六条，同时尽快修改专利法第六条，规定单位与发明人可以对职务发明创造专利确权、申请、收益等相关事项进行约定。十二届全国人大常委会将修改专利法列入常委会五年立法规划中第一类项目。据了解，国务院法制办会同国家知识产权局已经初步形成了《中华人民共和国专利法修正案（草案）》，目前修正案草案征求意见工作也已完成。下一步，教科文卫委员会将对议案提出的单位与发明人可以约定职务发明创造专利权归属问题加强研究，继续督促有关部门抓紧做好法律修正案草案有关工作，积极推动这部法律早日提交全国人大常委会审议。

4．金钢、卢云辉、周岚等等100名代表提出关于修改文物保护法的议案3件（第189、356、496号）。议案提出，尽快推动文物保护法的修法进程，采用概括性方式对文物的概念进行科学界定，使定义更为明晰；提高古籍保护要求，加强古籍整理和利用；加强对文物建筑的保护，对其修缮、技艺传承等作出具体规定。

修改文物保护法是第十二届全国人大常委会立法规划中的第二类项目。2013年，文化部根据第十一届全国人大常委会文物保护法执法检查报告的要求，启动了文物保护法修改工作，并于2015年向国务院报送了送审稿，国务院法制办于2015年12月向社会公开征求意见。目前，有关部门正积极开展立法调研。有关单位正在对文物的概念等问题开展专项课题研究。文化部已委托国家古籍保护中心组织专家研究起草了古籍保护条例（初稿），正在积极推进条例的出台。有关部门近年来十分重视文物建筑保护工作，形成了多项标准，推动保护工作规范化、制度化。我委十分重视文化保护法修改工作，同文化部等单位多次就有关问题进行研究探讨。我委将密切关注并积极推动这项立法工作，并督促有关部门在修法过程中认真研究吸纳代表们的意见和建议。

5．张伯礼、唐祖宣等62名代表提出制定《国民健康法》的议案2件（第52、154号），姜健、陈海啸等62名代表提出尽快制定基本医疗卫生法的议案2件（第275、347号）。制定基本医疗卫生法是全国人大常委会立法规划项目，从2014年底起，由我委牵头负责起草工作。法律草案起草工作已基本完成，在2017年12月召开的全国人大常委会进行初步审议。我委认为，制定基本医疗卫生法的主要目标和任务：一是建立基本医疗卫生制度，明确政府、社会、个人在医疗卫生和健康促进方面的权利和义务，提高国民健康水平；二是以法治手段推动和保障全国卫生与健康大会精神的落实，将人民健康放在优先发展的战略地位；三是将医改的成熟经验用法律的形式予以固化；四是以问题导向，解决深化医改中面临的体制性机制性问题。法律名称在法律草案起草过程争议较大，基本医疗卫生法与国民健康法、卫生基本法名称不同，但立法目的都是保障居民健康权益。经多方协调，我委拟将法律名称定为基本医疗卫生与健康促进法。从内容上看，主要是以建立基本医疗卫生制度为核心，适当扩充健康促进内容。代表提出的意见和建议，我们已在法律草案修改完善过程中进行了认真研究和充分吸纳。

6．谢子龙、李康等60名代表提出加快推进药品管理法修订的议案2件（第417号、493号）。议案提出，《药品管理法》中“药品”的概念有待进一步明确，对假劣药品的定义欠合理；对药品使用环节、包括互联网药品销售的监管需要明确和加强；执业药师配备及职责的要求缺失、部分条款的可操作性不强等，需要修订完善等；建议做好《药品管理法》和《中医药法》衔接工作，并能够增加鼓励包括少数民族药品发展的相关条款等。我委希望国务院及相关部门加快法律修改步伐，在起草修订草案过程中，认真研究和充分吸纳代表议案提出的意见和建议，抓紧修改和完善修订草案，争取早日提请全国人大常委会审议。

四、16件议案提出的9个立法项目确有立法必要，建议有关部门认真研究论证，加快调研起草工作，条件成熟时提请全国人大常委会审议

1．陈秀榕、胡季强、郭新志等92位代表提出关于制定家庭教育法的议案3件（第10、101、352号）。议案指出，家庭是社会的基本细胞，注重家庭、注重家教、注重家风，对于国家发展、民族进步、社会和谐具有十分重要的意义。面对社会转型、家庭变迁、教育变革的外部环境，我国目前家庭教育面临诸多不容忽视的问题和新的挑战。建议通过立法，明确家庭教育的法律地位与性质、应遵循的基本原则、界定家庭教育有关主体的职责范围、规范家庭教育指导行为。议案提出了立法框架或法律建议稿。

教育改革和发展规划纲要提出，要充分发挥家庭教育在儿童少年成长过程中的重要作用，制定有关家庭教育的法律。目前，由全国妇联牵头，教育部配合，相关部门参与的家庭教育立法工作机制已经建立，并开展了调研工作。我委重视家庭教育立法的代表议案办理工作，今年分别到山西、浙江调研，与议案领衔代表郭新志、胡季强面对面沟通。通过调研和与代表交换意见，我委认为，议案领衔代表及有关部门、专家学者和家长都对家庭教育的重要性达成了共识，但对拟制定的法律的内容、重点和实施途径等仍有不同意见。我委建议，立法需考虑家庭教育自主性和多样化，以及如何实施有效的促进与保障。我们将对代表们提出的立法可行性、必要性及立法建议认真研究，积极参与前期的立法调研和论证工作，适时推动家庭教育立法进程。

2．张淑琴等30位代表提出关于制定终身教育法的议案1件（第266号）。议案指出，终身教育的推行与终身教育体系的构建应与国家的发展目标、政策法规相适应，建议国家从战略发展的高度来规范、约束和指导终身教育的开展，制定终身教育法。议案从立法宗旨、立法目的、投入保障、领导体制和运行机制、政府职责、中央与地方的任务、监督机制等方面提出十二条立法建议。

教育改革和发展规划纲要明确提出了终身学习（终身教育）的立法任务。近年来，教育部重视终身教育立法研究工作，已经开展了国内外有关资料的收集整理工作，为立法提供参考借鉴。2013年，教育部报送全国人大的五年立法规划建议中，将终身学习法列入其中。2015年12月，全国人大常委会在修改教育法时增加了关于终身教育的相关内容。同时，福建、河北、云南、上海、太原、成都、宁波等省市已出台终身教育促进条例，在地方立法方面进行先行探索，取得了显著成果。我委认为，发展终身教育，对促进人的全面发展和社会进步都具有重要意义。建议教育部继续加强前期研究，进一步开展终身教育立法相关工作。

3．董雅娟等33位代表提出关于继续教育立法的议案1件（第268号）。议案指出，改革开放以来，我国继续教育事业发展很快，已成为我国各行业领域技术、管理等人员提高能力素质的重要途径和手段。目前虽然没有关于继续教育的专门法律，但教育法等法律都有涉及继续教育的条款，大多数省市区已制定了继续教育地方性法规或地方政府规章。为了保证继续教育的战略地位，充分发挥继续教育对各行业领域人员能力素质提升的重要作用，推动继续教育健康持续发展，建议尽快组织推动继续教育立法工作。议案提出了立法框架方案。

发展继续教育，对促进人的全面发展和社会进步都具有重要意义。2015年12月，全国人大常委会在修改教育法时增加了关于继续教育的相关内容。教育部及国务院有关部门、地方都在制定推进社区教育、老年教育、学习型城市建设、职工继续教育等领域发展的文件，全国157个地级市发布了有关推进社区教育、加快学习型社会建设的文件，占到全国333个地级市的近一半。

我委认为，继续教育是面向学校教育之后所有社会成员的教育活动，特别是成人教育活动，是终身学习体系的重要组成部分。近年来，教育部重视终身教育立法研究工作，已经开展了国内外有关资料的收集整理工作，为立法提供参考借鉴。鉴于继续教育和终身学习两者之间具有十分密切的联系，而教育改革和发展规划纲要已明确提出了终身学习（终身教育）的立法任务。因此，建议对继续教育不再单独立法，放入终身学习立法中综合考虑。议案对继续教育法总体框架提出的方案，对相关立法有帮助，建议国务院有关部委认真研究、参考借鉴。

4．花蓓、王家娟等62位代表提出关于修改教师法的议案2件（第75、446号）。议案指出，教师法已经实施了24年，在保护教师合法权益、保障教师待遇、加强教师队伍管理等方面发挥了重要作用。但目前教师法的一些规定已不适应时代发展的需要，建议尽快修改教师法，解决少数教师师德水平下滑，教师的专业化水平不均衡，福利待遇有差距，职业幸福感低等问题。议案建议对教师法从以下几个方面进行修改：提高教师待遇，保障教师权益，加强师德建设，修改教师法有关教育行业从业资格规定，设立一线教师、班主任荣誉勋章。

教育改革和发展规划纲要提出，要进一步完善教育法律法规，加快教育法制建设进程，修订教师法等有关法律。对代表们所提出的修改意见，我委进行了认真研究，认为具有很强的针对性和指导性。五年来，在义务教育法、职业教育法执法检查和相关调研中，教师队伍建设问题也一直作为工作重点。下一步，我们将继续关注教师法的修订工作，修法工作中认真研究采纳代表们的意见建议。

5．邓秀新等41位代表提出关于修订学位条例的议案1件（第143号）。议案指出，学位条例实施三十多年来，为培养社会主义现代化建设急需的高层次人才提供了坚强的保障，为高等教育事业发展作出了举足轻重、不可替代的重要贡献。当前，我国经济社会发生了翻天覆地的深刻变化，高等教育事业也经历了举世瞩目的重大变革，学位条例已不能完全满足改革实践与现实需要，一些做法已经突破了学位条例规定，修法任务非常紧迫。议案阐述了修法的必要性，并提出学位法（修订参考稿）。

近年来，教育部一直高度重视学位条例修订工作。2012年5月，委托相关单位开展修订课题研究工作。经广泛调研、咨询和论证，于2015年形成了“修订专家建议稿”等工作成果。2016年，国务院学位委员会办公室专门成立修订工作组，围绕学位授权和学位授予等重要问题，开展调研，全面梳理重大问题与政策建议，形成了学位法（参考稿）。该参考稿已经于2017年1月，经国务院学位委员会审议并原则通过。目前，教育部正在对学位条例修订的有关重大问题做进一步调研，力争在2017年内完成部内起草程序，适时提请国务院审议。我委认为，修订学位条例十分必要，议案对加快修订学位条例的分析和提出的修法建议稿有很强的参考意义，我们在修法中将认真研究、参考借鉴。

6．海南代表团、郑玉红、杨伟程、李桂杰等116名代表提出关于修订《中华人民共和国档案法》的议案4件（第11、184、298、445号）。议案提出，档案法制定迄今已30年，其中不少规定与档案工作和经济社会发展的现实需要之间不相适应的问题较为突出，建议尽快进行修订，理顺档案工作体制，加强档案开放与利用，加大对档案的收集、归档和保护力度，增加关于电子档案的规定等。

2007年，国家档案局正式启动了档案法修改工作。10年来，国家档案局多次召开座谈会、研讨会，并在系统内部以及中央国家机关、高校、研究院所等单位广泛征求意见。2015年12月，国家档案局向国务院提交了档案法修订草案送审稿，国务院法制办向有关部门、企事业单位征求了意见，目前正对征求到的意见和建议进行梳理，对有关重点难点问题进行研究论证。我委十分重视该法的修改工作以及相关议案的办理工作，2017年7月，我委组成调研组赴浙江就相关议案办理工作开展专题调研，与部分代表进行了座谈，面对面地听取代表们的意见。我委工作机构多次参加国家档案局组织的执法调研和座谈会。我委将积极推进该法的修改工作，并督促有关部门在工作中认真研究吸纳代表们的意见和建议。

7．谢子龙、宋礼华等60名代表提出制定执业药师法（或药师法）的议案2件（第410、488号）。议案提出，药品安全问题是重大的民生问题和公共安全问题，事关人民群众的生命健康和社会稳定。我国执业药师法律保障制度不够健全，患者不合理用药现象普遍存在。尽快出台《执业药师法》，实现与国际药师管理制度的接轨，有利于理顺我国执业药师管理体制、扩大执业药师队伍规模、提高执业药师整体素质、增强我国药事服务供给能力。我委曾于2016年8月专门就执业药师工作和相关立法问题赴部分地区进行了调研。2013年，国务院已将药师法列入了国务院立法计划的第三档。建议国务院及相关部门认真研究代表议案提出的问题和建议，推进执业药师法（或药师法）的立法进程，尽早提请全国人大常委会审议。

8．张加春等33名代表提出修改执业医师法的议案1件（第133号）。议案提出，法律条文中试用期一年等规定，不利于医师人才队伍建设，也不符合医疗人才成长的规律和内在需要，需要进行修订。执业医师法自1999年5月1日实施至今17年，不少条款已经不能完全适应当今社会的实际需要，包括在医师考试、医师资格、医师执业、行业管理等方面存在许多不足，应当修改完善。我委将进一步加强调研。希望国务院及相关部门认真研究代表议案提出的意见和建议，推进执业医师法修改的立法工作进程，尽早提请全国人大常委会审议。

9．杨伟程等31位代表提出修订体育法的议案1件（第263号）。议案提出，体育法施行20多年来，对推动体育事业进步，增强全民族身体素质，提高体育运动水平，促进社会主义物质文明和精神文明，加强和创新社会管理等发挥了重要作用。随着我国经济社会发展，体育体制改革步伐加快，体育法已不能完全适应体育市场化、产业化、职业化、社会化和现代化发展需要，应该适时对体育法进行修订。2015年，我委与国家体育总局联合召开了体育法颁布实施20周年座谈会，陈竺副委员长对做好修改体育法工作提出了要求。目前，修改体育法列入了《体育发展“十三五”规划》。国家体育总局一直在抓紧修订体育法的论证和调研工作，2017年成立了修改体育法工作机构，召开了座谈会，就修订体育法中遇到的重点问题进行研究论证。我委将积极关注体育法修改进展情况，加强协调沟通，推动法律修改进程。

五、24件议案提出的21个立法项目，有的在相关法律法规中已有规定，有的可通过加强执法监督、制定相关政策或规章解决议案所提问题，还有的待立法条件成熟后再纳入立法工作计划

（一）4件议案提出的4个立法项目已有相关法律法规，可通过加大执法监督力度、或制定政策解决议案所提问题

1．霍金花等43名代表提出的关于修改科学技术普及法的议案1件（第406号）。议案指出，当前科普工作内容极大丰富，手段日新月异，任务非常紧迫。科普法于实施至今已15年，难以应对科普工作的新形势、新问题、新任务，建议修改。明确科普工作的对象是全体公民，重点是未成年人、农民、城镇劳动者、领导干部和公务员、社区居民；适应科普信息化发展的新形势新要求，加强优质科普内容资源建设、科普阵地条件建设，创新科普公共服务机制，突出科普惠民服务。

我委将开展科学技术普及法实施情况调研纳入2017年工作安排，对科学技术普及法实施情况以及科普工作目前存在的困难和问题开展了深入调研，认真了解科普法实施的情况。建议有关部门和组织结合代表议案内容，进一步加大对现行科普法的贯彻实施力度。

2．吉炳伟等30名代表提出关于制定古都保护法的议案1件（第66号），杜国玲等32名代表提出关于尽快制定国家历史文化名城保护法的议案1件（第181号）。议案提出，当前我国古都保护面临巨大挑战，应整合现有文物保护、历史文化名城保护相关的法律法规规定，借鉴外国成功经验，进行古都保护立法；议案还提出历史文化名城保护中存在规划滞后、体制不顺、监督不到位等问题，亟需进行国家立法。

我委高度重视文化遗产的保护工作，历史文化名城（含古都）、名镇，是我国文化遗产的重要组成部分。目前，我国已经制定了文物保护法、城乡规划法和历史文化名城名镇名村保护条例等法律法规，一些地方根据实际出台了相关地方性法规和政府规章。古都保护作为历史文化名城保护的重要组成部分，已经纳入了相关法律法规的调整范围。关于历史文化名城保护工作中出现的规划滞后等一些具体问题，需要通过严格执法、加强监督等措施加以解决。我委将继续督促有关部门进一步加强执法，并在修改文物保护法和相关监督工作中认真研究吸纳代表们的意见和建议，积极推进历史文化名城等文化遗产的保护工作。

3．沈健等31名代表提出制定心理健康促进法的议案1件（第502号）。精神卫生既是全球性的重大公共卫生问题，也是较为严重的社会问题，为此，全国人大常委会于2012年颁布了精神卫生法。关于心理健康促进和精神障碍预防工作，精神卫生法在第二章作出规定，对政府、学校、医院、用人单位、基层组织等各有关方面在加强心理健康促进和精神障碍预防工作的职责加以明确，努力提高公众心理健康水平。在第二十三条也对心理咨询人员开展服务提出了要求。建议国务院有关部门进一步贯彻落实精神卫生法的有关规定，加强沟通协调，完善政策。

（二）9件议案提出的8个立法项目，相关部门正在研究制定修改相关法律法规

1．沈健等33位代表提出关于制定考试法的议案1件（第177号）。议案指出，我国各类国家考试涵盖了教育、司法、人事、卫生等多个领域，规模庞大。目前在全国范围内统一组织实施的考试达200多种，每年参考人数超过3000万人。考试已经成为衡量和选拔人才、保障社会公平正义的重要手段。然而，考试立法至今仍是空白，一些考试的设定依据来源于部门规章和规范性文件，法律位阶较低，有的考试还缺乏法律法规依据。虽然2015年11月1日起正式施行的《刑法修正案（九）》对考试作弊作出了明确规定，但从法治原则以及考试具体实施来看，无论是考试的设置还是组织实施过程都还存在行政程序简单、救济渠道不健全等诸多问题。建议制定国家考试法，保障国家考试的公平、科学与安全，维护公民合法权益。

我国是考试大国，考试种类多、参加人数多、社会影响大、利益群体广泛。据了解，教育部2009年曾起草过《考试法（草案）》并提交国务院审议。但由于各类国家考试分别由不同的部门负责组织实施，并分别以相应的法律、法规或者部门规章为设定依据，有关部门认为制定统一的《考试法》，难以适用不同考试的特殊情况，难以调整各种不同类型的考试，考试法立法工作没有继续推进。目前，教育部正在研究起草《国家教育考试条例》。我委认为，代表们在议案中所提建议很有参考性，希望教育部在制定国家教育考试条例时认真研究采纳。

2．买世蕊等30位代表提出关于制定高等院校招生与考试法的议案1件（第247号）。议案提出，为实现教育资源和受教育机会在地区之间的公平分配，全国性重点高等院校的招生名额，应按照各地参加高考的考生人数大致相同的比例，分配给各地，并增加对边远地区考生的招生名额。同时，应保障全国性重点高校的自主招生权利。建议制定高等院校招生与考试法，确立并保障各地考生的平等权利。

高考招生制度的功能是为高等学校提供选拔人才的依据，同时也肩负着许多社会功能和使命，关乎社会稳定和公平。2014年9月国务院印发《关于深化考试招生制度改革的实施意见》，启动了恢复高考以来最全面、最系统的一次考试招生制度改革。此后，教育部陆续出台了关于改进招生计划管理、实施高中学业水平考试和综合素质评价、规范高考加分、完善自主招生等20余个文件，基本形成了整体推进改革的政策体系。通过改革，中西部和人口大省高考录取率稳步提升；农村和贫困地区学生上重点高校人数大幅增加；高职院校分类考试模式更趋完善；高考命题质量和公信力得到提升；自主招生更加规范有序。据了解，教育部正在开展国家教育考试条例起草工作。目前，尚无制定专门的高等学校招生与考试法的立法计划。我委认为，代表议案关注高考公平问题，提出的建议非常重要，我们将在相关立法过程中予以借鉴吸收。

3．马文芳等30名代表提出制定烟害防治法的议案1件（第62号），张淑琴等30名代表提出的制定公共场所禁烟法的议案1件（第287号）。议案提出，香烟盒印制警示标识、禁止烟草广告和音像制品出现吸烟镜头、禁止公共场所吸烟、禁止使用有特殊意义的名称作为烟草商标、提高烟草税收、改革烟草行业管理体制等。国务院法制办于2016年将公共场所禁烟条例列入一类立法计划，目前正在审查修改国家卫计委报送的《公共场所控制吸烟条例（送审稿）》。据了解，该条例中许多禁止性条款的内容与议案的内容相同或相近。我委建议国务院有关部门继续深入论证重点难点问题，努力协调各方意见，进一步凝聚共识，加快立法进程。我委将继续督促推动全面控烟工作取得新进展。

4．谢子龙等30名代表提出制定互联网药品交易法的议案1件（第418号）。议案提出，应该明确互联网药品监管部门及其职责权限，经营企业的责任义务，消费者权益，药品交易的范围、服务的技术标注和交易规范，配送企业的资质和条件等。全国人大常委会于2017年上半年开展了药品管理法执法检查，并听取和审议了国务院关于药品管理工作的专题报告。在执法检查报告中提出加快药品管理法修订，“深入推进‘互联网＋药品’安全建设”等。修改药品管理法已经列入全国人大常委会立法规划，国家食品药品监管总局正在研究起草修订草案，并将对网络销售药品等做出相应规定。同时，国家食药总局正在研究起草网络药品经营监督管理办法，拟在规章层面明确并细化网络药品交易的管理要求。建议国务院相关部门认真研究吸纳代表议案中提出的意见和建议，加强立法研究，争取尽早将药品管理法修改草案提请全国人大常委会审议，并在工作中继续完善相关制度，切实保障人民群众用药安全。

5．李甦雁等31名代表提出关于加快儿童用药立法、保障儿童健康的议案1件（第501号）。议案提出，要根据儿童身心发展特点和儿童医疗卫生事业发展和需求，立法规范儿童专用药物的适宜的采购方式、临床使用规则，保障儿童安全及时获得安全有效的专用药物。全国人大常委会于2017年上半年开展了药品管理法执法检查，并听取审议国务院关于药品管理工作的专题报告。在执法检查报告中提出，加快药品管理法修订，大力支持和鼓励药物的研制和创新，加强对临床急需药品、短缺药品、儿童药品、罕见病用药研发等特殊人群用药保障。修改药品管理法已列入全国人大常委会立法规划。目前国家食品药品监管总局正在研究起草修改草案，拟在法律层面规定优先审评审批、有条件审批、市场独占期等鼓励政策，将儿童用药明确列入鼓励范围。我委建议国务院相关部门加快立法进程，积极研究吸纳代表所提意见和建议，不断完善儿童用药保障制度。

6．易连军等31位代表提出制定《中华人民共和国急救医疗服务法》的议案1件（第381号）。议案指出，急救医疗服务是医疗卫生事业的重要组成部分，是城市经济社会发展、精神文明建设和综合服务能力的重要标志。代表议案提出的目前急救服务工作存在一些比较突出的问题，对急救服务工作进行立法管理非常必要。目前，国家卫生计生委正在《院前医疗急救管理办法》的基础上，起草《医疗急救管理条例》。我委建议国务院相关方面认真研究代表议案提出的问题和建议，抓紧调研，加快法规起草进程，同时根据代表提出的突出问题，继续抓好政策完善和落实工作，为加快推进我国医疗急救事业全面、协调、可持续发展提供法律保障。

7．顾晋等30位代表提出修改《中华人民共和国母婴保健法》，明确我国助产士执业地位的议案1件（第114号），张艳丽等30位代表提出修改《中华人民共和国母婴保健法》的议案1件（第472号）。母婴保健法实施二十多年以来，对提升出生人口素质，保障母婴健康、规范助产人员操作及职责要求等起到了作用，目前其主要框架结构仍可以基本适应工作需要。2017年，国家卫生计生委启动了母婴保健法实施办法和计划生育技术服务管理条例合并修订研究工作。我委建议国务院相关部门认真研究代表议案提出的意见和建议，认真贯彻落实妇幼卫生工作方针，加快妇幼卫生健康服务体系建设，针对优生优育、生殖保健、助产人员地位与技能提升等所遇到的问题，统筹修订完善相关法规规章，为母婴保健法的修订完善积累经验。

（三）11件议案提出的9个立法项目，建议有关部门认真调研论证

1．周洪宇等30位代表提出的关于制定互联网教育法的议案1件（第404号）。议案指出，当前互联网及互联网教育正迅猛发展，互联网教育通过互联网技术与教育深度融合，突破了时空限制，实现教育资源利用最大化，已成为社会关注的热点和未来教育的重要形态。但目前的互联网教育泥沙俱下、良莠不齐，给我们带来无限期待的同时，也带来了许多新问题，新挑战。

国家高度重视现代信息技术在推进教育改革发展中的重要作用，积极完善相关的法律法规、政策制度。2015年12月，全国人大常委会审议通过《关于修改〈中华人民共和国教育法〉的决定》，将推进教育信息化要求上升至国家法律层面。《国家教育事业发展“十三五”规划》将积极发展“互联网＋教育”作为重要任务，提出要以教育信息化推动教育现代化，积极促进信息技术与教育的融合创新发展。

我委认为，互联网教育的实施主体、教育目标、服务对象复杂多样，其规范和管理的专业性、技术性较强。与其他教育法律比较起来，互联网教育法涉及面更广、涉及主体种类更多、法律关系更加复杂，对于各部门间的协同配合、资源统筹等机制要求更为迫切，超出了现有教育管理体制的管理范围。互联网教育发展应用中的许多问题仍在探索当中，需要开展一系列的专题研究，为立法做准备。

2．贺优琳、金硕仁、李光宇等99位代表提出关于修订义务教育法的议案3件（第42、459、423号），黄志明等31位代表关于修改教育法第二章基本教育制度第十九条的议案1件（第416号）。议案指出，2006年新修订的义务教育法及2015年修改的教育法是中国教育法制建设的重要标志，对包括义务教育在内的整个教育事业改革发展具有重要的推动作用。但时至今日，义务教育法首次修订后又过去了12年，这期间我国政治、经济、文化、社会等各方面均发生了深刻的变化。新义务教育法施行过程中也出现了一些新情况、新问题，如推动义务教育均衡发展、保障弱势群体受教育权、延长义务教育年限等问题，急需通过立法手段尽快加以解决和完善。在2015年修改教育法时仍然保留了“国家实行九年制义务教育制度”条款。部分议案还列举了一些地方已开展了12年义务教育。

教育法和义务教育法规定，我国实行九年义务教育制度。义务教育是国家统一实施的适龄儿童、少年必须接受的教育。代表在议案中针对义务教育均衡发展、问责制度、弱势儿童受教育权利、素质教育等问题所提的意见和建议，具有很强的针对性和现实意义，对于推进义务教育向更高质量、更加均衡的方向发展具有重要意义，我们将在今后的义务教育立法调研工作中认真研究，借鉴吸收。

这四件议案均提出要拓展我国的义务教育，要求把学前教育纳入义务教育。这将涉及我国基础教育的基本制度设计。我委将督请国务院有关部委加快研究，拟制出可行性方案。

3．史贵禄等30位代表提出制定《中华人民共和国抗菌药物管理法》的议案1件（第93号）。议案指出，抗菌药物滥用或不合理使用，会对患者身体健康造成不利影响，由此造成的细菌耐药，会降低治疗效果、拉长治疗周期、增加医疗支出，导致医疗资源浪费和抗菌药物资源的匮乏，给国民健康和社会造成很大危害。代表议案提出的抗菌药物管理问题非常重要，应当引起全社会的高度重视。建议国务院相关部门认真研究吸纳代表议案提出的问题和具体管理建议，加大相关规章规定的落实，加强管理和督查，适时研究制定相关管理法规。

4．周琦等32位代表提出制定肿瘤防治法的议案1件（第116号）。议案指出，随着我国老龄化进程的加快和环境污染、个人不健康生活方式等因素的持续影响，我国癌症总体发病率和死亡率呈上升趋势，恶性肿瘤的防治也一直是我国慢性疾病防治的重点和难点。《健康中国2030规划纲要》提出健康优先，把健康摆在优先发展的战略地位。包括肿瘤防治在内的慢性病防控工作是一项系统的社会工程，需要政府主导，社会各方面积极参与。建议国务院相关部门认真贯彻落实慢性病防治的相关规划，继续深入推进癌症防治体系建设，进一步完善相关配套规章，适时开展肿瘤防治立法研究论证工作。我委在推进基本医疗卫生与健康促进法等相关法律立法过程中，已认真研究了代表议案提出的具体建议，充分考虑癌症等重大疾病防控工作，以适当方式予以规定。

5．何彬生等31名代表提出建立反医疗暴力法的议案1件（第167号）。议案提出，要提高医疗暴力的预防措施、建立完善的转诊制度、提高医疗过程的公开性和透明性、完善医疗暴力的投诉和鉴定、明确相关责任人的法律责任，构建和谐的医患关系等。医疗场所暴力犯罪成因复杂，反映出当前医药卫生体制和环境中存在的一些突出问题，需要进一步加强法制建设和深化医药卫生体制改革。目前国务院法制办正在对国家卫计委报送的医疗纠纷预防与处理条例（送审稿）进行审查修改。建议国务院有关部门认真研究吸纳代表议案提出的意见和建议，及时制定完善有关法规制度，建立完善医疗纠纷预防和处理的长效机制，进一步加强医疗机构的医德医风建设，不断提高医疗服务质量，坚决打击涉医违法犯罪活动，积极维护正常的医疗秩序，持续加强宣传教育力度，努力营造尊医重卫的社会氛围。

6．瞿佳等30位代表提出制定遗体捐献法的议案1件（第430号）。议案指出，遗体捐献是高尚之举。目前医学教育过程中，尸源紧缺问题成为制约医学研究教学的难题，影响了医学和医疗卫生事业的发展。2007年3月，国务院颁布了《人体器官移植条例》，主要对人体器官移植进行了规范。加强和规范遗体捐献工作，对遗体捐献进行立法，有利于推动医学科研、教育和临床工作，也有利于移风易俗、促进社会主义精神文明建设。遗体捐献立法，涉及伦理、法律、文化、宗教等诸多领域，是遗体捐献和人体器官移植向安全、高质、高效方向发展的前提。对于代表在议案中反映的问题，建议国务院及其相关部门认真研究，逐步完善相关的配套规章，积累有关遗体捐献的管理经验，探索建立适合我国国情的遗体捐献管理制度，适时研究制定遗体捐献法律法规。

7．周洪江等30名代表提出修改食品安全法部分条款的议案1件（第469号）。议案提出，《食品安全法》中没有对食品标签“瑕疵”作出明确界定，从而对是否构成“瑕疵”难以准确认定，造成行政、司法机关在案件处理上存在理解和结果上的差异。我委建议国务院及有关部门认真研究代表议案反映的问题和意见，完善配套法规规章，待时机成熟时，再修改食品安全法。

8．黄志明等31位代表提出修改人口与计划生育法第四章奖励与社会保障第二十四条的议案1件（412号）。2013年12月28日，第十二届全国人民代表大会常务委员会第六次会议通过了《关于调整完善生育政策的决议》，启动实施一方是独生子女的夫妇可生育两个孩子的政策。2015年12月27日，第十二届全国人民代表大会常务委员会第十八次会议通过了《关于修改中华人民共和国人口与计划生育法的决定》，修改后的法律规定，国家提倡一对夫妻生育两个子女。当前，人口众多仍然是我国长期面临的基本国情，认真贯彻宪法和人口与计划生育法等法律和人口政策，促进人口长期均衡发展非常必要。随着经济社会发展，广大育龄人群的生育观念、生育意愿发生很大改变。我国人口结构已进入加速老龄化阶段，总和生育率长期低于更替水平，进一步加剧老龄化，对社会长期健康发展将造成不利影响。我委希望国务院相关部门认真研究代表议案提出的问题，加强与相关方面的沟通协调，坚持人口与发展综合决策，坚持计划生育基本国策，进一步完善计划生育政策和社会保障、税收、住房、教育等相关政策，人口集中地区要增加公共服务供给，保障女性生育权益，减轻家庭养育负担，促进人口长期均衡发展。同时，根据人口形势发展情况，研究人口与计划生育法修改完善事宜。
