# Supplementary material for: Mapping the evolution of fertility support policies in China: A content and instrumental analysis
Source: PLoS One. 2025 Oct 9;20(10):e0332137. doi: 10.1371/journal.pone.0332137 (PMC12510515; doi:10.1371/journal.pone.0332137)
Supplement: S1 Appendix — (ZIP) [file pone.0332137.s001.zip › S1 Appendix. 226 original policy documents/108-全国人民代表大会教育科学文化卫生委员会关于第十三届全国人民代表大会第二次会议主席团交付审议的代表提出的议案审议结果的报告(FBM-CLI-1-5054858).docx]

全国人民代表大会教育科学文化卫生委员会关于第十三届全国人民代表大会第二次会议主席团交付审议的代表提出的议案审议结果的报告

发布部门： 全国人大教育科学文化卫生委员会

发布日期：2019.12.28

实施日期：2019.12.28

时效性： 现行有效

效力级别： 工作文件

法规类别： 人大议事

全国人民代表大会教育科学文化卫生委员会关于第十三届全国人民代表大会第二次会议主席团交付审议的代表提出的议案审议结果的报告

（2019年12月28日第十三届全国人民代表大会常务委员会第十五次会议通过）

全国人民代表大会常务委员会：

第十三届全国人民代表大会第二次会议主席团交付全国人大教育科学文化卫生委员会（以下简称教科文卫委）审议的代表提出的议案共69件，其中教育方面27件、科技方面4件、文化方面12件、卫生健康与人口方面26件。69件议案中，建议制定法律的议案33件、修改法律的议案34件，共涉及41个立法项目，建议开展执法检查的议案2件。

按照全国人大常委会代表议案办理工作的有关规定和栗战书委员长关于做好代表议案办理工作的重要指示精神，我委高度重视，认真做好议案办理工作。一是加强研究部署。把代表议案办理摆上重要位置，注重与立法、监督等工作紧密结合，制定具体工作方案，在不同阶段召开4次专门会议，压实工作责任，推动工作落实。二是认真协商办理。及时召开有关部门参加的代表议案办理工作会议，协调分工，明确要求，促请有关部门提出议案处理初步意见。三是深入调查研究。在议案办理过程中，坚持问题导向，加强综合分析，把专题调研和工作调研结合起来，对梳理出的重点议题赴九省一市开展专题调研，与议案领衔代表直接交流，努力提高议案办理的质量和实效。四是做好沟通反馈。通过面对面交谈、电话沟通、邀请参加座谈或共同调研等方式，认真听取全部议案领衔代表和部分附议代表的意见建议，并把议案办理结果及时向代表反馈，做到件件有着落、有回音。2019年11月11日，教科文卫委召开第19次全体会议，对69件代表议案的办理意见进行审议，艾力更·依明巴海、蔡达峰副委员长参加了审议。现将审议结果报告如下：

一、21件议案提出的9个立法项目，已列入十三届全国人大常委会立法规划

（一）8件议案提出的4个立法项目列入立法规划第一类项目

1．关于制定学前教育法的议案（4件）

2．关于制定文化产业促进法的议案（1件）

3．关于修改著作权法的议案（2件）

4．关于修改文物保护法的议案（1件）

（二）13件议案提出的5个立法项目列入立法规划第二类项目

5．关于修改职业教育法的议案（2件）

6．关于修改教师法的议案（6件）

7．关于修改学位条例的议案（1件）

8．关于修改科技进步法的议案（2件）

9．关于修改执业医师法的议案（2件）

二、24件议案提出的12个立法项目，建议有关部门加强立法研究论证，条件成熟时，争取列入全国人大常委会年度立法工作计划

10．关于修改教育法的议案（2件）

11．关于修改国家通用语言文字法的议案（5件）

12．关于修改高等教育法部分条款的议案（1件）

13．关于制定终身教育法的议案（1件）

14．关于制定长城保护法的议案（1件）

15．关于制定全民阅读法的议案（3件）

16．关于修改献血法的议案（3件）

17．关于修改传染病防治法的议案（1件）

18．关于制定药师法的议案（1件）

19．关于制定执业护士法的议案（3件）

20．关于加快儿童用药立法保障儿童健康的议案（1件）

21．关于制定心理师法的议案（2件）

三、11件议案提出的11个立法项目，建议在已经确定的立法规划项目或条件成熟的立法项目中，充分吸收采纳代表议案所提出的立法建议

22．关于修改义务教育法，构建德智体美劳全面培养的教育体系的议案（1件）

23．关于修改教育法，明确教育惩戒权的议案（1件）

24．关于制定知识产权法典的议案（1件）

25．关于制定自主创新示范区法的议案（1件）

26．关于加快制定优秀历史建筑保护法的议案（1件）

27．关于制定革命文物保护法的议案（1件）

28．关于加快制定健康法，保障健康中国战略实施的议案（1件）

29．关于制定医疗法的议案（1件）

30．关于制定民办医疗管理法的议案（1件）

31．关于制定爱国卫生法的议案（1件）

32．关于进一步建立健全医学伦理法律法规的议案（1件）

四、11件议案提出的9个立法项目，建议有关部门通过修改或制定相关行政法规，加大现有法律法规的执法监督力度，对立法开展调研论证，改进相关工作，充分吸收代表建议以解决议案所提问题

33．关于修改义务教育法，延长义务教育年限的议案（2件）

34．关于对网络在线教育立法的议案（1件）

35．关于尽快制定古籍保护法的议案（1件）

36．关于完善文化立法的议案（1件）

37．关于修改人口与计划生育法的议案（2件）

38．关于制定公共场所禁烟法的议案（1件）

39．关于制定转基因食品管理法的议案（1件）

40．关于抓紧制定保健食品监督管理法的议案（1件）

41．关于启动罕见病诊疗及管理立法的议案（1件）

五、2件议案提出的2个监督项目，建议适时列入全国人大常委会监督工作计划

1．关于开展中医药法执法检查的议案（1件）

2．关于开展药品管理法执法检查的议案（1件）

上述69件议案的主要内容和具体审议意见，详见附件。

以上报告，请审议。

附件：全国人民代表大会教育科学文化卫生委员会关于第十三届全国人民代表大会第二次会议主席团交付审议的代表提出的议案的审议意见

全国人民代表大会

教育科学文化卫生委员会

2019年12月24日

附件：

全国人民代表大会教育科学文化卫生委员会关于第十三届全国人民代表大会

第二次会议主席团交付审议的代表提出的议案的审议意见

一、21件议案提出的9个立法项目，已列入十三届全国人大常委会立法规划

（一）8件议案提出的4个立法项目列入立法规划第一类项目

1．安徽代表团杨善竑等30名代表、北京代表团庞丽娟等45名代表、湖北代表团周洪宇等30名代表、天津代表团李建成等31名代表提出关于制定学前教育法的议案4件（第88号、第102号、第172号、第240号）。以上4件议案提出，近年来我国学前教育发展取得了显著成绩，但仍然是教育体系中最薄弱的短板，发展不充分不平衡，“入园难”“入园贵”等问题依然突出。建议全国人大常委会加快学前教育法立法进程，确保在本届任期内审议通过。议案对立法理念、立法重点等提出了具体建议。

党中央、国务院高度重视学前教育工作，2018年11月印发《中共中央国务院关于学前教育深化改革规范发展的若干意见》，明确了学前教育改革发展的原则与方向，为制定学前教育法提供了政策依据。十三届全国人大常委会高度重视学前教育立法，为推进这项工作，今年听取了国务院关于学前教育事业改革和发展情况的报告。教育部将学前教育立法作为今年重点任务，全面启动立法工作，已形成草案，并初步征求了地方意见。法律草案起草过程中，对近几年代表提出的意见建议进行认真研究梳理，并在草案中予以回应，计划年内形成送审稿报国务院审议。

教科文卫委经研究，同意教育部的意见，继续密切关注和积极推动学前教育立法工作。制定学前教育法列入了十三届全国人大常委会立法规划，我委已促请有关部门认真研究吸纳代表议案所提意见和建议，加快立法工作进程，尽早将法律草案提请全国人大常委会审议。

2．浙江代表团胡季强等30名代表提出关于制定文化产业促进法的议案1件（第168号）。议案提出，制定文化产业促进法是党的十八届四中全会的明确要求。近年来，我国文化产业发展迅速，成为国民经济支柱性产业的趋势日益明显。但文化产业发展中仍存在一些问题，如整体规模还不够大，地区发展不平衡等，文化产业经济政策也亟需进一步完善。加快推进文化产业领域立法工作，既是全面推进依法治国的必然要求，也是推动文化产业健康发展的客观需要。

原文化部会同有关部门于2015年启动了文化产业促进法起草工作，目前文化和旅游部已形成了法律草案稿，并于2019年6月底向社会公开征求意见。

十三届全国人大常委会第十一次会议听取和审议了国务院关于文化产业发展工作情况的报告，常委会组成人员对文化产业促进法立法工作提出了很好的意见和建议。教科文卫委已促请有关部门认真研究吸纳代表议案和常委会组成人员审议的意见和建议，加快立法进程，尽早将法律草案提请全国人大常委会审议。

3．上海代表团潘向黎等30名代表提出关于修改著作权法的议案1件（第9号）、山西代表团贾樟柯等31名代表提出关于在著作权法中给予视听作品导演及编剧作者权及收益权的议案1件（第341号）。

第9号议案提出，报刊转载法定许可制度在实施中存在一些问题，著作权人获得报酬的权益难以得到保障，建议在著作权法修改中取消报刊转载法定许可。同时建议在修法中参照专利法等法律规定，设置赔偿数额下限，切实保护著作权人权益。

国家版权局认为，报刊转载法定许可制度在实施中存在一些问题，要继续开展深入调研，听取各方意见，完善著作权法修订草案。

第341号议案提出，目前视听作品的著作权为制片者享有，导演和编剧等仅享有署名权，可获得劳动报酬，但不能享有著作权的经济收益，建议在著作权法修改中明确给予视听作品的导演和编剧等享有著作权经济收益的权利。

国家版权局认为，要对视听作品的归属问题结合电影产业等行业的发展情况进行深入研究论证，更好地平衡各方利益。

司法部认为，著作权法修订草案已于2018年11月经司法部部务会讨论通过，要平衡好作品创作、传播、使用的关系，维护好创作者、传播者、使用者三者的合法权益。同时认为议案提出的有关问题，目前还存在一定争议，下一步将在工作中研究考虑，积极推进立法进程。

教科文卫委经研究，同意司法部、国家版权局的意见，已促请有关部门在修改著作权法过程中认真研究吸纳代表议案所提意见和建议，尽早将法律修订草案提请全国人大常委会审议。

4．山西代表团王文保等30名代表提出关于修改文物保护法部分条款的议案1件（第342号）。议案提出，党的十九大以来，文物工作面临新形势新任务，为落实中央精神，让文物活起来，应加强文物的利用工作。建议：一是将文物保护利用纳入地方城乡建设规划和旅游规划；二是修改文物保护法第24条，删除“不得作为企业资产经营”的表述，并增加鼓励企业经营的相关规定；三是增加“利用传承”专章。

国家文物局认为，议案中提出的建议，将在修改文物保护法过程中认真加以研究。一是研究在修订草案中既增加鼓励社会各界参与文物合理利用的条款，也修改现行法律中不利于文物合理利用的条款。二是将文物保护法第24条作为深入研究的重点问题之一。三是在文物保护法总则中研究增加加强文物保护利用的相关制度。

教科文卫委经研究，同意国家文物局的意见，已促请有关部门在修改文物保护法过程中认真研究吸纳代表议案所提意见和建议，尽早将法律修订草案提请全国人大常委会审议。

（二）13件议案提出的5个立法项目列入立法规划第二类项目

5．湖北代表团周洪宇等30名代表、安徽代表团谢广祥等30名代表提出关于修改职业教育法的议案2件（第170号、第445号）。以上2件议案提出，职业教育发展在理念认识、性质定位、教育质量、现代职业教育体系建设等方面存在突出问题，迫切需要通过修订法律从根本上解决，以适应当前面临的新情况和新任务。建议从理顺管理体制、建立中等职业教育免费制度、适当发展本科和研究生层次高等职业教育、明确投入责任和标准、加强师资队伍建设、增加法律责任条款等方面修订完善现行职业教育法。

教育部认为，代表议案所提意见建议具有很强的现实意义，争取在本届全国人大期间完成此项立法任务。目前初步形成了职业教育法修订草案征求意见稿，代表议案中的多数建议在草案中已有回应，拟在年内提请国务院职业教育工作部际联席会议审议。人力资源社会保障部也对职业教育法提出了修改意见。

教科文卫委经研究，同意教育部、人力资源社会保障部的意见，继续密切关注和积极推动职业教育法修改工作。修改职业教育法列入了十三届全国人大常委会立法规划，我委已促请有关部门加强统筹协调，认真研究吸纳代表议案所提意见和建议，加快修法工作步伐，争取早日将法律修订草案提请全国人大常委会审议。

6．湖北代表团周洪宇等30名代表、江苏代表团王江等30名代表、河南代表团高阿莉等30名代表、四川代表团庹庆明等34名代表、辽宁代表团王家娟等30名代表、安徽代表团崔建梅等31名代表提出关于修改教师法的议案6件（第173号、第238号、第282号、第387号、第391号、第443号）。以上6件议案提出，教师法自1994年实施以来，对提高教师地位、加强教师队伍建设、保障教师合法权益、促进我国教育事业发展起到了重要作用。但随着我国经济社会发展，教育事业也发生了翻天覆地的变化，法律的一些规定已不能适应教育改革发展和教师队伍建设的实际和要求。建议加快修改教师法，确立公办中小学教师作为国家公职人员的法律地位，完善教师的权利义务、资格、待遇和奖励、法律责任等规定，尽快形成修订草案，尽早提交全国人大常委会审议。

教育部认为，修改教师法十分必要。2018年1月，中共中央国务院印发关于全面深化新时代教师队伍建设改革的意见，为教师队伍建设和教师法修改指明了方向。目前，教育部委托有关高校、专家，围绕教师队伍建设保障和奖励制度、资格和任用制度改革、培养培训、权利义务和法律责任、教师职业定位、教师法律制度的国际比较等六个方面展开研究，已经形成初步研究成果，提出修订建议，争取在2020年提请国务院审议。

教科文卫委经研究，同意教育部的意见。全国人大常委会一直密切关注教师队伍建设和教师法修改工作，在2019年审议国务院关于学前教育专项工作报告和开展高等教育法执法检查中，都将教师队伍建设问题作为重点内容。修改教师法列入了十三届全国人大常委会立法规划，我委已促请有关部门认真研究吸纳代表议案和常委会组成人员审议的意见和建议，加快立法工作进程，争取早日将法律修订草案提请全国人大常委会审议。

7．湖北代表团周洪宇等30位代表提出关于修改学位条例的议案1件（第258号）。议案提出，学位条例实施三十多年来，我国高等教育事业发生深刻变化，学位条例已不能满足当前学位管理实践的需求。建议加快修改学位条例，总结学位制度改革发展中行之有效的做法，加强顶层设计，着力解决存在问题，构建具有中国特色的学位制度。

教育部认为，修改学位条例，构建具有中国特色的学位制度意义重大。目前已明确6个方面的修订重点，力争尽快将学位条例修订草案提交全国人大常委会审议。

教科文卫委经研究，同意教育部的意见，继续关注和积极推动学位条例修改工作。修改学位条例列入了十三届全国人大常委会立法规划，我委已促请有关部门认真研究吸纳议案所提意见和建议，加大修法工作力度，争取早日将法律修订草案提请全国人大常委会审议。

8．上海代表团陈力等30名代表、河北代表团曹宝华等30名代表提出关于修改科技进步法的议案2件（第11号、第246号）。以上2件议案提出，对科技进步法进行修订完善。第11号议案提出，应重点关注培育国家战略科技力量、深度参与国际科技合作与竞争、充分保障创新主体权利、强化对科技成果转化的保障、促进科技与金融的深度融合、加大科技创新中的知识产权保护力度、优化科研人员评价和收入分配制度、依法严肃惩戒科研不端行为等内容。第246号议案提出，完善知识产权制度，增加规定激励自主创新、维护知识产权安全、明确各类创新主体职责和法律地位等内容。

科技进步法修改已列入十三届全国人大常委会立法规划，由教科文卫委负责牵头起草。我委积极落实全国人大常委会立法规划，制定修法工作方案，成立修订工作机构，已于2018年11月召开立法领导小组第一次全体会议，启动修订工作。与科技部等国务院有关部委和单位密切配合，加强调查研究，共同推进起草工作进程。先后召开政府部门及相关单位、科技界、产业界、地方科技管理机构、西部地区和基础研究专家学者参加的多场座谈会，王晨、艾力更·依明巴海、蔡达峰副委员长出席相关座谈会，并就科技进步法修改工作提出了明确要求。我委还组织多个调研组，赴地方开展立法调研。起草工作机构多次组织专题研究、专家论证，已经形成法律修改建议稿，代表议案所提出的许多修法建议已采纳。2019年11月，修改科技进步法领导小组召开第二次会议，对修改建议稿进行讨论，征求有关部委和专家意见，部署下一阶段修法工作。下一步，我委将继续深入开展相关调研，综合研究论证各方面的意见，进一步修改完善法律修订草案，争取早日提请全国人大常委会审议。

9．贵州代表团查艳等30名代表、四川代表团江吉村等31名代表提出关于修改执业医师法的议案2件（第133号、第328号）。以上2件议案提出，执业医师法实施20年来，在推动我国医师管理规范化、法治化方面起到了重要作用。随着时代进步，一些条款已不适应发展需要，如：须试用期满一年才能参加资格考试、关于执业地点限制过多、紧急情况下救治行为缺乏免责规定以及维护医师合法权益的力度不够等，均需要通过修订完善执业医师法予以解决。

执业医师法修改已列入十三届全国人大常委会立法规划，由教科文卫委负责牵头起草。我委积极落实全国人大常委会立法规划，制定修法工作方案，成立修订工作机构，已于2019年1月召开立法领导小组第一次全体会议，启动执业医师法修订工作。与国家卫生健康委及中国医师协会等有关部门和单位密切配合，加强调查研究，共同推进起草工作进程。我委以协助全国人大常委会听取和审议国务院关于医师队伍管理情况和执业医师法实施情况的报告为契机，会同有关方面认真研究吸收代表议案所提意见和建议，凝炼修法要点，提出10个重点问题，形成了执业医师法修订的初步框架草案，经2019年9月23日召开的立法领导小组第二次全体会议研究讨论。下一步，我委将综合各方面意见，深入开展研究论证，加大工作力度，争取早日将法律修订草案提请全国人大常委会审议。

二、24件议案提出的12个立法项目，建议有关部门加强立法研究论证，条件成熟时，争取列入全国人大常委会年度立法工作计划

10．湖北代表团周洪宇等30名代表、山东代表团张志勇等30名代表提出关于修改教育法的议案2件（第171号、第235号）。以上2件议案提出，教育法是我国教育工作的根本大法，2015年修订以来，以习近平同志为核心的党中央，对教育工作作出了一系列重大决策部署，对于建设教育强国，推进教育现代化，办好人民满意的教育具有重大意义。建议修改教育法总则相应条款，将党的重大理论创新成果写入法律。议案对部分条款提出具体修改建议。

教育部认为，代表议案所提意见、建议非常重要。教育部去年已着手开展教育法修订工作，深入贯彻落实2018年宪法修正案规定和习近平总书记在全国教育大会上的重要讲话精神，集中对教育法涉及的教育指导思想、地位和方针等重要条款进行了修改，代表议案提出的建议已在修改过程中认真研究吸收。经过反复论证和征求意见，教育部已于近期形成修正案（草案），提请国务院审议。

教科文卫委经研究，同意教育部的意见。我委已开展相关调研，并促请有关部门认真研究吸纳代表议案所提意见和建议，加强调研论证，条件成熟时，建议列入全国人大常委会年度立法工作计划。

11．安徽代表团杨善竑等30名代表、山东代表团张志勇等30名代表、河南代表团黄艳、马玉霞等60名代表、江苏代表团葛道凯等30名代表提出关于修改国家通用语言文字法的议案5件（第93号、第257号、第283号、第384号、第484号）。以上5件议案提出，国家通用语言文字法自2000年颁布以来，有力促进了国家通用语言文字推广普及和规范应用进程，为推进语言文字事业治理体系、治理能力现代化和实现依法治理创造了良好的条件。但随着经济社会的发展和信息化步伐的加快，语言生活日益纷繁复杂，法律在贯彻实施中遇到一些新问题、新挑战。建议进一步强调国家通用语言文字的地位和使用原则，适当扩大国家通用语言文字法的调整范围，明确各相关行业领域及从业人员使用国家通用语言文字的要求，明确汉语方言、繁体字异体字、少数民族语言文字、外国语言文字、国家通用盲文手语、网络空间语言文字的使用要求，厘清各级政府和部门的职责、细化奖惩措施等。

教育部认为，修订国家通用语言文字法是满足新时代事业发展、人民生活之所需。2013年以来，教育部围绕修法和制订国家通用语言文字法实施办法已经开展调研论证工作。2018年形成法律修订草案，围绕繁体字、方言、外语政策等召开10次专题座谈会，两次书面征求各有关方面的意见建议。法律草案主要从进一步突出国家通用语言文字的主体地位，加强网络空间语言文字监管，妥善处理语言文化多元现象，强化外国语言文字使用管理，促进法律贯彻实施等5个方面进行修订，认真吸收代表议案所提建议。教育部将扎实做好修法工作，争取早日提请全国人大常委会审议。

教科文卫委经研究，同意教育部的意见。我委已促请有关部门充分研究吸纳代表议案所提意见和建议，在已有工作基础上，进一步开展调研论证，条件成熟时，建议列入全国人大常委会年度立法工作计划。

12．湖南代表团杨尚真等30名代表提出关于修改高等教育法第3条、第32条、第53条促进一流高校招生计划均衡配置的议案1件（第329号）。议案提出，推动一流高校招生计划均衡配置有利于促进区域协调发展，有利于促进良好教育生态重构。建议通过修订高等教育法对生源配置进行规范，将其制度化、透明化、法治化。议案还提出对发展高等教育事业的指导思想进行完善，增加法律责任、明确惩罚规定等建议。

教育部认为，考试招生制度是国家基本教育制度，我国考试招生制度总体上符合国情，权威性、公平性得到社会认可。高考改革涉及城乡区域管理、社会保障、户籍制度改革、区域教育资源配置等诸多方面。代表议案提出的完善高等教育事业指导思想的意见建议十分重要。目前，教育部已启动教育法的修订，拟将有关内容写入教育指导思想。由于教育法在教育法律体系中居于基础地位，修改后可对高等教育法等其他教育法律发挥引领作用。

2019年，全国人大常委会对高等教育法贯彻实施情况进行执法检查，把“深化考试招生制度改革情况”作为检查的重点内容之一。王晨副委员长代表执法检查组所作的报告指出，考试招生制度改革还需加大力度，改革的整体性和协同性有待增强。检查组建议从加快发展高等教育的新形势新要求出发，适时修改高等教育法，将党的重大理论创新成果写入法律，把近年来国家和地方在高等教育改革发展中取得的成功经验和制度创新成果上升为法律。

教科文卫委已促请教育部等有关部门认真研究吸纳代表议案和高等教育法执法检查组的意见、建议，抓紧开展修法调研论证，适时列入全国人大常委会年度立法工作计划。

13．河南代表团买世蕊等30名代表提出关于制定终身教育法的议案1件（第299号）。议案提出，终身教育对于促进人的全面发展和社会进步具有重要意义。建议制定终身教育法，明确终身教育的组织实施、保障措施、监督管理、法律责任等，从战略发展的高度来规范和指导终身教育的开展，也为地方立法提供上位法依据。

教育部认为，宪法和有关法律对终身教育相关内容作了规定。目前全国有八个省市开展了有关终身教育的地方立法。考虑到终身教育法律制度的健全与完善还需要更多实践经验和理论成果支持，拟先在职业教育法等法律修订过程中，强化终身教育相关内容，推动健全终身教育体系。同时，在进一步深化终身教育立法研究基础上，拟于2020年启动终身教育法草案的起草工作。

教科文卫委经研究，同意教育部的意见。党的十九届四中全会明确提出，构建服务全民终身学习的教育体系，这是推进国家治理体系和治理能力现代化的重要举措。我委已促请有关部门认真贯彻党的十九届四中全会精神，研究吸纳代表议案所提意见和建议，抓紧开展立法调研论证，条件成熟时，建议列入全国人大常委会年度立法工作计划。

14．河北代表团提出关于制定长城保护法的议案1件（第169号）。议案提出，长城是中华民族的精神象征，是世界上现存体量最大、分布最广的历史文物。目前长城保护面临着多种自然病害和人为损坏的威胁，制约长城保护的诸多困难与问题尚未得到有效解决，建议制定长城保护法。

国家文物局认为，长城保护条例颁布实施十多年来，对于加强长城保护发挥了重要作用，但也需要根据经济社会发展和保护工作实践进行修订。在修订文物保护法时，对长城等大型文化线路遗产保护存在的突出问题予以重点关注。同时，加强长城保护总体规划的实施力度，在推进长城保护条例修订的基础上，开展长城保护法的立法论证。

教科文卫委经研究，同意国家文物局的意见。我委已组织开展专题调研，并促请有关部门加强关于长城保护的立法研究，条件成熟时，建议列入全国人大常委会年度立法工作计划。

15．河北代表团陈凤珍等32名代表、河南代表团买世蕊等30名代表提出关于制定全民阅读法的议案2件（第486号、第311号），山东代表团张淑琴等30名代表提出关于制定全民阅读促进法的议案1件（第261号）。以上3件议案提出，开展全民阅读对于提高公民科学文化素质、建设学习型社会和提升我国文化软实力等都具有重要意义。近年来，全民阅读活动不断深入，一些地方制定了相关法规，但仍存在资源不均衡、经费保障不到位等问题，需要国家通过立法加以保障。

中央宣传部认为，建立促进全民阅读的长效机制有其必要性。2013年3月，原国家新闻出版广电总局正式启动全民阅读促进条例的起草工作，目前已形成了一定成果，正在研究进一步加强全民阅读法治保障的措施。同时，全国已有8个省、市，通过地方立法促进全民阅读，全民阅读立法具有良好的工作基础，中央宣传部将继续就全民阅读立法的必要性和可行性开展进一步研究。

教科文卫委经研究，赞同中央宣传部的意见，已促请有关部门在已有工作基础上，认真研究代表议案提出的意见和建议，并及时总结地方以立法促进全民阅读的经验和做法，抓紧开展立法调研论证，条件成熟时，建议列入全国人大常委会年度立法工作计划。

16．河北代表团鲍守坤等30名代表、江苏代表团李叶红等30名代表、湖北代表团王岚等42名代表提出关于修改献血法的议案3件（第167号、第241号、第477号）。以上3件议案提出，献血法部分条款已不适应当前实际需要，建议进一步明确部门责任，建立健全地方政府领导、多部门合作、全社会参与的无偿献血长效工作机制，修改献血年龄、间隔周期、献血量等指标，完善无偿献血激励制度，确立志愿者组织在献血工作中的法律地位等。

国家卫生健康委认为，献血法实施以来，有关方面全面落实无偿献血制度，不断完善法律制度体系，改进血液质量控制系统，健全临床用血管理制度，血液供应水平、依法治理水平、血液安全水平及合理用血水平持续提升。但在取得显著成效的同时，也确实存在代表议案所反映的问题。国家卫生健康委已将献血法修订列入2018年该委立法工作计划，正在组织调研，征求有关方面的意见，初步形成了修订草案。

教科文卫委经研究，同意国家卫生健康委的意见，建议有关部门在修改献血法过程中，认真研究吸纳代表议案所提意见和建议，并加大科学献血的宣传力度，普及有关科学知识，及时总结经验，深入开展修法调研论证，条件成熟时，建议列入全国人大常委会年度立法工作计划。

17．福建代表团郑奎城等33名代表提出修改传染病防治法的议案1件（第174号）。议案提出，调整法定传染病分类、增加病种，加强学校等重点场所传染病防控，建立传染病防治联席会议制度，形成政府主导、部门联动、社会参与的传染病防控局面，完善传染病疫情事件通报制度，及时回应社会关切。

传染病防治工作事关人民群众的身体健康和生命安全，事关国民经济发展和社会稳定。全国人大常委会对此高度重视，于2018年对传染病防治法进行执法检查，指出传染病防治法的部分条款已不能适应当前防治工作的需要，建议研究修订传染病防治法等相关法律。目前，国家卫生健康委已将修订传染病防治法及其实施办法列入该委立法工作计划，组织专家对相关问题进行研究论证。教育部、海关总署等部门也在组织研究修订学校卫生工作条例、国境卫生检疫法及其实施细则。

教科文卫委经研究，同意国家卫生健康委等部门的意见，已促请有关部门认真研究吸纳代表议案所提意见和建议，深入开展调查研究，加大修法工作力度，条件成熟时，建议列入全国人大常委会年度立法工作计划。

18．河北代表团王连灵等31名代表提出制定药师法的议案1件（第244号）。议案提出，我国居民不合理用药问题普遍存在，涉药安全事件屡有发生，药师队伍数量不足、素质参差不齐，职称药师与执业药师双轨并行，队伍管理混乱。建议制定药师法，规范药师执业，保障居民用药安全。

国家卫生健康委认为，制定药师法将进一步规范我国药师管理工作，改变目前药师管理无法可依的现状。2019年，已联合国家药监局成立药师法起草专家组，加强立法研究和起草工作，尽快形成药师法草案。

教科文卫委经研究，同意国家卫生健康委的意见，已促请有关部门认真研究吸纳代表议案所提意见和建议，加大工作力度，加强调研论证，加快立法进程，条件成熟时，建议列入全国人大常委会年度立法工作计划。

19．湖南代表团胡春莲等37名代表、河南代表团黄玉梅等30名代表、河南代表团宋静等31名代表提出制定执业护士法的议案3件（第264号、第306号、第476号）。以上3件议案提出，目前存在临床一线护士数量配备不足、职业安全保障不健全、护士待遇偏低和职业尊严感不高等问题，影响到护士队伍健康发展，也影响到为人民群众提供健康服务的供给。建议制定执业护士法，维护护士合法权益，促进护士队伍建设。议案还提供了执业护士法建议草案。

国家卫生健康委认为，2008年护士条例颁布施行以来，从法规层面进一步维护护士的合法权益，规范护理行为。2019年，已将护士条例修订列入立法计划，并适时推动执业护士法的立法工作。

教科文卫委经研究，同意国家卫生健康委的意见，已促请有关部门认真研究吸纳代表议案所提意见和建议，加大护士条例等现有规章制度的实施力度，加强立法研究，条件成熟时，建议列入全国人大常委会年度立法工作计划。

20．江苏代表团李甦雁等30名代表提出关于加快儿童用药立法，保障儿童健康的议案1件（第389号）。议案提出，近年来儿童用药供给不足，剂型、用药信息缺乏，不合理用药现象普遍，药物不良反应率偏高等问题突出。相关部门为解决儿童用药问题出台了不少文件规定，但层级不够高，没有明确的法律依据，不能系统解决儿童用药存在的诸多问题。建议开展专门的儿童用药立法，为儿童用药提供强有力的法治保障。

国家卫生健康委认为，2014年原国家卫生计生委等6部门制定《关于保障儿童用药的若干意见》（国卫药政发〔2014〕29号），从鼓励研发创制、加快申报审评、确保生产供应、强化质量监管、推动合理用药、完善体系建设、提升综合能力等环节，对保障儿童用药提出了具体要求。相关部门对照职责分工，陆续出台细化配套措施，积极推动各项部署落地生效，保障儿童用药工作取得一定成效。但儿童用药的确还存在代表议案中提到的一些问题，主要原因是相对成人药，儿童用药市场需求量小，研发、生产成本高，儿科人群药物临床试验难度大，企业对研发、生产儿童药普遍缺乏积极性。目前，已启动儿童用药立法研究，并将在全国范围内开展立法可行性调查，广泛征求社会各方面意见，加快推进儿童用药立法工作。

教科文卫委经研究认为，儿童用药关系我国亿万儿童身体健康，多年来受到全国人大代表和社会各界的高度关注。相关部门针对儿童用药出台了多项举措和办法，为儿童用药立法提供了一定基础。我委已促请有关部门认真研究吸纳代表议案提出的问题和建议，加强儿童用药立法研究论证，条件成熟时，建议列入全国人大常委会年度立法工作计划。

21．河南代表团赵国祥等30名代表、浙江代表团蔡继明等31名代表提出关于制定心理师法的议案2件（第307号、第330号）。以上2件议案提出，近年来，我国国民心理健康问题高发，心理健康服务领域存在不少问题，心理健康服务管理依据不足，亟需进行专门立法，规范和促进心理健康服务行业健康发展。建议制定心理师法，对心理师的概念、从业资格和考试、管理体制、执业范围等方面进行规定。

国家卫生健康委认为，制定心理师法的议案具有较强的针对性和现实意义。在进一步贯彻落实精神卫生法有关规定的同时，对心理健康服务规范管理等问题开展深入调研，研究制定心理健康服务规范管理的相关文件或专门法律。

教科文卫委经研究，同意国家卫生健康委的意见，已促请有关部门认真研究吸纳代表议案所提意见和建议，加强立法研究论证，推动立法进程。

三、11件议案提出的11个立法项目，建议在已经确定的立法规划项目或条件成熟的立法项目中，充分吸收采纳代表议案所提出的立法建议

22．广东代表团阎武等43名代表提出关于修订义务教育法，构建德智体美劳全面培养的教育体系的议案1件（第63号）。议案提出，习近平总书记在全国教育大会上指出，要遵循教育规律，培养德智体美劳全面发展的社会主义建设者和接班人。建议通过修订义务教育法，从法律层面推动构建德智体美劳全面培养的教育体系，并增加减负提质的内容。

教育部认为，构建德智体美劳全面培养的教育体系是落实全国教育大会精神和习近平总书记重要讲话精神的明确要求。目前，教育部已经启动修改教育法，明确将“德智体美劳”全面发展写入第五条的教育方针当中。由于教育法在教育法律体系中居于基础性地位。教育法的修改，可对教育领域其他法律发挥引领作用。关于在义务教育法中增加减负提质的表述。义务教育法规定，要推进实施素质教育，要求学校把德育放在首位，保证学生的课外活动时间，组织开展文化娱乐等课外活动。教育部出台了《关于规范校外培训机构发展的意见》等文件，规范培训机构，开展专项治理。代表议案提出的建议，现行法律已有原则规定，相关文件中也规定了具体举措。目前，需要总结实践经验，进一步完善规定，加大实施力度。

教科文卫委经研究，同意教育部的意见。修改教育法可对义务教育法等教育领域法律发挥引领作用，我委已开展相关研究，并促请有关部门在修改教育法等法律时认真研究吸纳代表议案所提意见和建议；同时全面深入贯彻落实义务教育法和有关文件规定，大力实施素质教育，以减轻学生过重的课业负担。

23．河北代表团陈凤珍等31名代表提出关于修改教育法明确教育惩戒权的议案1件（第245号）。议案提出，应当通过立法明确界定、规范和保护教育惩戒权，保障教师全面履行职责，保护学生合法权益，弥补家庭教育不足，更好地促进学生健康成长。建议修改教育法，明确教师的教育惩戒权，并在教师法中对教育惩戒权的实施方式、限度以及相关权利救济途径作出规定。

教育部认为，现有相关法律中虽然没有直接使用“惩戒”的概念，但教育法、未成年人保护法等法律对学校及其他教育机构的惩戒权已有了相关规定。近期中共中央国务院印发的《关于深化教育教学改革全面提高义务教育质量的意见》要求，“制定实施细则，明确教师教育惩戒权”，教育部正在落实文件精神，着手起草相关规章。正在修订的未成年人保护法拟进一步明确对有不良行为的学生实施惩戒的规定。同时，在教师法的修订工作中也将对此重点研究吸收。

教科文卫委经研究，同意教育部的意见，已促请有关部门结合相关立法和监督工作，认真研究吸纳代表议案所提意见和建议，在修改教师法等相关法律的过程中对教育惩戒权问题予以重点研究明确。

24．上海代表团刘晓云等31名代表提出的关于制定知识产权法典的议案1件（第17号）。议案提出，知识产权立法体系化欠缺，法律规定不够完善，制约了知识产权司法保护作用进一步发挥，难以应对创新发展形势下的保护需求。为形成协调统一的知识产权法律体系，加大知识产权保护力度，建议编纂知识产权法典，确定知识产权的基本原理和程序规则，建立统一的侵权赔偿制度和符合知识产权案件特点的诉讼制度。

中央宣传部认为，在新的历史阶段，是否要制定知识产权法典，取决于条件时机、理论积累和制度实践等各种主客观因素，可以就立法的必要性和可行性进行深入调研论证。

最高人民法院认为，我国知识产权法律体系确实存在立法层级不一、体系化不健全等问题，但基本适应我国司法实践的需求。如果完全推翻现有的单行法体系，重新制定一部大而全的知识产权法典，在一定程度上会造成立法资源的浪费。

市场监管总局认为，代表议案提出制定知识产权法典等内容，对加强知识产权保护具有积极意义。目前可以结合我国实际，在充分发挥现有法律法规基础上统筹考虑，坚持问题导向，进一步修订完善相关法律法规，健全知识产权法律体系。

国家知识产权局认为，制定知识产权法典或知识产权基本法有利于在基本制度层面解决知识产权法律体系化、系统化、共同性以及前瞻性的问题，是全面完善知识产权保护法律体系的重要内容，也是落实《国家知识产权战略纲要》的关键举措。

就代表议案所提问题，全国人大常委会已对反不正当竞争法、行政许可法作了修改，专利法和著作权法修改已列入十三届全国人大常委会立法规划，其中专利法修正案（草案）已经十三届全国人大常委会第七次会议初审。

教科文卫委经研究认为，中办发〔2019〕56号文件《关于强化知识产权保护的意见》要求，“研究制定知识产权基础性法律的必要性和可行性，加快专利法、商标法、著作权法等修改完善”，是十分重要的。建议有关部门认真贯彻党中央、国务院决策部署，充分吸收代表议案所提意见，对知识产权基础性法律的有关问题进行深入研究，并继续抓紧推动知识产权单行法律的修改完善。

25．湖南代表团梁庆凯等30名代表提出的关于制定自主创新示范区法的议案1件（第438号）。议案提出，国家应加大对自主创新示范区建设的指导和支持力度，制定自主创新示范区法，为自主创新示范区在创新型国家建设中作出更大贡献提供法律保障。

科技部认为，国家自主创新示范区建设模式、运行机制、管理模式等各自有所不同，各地仍处在探索阶段，从国家层面出台国家自主创新示范区相关法律法规的时机目前暂不成熟。同时表示，积极支持有关地方根据实际情况，研究出台地方性法规、行政规章等规范性文件，推动国家自主创新示范区加快创新驱动发展。

教科文卫委经研究，同意科技部的意见。我委在组织开展科技进步法的修改过程中，将认真研究吸纳代表议案所提意见和建议，同时促请相关部门积极支持有关地方探索制定相应地方性法规。

26．上海代表团杲云等30名代表提出关于制定优秀历史建筑保护法的议案1件（第10号）。议案提出，优秀历史建筑是城市的重要名片，具有重要的保护价值。由于保护工作综合性较强，涉及产权归集、土地供应、修缮利用和资金安排等众多环节，虽然国家在不同层面出台了若干政策文件，但尚缺乏系统、整体、全面的反映保护要求的法律法规，建议制定优秀历史建筑保护法。

住房城乡建设部认为，历史建筑是中华优秀文化的重要载体，议案所提的建议对理顺优秀历史建筑保护机制，完善优秀历史建筑保护立法工作具有重要意义。2008年施行的《历史文化名城名镇名村保护条例》对历史建筑的保护提出严格要求，同时《历史文化名城名镇名村保护条例》因制定时间较早，随着经济社会的发展，执行中面临一些问题。当前需要尽快启动《历史文化名城名镇名村保护条例》修订，在此基础上做进一步调研、完善后再上升为法律。

国家文物局认为，优秀历史建筑是我国历史文化遗产的重要组成部分，代表议案针对当前优秀历史建筑保护存在的问题具有较为深刻的认识，将在文物保护法修订中加强对优秀历史建筑保护的立法研究，在不可移动文物保护中予以统筹考虑。

教科文卫委经研究，同意住房城乡建设部和国家文物局的意见，已促请有关部门在修改文物保护法过程中认真研究吸纳代表议案所提意见和建议，同时加快《历史文化名城名镇名村保护条例》的修改完善工作，进一步完善优秀历史建筑保护方面的法律法规。

27．安徽代表团韩再芬等30名代表提出关于制定革命文物保护法的议案1件（第458号）。议案提出，革命文物蕴含着中华民族和中国共产党人的崇高精神价值与优良革命传统，具有重要纪念意义、教育意义及史料价值。近年来，革命文物相关工作不断强化，但仍存在一些亟需改进的问题，如部分革命遗址未能及时列为相应级别的文物保护单位，一些革命文物保护不力，文物保护与基建存在矛盾，革命文物利用途径单一，管理体系不完善等，建议尽快制定革命文物保护法。

国家文物局认为，党中央高度重视革命文物保护，2018年7月中共中央办公厅、国务院办公厅出台了《关于实施革命文物保护利用工程（2018—2022年）的意见》，对新时代革命文物保护工作进行了全面部署。目前结合文物保护法修订，进一步明晰革命文物内涵外延，增加革命文物保护利用条款。同时，开展革命文物保护行政法规制定工作，鼓励革命文物资源密集地区加强地方立法。

教科文卫委经研究，同意国家文物局的意见，已促请有关部门在修改文物保护法过程中认真研究吸纳代表议案所提意见和建议，抓紧完善和落实相关法律法规，进一步提升依法保护利用革命文物的水平。

28．天津代表团张伯礼等32名代表提出关于加快制定健康法，保障健康中国战略实施的议案1件（第236号）。议案提出，我国居民面临老龄化问题突出、慢病负担加重以及亚健康困扰等严峻的健康挑战，部分青少年的健康状况也令人担忧，建议制定健康法，明确政府职责，形成由卫生健康主管部门牵头，各部门配合联动的管理体制和工作机制。议案还提出，建立全民健康档案信息数据库，开展全民健康教育，开设健康频道，制定餐饮业膳食营养限量标准等。

全国人大常委会法工委认为，基本医疗卫生与健康促进法的重要立法目的就是要推动和保障健康中国战略的实施，为实现全方位全周期维护人民健康提供法治基础。草案设“健康促进”专章，规定了政府和社会在构建健康支持性环境中的职责和任务。同时，据代表议案所提建议和各方面意见，对草案进行修改完善，进一步充实了健康促进方面的内容。建议综合考虑基本医疗卫生与健康促进法草案现有规定与议案所提建议的关系，对制定健康法的必要性、可行性等问题进行统筹研究。目前法工委正在根据常委会三次审议和征求社会意见的情况，进一步修改完善基本医疗卫生与健康促进法草案。

教科文卫委经研究，同意法工委的意见。我委将配合法工委进一步研究吸收代表议案所提建议，继续做好基本医疗卫生与健康促进法草案的修改完善工作。

29．浙江代表团陈爱莲等30名代表提出关于制定医疗法的议案1件（第36号）。议案提出，近年来我国医疗纠纷多发，医患关系引发的矛盾日益增多。构建和谐医患关系，有必要制定一部医疗法，从根本上解决医患关系问题。议案建议在医患双方基本权利义务、医疗主体制度、医疗行为监管、医疗纠纷解决途径、医疗风险化解机制等方面予以规定。

国家卫生健康委认为，2018年国务院颁布《医疗纠纷预防和处理条例》，内容包括从源头上预防医疗纠纷、及时化解医疗纠纷等，对完善多元化解机制，保护医患双方合法权益，构建和谐医患关系，提升医疗纠纷预防和处理工作法治化水平，具有重要作用和意义。代表议案涉及的相关内容在《医疗纠纷预防和处理条例》中已体现，下一步要加大条例的贯彻实施力度。

教科文卫委经研究认为，全国人大常委会正在审议的基本医疗卫生与健康促进法草案的第八章第九十六条，已对建立医疗纠纷预防和处理机制，妥善处理医疗纠纷，维护医疗秩序提出了总体要求。第二章、第四章的多项条款对患者和医务人员的权利义务及相互关系作出了明确规定。建议有关部门在法律草案修改完善过程中，充分研究吸纳代表议案所提意见。同时，已促请有关部门加大《医疗纠纷预防和处理条例》实施力度。

30．陕西代表团崔荣华等30名代表提出关于制定民办医疗管理法的议案1件（第71号）。议案提出，我国民营医院数量众多，床位数、诊疗人数规模巨大，但目前没有专门的医疗法或医疗机构管理法，民办医疗机构主要依据医疗机构管理条例和部门规章管理，层级较低，约束力有限，市场乱象频发，应该通过立法加强管理。同时，民办医疗机构的地位、权益，也需要通过立法予以保障，建议制定民办医疗管理法。

国家卫生健康委认为，社会办医是我国医疗卫生服务体系的重要组成部分，对增加医疗资源有效供给，满足人民群众多层次、多样化健康服务需求具有重要意义。深化医药卫生体制改革以来，党中央、国务院出台了一系列政策文件，支持、鼓励社会力量举办医疗机构，使社会办医与公立医疗机构享受同等待遇，促进共同发展。下一步，会同有关部门加强调查研究，进一步完善政策措施，为社会办医立法做好前期准备。

教科文卫委经研究认为，全国人大常委会正在审议的基本医疗卫生与健康促进法草案第三章，对社会力量举办的医疗机构的法律地位、优惠举措、享有权利等作了明确规定。我委将在修改完善该项法律草案过程中，认真研究吸纳代表议案所提意见。同时，建议有关部门加强调查研究，加大工作力度，推动解决民办医疗机构发展中遇到的困难和问题。

31．广东代表团段宇飞等34名代表提出关于制定爱国卫生法的议案1件（第61号）。议案提出，爱国卫生运动是群众路线运用于卫生防病工作的伟大创举和成功实践。新的发展时期影响健康的因素日益复杂，城市卫生管理面临挑战，群众健康素养有待提升，爱国卫生工作面临不少新情况、新问题，需要纳入法治化轨道。议案提出了爱国卫生法草案稿，建议尽快推进立法进程。

国家卫生健康委认为，在经济社会快速发展、人民群众观念及行为方式发生很大变化的背景下，爱国卫生运动需要运用法治思维、按照法治方法、遵循法治路径予以推进。目前全国已有28个省（区、市）颁布了爱国卫生地方性法规，为全国立法奠定了基础。自2015年开始，全国爱卫办启动爱国卫生立法研究工作，在委托研究、深入调研基础上，起草了《爱国卫生条例》（草稿），目前正在征求意见。下一步，全国爱卫办将把此项立法作为重点工作予以积极推进。

教科文卫委经研究，同意全国爱卫办的意见，建议在基本医疗卫生与健康促进法草案修改完善过程中，认真研究吸纳代表议案所提意见。同时，建议有关部门积极推进爱国卫生条例的制定，尽早颁布实施，为将来立法提供实践基础。

32．江苏代表团王静成等30名代表提出关于进一步建立健全医学伦理法律法规的议案1件（第392号）。议案提出，随着生物医学技术的进步，涉及人的临床试验、科学研究、器械验证项目越来越多。我国医学伦理工作起步较晚，相关伦理规范有待进一步健全，医学伦理水平与科技发展速度严重不匹配，伦理审查缺乏执行力，违背伦理事件时有发生，形势严峻。建议建立健全医学伦理法律，并提出了需要遵循的制度原则。

国家卫生健康委认为，目前对于医学伦理审查的法律监管体系已初步形成，伦理审查相关的制度也基本建立，下一步将继续推动相关工作，健全伦理审查监督管理体系。

教科文卫委经研究，在科技进步法修改、基本医疗卫生与健康促进法草案修改完善过程中，认真研究吸纳代表议案所提意见。同时，促请国家卫生健康委抓紧修订完善《涉及人的生物医学伦理审查办法》，加大有关医学伦理审查规范性文件的实施力度。

四、11件议案提出的9个立法项目，建议有关部门通过修改或制定相关行政法规，加大现有法律法规的执法监督力度，对立法开展调研论证，改进相关工作，充分吸收代表建议以解决议案所提问题

33．河南代表团李光宇等30名代表提出关于修改义务教育法，将学前教育纳入义务教育范围的议案1件（第115号），河南代表团买世蕊等30名代表提出关于修改义务教育法，逐步将九年义务教育延长为十三年义务教育的议案1件（第284号）。以上2件议案提出，义务教育是教育工作的重中之重，是提升国民素质的奠基性工程，建议修改义务教育法，延长义务教育培养年限。第115号议案提出，将九年义务教育制度扩大到十二年义务教育，把学前教育阶段纳入义务教育范围，推行幼儿园三年的全免费教育，学前教育阶段实行“一免一补”政策。第284号议案提出，将九年义务教育制度修改为十三年义务教育制度，鼓励各地逐步实行十五年义务教育制度，普及学前教育和高中教育。

教育部认为，关于义务教育延长为13年或15年、将学前教育纳入义务教育范围等问题，相关部门进行过多次专门研究，也广泛听取过社会意见。各方面普遍认为，目前修订义务教育法延长义务教育年限的条件尚不成熟。主要考虑：一是从义务教育性质和面临的困难看，义务教育是国家依法统一实施、所有适龄儿童少年必须接受的教育，具有普及、免费、均衡和强制的特点。由于我国全面普及九年义务教育时间较短，义务教育均衡发展的基础依然薄弱，城乡、区域、校际差距还较大，当前和今后一个时期巩固九年义务教育仍然是教育工作的重要任务。二是从发展阶段看，我国仍处于社会主义初级阶段，当前面临经济下行压力大、财政收支矛盾突出等新形势，贫困地区教育发展面临的任务依然十分艰巨，确保贫困地区学生“有学上、上好学”等方面还有不少短板和薄弱环节。财政教育投入需要坚持雪中送炭，既尽力而为，又量力而行。三是从体制机制看，学前教育当前主要应明确政府承担更多责任、加大投入，合理分担教育成本，尽快解决“入园难”“入园贵”问题。对于学前教育是否应该具有强制性，各界看法不一，需要进一步研究论证。高中教育尚未全面普及，投入保障机制还不完善、学校发展很不均衡，虽然一些地区探索在高中阶段实行免费教育，但并不是将高中阶段教育纳入义务教育。

教科文卫委经研究，同意教育部的意见，已促请有关部门认真研究代表议案所提意见，通过推进学前教育法立法进程，加快构建公益普惠、有质量的学前教育体系；加强调查研究，完善教育领域现行的法律法规；加大监督工作力度，着力解决代表议案所提问题。

34．山西代表团牛三平等34名代表提出关于对网络在线教育立法的议案1件（第64号）。议案提出，当前网络在线教育快速发展的同时，存在机构间恶性竞争、无资质办学、学费被挪用等乱象，但有效监管难。建议制定网络在线教育法，明确在线教育经营者的资质审核与登记许可要求，规范网络在线教育经营者的权利义务，明确监督管理部门及其职责，理顺网络在线教育争议解决途径。

教育部认为，党的十九大报告提出办好网络教育。依法加强网络在线教育管理，具有很强的现实必要性和紧迫性，但目前制定专门法律的时机还不够成熟。市场监管总局认为，进一步论证对网络在线教育单独立法的必要性和可行性，由相关部门根据实践基础和现实需要进行深入研究。工业和信息化部认为，要进一步加强对网络在线教育的监督管理。

教科文卫委经研究，同意教育部、市场监管总局、工业和信息化部的意见，已促请有关部门认真研究代表议案所提意见，制定和完善相应行政法规，为国家层面相关立法积累经验、提供支撑。同时，进一步加大监管力度，严格依法查处违法行为，引导和规范网络在线教育健康发展，着力解决议案所提问题。

35．贵州代表团曾丽等30名代表提出关于尽快制定古籍保护法的议案1件（第262号）。议案提出，古籍是中华优秀传统文化传承发展的重要载体，妥善保护和利用古籍，对继承和弘扬中华民族优秀传统文化，保障国家文化安全，维护中华民族的团结、和谐与统一，具有十分重大而深远的意义。近年来，尤其是“中华古籍保护计划”启动以来，全国古籍保护工作取得了重要阶段性成果，但是仍存在一些突出矛盾和问题，亟需通过国家立法加以规范。

文化和旅游部认为，代表议案针对古籍保护现状中存在的突出问题和法律法规空白，提出加快推动古籍保护立法，具有重要现实意义。要充分吸收议案的建议，积极推动古籍保护条例制定工作，鼓励和支持地方立法实践。

教科文卫委经研究，同意文化和旅游部的意见，已促请有关部门在制定古籍保护条例过程中认真研究吸纳代表议案所提意见和建议，加大古籍保护力度，并积极开展相关立法前期研究。

36．江苏代表团刘忠斌等30名代表提出关于完善文化立法的议案1件（第388号）。议案提出，社会主义文化繁荣兴盛离不开法治保障。当前文化法治建设取得了不少成果，但仍存在一些不足，如缺少统筹文化领域部门法的“基本法”、文化领域仍存在部分立法空白、相关法律配套规章和实施细则未及时出台等。建议制定文化领域基本法，完善文化法律体系，推动已出台法律的配套规章、实施细则的制定，加强执法。

文化和旅游部认为，以习近平同志为核心的党中央对文化立法高度重视，从立法理念、指导思想和具体项目方面对文化立法工作提出了新的更高要求。近5年来，随着公共文化服务保障法、公共图书馆法、博物馆条例等法律法规和一大批部门规章、地方性法规规章颁布实施，文化领域法律法规数量大幅度增加，有力改变了文化领域立法长期以来较为薄弱的局面。下一步，将加快制定文化产业促进法，加快研究出台衔接、配套的文化法规和政策，积极推动地方文化立法，加强对法律实施情况的检查。

教科文卫委经研究，同意文化和旅游部的意见，已促请有关部门认真研究代表议案所提意见和建议，加快文化产业促进法的立法进程，抓紧制定完善文化领域法律的配套法规规章，不断完善文化法律体系。同时，建议全国人大常委会在已有监督工作基础上，适时安排开展文化领域法律，如公共文化服务保障法等执法检查，更加有效推动相关法律贯彻实施。

37．陕西代表团史贵禄等30名代表、安徽代表团潘保春等30名代表提出关于修改人口与计划生育法的议案2件（第72号、第444号）。以上2件议案提出，当前我国生育率下降与人口老龄化叠加，对国家经济持续稳健发展构成威胁。2016年实施的二孩政策已不适应形势发展的需要。建议修改人口与计划生育法，废除对生育权、生育数量限制的内容，废除授权地方政府对计划生育进行规定的内容，废除对社会抚养费的规定，明确鼓励生育的措施，建立国家生育登记准备和引导机制，加大积极生育和优生优育宣传和服务保障力度等。

国家卫生健康委认为，党的十八大以来，以习近平同志为核心的党中央科学把握人口发展规律，从实现中华民族伟大复兴的战略高度出发，先后启动实施单独两孩和全面两孩政策，迈出生育政策调整完善的重要步伐。对现行人口与生育政策的调整，要统筹考虑我国的人口国情、财政状况以及各地经济社会发展水平和承受能力，需要作进一步的深入研究论证，科学评估政策实施效果，加强出生人口监测预测，推动完善政策法规和措施，更加充分地做好立法和政策储备。

教科文卫委经研究，同意国家卫生健康委的意见，已促请有关部门在相关工作中认真研究吸纳代表议案所提意见。我委将进一步加强人口与生育方面政策、法规的调查研究。

38．河南代表团买世蕊等30名代表提出关于制定公共场所禁烟法的议案1件（第308号）。议案提出，我国吸烟人数已超过3亿，每年有100多万人死于烟草相关的疾病，特别是青少年人群吸烟的危害更大。为此，我国多地制定了地方性法规，严格控制室内公共场所吸烟。《烟草控制框架公约》规定，缔约方应积极促进采取有效的立法、行政或其他措施，防止在室内工作场所、公共交通工具、室内公共场所接触烟草烟雾。控烟履约是我国政府对国际社会的庄严承诺，迫切需要制定配套的法律法规，建议制定公共场所禁烟法。

国家卫生健康委认为，我国控烟立法进展一直备受国内外关注。《烟草控制框架公约》于2006年1月9日在我国正式生效。作为公约缔约方，我国高度重视履约工作，2007年国务院成立了“烟草控制框架公约履约工作部际协调领导小组”，经过几次机构改革、职能调整，目前领导小组办公室设在国家卫生健康委，由国家卫生健康委牵头，8个部门共同承担履约职责。自我国开展控烟工作以来，国家卫生健康委持续推动控烟工作法治化进程，北京、上海、天津、广州、深圳等多地出台了控烟的地方性法规。2014年10月原国家卫生计生委形成了《公共场所控制吸烟条例》（草案），原国务院法制办将此条例草案上网公开征求意见。2016年，国家卫生健康委在委托相关单位对地方控烟立法实施效果等情况进行了第三方评估。

教科文卫委已促请有关部门在拟制定的《公共场所控制吸烟条例》中，充分吸纳代表议案所提意见和建议。同时，加大监督工作力度，积极推动公共场所控烟工作。

39．陕西代表团史贵禄等30名代表提出关于制定转基因食品管理法的议案1件（第74号）。议案提出，我国现行转基因食品方面的行政法规、部门规章层级较低，缺少全面系统的规定。建议通过制定专门的转基因食品管理法，明确行政主体职责，规范市场准入，完善转基因食品标签制度，统一转基因食品责任制度等。

农业农村部认为，我国现有转基因相关法律法规体系较为完善，对议案所提出的相关内容均有比较明确的规定，农业转基因生物安全评价严谨科学。2001年，国务院颁布了《农业转基因生物安全管理条例》，随后相关部门制定出台了一系列配套规章文件。国务院建立了农业转基因生物安全管理部际联席会议制度，负责研究和协调农业转基因生物安全管理工作的重大问题。建立了属地管理为主的监管体系，实施严格管理，对于生产应用的农业转基因生物进行前置审批。

市场监管总局表示，高度重视转基因食品相关工作，严格落实食品安全法相关要求，加强转基因食品标签标示等相关工作监管。2018年在全国范围内组织开展了为期一个月的食用植物油专项监督检查，查处标签标示不规范等行为。

教科文卫委经研究，同意农业农村部、市场监管总局的意见，已促请有关部门认真研究代表议案所提意见，进一步加大监督管理力度，抓好相关法规制度的落实。

40．广东代表团陈瑞爱等38名代表提出关于抓紧制定保健食品监督管理法的议案1件（第62号）。议案提出，我国保健食品行业规模大、发展快，同时存在非法经营、虚假或夸大宣传、违法广告、消费欺诈等突出问题。相对行业的快速发展，保健食品监督管理还没有专门立法，标准体系不健全，违法违规行为追究力度不够等，建议制定保健食品监督管理法。

市场监管总局认为，修订后的食品安全法有13项条款对保健食品的入市前许可管理、生产管理、市场监管、广告监管、违法处罚等给予了明确规定，最近又修订发布了食品安全法实施条例，基本做到了有法可依。2016年，颁布实施的《保健食品注册与备案管理办法》，为规范保健食品的注册与备案工作提供了政策依据。市场监管总局表示，要认真贯彻党中央、国务院的统一部署，落实“四个最严”要求，加强保健食品监管工作，在严格生产环节监管的同时，与相关部门密切配合，加强对营销环节乱象的整治。

教科文卫委经研究，同意市场监管总局的意见，已促请有关部门全面落实好食品安全法、食品安全法实施条例、保健食品注册与备案管理办法等相关法规和规章办法，进一步加大监管工作力度，严格生产、营销等各个环节监管，着力解决代表议案所提问题，切实保障人民群众健康。同时，开展相关立法前期研究工作。

41．山东代表团王威东等30名代表提出启动罕见病诊疗及管理立法的议案1件（第260号）。议案提出，我国现有罕见病患者2000多万人，能够得到有效治疗的只有5％，诊疗水平较低，严重影响广大罕见病患者的身体健康和生命安全。我国对罕见病诊治和管理起步较晚，现有法律法规不健全，罕见病的定义、发现、诊疗、药品研发和供给、医疗保障等各方面都缺乏法律规定和保障，建议启动罕见病诊疗及管理立法。

国家卫生健康委认为，党中央、国务院对罕见病问题高度重视，卫生、财政、民政、药监、医保等多部门加强协调配合，制定公布了我国第一批罕见病目录，建立了我国罕见病诊疗协作网，加快罕见病药物研发科技攻关和药品审评审批，提高罕见病预防和诊治水平。下一步，国家卫生健康委将抓紧做好建立罕见病患者登记制度、研究增加罕见病目录病种、开展医务人员培训、进一步提升诊断治疗水平等工作，继续加强与国务院相关部门协作，完善规章制度，加大工作力度，努力提高救治水平，造福广大罕见病患者。

教科文卫委经研究，同意国家卫生健康委的意见，已促请有关部门认真研究代表所提意见和建议，完善相关法律和规章制度，多方面采取有力措施，着力提高罕见病诊治水平，努力维护罕见病患者身体健康。

五、2件议案提出2项监督项目，建议适时列入全国人大常委会监督工作计划

1．天津代表团张伯礼等31名代表提出关于开展中医药法执法检查的议案1件（第237号）。议案提出，中医药法实施一年多来，许多部门和地区出台了配套法规和政策措施，较好地促进了中医药事业的传承创新发展，推动了中医药在健康中国建设中发挥更大作用。同时，由于中医药资源配置不合理，城乡和区域发展不平衡，影响了中医药服务的可及性；在中医药法贯彻落实上地区发展不够平衡，个别地区中医医疗机构建设出现倒退现象；在中医专长人员医师资格考核中，有的地方掌握偏宽，有的地方失之过严，产生了负面影响，相关政策规定有待完善。除此之外，仍存在中西医同级医院发展不平衡、中医医疗服务价值体现不够等现象。建议全国人大常委会组织开展中医药法执法检查，及时掌握中医药法实施过程中出现的新问题、新情况，督促中医药法的贯彻实施及配套的制度落实，进一步完善中医药制度体系。

全国人大常委会十分重视中医药法的宣传贯彻实施，在中医药法立法、听取审议专项工作报告、开展相关专题询问时，都对中医药相关问题给予了高度关注。

教科文卫委经研究认为，中医药法是2016年颁布实施的，在实施一段时间后，对该法的贯彻落实情况和中医药事业发展情况开展检查很有必要，建议全国人大常委会在研究监督工作计划时统筹考虑，适时对该法开展执法检查。

2．山东代表团胡桂花等36名代表提出关于开展药品管理法执法检查的议案1件（第239号）。议案提出，自药品管理法颁布实施以来，中国药品监督管理工作从行政管理体制逐步走上了法制化管理轨道。但药品管理法在贯彻和执行过程中还存在许多问题，药品安全风险依然存在，药品监管力度有待加强。建议开展药品管理法执法检查，以推动法律贯彻实施。

全国人大常委会高度重视药品管理法的实施和修改完善工作，栗战书委员长作出重要指示，要求认真落实“四个最严”要求，研究相关立法问题。药品管理法（修订案）经2019年8月十三届全国人大常委会第十二次会议审议通过，将原修正草案改为修订草案，将药品领域改革成果和行之有效的做法上升为法律，按照药品全过程、全链条管理的要求完善有关规定，对存在的突出问题及时予以规范，修订后的药品管理法于2019年12月1日起施行。2019年11月20日，在教科文卫委召开的疫苗管理法和药品管理法（修订）宣传贯彻座谈会上，王晨副委员长对做好法律的贯彻实施工作提出明确要求。我委将认真贯彻落实会议精神，结合代表议案提出的意见和建议，继续推动药品管理法律制度的贯彻实施，适时向全国人大常委会提出监督工作建议。
