# Supplementary material for: Mapping the evolution of fertility support policies in China: A content and instrumental analysis
Source: PLoS One. 2025 Oct 9;20(10):e0332137. doi: 10.1371/journal.pone.0332137 (PMC12510515; doi:10.1371/journal.pone.0332137)
Supplement: S1 Appendix — (ZIP) [file pone.0332137.s001.zip › S1 Appendix. 226 original policy documents/127-卫生部、教育部关于印发《助理全科医生培训标准(试行)》的通知(FBM-CLI-4-184923).docx]

卫生部、教育部关于印发《助理全科医生培训标准(试行)》的通知

发布部门： 卫生部(已撤销) 教育部 机构沿革

发文字号：卫科教发〔2012〕59号

发布日期：2012.09.10

实施日期：2012.09.10

时效性： 现行有效

效力级别： 部门规范性文件

法规类别： 卫生机构与人员

卫生部、教育部关于印发《助理全科医生培训标准（试行）》的通知

（卫科教发[2012]59号）

各省、自治区、直辖市卫生厅局、教育厅（教委），新疆生产建设兵团卫生局、教育局，卫生部直属有关单位：

为贯彻落实《国务院关于建立全科医生制度的指导意见》（国发〔2011〕23号），做好经济欠发达的农村地区助理全科医生培训工作，提升农村基层卫生人才队伍综合服务能力，我们组织制定了《助理全科医生培训标准（试行）》，现印发给你们（可从卫生部和教育部网站下载），请结合实际贯彻执行。为确保助理全科医生培训顺利实施，现就有关工作提出以下要求：

一、各省级卫生行政部门应当根据本地区经济社会发展的实际情况，研究确定实施本培训标准的经济欠发达农村地区的区域范围。结合本地区全科医生人才队伍建设总体战略与规划、培养需求和培养能力，正确处理全科医生规范化培养与助理全科医生培训的关系，科学编制本省（区、市）助理全科医生年度培训计划和中长期培训规划，并于每年10月底前将下一年度培训计划报卫生部备案。

二、助理全科医生培训工作是现阶段落实全科医生制度，加强农村基层卫生人才队伍建设的重要措施之一，其培训基地、师资建设以及培训期间人员管理参照全科医生规范化培养相关办法执行。各省级卫生行政部门应当主动协调有关部门，落实培训经费、培训期间工资待遇等保障政策，确保培训工作顺利实施。

三、各省级卫生和教育行政部门，应当组织相关培训单位完善招录、培训、管理、考核等环节制度建设，强化培训过程管理，确保培训质量，并及时将工作实施过程中的情况和问题反馈卫生部、教育部。

附件：助理全科医生培训标准（试行）

卫生部

教育部

2012年9月10日

助理全科医生培训标准（试行）

根据《国务院关于建立全科医生制度的指导意见》（国发〔2011〕23号）的要求，为做好经济欠发达的农村地区助理全科医生培训工作，制定本培训标准。

总　则

一、培训对象

临床医学专业三年制专科毕业，拟在或已经在农村基层医疗卫生机构从事全科医疗工作的人员。

二、培训目标

（一）拥护中国共产党领导，拥护社会主义制度，热爱祖国，遵守国家法律法规，贯彻执行党的卫生工作方针；热爱医疗卫生事业，具有强烈的职业责任感和良好的医德修养，愿意在农村基层为人民群众健康服务。

（二）理解生物-心理-社会医学模式，具有全科医学理念，掌握临床医学的基本理论、基本知识和基本技能以及公共卫生的相关知识和技能；熟悉全科医学的诊疗思维模式，能够运用全科医学的基本理论和原则指导医疗卫生实践；具有对农村常见病多发病的基本诊疗能力、预防保健工作能力；具有良好的医患沟通能力，以维护和促进健康为目标，向个人、家庭和农村社区提供以需求为导向的综合性、协调性、连续性的基本医疗和预防保健服务。

三、培训年限和方式

（一）培训年限

助理全科医生培训年限为2年（共104周）。因特殊情况不能按期完成培训任务者，允许申请延长培训年限，但原则上不超过1年。

具体时间安排。临床培训82周，安排在认定的临床培养基地进行；基层实践16周，安排在认定的基层实践基地进行；理论和综合素质课程采取集中与分散相结合的方式进行，其中集中理论授课2周，临床、基层实践和人文等综合素质课程穿插在临床培训、基层实践过程中进行；综合考试考核与结业1周，机动3周（基地可结合本地特点自行安排，如执业助理医师考前强化训练或由学员自选科室学习等）。

（二）培训方式

1.培训须在省级有关部门认定的全科医生培养基地进行，培养基地由临床培养基地（以有条件的二级综合医院为主）和基层实践基地（有条件的乡镇卫生院、社区卫生服务中心和专业公共卫生机构）组成；培训过程中综合素质和职业相关能力培养的课程可由有关高等医学院校承担。

2.培训以提高农村全科医疗服务能力和基本公共卫生服务能力为核心，主要由高等医学院校全科医学理论教师及具有带教资格的临床培养基地医师、基层实践基地医师共同组成的师资团队承担带教任务，培训全程实行指导教师制。

3.培训过程注重临床轮转和基层实践的实际效果。在带教师资的指导下，临床轮转阶段加强常见疾病诊疗思维的培养和诊疗技能的培训；基层实践阶段突出临床各科所学理论课程相关知识和技能的整合与应用，以及国家基本公共卫生服务规范相关内容的实践操作。临床轮转与基层实践穿插进行、有机衔接，使培训对象在培训期间始终着重强化以全科医生岗位需求为导向的临床能力培训和基层实践训练。

4.全科医学基本理论知识及全科医生职业理念和综合素质培养采取集中授课、讲座和见习等形式进行。理论培训的内容除安排一定学时集中授课外，其它内容均分散安排在临床培训与基层实践阶段穿插进行，使全科医学理论与实践紧密融合。

四、培训内容及要求

（一）培训内容

培训内容由三部分组成，即临床培训、基层实践、全科医学基本理论与职业理念和综合素质课程培训。

1.临床培训

临床科室轮转时间为82周。轮转期间，学员在具有带教资格的执业医师指导下参与临床基地中相关临床科室的医疗工作，轮转科室及时间分配详见表1。

临床培训期间，内科轮转时间为34周，其中内科门诊时间不少于5周，临床轮转协助管床数不少于3张；神经内科轮转时间为8周，其中安排神经内科门诊时间不少于2周，临床轮转协助管床数不少于3张；急诊急救10周，训练内容包括院内急救和院前急救两部分；其他临床科室的轮转时间详见表1。

基地可根据实际情况在临床科室轮转中安排地方病的学习。对于少见病种和季节性较强病种，可采用病例分析、讲座等形式进行学习。

轮转期间，每周安排不少于半天的集中学习，可采用单独开设的综合课程、系列讲座、案例讨论、技能模块训练等方式，学习临床诊疗和全科医学相关知识及技能。各培训基地根据大纲要求制定轮转计划。

在临床轮转期间，应及早穿插安排必要的基层见习与实践，引导学员根据农村基层全科医疗岗位需求加强针对性的临床能力训练。

临床培训的具体内容与要求，详见“细则”部分。

表1　临床轮转科室及时间分配表

内 容 科 室 时间分配（周）

临床培训（合计82周） 内科（心血管8周，呼吸6周，消化6周，内分泌代谢6周，泌尿4周，血液2周，风湿免疫2周；其中内科门诊时间不少于5周） 34

神经内科（其中门诊不少于2周） 8

急诊急救（院内急救和院前急救） 10

外科 6

妇产科（计划生育咨询1周，妇科门诊2周，产科门诊1周，产房1周，产科病房1周） 6

儿科（门诊2周、病房2周） 4

中医科 2

传染科 2

皮肤科 2

眼科 2

耳鼻咽喉科 2

精神科 2

康复医学科 2

2.基层实践

基层实践16周。其中全科医疗服务技能培训8周，预防保健与基本公共卫生服务技能培训及专业公共卫生机构实践7周（其中专业公共卫生机构实践4周），社区卫生服务管理技能培训1周。

基层实践的具体形式可采取在基层实践基地带教师资的指导下，从事全科医疗活动、公共卫生实践、健康教育小讲课、案例讨论、教学研讨会、预防保健服务工作实践和社区卫生调查等。在基层实践期间，可针对有关临床问题，加强临床基地相关带教医师对学员的业务技术指导。基层实践的具体内容与要求，详见“细则”部分。

3.理论培训

理论培训共计357学时，其中全科医学基本理论与职业理念和综合素质课程81学时，临床医疗服务相关课程126学时，基层全科医疗与公共卫生服务相关课程105学时，综合系列讲座45学时。

全科医学基本理论与职业理念和综合素质课程采用集中授课形式进行，其他内容穿插在临床培训及基层实践过程中进行。

理论培训课程及其时间安排详见表2，具体内容与要求详见“细则”。

表2 理论课程及其时间安排

类别 课程名称 学时 上课形式 建议上课时间

全科医学基本理论与职业理念和综合素质相关课程（81学时） 1.全科医学理念与基本

理论 27 集中 第1-2周

2.全科医疗中常见行为与心理学问题 18 集中 第1-2周

3.全科医疗中的医学伦理、法律法规问题及医患沟通 18 集中 第1-2周

4.卫生经济学在社区卫生服务中的应用 9 集中 第1-2周

5.文献检索与阅读 9 集中 第1-2周

临床医疗服务相关课程（126学时） 1.临床基本技能训练 36 集中或分散 临床阶段

2.临床综合课程（专业、

人文） 45 集中或分散 临床阶段

3.常见症状的鉴别诊断 27 分散 临床阶段

4.危重症的识别与处理 18 分散 临床阶段

基层全科医疗与公共卫生服务相关课程（105学时） 1.临床预防与健康教育 15 分散 基层实践阶段

2.社区常见慢性病健康

管理 18 分散 基层实践阶段

3.社区特殊人群保健 18 分散 基层实践阶段

4.突发事件卫生应急 18 分散 基层实践阶段

5.社区卫生服务管理 9 分散 基层实践阶段

6.预防接种 9 分散 基层实践阶段

7.重性精神疾病患者管

理 9 分散 基层实践阶段

8.法定传染病报告与处

理 9 分散 基层实践阶段

综合系列讲座（45学时） 1.现场急救与病人转运 12 分散 临床阶段

2.常见病的规范诊治与

管理 9 分散 临床阶段

3.社区康复理论与技术 9 分散 临床阶段

4.基层合理用药 15 分散 基层实践阶段

（二）培训要求

培训过程中要突出全科医生作为居民健康“守门人”的岗位特点，强化全心全意为居民健康服务的思想、良好的医患沟通能力、团队协作精神和医疗卫生法制观念，培养其良好的综合素质和职业精神。

五、考试与考核

培训考核分为培训过程考核和结业考核，以过程考核为重点。

（一）培训过程考核

1.临床培训阶段

临床培训阶段考核包括临床各科出科考核和临床培训阶段综合考核。

（1）出科考核。临床各科轮转结束时，由各临床科室根据培训内容要求进行出科考核，考核内容包括：①政治思想、医学伦理与职业道德；②服务态度与医患沟通；③完成本学科培训细则要求的培训内容及要求情况（轮转科室的时间、规定的病种、病例数、技能操作、病历质量等）；④专业理论知识的考核；⑤临床基本技能和临床思维能力考核。考核形式根据轮转科室时间的长短采取试卷考试、病例分析、临床技能实际操作等形式进行。

（2）阶段综合考核。在出科考核的基础上，对整个临床阶段培训完成情况进行综合考核，重点考核学员完成整体培训的情况，并据此对临床阶段的培训情况进行综合评判。

2.基层实践阶段

基层实践阶段重点对培训对象在全科医疗服务技能培训、预防保健与公共卫生服务技能培训、社区卫生服务管理技能培训等方面实践情况进行考核，考核内容包括业务能力和职业态度等综合表现。在基层实践阶段结束时组织阶段综合考核。可采用综合考卷和实际病人管理案例等多种形式进行

（二）结业考核

学员在2年培训结束时，过程考核合格者，须参加省级卫生行政部门统一组织的结业考核。考核内容包括基本理论、基本技能和综合能力等。

六、培训合格证书颁发

完成全程培训，各项考试、考核成绩合格，并且通过执业助理医师资格考试者，由省级卫生行政部门颁发统一印制的助理全科医生培训合格证书。

细　则

一、临床培训

（一）内科（34周）

1.轮转目的

（1）系统学习内科常见病、多发病的基础理论和基本知识，掌握病史采集、体格检查、病历书写、心电图基本操作与判读、常用临床检验和辅助检查结果的判读；培养正确的临床思维；掌握常见内科疾病的诊断与鉴别诊断、处理原则及转诊指征。

（2）具有正确评估及处理常见内科疾病的能力，识别疾病的不稳定状态及高危状态，并能在上级医生的指导下给予及时、正确的急救与转诊。

（3）具有一定的自我学习和在医疗实践中提高的能力。

2.基本要求

（1）常见症状

掌握以下常见症状的诊断与鉴别诊断、处理原则：发热、头痛、胸痛、心悸、呼吸困难、头晕、晕厥、意识障碍、咳嗽、咯血、黄疸、呕吐、腹痛、腹泻、便秘、呕血与便血、血尿、水肿、贫血、关节痛、淋巴结肿大、消瘦、肥胖。

（2）主要疾病

1）心血管系统：8周

①高血压

掌握：正常血压值，高血压的诊断标准、分级及危险分层；原发性高血压与继发性高血压的鉴别诊断；老年人高血压的临床特点；高血压一、二、三级预防原则；高血压的药物治疗和非药物治疗；高血压的急、慢性并发症的处理原则；高血压患者健康管理服务规范。

了解：高血压药物治疗进展动态。

②冠心病

掌握：冠心病的诊断，心绞痛的临床分型、临床表现、诊断与鉴别诊断，缓解期的治疗，急性期的处理及转诊指征；心肌梗死的诊断依据、心电图特征、鉴别诊断、处理原则与院前急诊处理，转诊指征及注意事项；；冠心病的一、二、三级预防原则及康复措施。

熟悉：心肌梗死恢复期、维持期的康复医疗。

了解：冠心病介入治疗与外科治疗的方法和适应症。

③充血性心力衰竭

掌握：心力衰竭的定义、分型、临床表现、诊断与鉴别诊断、心功能分级、治疗原则；心力衰竭的预防原则及康复；常用药物的适应症、禁忌症。

熟悉：常用药物的作用机制、药物过量的临床表现与处理原则。

了解：病因、诱发因素。

④心律失常

掌握：常见心律失常的临床表现及心电图诊断（窦性心动过速、房性期前收缩、室上性心动过速、心房扑动、心房颤动、室性期前收缩、室性心动过速、心室扑动、心室颤动、窦性心动过缓、房室阻滞）； 低危心律失常患者的处理原则及高危心律失常患者的识别及转诊处理。

熟悉：介入治疗与起搏器治疗后病人的社区照顾与随访；抗心律失常药物的分类、作用特点和临床应用。

了解：心律失常介入治疗的适应症和禁忌症。

⑤相关的诊疗方法

掌握：心电图机操作，正常与异常心电图的判读。

了解：动态心电图，动态血压测定，心电图运动试验，超声心动图的应用范围。

2）呼吸系统：6周

①上呼吸道感染

掌握：上呼吸道感染的临床表现、诊断与鉴别诊断、合理用药的原则。

熟悉：上呼吸道感染常见并发症的预防与处理。

②支气管哮喘

掌握：支气管哮喘的临床表现、诊断与鉴别诊断、治疗原则、缓解期社区防治。

熟悉：重症哮喘的诱因及临床表现、急救原则。

了解：支气管哮喘的病因、发病机理、流行病学特点、峰流速仪和呼吸机的使用。

③慢性支气管炎和慢性阻塞性肺疾病

掌握：慢性支气管炎和慢性阻塞性肺疾病的病因、临床表现、诊断与鉴别诊断；急性发作期及慢性迁延期的治疗，三级预防措施与康复。

熟悉：慢性支气管炎和慢性阻塞性肺疾病的分型、发病机理、流行病学特点及社区预防管理。

④呼吸衰竭

熟悉：呼吸衰竭的临床表现、诊断（肺性脑病的识别与鉴别）及正确及时的转诊；呼吸衰竭的紧急处置原则。

⑤肺炎（重点社区获得性肺炎）

掌握：常见肺炎的病因、临床表现、诊断与鉴别诊断和治疗原则；抗生素的合理使用原则（包括抗生素的停药指征）。

熟悉：肺炎急性并发症的临床表现及处理原则。

⑥相关的诊疗方法

掌握：吸痰术；胸部X线片判读。

熟悉：胸腔穿刺的适应症、禁忌症及操作方法。

了解：常规肺功能测定方法；血气分析结果的判定；痰涂片、抗酸染色、革兰氏染色、痰脱落细胞检查、结核菌素试验的临床应用；胸部CT检查的适应症、禁忌症；胸水检查结果的判读。

3）消化系统：6周

①慢性胃炎

掌握：慢性胃炎的临床表现、诊断与鉴别诊断和常用的治疗方法。

②消化性溃疡

掌握：消化性溃疡临床表现、诊断与鉴别诊断、药物治疗、并发消化道大出血的急诊处理及转诊措施。

熟悉：溃疡病的并发症及处理原则。

了解：消化性溃疡发病机制、幽门螺旋杆菌感染检出与治疗方法、手术治疗指征及胃镜等检查前的准备；各项辅助检查的临床意义。

③急、慢性腹泻

掌握：急、慢性腹泻的常见病因、诊断与鉴别诊断、治疗原则及转诊指征；肠道传染病处置措施及报告程序。

熟悉：感染和非感染性肠道疾病的预防原则。

了解：结肠镜检、钡灌肠的适应症及准备工作。

④肝硬化

掌握：肝硬化病因、临床表现。

熟悉：肝硬化辅助检查的临床意义，治疗要点，常见并发症的识别与转诊指征；肝性脑病的诱因及预防。

⑤胃食管反流病

掌握：胃食管反流病的临床表现、诊断与鉴别诊断、处理原则。

熟悉：胃食管反流病的并发症。

了解：胃食管反流病的发病机制。

⑥胆囊炎

熟悉：胆囊炎的临床表现、诊断与鉴别诊断、处理和转诊原则。

⑦胰腺炎

熟悉：胰腺炎的临床表现、诊断与鉴别诊断、处理和转诊原则。

⑧相关的诊疗方法

掌握：灌肠法的适应症、操作方法及注意事项；留置胃管的操作方法、步骤以及注意事项。

熟悉：电子胃镜、电子结肠镜和消化系统X线检查的适应症、禁忌症和并发症；腹腔穿刺的适应症、禁忌症及操作方法。

4）内分泌及代谢系统：6周

①糖尿病

掌握：糖尿病的分型与诊断标准、治疗原则；低血糖的原因、临床表现及预防；糖尿病常见并发症的临床表现、治疗原则及转诊指征；糖尿病患者的健康管理服务规范。

熟悉：糖尿病酮症酸中毒和高血糖高渗状态的诱发因素；胰岛素的正确应用。

了解：糖尿病流行趋势及发病机理。

②血脂异常和脂蛋白异常血症

掌握：脂蛋白异常血症的分类；非药物与药物治疗方法。

熟悉：血脂异常的各项实验室检查标准。

了解：血脂异常和脂蛋白异常血症药物治疗的不良反应及监测指标。

③甲状腺功能亢进

掌握：甲状腺功能亢进的临床表现、诊断与鉴别诊断；甲状腺危象诱因、临床表现及转诊指征。

熟悉：甲状腺功能亢进的药物治疗（包括药物的副作用，日常监测项目）；甲状腺功能亢进的实验室检查结果判读。

了解：甲状腺功能亢进性心脏病的临床表现、三种治疗方案（药物、放射性碘治疗、手术治疗）的优缺点，甲状腺危象的预防。

④甲状腺功能减退

掌握：甲状腺功能减退的临床表现、诊断与鉴别诊断、转诊指征。

熟悉：甲状腺功能减退的药物治疗、实验室检查；亚临床甲状腺功能减退的诊断、治疗原则。

了解：甲状腺功能减退的病因、发病机制与并发症。

⑤相关的诊疗方法

掌握：糖尿病的实验室检查结果判读及快速血糖检测。

熟悉：甲状腺功能辅助检查结果的判读。

了解：内分泌试验（口服葡萄糖耐量实验、胰岛功能检查等）的测定要求。

5）血液系统：2周

①贫血

掌握：缺铁性贫血和营养不良性贫血的病因、临床表现、诊断与鉴别诊断、治疗原则；预防和筛查方法；贫血的转诊指征。

熟悉：再生障碍性贫血和溶血性贫血病因、临床表现、诊断与鉴别诊断、治疗原则。

②出血性疾病

掌握：过敏性紫癜与血小板减少性紫癜的病因、诊断与鉴别诊断、转诊指征。

③急、慢性白血病

熟悉：急、慢性白血病的临床表现及转诊指征。

了解：急、慢性白血病的治疗原则；实验室检查及其临床意义。

④相关的诊疗方法

熟悉：骨髓穿刺的适应症、禁忌症；输血的适应症及注意事项。

6）泌尿系统：4周

①泌尿系统感染

掌握：急、慢性泌尿系统感染的诱因、临床表现、鉴别诊断、常规治疗及预防措施。

②肾小球肾炎

熟悉：急、慢性肾小球肾炎的临床特点、诊断与鉴别诊断、治疗、转诊指征及预防原则。

了解：肾上腺皮质激素、免疫抑制剂和抗凝剂的应用；继发性肾小球疾病（糖尿病肾病、高血压肾动脉硬化）的诊断与处理原则。

③慢性肾功能不全

熟悉：慢性肾功能不全的临床表现、诊断、治疗原则、转诊指征、预防及早期筛查。

了解：非透析疗法（营养疗法）；腹膜透析、血液透析的适应症；慢性肾功能不全的分期及鉴别诊断。

④相关的诊疗方法：

掌握：导尿术的适应症、操作方法及注意事项。

熟悉：肾功能检查方法、应用和结果判断；尿标本的采集方法。

7）风湿性疾病：2周

①系统性红斑狼疮

熟悉：系统性红斑狼疮的临床表现、诊断与鉴别诊断。

了解：系统性红斑狼疮的诱发因素、活动度的判定、药物治疗及其预后。

②类风湿关节炎

熟悉：类风湿关节炎的临床表现、诊断与鉴别诊断。

了解：类风湿关节炎的实验室和辅助检查、治疗方法。

8）常见地方病

熟悉：当地常见地方病的病因、临床表现、诊断与鉴别诊断、治疗原则及地方病监测和防治措施。

了解：当地常见地方病的实验室和辅助检查、治疗方法。

（3）临床常用注射技能

掌握：肌肉注射、皮内注射、皮下注射和静脉输液操作技术。

内科轮转学习主要疾病和例数要求见表3，基本技能要求见表4。

表3 内科轮转学习主要疾病和例数要求

疾 病 名 称 最低例数

心血管系统：

1.高血压 8

2.冠心病 4

3.充血性心力衰竭 4

4.常见心律失常 8

呼吸系统：

1.上呼吸道感染 6

2.支气管哮喘 2

3.慢性支气管炎和慢性阻塞性肺疾病 4

4.呼吸衰竭 2

5.肺炎 3

消化系统：

1.慢性胃炎 4

2.消化性溃疡 3

3.急、慢性腹泻 2

4.肝硬化 1

5.胃食管反流病 1

6.胆囊炎 1

7.胰腺炎 1

内分泌及代谢系统：

1.糖尿病 10

2.血脂异常和脂蛋白异常血症 6

3.甲状腺功能亢进 4

4.甲状腺功能减退 4

血液系统：

1.贫血 3

2.出血性疾病 1

3.急、慢性白血病 1

泌尿系统：

1.泌尿系统感染 5

2.肾小球肾炎 4

3.慢性肾功能不全 4

风湿性疾病：

1.系统性红斑狼疮 1

2.类风湿性关节炎 2

常见地方病： 根据各地要求

表4 内科轮转基本技能要求

操 作 技 术 名 称 最低例数

掌握：

1.吸痰术 6

2.胸部X线片判读 15

3.心电图机操作，正常与异常心电图的判读 15

4.灌肠法的适应症、操作方法及注意事项 1

5.留置胃管的操作方法、步骤以及注意事项 1

6.糖尿病的实验室检查结果判读及快速血糖检测 5

7.导尿术的适应症、操作方法及注意事项 2

8.肌肉注射、皮内注射、皮下注射和静脉输液操作技术 各5

熟悉：

1.胸腔、腹腔及骨髓穿刺的适应症、禁忌症及操作方法

2.电子胃镜、电子结肠镜和消化系统X线检查的适应症、禁忌症和并发症

3.甲状腺功能辅助检查结果的判读

4.输血的适应症及注意事项

5.肾功能检查方法、应用范围和结果判断

6.尿标本的采集方法

了解：

1.动态心电图，动态血压测定，心电图运动试验，超声心动图、颈动脉超声的应用范围

2.肺功能测定方法

3.血气分析结果的判定

4.痰涂片、抗酸染色、革兰氏染色、痰脱落细胞检查、结核菌素试验的临床应用范围

5.胸部CT检查的适应症、禁忌症

6.胸水检查结果判读

7.内分泌试验（口服葡萄糖耐量实验、胰岛功能检查等）的测定要求

注：各科轮转要求的病例数和基本技能操作的例数为最低要求；对于有创性技能操作，可在模型上进行；未明确要求例数的，各地根据实际情况酌情安排。

（二）神经内科（8周）

1.轮转目的

系统学习神经内科常见疾病的基础理论和基本知识，掌握病史采集、体格检查、病历书写等临床技能；培养正确的临床思维；掌握神经内科常见疾病的诊断和处理；了解神经内科常见疾病的CT、MRI阅片。

2.基本要求

（1）常见症状

掌握以下常见症状的诊断与鉴别诊断、处理原则：意识障碍、认知障碍、构音障碍、面肌瘫痪、眩晕、头痛、晕厥、癫痫发作、感觉障碍、瘫痪、肌肉萎缩、步态异常、共济失调、尿便障碍。

（2）主要疾病

1）短暂性脑缺血发作

掌握：短暂性脑缺血发作的定义、临床表现、诊断与鉴别诊断、治疗原则、院前急诊处理及转诊指征。

了解：短暂性脑缺血发作的病因及发病机制；治疗新进展。

2）动脉粥样硬化性脑血栓（脑梗死）

掌握：动脉粥样硬化性脑血栓的定义、临床表现、诊断与鉴别诊断、院前急诊处理及转诊指征；缓解期的治疗；针对可干预的危险因素的二级预防原则；康复指征。

熟悉：动脉粥样硬化性脑血栓的急性期的处理原则。

了解：动脉粥样硬化性脑血栓的病因、发病机制。

3）脑栓塞

掌握：脑栓塞的定义、临床表现、诊断与鉴别诊断、院前急诊处理及转诊指征；缓解期的治疗；针对可干预的危险因素的二级预防原则；康复指征。

熟悉：脑栓塞的病因及发病机制。

4）脑出血

掌握：脑出血的定义、临床表现、诊断与鉴别诊断、院前急诊处理及转诊指征；急性期不同的处理原则；康复指征。

熟悉：脑出血的病因。

了解：脑出血的发病机制、病理。

5）蛛网膜下腔出血

掌握：蛛网膜下腔出血的定义、临床表现、诊断与鉴别诊断、院前急诊处理及转诊指征、治疗原则；康复指征。

熟悉：蛛网膜下腔出血急性期的处理。

了解：蛛网膜下腔出血的病因、发病机制、病理。

6）其他疾病，如痴呆症、帕金森病、面神经麻痹、脑膜炎等。

熟悉：痴呆症、帕金森病、面神经麻痹、脑膜炎等疾病的诊断要点及治疗方法。

了解：痴呆症、帕金森病、面神经麻痹、脑膜炎等疾病的病因及发病机制。

（3）基本技能

掌握：神经系统的体格检查技术。

熟悉：腰椎穿刺的适应症与注意事项；卧床病人的护理。

了解：头颅CT、MRI阅片。

神经内科轮转学习主要疾病和例数要求见表5，基本技能要求见表6。

表5 神经内科轮转学习主要疾病和例数要求

疾 病 名 称 最低例数

1.短暂性脑缺血发作 3

2.动脉粥样硬化性脑血栓（脑梗死） 3

3.脑栓塞 2

4.脑出血 2

5.蛛网膜下腔出血 1

6.其他疾病（痴呆症、帕金森病、面神经麻痹、脑膜炎） 各1

表6 神经内科轮转基本技能要求

操 作 技 术 名 称 最低例数

掌握：

神经系统体格检查 5

熟悉：

腰椎穿刺

卧床病人的护理

了解：

头颅CT阅片

头颅MRI阅片

（三）急诊急救（10周）

急诊急救培训包括院内急救和院前急救两部分内容。

1.轮转目的

（1）在上级医师指导下诊治急诊病人，了解急诊急救的诊治流程，学习常见急症的诊断方法与抢救原则，掌握心肺复苏术、电除颤、洗胃、包扎、患者搬运等急救技能；培养正确的临床思维和医患沟通技巧；

（2）具有正确评估及处理农村地区常见急症的能力，对危重症能正确评估其潜在风险，并给予正确的急救与处理。

2.基本要求

（1）基本知识

掌握：急症的诊断和抢救原则；常见急症的识别与初步处理原则、转诊指征；院前急救流程及急救技术；常用急救药物的用药原则和注意事项。

熟悉：高级心肺复苏的步骤、条件以及心肺复苏的终止指标；急症缓解后处理原则。

了解：现代急诊医学主要内容；现代急诊医疗体系基本组织形式；灾难抢救、重大交通事故、地震、水灾、火灾等重大抢救处理及防疫原则。

（2）院内急救

1）心脏骤停

掌握：心脏骤停的快速诊断、复苏流程。

熟悉：心脏骤停的高级心肺脑复苏术。

了解：心脏骤停的定义、病因。

2）急性左心衰竭

掌握：急性左心衰竭诊断和急救处理。

熟悉：急性左心衰竭的常见原因。

3）自发性气胸

掌握：自发性气胸的临床表现与体征及影像学特点。

熟悉：自发性气胸的病因及处理方法。

了解：自发性气胸的发病机制。

4）重症哮喘

掌握：重症哮喘的临床表现、诊断与抢救处理原则。

5）糖尿病酮症酸中毒

掌握：糖尿病酮症酸中毒的诊断标准和急救原则。

熟悉：糖尿病酮症酸中毒的鉴别诊断。

6）心绞痛、急性心肌梗死

掌握：心绞痛、急性心肌梗死的临床表现、心电图特点、诊断与鉴别诊断、急救原则。

熟悉：急性心肌梗死溶栓和急诊介入的指征。

了解：冠心病的介入治疗与外科治疗的方法和适应症。

7）休克

熟悉：休克的基本分类和抢救原则。

8）上消化道出血

掌握：上消化道出血的诊断、救治及转送方式。

熟悉：上消化道出血的常见病因、失血量估计、是否继续出血的判断方法。

了解：上消化道出血内镜治疗指征与外科手术指征。

9）阵发性室上性心动过速

掌握：阵发性室上性心动过速的识别和急诊处理。

10）癫痫持续状态

掌握：癫痫持续状态的临床表现、诊断标准和急救处理。

熟悉：癫痫持续状态的搬运方法。

了解：癫痫持续状态的病因。

11）中毒与意外伤害

掌握：常见中毒诊断与鉴别诊断、急救原则；常见意外伤害的紧急救治原则及其转运注意事项。

了解：毒物在体内的代谢过程及中毒机制；农村常见意外伤害的原因。

12）急腹症

掌握：急腹症的临床表现、诊断与鉴别诊断。

熟悉：急腹症的处理原则及转诊指征。

13）脑出血、脑血栓

掌握：脑出血、脑血栓形成的诊断和急诊处理。

了解：脑出血的外科处理，脑血栓的溶栓治疗。

14）创伤

掌握：颅脑外伤诊治程序；气胸、肺挫伤、肋骨骨折的诊断。

熟悉：多发伤的诊治流程；骨折伤情判断程序。

15）犬咬伤（狂犬病暴露）

掌握：狂犬病暴露后的伤口处理；疫苗注射时间、流程及注意事项。

熟悉：狂犬病暴露后处置规范。

（3）院前急救：

1）创伤

掌握：多发创伤现场急救及转送原则；颅脑外伤的现场急救原则及转送指征；气胸、肺挫伤、肋骨骨折的诊断、现场急救及转送指征；骨折急救方法以及颈椎外伤、脊椎外伤、合并截瘫、四肢骨折病人的搬运方法；手外伤伤口紧急处理方法及断指保存方法；烧伤现场急救原则及转院指征。

熟悉：多发性创伤诊断程序；骨折伤情判断程序。

了解：胸部外伤和骨折分类。

2）中毒和意外伤害

掌握：常见中毒的诊断、鉴别诊断、急救及转送原则； 中暑和动物咬伤的紧急处理方法； 淹溺的紧急处理方法。

熟悉：动物咬伤的临床表现及处理原则。

了解：毒物在体内的代谢过程及中毒机制；中暑的病理生理及临床分类；动物咬伤主要毒理作用机制及预后。

3）心脏骤停

掌握：心脏骤停的现场识别和复苏流程。

熟悉：心肺复苏终止指征。

4）阵发性室上性心动过速

掌握：阵发性室上性心动过速的识别和非药物治疗的方法。

5）昏迷

掌握：昏迷患者的现场救治和转运原则。

熟悉：昏迷的常见病因。

6）急性心肌梗死

掌握：急性心肌梗死现场救治原则。

熟悉：急性心肌梗死转运监护和处理。

7）脑血管病

掌握：脑血管病现场识别、处理及转运监护原则。

8）低血糖症

掌握：低血糖症的现场识别、处理及转院指征。

熟悉：低血糖症的病因。

（4）基本技能

掌握：院内单、双人心肺复苏技术、电除颤术；洗胃术操作方法及准备工作；创伤的包扎止血固定；骨折石膏固定后的护理技术和注意事项。

了解：气管插管术的适应症和注意事项。

急诊急救轮转学习主要疾病和例数要求见表7，基本技能要求见表8。

表7 急诊急救学习主要疾病和例数要求

疾 病 名 称 最低例数

1.心脏骤停 2

2.急性左心衰竭 2

3.自发性气胸 1

4.重症哮喘 1

5.糖尿病酮症酸中毒 4

6.心绞痛、急性心肌梗死 各2

7.休克 2

8.上消化道出血 2

9.阵发性室上性心动过速 2

10.癫痫持续状态 1

11.中毒与意外伤害 3

12.急腹症 3

13.脑出血、脑血栓 各4

14.创伤 5

15.犬咬伤（狂犬病暴露） 1

16.昏迷 2

17.脑血管病 2

18.低血糖症 2

表8 急诊急救的基本技能要求

操 作 技 术 名 称 最低例数

掌握：

1.院内单、双人心肺复苏技术、电除颤术

3

2.洗胃术操作方法及准备工作 3

3.创伤的包扎止血固定 3

4.骨折石膏固定后的护理技术和注意事项 2

了解：

气管插管术

（四）外科 （6周）

1.轮转目的

（1）学习外科常见疾病的基础理论和基本知识，掌握门诊病人的病史采集、体格检查等临床技能及体表清创缝合、引流、换药、拆线等必要的诊疗技术；培养正确的临床思维；掌握农村地区外科常见疾病的诊断和处理。

（2）具有正确评估及处理外科常见疾病的能力，判断是否需要急诊手术，并能给予正确的急救与处理。

2.基本要求

（1）常见症状

掌握：以下常见症状的诊断与鉴别诊断、处理原则：体表肿物、腹痛腹胀、恶心呕吐、排尿困难、腰腿痛和颈肩痛。

（2）主要疾病

掌握以下外科常见疾病的诊断与鉴别诊断，熟悉转院标准及急诊手术指征，了解治疗方案。

1）外科感染（软组织感染、破伤风）

掌握：软组织感染与破伤风的临床表现及治疗方法，抗生素的合理使用。

熟悉：破伤风疫苗的使用原则和注意事项。

2）体表肿瘤（脂肪瘤、皮脂腺囊肿）

掌握：脂肪瘤、皮脂腺囊肿的诊断标准。

3）腹部疾病

①腹股沟疝

掌握：腹股沟疝的临床特点及诊断。

熟悉：腹股沟疝的治疗原则及转诊指征。

②阑尾炎

掌握：急、慢性阑尾炎的临床表现及诊断。

熟悉：急、慢性阑尾炎的治疗原则及转诊指征。

了解：特殊类型阑尾炎的临床特点；阑尾炎手术治疗的方法和并发症。

③肠梗阻

熟悉：单纯性与绞窄性肠梗阻的临床特点、治疗原则。

了解：急性肠梗阻的病因、临床分型和治疗原则。

4）外周血管疾病（大隐静脉曲张）

熟悉：大隐静脉曲张的诊断与鉴别诊断。

了解：大隐静脉曲张的治疗方案。

5）乳腺疾病（急性乳腺炎、乳腺增生）

掌握：乳房检查方法。

熟悉：急性乳腺炎与乳腺增生的诊断与鉴别诊断。

了解：急性乳腺炎与乳腺增生的进一步检查方法及治疗方案。

6）肛门直肠疾病（痔）

掌握：肛门直肠疾病的常用诊断方法（肛门指诊方法）。

了解：痔的临床表现与治疗方案。

7）泌尿系结石与前列腺增生症

熟悉：泌尿系结石与前列腺增生症的病因、诊断、治疗原则及转诊指征。

8）腰腿痛和颈肩痛

掌握：腰腿痛和颈肩痛的临床特点、诊断和治疗、康复原则；疼痛封闭治疗的适应症、方法和注意事项。

了解：腰腿痛和颈肩痛的病因及发病机制；各关节穿刺部位和方法。

9）常见恶性肿瘤（肺癌、肝癌、胃癌、乳腺癌、结直肠癌等）

熟悉：临床表现、诊断与鉴别诊断。

了解：治疗原则。

（3）其他相关知识与基本技能

掌握：外科常用的消毒剂、消毒方法及注意事项；外科疾病的查体和物理诊断方法；无菌操作原则（手术野准备）；外伤的清创缝合技术；换药及拆线操作技术；疼痛封闭治疗操作。

熟悉：感染伤口清创原则与方法；体表肿物切除术；不同手术换药及拆线的时间；伤口和造瘘口护理。

了解：灭菌的常用方法及灭菌后物品的使用期限。

外科轮转学习主要疾病和例数要求见表9，基本技能要求见表10。

表9 外科轮转学习主要疾病和例数要求

疾 病 名 称 最低例数

1.外科感染（软组织感染、破伤风） 各2

2.体表肿瘤（脂肪瘤、皮脂腺囊肿） 各1

3.腹部疾病

（1）腹股沟疝 2

（2）阑尾炎 2

（3）肠梗阻 1

4.外周血管疾病（大隐静脉曲张） 1

5.乳腺疾病（急性乳腺炎、乳腺增生） 各2

6.肛门直肠疾病（痔） 2

7.泌尿系结石与前列腺增生症 各2

8.腰腿痛和颈肩痛 各2

9.常见肿瘤（肺癌、肝癌、胃癌、乳腺癌、结肠癌等） 各1

表10 外科轮转基本技能要求

操 作 技 术 名 称 最低例数

掌握：

1.外科疾病的查体和物理诊断方法 5

2.无菌操作（手术野准备） 2

3.外伤的清创缝合 2

4.伤口的换药及拆线 2

5.肛门指诊 1

6.疼痛封闭治疗 3

熟悉：

1.感染伤口清创原则与方法

2.体表肿物切除术

3.术后换药及拆线

4.伤口和造瘘口护理

了解：

灭菌的常用方法及灭菌后物品的使用期限

（五）妇产科（6周）

1.轮转目的

掌握妇产科常见疾病的基础理论和基本知识；掌握门诊常见妇科疾病的处理流程；掌握围生期保健的主要内容和相应的处理原则；掌握计划生育指导、常用计划生育手术的适应症；掌握妇产科常见急危重症的诊断和评估、转诊指征及其转诊注意事项。

2.基本要求

（1）常见症状：掌握以下常见症状的诊断与鉴别诊断、处理原则：白带异常、阴道异常出血、急性腹痛、慢性腹痛、盆腔肿物、腹胀。

（2）主要疾病

1）宫颈和阴道炎症

熟悉：常见宫颈和阴道炎症的临床表现、诊断与鉴别诊断、治疗。

2）阴道异常出血

了解：导致阴道异常出血的常见疾病及其特征。

3）子宫肌瘤、卵巢囊肿

熟悉：子宫及卵巢良性肿瘤的临床表现、体征、处理原则、转诊指征。

4）妇科急腹症

熟悉：异位妊娠、急性盆腔炎、卵巢囊肿蒂扭转的临床表现、常用辅助检查方法、适时转诊的指征。

（3）基本技能

1）围生期保健

掌握：妊娠早、中、晚期诊断方法；孕期保健的检查内容和意义；临产表现及护理；产妇及新生儿的护理；产后保健内容等。

熟悉：接生技术；高危妊娠的识别、诊断、转诊指征；新生儿窒息复苏的基本技术，异常产褥的诊断、处理原则、转诊指征。

了解：产后出血的急救处理。

2）围绝经期保健

掌握：围绝经期综合征的临床表现及诊断；围绝经期骨质疏松症的预防与治疗。

熟悉：其它围绝经期常见的健康问题及预防。

3）计划生育

掌握：各种避孕方法的适应症和禁忌症，取放宫内节育器等计划生育手术适应症和禁忌症、高危手术的识别及转诊指征。

熟悉：计划生育手术并发症的识别、处理及转诊指征；优生优育的咨询与指导。

4）其他相关技能

掌握：妇科双合诊检查技术；窥阴器的使用方法；尿妊娠试验（人绒毛膜促性腺激素测定试验）试纸的使用方法、血清绒毛膜促性腺激素β亚基测定的指征；宫颈细胞学筛查的方法、结果判断和宫颈涂片操作技术；阴道分泌物悬滴检查相关原则和技术。

熟悉：孕期四步触诊检查法；骨盆外测量方法。

了解：刮宫术操作原则及过程；宫内节育器置入术及取出术的操作原则及过程；妇科B超检查的临床应用。

妇产科轮转学习主要疾病和例数要求见表11，基本技能要求见表12。

表11　妇产科轮转学习主要疾病和例数要求

疾 病 名 称 最低例数

1.宫颈和阴道炎症 10

2.阴道异常出血 5

3.妇科肿瘤（子宫肌瘤、卵巢囊肿） 各3

4.妇科急腹症 2

表12 妇产科轮转基本技能要求

操 作 技 术 名 称 最低例数

掌握：

1.围生期保健

2.围绝经期保健

3.计划生育 10

3

1

4.妇科双合诊检查技术 10

5.窥阴器的使用方法 10

6.宫颈涂片技术 5

7.阴道分泌物悬滴检查 5

熟悉：

1.孕期四步触诊检查法

2.骨盆外测量

3.助产过程（助产人员协助）

了解：

1.刮宫术

2.宫内节育器置入术

3.宫内节育器取出术

4.妇科B超检查的临床应用

（六）儿科（4周）

1.轮转目的

掌握儿科常见疾病的基础理论和基本知识；掌握儿科病史采集和体格检查的特殊性；小儿用药特点、药物剂量的计算方法；小儿生长发育指标的正常值和测量方法；儿科常见疾病的诊断和处理原则。了解儿童生长发育规律和影响因素。

2.基本要求

（1）主要疾病

1）新生儿肺炎

掌握：新生儿肺炎的预防措施及转诊原则。

熟悉：新生儿肺炎的临床表现及诊断。

了解：新生儿肺炎的病因及治疗原则。

2）新生儿黄疸

掌握：新生儿黄疸的分类、诊断与鉴别诊断、转诊原则。

熟悉：新生儿生理性黄疸的发展过程；高胆红素血症的临床表现、危害性及防治方法。

了解：新生儿时期胆红素代谢的特点；胆红素脑病的临床表现及防治方法。

3）小儿贫血

掌握：小儿贫血的诊断标准、鉴别诊断、治疗及预防原则。

熟悉：营养性缺铁性贫血病因、发病机制及临床表现。

了解：小儿铁代谢、叶酸和维生素B12代谢的特点。

4）佝偻病及婴儿手足搐搦症

掌握：佝偻病及婴儿手足搐搦症的临床表现及各期的诊断、治疗及预防原则。

熟悉：佝偻病及婴儿手足搐搦症的病因及发病原理。

了解：维生素D的主要生理功能，维生素D过量或中毒的临床表现及防治措施。

5）常见呼吸道疾病（包括上呼吸道感染、支气管炎、肺炎、喉炎）

掌握：常见呼吸道疾病的诊断、处理原则及转诊指征。

熟悉：常见呼吸道疾病的临床特点。

6）小儿腹泻

掌握：小儿腹泻的临床表现、诊断要点、治疗（包括液体治疗）原则及转诊指征。

熟悉：小儿腹泻的病因及鉴别诊断。

7）小儿腹痛

熟悉：小儿腹痛的病因、检查方法、诊断与鉴别诊断、内外科处理原则及转诊指征。

8）小儿惊厥

掌握：高热惊厥的临床表现、诊断与鉴别诊断、急救措施和预防原则。

熟悉：高热惊厥的病因、发病机制；癫痫持续状态的定义、危害性及治疗原则。

9）先天性心脏病

熟悉：小儿各年龄段心界、心率和血压的正常值；先天性心脏病的临床分类及特点。

了解：小儿循环系统解剖生理特点；房间隔缺损、室间隔缺损、动脉导管未闭；法洛四联症的临床表现、诊断要点及转诊原则。

10）小儿常见急性传染病（手足口病、麻疹、水痘、流行性腮腺炎、猩红热、脊髓灰质炎等）

掌握：小儿常见急性传染病的临床表现、诊断要点、转诊指征。

熟悉：小儿常见急性传染病的病因、流行病学特点、鉴别诊断、防治原则及常见并发症。

（2）其他相关知识与基本技能

掌握：儿童体格检查及其各项测量值的正常范围；小儿生长发育与评估；婴儿配奶方法。

熟悉：小儿神经、精神发育的规律；小儿用药特点及药物剂量的计算方法。

了解：小儿生长发育的规律、临床意义及影响生长发育的因素。

儿科轮转学习主要疾病及例数要求见表13，基本技能要求见表14。

表13 儿科轮转学习主要疾病及例数要求

疾 病 名 称 最低例数

1.新生儿肺炎 1

2.新生儿黄疸 1

3.小儿贫血 1

4.佝偻病及婴儿手足搐搦症 1

5.小儿常见呼吸道疾病（包括上呼吸道感染、支气管炎、肺炎、喉炎） 各5

6.小儿腹泻 1

7.小儿腹痛 2

8.小儿惊厥 2

9.先天性心脏病 1

10.小儿常见急性传染病（手足口病、麻疹、水痘、流行性腮腺炎、猩红热、脊髓灰质炎等） 各1

表14 儿科轮转学习基本技能要求

操 作 技 术 名 称 最低例数

掌握：

1.小儿生长发育与评估 3

2.儿童体格检查及其各项测量值的正常范围 3

3. 婴儿配奶方法 3

熟悉：

小儿用药特点及药物剂量的计算方法

（七）中医科（2周）

1.轮转目的

熟悉和了解中成药的使用和部分中医实用技能。

2.基本要求

（1）中成药的使用

熟悉：常用中成药的适应症、使用注意事项、常见副作用及处理原则。

了解：临床常见疾病的辨证施治方法及食疗常识；孕产妇常见疾病的用药禁忌；流感、秋季腹泻等常见传染病的中医预防。

（2）实用技能

1）耳穴疗法之压豆法

熟悉：耳穴压豆法的原理、耳穴分布图、配方取穴法、适应禁忌症、物品准备、操作方法、护理及注意事项。

2）刮痧

熟悉：刮痧施治原则、适应症和禁忌症、操作方法及注意事项。

了解：常用刮痧板材料、刮痧润滑剂型。

3）拔罐

熟悉：拔罐施治原则、适应症和禁忌症、临床操作的基本方法及注意事项。

4）针灸

熟悉：针灸适应症、注意事项。

5）按摩

熟悉：推拿常用腧穴；常见病的推拿手法及常用保健推拿法。

（八）眼科（2周）

1.轮转目的

掌握眼科常见疾病的基础理论和基本知识；掌握病史采集、体格检查等临床技能；掌握眼科常见疾病的诊断与鉴别诊断、处理原则；了解常用的诊疗技术。

2.基本要求

（1）常见症状

掌握以下常见症状的诊断与鉴别诊断、处理原则：视力障碍、感觉异常（眼红、眼痛、畏光、流泪、眼睑痉挛）、外观异常、视疲劳等。

（2）主要疾病

1）睑腺炎（麦粒肿）

掌握：睑腺炎（麦粒肿）的临床表现、诊断与鉴别诊断、治疗原则。

熟悉：睑腺炎（麦粒肿）的局部治疗方法。

2）睑板腺囊肿（霰粒肿）

掌握：睑板腺囊肿（霰粒肿）的临床表现、诊断与鉴别诊断、治疗原则。

熟悉：睑板腺囊肿（霰粒肿）的局部治疗方法。

3）结膜炎

掌握：结膜炎的分类、临床表现、诊断与鉴别诊断、治疗原则。

熟悉：结膜炎的病因、治疗用药。

4）白内障

掌握：白内障的临床表现、诊断与鉴别诊断、治疗原则。

熟悉：老年性白内障的分型、分期。

5）青光眼

熟悉：青光眼的分型、临床表现、诊断与鉴别诊断、治疗原则。

6）眼外伤

掌握：不同原因造成眼外伤的临床特点、诊断与鉴别诊断、治疗原则；酸碱化学伤的急救冲洗技术及其注意事项。

（3）基本技能

掌握：外眼一般检查技术；眼底镜的使用及正常眼底的识别；眼冲洗治疗。

了解：结膜囊异物处理方法；眼压测定；视力检测。

眼科轮转学习病种及例数要求见表15，操作基本技能要求见表16。

表15　眼科轮转学习病种及例数要求

疾 病 名 称 最低例数

1.睑腺炎（麦粒肿） 3

2.睑板腺囊肿（霰粒肿） 3

3.结膜炎 5

4.白内障 5

5.青光眼 3

6.眼外伤 1

表16 眼科轮转学习操作基本技能要求

操 作 技 术 名 称 最低例数

掌握：

1. 外眼一般检查 5

2. 眼底镜的使用及正常眼底的识别 10

3. 眼冲洗治疗 3

了解：

1.结膜囊异物处理方法

2.眼压测定

3.视力检测

（九）耳鼻咽喉科（2周）

1.轮转目的

学习耳鼻喉科常见疾病的基础理论和基本知识，掌握病史采集、体格检查等临床技能；掌握耳鼻喉科常见疾病的诊断和处理原则；了解耳鼻喉科常用的诊疗技术；培养正确的临床思维。

2.基本要求

（1）主要疾病

1）耳外伤、鼻外伤

掌握：耳外伤、鼻外伤的处理原则。

熟悉：耳外伤、鼻外伤的重症病人的临床特征、转诊指征和转诊注意事项。

2）鼻出血

掌握：鼻出血的紧急处理原则。

熟悉：鼻出血的常见病因、进一步处理方法。

3）鼻炎、鼻窦炎

掌握：鼻炎、鼻窦炎的临床表现与治疗原则。

4）急慢性扁桃体炎

掌握：急慢性扁桃体炎及并发症的诊断、治疗原则。

熟悉：急慢性扁桃体炎的局部治疗方法。

5）急性喉炎、会厌炎

掌握：急性喉炎、会厌炎的临床表现、治疗原则；重症患者转院前处理及途中的处理。

6）突发性耳聋

了解：突发性耳聋的临床表现与治疗原则。

7）中耳炎

熟悉：中耳炎的常见类型、临床表现及治疗原则。

（2）基本技能

掌握：耳鼻咽喉一般检查；耳镜使用；鼻咽镜及间接喉镜使用；外耳道异物（耵聍）取除。

了解：听力检测；上颌窦穿刺术。

耳鼻喉科轮转学习病种及例数要求见表17，操作基本技能要求见表18。

表17 耳鼻喉科轮转学习病种及例数要求

疾 病 名 称 最低例数

1.耳外伤、鼻外伤 1

2.鼻出血 2

3.鼻炎、鼻窦炎 6

4.急、慢性扁桃体炎 4

5.急性喉炎、会厌炎 1

6.突发性耳聋 1

7.中耳炎 4

表18 耳鼻喉科轮转学习操作基本技能要求

操 作 技 术 名 称 最低例数

掌握：

1.耳鼻咽喉一般检查 5

2.耳镜使用 4

3.鼻咽镜及间接喉镜使用 3

4.外耳道异物（耵聍）取除 3

了解：

1.听力检测

2.上颌窦穿刺术

（十）皮肤科（2周）

1.轮转目的

学习皮肤科常见疾病的基础理论和基本知识，掌握病史采集、体格检查等临床技能；掌握皮肤科常见疾病的诊断和处理原则；了解皮肤科常用的诊疗技术。

2.基本要求

（1）常见症状

掌握以下常见症状的诊断与鉴别诊断及处理原则：斑疹、丘疹、风团、水疱、脓疱、浸渍、糜烂、溃疡。

（2）主要疾病

1）湿疹

掌握：湿疹的临床表现、诊断与鉴别诊断、治疗原则。

了解：湿疹的病因与发病机制。

2）接触性皮炎

熟悉：一般接触性皮炎的临床表现、诊断与鉴别诊断及治疗原则；重症接触性皮炎的处理原则。

了解：接触性皮炎的病因与发病机制。

3）药疹（药物性皮炎）

掌握：一般药疹的临床表现、诊断与鉴别诊断及治疗原则、预防方法。

熟悉：重症药疹的临床表现及处理原则。

了解：药疹的病因和发病机制。

4）荨麻疹

掌握：一般荨麻疹的临床表现、诊断、治疗及重症荨麻疹的急救处理原则。

了解：荨麻疹的病因及发病机制。

5）银屑病

熟悉：寻常性银屑病的临床表现、诊断与治疗原则。

了解：银屑病的病因及诱发因素；特殊类型银屑病表现。

6）皮肤真菌感染

熟悉：手足癣和体股癣的临床表现、诊断与鉴别诊断及治疗原则。

了解：甲癣、头癣和花斑癣的临床表现、诊断与鉴别诊断及治疗原则。

7）单纯疱疹和带状疱疹

掌握：单纯疱疹和带状疱疹的临床表现、诊断与鉴别诊断及治疗原则。

8）疣

了解：各种疣（寻常疣、扁平疣、传染性软疣）的临床表现及治疗原则。

9）痤疮

了解：寻常性痤疮的临床表现、诊断与鉴别诊断及治疗原则。

10）疥疮和阴虱

熟悉：疥疮和阴虱病的临床表现、治疗原则及预防。

了解：疥疮和阴虱病的致病因素、传染途径。

11）性传播疾病

熟悉：梅毒、淋病的病因及传播途径；后天性梅毒的分期、各期的临床表现及胎传梅毒的临床表现、梅毒血清学检查的临床意义及梅毒的诊断与鉴别诊断及治疗；淋病的临床表现、诊断与鉴别诊断及治疗原则。

了解：性传播性疾病概念；非淋菌性尿道炎、生殖器疱疹及尖锐湿疣的临床表现、诊断及治疗原则。

（3）其他相关知识及基本技能

掌握：常见皮肤病和性病的临床症状；常用外用药的性能、剂型及外用药治疗原则。

熟悉：常见皮肤病和性病的预防原则、常用内服药物的使用；皮炎、湿疹与真菌感染性皮肤病的鉴别诊断。

了解：正常皮肤的组织构造及生理功能；常见皮肤性病的冷冻、激光治疗适应症。

皮肤科轮转学习病种和例数要求见表19。

表19　皮肤科轮转学习病种和例数要求

疾 病 名 称 最低例数

1.湿疹 5

2.接触性皮炎 2

3.药疹（药物性皮炎） 1

4.荨麻疹 5

5.银屑病 2

6.皮肤真菌感染、癣 5

7.单纯疱疹和带状疱疹 3

8.疣 2

9.痤疮 1

10.疥疮和阴虱病 1

11.性传播疾病 1

（十一）康复科（2周）

1.轮转目的

掌握社区康复的基本理论和基础知识，农村地区常见疾病的康复原则；熟悉常用的康复技术。

2.基本要求

掌握：脑血管疾病及颅脑损伤所致功能障碍康复的最佳时间、康复适应症及禁忌症、康复注意事项及转诊指征。

熟悉：常见疾病及损伤的康复评定及原则，包括脑卒中、周围神经损伤、小儿脑瘫、骨关节疾病、骨折等。

了解：运动疗法、作业治疗、言语治疗、常用物理因子治疗。

（十二）传染科（2周）

1.轮转目的

学习传染科常见疾病基础理论和基本知识，掌握病史采集、体格检查、病历书写等临床技能；对常见传染病能够做出诊断及给予相应治疗和转诊；掌握法定传染病报告制度和报告程序。

2．基本要求

（1）主要疾病

1）细菌性痢疾及其他感染性腹泻

掌握：细菌性痢疾及其他感染性腹泻的诊断与鉴别诊断、治疗原则及方法、转诊指征、预防措施及报告程序。

熟悉：细菌性痢疾及其他感染性腹泻的病因。

了解：细菌性痢疾及其他感染性腹泻的流行病学特征。

2）病毒性肝炎

熟悉：病毒性肝炎的临床表现、诊断与鉴别诊断、转诊原则；肝功能及各种实验室检查指标及其临床意义；传播途径及报告程序。

了解：病毒性肝炎的病原学基础。

3）结核病

掌握：结核病的临床表现、诊断与鉴别诊断、治疗、预防原则、报告及转诊程序。

熟悉：结核病的国内流行趋势；常用药物的副作用。

4）获得性免疫缺陷综合症

掌握：获得性免疫缺陷综合症的临床表现。

熟悉：获得性免疫缺陷综合症的诊断与鉴别诊断、治疗原则、预防措施、报告及转诊程序。

了解：获得性免疫缺陷综合症的病因、流行病学特征和发病机制。

5）其他传染病和寄生虫病：如霍乱、流行性脑脊髓膜炎、麻疹、猩红热、水痘、手足口病、其它地方性传染病和常见寄生虫病等。

掌握：其他传染病和寄生虫病的临床表现、诊断要点、转诊注意事项。

熟悉：其他传染病和寄生虫病的鉴别诊断、治疗原则、预防措施、报告及转诊程序。

了解：其他传染病和寄生虫病的病因、流行病学特征和发病机制。

6）突发急性传染病：如鼠疫、人感染高致病性禽流感、传染性非典型肺炎等。

熟悉：突发急性传染病的临床表现、诊断、预防原则、报告及转诊程序。

了解：突发急性传染病的国内流行趋势。

7）其他相关理论与知识

熟悉：常规消毒、隔离方法、自我防护，传染病社区管理与方法。

传染科轮转学习病种和例数要求见表20。

表20 传染科轮转学习病种和例数要求

疾 病 名 称 最低例数

1.细菌性痢疾及其他感染性腹泻 1

2.病毒性肝炎 1

3.结核病 1

4.获得性免疫缺陷综合症

5.其他常见传染病和寄生虫病（霍乱、流行性脑脊髓膜炎、麻疹、猩红热、水痘、手足口病、其它地方性传染病和常见寄生虫病） 各1

6.突发急性传染病（鼠疫、人感染高致病性禽流感、传染性非典型肺炎）

（十三）精神科（2周）

1.轮转目的

了解精神疾病的主要临床表现、处理原则、常用筛检量表的使用。

2.基本要求

熟悉：焦虑症、抑郁症的常见临床表现和治疗原则；典型精神分裂症的临床表现；精神病人的社区和家庭康复原则与方法。

了解：急性精神药物中毒的识别、初步处理和转诊原则；常用筛检量表如抑郁自评量表（SDS）、焦虑自评量表（SAS）的使用指征；酒精与药物依赖的识别、处理原则和转诊原则。

二、基层实践

基层实践分为三个部分：全科医疗服务技能培训8周；预防保健与基本公共卫生服务技能培训及专业公共卫生机构实践7周（其中专业公共卫生机构实践4周）；社区卫生服务管理技能培训1周。

（一）全科医疗服务技能（8周）

1.基层常见健康问题的诊治

掌握：基层常见健康问题的临床表现、诊断与鉴别诊断、治疗原则及预防措施；常见危急重症院前急救及转运。

（1）上呼吸道感染

掌握：临床表现、诊断与鉴别诊断及治疗原则（防止抗生素的滥用）。

（2）支气管哮喘

掌握：病因、临床表现、诊断与鉴别诊断、治疗原则、缓解期的社区防治。

（3）慢性支气管炎和慢性阻塞性肺疾病

掌握：病因、临床表现、分型、诊断与鉴别诊断、急性发作期及慢性迁延期的治疗、预防管理。

（4）泌尿系统感染

掌握：急、慢性泌尿系统感染的诱因、临床表现、诊断与鉴别诊断、常规治疗及预防措施。

（5）腹泻、胃炎、胃溃疡

掌握：腹泻、胃炎、胃溃疡的诊断与处理。

（6）骨质疏松症、骨性关节病

熟悉：骨质疏松症、骨性关节病的临床特点、治疗原则及一、二、三级预防措施。

（7）常见肿瘤（如肺癌、肝癌、胃癌、乳腺癌、结直肠癌等）、老年痴呆、帕金森病、皮肤瘙痒症

熟悉：常见肿瘤、老年痴呆、帕金森病、皮肤瘙痒症等疾病的临床特点、治疗原则、预防措施及社区管理。

（8）外科常见疾病（如疖肿、痔疮、阑尾炎等）

掌握：常见外科疾病如疖肿、痔疮、阑尾炎等疾病的识别及创伤、犬咬伤的处理。

（9）眼耳鼻喉科常见疾病（结膜炎、角膜异物、鼻炎等）

熟悉：结膜炎、角膜异物、鼻炎、鼻窦炎等常见病的一般处理。

（10）社区常见精神疾病

掌握：焦虑症与抑郁症的常见临床表现和治疗原则。

熟悉：常见身心疾病的识别与处理。

了解：社区接诊精神疾病时的注意事项。

（11）中毒

掌握：食物中毒、有机磷中毒、一氧化碳中毒的院前急救。

（12）常见伤害

掌握：溺水、电击伤、切割伤、动物咬伤等常见伤害的院前处理原则与方法。

（13）常见地方病

熟悉：当地常见地方病的临床特点、治疗原则及一、二、三级预防措施。

2.常见慢性病的诊治与管理

掌握：常见慢性病（高血压、冠心病、脑卒中、糖尿病、慢性阻塞性肺疾病等）的临床特点、诊断与鉴别诊断、治疗原则、一、二、三级预防措施、转诊指征；高血压和糖尿病患者的规范管理。

3.全科医生接诊方式和医患沟通技巧

掌握：全科医生的接诊方式；医患沟通的技巧和注意事项。

熟悉：基层医疗中导致医疗纠纷发生的常见影响因素。

4.全科医疗健康档案的建立、管理与使用

掌握：在培训期间，每人至少完成2份不同健康问题的个人健康档案并能实行动态管理；家庭健康档案2份并进行连续管理。

5.家庭病床管理

了解：家庭病床的建立与管理流程，参与1例家庭病床的管理。

6.社区康复

掌握：脑血管疾病所致功能障碍康复的最佳时间、康复指征及转诊指征。

熟悉：基层医疗中常用康复技术。

7.基层医疗用药

掌握：国家基本药物的用法、用量、常见的不良反应、药物的相互作用以及使用注意事项。

熟悉：其它常用药物的用法、用量、常见的不良反应、药物的相互作用以及使用注意事项。

全科医疗服务主要技能要求见表21。

表21　全科医疗服务基本技能要求

操 作 技 术 名 称 最低例数

掌握：

1.全科医疗健康档案的书写 2

2.规范管理高血压 2

3.规范管理糖尿病 2

了解：

管理家庭病床

基层医疗中常用康复技术

（二）预防保健与基本公共卫生服务技能（7周）

掌握《国家基本公共卫生服务规范》的主要内容。具体内容要求如下：

1.健康教育

掌握：

（1）针对不良生活方式开展合理膳食、控制体重、适当运动、心理平衡、改善睡眠、限盐、控烟、限酒等健康生活方式和可干预危险因素的健康教育。

（2）针对不同病种开展高血压、糖尿病、冠心病、脑卒中、哮喘、乳腺癌和宫颈癌、结核病、病毒性肝炎、艾滋病、流感、手足口病、麻疹、狂犬病、地方病、口腔疾病等重点疾病健康教育。

（3）针对青少年、妇女、老年人、残疾人、0～6岁儿童家长、农民工等不同人群进行健康教育。

（4）开展个体化健康教育。结合以上内容要求，在培训期间参与2个以上健康问题的健康教育，独立完成1次健康教育活动，包括设计计划方案、实施教育、咨询、评价等活动，时间不少于1小时，听众不少于15人。

2.特殊人群保健

（1）儿童保健

掌握：

1）新生儿访视的内容和技巧；儿童体格检查操作技术，如测量身高、体重、头围、胸围等，并能针对体检结果作出恰当的评价和指导；

2）婴儿喂养指导和儿童营养咨询技术；辅助食品添加的顺序及原则；

3）视力及听力筛查，龋齿问题的健康教育；

4）免疫规划程序和预防接种的方法、注意事项以及不良反应处理；

5）儿童系统管理方法。完成新生儿访视、儿童智力发育测查、跟随指导医师进行儿童预防接种。

熟悉：

1）各年龄儿童保健原则、具体措施以及小儿保健组织机构；儿童青少年性教育内容；疫苗冷链管理。

2）儿童常见伤害（烧烫伤、溺水、跌落、意外中毒、电击伤、锐器伤、动物咬伤）及其预防策略；儿童龋齿及预防策略；家庭和学校食品安全；食源性疾病的预防措施。

了解：学校卫生服务的主要内容及工作规范。

（2）老年人保健

掌握：老年人健康综合评估的内容与方法。

熟悉：影响老年人功能减退的因素及其预防措施；运动锻炼的积极作用与方式；老年家庭安全问题及常见家庭伤害预防；老年营养配餐原则。

了解：老年患者的心理问题及处理；社区老年人护理需求的评估；临终关怀的概念、心理与社会方面的照顾原则。

（3）妇女保健

掌握：经期卫生；婚前检查的重要性及计划生育指导；孕期（早期、中期、晚期）的健康管理；产褥期的产后访视、产褥期卫生、乳房护理及母乳喂养的有关知识；产后抑郁症筛检；围绝经期综合征的预防与诊治、激素替代疗法的适应征；妇科常见疾病（宫颈癌、乳腺癌）普查的意义和方法。

（4）残疾人保健

熟悉：国家有关残疾人权益的政策、法规；残疾人心理特点及其特殊的心理需求。

3.社区卫生服务调查与评估

了解：通过参与社区卫生诊断，了解疾病谱及疾病顺位，确定需优先解决的问题。

4.传染病管理

掌握：法定传染病处理方法、报告程序及报告卡填写；常见传染病如肝炎、结核的管理办法。

5.重性精神疾病管理

熟悉：居家重性精神疾病患者的管理要求。

了解：三级防治网络，重性精神疾病管理治疗工作流程。具体包括：患者信息管理、随访评估、分类干预、健康体检、转诊原则及标准、社区家庭康复原则与方法、监护人看护要求。

6.突发事件卫生应急

熟悉：突发公共卫生事件和突发事件紧急医学救援基本知识、信息报告、处置原则、处置流程与规范；国家卫生应急相关法律法规；食品安全事故应急处置与信息报告程序。

了解：突发公共卫生事件分级理念；突发公共卫生事件风险评估和风险沟通的基本概念、基本原则。

7.卫生监督协管

掌握：食品安全信息报告程序、食物中毒和食源性疾病等流行病学调查程序与方法；职业卫生咨询指导内容。

了解： 饮用水卫生安全巡查内容；学校传染病防控措施落实情况巡访内容及报告程序；非法行医和非法采供血信息报告。

基本公共卫生服务主要技能要求见表22。

表22 基本公共卫生服务主要技能要求

操 作 技 术 名 称 最低例数

掌握：

1.新生儿访视 2

2.儿童智力测查 2

3.儿童预防接种 20

4.视力筛查 2

5.听力筛查 2

6.老年人健康综合评估 2

7.产后访视 2

（三）社区卫生服务管理技能（1周）

熟悉：社区卫生服务/全科医疗服务质量考核指标体系、评价方法和程序；本地区和本机构卫生服务信息系统的内容及管理程序；社区卫生服务团队的服务模式、意义；人际沟通技巧，包括医患沟通、与社会工作者等沟通的技巧；国家和地方基本医疗、基本公共卫生、基本药物制度及内容；基层卫生服务机构的设置、管理要求和医疗、药品、财务与信息等管理原则及程序；与基层卫生服务有关的卫生法律法规。

三、理论培训

理论培训共由17门课程、4个系列讲座组成，总共357学时。理论课程按内容分为四个模块：全科医学基本理论与职业理念和综合素质相关课程（81学时）；临床医疗服务相关课程（126学时）；全科医疗与公共卫生服务相关课程（105学时）；综合系列讲座（45学时），其中全科医学基本理论与职业理念和综合素质课程，安排在培训开始的第1-2周，采用集中授课的形式进行；临床医疗服务相关课程和全科医疗与公共卫生服务相关课程，安排在临床培训和基层实践过程中进行；综合系列讲座亦根据具体内容安排在临床培训或基层实践过程中进行。

理论课程的总体进度安排，详见表23。

表23 理论课程总进度安排

第1-2周 第3-84周 第85-100周

集中授课（81） 临床纵向课程（156学时） 社区纵向课程（120学时）

课程名称 学时数 课程名称 学时数 课程名称 学时数

1.全科医学理念与基本理论 27 1.临床基本技能训

练 36 1.临床预防与健康教育 15

2.全科医疗中的常见行为与心理学问题 18 2.临床综合课程讲

座 45 2.社区常见慢性病管理 18

3.全科医疗中的医学伦理、法律法规问题及医患沟通 18 3.常见症状的鉴别

诊断 27 3.社区特殊人群保

健 18

4.卫生经济学在社区卫生服务中的应用 9 4.危重症的识别与

处理 18 4.突发事件卫生应

急 18

5.文献检索与阅读 9 　 5.社区卫生服务管

理 9

　 6.预防接种 9

　 7.重性精神疾病患者管理 9

　 8.法定传染病报告与处理 9

讲座1：现场急救与病人转运 12 讲座1：基层合理用药

讲座2：常见病的规范诊治与管理 9

讲座3：社区康复理论与技术 9 15

注：理论课集中培训按8学时/天；临床和基层医疗实践阶段课程和讲座按4-8学时/周实施。

注：理论课集中培训按8学时/天；临床和基层医疗实践阶段课程和讲座按4-8学时/周实施。

（一）全科医学基本理论与职业理念和综合素质相关课程 （81学时）

1.全科医学理念与基本理论（27学时）

掌握：全科医学的核心理论、全科医生的角色与专业素质要求、全科医生的工作内容与方式、全科医生的临床思维与以病人为中心的照顾原则、全科医疗服务模式与原则、全科医疗与其它专科医疗的区别与联系、临床预防的概念与方法。

熟悉：循证医学基本概念及其在全科医疗中的应用；社区卫生服务与全科医疗相关政策；社区卫生服务、社区卫生诊断的概念及其与全科医疗服务的关系。

了解：全科医学与其它临床专业学科的区别与联系、全科医学与替代医学、全科医疗与社区卫生服务的关系。

2.全科医疗中的常见行为与心理学问题（18学时）

熟悉：行为医学与心理学的研究内容、人类行为与健康的关系、行为障碍的分类与特征；常见心身疾病及其识别、应激、常用心理行为干预技术。

了解：行为障碍的评估与诊断方法等。

3.全科医疗中的医学伦理学、法律问题及医患沟通（18学时）

掌握：伦理学的基本原则及其内涵；伦理学基本原则的实际应用；人际沟通和医患沟通的基本原则。

熟悉：基层医疗中常见伦理问题及其应对原则；与全科医疗服务相关的法律、法规；医生的权利与义务，病人的基本权利；医患沟通。

了解：基层医疗卫生服务机构中常见医疗纠纷案件的原因和特点。

4.卫生经济学在基层医疗卫生服务中的应用（9学时）

熟悉：卫生经济学研究的基本问题；卫生服务需求、卫生服务的投入、产出、卫生总费用、疾病负担、成本效益、成本效用、社会效益、经济效益的概念及其在卫生服务规划中的实际意义；基层医疗卫生服务需要与需求。

了解：卫生经济学的概念；卫生政策制定与卫生经济学研究关系。

5.文献检索与阅读（9学时）

掌握：全科医学主要期刊的特点及其检索途径。

熟悉：文献阅读与利用的原则和方法。

（二）临床医疗服务相关课程 （126学时）

1.临床基本技能训练课程 （36学时）

掌握：门诊及住院病历的书写；规范的体格检查操作；无菌操作的基本步骤与方法；临床常见X线、B超结果的判读；心电图机操作及正常心电图和异常心电图的鉴别；常用实验室检查项目和化验值的解读；轮转各科常用的基本操作技能。

2.临床综合课程 （45学时）

掌握：临床常见病的诊断与鉴别诊断、治疗原则；疾病康复相关知识和技能；慢性病的治疗与管理；常见传染病的临床表现及预防措施；相关卫生政策法规；医学人文相关知识和技能。

3.常见症状的鉴别诊断 （27学时）

掌握：发热、胸痛、腹痛、头晕、意识障碍、呕吐、便血、贫血、搔痒等社区居民常见临床症状的鉴别诊断和处理原则。

4.危重症的识别与处理 （18学时）

掌握：掌握昏迷患者的识别、处理（现场和急诊）与转运原则；呼吸困难的识别、处理与转运原则；心脏骤停的现场处理与心肺复苏的流程；胸痛患者的诊治流程；儿童急性中毒的识别与处理。

熟悉：紧急分娩的处理；急性喉头水肿、眼内异物的紧急处理原则及其转运注意事项。

（三）全科医疗与公共卫生服务相关课程 （105学时）

1.临床预防与健康教育（15学时）

掌握：疾病预防的三级策略；临床预防的概念、方法；健康教育的基本理论、基本方法、设计思路；群体健康教育和个体健康教育的实施步骤与实施注意事项等。

熟悉：健康教育的效果评估；儿童青少年常见伤害及其预防策略；食品安全；常见食源性疾病和食物中毒及其预防策略；食物中毒的调查方法、内容与处理措施。

了解：健康教育、健康宣传、健康促进的区别。

2.社区常见慢性病管理（18学时）

掌握：社区常见慢性疾病（高血压病、糖尿病、脑卒中、冠心病、慢性阻塞性肺疾病）的管理规范、指南及其临床应用、慢性病管理效果评估等。

熟悉：慢性病防治策略与措施；慢性病的主要危险因素及干预；多种慢性病共存时，用药的注意事项及其药物副作用的监测方法。

了解：慢性病的流行概况。

3.社区特殊人群保健（18学时）

掌握：婴幼儿保健、儿童保健、青少年期保健、妇女保健、男性保健、老年保健、残疾人保健的相关组织管理体系、保健内容与保健方法。

4.突发事件卫生应急（18学时）

掌握：突发公共卫生事件和突发事件紧急医学救援基本知识、信息报告、处置原则、处置流程与规范；卫生应急相关法律法规、基本知识和基本技能；我国卫生应急相关法律法规、卫生应急管理体系和基本知识。

熟悉：突发公共卫生事件分级理念。

了解：突发公共卫生事件风险评估和风险沟通的基本概念、基本原则。

5.社区卫生服务管理 （9学时）

熟悉：社区卫生服务管理的相关概念、社区卫生服务发展相关政策及其对社区卫生服务的影响、团队合作的原则、全科医疗健康档案的建立与管理、医疗行为规范、医疗服务质量管理、绩效考核设计与实施等内容。

了解：我国卫生体系的组成和医疗保障制度，重点了解新型农村合作医疗制度。

6.预防接种（9学时）

掌握：国家免疫规划疫苗免疫程序、预防接种禁忌症、疑似预防接种异常反应的诊断、报告要求及处置原则，提高疫苗针对传染病的报告、调查及采样等实践技能。

了解：《疫苗流通和预防接种管理条例》。

7.重性精神疾病患者管理（9学时）

掌握：居家重性精神疾病患者的管理规范和工作流程、患者分类干预要求、考核评估指标等。

8.法定传染病报告与处理（9学时）

掌握：法定传染病报告程序及报告卡填写；法定传染病处理方法。

（四）综合系列讲座 （45学时）

1.现场急救与病人转运（12学时）

掌握：农村常见创伤的现场急救与转运方法和注意事项；气道异物、儿童溺水、电击伤、犬咬伤、蛇咬伤等急症的处理与转运注意事项。

熟悉：急救药物的保存、使用注意事项。

2.常见病的规范诊治与管理（9学时）

掌握：农村常见病规范诊治与科学管理的原则。

熟悉：常见功能性疾病的诊断和处理原则。

3.社区康复理论与技术（9学时）

掌握：社区康复医学的概念、研究范畴。

熟悉：适用于农村常用康复技术及应用原则。

4.基层合理用药（15学时）

掌握：农村地区基层医疗中常见病所使用基本药物的作用原理、用药原则、用药剂量、药物副作用的监测及注意事项；农村地区常用抗生素的合理使用及其用药禁忌；农村地区常用中成药的合理使用及其注意事项。

了解：国家基本药物制度、《国家基本药物处方集》、《国家基本药物临床应用指南》的合理使用原则；癌症患者常用化疗药物的药理作用、副作用及其注意事项。
