# Supplementary material for: Mapping the evolution of fertility support policies in China: A content and instrumental analysis
Source: PLoS One. 2025 Oct 9;20(10):e0332137. doi: 10.1371/journal.pone.0332137 (PMC12510515; doi:10.1371/journal.pone.0332137)
Supplement: S1 Appendix — (ZIP) [file pone.0332137.s001.zip › S1 Appendix. 226 original policy documents/130-卫生部关于印发《全科医生规范化培养标准(试行)》的通知(FBM-CLI-4-180284).docx]

卫生部关于印发《全科医生规范化培养标准(试行)》的通知

发布部门： 卫生部(已撤销) 机构沿革

发文字号：卫科教发〔2012〕48号

发布日期：2012.07.02

实施日期：2012.07.02

时效性： 现行有效

效力级别： 部门规范性文件

法规类别： 医务工作 卫生机构与人员

卫生部关于印发《全科医生规范化培养标准（试行）》的通知

（卫科教发〔2012〕48号）

各省、自治区、直辖市卫生厅局、教育厅局，新疆生产建设兵团卫生局、教育局，卫生部直属有关单位，教育部直属有关高校：

为贯彻落实《国务院关于建立全科医生制度的指导意见》（国发〔2011〕23号），规范并加快全科医生培养，卫生部、教育部组织制定了《全科医生规范化培养标准（试行）》，现印发你们，请结合实际贯彻执行。为做好标准的实施工作，提出以下要求：

一、各省级卫生、教育行政部门应当结合本地区全科医学人才培养需求和培养能力，科学编制本省（区、市）年度全科医生规范化培养计划和中长期培养规划，并于每年10月底前将下一年度培养计划报卫生部和教育部备案。

二、各省级卫生、教育行政部门应当主动协调有关部门，落实培养经费、培训期间人员管理等保障政策，加强全科医生培养能力建设，确保培养工作顺利实施。

三、各省级卫生、教育行政部门和有关培养单位，应当建立和完善包括招录、培养、管理、考核、学位授予等环节的全科医生培养制度，强化培养过程管理，确保培养质量。

2012年7月2日

全科医生规范化培养标准（试行）

根据《国务院关于建立全科医生制度的指导意见》等文件要求，为做好全科医生规范化培养工作，制定本培养标准。

一、培养目标

为基层培养具有高尚职业道德和良好专业素质，掌握专业知识和技能，能独立开展工作，以人为中心、以维护和促进健康为目标，向个人、家庭与社区居民提供综合性、协调性、连续性的基本医疗卫生服务的合格全科医生。

二、培养年限和方式

全科医生规范化培养年限为3年（实际培训时间不少于33个月）。因特殊情况不能按期完成培训任务者，允许申请延长培养年限。

全科医生规范化培养以提高临床和公共卫生实践能力为主，以住院医师的身份在国家认定的全科医生规范化培养基地的各相关临床科室和基层实践基地进行轮转培训，具体培养安排见表1。

（一）临床科室轮转培训

全科住院医师参加临床培养基地中主要临床科室的诊疗工作，接受临床基本技能训练，同时学习相关专业理论知识。总计培训时间为27个月。轮转期间，内科和神经内科病种及其例数的要求主要在病房完成，不足部分在门诊补充，

内科安排病房时间应当不少于8个月，管理床位数不少于5张；神经内科安排病房时间应当不少于1个月，管理床位数不少于3张；儿科轮转可安排在门诊或病房完成；其他科室轮转可安排在门诊完成；部分科室（如康复科、中医科）轮转可在基层实践基地完成；少见病种、地方病、传染病及季节性较强的病种，可采用病例分析、讲座等形式进行学习。临床科室轮转期间每周应当安排不少于半天时间学习相关学科知识。对于轮转时间较长的内科等科室，可结合实际情况分段进行安排，以促进学员的消化和理解。

（二）基层实践培训

主要在基层医疗卫生机构与专业公共卫生机构完成，全科住院医师接受全科医疗服务、预防保健与公共卫生服务、基层医疗卫生管理等技能训练。总计培训时间为6个月，具体时间安排可根据实际情况集中或与临床科室轮转部分穿插进行。

表1 培养方式及时间分配表

培养方式 具体科室 时间分配（月）

临床科室轮转培训（合计27个月） 内科 12

神经内科 2

儿科 2

外科 2

妇产科 1

急诊医学科 3.5

皮肤科 0.5

眼科 0.5

耳鼻咽喉科 0.5

传染科 0.5

精神科 1

康复医学科 0.5

中医科 0.5

选修科室 0.5

基层实践培训（合计6个月） 基层实践基地 6

注：各培养基地根据本标准要求制定轮转计划。

三、培养内容和要求

全科医生规范化培养内容包括理论培训、临床技能培训和基层医疗卫生实践。理论培训内容以临床实际需要为重点，主要包括：①医学伦理与医患沟通；②有关法律、法规（具体见附表有关医疗卫生法律法规推荐目录）；③临床科研设计与方法；④临床专业相关理论；⑤全科医学、社区卫生服务和公共卫生。时间安排可集中或分散在3年培养过程中完成。可采用集中面授、远程教学、临床医学系列讲座、专题讲座、临床案例讨论、读书报告会等多种形式进行。理论和临床技能培训内容详见各科室轮转具体要求。

（一）内科（12个月）

1.轮转目的

（1）通过内科培训，系统学习内科常见病、多发病的基础理论和基本知识，掌握病史采集、体格检查、病历书写等临床技能及心电图检查等必要的诊疗技术；培养缜密的临床思维；掌握内科常见疾病的诊断、治疗，急危重症的处理原则及转诊指征，以及专科治疗后的社区照顾与随访。

（2）内科学习结束时，全科住院医师应具有正确评估及处理内科常见疾病的能力，识别疾病的不稳定状态及高危状态，并能给予正确的急救与转诊。

2.基本要求

（1）症状学

掌握以下常见症状的诊断、鉴别诊断和处理原则：发热、头痛、胸痛、心悸、腹痛、头晕、晕厥、意识障碍、咳嗽、咯血、黄疸、呕吐、腹泻、便秘、血尿、蛋白尿、呕血与便血、水肿（浆膜腔积液）、贫血、关节痛、淋巴结肿大、消瘦、肥胖。

（2）主要疾病

1）心血管系统疾病

①高血压病

掌握：正常血压值，高血压的诊断标准、分型，高血压的分级及危险分层，原发性高血压与继发性高血压的鉴别诊断，高血压的急、慢性并发症，老年高血压的临床特点，高血压的非药物治疗和药物治疗，高血压急症的治疗原则，高血压一、二、三级预防原则，高血压患者健康管理服务规范。

熟悉：高血压药物治疗进展。

了解：高血压流行趋势及发病机制。

②冠心病

掌握：冠心病的诊断，心绞痛的临床分型、临床表现及鉴别诊断、缓解期的治疗、急性期的处理及转诊指征，心肌梗死的诊断依据、心电图特征、与其他疾病的鉴别诊断、处理原则与院前急诊处理、转诊指征及注意事项，介入治疗后病人的社区照顾与随访，冠心病一、二、三级预防原则及康复措施。

熟悉：心肌梗死恢复期、维持期的康复治疗。

了解：缺血性心脏病的临床分型；冠心病介入治疗与外科治疗的方法和适应证。

③慢性心力衰竭

掌握：心力衰竭的定义、分型、临床表现、诊断与鉴别诊断、心功能分级，急性心力衰竭的急救与转诊，常用药物的作用机制、适应证、禁忌证，药物过量的临床表现与处理原则，心力衰竭的预防原则及康复。

熟悉：慢性心力衰竭的病因、诱发因素。

④心律失常

掌握：常见心律失常的临床表现及心电图诊断，低危心律失常的诊断和处理，高危心律失常的识别、急诊处理及转诊、介入治疗后病人的社区照顾与随访。

熟悉：抗心律失常药物的分类、作用特点和临床应用。

了解：常见心律失常的病因、血流动力学改变，永久性心脏起搏器植入术的适应证及术后的注意事项，心律失常介入治疗的适应证和禁忌证。

2）呼吸系统疾病

①上呼吸道感染

掌握：上呼吸道感染的临床表现、诊断、用药原则，抗生素的合理使用原则。

熟悉：上呼吸道症状的鉴别诊断。

了解：上呼吸道感染常见并发症的预防与处理。

②支气管哮喘

掌握：支气管哮喘的临床表现、诊断与鉴别诊断、治疗原则，重症哮喘的诱因及临床表现、急救原则、缓解期社区防治。

熟悉：支气管哮喘的病因、流行病学特点。

了解：支气管哮喘的发病机制，峰流速仪和呼吸机的使用。

③慢性支气管炎和慢性阻塞性肺疾病

掌握：慢性支气管炎和慢性阻塞性肺疾病的病因、临床表现、分型、诊断与鉴别诊断，急性发作期及慢性迁延期的治疗，三级预防措施与康复。

熟悉：慢性支气管炎和慢性阻塞性肺疾病的发病机制、流行病学特点，社区预防管理及家庭氧疗方法。

④肺炎

掌握：肺炎的病因、临床表现与诊断，常见肺炎的鉴别和治疗原则，抗生素的合理使用原则。

熟悉：急性并发症的临床表现及处理原则。

⑤ 睡眠呼吸暂停低通气综合征

掌握：睡眠呼吸暂停低通气综合征的诊断标准、分型、常见病因、临床表现及并发症。

熟悉：睡眠呼吸监测（多导睡眠图），睡眠呼吸暂停低通气综合征的防治原则，无创正压通气治疗机的操作和使用。

了解：睡眠呼吸暂停低通气综合征的发病机制。

⑥急性肺梗死

掌握：急性肺梗死的临床表现、诊断与鉴别诊断、急诊处理原则。

熟悉：急性肺梗死的防治原则。

了解：急性肺梗死的发病机制。

3）消化系统疾病

①急、慢性胃炎

掌握：急、慢性胃炎的分型及临床表现、鉴别诊断和常用治疗方法。

②消化性溃疡

掌握：消化性溃疡的临床表现、诊断及鉴别诊断、药物治疗，并发消化道大出血的急诊处理及转诊措施。

熟悉：消化性溃疡的发病机制、并发症及处理，溃疡病的手术治疗指征。

了解：各项辅助检查的临床意义及检查前的准备。

③急、慢性腹泻

掌握：急、慢性腹泻的常见病因及鉴别诊断、常规治疗、特殊性治疗和预防原则。

熟悉：感染和非感染性肠道疾病的鉴别诊断，肠道传染病报告程序及消毒处理措施。

了解：结肠镜检、钡灌肠的适应证及检查前的准备工作。

④胃食管反流病

掌握：胃食管反流病的临床表现及处理原则。

熟悉：胃食管反流病的发病机制与并发症。

⑤肝硬化

掌握：肝硬化的病因、肝功能代偿期与失代偿期的临床表现、肝性脑病的诱因及预防。

熟悉：肝硬化的发病机制、辅助检查的临床意义、治疗要点。

4）泌尿系统疾病

①泌尿系统感染

掌握：急、慢性泌尿系统感染的诱因、临床表现、鉴别诊断、常规治疗及预防措施。

②肾小球病

掌握：急、慢性肾小球肾炎临床特点、诊断与鉴别诊断，治疗和预防原则。

熟悉：肾上腺皮质激素、免疫抑制剂和抗凝剂的应用，急、慢性肾炎并发症的处理，继发性肾小球疾病（糖尿病肾病、高血压肾小动脉性肾硬化）的诊断与处理原则。

③慢性肾功能不全

掌握：慢性肾功能不全的诊断、治疗原则、预防及早期筛查。

熟悉：非透析疗法，腹膜透析的适应证及实施步骤。

了解：血液透析的适应证。

5）内分泌及代谢系统疾病

①糖尿病

掌握：糖尿病的分型与诊断标准、药物治疗、非药物疗法，低血糖、酮症酸中毒、高血糖高渗状态诱发因素、诊断及救治措施、转诊指征，糖尿病患者健康管理服务规范。

熟悉：糖尿病慢性并发症及处理原则。

了解：糖尿病流行趋势及发病机制。

②血脂异常和脂蛋白异常血症

掌握：脂蛋白异常血症的分类，血脂异常的各项实验室检查标准，药物与非药物治疗方法 。

熟悉：血脂异常治疗进展。

了解：血脂、脂蛋白、载脂蛋白及其代谢。

③痛风

掌握：痛风的临床表现、诊断与鉴别诊断、防治原则。

了解：痛风的病因、发病机制、分类。

④甲状腺功能亢进（Graves病）

掌握：甲状腺功能亢进的临床表现、诊断与鉴别诊断、药物治疗，甲状腺危象诱因及临床表现。

熟悉：甲状腺功能亢进的实验室检查。

了解：甲状腺功能亢进的病因学、分类、放射性碘治疗、手术治疗的适应证、禁忌证及副作用。

⑤甲状腺功能减退

熟悉：甲状腺功能减退的临床表现、诊断与鉴别诊断、处理原则。

了解：甲状腺功能减退的发病机制与并发症。

6）风湿性疾病

①系统性红斑狼疮

掌握：系统性红斑狼疮的临床表现、诊断与鉴别诊断。

熟悉：系统性红斑狼疮的免疫学检查指标、疾病活动度的判定、药物治疗及其预后。

了解：系统性红斑狼疮的病因、发病机制、诱发因素。

②类风湿关节炎

掌握：类风湿关节炎的临床表现、诊断与鉴别诊断、康复。

熟悉：类风湿关节炎的实验室和辅助检查、治疗方法。

了解：类风湿关节炎的病因、发病机制。

7）血液系统疾病

①贫血

掌握：缺铁性贫血、再生障碍性贫血、营养不良性贫血的病因、临床表现、诊断与鉴别诊断、治疗原则、预防和筛查方法。

了解：溶血性贫血的实验室检查。

②出血性疾病

掌握：过敏性紫癜与血小板减少性紫癜的病因、诊断与鉴别诊断、治疗原则。

③急、慢性白血病

熟悉：急、慢性白血病的临床表现。

了解：急、慢性白血病的实验室检查、诊断、药物治疗原则。

8）老年疾病与老年问题

掌握：常见老年疾病与老年问题的诊治（骨质疏松、跌倒、前列腺增生、痴呆、尿失禁、便秘等），老年人患病的特点，老年人合理用药的原则。

了解：老龄化社会的标准，老年人药物代谢特点（包括药物在体内的吸收、分布、代谢和排泄特点、药物耐受性、组织感受性、药物间的相互作用、药物与疾病相互作用等），老年人心理与沟通。

9）各系统常见肿瘤

掌握：各系统常见肿瘤的临床表现与早期诊断方法。

熟悉：各系统常见肿瘤的实验室和辅助检查、治疗原则、康复。

了解：各系统常见肿瘤的病因、发病机制。

10）地方病

熟悉：当地常见地方病的病因、临床表现、诊断与鉴别诊断、治疗原则、监测和防治措施。

内科轮转期间学习病种及其例数要求，见表2。

表2　内科学习病种和例数要求

病 种 最低例数

心血管系统疾病

① 高血压 10

② 冠心病 5

③ 充血性心力衰竭 5

④ 常见心律失常（包括窦性心动过速、房性期前收缩、房性心动过速、

心房扑动、 心房颤动、室性期前收缩、室性心动过速、心室扑动、心室颤动、窦性心动过缓、房室传导阻滞） 15

呼吸系统疾病

① 呼吸道感染 5

② 支气管哮喘 5

③ 慢性支气管炎和慢性阻塞性肺疾病 5

④ 肺炎 5

⑤ 睡眠呼吸暂停低通气综合征 2

⑥ 急性肺梗死 2

消化系统疾病

① 急、慢性胃炎 5

② 消化性溃疡 5

③ 急、慢性腹泻 2

④ 胃食管反流病 2

⑤ 肝硬化 2

泌尿系统疾病

① 泌尿系统感染 10

② 肾小球肾病 10

③ 慢性肾功能不全 5

内分泌及代谢系统疾病

①糖尿病 10

②血脂异常和脂蛋白异常血症 5

③痛风 2

④甲状腺功能亢进（Graves病） 2

风湿性疾病

① 系统性红斑狼疮 2

② 类风湿关节炎 2

　 续表

病 种 最低例数

血液系统疾病

① 贫血（包括缺铁性贫血、再生障碍性贫血、营养不良性贫血） 5

② 出血性疾病 2

③ 急、慢性白血病 不做具体要求

老年疾病与老年问题（包括骨质疏松、跌倒、前列腺增生、痴呆、尿失禁、便秘等） 5

各系统常见肿瘤 5

地方病 根据各地要求

（3）基本技能要求：见表3。

表3　内科基本技能要求

操 作 技 术 名 称 最低例数

掌握：

系统查体和物理诊断 10

吸痰术 10

胸部X线读片 10

心电图机操作，书写心电图诊断报告（包括左、右心室肥大，心房肥大，

左右束支传导阻滞，房室传导阻滞，心肌梗死及各种常见心律失常） 30

直肠指诊检查技术 2

临床常用检验正常值及临床意义 50

熟悉： 不做具体要求

胸腔、腹腔及骨髓穿刺技术

输液的操作方法、步骤以及注意事项

导尿术的适应证、操作方法及注意事项

灌肠法的适应证、操作方法及注意事项

各种注射操作方法、适应证及注意事项

标本（粪便、尿、痰、血液标本）采集方法、步骤

了解： 不做具体要求

动态心电图，动态血压测定，心电图运动试验，超声心动图、颈动脉超声的

应用范围

支气管镜的适应证、禁忌证、并发症及病人检查的准备

肺功能测定方法

CT检查的适应证、禁忌证

磁共振检查的适应证、禁忌证

胃镜、结肠镜、胃液分析、十二指肠液分析及消化道X线检查的适应

症、禁忌证、并发症

超声和核医学检查的适应证、禁忌证

三腔两囊管插管术的适应证、禁忌证、操作方法

骨髓穿刺的适应证、禁忌证及操作方法；输血的适应证

（二）神经内科（2个月）

1.轮转目的

通过神经内科培训，系统学习神经内科常见疾病的基础理论和基本知识，掌握病史采集、体格检查、病历书写等临床技能；了解CT、MRI等必要的诊疗技术；培养正确的临床思维；掌握神经内科常见疾病的诊断和处理。

2.基本要求

（1）症状学

掌握以下常见症状的诊断、鉴别诊断和处理原则：意识障碍、认知障碍、构音障碍、眼球运动障碍、面肌瘫痪、听觉障碍、眩晕、头痛、晕厥、癫痫发作、感觉障碍、瘫痪、肌肉萎缩、步态异常、不自主运动、共济失调、尿便障碍。

（2）主要疾病

1）短暂性脑缺血发作

掌握：短暂性脑缺血发作的定义、临床表现、诊断与鉴别诊断、治疗原则，院前急诊处理及转诊指征。

熟悉：短暂性脑缺血发作的治疗进展、血管介入治疗。

了解：短暂性脑缺血发作的病因及发病机制。

2）动脉粥样硬化性脑血栓形成

掌握：动脉粥样硬化性脑血栓形成的定义、临床表现、诊断与鉴别诊断，院前急诊处理及转诊指征，缓解期的治疗，针对可干预的危险因素的二、三级预防原则，康复指征。

熟悉：急性期的处理原则。

了解：动脉粥样硬化性脑血栓形成的病因、发病机制。

3）脑栓塞

掌握：脑栓塞的定义、临床表现、诊断与鉴别诊断，院前急诊处理及转诊指征，缓解期的治疗，针对可干预的危险因素的二、三级预防原则，康复指征。

熟悉：脑栓塞的病因及发病机制。

4）腔隙性脑梗死

掌握：腔隙性脑梗死的定义、临床表现、诊断与鉴别诊断、治疗原则。

熟悉：腔隙性脑梗死的常见综合征。

了解：腔隙性脑梗死的病因及发病机制。

5）脑出血

掌握：脑出血的定义、临床表现、诊断与鉴别诊断，院前急诊处理及转诊指征，急性期不同的处理原则，康复指征。

熟悉：脑出血的病因。

了解：脑出血的发病机制、病理。

6）蛛网膜下腔出血

掌握：蛛网膜下腔出血的定义、临床表现、诊断与鉴别诊断，院前急诊处理及转诊指征，治疗原则，康复指征。

熟悉：急性期的处理。

了解：蛛网膜下腔出血的病因、发病机制、病理。

7）高血压脑病

掌握：高血压脑病的定义、临床表现、诊断与鉴别诊断，院前急诊处理及转诊指征，治疗原则和处理方法，恢复期的预防，康复指征。

了解：高血压脑病的病因。

8）其他疾病：如血管性痴呆、阿尔茨海默病、帕金森病、面神经麻痹、偏头痛、脑肿瘤、脑膜炎等。

熟悉：上述疾病的诊断要点、治疗方法及康复措施。

了解：上述疾病的病因、发病机制。

神经内科轮转期间学习病种及其例数要求，见表4。

表4　神经内科学习病种和例数要求

病 种 最低例数

短暂性脑缺血发作 3

动脉粥样硬化性脑血栓形成 3

脑栓塞 3

腔隙性脑梗死 3

脑出血 3

蛛网膜下腔出血 2

高血压脑病 2

其他疾病（如血管性痴呆、阿尔茨海默病、帕金森病、面神经麻痹、偏头痛、

三叉神经痛、重症肌无力、癫痫、多发性硬化症、脑肿瘤、脑膜炎等） 不做具体要求

（3）基本技能要求：见表5。

表5　神经内科基本技能要求

操作技术名称 最低例数

掌握：

体格检查 5

头颅CT阅片 5

了解： 不做具体要求

头颅MRI阅片

腰椎穿刺

（三）儿科 （2个月）

1.轮转目的

通过儿科培训，学习儿科常见疾病的基础理论和基本知识，掌握儿科病史采集和体格检查的特殊性，小儿用药特点、药物剂量的计算方法以及正确配奶的方法，小儿生长发育指标的正常值和测量方法，儿科常见疾病的诊断和处理原则；了解儿童生长发育规律和影响因素。

2. 基本要求

（1）症状学

掌握以下常见症状的诊断、鉴别诊断和处理原则：小儿发热、咳嗽、气促、喘息、呕吐、肥胖、婴儿哭闹、青紫、头痛、小儿高血压、腹痛、肝脾肿大、腹部肿块、便血、血尿、智力运动发育落后。

（2）小儿生长发育与主要疾病

1）小儿生长发育与评估

掌握：体重、身长、头围、前囟、牙齿、体格发育指标的正常值测量以及计算方法。

熟悉：小儿神经、精神发育的规律。

了解：小儿生长发育的规律、临床意义及影响生长发育的因素。

2）新生儿常见疾病

①新生儿窒息

掌握：Apgar评分法，新生儿窒息的复苏以及转诊指征。

熟悉：新生儿窒息的临床表现（青紫窒息、苍白窒息）及诊断。

了解：新生儿窒息的病因、并发症及后遗症。

②新生儿肺炎

掌握：新生儿肺炎的预防措施及转诊原则。

熟悉：新生儿肺炎的临床表现及诊断。

了解：新生儿肺炎的病因及治疗原则。

③新生儿黄疸

掌握：新生儿黄疸的分类诊断及鉴别诊断。

熟悉：新生儿生理性黄疸的发展过程。

了解：新生儿时期胆红素代谢的特点，高胆红素血症、胆红素脑病的临床表现、危害性及防治方法。

④新生儿败血症

熟悉：新生儿败血症的诊断与防治原则。

了解：新生儿败血症的病因。

⑤新生儿出血症

了解：新生儿出血症的病因及发病机制。

3）营养性疾病

①营养不良

掌握：营养不良的临床表现、诊断、治疗原则与预防措施。

熟悉：营养不良的病因、病理生理。

②单纯性肥胖症

掌握：单纯性肥胖症的临床表现、诊断、防治措施。

了解：单纯性肥胖症的定义、病因及病理生理。

③小儿贫血

掌握：WHO关于贫血的诊断与鉴别诊断、治疗及预防原则。

熟悉：营养性缺铁性贫血及营养性巨幼红细胞性贫血的病因、发病机制及临床表现。

了解：小儿铁代谢及小儿叶酸、维生素B12代谢的特点。

④佝偻病及婴儿手足搐搦症

掌握：佝偻病及婴儿手足搐搦症的临床表现、各期的诊断、治疗及预防原则。

熟悉：佝偻病及婴儿手足搐搦症的病因及发病机制。

了解：维生素D的主要生理功能，维生素D过量或中毒的临床表现、防治措施。

4）各系统疾病

①呼吸道疾病（包括上呼吸道感染、哮喘、喉炎、肺炎）

掌握：呼吸道疾病的诊断、处理原则及转诊指征。

熟悉：呼吸道疾病的临床特点。

②小儿腹泻

掌握：小儿腹泻的临床表现、诊断要点及治疗原则（包括液体治疗）。

熟悉：小儿腹泻的病因、发病机制及鉴别诊断。

③小儿腹痛

熟悉：小儿腹痛的病因、检查方法、鉴别诊断、内外科处理原则及转诊指征。

④小儿惊厥、癫痫

掌握：高热惊厥的诊断与鉴别诊断、急救措施和预防原则；癫痫的治疗原则，常用抗癫痫药的使用方法。

熟悉：高热惊厥的临床表现；各型癫痫的诊断要点。

了解：高热惊厥的病因、发病机制；癫痫的病因、临床分型及各型特点，癫痫持续状态的定义、危害性及治疗原则。

⑤急性肾炎及肾病综合征

掌握：急性肾炎和肾病综合征的诊断要点和转诊指征。

熟悉：急性肾炎和肾病综合征的病因及发病机制。

了解：急性肾炎重症病例（高血压脑病及急性肾衰竭）、慢性肾炎急性发作及泌尿系统感染的诊断要点与鉴别。

⑥先天性心脏病

熟悉：小儿各年龄段心界、心率和血压的正常值，先天性心脏病的临床分类及特点。

了解：小儿循环系统解剖生理特点，房间隔缺损、室间隔缺损、动脉导管未闭、法洛四联征的临床表现、诊断要点及转诊原则。

⑦病毒性心肌炎

熟悉：病毒性心肌炎的临床表现、诊断要点及转诊原则。

了解：病毒性心肌炎的发病机制及预防原则。

⑧小儿糖尿病

掌握：小儿糖尿病的诊断、治疗原则与日常管理。

熟悉：小儿糖尿病实验室检查，酮症酸中毒的早期发现和紧急处理。

了解：小儿糖尿病的发病特点。

⑨小儿急性白血病

掌握：小儿急性白血病的常见临床表现。

熟悉：小儿急性白血病的诊断方法。

了解：小儿急性白血病的治疗原则。

5）小儿常见急性传染病（包括脊髓灰质炎、麻疹、水痘、风疹、流行性腮腺炎、猩红热、手足口病等）

掌握：小儿常见急性传染病的临床表现及诊断要点。

熟悉：鉴别诊断、防治原则及常见并发症。

了解：小儿常见急性传染病病因、流行病学特点、监测与报告及随诊要求。

儿科轮转期间学习病种及其例数要求，见表6。

表6 儿科学习病种和例数要求

病 种 最低例数

新生儿常见疾病

①新生儿窒息 2

②新生儿肺炎 2

③新生儿黄疸 2

④新生儿败血症 不做具体要求

⑤新生儿出血症 不做具体要求

营养性疾病

①营养不良 2

②单纯性肥胖症 2

③小儿贫血 2

④佝偻病及婴儿手足搐搦症 2

病 种 最低例数

各系统疾病

①吸道疾病（包括上呼吸道感染、哮喘、喉炎、肺炎） 5

②小儿腹泻 5

③小儿腹痛 不做具体要求

④小儿惊厥、癫痫 2

⑤急性肾炎及肾病综合征 2

⑥先天性心脏病 不做具体要求

⑦病毒性心肌炎 不做具体要求

⑧小儿糖尿病 1

⑨小儿急性白血病 1

小儿常见急性传染病（包括脊髓灰质炎、麻疹、水痘、风疹、流行性腮

腺炎、猩红热、手足口病等） 5

（3）基本技能要求：见表7。

表7 儿科基本技能要求

操作技术名称 最低例数

掌握：

小儿生长发育与评估 10

小儿查体方法 5

婴儿配奶方法 5

小儿用药特点、药物剂量计算方法 5

了解： 不做具体要求

小儿股静脉穿刺、头皮静脉穿刺、肌肉注射、皮下皮内注射、

儿童心肺复苏等

（四）外科 （2个月）

1.轮转目的

（1）通过外科培训，学习外科（主要为普通外科及骨科）常见疾病的基础理论和基本知识，掌握病史采集、体格检查等临床技能及清创缝合、引流、换药、拆线等必要的诊疗技术；建立正确的临床思维；掌握社区外科常见疾病的诊断和处理原则。

（2）外科学习结束时，全科住院医师应具有正确评估及处理外科常见疾病的能力，掌握危重症患者的识别及转诊指征，并能给予正确的急救与处理。

2.基本要求

（1）症状学

掌握以下常见症状的诊断与鉴别诊断、处理原则：体表肿物、颈部肿物、乳腺肿物、腹部肿块、腹痛、呕血与便血、腰腿痛和颈肩痛。

（2）主要疾病

1）外科感染

掌握：常见软组织感染的病因、临床表现、防治原则，抗生素的合理应用。

熟悉：常见软组织感染的概念和转归，败血症、脓血症、破伤风、局部化脓感染的临床表现、防治原则和转诊指征。

了解：气性坏疽的临床表现和转诊原则。

2）水、电解质和酸碱平衡失调

熟悉：水、电解质和酸碱平衡失调的临床表现、防治原则和转诊指征。

了解：体液平衡及渗透压调节、酸碱平衡的维持。

3）颈部疾病

掌握：甲状腺肿物的诊断与鉴别诊断、治疗原则。

熟悉：颈部肿块的鉴别诊断。

4）乳房疾病

掌握：乳房检查方法；急性乳腺炎的诊断、预防和治疗；乳房脓肿的切开引流、注意事项和并发症；乳腺增生的分型、临床表现和鉴别诊断。

熟悉：乳房肿块、乳腺增生、乳头溢液的诊断；乳腺癌的临床表现、诊断方法和防治原则。

5）腹部疾病

①腹外疝

熟悉：腹外疝的诊断、鉴别诊断要点、治疗原则及转诊指征。

了解：腹外疝的临床类型。

②阑尾炎

掌握：急、慢性阑尾炎的临床表现、诊断与鉴别诊断、治疗原则，转诊指征。

熟悉：特殊类型阑尾炎的临床特点。

了解：手术治疗的方法和并发症。

③肠梗阻

掌握：单纯性与绞窄性肠梗阻的临床特点、治疗原则。

熟悉：急性肠梗阻的病因、临床分型和治疗原则。

④溃疡病穿孔

掌握：溃疡病穿孔的临床表现、诊断与外科治疗指征。

了解：溃疡病穿孔的外科手术方法和并发症。

⑤胆囊炎、胆石症

掌握：急性胆囊炎、急性化脓性胆管炎、胆石症、胆道蛔虫症的临床表现、诊断，有关外科黄疸型疾病的鉴别诊断和治疗原则。

熟悉：胆囊炎、胆石症超声检查的诊断依据。

了解：T型管放置、护理、造影及拔管适应证；经皮肝穿刺胆管造影（PTC）、经皮肝穿刺置管引流（PTCD）、内镜逆行胰胆管造影（RCP）的适应证。

⑥胰腺疾病

掌握：急、慢性胰腺炎的临床表现、诊断和治疗原则。

熟悉：急、慢性胰腺炎的病理；胰腺癌和壶腹部癌的临床表现和诊断方法。

了解：慢性胰腺炎的临床表现、诊断和治疗原则。

⑦胃癌、结直肠癌与肝癌

掌握：胃癌、结直肠癌与肝癌的临床表现和诊断方法。

熟悉：胃癌、结直肠癌与肝癌的处理原则和手术适应证。

6）肛门直肠疾病

掌握：肛裂、肛瘘、痔的临床表现与防治原则。

7）周围血管疾病

掌握：下肢静脉曲张的临床表现、特殊检查、防治措施、手术的适应证。

了解：血栓闭塞性脉管炎、下肢深部静脉炎、囊状淋巴瘤的诊断要点和治疗原则。

8）泌尿系结石与前列腺疾病

掌握：急性尿潴留的病因、治疗原则；泌尿系结石和前列腺炎的临床诊断和处理原则。

熟悉：泌尿系结石的病理；前列腺增生症、前列腺癌的诊断要点、治疗原则和预防。

9）腰腿痛和颈肩痛

掌握：腰腿痛和颈肩痛的临床特点、诊断、治疗和康复原则；疼痛封闭治疗的适应证、方法和注意事项。

了解：腰腿痛和颈肩痛的病因及发病机制；各关节穿刺部位和方法。

10）骨关节病与骨肿瘤

熟悉：急、慢性血源性骨髓炎的临床表现和治疗原则；退行性骨关节病的诊断与治疗。

了解：骨结核的好发部位、病理变化特点、诊断与治疗；良性与恶性骨肿瘤的临床特点、治疗、康复原则。

11）其他相关理论与知识

掌握：外科常用的消毒剂、消毒方法及注意事项，无菌操作原则。

熟悉：清创原则与方法。

了解：灭菌的常用方法及灭菌后物品的使用期限。

外科轮转期间学习病种及其例数要求，见表8。

表8 外科学习病种和例数要求

病 种 最低例数

　外科感染 5

水、电解质和酸碱失调 不做具体要求

颈部疾病 3

乳房疾病 3

腹部疾病

①腹外疝 不做具体要求

②阑尾炎 2

③肠梗阻 2

④溃疡病穿孔 2

⑤胆囊炎、胆石症 2

⑥胰腺疾病 2

⑦胃癌、结直肠癌与肝癌 2

肛门直肠疾病 2

周围血管疾病 2

泌尿系结石与前列腺疾病 2

腰腿痛和颈肩痛 5

骨关节病与骨肿瘤 不做具体要求

（3）基本技能要求：见表9。

表9　外科基本技能要求

操作技术名称 最低例数

掌握：

外科疾病的查体和物理诊断 3

无菌操作 3

小伤口清创缝合 3

各种伤口换药与拆线 3

体表肿物切除 3

浅表脓肿的切开引流 3

小夹板、石膏固定 3

疼痛封闭治疗 3

肛门指诊操作 3

了解： 不做具体要求

各关节穿刺方法

肛门镜的使用方法

（五）妇产科 （1个月）

1.轮转目的

通过妇产科培训，熟悉门诊常见妇科疾病的处理流程；掌握围生期保健的主要内容和相应的处理原则；掌握计划生育指导和非手术措施的适应证，了解常用计划生育手术的适应证；对常见妇产科问题做出正确的诊断和评估，同时做出恰当的转诊。

2.基本要求

（1）症状学

掌握以下常见症状的诊断、鉴别诊断和处理原则：白带异常、阴道异常出血、急性腹痛、慢性腹痛、盆腔肿物、腹胀。

（2）主要疾病

1）常见宫颈和阴道炎症

熟悉：各种阴道炎的诊断与鉴别诊断、治疗；学习阴道分泌物悬滴检查方法和宫颈细胞学筛查的方法和结果判断。

2）阴道异常出血

了解：导致阴道异常出血的常见疾病及其特征；早孕HCG试纸使用方法、β-HCG指标的临床意义、妇科B超检查的临床应用、宫颈癌普查的宫颈涂片方法及病理结果的判断等。

3）子宫肌瘤、卵巢囊肿

熟悉：子宫良性肿瘤的临床表现、处理原则，包括适时转诊的指征。

了解：妇科窥阴器的使用方法和双合诊技术、妇科B超检查的临床应用。

4）导致急性腹痛的疾病

熟悉：异位妊娠、急性盆腔炎、卵巢囊肿蒂扭转的临床表现、常用辅助检查方法、适时转诊的指征。

（3）基本技能要求

1）围生期保健

掌握：妊娠早、中、晚期诊断及早孕HCG试纸的使用方法；孕期保健的检查内容和意义；临产表现及护理；产后保健内容等。

熟悉：高危妊娠的识别、诊断及转诊适应证；异常产褥的诊断、处理原则和转诊指征；产后抑郁症的诊断处理原则。

2）更年期保健

掌握：围绝经期综合征的临床表现及诊断；常见的健康问题及预防。

熟悉：更年期骨质疏松症的预防与治疗。

3）计划生育

掌握：各种避孕方法的适应证和禁忌证，避孕失败后补救措施的适应证和禁忌证；优生优育的指导及内容。

熟悉：人工流产术后并发症的观察及处理原则；药物流产常见的并发症及转诊指征。

4）其他技能要求，见表10。

表10　妇产科基本技能要求

操作技术名称 最低例数

掌握：

围生期保健 10

更年期保健 5

计划生育 5

熟悉： 不做具体要求

孕期四步触诊检查法

骨盆外测量

了解：

妇科检查双合诊技术 不做具体要求

窥阴器的使用方法

子宫颈涂片技术

阴道分泌物悬滴检查

（六）急诊科 （3.5个月）

1.轮转目的

（1）通过急诊科培训，在上级医师指导下诊治急诊病人，学习常见急症的诊断方法与抢救治疗，掌握病史采集、体格检查等临床技能及心电图检查等必要的诊疗技术；掌握心肺复苏术、电除颤术、洗胃、包扎、患者搬运等急救技能；培养正确的临床思维；掌握社区常见急症的诊断与处理。

（2）急诊科学习结束时，全科住院医师应具有正确评估及处理社区常见急症的能力，识别疾病的不稳定状态及高危状态，并能给予正确的急救与处理，同时适时准确地转诊病人。

2.基本要求

（1）基本理论

掌握：突发急症主要判断方法及紧急处置原则；常用急救药物的作用、副作用及使用方法；生命体征观察方法、记录及临床意义；院前急救流程。

熟悉：高级心肺复苏术的步骤和条件；心肺复苏的终止指征。

了解：现代急诊医学主要内容；现代急诊医疗体系基本组织形式；灾难抢救、重大交通事故、地震、水灾、火灾等重大抢救处理及防疫原则。

（2）主要疾病

1）心脏骤停

掌握：心脏骤停的快速判定、初级心肺复苏（BLS）、电除颤与简易呼吸器的使用。

熟悉：心脏骤停的高级心肺脑复苏。

了解：心脏骤停的定义、病因、病理生理。

2）急性气道梗阻

掌握：急性气道梗阻的判断与处理方法。

熟悉：急性气道梗阻的常见原因。

了解：环甲膜穿刺术的指征。

3）急性呼吸衰竭

掌握：急性呼吸衰竭的临床表现、诊断标准与治疗原则。

熟悉：急性呼吸衰竭常见病因。

了解：呼吸机的使用指征及基本原理。

4）急性呼吸窘迫综合征（ARDS）

掌握：ARDS的诊断标准。

熟悉：ARDS的常见病因。

了解：ARDS的病理生理改变与影像学特点。

5）自发性气胸

掌握：自发性气胸的临床表现与体征及影像学特点。

熟悉：自发性气胸的病因及处理方法。

了解：自发性气胸的发病机制。

6）心绞痛、急性心肌梗死

掌握：心绞痛、急性心肌梗死的临床表现、心电图特点、诊断及鉴别诊断、急救及转送方法。

熟悉：心肌坏死标志物的临床意义。

了解：冠心病的溶栓治疗，冠心病的介入治疗与外科手术治疗的方法和适应证。

7）休克

掌握：过敏性休克、低血容量性休克和感染性休克在社区中的急救技术处理及转诊原则。

熟悉：休克的临床表现、诊断和防治。

了解：休克的病因、病理生理变化。

8）上消化道出血

掌握：上消化道出血的诊断、救治及转送方式。

熟悉：上消化道出血的常见病因、失血量估计、是否继续出血的判断方法。

了解：上消化道出血内镜治疗与外科手术原则。

9）急性肾衰竭

熟悉：急性肾衰竭的常见病因、诊断要点及血液透析的指征。

了解：急性肾衰竭的病因、发病机制及分类、早期治疗和进一步治疗的原则。

10）癫痫持续状态

熟悉：癫痫持续状态的分类及临床表现、紧急救治及搬运方法。

了解：癫痫持续状态的病因。

11）中毒与意外伤害

①常见中毒

掌握：常见中毒的诊断与鉴别诊断、急救及转送原则。

了解：毒物在体内的代谢过程及中毒机制。

②中暑

掌握：中暑的紧急处理方法。

了解：中暑的病理生理及临床分类。

③淹溺

掌握：淹溺的现场急救方法。

了解：淹溺的病理生理。

④动物咬、蜇伤

掌握：动物咬、蜇伤的紧急处理方法。

熟悉：动物咬、蜇伤的临床表现及处理原则。

了解：主要毒理作用机制及预后。

12）急腹症

掌握：急腹症的临床表现与处理原则。

熟悉：急腹症的诊断方法和转诊指征。

13）创伤

掌握：多发创伤现场急救及转送原则；颅脑外伤诊断程序、现场急救原则及转送指征；气胸、肺挫伤、肋骨骨折的诊断、现场急救及转送指征；骨折急救方法以及颈椎外伤、脊椎外伤、合并截瘫、四肢骨折病人的搬运方法；手外伤伤口紧急处理方法及断指保存方法；烧伤现场急救原则及转送指征。

熟悉：多发性创伤诊断程序；骨折伤情判断程序。

了解：颅脑外伤分类；胸部外伤分类；骨折分类；手外伤诊断要点；烧伤进一步治疗原则。

14）其他相关理论与知识

掌握：呼叫“120”急救电话要点；使用救护车转运病人指征和转运前准备。

急诊医学科轮转期间学习病种及其例数要求，见表11。

表11　急诊医学科学习病种和例数要求

病 种 最低例数

心脏骤停 2

急性气道梗阻 2

急性呼吸衰竭 2

急性呼吸窘迫综合征（ARDS） 2

自发性气胸 2

心绞痛、急性心肌梗死 5

休克 2

上消化道出血 2

急性肾衰竭 不做具体要求

癫痫持续状态 2

中毒与意外伤害（包括常见中毒、中暑、淹溺、动物咬、蜇伤等） 2

急腹症 5

创伤 5

（3）基本技能要求：见表12。

表12　急诊医学科基本技能要求

操作技术名称 最低例数

掌握：

初级心肺复苏技术、电除颤术、简易呼吸器的使用 3

洗胃术操作方法及准备工作 3

创伤的包扎止血固定 3

了解： 不做具体要求

气管插管或切开术、环甲膜穿刺术等

（七）皮肤科 （0.5个月）

1.轮转目的

通过皮肤科培训，学习皮肤科常见疾病的基础理论和基本知识，掌握病史采集、体格检查等临床技能；掌握皮肤科常见疾病的诊断和处理原则；了解皮肤科常用的诊疗技术；培养正确的临床思维。

2.基本要求

（1）症状学

掌握以下常见症状的诊断与鉴别诊断、处理原则：包括斑疹、丘疹、风团、水疱、脓疱、浸渍、糜烂、溃疡。

（2）主要疾病

1）湿疹

掌握：湿疹的临床表现、诊断与鉴别诊断、治疗原则。

熟悉：重症湿疹的处理原则。

了解：湿疹的病因与发病机制。

2）接触性皮炎

掌握：接触性皮炎的临床表现、诊断与鉴别诊断、治疗原则。

熟悉：重症接触性皮炎的处理原则。

了解：接触性皮炎的病因与发病机制。

3）药疹

掌握：药疹的临床表现、诊断与鉴别诊断、治疗原则、预防方法。

熟悉：重症药疹的临床表现及处理原则。

了解：药疹的病因和发病机制。

4）荨麻疹

掌握：急、慢性荨麻疹的临床表现、诊断、治疗，重症荨麻疹的急救处理。

熟悉：特殊类型荨麻疹的临床表现与治疗原则。

了解：荨麻疹的病因及发病机制。

5）银屑病

掌握：寻常性银屑病的临床表现、诊断与治疗原则。

了解：银屑病的病因及诱发因素；特殊类型银屑病表现。

6）皮肤真菌感染、癣

掌握：手足癣及体股癣的临床表现、诊断与鉴别诊断、治疗原则。

熟悉：头癣、甲癣及花斑癣的临床表现及治疗方法。

了解：浅部真菌病的常见病原菌及检查方法。

7）单纯疱疹和带状疱疹

掌握：单纯疱疹和带状疱疹的临床表现、诊断与鉴别诊断、治疗原则。

8）疣

了解：各种疣（寻常疣、扁平疣、传染性软疣）的临床表现及治疗原则。

9）性传播疾病

熟悉：梅毒、淋病的病因及传播途径；后天性梅毒的分期、各期的临床表现、胎传梅毒的临床表现、梅毒血清学检查的临床意义、梅毒的诊断与鉴别诊断、治疗；淋病的临床表现、诊断及治疗原则。

了解：性传播性疾病概念及目前我国性传播性疾病的概况；非淋菌性尿道炎、生殖器疱疹、尖锐湿疣的临床表现、诊断及治疗原则。

10）日光性皮炎

掌握：日光性皮炎的临床表现、诊断与鉴别诊断、治疗原则。

了解：日光性皮炎的病因、发病机制。

11）痤疮

掌握：寻常性痤疮的临床表现、诊断、鉴别诊断及治疗原则。

了解：痤疮的病因、发病机制。

皮肤科轮转期间学习病种及其例数要求，见表13。

表13　皮肤科学习病种和例数要求

病 种 最低例数

湿疹 5

接触性皮炎 5

药疹 2

荨麻疹 5

银屑病 2

皮肤真菌感染、癣 5

单纯疱疹和带状疱疹 5

日光性皮炎 1

痤疮 3

疣 不做具体要求

性传播疾病 不做具体要求

（3）基本技能要求

了解：皮肤活检方法，冷冻、激光的治疗适应证。

（八）眼科（ 0.5个月）

1.轮转目的

通过眼科培训，学习眼科常见疾病的基础理论和基本知识，掌握病史采集、体格检查等临床技能；掌握眼科常见疾病的诊断和处理原则；了解眼科常用的诊疗技术；培养正确的临床思维。

2.基本要求

（1）症状学

掌握以下常见症状的诊断与鉴别诊断、处理原则：视力障碍、感觉异常（眼红、眼痛、畏光、流泪、眼睑痉挛等）、外观异常、视疲劳。

（2）主要疾病

1）睑腺炎（麦粒肿）

掌握：睑腺炎（麦粒肿）的临床表现、诊断与鉴别诊断、治疗原则。

熟悉：睑腺炎（麦粒肿）的局部治疗方法。

2）睑板腺囊肿（霰粒肿）

掌握：睑板腺囊肿（霰粒肿）的临床表现、诊断与鉴别诊断、治疗原则。

熟悉：睑板腺囊肿（霰粒肿）的局部治疗方法。

3）结膜炎

掌握：结膜炎的分类、临床表现、诊断与鉴别诊断、治疗原则。

熟悉：结膜炎的病因、治疗用药。

4）白内障

掌握：白内障的分型、临床表现、诊断与鉴别诊断、治疗原则。

熟悉：老年性白内障的分型、分期。

5）青光眼

掌握：青光眼的分型、临床表现、诊断与鉴别诊断、治疗原则。

熟悉：青光眼的分期、治疗用药。

眼科轮转期间学习病种及其例数要求，见表14。

表14　眼科学习病种和例数要求

病 种 最低例数

睑腺炎（麦粒肿） 3

睑板腺囊肿（霰粒肿） 3

结膜炎 5

白内障 5

青光眼 3

（3）基本技能要求：见表15。

表15　眼科学习基本技能要求

操作技术名称 最低例数

掌握：

视力检查、眼底镜的使用及正常眼底的识别 10

眼冲洗治疗 3

外眼一般检查 5

结膜异物处理方法 3

了解： 不做具体要求

眼压测定

（九）耳鼻咽喉科（0.5个月）

1.轮转目的

通过耳鼻喉科培训，学习耳鼻喉科常见疾病的基础理论和基本知识，掌握病史采集、体格检查等临床技能；掌握耳鼻喉科常见疾病的诊断和处理原则；了解耳鼻喉科常用的诊疗技术；培养正确的临床思维。

2.基本要求

（1）症状学

掌握以下常见症状的诊断与鉴别诊断、处理原则：鼻阻塞、鼻音、鼻漏、鼻出血、嗅觉障碍、鼻源性头痛、咽痛、咽感觉异常、声音异常、吞咽困难、饮食反流、耳痛、耳流脓、眩晕、耳鸣、耳聋。

（2）主要疾病

1）鼻外伤及耳鼻喉异物

掌握：鼻外伤及耳鼻喉异物的临床表现、紧急处理原则。

了解：鼻外伤及耳鼻喉异物的进一步处理方法。

2）鼻出血

掌握：鼻出血的紧急处理原则。

熟悉：鼻出血的常见病因。

了解：鼻出血的进一步处理方法。

3）鼻炎、鼻窦炎

掌握：鼻炎、鼻窦炎的临床表现、鉴别诊断及治疗原则。

熟悉：鼻炎、鼻窦炎的特异性治疗方法。

4）扁桃体炎

掌握：急、慢性扁桃体炎及并发症的诊断与鉴别诊断、治疗原则。

熟悉：扁桃体炎的局部治疗方法。

5）突发性耳聋

掌握：突发性耳聋的定义、临床表现、诊断与鉴别诊断、治疗原则。

熟悉：突发性耳聋的病因、诱发因素。

6）中耳炎

掌握：中耳炎常见类型的诊断与鉴别诊断、治疗原则。

熟悉：中耳炎局部治疗方法。

7）腺样体肥大、耳鼻喉常见肿瘤

熟悉：上述疾病的临床表现及处理原则。

耳鼻咽喉科轮转期间学习病种及其例数要求，见表16。

表16　耳鼻咽喉科学习病种和例数要求

病 种 最低例数

鼻外伤及耳鼻喉异物 2

鼻出血 2

鼻炎、鼻窦炎 5

扁桃体炎 5

突发性耳聋 2

中耳炎 5

腺样体肥大、耳鼻喉常见肿瘤 不做具体要求

（3）基本技能要求：见表17。

表17 耳鼻咽喉科基本技能要求

操作技术名称 最低例数

掌握：

外鼻、鼻腔、鼻窦、外耳、鼓膜及咽喉的检查方法 5

鼻镜、耳镜的使用方法 5

了解： 不做具体要求

音叉检查方法、语言测听法

间接喉镜的使用方法

纤维鼻咽镜、鼻内窥镜使用方法

外耳道疖切开术、鼻腔异物、咽异物取出术

（十）传染科 （0.5个月）

1.轮转目的

通过传染科培训，学习传染科常见疾病基础理论和基本知识，掌握病史采集、体格检查、病历书写等临床技能；能够对常见传染科问题做出正确的诊断、评估和转诊；掌握常见传染病的预防原则和方法、法定传染病的报告程序和随访管理。

2.基本要求

（1）主要疾病

1）细菌性痢疾及其他感染性腹泻

掌握：细菌性痢疾及其他感染性腹泻的诊断与鉴别诊断、治疗原则与方法、转诊指征及预防措施。

熟悉：细菌性痢疾及其他感染性腹泻的病因、流行病学特点和发病机制。

2）病毒性肝炎

掌握：肝功能及各种实验室检查的临床意义、诊断及鉴别诊断、预防原则。

熟悉：病毒性肝炎的分型、病原学、传染途径，各型临床表现、治疗原则。

3）结核病

掌握：结核病的诊断与鉴别诊断、治疗原则、常用药物的不良反应及处理方法。

熟悉：预防控制结核病的基本原则、疫情报告与转诊，结核病患者的督导治疗管理。

了解：结核病病因、发病机制和流行趋势。

4）艾滋病

掌握：传播途径、预防原则和治疗方法。

熟悉：咨询检测方法、随访管理。

了解：流行趋势。

5）其他传染病和寄生虫病：包括流行性脑脊髓膜炎、流行性出血热、霍乱、麻风病、常见寄生虫病等。

熟悉：诊断及鉴别诊断、转诊指征、预防与治疗原则。

了解：病因、流行病学特点及发病机制。

（注：在高发病地区根据发病率掌握一定的病例数。非高发地区可通过讲座等形式学习。）

传染科轮转期间学习病种及其例数要求，见表18。

表18　传染科学习病种和例数要求

病 种 最低例数

细菌性痢疾及其他感染性腹泻 2

病毒性肝炎 2

结核病 2

其他常见传染病（包括流行性脑脊髓膜炎、流行性出血热、霍乱、

获得性免疫缺陷综合征、常见寄生虫病、麻风病等） 不做具体要求

（2）其他相关理论与知识

熟悉：常规消毒、隔离方法、自我防护，传染病社区管理与方法。

（十一）精神科 （1个月）

掌握：常见精神症状，如幻觉、妄想、抑郁等；精神分裂症、抑郁症、焦虑症的常见临床表现、检查方法、治疗原则和基本治疗药物，常见药物不良反应的识别与转诊。

熟悉：常见躯体疾病所致精神障碍的临床表现及处理原则；常用筛检量表如抑郁自评量表（SDS）、焦虑自评量表（SAS）的使用指征；社区接诊精神疾病时的注意事项。

了解：酒与药物依赖的识别、处理原则和转诊指征；精神病人的社区家庭康复原则与方法。

（十二）康复医学科（0.5个月）

掌握：脑血管疾病所致功能障碍康复的最佳时间、康复指征、转诊指征。

熟悉：脑血管疾病所致功能障碍、骨关节病、各种常见损伤等疾病的康复评定。

了解：常用物理因子治疗、作业治疗、言语治疗的方法和康复注意事项。

（十三）中医科（0.5个月）

掌握：常用中成药的适应证、常见副作用和使用注意事项。

熟悉：常用中医适宜技术；中医的饮食、养生常识。

了解：中医全科医学概论；中医基础理论在临床实践的运用，中医诊断思维和治疗的基本规律及技巧，临床常见症候的辨证施治方法。

（十四）选修科室（0.5个月）

根据实际需求安排。

（十五）基层实践基地（6个月）

通过在基层医疗卫生服务机构和专业公共卫生机构直接参加全科医疗实践、居民健康管理和公共卫生实践，树立以人为中心、家庭为单位、社区为基础的观念，培养为个体与群体提供连续性、综合性、协调性、人性化服务的能力；基层医疗卫生服务综合管理和团队合作的能力；结合实际工作发现问题、解决问题、开展科研教学工作的能力。

培训形式包括在基层带教医师的指导下从事全科医疗和公共卫生实践；集中授课；案例讨论；教学研讨会；社区卫生调查等。

1.全科医学和社区卫生服务理论

（1）全科/家庭医学的主要概念与原则

掌握：医学模式转变与健康观的理论，全科医学、全科医疗、全科医生概念，全科医疗的基本原则；全科医学对个人、家庭和社区进行综合性、连续性、协调性一体化照顾的理论。

熟悉：全科医生的角色与素质要求、全科医疗与专科医疗的区别和联系。

了解：全科医学的历史起源，全科医学与其他学科的关系，国内外全科医学发展概况，我国发展全科医学的必然性、迫切性、特点与可行途径。

（2）全科医生的临床思维与工作方式

掌握：以人为中心、家庭为单位、社区为基础、预防为导向的基本原则和方法；全生命周期保健原则和内容。

熟悉：生命周期各阶段的主要疾病的社区规范化管理。

了解：生物医学模式的优势与缺陷、全科医疗成本效益原则及其与医疗保障体系的衔接。

（3）常见慢性非传染性疾病健康管理与评价

掌握：慢性非传染性疾病的全科医疗管理技能，包括主要慢性非传染性疾病的常见危险因素及评价，筛检原则与方法；社区为基础的慢性非传染性疾病防治原则、规范化管理与评价。

（4）健康档案

掌握：健康档案的建立和使用。

熟悉：基层医疗卫生服务机构的信息系统及其使用。

（5）健康教育

掌握：健康教育的基本概念，健康教育常用方法及其特点，居民健康教育的计划、实施、评价方法。

（6）社区卫生服务调查的基本技术

掌握：资料收集与整理的基本方法。

熟悉：计数资料和计量资料的分类及其基本统计分析方法；调查报告的撰写及应用。

（7）社区卫生服务管理

掌握：社区卫生服务管理的基本知识和基本概念。

熟悉：常用的质量评价指标和管理原则。

了解：社区卫生人力资源、财务和其他资源管理的基本方法。

2. 全科医疗服务技能

（1）掌握

1）全科医疗接诊方式的特点、通过良好的沟通与居民建立和谐信任关系的技巧。

2）健康档案的书写与使用。培训期间，每人至少完成20份不同健康问题的个人健康档案，并能实行动态管理；家庭健康档案10份，并进行连续管理。

3）常见病的诊断和治疗。急危重症的识别与转诊。慢性非传染性疾病的规范化管理。高血压、冠心病、脑血管病、糖尿病、慢性阻塞性肺疾病、骨质疏松症的临床特点与治疗原则，一、二、三级预防措施及规范管理的基本技术；根据《国家基本公共卫生服务规范》管理高血压和糖尿病。

4）健康教育技能。包括设计计划方案，实施教育、咨询、评价等活动；在培训期间参与5个以上健康问题的健康教育，独立完成5次以上健康教育，时间不少于1小时，听众不少于15人。

5）家庭访视与家庭病床管理。培训期间，每位全科住院医师至少与5个家庭建立长期联系，该家庭中应包括老人、儿童、残疾人、妇女等四类人中的两类人，对家庭进行评估并实施以家庭为单位的照顾。

6）社区卫生服务调查与评估。参与社区卫生诊断，建立合理利用社区资源的意识，结业时写出有针对性的调查报告。

7）国家基本药物的用法、用量、常见的不良反应、药物的相互作用以及使用注意事项。

（2）熟悉

1）常见慢性非传染性疾病的康复指征。

2）肿瘤、帕金森病、老年期痴呆、睡眠呼吸暂停综合征等疾病的临床特点、治疗原则、预防措施及社区管理。

3）焦虑症、抑郁症的诊断与处理，常见身心疾患的识别与处理；社区常见心理问题及睡眠障碍的问诊技术及处理原则。

（3）了解

1）常用药物的储存方法。

2）膳食及疾病对药物作用的影响。

3）应用卫生经济学的基本知识为病人合理用药。

全科医疗服务主要技能要求，见表19。

表19　全科医疗服务主要技能要求

操作技术名称 最低例数

健康档案的书写与使用 20

健康教育 5

家庭访视 5

规范管理高血压 10

规范管理糖尿病 10

管理家庭病床 2

3. 基本公共卫生服务技能

掌握《国家基本公共卫生服务规范》的主要内容：城乡居民健康档案管理；健康教育；预防接种； 0至6岁儿童健康管理；孕产妇健康管理；老年人健康管理；高血压患者健康管理；2型糖尿病患者健康管理；重性精神疾病患者管理；传染病及突发公共卫生事件的报告和处理；卫生监督协管。具体要求如下：

（1）特殊人群保健

1）儿童保健

掌握：

①新生儿访视的内容和技巧；儿童体格检查操作技术（测量身高、体重、头围、胸围等），并能针对体检结果作出恰当的评价和指导。

②婴儿喂养指导和儿童营养咨询技术，辅助食品添加的顺序及原则。

③有关视力及听力筛查、口腔问题的健康教育。

④国家免疫规划疫苗免疫程序和其他预防接种方法，注意事项以及不良反应处理。

⑤儿童系统管理方法。完成新生儿访视、完成儿童智力发育测量、跟随指导医师完成儿童预防接种等工作。

熟悉：各年龄儿童保健原则、具体措施以及小儿保健组织机构；学校卫生、安全教育、性教育内容；冷链管理；儿童智力发育测量（DDST）及评价。

2）老年人保健

掌握：老年人健康综合评估的内容和方法；健康生活方式指导。

熟悉：影响老年人功能减退的因素及其预防措施；运动锻炼的积极作用与方式；老年家庭安全问题与老年营养的要求；生命质量的内涵、概念、测定方法。

了解：健康预期寿命的内涵和表达方法；老年人常见负性情绪及其表现特点；老年患者的心理问题及处理；老年口腔问题的健康教育；社区老年人分级护理概念及护理需求的评估；临终关怀的概念、镇痛、心理与社会方面的照顾原则。

3）妇女保健

熟悉：经期卫生及劳动保护；婚前检查的重要性及计划生育指导；孕期饮食、营养、起居环境、性生活、胎动自我监测和乳房护理；产褥期的产后访视、产褥期卫生、乳房护理及母乳喂养的有关知识；产后抑郁症筛检；围绝经期综合征的预防与诊治、激素替代疗法的适应证；妇科常见疾病（宫颈癌、乳腺癌）普查的意义和方法；孕产妇常见口腔问题的健康教育。

4）残疾人保健

熟悉：国家有关残疾人权益的政策、法规，社区康复的组织与实施。

了解：残疾人心理特点及其需求、咨询与康复指导。

（2）传染病管理

掌握：法定传染病的报告和处理方法、传播途径及预防原则；计划免疫程序、安全性与有效性。

熟悉：常见传染病的检测方法和社区用药原则，传染病人的社区随访管理方法。

（3）精神疾病管理

熟悉：重性精神病患者管理服务规范，包括随访内容、转诊原则及标准、监护人管理、社区及家庭康复的原则及方法。

（4）突发公共卫生事件

掌握：突发公共卫生事件的报告程序、防疫原则。

熟悉：突发公共卫生事件的应急措施、处理原则。

（5）卫生监督协管

熟悉：《卫生监督协管服务规范》的内容。

基本公共卫生服务主要技能要求，见表20。

表20 基本公共卫生服务主要技能要求

操作技术名称 最低例数

新生儿访视 5

儿童智力发育测查 5

儿童预防接种 10

老年人健康综合评估 10

4．基层医疗卫生服务机构管理技能了解：

（1）本地区和本机构卫生服务信息系统的内容及管理程序。

（2）基层医疗卫生服务团队合作的服务模式，团队建设的意义和方法。

（3）人际沟通技巧，包括与基层管理者、社会工作者等沟通的技巧。

四、培养考核

学员培训结束时，过程考核合格者需参加省级卫生行政部门统一组织的结业考核，完成全程培训，各项考试、考核合格者，由省级卫生行政部门颁发卫生部统一印制的相应合格证书。

附表

有关医疗卫生法律法规及重要文件推荐目录

《执业医师法》

《药品管理法》

《国家基本药物处方集》 《国家基本药物临床应用指南》

《处方管理办法》

《侵权责任法》

《医疗事故处理条例》

《医疗机构管理条例》

《国家基本公共卫生服务规范》 《传染病防治法》

《突发公共卫生事件应急条例》

《放射诊疗管理规定》

《食品安全法》

《食品安全法实施条例》

《职业病防治法》

《国家职业病防治规划（2009-2015年）》

《尘肺病防治条例》

《职业病诊断与鉴定管理办法》

《职业健康监护管理办法》

《国家职业卫生标准管理办法》

《献血法》

《血液制品管理条例》

《血站管理办法》

《抗菌药物临床应用管理办法》

《卫生监督协管服务规范》

《病历书写基本规范》

《医疗废物管理条例》

《医疗技术临床应用管理办法》

《医疗卫生机构医疗废物管理办法》

《抗菌药物临床应用指导原则》

《医院感染管理办法》

《医疗废物管理条例》

《血吸虫病防治条例》

《疫苗流通和预防接种管理条例》

《艾滋病防治条例》
