# Supplementary material for: Mapping the evolution of fertility support policies in China: A content and instrumental analysis
Source: PLoS One. 2025 Oct 9;20(10):e0332137. doi: 10.1371/journal.pone.0332137 (PMC12510515; doi:10.1371/journal.pone.0332137)
Supplement: S1 Appendix — (ZIP) [file pone.0332137.s001.zip › S1 Appendix. 226 original policy documents/146-中国工运事业和工会工作“十四五”发展规划(FBM-CLI-5-5016911).docx]

中国工运事业和工会工作“十四五”发展规划

发布部门： 中华全国总工会

发布日期：2021.07.16

实施日期：2021.07.16

时效性： 现行有效

效力级别： 团体规定

法规类别： 计划综合规定

中国工运事业和工会工作“十四五”发展规划

（中华全国总工会 2021年7月16日）

目　录

一、开创中国工运事业和工会工作新局面

1.党的十八大以来中国工运事业和工会工作蓬勃发展。

2.“十四五”时期中国工运事业和工会工作面临新形势新任务新要求。

--进入新发展阶段工会面临新形势。

--贯彻新发展理念工会面临新任务。

--构建新发展格局工会面临新要求。

二、“十四五”时期中国工运事业和工会工作的总体要求

3.指导思想。

4.基本原则。

--坚持党的领导。

--坚持正确方向。

--坚持服务大局。

--坚持职工为本。

--坚持改革创新。

--坚持法治保障。

5.主要目标。

--工会理论武装得到新加强。

--职工思想引领取得新进展。

--职工建功立业展现新作为。

--维护职工权益取得新实效。

--服务职工水平实现新提升。

--工会组织建设呈现新活力。

三、加强职工思想政治引领，团结引导职工坚定不移听党话、跟党走

6.以习近平新时代中国特色社会主义思想武装职工。

7.以理想信念教育职工。

8.以社会主义核心价值观引领职工。

9.以先进职工文化感染职工。

四、深化产业工人队伍建设改革，在推动高质量发展中充分发挥工人阶级主力军作用

10.促进产业工人队伍建设改革走深走实。

11.推动构建产业工人全面发展制度体系。

12.广泛深入持久开展劳动和技能竞赛。

13.大力弘扬劳模精神、劳动精神、工匠精神。

五、高举维护职工合法权益旗帜，增强职工群众获得感幸福感安全感

14.维护职工劳动经济权益。

15.维护职工民主政治权利。

16.维护新就业形态劳动者合法权益。

17.做好农民工维权服务工作。

18.提升女职工维权服务水平。

六、建立健全高标准职工服务体系，不断提升职工生活品质

19.加强服务阵地建设。

20.健全困难职工家庭常态化帮扶机制。

21.实施提升职工生活品质行动。

22.打造服务职工系列品牌。

七、构建和谐劳动关系，推动共建共治共享社会治理

23.加大劳动法律法规源头参与力度。

24.推动完善构建和谐劳动关系制度机制。

25.推进工会工作法治化建设。

26.健全落实“五个坚决”要求的长效机制。

八、加快智慧工会建设，打造工会工作升级版

27.构建基于大数据技术的工会治理能力提升体系。

28.构建基于互联网技术的工会服务应用创新体系。

29.构建基于云计算技术的工会网信基础支撑体系。

30.巩固发展工会网上舆论阵地。

九、深化工会和职工对外交流交往合作，为推动构建人类命运共同体作贡献

31.拓展工会和职工国际交流交往合作的深度和广度。

32.加强与港澳台工会组织和劳动界交流合作。

十、深化工会改革创新，推动新时代工会工作高质量发展

33.系统谋划推进工会改革。

34.健全工会工作制度机制。

35.激发基层工会活力。

36.改进完善工会组织体系。

37.充分发挥产业工会作用。

38.深化财务管理改革。

39.加强工会经费审查审计监督。

40.提高工会资产管理效能。

十一、坚持以党的政治建设为统领，提高工会工作能力和水平

41.全面加强工会系统党的建设。

42.深化工会干部队伍建设。

43.不断拓宽工会理论研究新路子。

十二、加强规划落实的组织保障

44.加强组织领导。

45.加强支撑保障。

46.加强总结推广。

“十四五”时期是我国全面建成小康社会、实现第一个百年奋斗目标之后，乘势而上开启全面建设社会主义现代化国家新征程、向第二个百年奋斗目标进军的第一个五年，是中国工运事业和工会工作围绕中心、服务大局，立足新发展阶段、贯彻新发展理念、推动构建新发展格局，履行职责使命，实现高质量发展的五年。党的十九届五中全会审议通过的《中共中央关于制定国民经济和社会发展第十四个五年规划和二〇三五年远景目标的建议》和十三届全国人民代表大会第四次会议审查批准的《中华人民共和国国民经济和社会发展第十四个五年规划和2035年远景目标纲要》擘画了我国未来5年和15年发展的宏伟蓝图。实现这一奋斗目标，工人阶级使命光荣，工会组织责任重大。为充分发挥工会组织作用，团结动员亿万职工为全面建设社会主义现代化国家、实现中华民族伟大复兴的中国梦贡献智慧和力量，特制定本规划。

一、开创中国工运事业和工会工作新局面

1.党的十八大以来中国工运事业和工会工作蓬勃发展。

在以习近平同志为核心的党中央坚强领导下，我国工人阶级以高度的主人翁使命感和历史责任感，积极投身进行伟大斗争、建设伟大工程、推进伟大事业、实现伟大梦想的火热实践，推动党和国家事业取得决定性成就、发生历史性变革。各级工会坚持以习近平新时代中国特色社会主义思想为指导，学习贯彻习近平总书记关于工人阶级和工会工作的重要论述，以保持和增强工会组织和工会工作政治性、先进性、群众性为主线，忠诚履职、积极作为，各项工作取得了显著成效。思想政治引领明显加强，职工团结奋斗的思想基础更加巩固；劳模精神、劳动精神、工匠精神有力弘扬，工人阶级主力军作用充分发挥；维权服务力度不断加大，职工群众获得感、幸福感、安全感不断提升；产业工人队伍建设改革扎实推进，产业工人地位作用更加彰显；工会改革创新持续深化，工会组织吸引力凝聚力战斗力切实增强；工会系统党的建设全面加强，风清气正的政治生态进一步形成。这些成绩的取得，是在党的领导下各级工会组织与广大职工努力奋斗的结果，为“十四五”时期工运事业和工会工作发展奠定了坚实基础。

2.“十四五”时期中国工运事业和工会工作面临新形势新任务新要求。

--进入新发展阶段工会面临新形势。新发展阶段是我们党带领人民迎来从站起来、富起来到强起来历史性跨越的新阶段，是我国社会主义发展进程中的一个重要阶段。我国发展的内部条件和外部环境发生深刻复杂变化。当今世界正经历百年未有之大变局，新一轮科技革命和产业变革深入发展，新冠肺炎疫情影响广泛深远，经济全球化遭遇逆流。我国已转向高质量发展阶段，既具有制度优势显著、治理效能提升、经济长期向好等优势和条件，同时又面临发展不平衡不充分问题仍然突出、重点领域关键环节改革任务仍然艰巨、创新能力不适应高质量发展要求等问题。面对复杂多变的国际国内形势，工会面临的机遇和挑战都前所未有。如何把握“两个大局”，心怀“国之大者”，在纷繁复杂的国际局势中保持清醒、坚守定力，在艰巨繁重的改革发展稳定任务中实现好维护好发展好广大职工合法权益，团结动员广大职工为促进高质量发展贡献智慧和力量，为全面建设社会主义现代化国家开好局起好步建功立业，成为摆在各级工会面前的重大课题。

--贯彻新发展理念工会面临新任务。党的十九届五中全会强调要坚定不移贯彻新发展理念，将新发展理念贯穿“十四五”规划和2035年远景目标的全过程和全领域。各级工会组织必须适应职工队伍规模结构、就业方式、分配方式、利益诉求、思想观念的深刻变化，适应新技术新业态新模式背景下劳动关系的深刻调整，提高贯彻新发展理念的思想自觉和行动自觉。贯彻创新发展理念，要求工会必须尊重基层和职工群众的首创精神，把蕴藏在职工群众中的创造活力激发出来；推进工会自身改革，切实解决工会组织体制机制不够完善、工作载体手段不够丰富、服务群众工作本领有待增强等问题。贯彻协调发展理念，要求工会必须树立全国工会“一盘棋”理念，既全面推进、又突出重点，加强分类指导，解决好发展不平衡的问题，增进工作的系统性、整体性、协同性。贯彻绿色发展理念，要求工会把绿色发展理念融入职工的生产生活实践，引导广大职工践行绿色生产生活方式。贯彻开放发展理念，要求工会坚持开门办会，让职工群众充分参与到工会工作中来，积极运用社会资源和力量推动工会工作；加大中国工会和职工对外交流交往力度，有力服务国家总体外交。贯彻共享发展理念，要求工会必须贯彻以人民为中心的发展思想，切实履行维护职工合法权益、竭诚服务职工群众的基本职责，让改革发展成果更多更公平惠及职工群众，在推动实现共同富裕中展现工会作为。

--构建新发展格局工会面临新要求。新发展格局是以习近平同志为核心的党中央积极应对国际国内形势变化、与时俱进提升我国经济发展水平、塑造国际经济合作和竞争新优势而作出的战略抉择。构建以国内大循环为主体、国内国际双循环相互促进的新发展格局，需要工会深刻认识国际国内复杂形势变化，特别是中美经贸摩擦、供给侧结构性改革等对职工队伍和工会工作带来的影响，立足国内办好自己的事情，找准结合点、切入点、着力点，发挥政治优势、组织优势、制度优势、群众优势、资源优势，将职工的思想凝聚到促进高质量发展上来，将职工的力量汇聚到建功立业上来；围绕扩大内需这个战略基点，积极加强就业创业服务，推动构建收入分配新格局、完善社会保障体系，在发展基础上努力提高职工收入水平，提高消费意愿和能力，在满足职工美好生活需要的同时，为扩大内需、促进双循环特别是国内经济大循环奠定厚实基础；围绕创新驱动这个关键所在，以深化产业工人队伍建设改革为抓手增强发展的内生动力，瞄准提升产业基础高级化、产业链现代化水平等目标，持续提升产业工人队伍素质、激发创新创造活力，在关键核心技术攻关、解决“卡脖子”等问题上发挥作用，推动实现高水平科技自立自强，使产业工人成为支撑中国制造、中国创造的重要力量。

二、“十四五”时期中国工运事业和工会工作的总体要求

3.指导思想。

以习近平新时代中国特色社会主义思想为指导，全面贯彻党的十九大和十九届二中、三中、四中、五中全会精神，学习贯彻习近平总书记关于工人阶级和工会工作的重要论述，增强“四个意识”、坚定“四个自信”、做到“两个维护”，围绕把握新发展阶段、贯彻新发展理念、构建新发展格局、推动高质量发展，坚持稳中求进工作总基调，牢牢把握为实现中华民族伟大复兴中国梦而奋斗的工运时代主题，坚定不移走中国特色社会主义工会发展道路，以保持和增强工会组织和工会工作政治性、先进性、群众性为主线，以产业工人队伍建设改革和工会改革为动力，以推动工会工作高质量发展为着力点，使职工的理想信念更加坚定，权益保障更加充分，劳动关系更加和谐，党执政的阶级基础和群众基础更加牢固，广大职工在全面建设社会主义现代化国家开好局、起好步中主力军作用更加彰显。

4.基本原则。

--坚持党的领导。将自觉接受党的领导作为工会根本政治原则，把党的政治建设摆在首位，全面贯彻党的基本理论、基本路线、基本方略，不折不扣将党中央决策部署贯彻到工会各项工作中去，将党的意志主张落实到广大职工中去，充分发挥党联系职工群众的桥梁纽带作用，团结引导广大职工坚定不移听党话、矢志不渝跟党走，始终做党执政的坚实依靠力量。

--坚持正确方向。持之以恒以党的创新理论武装头脑、指导实践、推动工作，不断提高政治判断力、政治领悟力、政治执行力，始终在政治立场、政治方向、政治原则、政治道路上同以习近平同志为核心的党中央保持高度一致。

--坚持服务大局。围绕党和国家工作大局，谋划和推进工会工作，坚持在大局下思考、大局下行动，组织动员广大职工充分发挥工人阶级主力军作用，以满腔热情投身全面建设社会主义现代化国家的伟大实践。

--坚持职工为本。牢固树立以职工为中心的工作导向，把联系和服务职工作为工会工作的生命线，扎实履行维护职工合法权益、竭诚服务职工群众的基本职责，不断提升职工群众的获得感、幸福感、安全感，推动实现共同富裕。

--坚持改革创新。系统谋划和扎实推进工会改革，坚持系统观念，增强统筹意识，发挥改革的突破性和引导性作用，着力破除制约工会高质量发展、影响职工高品质生活的体制机制障碍，固根基、扬优势、补短板、强弱项，不断推动工会理论创新、体制创新、工作创新，把改革创新贯穿于工会工作全过程和各方面。

--坚持法治保障。按照全面推进依法治国总目标要求，自觉把工会工作置于法治国家、法治政府、法治社会建设全局中谋划和推进，坚持依法建会、依法管会、依法履职、依法维权，不断提升工会法治化建设水平，推动国家治理体系和治理能力现代化。

5.主要目标。

今后5年，工运事业和工会工作发展要坚持目标导向和问题导向相结合，坚持守正和创新相统一，努力实现以下主要目标：

--工会理论武装得到新加强。习近平新时代中国特色社会主义思想更加深入人心，学习贯彻习近平总书记关于工人阶级和工会工作的重要论述取得重要理论成果和实践成效，运用马克思主义立场、观点、方法解决实际问题的能力切实加强。

--职工思想引领取得新进展。面向职工群众的理论宣讲形成制度性安排，党的创新理论不断走近职工身边、走进职工心里；理想信念教育常态化开展、制度化推进，“中国梦·劳动美”主题宣传教育活动更加丰富，广大职工在理想信念、价值理念、道德观念上紧紧团结在一起，对中国特色社会主义的道路自信、理论自信、制度自信、文化自信不断增强。

--职工建功立业展现新作为。广大职工主人翁意识进一步增强，劳模精神、劳动精神、工匠精神大力弘扬，劳动和技能竞赛广泛深入持久开展，群众性创新活动成果显著；产业工人队伍建设改革取得突破性进展，在推动高质量发展中的工人阶级主力军作用充分彰显。

--维护职工权益取得新实效。劳动法律法规体系不断完善，职工合法权益维护机制不断健全，新就业形态劳动者建会入会和权益维护形成制度保障，劳动关系协调机制有效运行，工会参与劳动争议预防调处化解的水平不断提升，维护劳动领域安全稳定体系和能力建设有效推进，在助推建设更高水平的平安中国中作用积极发挥。

--服务职工水平实现新提升。联系服务职工长效机制建立健全，工会服务阵地建设明显加强，服务职工“最后一公里”问题有效解决，困难职工家庭常态化帮扶机制有效运行，工会服务职工品牌项目叫响做实。

--工会组织建设呈现新活力。工会改革创新持续深化，联系广泛、服务职工的工会工作体系日益健全，智慧工会建设取得实质性进展，基层工会组织设置、运行机制进一步健全，基层基础薄弱问题得到有效解决，工会组织覆盖面不断扩大、凝聚力进一步增强。

三、加强职工思想政治引领，团结引导职工坚定不移听党话、跟党走

6.以习近平新时代中国特色社会主义思想武装职工。

建立健全职工思想政治工作的领导体制和工作机制，完善党的创新理论和工会理论下基层长效机制，落实基层联系点、送教到基层等制度，建立健全企业班组常态化学习制度，组织专家、学者、先进人物等广泛开展有特色、接地气、入人心的宣传宣讲活动，推动习近平新时代中国特色社会主义思想进企业、进车间、进学校、进教材、进头脑，打牢广大职工团结奋斗的思想基础。

7.以理想信念教育职工。

深化中国特色社会主义和中国梦宣传教育，加强爱国主义、集体主义、社会主义教育，弘扬党和人民在各个历史时期奋斗中形成的伟大精神，深入开展“永远跟党走”、“党旗在基层一线高高飘扬”等系列主题宣传教育活动，在广大职工中唱响共产党好、社会主义好、改革开放好、伟大祖国好、各族人民好的时代主旋律。广泛开展党史学习教育，高质量完成学习教育各项任务，引领广大职工学史明理、学史增信、学史崇德、学史力行。深入开展党史、新中国史、改革开放史、社会主义发展史宣传教育，引导广大职工群众深刻认识中国共产党为什么能、马克思主义为什么行、中国特色社会主义为什么好，增强听党话、跟党走的思想自觉和行动自觉。围绕2025年全总成立100周年，组织召开系列庆祝活动；推动建立中国工运史馆，探索筹建国家劳模风采展示馆或博物馆，加强对红色工运的重要人物、重要遗址（旧址）、重大事件、重点纪念场馆等的梳理发掘、修建修缮、展示展陈等综合性保护、修复、开发工作；组织开展百年中国工运史宣传教育，向全社会广泛宣传工人阶级和工人运动的光荣历史、奋斗历程、辉煌成就；组织开展百年中国工运史系列研究。推动理想信念教育常态化制度化，通过劳模宣讲、演讲比赛、知识竞赛、读书诵读等方式，运用“学习强国”、职工书屋等学习平台，引导广大职工紧跟共产党、奋进新时代。

8.以社会主义核心价值观引领职工。

坚持把社会主义核心价值观融入职工生产生活，内化为职工的情感认同和行为习惯。深入开展以劳动创造幸福为主题的宣传教育，推动建立健全新时代劳动教育理论和实践体系。深化以职业道德为重点的社会公德、职业道德、家庭美德、个人品德等“四德”建设，组织开展全国职工职业道德建设评选表彰。积极参与群众性精神文明创建活动，推进家庭、家教、家风建设，广泛开展学雷锋志愿活动，展示新时代职工文明形象。

9.以先进职工文化感染职工。

推动建立健全党委领导、行政支持、工会运作、职工参与的职工文化共建共享机制。丰富职工文化产品供给。打造“中国梦·劳动美”系列职工文化品牌，每年举办“中国梦·劳动美”--庆祝“五一”国际劳动节特别节目，广泛组织开展职工运动会、职工文艺展演、职工艺术节等全国性、区域性、行业性职工文体活动。加强职工文化阵地建设。探索建立以全总文工团为主体的职工艺术阵地联盟，整合工人文化宫、职工艺术院团资源，推动在街道社区、产业园区、商圈楼宇等职工聚集区建设职工文化场馆，构建立体化、多元化职工文化服务网络。建好、管好、用好职工书屋，力争到2025年底全国工会职工书屋示范点达到1.6万家，带动各级工会自建职工书屋达到15万家，实现各类便利型阅读点、劳模工匠书架广泛覆盖；电子职工书屋覆盖职工逾5000万人，基本形成覆盖大多数职工的工会阅读推广服务体系。创新文化服务方式。搭建“互联网＋职工文化”平台，推动职工文化网络化传播，为职工提供“菜单式”、“订单式”文化服务；持续开展“阅读经典好书　 争当时代工匠”、“玫瑰书香”等主题阅读活动。加强职工文化人才队伍建设。打造一支专业化、社团化、志愿化相结合的职工文化人才队伍，培育一批德艺双馨、具有一定社会影响力的职工文化建设领军人才，创作一批思想性强、艺术性高、社会影响大、群众口碑好的精品力作。

四、深化产业工人队伍建设改革，在推动高质量发展中充分发挥工人阶级主力军作用

10.促进产业工人队伍建设改革走深走实。

按照政治上保证、制度上落实、素质上提高、权益上维护的总体思路，围绕造就一支有理想守信念、懂技术会创新、敢担当讲奉献的宏大的产业工人队伍，聚焦产业工人思想引领、建功立业、素质提升、地位提高、队伍壮大等重点任务，总结推进产业工人队伍建设改革以来取得的经验，查找存在的问题与不足，推动产业工人队伍建设改革向纵深发展、向基层延伸。坚持党委统一领导，政府有关部门各司其职，工会、行业协会、企业代表组织充分发挥作用，统筹社会组织的协同力量，完善合力推进产业工人队伍建设改革的工作格局。充分发挥产业工人队伍建设改革协调小组作用，强化贯彻落实协调机制，履行工会宏观指导、政策协调、组织推进、督促检查的职责，每年制定产业工人队伍建设改革要点，压实部门责任，强化分类指导，增强改革的系统性、整体性、协同性。健全产业工人队伍建设改革情况监督检查和信息反馈制度，推动各地将产业工人队伍建设改革纳入各级党委和政府目标考核体系，建立党委和政府联合督查督办工作机制。建立产业工人队伍建设改革效能评估机制，开展改革情况绩效评估，探索实行第三方评估，确保改革举措落地见效。探索建立企业主体作用发挥机制，保护企业人才培养积极性。鼓励各地、各相关责任单位因地制宜大胆探索试点，形成一批具有部门、地方、产业和企业特色的改革成果。

11.推动构建产业工人全面发展制度体系。

强化系统集成，在系统梳理整合现有政策制度基础上，突出补齐制度缺项和政策短板，推动形成系统完备、科学规范、运行高效的制度体系，着力提升改革的政策效能。健全保障产业工人主人翁地位制度体系，完善产业工人参政议政制度，提高产业工人在各级党组织、人大、政协、群团组织代表大会代表和委员会委员中的比例；探索实行产业工人在群团组织挂职和兼职制度。健全产业工人技能形成制度体系，重点推动完善现代职业教育制度、职工技能培训制度、高技能人才培养机制、“互联网＋”培训机制等，畅通技术工人成长成才通道；实施高技能领军人才和优秀产业技术紧缺人才境外培训计划；构建“互联网＋职工素质建设工程”模式，完善中国职工经济技术信息化服务平台，做大做精做强全国产业工人学习社区，加强“技能强国--全国产业工人技能学习平台”建设，推进技能实训基地建设，拓展工会职业培训空间。健全产业工人发展制度体系，推动完善职业技能评价制度、体现技能价值激励导向的工资分配制度、个人学习账号和学分累计制度等，促进学历、非学历教育与职业培训衔接互认，搭建产业工人成长平台。健全产业工人队伍建设支撑保障制度体系，推动完善财政和社会多元投入机制，发挥工会职工创新补助资金作用，加大对产业工人创新创效扶持力度。

12.广泛深入持久开展劳动和技能竞赛。

制定并落实“十四五”劳动和技能竞赛规划，推动建立健全职工劳动和技能竞赛体系。围绕国家重大战略、重大工程、重大项目、重点产业，广泛深入持久开展“建功‘十四五’、奋进新征程”主题劳动和技能竞赛。聚焦推动西部大开发形成新格局、推动东北振兴取得新突破、推动中部地区高质量发展战略，以及推进京津冀协同发展、长江经济带发展、粤港澳大湾区建设、长三角一体化发展、成渝地区双城经济圈、黄河流域生态保护等开展区域性劳动和技能竞赛，搭建交流合作平台，助力区域协调发展；按照国家碳达峰、碳中和部署，聚焦推动绿色发展，组织职工节能减排竞赛，推进重点行业和重要领域绿色化改造。以技术创新为导向，创新竞赛方式和载体，发挥网络平台作用，增强活动的便利性和群众性；加强非公企业劳动和技能竞赛工作，探索新产业新业态开展竞赛的新形式。积极推动将新职业新工种纳入职业分类大典，加强对全国职工职业技能竞赛的规划和指导，联合人力资源社会保障部等部门定期举办全国职工职业技能大赛，与有关部门共同主办国家级一类、二类等职业技能竞赛，指导带动各地层层开展技能比赛，打造职工技能竞赛品牌。组织职工积极参加技术革新、技术协作、发明创造、合理化建议、网上练兵和“小发明、小创造、小革新、小设计、小建议”等群众性创新活动。

13.大力弘扬劳模精神、劳动精神、工匠精神。

学习贯彻习近平总书记在全国劳动模范和先进工作者表彰大会上重要讲话精神，加大对劳动模范和先进工作者的宣传力度，讲好劳模故事、讲好劳动故事、讲好工匠故事，营造劳动光荣的社会风尚和精益求精的敬业风气。进一步做好劳模培养选树和管理服务工作，完善全国工会劳模工作管理平台，推动完善劳模政策，提升劳模地位，落实劳模待遇，形成尊重劳动、尊重知识、尊重人才、尊重创造良好氛围。做好劳模和五一劳动奖、工人先锋号等评选表彰工作，持续开展“最美职工”、“大国工匠”等主题宣传，“十四五”期间重点选树宣传100名左右的劳模工匠先进典型，加快培育、选树一批在全国有影响力、在行业有号召力的领军型劳模，打造新时代劳动者的标杆旗帜。加大劳模教育培养力度，鼓励各级工会开展劳模教育培训，叫响做实由劳模学员、劳模辅导员、劳模学院、劳模宣讲团等构成的“劳模＋”品牌。用好全国劳模专项补助资金，深入开展劳模定期走访慰问、及时帮扶救助、开展健康体检和疗休养等工作。深化劳模和工匠人才创新工作室创建工作，加强分级分类管理，形成以全国示范性创新工作室为引领、以省市级创新工作室为主体、基层创新工作室蓬勃发展的工作体系，确保到2025年底全国示范性劳模和工匠人才创新工作室达到500家左右，各级各类创新工作室达到15万家。规范和推广“港口工匠创新联盟”等做法，探索创建跨区域、跨行业、跨企业的创新工作室联盟，指导开展创新工作室联盟试点。深化新时代工匠学院建设。统筹各地工匠人才选树、命名、宣传，推动设立国家级大国工匠评选表彰奖项。开展创新工作室领衔人培训、交流等活动，积极组织推荐创新工作室的成果和专利参加各类奖项评选和展示交流。加强劳模和工匠人才创新工作室信息化管理，进一步完善创新工作室网络工作平台。举办大国工匠创新交流大会、职工创新创业博览会。探索全国职工技能成果转化工作，指导各地做好先行先试工作。深入开展“大国工匠进校园”、“劳模进校园”、“奋斗的我·最美的国”新时代先进人物进校园活动。

五、高举维护职工合法权益旗帜，增强职工群众获得感幸福感安全感

14.维护职工劳动经济权益。

高度关注深化供给侧结构性改革，实现碳达峰、碳中和目标中的产业结构转型、绿色转型等对就业结构、就业方式等带来的影响，加大对职工就业、收入分配、社会保障、劳动安全卫生等权益的维护力度。积极推动落实就业优先政策，参与就业创业政策制定，深化工会就业创业服务，广泛开展工会就业创业系列服务活动月以及“京津冀蒙跨区域招聘”、“阳光就业暖心行动”等活动，加强“工E就业”、“工会就业服务号”等全国工会就业服务网上平台建设，推动工会网上就业服务体系化建设。积极开展就业技能培训，深入推进以训稳岗；鼓励引导各地工会开展家政服务人员培训，年均培训达到20万人次，每年至少推树30名“最美家政人”。推动劳务派遣用工依法规范，促进共享用工规范有序。推动各地合理调整最低工资标准。指导企业依法开展工资集体协商，促进企业健全反映劳动力市场供求关系和企业经济效益的工资决定和合理增长机制；总结指导企业技能要素参与分配的经验做法，推动提高技术工人待遇政策的落实。推动完善职工社会保险制度和分层分类社会救助体系，健全覆盖全民、统筹城乡、公平统一、可持续的多层次社会保障体系。做好工会劳动保护工作，加强对职工安全生产和职业健康知识的教育培训，提高职工事故防范、应急处置和自我保护能力；在重点行业领域探索开展职工安全技能竞赛，深化“安康杯”竞赛等群众性安全生产和职业健康活动。发挥工会劳动保护监督检查作用，督促企业落实安全生产和职业病防治主体责任。积极参加国家安全生产工作巡查、督查、考核和生产安全事故调查处理工作，维护好伤亡职工的合法权益。在重点行业中推行劳动安全卫生专项集体合同制度。

15.维护职工民主政治权利。

推动企业民主管理立法和有关政策的制定完善，创新民主管理实践形式，深化民主管理载体建设。推动健全省级厂务公开协调领导机构。进一步健全以职工代表大会为基本形式的企事业单位民主管理制度体系，加强职工代表大会、厂务公开以及职工董事职工监事的制度衔接，促进职代会与集体协商、工会劳动法律监督、法律援助等有机结合，融入企业内部自主调处、群体性劳动关系矛盾快速处置机制。聚焦国企改革三年行动计划落实，推进企业集团职代会制度建设，推动将职工代表大会等企业民主管理纳入公司章程，融入企业治理结构和管理体系，探索中国特色现代企业制度下的民主管理实现途径。深化创新区域（行业）职工代表大会制度，强化分类指导，积极扩大民主管理工作对中小微企业的有效覆盖。制定企业民主管理程序指引或操作指南。坚持每年开展企业民主管理师资培训。深入开展“聚合力、促发展”职工代表优秀提案征集推荐活动、全国厂务公开民主管理评选表彰活动。

16.维护新就业形态劳动者合法权益。

配合人社部门研究制定维护新就业形态劳动者劳动保障权益政策。积极推动新就业形态劳动者参加社会保险制度，推动研究出台新就业形态劳动者职业伤害保障办法等相关政策措施。推动灵活用工集中的行业制定劳动定额指导标准。加强平台网约劳动者收入保障，推动平台企业、关联企业与劳动者就劳动报酬、支付周期、休息休假和职业安全保障等事项开展协商。推动平台网约劳动者民主参与，督促平台运营企业建立争议处理、投诉机制。指导推动快递、外卖、网约出行、网约货运、家政、保洁等灵活就业人员较多的行业建立、完善劳动者权益保障机制，加强对平台网约劳动者的法律援助和生活服务。积极参与国家企业社会责任制度建设，推动落实企业社会责任。加强对各类社会组织和新阶层新群体的主动关注、积极联系、有效覆盖。

17.做好农民工维权服务工作。

建立健全工会系统欠薪报告制度和欠薪案件反馈督办机制，推动解决拖欠农民工工资问题，深入实施农民工学历与能力提升行动计划，深化农民工“求学圆梦行动”，设立专项扶持资金，每年帮助30万名职工特别是农民工提升学历水平。深入开展“尊法守法·携手筑梦”服务农民工公益法律服务行动，健全农民工法律援助服务网络，开辟农民工劳动争议案件“绿色通道”。创新农民工组织形式和入会方式，逐步建立城乡一体的农民工流动会员管理制度，提高农民工入会的积极性和主动性。推进农民工平等享受城镇基本公共服务。

18.提升女职工维权服务水平。

积极参与性别平等和女职工权益保障法律法规政策制定修订，推动用人单位建立健全工作场所性别平等制度机制，推行女职工权益保护专项集体合同，促进家庭友好型工作场所建设，帮助职工平衡工作与家庭。强化监督维权，协调推动侵害女职工权益案件调查处理；组织开展女职工维权行动月活动，深化普法宣传到基层活动。实施“女职工关爱行动”，管好用好“关爱女职工专项基金”，做好女职工“两癌”检查、女职工休息哺乳室建设、工会爱心托管服务、“会聚良缘”工会婚恋服务等工作。加强对适婚职工的婚恋观、家庭观教育引导，重视和做好应对人口老龄化国家战略、实施三孩生育政策中女职工就业、生育保险、休息休假等权益维护工作。

六、建立健全高标准职工服务体系，不断提升职工生活品质

19.加强服务阵地建设。

推进“会、站、家”一体化建设，加强枢纽型社会组织平台功能建设。培育壮大基层工会服务阵地，拓展服务项目，整合社会资源，推动开放共享，实现区域内职工活动与服务基本覆盖。按照“突出公益、聚焦主业、自主经营、依法监管”的工作要求，更好发挥工人疗休养院、工人文化宫、职工互助保障组织等服务职工的作用。加强工人文化宫规范化建设管理，“十四五”期间，全国建设100家标准化工人文化宫，推动经济较发达、职工人数多的县（县级市）实现工人文化宫建设全覆盖；整合工会资源，把县级工人文化宫打造成工会组织综合服务阵地。推进工人疗休养院改革发展，提升综合服务水平，“十四五”期间，各省级总工会至少有一家具有区位和资源优势、具有特色疗养服务和较强接待能力的工人疗休养院，全国工会每年组织劳模、职工疗休养达到500万人次，其中技术工人疗休养达到100万人次。充分发挥职工互助保障组织作用，加强和规范职工互助保障活动管理，推动实现全国职工互助保障活动省级统筹或管理，到2025年底参加职工互助保障活动的会员达到8000万人次左右，会员受益面和保障程度同步提高。加强职工院校和职业培训机构建设。推动职工旅行社、工会宾馆等积极承担劳模、职工疗休养等公益服务业务。

20.健全困难职工家庭常态化帮扶机制。

积极参与社会救助制度顶层设计，促进困难职工帮扶与社会救助体系相衔接。巩固拓展解困脱困工作成果，健全困难职工家庭生活状况监测预警机制和常态化帮扶机制。积极争取各级财政、社会资源、工会经费等多渠道投入帮扶资金，对深度困难、相对困难、意外致困等不同困难类型的困难职工家庭精准帮扶、分类施策，形成层次清晰、各有侧重、有机衔接的梯度帮扶工作格局，每年保障5万户以上深度困难职工家庭生活，解决15万户以上相对困难职工家庭、意外致困家庭生活暂时困难，引入公益慈善、爱心企业、志愿服务、专业机构等各类社会资源，推进困难职工帮扶与政府救助、公益慈善力量有机结合。推进“以工代赈”式救助帮扶，强化物质帮扶与扶志、扶智相结合，有效激发困难职工家庭解困脱困的内生动力。

21.实施提升职工生活品质行动。

以精准服务为导向，以满足职工美好生活需要为目标，制定实施工会提升职工生活品质行动方案，推行工会服务职工工作项目清单制度；建立工会帮扶工作智能化平台，健全工会服务职工满意度评价机制。开展帮扶中心赋能增效和幸福企业建设试点工作，提升职工服务中心（困难职工帮扶中心）综合服务职工功能，深入推进职工生活幸福型企业建设工作，精准对接社会资源与职工需求，培育一批服务项目，引导企业改善职工生产生活条件。2021年完成20家试点职工服务中心（困难职工帮扶中心）的综合服务能力建设、50家职工生活幸福型企业的标准化建设，孵化100家服务职工类社会资源；到2025年底实现县级以上工会职工服务中心（困难职工帮扶中心）综合服务职工能力全面提升，1万家企业完成职工生活幸福型企业标准化建设。

22.打造服务职工系列品牌。

健全完善常态化送温暖机制，继续叫响做实送温暖、金秋助学、阳光就业、职工医疗互助、工会法律援助、关爱农民工子女等工会工作传统品牌。“十四五”期间，各级工会每年筹集送温暖资金30亿元以上，走访慰问各类职工500万人以上。规范工会户外劳动者服务站点建设，引导更多社会资源参与，分批次推树1万个最美工会户外劳动者服务站点，设立专项奖补资金。做实叫响职工之家品牌，规范开展全国模范职工之家评选表彰，到2025年底建立起完善的模范职工之家动态复查监管机制。发挥模范职工之家示范引领作用，探索开展模范职工之家“结对共建”活动，普遍提升职工之家建设质量。加快推进工会志愿服务体系建设，建设管理服务平台，打造职工志愿服务品牌。按照“机制不变、力度不减、突出重点、建立品牌”的总体思路，聚焦思想引领、建功立业、劳动关系协调、就业帮扶、工会自身建设等重点任务，深入开展第三轮全国工会对口援疆援藏工作；帮助定点帮扶县巩固拓展脱贫攻坚成果，实现同乡村振兴有效衔接。

七、构建和谐劳动关系，推动共建共治共享社会治理

23.加大劳动法律法规源头参与力度。

积极推动和参与全国人大与社会组织协商立法的制度机制建设，推动涉及职工切身利益的法律法规政策制定和修改。推动和参与《工会法》修订完善，推动制定《基本劳动标准法》、《集体合同法》、《企业民主管理法》等相关劳动法律法规，进一步完善工会协调劳动关系法律制度体系。

24.推动完善构建和谐劳动关系制度机制。

进一步推动贯彻落实《中共中央 国务院关于构建和谐劳动关系的意见》，完善工会劳动关系发展态势监测和分析研判机制，打造来源可靠、覆盖广泛、运行顺畅、反应迅速的工会劳动关系监测系统，建设具有工会特色的劳动关系数据库。促进健全劳动关系协调机制，探索推进工会劳动关系调处标准化建设，构建劳动争议受理、调查、协调、调解、签约、结案、回访、归档等一体化业务标准体系；进一步健全协调劳动关系三方组织体系，重点推动工业园区、乡镇（街道）和行业系统建立三方机制，努力构建多层次、全方位、网格化劳动关系协商协调格局。大力推进行业性、区域性集体协商。以正常经营、已建工会的百人以上企业为重点，巩固集体协商建制率，确保重点企业单独签订集体合同率动态保持在80%以上；推动企业建立健全多形式多层级的沟通协商机制，应急、应事、一事一议开展灵活协商。开展集体协商质效评估工作，力争到2025年底覆盖60%以上的重点企业。举办城市工会集体协商竞赛活动。加强专职集体协商指导员队伍建设，力争到2025年底，基本实现专职集体协商指导员队伍对县级以上工会组织的全覆盖；加强对从事集体协商工作的工会干部、专职集体协商指导员和职工方协商代表的培训力度，全总每年重点培训100人次，各省、市级总工会每年培训不少于100人次，各县级总工会每年培训不少于30人次。健全完善劳动争议多元化解机制，推进企业和行业性、区域性劳动争议调解组织建设，完善诉调对接工作机制和调解协议履行机制，加强工会参与劳动争议调解工作与人民调解、仲裁调解、司法调解的联动协作和平台对接，不断提升劳动争议调裁审对接工作信息化、智能化水平。完善工会劳动法律法规监督机制，落实《工会劳动法律监督办法》，突出预防和协商的监督理念，重点围绕用人单位恶意欠薪、违法超时加班、违法裁员、未缴纳或未足额缴纳社会保险费等问题，规范和加强工会劳动法律监督工作。推行工会劳动法律监督“一函两书”、劳动用工法律体检、劳动用工监督评估等做法，推动各地工会建立健全与劳动保障监察机构的联动协作机制，全面提升监督实效。开展工会劳动保障法律监督员、劳动人事争议调解员和兼职仲裁员、劳动关系协调员（师）等专项培训。深化和谐劳动关系创建活动，扩大创建活动在非公有制企业和中小企业的覆盖面，推动区域性创建活动由工业园区向企业比较集中的乡镇（街道）、村（社区）拓展。配合行业主管部门构建和谐劳动关系企业指标体系，掌握在企业社会责任认证中的主动权、话语权。推进基层协调劳动关系工作服务站建设，建成一批可复制、可借鉴、可推广的和谐劳动关系示范点。

25.推进工会工作法治化建设。

加强工会法治宣传教育，不断增强职工群众法治观念、法治意识。实施工会系统“八五”普法规划，建设全国工会普法资源库，打造工会法治宣传教育活动品牌，培育工会法治宣传教育基地，壮大普法志愿者队伍。做强做实工会法律服务，加快法律服务站点建设，推进服务触角进一步向基层延伸。切实加强与司法行政部门沟通协作，进一步加大职工法律援助工作力度。评选表彰“全国维护职工权益杰出律师”，吸引和组织更多的社会律师等法律专业人才参与工会法律服务工作。进一步落实工会干部特别是领导干部学法用法制度，不断增强运用法治思维、法治方式开展工会工作的能力和水平。

26.健全落实“五个坚决”要求的长效机制。

认真贯彻落实总体国家安全观，围绕统筹发展和安全，坚持底线思维、增强忧患意识，坚持维权维稳相统一，发扬斗争精神、增强斗争本领，做到守土有责、守土负责、守土尽责，切实维护劳动领域政治安全，促进职工队伍团结统一与社会和谐稳定。参与推进市域社会治理现代化试点和工会系统平安中国建设，建立健全工会系统平安中国建设工作的能力体系。落实“五个坚决”要求，推进工会维护劳动领域安全稳定体系和能力建设，建立健全工会维护劳动领域政治安全长效机制，做好职工队伍稳定风险隐患专项排查化解工作，防患于未然，把风险隐患化解在基层一线、消除在萌芽状态。落实意识形态工作责任制，加强对意识形态风险隐患梳理排查、突发事件引导处置，牢牢掌握劳动领域意识形态斗争主导权。深化工会对劳动领域社会组织政治引领、示范带动、联系服务工作，形成党委全面领导、政府重视支持、工会联系引导、各方密切协作、社会组织专业服务、职工群众广泛参与的工作格局，推动建立创新示范基地，在条件成熟的地方培育孵化党委领导、工会主管的劳动领域社会组织或劳动领域社会组织联合会。健全完善工会信访治理体系，建好全国工会信访工作信息平台，完善信访矛盾多元化解机制，健全完善律师等第三方参与工会信访工作的组织形式和制度化渠道。

八、加快智慧工会建设，打造工会工作升级版

27.构建基于大数据技术的工会治理能力提升体系。

建立和完善工会数据资源管理体系，建设工会智能数字“云脑”平台、大数据分析研判和决策支撑系统、上下联动的应用市场。应用区块链技术，建立多节点的可信“工会身份链”，打造基于会员实名制数据的数字身份账户系统。整合共享各级工会数据和应用资源，强化基础数据采集校验能力和平台间对接联动，促进工会信息资源开放与应用，实现基础信息资源和业务信息资源的集约化采集、网络化汇聚、精准化管理。通过工会智能数字“云脑”体系，将数据能力和应用能力向各级工会赋能，为加强工会精准服务、业务协同、宏观决策提供技术和数据支撑。

28.构建基于互联网技术的工会服务应用创新体系。

建设全国工会服务平台，打造以媒体宣传、就业服务、技能提升、法律维权、职工帮扶、文化服务为重点的网上服务应用。创新网上普惠服务模式，推行网上普惠服务精准化，提升工会服务平台用户活跃度、满意度。创新工会多元化服务，推进与政务服务、社会服务、企业服务有机结合，实现工会网上服务资源优化配置和共享。构建工会网上服务评价体系。建设工会业务管理和网上协同办公平台，整合全总本级重点业务应用，推动工会工作流程再造、业务功能延伸和领域拓展，实现跨层级、跨地域、跨产业、跨工作部门的网上工作协同。

29.构建基于云计算技术的工会网信基础支撑体系。

完善工会信息基础设施建设，建设全总“工会云”、网络安全态势感知平台、运维平台及灾备系统。编制实施工会系统数据资源标准规范和开放利用标准，做好与国家基础数据库和重大信息化工程之间的标准衔接。加快工会电子政务网络建设，实现与同级政务网络平台安全接入。加强工会网络安全保障体系建设，严格落实网络安全等级保护、商用密码应用等网络安全法律法规和政策标准要求，落实安全可靠产品及国产密码应用，强化重要数据和个人信息保护，在建设和运维运营中同步加强网络安全保护，提升应对处置网络安全突发事件和重大风险防控能力。

30.巩固发展工会网上舆论阵地。

做强工会主流媒体，推进工会媒体深度融合，打造以工人日报、中工网、《中国工运》、《中国工人》为龙头的工会媒体集群，做大做强工会传媒旗舰，建强各级工会融媒体中心，构建网上网下一体，以新技术为支撑、“工”字特色内容建设为根本、新型运行管理模式为保障的报网端微刊全媒体传播体系。多措并举提升工会新闻发布水平，增强工会新闻发布触达率和实效性。做强叫响网评专栏，建设一支政治素质过硬、敏锐性高、责任心强、业务本领好的工会网评队伍。健全网络舆情应急处置制度，提高网络舆情信息监测的针对性、时效性，增强应急处置能力。推进职工网络素养提升主题活动，深入开展“网聚职工正能量 争做中国好网民”主题活动。参与举办国家网络安全宣传周。强化各级工会网站内容建设、功能建设、制度建设，完善网站信息发布和内容更新保障机制，做优工会知识服务平台，推动工会网站数据共享交换。

九、深化工会和职工对外交流交往合作，为推动构建人类命运共同体作贡献

31.拓展工会和职工国际交流交往合作的深度和广度。

坚持独立自主、互相尊重、求同存异、加强合作、增进友谊的工会外事工作方针，发挥民间外交优势，服务国家总体外交。广泛开展与周边国家、广大发展中国家工会组织和职工的友好交流。积极参与二十国集团劳动会议、金砖国家工会论坛、亚欧劳工论坛等多边机制，推动建设更加公正合理的全球治理体系。积极开展对欧工作，继续举办中德工会论坛，探索开展中欧工会绿色经济、数字经济对话交流活动，助力中欧绿色和数字领域伙伴关系发展。加强与重点国家工会的对话交流和高层交往，开展与美国等西方国家工会的对话交流。积极服务“一带一路”建设，搭建中资企业与有关国家工会组织的交流沟通平台，开展与“一带一路”沿线国家工会组织和职工的交流交往活动，加强职工技能国际交流。继续推进“一带一路”沿线国家工会干部来华进修汉语项目和“一带一路”职工人文交流项目。积极参加国际劳工组织理事会选举，参与国际劳工大会、理事会及有关会议和工作机制，深化与国际劳工组织有关的南南合作项目，加强对国际劳工公约、重要投资和贸易协定中的劳工条款等问题的研究，在劳工领域维护我主权、安全和发展利益。继续开展力所能及的对外援助。创新外事工作方式方法，实行线上交流与面对面交流相结合，提升工会和职工对外交流交往效率。加强工会外宣工作，面向国际劳工界广泛宣传习近平新时代中国特色社会主义思想，宣传中国式民主，讲好中国故事、讲好中国自由民主人权故事、讲好中国工人阶级故事、讲好中国工会故事。

32.加强与港澳台工会组织和劳动界交流合作。

加强同港澳台工会、劳工团体组织的沟通联系，支持港澳爱国工会力量，支持坚持一个中国原则和“九二共识”的台湾工会团体力量，做好港澳工会青年研讨营、港澳工会“五一”代表团、海峡职工论坛、台湾工会青年研讨营等品牌交流活动。联合协作开展职工职业技能竞赛，组织开展文化、体育交流活动，指导各地工会开展与港澳台工会交流合作项目。推动在内地工作港澳职工和台湾同胞享受同等工会服务，探索在内地（大陆）工作的港澳台职工纳入劳模等称号评选范围，引导港澳台职工融入祖国发展，投身粤港澳大湾区建设。着力开展爱国主义教育、国情国策宣讲，提高港澳职工爱国精神和国家意识。加强粤港澳三地工会协调合作，围绕粤港澳大湾区建立职工服务体系，支持港澳工会依法开展内地服务工作，构建粤港澳大湾区工会工作新格局。

十、深化工会改革创新，推动新时代工会工作高质量发展

33.系统谋划推进工会改革。

把增强政治性、先进性、群众性贯穿工会改革全过程，提出深化工会改革的总体思路、重点任务、具体举措、方法路径，明确改革的任务书、时间表、路线图、责任链，对改革任务、责任、进展、薄弱环节等进行盘点、跟踪问效。坚持问题导向、目标导向，对着问题去、盯着问题改，提出更多具有创新性引领性改革举措。支持基层工会组织开展差别化改革创新，切实增强团结教育、维护权益、服务职工功能。

34.健全工会工作制度机制。

系统总结党的十八大以来特别是中央党的群团工作会议以来工会改革的成绩和经验，做好工会改革总结评估，探索新时代工会工作的发展特点和规律，坚持和完善自觉接受党的领导制度，不断巩固党执政的阶级基础和群众基础；坚持和完善发挥工人阶级主力军作用制度，推动健全保障职工主人翁地位的各项制度安排；坚持和完善强化职工思想政治引领制度，加强和改进职工思想政治工作制度、职工文化建设制度；坚持和完善推进产业工人队伍建设改革制度，造就一支宏大的高素质的产业工人大军；坚持和完善维权服务制度，完善维护职工合法权益的制度，构建服务职工工作体系；坚持和完善劳动关系协调机制，推动完善社会治理体系；坚持和完善深化工会改革创新制度，密切联系职工群众；坚持和完善加强工会系统党的建设制度，努力提高工会系统党的建设的质量。做实全总深化工会改革领导小组工作机制，建立年度全国工会改革会议制度，搭建全国工会改革经验做法交流平台，对创新做法进行年度评比激励。健全完善改革评估长效机制，开展年度改革总结和评估工作，加强对制度执行的组织领导和监督检查，推动工会各项工作制度化、科学化、规范化。

35.激发基层工会活力。

树立落实到基层、落实靠基层理念，坚持把改革向基层延伸，把力量和资源充实到基层一线，使基层工会组织建起来、转起来、活起来。树立依靠会员办会理念，完善基层工会会务公开制度机制，保障会员的知情权、参与权、表达权、监督权。坚持不懈推进基层工会会员代表大会制度和民主选举制度落实落地，落实会员代表常任制，选优配强基层工会领导班子。到2025年底普遍实现基层工会按期换届选举，建设一支政治素质好、业务能力强，知职工、懂职工、爱职工的基层工会干部队伍。加强工会小组建设，选好工会小组长，不断壮大工会积极分子队伍。探索建立工会领导机关干部联系基层工会的工作机制，加强对下级工会的指导服务，积极协调解决基层工会面临的实际困难和问题。建立健全激励和保障机制，提升基层工会干部履职能力，让他们在政治上有地位、经济上有获得、履职上有保障、职业上有发展，不断增强工作积极性和职业荣誉感。推动实行非公有制企业兼职工会干部履职补贴制度。健全完善会员代表大会评议职工之家制度，深入开展会员评家工作，到2025年底实现基层工会普遍开展会员评家，以评家促进建家。加强对社会化工会工作者、专职集体协商指导员等的统筹管理，在薪酬福利、绩效奖惩、教育培训、职业发展等方面提供规范化指导，加强社会工作岗位开发设置。进一步加强工会社会工作专业人才队伍建设，不断提升服务职工群众的能力水平，壮大基层工会力量，力争到“十四五”末，全国社会化工会工作者总数稳定在4.5万人左右。

36.改进完善工会组织体系。

创新组织形式，理顺组织体制，构建纵横交织、覆盖广泛的工会组织体系。坚持以党建带工建为引领，完善党委领导、政府支持、工会主导、社会力量参与的建会入会工作格局，着力扩大工会组织覆盖面，实现组建工会和发展会员工作持续稳步发展。力争到“十四五”末，全国新组建基层工会组织60万个以上，新发展会员4000万人以上。在巩固传统领域建会入会基础上，重点加强“三新”领域工会组织建设，不断拓展建会入会新的增长点。以25人以上非公有制企业为重点，因地制宜、因行业制宜开展建会集中行动，推进规模较大的非公有制企业和社会组织依法规范建立工会组织。切实加强区域性、行业性工会联合会建设，健全乡镇（街道）-村（社区）-企业“小三级”工会组织体系，不断扩大对小微企业的有效覆盖。持续深化“八大群体”入会工作，聚焦货车司机、网约车司机、快递员、外卖配送员等重点群体，开展新就业形态劳动者入会集中行动，推动重点行业头部企业建立和完善工会组织。制定出台新就业形态劳动者入会相关意见，创新方式、优化程序，推行网上申请入会、集中入会仪式等做法，着力破解建会入会难题，最大限度地把农民工、灵活就业、新就业形态劳动者组织到工会中来。修订组建工会和发展会员考核奖励办法，完善考核通报等制度机制。联合国务院国资委制定加强和改进中央企业工会组织建设的指导意见，依法纠正国有企业在改革改制中随意撤并工会组织和工作机构、弱化工会组织地位作用问题。依法依规逐步调整和理顺产业工会与地方工会，与中央企业、企业集团及所属企业工会关系，与机关所属企事业单位工会的关系，进一步畅通体系、扩面提质。

37.充分发挥产业工会作用。

定期召开产业工会工作会议，及时研究解决产业工会工作中的重大问题。进一步明确全国产业工会、省级产业工会、城市产业工会、县级产业（行业）工会职责定位和工作重点。发挥产业工会全委会联合制、代表制组织制度优势，调整和优化产业工会委员单位组成，适当扩大非公有制企业、社会组织委员名额比例，增强代表性。到2025年底，各产业工会全国委员会委员和常务委员会成员中劳模和一线职工代表比例达到10%以上。完善与有关政府部门、行业协会的联席会议制度，产业协调劳动关系三方机制，探索创新产业工会行业联委会工作模式，发挥产业工会系统中的人大代表和政协工会界委员作用，支持产业工会参与产业、行业政策以及涉及产业职工切身利益的法规政策制定，及时发布具有行业指导意义的参考标准，开展国家重点工程和重大项目劳动竞赛、职工技能竞赛、培育大国工匠、职业技术培训、中心城市及县（区）范围内的行业集体协商等具有产（行）业特色的工作，更好发挥产业工会作用。建立健全产业工会工作评价体系和激励机制，加大对产业工会机构建设、经费投入、资源保障、活动开展等方面的支持力度，为产业工会发挥更大作用提供有利条件。

38.深化财务管理改革。

健全完善管理体制、经费收缴、预（决）算管理、财务监督与绩效管理等财务管理制度体系，建立财务管理公告制度。开发建设全总与省级工会贯通的工会经费收缴管理信息系统，及时准确掌握各级工会经费收缴情况。逐步扩大在京中央企业工会与全总建立财务关系的覆盖面，到2025年底基本实现全覆盖。积极推进工会经费收入电子票据改革试点。启动工会经费收缴改革，到2025年底基本形成权责清晰、财力协调、区域均衡的工会经费分配关系。深化工会全面预算管理，加强预算定额标准体系建设，逐步厘清全总本级和省级工会的事权和支出责任，稳步推进预算分配改革，建立完善转移支付制度，积极探索基层工会组织经费直达机制，出台促进基层工会留成经费足额到位的指导意见，推动解决县级以下特别是基层工会经费不足的突出问题。全面实施预算绩效管理，到2025年底基本实现县级以上工会预算绩效管理全覆盖。建立普惠职工的经费保障机制，将更多的工会经费用于直接服务职工群众。加强经济活动内部控制，强化财务监督检查，定期开展重大经济政策落实情况和重大项目预算执行情况专项监督。稳步推进工会财务信息公开。

39.加强工会经费审查审计监督。

按照工会一切经济活动都要纳入经审监督范围的总体要求，到2025年底形成以国家审计为指导、以工会经审组织为主体、以社会审计为补充、以职工会员监督为基础的工会常态化经审监督体系，不断拓展工会审查审计监督的广度和深度。逐步完善工会经审制度体系和工作机制，到2025年底形成覆盖主要审计类型的实务指南体系。加强审计项目和审计组织方式“两统筹”，实现工会经费审查监督、政策跟踪审计、预算执行审计、财务收支审计、经济责任审计、专项审计调查等统筹融合。深化预决算审查工作，审查监督重点向支出预算和政策执行拓展，建立经审会向同级工会党组织提交审计工作报告制度，完善以审计为基础的预决算审查机制。转变审计理念思路，把助力政策落实摆在突出位置，对政策落实情况进行全过程、全链条监督，推动工会重大决策部署落地见效。完善审计结果运用，做好审计整改“后半篇文章”，发挥工会经审组织的“审、帮、促”作用。加快推进工会经审工作信息化建设，构建全国工会经审工作平台，积极推广计算机审计、大数据审计等先进审计技术方法，开展“总体分析、发现疑点、分散核实、系统研究”的数字化审计，提高运用信息化技术查核问题、评价判断和分析问题能力。培养造就高素质专业化工会经审干部队伍，到2025年底，将全国工会专兼职经审干部轮训一遍。

40.提高工会资产管理效能。

积极推动职工文化教育事业、职工疗休养事业、职工互助保障事业纳入国家公共文化、卫生、保障服务体系，强化工会资产服务职工、服务基层功能。加强工会资产制度建设，积极探索工会资产制度的实现形式，到2025年底形成较为完善的工会资产制度体系。实施工会企事业经营业绩考核工作专项行动，到2025年底实现各级工会对本级工会企事业单位经营业绩考核全覆盖。深化工会资产体制机制改革创新，落实“统一所有、分级监管、单位使用”的工会资产监督管理体制，加强工会资产基础管理。建立健全工会资产统计制度、报告制度，加大工会资产产权登记工作力度，到2025年底工会资产不动产产权登记率逐步提高。加强工会资产管理信息化建设。积极稳妥推进工会事业单位改革工作，依法依规做好工人文化宫、工人疗休养院等工会资产阵地保护工作。

十一、坚持以党的政治建设为统领，提高工会工作能力和水平

41.全面加强工会系统党的建设。

把学习贯彻习近平新时代中国特色社会主义思想作为重大政治任务，切实用以武装头脑、指导实践、推动工作。坚持以党的政治建设为统领，牢固树立政治机关意识，推进模范机关建设，严格执行重大事项请示报告制度，确保习近平总书记重要指示批示精神和党中央重大决策部署在工会系统有效落实落地。健全完善理论武装长效机制，综合运用党组理论学习中心组学习、工会干部教育培训、党校和工会院校学习、网络学习培训等平台和载体，探索构建理论学习培训制度体系和成果评价体系。扎实开展党史学习教育、理想信念教育和中国工运史教育，巩固深化“不忘初心、牢记使命”主题教育成果。加强工会系统基层党组织建设，做好发展党员和党员教育管理工作。坚持党建带工建，积极探索“互联网＋党建”工作模式，构建党建和工会业务工作深度融合的长效机制，全面强化基层党组织的政治功能和组织力。坚持全面从严治党，推动各级工会领导干部认真落实全面从严治党的主体责任、管党治党的政治责任。突出抓好政治监督，健全内部巡视制度机制，用好“四种形态”，持之以恒正风肃纪反腐。锲而不舍落实中央八项规定及其实施细则精神，全面检视、靶向纠治“四风”，坚决防止反弹回潮。落实为基层减负各项规定，建立健全联系服务职工长效机制。

42.深化工会干部队伍建设。

坚持把好干部标准贯穿各级工会干部选育管用全过程，建设忠诚干净担当的高素质专业化工会干部队伍。坚持党管干部原则，突出政治标准，严把政治关、能力关、廉洁关，建立健全崇尚实干、带动担当、加油鼓劲的正向激励体系。优化工会领导机关领导班子配备，增强整体功能。发现培养选拔优秀年轻干部，加强对处级以下年轻干部的教育管理监督，拓宽来源渠道，加大年轻干部轮岗交流力度，做好挂职援派工作。完善优秀年轻干部人选库。加大工会干部管理监督力度，健全干部考核评价机制，推进工会干部监督制度化规范化建设，逐步形成适应工会机关实际的干部监督制度体系。按照有关规定做好工会干部双重管理工作。深化工会干部教育培训，2024年召开全国工会干部教育培训工作会议，研究制定全国工会干部教育培训五年规划，编写学习贯彻习近平总书记关于工人阶级和工会工作的重要论述教材、中国工运史教材，建强用好全国工会干部教育培训网。

43.不断拓宽工会理论研究新路子。

坚持把开展工会理论研究和调查研究作为重大任务，列入各级工会领导机关重要议事日程，构建上下结合、内外协作、整体推进的全方位研究格局。突出把深入学习和研究阐释习近平总书记关于工人阶级和工会工作的重要论述作为首要任务，每年举办学习习近平总书记关于工人阶级和工会工作的重要论述理论研讨会。加快构建中国特色工会学理论体系和工会干部培训教学体系，推进工会与劳动关系领域学科建设。加强工会研究阵地和智库建设，加强工会研究队伍建设，推出一批有深度、有价值、有分量的研究成果。县级以上工会领导机关要加强中长期工会理论研究与建设规划，每年制定年度研究计划，对本级工会理论研究和调查研究工作进行统筹安排。加大理论研究成果交流推广力度，推进应用转化，推动形成工作性意见、转化为政策制度、上升为法律法规。

十二、加强规划落实的组织保障

44.加强组织领导。

各级工会要把落实规划摆上重要工作位置、列入重要工作日程，坚持主要领导亲自抓、负总责，加强统筹协调，落实责任分工，及时研究解决规划实施中的重大问题。要把推进规划落实情况纳入对工会领导班子和领导干部的考核体系，抓好过程管理和目标考核，层层传导压力，逐级压实责任。各级工会要按照规划统一部署，结合当地实际制定实施方案，逐条逐项细化举措，明确落实规划的时间表、路线图和任务书，坚决避免“有部署、无落实”现象。坚持系统观念，注重传承创新，认真对照规划目标任务，对接已经出台的专项工作规划、结合已经部署的各项改革任务统筹抓好规划落实。

45.加强支撑保障。

各级工会要围绕规划确定的目标任务，建立健全规划落实的支撑保障机制，合理调配工作力量，建立多元化投入保障体系，加强预算保障，把更多资源力量用到重要领域、重点任务和关键环节。各地工会要积极主动作为，加强调研检查，推动规划落实纳入当地经济和社会发展总体规划落实的“大盘子”，努力实现一体部署、一体推进、一体检查。要依托工会系统研究平台阵地，发挥系统内外专家智库作用，围绕规划落实中的重大问题开展调查研究，为落实规划提供理论支撑和专业支持。

46.加强总结推广。

要建立规划落实情况的督促检查和工作通报制度，适时对目标任务完成情况开展调研检查，对工作进展情况、典型做法经验等进行通报，鼓励先进、鞭策后进。根据形势变化和工作要求，定期评估工作进展成效，做到一年一评估，以钉钉子精神一抓到底、抓出成效，增强规划落实的系统性和实效性。加强对规划的阐释解读，将规划作为工会干部教育培训的重要内容，引导广大工会干部全面准确理解规划、自觉推动落实规划。发挥工会系统媒体阵地作用，积极回应与规划有关的社会关切，结合调研督导、送教到基层等，以职工群众喜闻乐见、易于接受的形式，做好规划内容的宣传宣讲，凝聚广泛共识，争取各方支持，营造良好氛围。
