# Supplementary material for: Mapping the evolution of fertility support policies in China: A content and instrumental analysis
Source: PLoS One. 2025 Oct 9;20(10):e0332137. doi: 10.1371/journal.pone.0332137 (PMC12510515; doi:10.1371/journal.pone.0332137)
Supplement: S1 Appendix — (ZIP) [file pone.0332137.s001.zip › S1 Appendix. 226 original policy documents/151-中华人民共和国妇女权益保障法(2022修订)(FBM-CLI.1.5137689).docx]

中华人民共和国妇女权益保障法(2022修订)

制定机关： 全国人大常委会

发文字号：中华人民共和国主席令第122号

公布日期：2022.10.30

施行日期：2023.01.01

时效性： 现行有效

效力位阶： 法律

法规类别： 老少妇幼残保护

2018-2022对照 2005-2018编注 1992-2005编注

中华人民共和国主席令

（第一二二号）

　　《中华人民共和国妇女权益保障法》已由中华人民共和国第十三届全国人民代表大会常务委员会第三十七次会议于2022年10月30日修订通过，现予公布，自2023年1月1日起施行。

中华人民共和国主席　习近平

　　2022年10月30日

中华人民共和国妇女权益保障法

　　（1992年4月3日第七届全国人民代表大会第五次会议通过　根据2005年8月28日第十届全国人民代表大会常务委员会第十七次会议《关于修改〈中华人民共和国妇女权益保障法〉的决定》第一次修正　根据2018年10月26日第十三届全国人民代表大会常务委员会第六次会议《关于修改〈中华人民共和国野生动物保护法〉等十五部法律的决定》第二次修正　2022年10月30日第十三届全国人民代表大会常务委员会第三十七次会议修订）

目　　录

　　第一章　总　　则

　　第二章　政治权利

　　第三章　人身和人格权益

　　第四章　文化教育权益

　　第五章　劳动和社会保障权益

　　第六章　财产权益

　　第七章　婚姻家庭权益

　　第八章　救济措施

　　第九章　法律责任

　　第十章　附　　则

第一章　总　　则

　　第一条　为了保障妇女的合法权益，促进男女平等和妇女全面发展，充分发挥妇女在全面建设社会主义现代化国家中的作用，弘扬社会主义核心价值观，根据宪法，制定本法。

法宝联想：司法案例 12 篇期刊 6 篇律所实务 2 篇专题参考 1 篇

　　第二条　男女平等是国家的基本国策。妇女在政治的、经济的、文化的、社会的和家庭的生活等各方面享有同男子平等的权利。

　　国家采取必要措施，促进男女平等，消除对妇女一切形式的歧视，禁止排斥、限制妇女依法享有和行使各项权益。

　　国家保护妇女依法享有的特殊权益。

法宝联想：中央法规 1 篇草案 1 篇司法案例 792 篇期刊 17 篇专题参考 9 篇

　　第三条　坚持中国共产党对妇女权益保障工作的领导，建立政府主导、各方协同、社会参与的保障妇女权益工作机制。

　　各级人民政府应当重视和加强妇女权益的保障工作。

　　县级以上人民政府负责妇女儿童工作的机构，负责组织、协调、指导、督促有关部门做好妇女权益的保障工作。

　　县级以上人民政府有关部门在各自的职责范围内做好妇女权益的保障工作。

法宝联想：司法案例 30 篇期刊 2 篇专题参考 2 篇

　　第四条　保障妇女的合法权益是全社会的共同责任。国家机关、社会团体、企业事业单位、基层群众性自治组织以及其他组织和个人，应当依法保障妇女的权益。

　　国家采取有效措施，为妇女依法行使权利提供必要的条件。

法宝联想：地方法规 10 篇司法案例 583 篇期刊 4 篇律所实务 1 篇专题参考 3 篇

　　第五条　国务院制定和组织实施中国妇女发展纲要，将其纳入国民经济和社会发展规划，保障和促进妇女在各领域的全面发展。

　　县级以上地方各级人民政府根据中国妇女发展纲要，制定和组织实施本行政区域的妇女发展规划，将其纳入国民经济和社会发展规划。

　　县级以上人民政府应当将妇女权益保障所需经费列入本级预算。

法宝联想：司法案例 13 篇专题参考 1 篇

　　第六条　中华全国妇女联合会和地方各级妇女联合会依照法律和中华全国妇女联合会章程，代表和维护各族各界妇女的利益，做好维护妇女权益、促进男女平等和妇女全面发展的工作。

　　工会、共产主义青年团、残疾人联合会等群团组织应当在各自的工作范围内，做好维护妇女权益的工作。

法宝联想：地方法规 1 篇司法案例 298 篇期刊 4 篇律所实务 2 篇专题参考 1 篇

　　第七条　国家鼓励妇女自尊、自信、自立、自强，运用法律维护自身合法权益。

　　妇女应当遵守国家法律，尊重社会公德、职业道德和家庭美德，履行法律所规定的义务。

法宝联想：司法案例 13 篇期刊 4 篇律所实务 1 篇

　　第八条　有关机关制定或者修改涉及妇女权益的法律、法规、规章和其他规范性文件，应当听取妇女联合会的意见，充分考虑妇女的特殊权益，必要时开展男女平等评估。

法宝联想：司法案例 4 篇

　　第九条　国家建立健全妇女发展状况统计调查制度，完善性别统计监测指标体系，定期开展妇女发展状况和权益保障统计调查和分析，发布有关信息。

法宝联想：中央法规 1 篇司法案例 1 篇期刊 1 篇

　　第十条　国家将男女平等基本国策纳入国民教育体系，开展宣传教育，增强全社会的男女平等意识，培育尊重和关爱妇女的社会风尚。

法宝联想：中央法规 2 篇司法案例 1 篇期刊 6 篇专题参考 2 篇

　　第十一条　国家对保障妇女合法权益成绩显著的组织和个人，按照有关规定给予表彰和奖励。

法宝联想：地方法规 1 篇司法案例 2 篇期刊 2 篇

第二章　政治权利

　　第十二条　国家保障妇女享有与男子平等的政治权利。

法宝联想：司法案例 18 篇期刊 3 篇

　　第十三条　妇女有权通过各种途径和形式，依法参与管理国家事务、管理经济和文化事业、管理社会事务。

　　妇女和妇女组织有权向各级国家机关提出妇女权益保障方面的意见和建议。

法宝联想：司法案例 14 篇期刊 2 篇

　　第十四条　妇女享有与男子平等的选举权和被选举权。

　　全国人民代表大会和地方各级人民代表大会的代表中，应当保证有适当数量的妇女代表。国家采取措施，逐步提高全国人民代表大会和地方各级人民代表大会的妇女代表的比例。

　　居民委员会、村民委员会成员中，应当保证有适当数量的妇女成员。

法宝联想：司法案例 5 篇期刊 2 篇专题参考 2 篇

　　第十五条　国家积极培养和选拔女干部，重视培养和选拔少数民族女干部。

　　国家机关、群团组织、企业事业单位培养、选拔和任用干部，应当坚持男女平等的原则，并有适当数量的妇女担任领导成员。

　　妇女联合会及其团体会员，可以向国家机关、群团组织、企业事业单位推荐女干部。

　　国家采取措施支持女性人才成长。

法宝联想：中央法规 1 篇司法案例 5 篇期刊 1 篇

　　第十六条　妇女联合会代表妇女积极参与国家和社会事务的民主协商、民主决策、民主管理和民主监督。

法宝联想：司法案例 12 篇期刊 3 篇

　　第十七条　对于有关妇女权益保障工作的批评或者合理可行的建议，有关部门应当听取和采纳；对于有关侵害妇女权益的申诉、控告和检举，有关部门应当查清事实，负责处理，任何组织和个人不得压制或者打击报复。

法宝联想：司法案例 4 篇期刊 1 篇专题参考 1 篇

第三章　人身和人格权益

　　第十八条　国家保障妇女享有与男子平等的人身和人格权益。

法宝联想：地方法规 2 篇司法案例 2 篇

　　第十九条　妇女的人身自由不受侵犯。禁止非法拘禁和以其他非法手段剥夺或者限制妇女的人身自由；禁止非法搜查妇女的身体。

法宝联想：司法案例 2 篇

　　第二十条　妇女的人格尊严不受侵犯。禁止用侮辱、诽谤等方式损害妇女的人格尊严。

法宝联想：司法案例 6 篇期刊 1 篇

　　第二十一条　妇女的生命权、身体权、健康权不受侵犯。禁止虐待、遗弃、残害、买卖以及其他侵害女性生命健康权益的行为。

　　禁止进行非医学需要的胎儿性别鉴定和选择性别的人工终止妊娠。

　　医疗机构施行生育手术、特殊检查或者特殊治疗时，应当征得妇女本人同意；在妇女与其家属或者关系人意见不一致时，应当尊重妇女本人意愿。

法宝联想：法律动态 1 篇司法案例 13 篇期刊 2 篇

　　第二十二条　禁止拐卖、绑架妇女；禁止收买被拐卖、绑架的妇女；禁止阻碍解救被拐卖、绑架的妇女。

　　各级人民政府和公安、民政、人力资源和社会保障、卫生健康等部门及村民委员会、居民委员会按照各自的职责及时发现报告，并采取措施解救被拐卖、绑架的妇女，做好被解救妇女的安置、救助和关爱等工作。妇女联合会协助和配合做好有关工作。任何组织和个人不得歧视被拐卖、绑架的妇女。

法宝联想：中央法规 1 篇地方法规 1 篇法律动态 2 篇司法案例 163 篇期刊 7 篇律所实务 3 篇专题参考 1 篇

　　第二十三条　禁止违背妇女意愿，以言语、文字、图像、肢体行为等方式对其实施性骚扰。

　　受害妇女可以向有关单位和国家机关投诉。接到投诉的有关单位和国家机关应当及时处理，并书面告知处理结果。

　　受害妇女可以向公安机关报案，也可以向人民法院提起民事诉讼，依法请求行为人承担民事责任。

法宝联想：法律动态 3 篇司法案例 217 篇期刊 4 篇律所实务 7 篇专题参考 3 篇

　　第二十四条　学校应当根据女学生的年龄阶段，进行生理卫生、心理健康和自我保护教育，在教育、管理、设施等方面采取措施，提高其防范性侵害、性骚扰的自我保护意识和能力，保障女学生的人身安全和身心健康发展。

　　学校应当建立有效预防和科学处置性侵害、性骚扰的工作制度。对性侵害、性骚扰女学生的违法犯罪行为，学校不得隐瞒，应当及时通知受害未成年女学生的父母或者其他监护人，向公安机关、教育行政部门报告，并配合相关部门依法处理。

　　对遭受性侵害、性骚扰的女学生，学校、公安机关、教育行政部门等相关单位和人员应当保护其隐私和个人信息，并提供必要的保护措施。

法宝联想：司法案例 245 篇期刊 4 篇专题参考 1 篇

　　第二十五条　用人单位应当采取下列措施预防和制止对妇女的性骚扰：

　　（一）制定禁止性骚扰的规章制度；

　　（二）明确负责机构或者人员；

　　（三）开展预防和制止性骚扰的教育培训活动；

　　（四）采取必要的安全保卫措施；

　　（五）设置投诉电话、信箱等，畅通投诉渠道；

　　（六）建立和完善调查处置程序，及时处置纠纷并保护当事人隐私和个人信息；

　　（七）支持、协助受害妇女依法维权，必要时为受害妇女提供心理疏导；

　　（八）其他合理的预防和制止性骚扰措施。

法宝联想：中外条约 1 篇司法案例 8 篇期刊 2 篇律所实务 2 篇

　　第二十六条　住宿经营者应当及时准确登记住宿人员信息，健全住宿服务规章制度，加强安全保障措施；发现可能侵害妇女权益的违法犯罪行为，应当及时向公安机关报告。

法宝联想：司法案例 92 篇案例报道 1 篇期刊 5 篇专题参考 2 篇

　　第二十七条　禁止卖淫、嫖娼；禁止组织、强迫、引诱、容留、介绍妇女卖淫或者对妇女进行猥亵活动；禁止组织、强迫、引诱、容留、介绍妇女在任何场所或者利用网络进行淫秽表演活动。

法宝联想：中央法规 1 篇地方法规 2 篇中外条约 1 篇司法案例 416 篇案例报道 2 篇期刊 8 篇律所实务 3 篇专题参考 2 篇

　　第二十八条　妇女的姓名权、肖像权、名誉权、荣誉权、隐私权和个人信息等人格权益受法律保护。

　　媒体报道涉及妇女事件应当客观、适度，不得通过夸大事实、过度渲染等方式侵害妇女的人格权益。

　　禁止通过大众传播媒介或者其他方式贬低损害妇女人格。未经本人同意，不得通过广告、商标、展览橱窗、报纸、期刊、图书、音像制品、电子出版物、网络等形式使用妇女肖像，但法律另有规定的除外。

法宝联想：司法案例 206 篇期刊 5 篇专题参考 1 篇

　　第二十九条　禁止以恋爱、交友为由或者在终止恋爱关系、离婚之后，纠缠、骚扰妇女，泄露、传播妇女隐私和个人信息。

　　妇女遭受上述侵害或者面临上述侵害现实危险的，可以向人民法院申请人身安全保护令。

法宝联想：法律动态 2 篇司法案例 20 篇案例报道 1 篇期刊 2 篇

　　第三十条　国家建立健全妇女健康服务体系，保障妇女享有基本医疗卫生服务，开展妇女常见病、多发病的预防、筛查和诊疗，提高妇女健康水平。

　　国家采取必要措施，开展经期、孕期、产期、哺乳期和更年期的健康知识普及、卫生保健和疾病防治，保障妇女特殊生理时期的健康需求，为有需要的妇女提供心理健康服务支持。

法宝联想：中央法规 2 篇地方法规 1 篇司法案例 3129 篇期刊 4 篇律所实务 2 篇专题参考 3 篇

　　第三十一条　县级以上地方人民政府应当设立妇幼保健机构，为妇女提供保健以及常见病防治服务。

　　国家鼓励和支持社会力量通过依法捐赠、资助或者提供志愿服务等方式，参与妇女卫生健康事业，提供安全的生理健康用品或者服务，满足妇女多样化、差异化的健康需求。

　　用人单位应当定期为女职工安排妇科疾病、乳腺疾病检查以及妇女特殊需要的其他健康检查。

法宝联想：司法案例 347 篇期刊 1 篇律所实务 3 篇

　　第三十二条　妇女依法享有生育子女的权利，也有不生育子女的自由。

法宝联想：地方法规 1 篇司法案例 16408 篇期刊 15 篇律所实务 1 篇专题参考 2 篇

　　第三十三条　国家实行婚前、孕前、孕产期和产后保健制度，逐步建立妇女全生育周期系统保健制度。医疗保健机构应当提供安全、有效的医疗保健服务，保障妇女生育安全和健康。

　　有关部门应当提供安全、有效的避孕药具和技术，保障妇女的健康和安全。

法宝联想：地方法规 1 篇司法案例 19079 篇案例报道 1 篇期刊 17 篇律所实务 3 篇专题参考 2 篇

　　第三十四条　各级人民政府在规划、建设基础设施时，应当考虑妇女的特殊需求，配备满足妇女需要的公共厕所和母婴室等公共设施。

法宝联想：中外条约 1 篇司法案例 18 篇期刊 3 篇

第四章　文化教育权益

　　第三十五条　国家保障妇女享有与男子平等的文化教育权利。

法宝联想：司法案例 8 篇期刊 4 篇律所实务 1 篇

　　第三十六条　父母或者其他监护人应当履行保障适龄女性未成年人接受并完成义务教育的义务。

　　对无正当理由不送适龄女性未成年人入学的父母或者其他监护人，由当地乡镇人民政府或者县级人民政府教育行政部门给予批评教育，依法责令其限期改正。居民委员会、村民委员会应当协助政府做好相关工作。

　　政府、学校应当采取有效措施，解决适龄女性未成年人就学存在的实际困难，并创造条件，保证适龄女性未成年人完成义务教育。

法宝联想：司法案例 12 篇期刊 3 篇专题参考 1 篇

　　第三十七条　学校和有关部门应当执行国家有关规定，保障妇女在入学、升学、授予学位、派出留学、就业指导和服务等方面享有与男子平等的权利。

　　学校在录取学生时，除国家规定的特殊专业外，不得以性别为由拒绝录取女性或者提高对女性的录取标准。

　　各级人民政府应当采取措施，保障女性平等享有接受中高等教育的权利和机会。

法宝联想：司法案例 11 篇期刊 5 篇专题参考 2 篇

　　第三十八条　各级人民政府应当依照规定把扫除妇女中的文盲、半文盲工作，纳入扫盲和扫盲后继续教育规划，采取符合妇女特点的组织形式和工作方法，组织、监督有关部门具体实施。

法宝联想：中外条约 1 篇法律动态 1 篇司法案例 20 篇期刊 4 篇

　　第三十九条　国家健全全民终身学习体系，为妇女终身学习创造条件。

　　各级人民政府和有关部门应当采取措施，根据城镇和农村妇女的需要，组织妇女接受职业教育和实用技术培训。

法宝联想：地方法规 2 篇中外条约 1 篇司法案例 7 篇期刊 14 篇律所实务 1 篇专题参考 5 篇

　　第四十条　国家机关、社会团体和企业事业单位应当执行国家有关规定，保障妇女从事科学、技术、文学、艺术和其他文化活动，享有与男子平等的权利。

法宝联想：中外条约 1 篇法律动态 1 篇司法案例 67 篇期刊 16 篇律所实务 13 篇专题参考 7 篇

第五章　劳动和社会保障权益

　　第四十一条　国家保障妇女享有与男子平等的劳动权利和社会保障权利。

法宝联想：司法案例 56 篇期刊 4 篇律所实务 1 篇专题参考 1 篇

　　第四十二条　各级人民政府和有关部门应当完善就业保障政策措施，防止和纠正就业性别歧视，为妇女创造公平的就业创业环境，为就业困难的妇女提供必要的扶持和援助。

法宝联想：法律动态 1 篇司法案例 24 篇期刊 17 篇律所实务 1 篇专题参考 6 篇

　　第四十三条　用人单位在招录（聘）过程中，除国家另有规定外，不得实施下列行为：

　　（一）限定为男性或者规定男性优先；

　　（二）除个人基本信息外，进一步询问或者调查女性求职者的婚育情况；

　　（三）将妊娠测试作为入职体检项目；

　　（四）将限制结婚、生育或者婚姻、生育状况作为录（聘）用条件；

　　（五）其他以性别为由拒绝录（聘）用妇女或者差别化地提高对妇女录（聘）用标准的行为。

法宝联想：司法案例 1162 篇期刊 3 篇律所实务 3 篇专题参考 1 篇

　　第四十四条　用人单位在录（聘）用女职工时，应当依法与其签订劳动（聘用）合同或者服务协议，劳动（聘用）合同或者服务协议中应当具备女职工特殊保护条款，并不得规定限制女职工结婚、生育等内容。

　　职工一方与用人单位订立的集体合同中应当包含男女平等和女职工权益保护相关内容，也可以就相关内容制定专章、附件或者单独订立女职工权益保护专项集体合同。

法宝联想：地方法规 1 篇司法案例 646 篇期刊 5 篇律所实务 3 篇

　　第四十五条　实行男女同工同酬。妇女在享受福利待遇方面享有与男子平等的权利。

法宝联想：司法案例 21 篇期刊 2 篇律所实务 1 篇

　　第四十六条　在晋职、晋级、评聘专业技术职称和职务、培训等方面，应当坚持男女平等的原则，不得歧视妇女。

法宝联想：中央法规 1 篇地方法规 10 篇中外条约 1 篇法律动态 1 篇司法案例 57 篇案例报道 2 篇期刊 12 篇律所实务 1 篇专题参考 4 篇

　　第四十七条　用人单位应当根据妇女的特点，依法保护妇女在工作和劳动时的安全、健康以及休息的权利。

　　妇女在经期、孕期、产期、哺乳期受特殊保护。

法宝联想：司法案例 74 篇期刊 20 篇律所实务 3 篇专题参考 6 篇

　　第四十八条　用人单位不得因结婚、怀孕、产假、哺乳等情形，降低女职工的工资和福利待遇，限制女职工晋职、晋级、评聘专业技术职称和职务，辞退女职工，单方解除劳动（聘用）合同或者服务协议。

　　女职工在怀孕以及依法享受产假期间，劳动（聘用）合同或者服务协议期满的，劳动（聘用）合同或者服务协议期限自动延续至产假结束。但是，用人单位依法解除、终止劳动（聘用）合同、服务协议，或者女职工依法要求解除、终止劳动（聘用）合同、服务协议的除外。

　　用人单位在执行国家退休制度时，不得以性别为由歧视妇女。

法宝联想：司法案例 289 篇期刊 5 篇律所实务 2 篇专题参考 2 篇

　　第四十九条　人力资源和社会保障部门应当将招聘、录取、晋职、晋级、评聘专业技术职称和职务、培训、辞退等过程中的性别歧视行为纳入劳动保障监察范围。

法宝联想：司法案例 52 篇期刊 3 篇律所实务 2 篇

　　第五十条　国家发展社会保障事业，保障妇女享有社会保险、社会救助和社会福利等权益。

　　国家提倡和鼓励为帮助妇女而开展的社会公益活动。

法宝联想：地方法规 1 篇司法案例 52 篇案例报道 1 篇期刊 6 篇专题参考 1 篇

　　第五十一条　国家实行生育保险制度，建立健全婴幼儿托育服务等与生育相关的其他保障制度。

　　国家建立健全职工生育休假制度，保障孕产期女职工依法享有休息休假权益。

　　地方各级人民政府和有关部门应当按照国家有关规定，为符合条件的困难妇女提供必要的生育救助。

法宝联想：中央法规 2 篇司法案例 41 篇案例报道 1 篇期刊 29 篇律所实务 3 篇专题参考 8 篇

　　第五十二条　各级人民政府和有关部门应当采取必要措施，加强贫困妇女、老龄妇女、残疾妇女等困难妇女的权益保障，按照有关规定为其提供生活帮扶、就业创业支持等关爱服务。

法宝联想：司法案例 897 篇期刊 2 篇律所实务 1 篇专题参考 1 篇

第六章　财产权益

　　第五十三条　国家保障妇女享有与男子平等的财产权利。

法宝联想：地方法规 2 篇司法案例 6 篇期刊 1 篇专题参考 1 篇

　　第五十四条　在夫妻共同财产、家庭共有财产关系中，不得侵害妇女依法享有的权益。

法宝联想：司法案例 3 篇期刊 1 篇专题参考 1 篇

　　第五十五条　妇女在农村集体经济组织成员身份确认、土地承包经营、集体经济组织收益分配、土地征收补偿安置或者征用补偿以及宅基地使用等方面，享有与男子平等的权利。

　　申请农村土地承包经营权、宅基地使用权等不动产登记，应当在不动产登记簿和权属证书上将享有权利的妇女等家庭成员全部列明。征收补偿安置或者征用补偿协议应当将享有相关权益的妇女列入，并记载权益内容。

法宝联想：地方法规 3 篇司法案例 649 篇期刊 2 篇专题参考 2 篇

　　第五十六条　村民自治章程、村规民约，村民会议、村民代表会议的决定以及其他涉及村民利益事项的决定，不得以妇女未婚、结婚、离婚、丧偶、户无男性等为由，侵害妇女在农村集体经济组织中的各项权益。

　　因结婚男方到女方住所落户的，男方和子女享有与所在地农村集体经济组织成员平等的权益。

法宝联想：司法案例 78 篇专题参考 1 篇

　　第五十七条　国家保护妇女在城镇集体所有财产关系中的权益。妇女依照法律、法规的规定享有相关权益。

法宝联想：司法案例 17 篇律所实务 1 篇

　　第五十八条　妇女享有与男子平等的继承权。妇女依法行使继承权，不受歧视。

　　丧偶妇女有权依法处分继承的财产，任何组织和个人不得干涉。

法宝联想：法律动态 1 篇司法案例 2 篇期刊 4 篇律所实务 1 篇专题参考 3 篇

　　第五十九条　丧偶儿媳对公婆尽了主要赡养义务的，作为第一顺序继承人，其继承权不受子女代位继承的影响。

法宝联想：草案 1 篇司法案例 3 篇期刊 3 篇

第七章　婚姻家庭权益

　　第六十条　国家保障妇女享有与男子平等的婚姻家庭权利。

法宝联想：司法案例 1 篇期刊 3 篇专题参考 1 篇

　　第六十一条　国家保护妇女的婚姻自主权。禁止干涉妇女的结婚、离婚自由。

　　第六十二条　国家鼓励男女双方在结婚登记前，共同进行医学检查或者相关健康体检。

　　第六十三条　婚姻登记机关应当提供婚姻家庭辅导服务，引导当事人建立平等、和睦、文明的婚姻家庭关系。

　　第六十四条　女方在怀孕期间、分娩后一年内或者终止妊娠后六个月内，男方不得提出离婚；但是，女方提出离婚或者人民法院认为确有必要受理男方离婚请求的除外。

　　第六十五条　禁止对妇女实施家庭暴力。

　　县级以上人民政府有关部门、司法机关、社会团体、企业事业单位、基层群众性自治组织以及其他组织，应当在各自的职责范围内预防和制止家庭暴力，依法为受害妇女提供救助。

　　第六十六条　妇女对夫妻共同财产享有与其配偶平等的占有、使用、收益和处分的权利，不受双方收入状况等情形的影响。

　　对夫妻共同所有的不动产以及可以联名登记的动产，女方有权要求在权属证书上记载其姓名；认为记载的权利人、标的物、权利比例等事项有错误的，有权依法申请更正登记或者异议登记，有关机构应当按照其申请依法办理相应登记手续。

　　第六十七条　离婚诉讼期间，夫妻一方申请查询登记在对方名下财产状况且确因客观原因不能自行收集的，人民法院应当进行调查取证，有关部门和单位应当予以协助。

　　离婚诉讼期间，夫妻双方均有向人民法院申报全部夫妻共同财产的义务。一方隐藏、转移、变卖、损毁、挥霍夫妻共同财产，或者伪造夫妻共同债务企图侵占另一方财产的，在离婚分割夫妻共同财产时，对该方可以少分或者不分财产。

　　第六十八条　夫妻双方应当共同负担家庭义务，共同照顾家庭生活。

　　女方因抚育子女、照料老人、协助男方工作等负担较多义务的，有权在离婚时要求男方予以补偿。补偿办法由双方协议确定；协议不成的，可以向人民法院提起诉讼。

　　第六十九条　离婚时，分割夫妻共有的房屋或者处理夫妻共同租住的房屋，由双方协议解决；协议不成的，可以向人民法院提起诉讼。

　　第七十条　父母双方对未成年子女享有平等的监护权。

　　父亲死亡、无监护能力或者有其他情形不能担任未成年子女的监护人的，母亲的监护权任何组织和个人不得干涉。

　　第七十一条　女方丧失生育能力的，在离婚处理子女抚养问题时，应当在最有利于未成年子女的条件下，优先考虑女方的抚养要求。

第八章　救济措施

　　第七十二条　对侵害妇女合法权益的行为，任何组织和个人都有权予以劝阻、制止或者向有关部门提出控告或者检举。有关部门接到控告或者检举后，应当依法及时处理，并为控告人、检举人保密。

　　妇女的合法权益受到侵害的，有权要求有关部门依法处理，或者依法申请调解、仲裁，或者向人民法院起诉。

　　对符合条件的妇女，当地法律援助机构或者司法机关应当给予帮助，依法为其提供法律援助或者司法救助。

　　第七十三条　妇女的合法权益受到侵害的，可以向妇女联合会等妇女组织求助。妇女联合会等妇女组织应当维护被侵害妇女的合法权益，有权要求并协助有关部门或者单位查处。有关部门或者单位应当依法查处，并予以答复；不予处理或者处理不当的，县级以上人民政府负责妇女儿童工作的机构、妇女联合会可以向其提出督促处理意见，必要时可以提请同级人民政府开展督查。

　　受害妇女进行诉讼需要帮助的，妇女联合会应当给予支持和帮助。

　　第七十四条　用人单位侵害妇女劳动和社会保障权益的，人力资源和社会保障部门可以联合工会、妇女联合会约谈用人单位，依法进行监督并要求其限期纠正。

　　第七十五条　妇女在农村集体经济组织成员身份确认等方面权益受到侵害的，可以申请乡镇人民政府等进行协调，或者向人民法院起诉。

　　乡镇人民政府应当对村民自治章程、村规民约，村民会议、村民代表会议的决定以及其他涉及村民利益事项的决定进行指导，对其中违反法律、法规和国家政策规定，侵害妇女合法权益的内容责令改正；受侵害妇女向农村土地承包仲裁机构申请仲裁或者向人民法院起诉的，农村土地承包仲裁机构或者人民法院应当依法受理。

　　第七十六条　县级以上人民政府应当开通全国统一的妇女权益保护服务热线，及时受理、移送有关侵害妇女合法权益的投诉、举报；有关部门或者单位接到投诉、举报后，应当及时予以处置。

　　鼓励和支持群团组织、企业事业单位、社会组织和个人参与建设妇女权益保护服务热线，提供妇女权益保护方面的咨询、帮助。

　　第七十七条　侵害妇女合法权益，导致社会公共利益受损的，检察机关可以发出检察建议；有下列情形之一的，检察机关可以依法提起公益诉讼：

　　（一）确认农村妇女集体经济组织成员身份时侵害妇女权益或者侵害妇女享有的农村土地承包和集体收益、土地征收征用补偿分配权益和宅基地使用权益；

　　（二）侵害妇女平等就业权益；

　　（三）相关单位未采取合理措施预防和制止性骚扰；

　　（四）通过大众传播媒介或者其他方式贬低损害妇女人格；

　　（五）其他严重侵害妇女权益的情形。

　　第七十八条　国家机关、社会团体、企业事业单位对侵害妇女权益的行为，可以支持受侵害的妇女向人民法院起诉。

第九章　法律责任

　　第七十九条　违反本法第二十二条第二款规定，未履行报告义务的，依法对直接负责的主管人员和其他直接责任人员给予处分。

　　第八十条　违反本法规定，对妇女实施性骚扰的，由公安机关给予批评教育或者出具告诫书，并由所在单位依法给予处分。

　　学校、用人单位违反本法规定，未采取必要措施预防和制止性骚扰，造成妇女权益受到侵害或者社会影响恶劣的，由上级机关或者主管部门责令改正；拒不改正或者情节严重的，依法对直接负责的主管人员和其他直接责任人员给予处分。

　　第八十一条　违反本法第二十六条规定，未履行报告等义务的，依法给予警告、责令停业整顿或者吊销营业执照、吊销相关许可证，并处一万元以上五万元以下罚款。

　　第八十二条　违反本法规定，通过大众传播媒介或者其他方式贬低损害妇女人格的，由公安、网信、文化旅游、广播电视、新闻出版或者其他有关部门依据各自的职权责令改正，并依法给予行政处罚。

　　第八十三条　用人单位违反本法第四十三条和第四十八条规定的，由人力资源和社会保障部门责令改正；拒不改正或者情节严重的，处一万元以上五万元以下罚款。

　　第八十四条　违反本法规定，对侵害妇女权益的申诉、控告、检举，推诿、拖延、压制不予查处，或者对提出申诉、控告、检举的人进行打击报复的，依法责令改正，并对直接负责的主管人员和其他直接责任人员给予处分。

　　国家机关及其工作人员未依法履行职责，对侵害妇女权益的行为未及时制止或者未给予受害妇女必要帮助，造成严重后果的，依法对直接负责的主管人员和其他直接责任人员给予处分。

　　违反本法规定，侵害妇女人身和人格权益、文化教育权益、劳动和社会保障权益、财产权益以及婚姻家庭权益的，依法责令改正，直接负责的主管人员和其他直接责任人员属于国家工作人员的，依法给予处分。

　　第八十五条　违反本法规定，侵害妇女的合法权益，其他法律、法规规定行政处罚的，从其规定；造成财产损失或者人身损害的，依法承担民事责任；构成犯罪的，依法追究刑事责任。

第十章　附　　则

　　第八十六条　本法自2023年1月1日起施行。

背景资料

立法草案

全国人民代表大会宪法和法律委员会关于《中华人民共和国妇女权益保障法(修订草案三次审议稿)》修改意见的报告

全国人民代表大会宪法和法律委员会关于《中华人民共和国妇女权益保障法(修订草案)》审议结果的报告

妇女权益保障法(修订草案二次审议稿)征求意见

全国人民代表大会宪法和法律委员会关于《中华人民共和国妇女权益保障法(修订草案)》修改情况的汇报

妇女权益保障法(修订草案)征求意见

关于《中华人民共和国妇女权益保障法(修订草案)》的说明

全国人大法律委员会关于治安管理处罚法(草案三次审议稿)、公证法(草案三次审议稿)和关于修改妇女权益保障法的决定(草案)修改意见的报告(节选)(妇女权益保障法)

全国人大法律委员会关于《中华人民共和国妇女权益保障法修正案(草案)》审议结果的报告

更多

本篇引用的法规

中央法规

中华全国妇女联合会章程(2018修改)

全国人民代表大会常务委员会关于修改《中华人民共和国野生动物保护法》等十五部法律的决定(含：计量法、防治法、保障法、妇女权益保障法、广告法、节约能源法、防沙治沙法、农业机械化促进法、农产品质量安全法、循环经济促进法、旅游法、环境保护税法、公共图书馆法、船舶吨税法)

中华人民共和国宪法(2018修正)

全国人民代表大会常务委员会关于修改《中华人民共和国妇女权益保障法》的决定(2005)

中国妇女发展纲要（２００１－２０１０年）

引用本篇的法规 案例 论文

法律

全国人民代表大会宪法和法律委员会关于第十三届全国人民代表大会第五次会议主席团交付审议的代表提出的议案审议结果的报告

司法解释

最高人民检察院、中华全国妇女联合会关于印发《妇女权益保障检察公益诉讼典型案例》的通知

地方法规规章

昆明市人民政府关于印发昆明妇女发展规划和昆明儿童发展规划的通知

上海市妇女权益保障条例

崇左市人民政府关于印发崇左市妇女发展规划和崇左市儿童发展规划的通知

怒江州人民政府关于印发怒江妇女发展规划和怒江儿童发展规划的通知

邯郸市人民政府关于印发邯郸市妇女儿童发展规划(2021—2030年)的通知

红河州人民政府关于印发红河州妇女发展规划和红河州儿童发展规划的通知

天津高院发布十一起老年人权益保护典型案例
