# Supplementary material for: Mapping the evolution of fertility support policies in China: A content and instrumental analysis
Source: PLoS One. 2025 Oct 9;20(10):e0332137. doi: 10.1371/journal.pone.0332137 (PMC12510515; doi:10.1371/journal.pone.0332137)
Supplement: S1 Appendix — (ZIP) [file pone.0332137.s001.zip › S1 Appendix. 226 original policy documents/173-中华人民共和国未成年人保护法(2020修订)(FBM-CLI-1-346976).docx]

中华人民共和国未成年人保护法(2020修订)

发布部门： 全国人大常委会

发文字号：中华人民共和国主席令第57号

发布日期：2020.10.17

实施日期：2021.06.01

时效性： 现行有效

效力级别： 法律

法规类别： 老少妇幼残保护

专题分类： 未成年人保护

2012-2020对照 2006-2012编注 1991-2006对照

中华人民共和国主席令

（第五十七号）

《中华人民共和国未成年人保护法》已由中华人民共和国第十三届全国人民代表大会常务委员会第二十二次会议于2020年10月17日修订通过，现予公布，自2021年6月1日起施行。

中华人民共和国主席 习近平

2020年10月17日

中华人民共和国未成年人保护法

（1991年9月4日第七届全国人民代表大会常务委员会第二十一次会议通过　2006年12月29日第十届全国人民代表大会常务委员会第二十五次会议第一次修订　根据2012年10月26日第十一届全国人民代表大会常务委员会第二十九次会议《关于修改〈中华人民共和国未成年人保护法〉的决定》修正　2020年10月17日第十三届全国人民代表大会常务委员会第二十二次会议第二次修订）

目录

第一章　总则

第二章　家庭保护

第三章　学校保护

第四章　社会保护

第五章　网络保护

第六章　政府保护

第七章　司法保护

第八章　法律责任

第九章　附则

第一章　总则

第一条　为了保护未成年人身心健康，保障未成年人合法权益，促进未成年人德智体美劳全面发展，培养有理想、有道德、有文化、有纪律的社会主义建设者和接班人，培养担当民族复兴大任的时代新人，根据宪法，制定本法。

第二条　本法所称未成年人是指未满十八周岁的公民。

第三条　国家保障未成年人的生存权、发展权、受保护权、参与权等权利。

未成年人依法平等地享有各项权利，不因本人及其父母或者其他监护人的民族、种族、性别、户籍、职业、宗教信仰、教育程度、家庭状况、身心健康状况等受到歧视。

第四条　保护未成年人，应当坚持最有利于未成年人的原则。处理涉及未成年人事项，应当符合下列要求：

（一）给予未成年人特殊、优先保护；

（二）尊重未成年人人格尊严；

（三）保护未成年人隐私权和个人信息；

（四）适应未成年人身心健康发展的规律和特点；

（五）听取未成年人的意见；

（六）保护与教育相结合。

第五条　国家、社会、学校和家庭应当对未成年人进行理想教育、道德教育、科学教育、文化教育、法治教育、国家安全教育、健康教育、劳动教育，加强爱国主义、集体主义和中国特色社会主义的教育，培养爱祖国、爱人民、爱劳动、爱科学、爱社会主义的公德，抵制资本主义、封建主义和其他腐朽思想的侵蚀，引导未成年人树立和践行社会主义核心价值观。

第六条　保护未成年人，是国家机关、武装力量、政党、人民团体、企业事业单位、社会组织、城乡基层群众性自治组织、未成年人的监护人以及其他成年人的共同责任。

国家、社会、学校和家庭应当教育和帮助未成年人维护自身合法权益，增强自我保护的意识和能力。

第七条　未成年人的父母或者其他监护人依法对未成年人承担监护职责。

国家采取措施指导、支持、帮助和监督未成年人的父母或者其他监护人履行监护职责。

第八条　县级以上人民政府应当将未成年人保护工作纳入国民经济和社会发展规划，相关经费纳入本级政府预算。

第九条　县级以上人民政府应当建立未成年人保护工作协调机制，统筹、协调、督促和指导有关部门在各自职责范围内做好未成年人保护工作。协调机制具体工作由县级以上人民政府民政部门承担，省级人民政府也可以根据本地实际情况确定由其他有关部门承担。

第十条　共产主义青年团、妇女联合会、工会、残疾人联合会、关心下一代工作委员会、青年联合会、学生联合会、少年先锋队以及其他人民团体、有关社会组织，应当协助各级人民政府及其有关部门、人民检察院、人民法院做好未成年人保护工作，维护未成年人合法权益。

第十一条　任何组织或者个人发现不利于未成年人身心健康或者侵犯未成年人合法权益的情形，都有权劝阻、制止或者向公安、民政、教育等有关部门提出检举、控告。

国家机关、居民委员会、村民委员会、密切接触未成年人的单位及其工作人员，在工作中发现未成年人身心健康受到侵害、疑似受到侵害或者面临其他危险情形的，应当立即向公安、民政、教育等有关部门报告。

有关部门接到涉及未成年人的检举、控告或者报告，应当依法及时受理、处置，并以适当方式将处理结果告知相关单位和人员。

第十二条　国家鼓励和支持未成年人保护方面的科学研究，建设相关学科、设置相关专业，加强人才培养。

第十三条　国家建立健全未成年人统计调查制度，开展未成年人健康、受教育等状况的统计、调查和分析，发布未成年人保护的有关信息。

第十四条　国家对保护未成年人有显著成绩的组织和个人给予表彰和奖励。

第二章　家庭保护

第十五条　未成年人的父母或者其他监护人应当学习家庭教育知识，接受家庭教育指导，创造良好、和睦、文明的家庭环境。

共同生活的其他成年家庭成员应当协助未成年人的父母或者其他监护人抚养、教育和保护未成年人。

第十六条　未成年人的父母或者其他监护人应当履行下列监护职责：

（一）为未成年人提供生活、健康、安全等方面的保障；

（二）关注未成年人的生理、心理状况和情感需求；

（三）教育和引导未成年人遵纪守法、勤俭节约，养成良好的思想品德和行为习惯；

（四）对未成年人进行安全教育，提高未成年人的自我保护意识和能力；

（五）尊重未成年人受教育的权利，保障适龄未成年人依法接受并完成义务教育；

（六）保障未成年人休息、娱乐和体育锻炼的时间，引导未成年人进行有益身心健康的活动；

（七）妥善管理和保护未成年人的财产；

（八）依法代理未成年人实施民事法律行为；

（九）预防和制止未成年人的不良行为和违法犯罪行为，并进行合理管教；

（十）其他应当履行的监护职责。

第十七条　未成年人的父母或者其他监护人不得实施下列行为：

（一）虐待、遗弃、非法送养未成年人或者对未成年人实施家庭暴力；

（二）放任、教唆或者利用未成年人实施违法犯罪行为；

（三）放任、唆使未成年人参与邪教、迷信活动或者接受恐怖主义、分裂主义、极端主义等侵害；

（四）放任、唆使未成年人吸烟（含电子烟，下同）、饮酒、赌博、流浪乞讨或者欺凌他人；

（五）放任或者迫使应当接受义务教育的未成年人失学、辍学；

（六）放任未成年人沉迷网络，接触危害或者可能影响其身心健康的图书、报刊、电影、广播电视节目、音像制品、电子出版物和网络信息等；

（七）放任未成年人进入营业性娱乐场所、酒吧、互联网上网服务营业场所等不适宜未成年人活动的场所；

（八）允许或者迫使未成年人从事国家规定以外的劳动；

（九）允许、迫使未成年人结婚或者为未成年人订立婚约；

（十）违法处分、侵吞未成年人的财产或者利用未成年人牟取不正当利益；

（十一）其他侵犯未成年人身心健康、财产权益或者不依法履行未成年人保护义务的行为。

第十八条　未成年人的父母或者其他监护人应当为未成年人提供安全的家庭生活环境，及时排除引发触电、烫伤、跌落等伤害的安全隐患；采取配备儿童安全座椅、教育未成年人遵守交通规则等措施，防止未成年人受到交通事故的伤害；提高户外安全保护意识，避免未成年人发生溺水、动物伤害等事故。

第十九条　未成年人的父母或者其他监护人应当根据未成年人的年龄和智力发展状况，在作出与未成年人权益有关的决定前，听取未成年人的意见，充分考虑其真实意愿。

第二十条　未成年人的父母或者其他监护人发现未成年人身心健康受到侵害、疑似受到侵害或者其他合法权益受到侵犯的，应当及时了解情况并采取保护措施；情况严重的，应当立即向公安、民政、教育等部门报告。

第二十一条　未成年人的父母或者其他监护人不得使未满八周岁或者由于身体、心理原因需要特别照顾的未成年人处于无人看护状态，或者将其交由无民事行为能力、限制民事行为能力、患有严重传染性疾病或者其他不适宜的人员临时照护。

未成年人的父母或者其他监护人不得使未满十六周岁的未成年人脱离监护单独生活。

第二十二条　未成年人的父母或者其他监护人因外出务工等原因在一定期限内不能完全履行监护职责的，应当委托具有照护能力的完全民事行为能力人代为照护；无正当理由的，不得委托他人代为照护。

未成年人的父母或者其他监护人在确定被委托人时，应当综合考虑其道德品质、家庭状况、身心健康状况、与未成年人生活情感上的联系等情况，并听取有表达意愿能力未成年人的意见。

具有下列情形之一的，不得作为被委托人：

（一）曾实施性侵害、虐待、遗弃、拐卖、暴力伤害等违法犯罪行为；

（二）有吸毒、酗酒、赌博等恶习；

（三）曾拒不履行或者长期怠于履行监护、照护职责；

（四）其他不适宜担任被委托人的情形。

第二十三条　未成年人的父母或者其他监护人应当及时将委托照护情况书面告知未成年人所在学校、幼儿园和实际居住地的居民委员会、村民委员会，加强和未成年人所在学校、幼儿园的沟通；与未成年人、被委托人至少每周联系和交流一次，了解未成年人的生活、学习、心理等情况，并给予未成年人亲情关爱。

未成年人的父母或者其他监护人接到被委托人、居民委员会、村民委员会、学校、幼儿园等关于未成年人心理、行为异常的通知后，应当及时采取干预措施。

第二十四条　未成年人的父母离婚时，应当妥善处理未成年子女的抚养、教育、探望、财产等事宜，听取有表达意愿能力未成年人的意见。不得以抢夺、藏匿未成年子女等方式争夺抚养权。

未成年人的父母离婚后，不直接抚养未成年子女的一方应当依照协议、人民法院判决或者调解确定的时间和方式，在不影响未成年人学习、生活的情况下探望未成年子女，直接抚养的一方应当配合，但被人民法院依法中止探望权的除外。

第三章　学校保护

第二十五条　学校应当全面贯彻国家教育方针，坚持立德树人，实施素质教育，提高教育质量，注重培养未成年学生认知能力、合作能力、创新能力和实践能力，促进未成年学生全面发展。

学校应当建立未成年学生保护工作制度，健全学生行为规范，培养未成年学生遵纪守法的良好行为习惯。

第二十六条　幼儿园应当做好保育、教育工作，遵循幼儿身心发展规律，实施启蒙教育，促进幼儿在体质、智力、品德等方面和谐发展。

第二十七条　学校、幼儿园的教职员工应当尊重未成年人人格尊严，不得对未成年人实施体罚、变相体罚或者其他侮辱人格尊严的行为。

第二十八条　学校应当保障未成年学生受教育的权利，不得违反国家规定开除、变相开除未成年学生。

学校应当对尚未完成义务教育的辍学未成年学生进行登记并劝返复学；劝返无效的，应当及时向教育行政部门书面报告。

第二十九条　学校应当关心、爱护未成年学生，不得因家庭、身体、心理、学习能力等情况歧视学生。对家庭困难、身心有障碍的学生，应当提供关爱；对行为异常、学习有困难的学生，应当耐心帮助。

学校应当配合政府有关部门建立留守未成年学生、困境未成年学生的信息档案，开展关爱帮扶工作。

第三十条　学校应当根据未成年学生身心发展特点，进行社会生活指导、心理健康辅导、青春期教育和生命教育。

第三十一条　学校应当组织未成年学生参加与其年龄相适应的日常生活劳动、生产劳动和服务性劳动，帮助未成年学生掌握必要的劳动知识和技能，养成良好的劳动习惯。

第三十二条　学校、幼儿园应当开展勤俭节约、反对浪费、珍惜粮食、文明饮食等宣传教育活动，帮助未成年人树立浪费可耻、节约为荣的意识，养成文明健康、绿色环保的生活习惯。

第三十三条　学校应当与未成年学生的父母或者其他监护人互相配合，合理安排未成年学生的学习时间，保障其休息、娱乐和体育锻炼的时间。

学校不得占用国家法定节假日、休息日及寒暑假期，组织义务教育阶段的未成年学生集体补课，加重其学习负担。

幼儿园、校外培训机构不得对学龄前未成年人进行小学课程教育。

第三十四条　学校、幼儿园应当提供必要的卫生保健条件，协助卫生健康部门做好在校、在园未成年人的卫生保健工作。

第三十五条　学校、幼儿园应当建立安全管理制度，对未成年人进行安全教育，完善安保设施、配备安保人员，保障未成年人在校、在园期间的人身和财产安全。

学校、幼儿园不得在危及未成年人人身安全、身心健康的校舍和其他设施、场所中进行教育教学活动。

学校、幼儿园安排未成年人参加文化娱乐、社会实践等集体活动，应当保护未成年人的身心健康，防止发生人身伤害事故。

第三十六条　使用校车的学校、幼儿园应当建立健全校车安全管理制度，配备安全管理人员，定期对校车进行安全检查，对校车驾驶人进行安全教育，并向未成年人讲解校车安全乘坐知识，培养未成年人校车安全事故应急处理技能。

第三十七条　学校、幼儿园应当根据需要，制定应对自然灾害、事故灾难、公共卫生事件等突发事件和意外伤害的预案，配备相应设施并定期进行必要的演练。

未成年人在校内、园内或者本校、本园组织的校外、园外活动中发生人身伤害事故的，学校、幼儿园应当立即救护，妥善处理，及时通知未成年人的父母或者其他监护人，并向有关部门报告。

第三十八条　学校、幼儿园不得安排未成年人参加商业性活动，不得向未成年人及其父母或者其他监护人推销或者要求其购买指定的商品和服务。

学校、幼儿园不得与校外培训机构合作为未成年人提供有偿课程辅导。

第三十九条　学校应当建立学生欺凌防控工作制度，对教职员工、学生等开展防治学生欺凌的教育和培训。

学校对学生欺凌行为应当立即制止，通知实施欺凌和被欺凌未成年学生的父母或者其他监护人参与欺凌行为的认定和处理；对相关未成年学生及时给予心理辅导、教育和引导；对相关未成年学生的父母或者其他监护人给予必要的家庭教育指导。

对实施欺凌的未成年学生，学校应当根据欺凌行为的性质和程度，依法加强管教。对严重的欺凌行为，学校不得隐瞒，应当及时向公安机关、教育行政部门报告，并配合相关部门依法处理。

第四十条　学校、幼儿园应当建立预防性侵害、性骚扰未成年人工作制度。对性侵害、性骚扰未成年人等违法犯罪行为，学校、幼儿园不得隐瞒，应当及时向公安机关、教育行政部门报告，并配合相关部门依法处理。

学校、幼儿园应当对未成年人开展适合其年龄的性教育，提高未成年人防范性侵害、性骚扰的自我保护意识和能力。对遭受性侵害、性骚扰的未成年人，学校、幼儿园应当及时采取相关的保护措施。

第四十一条　婴幼儿照护服务机构、早期教育服务机构、校外培训机构、校外托管机构等应当参照本章有关规定，根据不同年龄阶段未成年人的成长特点和规律，做好未成年人保护工作。

第四章　社会保护

第四十二条　全社会应当树立关心、爱护未成年人的良好风尚。

国家鼓励、支持和引导人民团体、企业事业单位、社会组织以及其他组织和个人，开展有利于未成年人健康成长的社会活动和服务。

第四十三条　居民委员会、村民委员会应当设置专人专岗负责未成年人保护工作，协助政府有关部门宣传未成年人保护方面的法律法规，指导、帮助和监督未成年人的父母或者其他监护人依法履行监护职责，建立留守未成年人、困境未成年人的信息档案并给予关爱帮扶。

居民委员会、村民委员会应当协助政府有关部门监督未成年人委托照护情况，发现被委托人缺乏照护能力、怠于履行照护职责等情况，应当及时向政府有关部门报告，并告知未成年人的父母或者其他监护人，帮助、督促被委托人履行照护职责。

第四十四条　爱国主义教育基地、图书馆、青少年宫、儿童活动中心、儿童之家应当对未成年人免费开放；博物馆、纪念馆、科技馆、展览馆、美术馆、文化馆、社区公益性互联网上网服务场所以及影剧院、体育场馆、动物园、植物园、公园等场所，应当按照有关规定对未成年人免费或者优惠开放。

国家鼓励爱国主义教育基地、博物馆、科技馆、美术馆等公共场馆开设未成年人专场，为未成年人提供有针对性的服务。

国家鼓励国家机关、企业事业单位、部队等开发自身教育资源，设立未成年人开放日，为未成年人主题教育、社会实践、职业体验等提供支持。

国家鼓励科研机构和科技类社会组织对未成年人开展科学普及活动。

第四十五条　城市公共交通以及公路、铁路、水路、航空客运等应当按照有关规定对未成年人实施免费或者优惠票价。

第四十六条　国家鼓励大型公共场所、公共交通工具、旅游景区景点等设置母婴室、婴儿护理台以及方便幼儿使用的坐便器、洗手台等卫生设施，为未成年人提供便利。

第四十七条　任何组织或者个人不得违反有关规定，限制未成年人应当享有的照顾或者优惠。

第四十八条　国家鼓励创作、出版、制作和传播有利于未成年人健康成长的图书、报刊、电影、广播电视节目、舞台艺术作品、音像制品、电子出版物和网络信息等。

第四十九条　新闻媒体应当加强未成年人保护方面的宣传，对侵犯未成年人合法权益的行为进行舆论监督。新闻媒体采访报道涉及未成年人事件应当客观、审慎和适度，不得侵犯未成年人的名誉、隐私和其他合法权益。

第五十条　禁止制作、复制、出版、发布、传播含有宣扬淫秽、色情、暴力、邪教、迷信、赌博、引诱自杀、恐怖主义、分裂主义、极端主义等危害未成年人身心健康内容的图书、报刊、电影、广播电视节目、舞台艺术作品、音像制品、电子出版物和网络信息等。

第五十一条　任何组织或者个人出版、发布、传播的图书、报刊、电影、广播电视节目、舞台艺术作品、音像制品、电子出版物或者网络信息，包含可能影响未成年人身心健康内容的，应当以显著方式作出提示。

第五十二条　禁止制作、复制、发布、传播或者持有有关未成年人的淫秽色情物品和网络信息。

第五十三条　任何组织或者个人不得刊登、播放、张贴或者散发含有危害未成年人身心健康内容的广告；不得在学校、幼儿园播放、张贴或者散发商业广告；不得利用校服、教材等发布或者变相发布商业广告。

第五十四条　禁止拐卖、绑架、虐待、非法收养未成年人，禁止对未成年人实施性侵害、性骚扰。

禁止胁迫、引诱、教唆未成年人参加黑社会性质组织或者从事违法犯罪活动。

禁止胁迫、诱骗、利用未成年人乞讨。

第五十五条　生产、销售用于未成年人的食品、药品、玩具、用具和游戏游艺设备、游乐设施等，应当符合国家或者行业标准，不得危害未成年人的人身安全和身心健康。上述产品的生产者应当在显著位置标明注意事项，未标明注意事项的不得销售。

第五十六条　未成年人集中活动的公共场所应当符合国家或者行业安全标准，并采取相应安全保护措施。对可能存在安全风险的设施，应当定期进行维护，在显著位置设置安全警示标志并标明适龄范围和注意事项；必要时应当安排专门人员看管。

大型的商场、超市、医院、图书馆、博物馆、科技馆、游乐场、车站、码头、机场、旅游景区景点等场所运营单位应当设置搜寻走失未成年人的安全警报系统。场所运营单位接到求助后，应当立即启动安全警报系统，组织人员进行搜寻并向公安机关报告。

公共场所发生突发事件时，应当优先救护未成年人。

第五十七条　旅馆、宾馆、酒店等住宿经营者接待未成年人入住，或者接待未成年人和成年人共同入住时，应当询问父母或者其他监护人的联系方式、入住人员的身份关系等有关情况；发现有违法犯罪嫌疑的，应当立即向公安机关报告，并及时联系未成年人的父母或者其他监护人。

第五十八条　学校、幼儿园周边不得设置营业性娱乐场所、酒吧、互联网上网服务营业场所等不适宜未成年人活动的场所。营业性歌舞娱乐场所、酒吧、互联网上网服务营业场所等不适宜未成年人活动场所的经营者，不得允许未成年人进入；游艺娱乐场所设置的电子游戏设备，除国家法定节假日外，不得向未成年人提供。经营者应当在显著位置设置未成年人禁入、限入标志；对难以判明是否是未成年人的，应当要求其出示身份证件。

第五十九条　学校、幼儿园周边不得设置烟、酒、彩票销售网点。禁止向未成年人销售烟、酒、彩票或者兑付彩票奖金。烟、酒和彩票经营者应当在显著位置设置不向未成年人销售烟、酒或者彩票的标志；对难以判明是否是未成年人的，应当要求其出示身份证件。

任何人不得在学校、幼儿园和其他未成年人集中活动的公共场所吸烟、饮酒。

第六十条　禁止向未成年人提供、销售管制刀具或者其他可能致人严重伤害的器具等物品。经营者难以判明购买者是否是未成年人的，应当要求其出示身份证件。

第六十一条　任何组织或者个人不得招用未满十六周岁未成年人，国家另有规定的除外。

营业性娱乐场所、酒吧、互联网上网服务营业场所等不适宜未成年人活动的场所不得招用已满十六周岁的未成年人。

招用已满十六周岁未成年人的单位和个人应当执行国家在工种、劳动时间、劳动强度和保护措施等方面的规定，不得安排其从事过重、有毒、有害等危害未成年人身心健康的劳动或者危险作业。

任何组织或者个人不得组织未成年人进行危害其身心健康的表演等活动。经未成年人的父母或者其他监护人同意，未成年人参与演出、节目制作等活动，活动组织方应当根据国家有关规定，保障未成年人合法权益。

第六十二条　密切接触未成年人的单位招聘工作人员时，应当向公安机关、人民检察院查询应聘者是否具有性侵害、虐待、拐卖、暴力伤害等违法犯罪记录；发现其具有前述行为记录的，不得录用。

密切接触未成年人的单位应当每年定期对工作人员是否具有上述违法犯罪记录进行查询。通过查询或者其他方式发现其工作人员具有上述行为的，应当及时解聘。

第六十三条　任何组织或者个人不得隐匿、毁弃、非法删除未成年人的信件、日记、电子邮件或者其他网络通讯内容。

除下列情形外，任何组织或者个人不得开拆、查阅未成年人的信件、日记、电子邮件或者其他网络通讯内容：

（一）无民事行为能力未成年人的父母或者其他监护人代未成年人开拆、查阅；

（二）因国家安全或者追查刑事犯罪依法进行检查；

（三）紧急情况下为了保护未成年人本人的人身安全。

第五章　网络保护

第六十四条　国家、社会、学校和家庭应当加强未成年人网络素养宣传教育，培养和提高未成年人的网络素养，增强未成年人科学、文明、安全、合理使用网络的意识和能力，保障未成年人在网络空间的合法权益。

第六十五条　国家鼓励和支持有利于未成年人健康成长的网络内容的创作与传播，鼓励和支持专门以未成年人为服务对象、适合未成年人身心健康特点的网络技术、产品、服务的研发、生产和使用。

第六十六条　网信部门及其他有关部门应当加强对未成年人网络保护工作的监督检查，依法惩处利用网络从事危害未成年人身心健康的活动，为未成年人提供安全、健康的网络环境。

第六十七条　网信部门会同公安、文化和旅游、新闻出版、电影、广播电视等部门根据保护不同年龄阶段未成年人的需要，确定可能影响未成年人身心健康网络信息的种类、范围和判断标准。

第六十八条　新闻出版、教育、卫生健康、文化和旅游、网信等部门应当定期开展预防未成年人沉迷网络的宣传教育，监督网络产品和服务提供者履行预防未成年人沉迷网络的义务，指导家庭、学校、社会组织互相配合，采取科学、合理的方式对未成年人沉迷网络进行预防和干预。

任何组织或者个人不得以侵害未成年人身心健康的方式对未成年人沉迷网络进行干预。

第六十九条　学校、社区、图书馆、文化馆、青少年宫等场所为未成年人提供的互联网上网服务设施，应当安装未成年人网络保护软件或者采取其他安全保护技术措施。

智能终端产品的制造者、销售者应当在产品上安装未成年人网络保护软件，或者以显著方式告知用户未成年人网络保护软件的安装渠道和方法。

第七十条　学校应当合理使用网络开展教学活动。未经学校允许，未成年学生不得将手机等智能终端产品带入课堂，带入学校的应当统一管理。

学校发现未成年学生沉迷网络的，应当及时告知其父母或者其他监护人，共同对未成年学生进行教育和引导，帮助其恢复正常的学习生活。

第七十一条　未成年人的父母或者其他监护人应当提高网络素养，规范自身使用网络的行为，加强对未成年人使用网络行为的引导和监督。

未成年人的父母或者其他监护人应当通过在智能终端产品上安装未成年人网络保护软件、选择适合未成年人的服务模式和管理功能等方式，避免未成年人接触危害或者可能影响其身心健康的网络信息，合理安排未成年人使用网络的时间，有效预防未成年人沉迷网络。

第七十二条　信息处理者通过网络处理未成年人个人信息的，应当遵循合法、正当和必要的原则。处理不满十四周岁未成年人个人信息的，应当征得未成年人的父母或者其他监护人同意，但法律、行政法规另有规定的除外。

未成年人、父母或者其他监护人要求信息处理者更正、删除未成年人个人信息的，信息处理者应当及时采取措施予以更正、删除，但法律、行政法规另有规定的除外。

第七十三条　网络服务提供者发现未成年人通过网络发布私密信息的，应当及时提示，并采取必要的保护措施。

第七十四条　网络产品和服务提供者不得向未成年人提供诱导其沉迷的产品和服务。

网络游戏、网络直播、网络音视频、网络社交等网络服务提供者应当针对未成年人使用其服务设置相应的时间管理、权限管理、消费管理等功能。

以未成年人为服务对象的在线教育网络产品和服务，不得插入网络游戏链接，不得推送广告等与教学无关的信息。

第七十五条　网络游戏经依法审批后方可运营。

国家建立统一的未成年人网络游戏电子身份认证系统。网络游戏服务提供者应当要求未成年人以真实身份信息注册并登录网络游戏。

网络游戏服务提供者应当按照国家有关规定和标准，对游戏产品进行分类，作出适龄提示，并采取技术措施，不得让未成年人接触不适宜的游戏或者游戏功能。

网络游戏服务提供者不得在每日二十二时至次日八时向未成年人提供网络游戏服务。

第七十六条　网络直播服务提供者不得为未满十六周岁的未成年人提供网络直播发布者账号注册服务；为年满十六周岁的未成年人提供网络直播发布者账号注册服务时，应当对其身份信息进行认证，并征得其父母或者其他监护人同意。

第七十七条　任何组织或者个人不得通过网络以文字、图片、音视频等形式，对未成年人实施侮辱、诽谤、威胁或者恶意损害形象等网络欺凌行为。

遭受网络欺凌的未成年人及其父母或者其他监护人有权通知网络服务提供者采取删除、屏蔽、断开链接等措施。网络服务提供者接到通知后，应当及时采取必要的措施制止网络欺凌行为，防止信息扩散。

第七十八条　网络产品和服务提供者应当建立便捷、合理、有效的投诉和举报渠道，公开投诉、举报方式等信息，及时受理并处理涉及未成年人的投诉、举报。

第七十九条　任何组织或者个人发现网络产品、服务含有危害未成年人身心健康的信息，有权向网络产品和服务提供者或者网信、公安等部门投诉、举报。

第八十条　网络服务提供者发现用户发布、传播可能影响未成年人身心健康的信息且未作显著提示的，应当作出提示或者通知用户予以提示；未作出提示的，不得传输相关信息。

网络服务提供者发现用户发布、传播含有危害未成年人身心健康内容的信息的，应当立即停止传输相关信息，采取删除、屏蔽、断开链接等处置措施，保存有关记录，并向网信、公安等部门报告。

网络服务提供者发现用户利用其网络服务对未成年人实施违法犯罪行为的，应当立即停止向该用户提供网络服务，保存有关记录，并向公安机关报告。

第六章　政府保护

第八十一条　县级以上人民政府承担未成年人保护协调机制具体工作的职能部门应当明确相关内设机构或者专门人员，负责承担未成年人保护工作。

乡镇人民政府和街道办事处应当设立未成年人保护工作站或者指定专门人员，及时办理未成年人相关事务；支持、指导居民委员会、村民委员会设立专人专岗，做好未成年人保护工作。

第八十二条　各级人民政府应当将家庭教育指导服务纳入城乡公共服务体系，开展家庭教育知识宣传，鼓励和支持有关人民团体、企业事业单位、社会组织开展家庭教育指导服务。

第八十三条　各级人民政府应当保障未成年人受教育的权利，并采取措施保障留守未成年人、困境未成年人、残疾未成年人接受义务教育。

对尚未完成义务教育的辍学未成年学生，教育行政部门应当责令父母或者其他监护人将其送入学校接受义务教育。

第八十四条　各级人民政府应当发展托育、学前教育事业，办好婴幼儿照护服务机构、幼儿园，支持社会力量依法兴办母婴室、婴幼儿照护服务机构、幼儿园。

县级以上地方人民政府及其有关部门应当培养和培训婴幼儿照护服务机构、幼儿园的保教人员，提高其职业道德素质和业务能力。

第八十五条　各级人民政府应当发展职业教育，保障未成年人接受职业教育或者职业技能培训，鼓励和支持人民团体、企业事业单位、社会组织为未成年人提供职业技能培训服务。

第八十六条　各级人民政府应当保障具有接受普通教育能力、能适应校园生活的残疾未成年人就近在普通学校、幼儿园接受教育；保障不具有接受普通教育能力的残疾未成年人在特殊教育学校、幼儿园接受学前教育、义务教育和职业教育。

各级人民政府应当保障特殊教育学校、幼儿园的办学、办园条件，鼓励和支持社会力量举办特殊教育学校、幼儿园。

第八十七条　地方人民政府及其有关部门应当保障校园安全，监督、指导学校、幼儿园等单位落实校园安全责任，建立突发事件的报告、处置和协调机制。

第八十八条　公安机关和其他有关部门应当依法维护校园周边的治安和交通秩序，设置监控设备和交通安全设施，预防和制止侵害未成年人的违法犯罪行为。

第八十九条　地方人民政府应当建立和改善适合未成年人的活动场所和设施，支持公益性未成年人活动场所和设施的建设和运行，鼓励社会力量兴办适合未成年人的活动场所和设施，并加强管理。

地方人民政府应当采取措施，鼓励和支持学校在国家法定节假日、休息日及寒暑假期将文化体育设施对未成年人免费或者优惠开放。

地方人民政府应当采取措施，防止任何组织或者个人侵占、破坏学校、幼儿园、婴幼儿照护服务机构等未成年人活动场所的场地、房屋和设施。

第九十条　各级人民政府及其有关部门应当对未成年人进行卫生保健和营养指导，提供卫生保健服务。

卫生健康部门应当依法对未成年人的疫苗预防接种进行规范，防治未成年人常见病、多发病，加强传染病防治和监督管理，做好伤害预防和干预，指导和监督学校、幼儿园、婴幼儿照护服务机构开展卫生保健工作。

教育行政部门应当加强未成年人的心理健康教育，建立未成年人心理问题的早期发现和及时干预机制。卫生健康部门应当做好未成年人心理治疗、心理危机干预以及精神障碍早期识别和诊断治疗等工作。

第九十一条　各级人民政府及其有关部门对困境未成年人实施分类保障，采取措施满足其生活、教育、安全、医疗康复、住房等方面的基本需要。

第九十二条　具有下列情形之一的，民政部门应当依法对未成年人进行临时监护：

（一）未成年人流浪乞讨或者身份不明，暂时查找不到父母或者其他监护人；

（二）监护人下落不明且无其他人可以担任监护人；

（三）监护人因自身客观原因或者因发生自然灾害、事故灾难、公共卫生事件等突发事件不能履行监护职责，导致未成年人监护缺失；

（四）监护人拒绝或者怠于履行监护职责，导致未成年人处于无人照料的状态；

（五）监护人教唆、利用未成年人实施违法犯罪行为，未成年人需要被带离安置；

（六）未成年人遭受监护人严重伤害或者面临人身安全威胁，需要被紧急安置；

（七）法律规定的其他情形。

第九十三条　对临时监护的未成年人，民政部门可以采取委托亲属抚养、家庭寄养等方式进行安置，也可以交由未成年人救助保护机构或者儿童福利机构进行收留、抚养。

临时监护期间，经民政部门评估，监护人重新具备履行监护职责条件的，民政部门可以将未成年人送回监护人抚养。

第九十四条　具有下列情形之一的，民政部门应当依法对未成年人进行长期监护：

（一）查找不到未成年人的父母或者其他监护人；

（二）监护人死亡或者被宣告死亡且无其他人可以担任监护人；

（三）监护人丧失监护能力且无其他人可以担任监护人；

（四）人民法院判决撤销监护人资格并指定由民政部门担任监护人；

（五）法律规定的其他情形。

第九十五条　民政部门进行收养评估后，可以依法将其长期监护的未成年人交由符合条件的申请人收养。收养关系成立后，民政部门与未成年人的监护关系终止。

第九十六条　民政部门承担临时监护或者长期监护职责的，财政、教育、卫生健康、公安等部门应当根据各自职责予以配合。

县级以上人民政府及其民政部门应当根据需要设立未成年人救助保护机构、儿童福利机构，负责收留、抚养由民政部门监护的未成年人。

第九十七条　县级以上人民政府应当开通全国统一的未成年人保护热线，及时受理、转介侵犯未成年人合法权益的投诉、举报；鼓励和支持人民团体、企业事业单位、社会组织参与建设未成年人保护服务平台、服务热线、服务站点，提供未成年人保护方面的咨询、帮助。

第九十八条　国家建立性侵害、虐待、拐卖、暴力伤害等违法犯罪人员信息查询系统，向密切接触未成年人的单位提供免费查询服务。

第九十九条　地方人民政府应当培育、引导和规范有关社会组织、社会工作者参与未成年人保护工作，开展家庭教育指导服务，为未成年人的心理辅导、康复救助、监护及收养评估等提供专业服务。

第七章　司法保护

第一百条　公安机关、人民检察院、人民法院和司法行政部门应当依法履行职责，保障未成年人合法权益。

第一百零一条　公安机关、人民检察院、人民法院和司法行政部门应当确定专门机构或者指定专门人员，负责办理涉及未成年人案件。办理涉及未成年人案件的人员应当经过专门培训，熟悉未成年人身心特点。专门机构或者专门人员中，应当有女性工作人员。

公安机关、人民检察院、人民法院和司法行政部门应当对上述机构和人员实行与未成年人保护工作相适应的评价考核标准。

第一百零二条　公安机关、人民检察院、人民法院和司法行政部门办理涉及未成年人案件，应当考虑未成年人身心特点和健康成长的需要，使用未成年人能够理解的语言和表达方式，听取未成年人的意见。

第一百零三条　公安机关、人民检察院、人民法院、司法行政部门以及其他组织和个人不得披露有关案件中未成年人的姓名、影像、住所、就读学校以及其他可能识别出其身份的信息，但查找失踪、被拐卖未成年人等情形除外。

第一百零四条　对需要法律援助或者司法救助的未成年人，法律援助机构或者公安机关、人民检察院、人民法院和司法行政部门应当给予帮助，依法为其提供法律援助或者司法救助。

法律援助机构应当指派熟悉未成年人身心特点的律师为未成年人提供法律援助服务。

法律援助机构和律师协会应当对办理未成年人法律援助案件的律师进行指导和培训。

第一百零五条　人民检察院通过行使检察权，对涉及未成年人的诉讼活动等依法进行监督。

第一百零六条　未成年人合法权益受到侵犯，相关组织和个人未代为提起诉讼的，人民检察院可以督促、支持其提起诉讼；涉及公共利益的，人民检察院有权提起公益诉讼。

第一百零七条　人民法院审理继承案件，应当依法保护未成年人的继承权和受遗赠权。

人民法院审理离婚案件，涉及未成年子女抚养问题的，应当尊重已满八周岁未成年子女的真实意愿，根据双方具体情况，按照最有利于未成年子女的原则依法处理。

第一百零八条　未成年人的父母或者其他监护人不依法履行监护职责或者严重侵犯被监护的未成年人合法权益的，人民法院可以根据有关人员或者单位的申请，依法作出人身安全保护令或者撤销监护人资格。

被撤销监护人资格的父母或者其他监护人应当依法继续负担抚养费用。

第一百零九条　人民法院审理离婚、抚养、收养、监护、探望等案件涉及未成年人的，可以自行或者委托社会组织对未成年人的相关情况进行社会调查。

第一百一十条　公安机关、人民检察院、人民法院讯问未成年犯罪嫌疑人、被告人，询问未成年被害人、证人，应当依法通知其法定代理人或者其成年亲属、所在学校的代表等合适成年人到场，并采取适当方式，在适当场所进行，保障未成年人的名誉权、隐私权和其他合法权益。

人民法院开庭审理涉及未成年人案件，未成年被害人、证人一般不出庭作证；必须出庭的，应当采取保护其隐私的技术手段和心理干预等保护措施。

第一百一十一条　公安机关、人民检察院、人民法院应当与其他有关政府部门、人民团体、社会组织互相配合，对遭受性侵害或者暴力伤害的未成年被害人及其家庭实施必要的心理干预、经济救助、法律援助、转学安置等保护措施。

第一百一十二条　公安机关、人民检察院、人民法院办理未成年人遭受性侵害或者暴力伤害案件，在询问未成年被害人、证人时，应当采取同步录音录像等措施，尽量一次完成；未成年被害人、证人是女性的，应当由女性工作人员进行。

第一百一十三条　对违法犯罪的未成年人，实行教育、感化、挽救的方针，坚持教育为主、惩罚为辅的原则。

对违法犯罪的未成年人依法处罚后，在升学、就业等方面不得歧视。

第一百一十四条　公安机关、人民检察院、人民法院和司法行政部门发现有关单位未尽到未成年人教育、管理、救助、看护等保护职责的，应当向该单位提出建议。被建议单位应当在一个月内作出书面回复。

第一百一十五条　公安机关、人民检察院、人民法院和司法行政部门应当结合实际，根据涉及未成年人案件的特点，开展未成年人法治宣传教育工作。

第一百一十六条　国家鼓励和支持社会组织、社会工作者参与涉及未成年人案件中未成年人的心理干预、法律援助、社会调查、社会观护、教育矫治、社区矫正等工作。

第八章　法律责任

第一百一十七条　违反本法第十一条第二款规定，未履行报告义务造成严重后果的，由上级主管部门或者所在单位对直接负责的主管人员和其他直接责任人员依法给予处分。

第一百一十八条　未成年人的父母或者其他监护人不依法履行监护职责或者侵犯未成年人合法权益的，由其居住地的居民委员会、村民委员会予以劝诫、制止；情节严重的，居民委员会、村民委员会应当及时向公安机关报告。

公安机关接到报告或者公安机关、人民检察院、人民法院在办理案件过程中发现未成年人的父母或者其他监护人存在上述情形的，应当予以训诫，并可以责令其接受家庭教育指导。

第一百一十九条　学校、幼儿园、婴幼儿照护服务等机构及其教职员工违反本法第二十七条、第二十八条、第三十九条规定的，由公安、教育、卫生健康、市场监督管理等部门按照职责分工责令改正；拒不改正或者情节严重的，对直接负责的主管人员和其他直接责任人员依法给予处分。

第一百二十条　违反本法第四十四条、第四十五条、第四十七条规定，未给予未成年人免费或者优惠待遇的，由市场监督管理、文化和旅游、交通运输等部门按照职责分工责令限期改正，给予警告；拒不改正的，处一万元以上十万元以下罚款。

第一百二十一条　违反本法第五十条、第五十一条规定的，由新闻出版、广播电视、电影、网信等部门按照职责分工责令限期改正，给予警告，没收违法所得，可以并处十万元以下罚款；拒不改正或者情节严重的，责令暂停相关业务、停产停业或者吊销营业执照、吊销相关许可证，违法所得一百万元以上的，并处违法所得一倍以上十倍以下的罚款，没有违法所得或者违法所得不足一百万元的，并处十万元以上一百万元以下罚款。

第一百二十二条　场所运营单位违反本法第五十六条第二款规定、住宿经营者违反本法第五十七条规定的，由市场监督管理、应急管理、公安等部门按照职责分工责令限期改正，给予警告；拒不改正或者造成严重后果的，责令停业整顿或者吊销营业执照、吊销相关许可证，并处一万元以上十万元以下罚款。

第一百二十三条　相关经营者违反本法第五十八条、第五十九条第一款、第六十条规定的，由文化和旅游、市场监督管理、烟草专卖、公安等部门按照职责分工责令限期改正，给予警告，没收违法所得，可以并处五万元以下罚款；拒不改正或者情节严重的，责令停业整顿或者吊销营业执照、吊销相关许可证，可以并处五万元以上五十万元以下罚款。

第一百二十四条　违反本法第五十九条第二款规定，在学校、幼儿园和其他未成年人集中活动的公共场所吸烟、饮酒的，由卫生健康、教育、市场监督管理等部门按照职责分工责令改正，给予警告，可以并处五百元以下罚款；场所管理者未及时制止的，由卫生健康、教育、市场监督管理等部门按照职责分工给予警告，并处一万元以下罚款。

第一百二十五条　违反本法第六十一条规定的，由文化和旅游、人力资源和社会保障、市场监督管理等部门按照职责分工责令限期改正，给予警告，没收违法所得，可以并处十万元以下罚款；拒不改正或者情节严重的，责令停产停业或者吊销营业执照、吊销相关许可证，并处十万元以上一百万元以下罚款。

第一百二十六条　密切接触未成年人的单位违反本法第六十二条规定，未履行查询义务，或者招用、继续聘用具有相关违法犯罪记录人员的，由教育、人力资源和社会保障、市场监督管理等部门按照职责分工责令限期改正，给予警告，并处五万元以下罚款；拒不改正或者造成严重后果的，责令停业整顿或者吊销营业执照、吊销相关许可证，并处五万元以上五十万元以下罚款，对直接负责的主管人员和其他直接责任人员依法给予处分。

第一百二十七条　信息处理者违反本法第七十二条规定，或者网络产品和服务提供者违反本法第七十三条、第七十四条、第七十五条、第七十六条、第七十七条、第八十条规定的，由公安、网信、电信、新闻出版、广播电视、文化和旅游等有关部门按照职责分工责令改正，给予警告，没收违法所得，违法所得一百万元以上的，并处违法所得一倍以上十倍以下罚款，没有违法所得或者违法所得不足一百万元的，并处十万元以上一百万元以下罚款，对直接负责的主管人员和其他责任人员处一万元以上十万元以下罚款；拒不改正或者情节严重的，并可以责令暂停相关业务、停业整顿、关闭网站、吊销营业执照或者吊销相关许可证。

第一百二十八条　国家机关工作人员玩忽职守、滥用职权、徇私舞弊，损害未成年人合法权益的，依法给予处分。

第一百二十九条　违反本法规定，侵犯未成年人合法权益，造成人身、财产或者其他损害的，依法承担民事责任。

违反本法规定，构成违反治安管理行为的，依法给予治安管理处罚；构成犯罪的，依法追究刑事责任。

第九章　附 则

第一百三十条　本法中下列用语的含义：

（一）密切接触未成年人的单位，是指学校、幼儿园等教育机构；校外培训机构；未成年人救助保护机构、儿童福利机构等未成年人安置、救助机构；婴幼儿照护服务机构、早期教育服务机构；校外托管、临时看护机构；家政服务机构；为未成年人提供医疗服务的医疗机构；其他对未成年人负有教育、培训、监护、救助、看护、医疗等职责的企业事业单位、社会组织等。

（二）学校，是指普通中小学、特殊教育学校、中等职业学校、专门学校。

（三）学生欺凌，是指发生在学生之间，一方蓄意或者恶意通过肢体、语言及网络等手段实施欺压、侮辱，造成另一方人身伤害、财产损失或者精神损害的行为。

第一百三十一条　对中国境内未满十八周岁的外国人、无国籍人，依照本法有关规定予以保护。

第一百三十二条　本法自2021年6月1日起施行。
