# Supplementary material for: Mapping the evolution of fertility support policies in China: A content and instrumental analysis
Source: PLoS One. 2025 Oct 9;20(10):e0332137. doi: 10.1371/journal.pone.0332137 (PMC12510515; doi:10.1371/journal.pone.0332137)
Supplement: S1 Appendix — (ZIP) [file pone.0332137.s001.zip › S1 Appendix. 226 original policy documents/199-“十四五”公共服务规划.docx]

“十四五”公共服务规划

序言

公共服务关乎民生，连接民心。“十三五”时期，在以习近平同志为核心的党中央坚强领导下，我国公共服务体系日益健全完善，基本民生底线不断筑牢兜实，公共服务供给水平全面提升，多层次多样化需求得到更好满足。“十四五”时期，推动公共服务发展，健全完善公共服务体系，持续推进基本公共服务均等化，着力扩大普惠性非基本公共服务供给，丰富多层次多样化生活服务供给，是落实以人民为中心的发展思想、改善人民生活品质的重大举措，是促进社会公平正义、扎实推动共同富裕的应有之义，是促进形成强大国内市场、构建新发展格局的重要内容，对增强人民群众获得感、幸福感、安全感，促进人的全面发展和社会全面进步，具有十分重要的意义。

本规划依据《中华人民共和国国民经济和社会发展第十四个五年规划纲要》编制，主要涵盖幼有所育、学有所教、劳有所得、病有所医、老有所养、住有所居、弱有所扶、优军服务保障和文体服务保障等领域的公共服务。本规划是“十四五”时期乃至更长一段时期促进公共服务发展的综合性、基础性、指导性文件。规划期为2021—2025年。

需要说明的是，从服务供给的权责分类来看，公共服务包括基本公共服务、普惠性非基本公共服务两大类。其中，基本公共服务是保障全体人民生存和发展基本需要、与经济社会发展水平相适应的公共服务，由政府承担保障供给数量和质量的主要责任，引导市场主体和公益性社会机构补充供给。非基本公共服务是为满足公民更高层次需求、保障社会整体福利水平所必需但市场自发供给不足的公共服务，政府通过支持公益性社会机构或市场主体，增加服务供给、提升服务质量，推动重点领域非基本公共服务普惠化发展，实现大多数公民以可承受价格付费享有。此外，为满足公民多样化、个性化、高品质服务需求，一些完全由市场供给、居民付费享有的生活服务，可以作为公共服务体系的有益补充，政府主要负责营造公平竞争的市场环境，引导相关行业规范可持续发展，做好生活服务与公共服务衔接配合。随着我国经济社会发展水平的不断提升，基本公共服务、非基本公共服务与生活服务之间的边界也将随之发生变化，公共服务体系的范围、水平和质量都将稳步有序提升，不断满足人民群众日益增长的美好生活需要。

第一章规划背景

我国进入新发展阶段，公共服务发展基础更加坚实，发展条件深刻变化，进一步发展面临新的机遇和挑战。

第一节发展基础

城乡区域基本公共服务均等化水平不断提高。基本公共服务制度体系更加健全，建立了基本公共服务清单制度，出台了国家《基本公共服务标准（2021年版）》，明确了国家向全民提供基本公共服务的底线范围，为政府履行职责和公民享有相应权利提供了依据。基本公共服务资源持续向基层、农村、边远地区和困难群众倾斜，城乡区域人群间基本公共服务差距不断缩小。中西部地区公共服务设施条件明显改善，部分指标逐步追平东部地区。城乡之间制度性差异明显减少，实现了“新农合”与城镇居民医保制度并轨运行，全面建立统一的城乡居民医保制度，统筹城乡的居民基本养老保险制度逐步健全。基本公共服务逐步覆盖全部城镇常住人口，截至2020年，全国96.8%的县级单位实现义务教育基本均衡发展，85.8%的进城务工人员随迁子女在公办学校就读或者享受政府购买学位的服务。农村基本公共服务供给持续改善，教育扶贫、健康扶贫、农村危房改造在助力打赢脱贫攻坚战中发挥了积极作用。

公共服务供给保障能力全面提升。全国一般公共预算安排的基本公共服务领域支出持续增加，重点领域服务保障能力明显增强。到“十三五”末，覆盖全学段的学生资助政策体系更加完善，普惠性幼儿园覆盖率达到84.7%，九年义务教育巩固率达到95.2%、大班额基本消除，高中阶段教育毛入学率达到91.2%，高等教育毛入学率54.4%，进入普及化发展阶段。全民健康保障能力显著提升，每千人口医疗卫生床位数达到6.5张，每千人口拥有执业（助理）医师数达到2.9人。公共文化体育设施更加完善，每万人口拥有公共文化设施建筑面积达到444.1平方米，人均体育场地面积2.2平方米，公共体育服务网络基本形成。养老服务能力加快提升，全国养老机构和设施总数达到31.9万个，养老服务床位数达到823.8万张。婴幼儿照护服务加快发展，每千人口拥有3岁以下婴幼儿托位数1.8个。困难残疾人生活补贴涉及人数为1212.6万人，重度残疾人护理补贴涉及人数为1473.8万人。公租房保障能力增强，城镇低保、低收入住房困难家庭基本实现应保尽保。帮助和支持2568万贫困人口、3500多万边缘贫困群体住上安全住房，农村贫困群众住房安全得到历史性解决。生活服务快速发展。高端医疗、文化、旅游、体育、家政等服务逐渐成为广大人民群众服务消费的重要组成部分，生活服务取得长足发展。2019年文化及相关产业增加值为44363亿元，占GDP比重由2013年的3.69%提高到4.5%。2019年全国旅游业总收入达到6.6万亿元，国内游客达到60亿人次。2019年体育产业总规模达到2.95万亿元，增加值达到1.12万亿元。家政服务业加速提质扩容，2019年全国家政服务业营业收入达6900亿元，家政

从业人员达到3100万人。生活服务新业态、新模式不断涌现，朝数字化、网络化、智能化、多元化、协同化方向发展。人民生活得到显著改善。幼有所育、学有所教、劳有所得、病有所医、老有所养、住有所居、弱有所扶取得新进展、新成效。截至2020年，劳动年龄人口平均受教育年限达到10.8年，义务教育普及程度达到世界高收入国家平均水平。建成世界上规模最大的社会保障体系，参加基本医疗保险人数13.6亿人，基本养老保险参保人数达到9.99亿人。2019年，人均预期寿命达到77.3岁，主要健康指标已经总体上优于中高收入国家平均水平。建成世界最大住房保障体系，帮助2亿多城镇困难群众改善了住房条件。新冠肺炎疫情防控取得重大战略成果，应对突发事件的公共服务能力和水平大幅提高。

第二节机遇挑战

“十四五”时期是我国全面建成小康社会、实现第一个百年奋斗目标之后，乘势而上开启全面建设社会主义现代化国家新征程、向第二个百年奋斗目标进军的第一个五年。人民群众日益增长的美好生活的需要对公共服务体系提出了新的更高要求。国际国内环境的深刻变化，世界正在经历百年未有之大变局，不稳定性不确定性明显增加，对公共服务发展既是机遇也是挑战。

——我国社会主要矛盾已经转化为人民日益增长的美好生活需要和不平衡不充分的发展之间的矛盾，人民群众对美好生活更加向往，教育、医疗、养老、托育等公共服务保障水平成为影响人民群众获得感、幸福感、安全感的重要因素。

——我国经济已转向高质量发展阶段，经济长期向好，发展韧性强劲，转型升级潜力足，内需空间广阔，使得公共服务加快发展、人民生活持续改善的物质基础日趋雄厚。

——人口结构持续变迁，老龄化程度进一步加深，家庭结构小型化趋势明显，人员流动更加频繁，人民群众生存发展对公共服务的依赖性逐渐增强。

——新一轮科技革命深入发展，大数据、云计算、人工智能、物联网、区块链等新技术手段涌现，科技助推公共服务发展能力越来越强。

面对新形势、新挑战，我国公共服务发展不平衡不充分的问题仍然比较突出。基本公共服务仍存短板弱项，区域间、城乡间、人群间的基本公共服务仍有差距，均等化水平尚待进一步提高。非基本公共服务供给不足，优质资源总体短缺，扩供给促普惠仍需下更大功夫。公共服务资源配置机制不尽完善，设施布局与人口分布匹配不够，服务效能有待提高。

必须深刻认识环境变化带来的新机遇新挑战，树立底线思维，增强机遇意识和风险意识，准确识变、科学应变、主动求变，善于在危机中育先机、于变局中开新局，攻坚克难、改革创新，推动公共服务体系建设取得新突破，迈上新台阶，进一步彰显中国特色社会主义制度优越性。

第二章总体思路

“十四五”时期，必须明确指导思想，把握基本原则，锚定发展目标，扎实推动公共服务高质量发展。

第一节指导思想

高举中国特色社会主义伟大旗帜，以习近平新时代中国特色社会主义思想为指导，深入贯彻党的十九大和十九届二中、三中、四中、五中、六中全会精神，坚持以人民为中心的发展思想，立足新发展阶段，完整、准确、全面贯彻新发展理念，构建新发展格局，以推动高质量发展为主题，树立系统观念，强化底线思维，牢牢抓住人民群众最关心最直接最现实的民生问题，科学合理界定基本公共服务与非基本公共服务范围，正确处理政府和市场关系，持续推进基本公共服务均等化，多元扩大普惠性非基本公共服务供给，丰富多层次多样化生活服务供给，切实兜牢基本民生保障底线，稳步提升公共服务保障水平，不断满足人民群众美好生活需要，努力增进全体人民的获得感、幸福感、安全感，促进人的全面发展和社会全面进步，推动全体人民共同富裕迈出坚实步伐。

第二节基本原则

界定科学、权责清晰。坚持社会效益优先，突出社会公平，科学界定基本和非基本公共服务范围，明确政府和社会、个人的权责边界，突出政府在基本公共服务保障中的主体地位，合理增加公共消费，保持适当的民生支出力度和效度，保障民生改善的

稳定性和可持续性。

尽力而为、量力而行。充分考虑经济发展状况和财政负担能力，既要关注回应群众呼声，统筹各渠道资源，稳妥有序提升公共服务保障水平，又要合理引导社会预期，不吊高胃口、不过度承诺。新增公共服务事项要加强事前论证和风险评估，实现公共服务保障水平与经济社会发展水平“同频共振”。

政府主导、分类施策。统筹有效市场和有为政府作用，强化各级政府对基本公共服务供给的兜底责任，不断织密民生保障网，立足社会公平持续推进基本公共服务均等化。发挥政府引导作用，优化资源配置，吸引社会参与，不断扩大普惠性非基本公共服务供给。充分发挥市场机制作用，加强标准化品牌化建设，鼓励生活服务高品质多样化升级。

多元参与、共建共享。厘清政府权责边界，强化政府基本公共服务兜底保障职责。进一步放宽市场准入，放管结合，支持社会力量参与公共服务，发挥好各类企事业单位、协会商会、公益团体等市场主体和社会组织的作用。调动群众自我管理自我服务的积极性，广泛参与公共服务。形成政府、社会、个人协同发力、共建共享的公共服务发展格局。

第三节主要目标

到2025年，公共服务制度体系更加完善，政府保障基本、社会多元参与、全民共建共享的公共服务供给格局基本形成，民生福祉达到新水平。

基本公共服务均等化水平明显提高。国家基本公共服务制度更加完善，国家基本公共服务标准得到有效落实，标准化手段得到普及应用，基本公共服务实现目标人群全覆盖、服务全达标、投入有保障，地区、城乡、人群间的基本公共服务供给差距明显缩小，实现均等享有、便利可及。

普惠性非基本公共服务实现提质扩容。紧扣人民群众“急难愁盼”的突出问题，坚持社会效益优先，普惠性非基本公共服务数量和质量都得到较大提升，服务内容更加丰富、获取方式更加便捷、供给主体更加多元，推动普惠性非基本公共服务付费可享有、价格可承受、质量有保障、安全有监管，逐步实现幼有善育、学有优教、劳有厚得、病有良医、老有颐养、住有宜居、弱有众扶。

生活服务高品质多样化升级。适应人民群众多样化、个性化、高品质的健康、养老、托育、文化、旅游、广电、体育、家政等服务需求，生活服务标准化、品牌化建设取得重大突破，产业规模明显扩大，新业态、新模式不断涌现，在增加国内消费、扩大有效需求的同时，为今后公共服务提质升级蓄势储能，逐步形成需求牵引供给、供给创造需求的更高水平的动态平衡。

第三章推进基本公共服务均等化

享有基本公共服务是公民的基本权利，保障人人享有基本公共服务是政府的重要职责。坚持以促进机会均等为核心，推动实现全体公民都能公平可及地获得大致均等的基本公共服务。

第一节推进基本公共服务标准体系建设

健全完善基本公共服务标准体系。统筹经济社会发展水平和财政承受能力等因素，围绕“幼有所育、学有所教、劳有所得、病有所医、老有所养、住有所居、弱有所扶、优军服务有保障、文体服务有保障”的民生保障目标，完善国家基本公共服务标准，明确基本公共服务项目的服务对象、服务内容、服务标准、牵头负责单位及支出责任，作为各地区提供基本公共服务的基准和人民群众享有相应权利的重要依据。各地区对照国家基本公共服务标准，细化地方具体实施配套标准，对有国家统一标准的基本公共服务项目，应按照不低于国家标准执行，确保内容无缺项、目标人群全覆盖、标准不高攀、投入有保障、服务可持续。统筹设施建设、设备配置、人员配备、服务管理等软硬件标准要素，完善重点行业领域标准规范，加强各行业标准间的统筹衔接。推动基层服务机构标准化管理。

推动基本公共服务达标。加强对国家基本公共服务标准实施情况监测预警，强化实施效果反馈利用，推动国家基本公共服务标准落地落实。逐步建立具备查询、公开、宣传、共享等一体化功能的基本公共服务标准信息资源库，集中公开各级各类基本公共服务标准，并逐步将基本公共服务标准信息资源库纳入全国公共数据服务体系，加快推进数据深度挖掘与共享开放。开展重点领域基本公共服务标准化工程。加快公共教育、社会保险、公共文化体育、残疾人服务等重点领域国家标准、行业标准制修订，建立与国家基本公共服务标准相配套的支撑标准体系，强化标准信息公开与实施推广，持续开展国家基本公共服务标准化试点。适应国家人口发展战略和适龄儿童变化情况，制定完善义务教育学位配置标准，保障足够的公办学校学位供给，健全校园校舍、师资队伍、教学装备、经费保障等办学条件标准化推进机制，确保“两免一补”等资助政策城乡学生全覆盖。研究提出残疾人服务标准体系，开展残疾人康复、托养照护、就业服务、无障碍和残疾人服务资源管理、信息化服务平台建设等方面的标准试点。组织开展人社领域公共服务标准化试点、社会保险等领域专项标准试点，持续优化社会保险标准化体系，推动全国范围内社保经办服务标准化供给。制定国家基本养老服务标准，科学界定基本养老服务对象，细化服务项目和标准，完善设施建设、功能布局、设备配置、人员配备、服务流程、管理规范等软硬件标准和质量要求。细化完善全民健身基本公共服务标准体系，建立全民健身基本公共服务跟踪评估和监督反馈机制，推动全民健身基本公共服务全覆盖。推动基本公共服务标准动态调整常态化、制度化。按照稳妥有序、论证充分的原则，在保持国家基本公共服务范围和标准总体稳定的基础上，结合经济社会发展情况、兼顾财政承受能力，适时对国家基本公共服务标准进行动态调整。

第二节补齐基本公共服务短板

对标对表国家基本公共服务标准，结合地方实施标准，采取针对性更强、覆盖面更广、作用更直接、效果更明显的举措，促进公共服务资源向基层延伸、向农村覆盖、向边远地区和生活困难群众倾斜，加快补齐基本公共服务的软硬件短板弱项。

义务教育。加强教师队伍建设，依法保障教师工资收入水平，落实教职工编制标准和统筹管理规定，确保教职工编制全面达标。统筹教师编制配置和跨区调整，推进“县管校聘”管理改革，推动县（区）域内义务教育校长教师交流轮岗，促进优秀骨干教师在学校间均衡配置。继续实施农村义务教育阶段教师特岗计划、“三区”人才支持计划、教师专项计划和银龄讲学计划，落实好乡村教师生活补助政策。科学规划城乡义务教育学校（含特殊教育学校，下同）布局，推进义务教育学校标准化建设，改善乡村小规模学校和乡镇寄宿制学校条件，合理有序扩大城镇义务教育学校学位供给。

就业社保。制定实施公共就业创业服务设施、设备配置、人员配备、服务规范等指导性标准，加强基层公共就业创业服务平台建设。鼓励依托公共实训基地，加大对新型农民创新创业支持力度。在欠发达地区特别是脱贫地区、易地扶贫搬迁大型安置区建设公共就业服务机构，提供全方位公共就业服务。加快推动公共就业创业服务数字化转型，打造集政策解读推送、业务办理咨询于一体的线上智能服务、线下自助服务体系。完善流动人员人事档案管理服务设施。提升社会保险经办能力，推进社保转移接续，完善全国统一的社会保险公共服务平台。推动灵活就业人员在就业地参加社会保险，实现法定人群全覆盖。推进失业保险、工伤保险向职业劳动者广覆盖，实现失业保险省级统筹，工伤保险省级统筹更加完善。加强劳动保障监察执法维权服务能力，切实提高监察执法效能。

医疗卫生。扩大医护人员特别是儿科、全科、麻醉科、精神科、老年医学科等短缺医师和注册护士规模，提升医护人员培养质量。多途径培养培训乡村医疗卫生工作队伍，改善乡村卫生服务水平。以城市社区和农村基层、边境口岸城市、县级医院为重点，完善城乡医疗服务网络。加强社区卫生服务中心(站)、乡镇卫生院和村卫生室的标准化建设，加大基层中医药人才培养力度，鼓励引导基层医疗卫生机构提供适宜的中医药服务。加强妇幼保健、传染病、精神病等诊疗能力建设。以儿科、全科、精神科等紧缺专业为重点，加强住院医师规范化培训基地建设。完善医疗保障经办管理服务网络，推进标准化、信息化建设，提升基层医疗保障经办服务能力建设。健全基本医疗保险稳定可持续筹资和待遇调整机制，完善医疗保险缴费参保政策。做实基本医疗保险市级统筹，推动省级统筹。完善职工基本医疗保险门诊共济保障机制，健全重大疾病医疗保险和救助制度。完善跨省异地就医直接结算制度体系，加强国家、省级异地就医结算中心建设和跨区域业务协作，全面提升管理服务能力。将符合条件的互联网医疗服务纳入医保支付范围。健全公共卫生应急管理体系，完善重大疫情防控体制机制，提升疫情监测预警能力，提高应对突发重大公共卫生事件的能力和水平。

养老服务。加强乡镇（街道）范围内具备综合功能的养老服务机构建设。加强公办养老机构建设，落实新建城区、居住(小)区按照人均不少于0.1平方米的标准配建养老服务设施，稳步提高护理型床位占比。实施特困人员供养服务设施和服务质量达标工程，提升特困人员供养服务机构托底保障能力。鼓励有条件的特困人员供养服务设施(敬老院)在满足特困人员集中供养需求的前提下，逐步为最低生活保障家庭、最低生活保障边缘家庭、重度残疾人家庭、计划生育特殊家庭和原建档立卡贫困家庭中的老年人，提供低偿或无偿的集中托养服务。健全养老服务培训机制，开展养老服务人才培训提升行动，壮大养老护理员、老年社会工作者队伍。逐步提升老年人福利水平，完善经济困难高龄失能老年人补贴制度。健全养老保险制度体系，促进基本养老保险基金长期平衡。实现企业职工基本养老保险全国统筹。完善城镇职工基本养老金合理调整机制，适时调整城乡居民基础养老金标准。

住房保障。做好城镇住房和收入困难家庭公租房保障，实行实物保障和货币补贴并举，合理确定实物公租房保有量，对城镇户籍低保、低收入住房困难家庭依申请应保尽保。稳步推进棚户区改造，坚持因地制宜、量力而行，严格把好棚户区改造范围和标准，科学确定棚户区改造年度计划，重点改造老城区内脏乱差的棚户区，加强配套基础设施建设和工程质量安全监管，加快工程进度和回迁安置。结合农村危房改造，对符合条件的农村低收入群体等重点对象住房安全做到应保尽保。

文化体育。推进城乡公共文化服务体系一体建设。充分利用现有城乡公共设施，统筹建设基层综合文化服务中心。以县级文化馆、图书馆为中心推进总分馆制建设，实现城乡社区公共文化服务资源整合和互联互通。推进全国智慧图书馆体系建设、公共文化云建设。提升农家书屋服务能力，推动农村电影放映优化升级。加强智慧广电基础设施建设，推进实施智慧广电固边工程和市级广电融合发展提升工程，推动应急广播体系建设和有线高清交互数字电视机顶盒推广普及，强化数字文化服务和流动文化服务，实施戏曲公益性演出项目。推动省市级电视台开设手语节目或加配字幕。加强县级公共体育场、健身步道、体育公园、农民体育健身工程等公共健身设施建设，合理利用体育中心、闲置厂房、校舍操场、社区空置场所等，拓展公共体育活动场所，有条件的公园绿地可建设非标准的健身场地设施。实施乡镇（街道）全民健身场地器材补短板工程。在社区推广社会体育指导员制度，开展全民健身志愿服务。支持全国爱国主义教育示范基地、博物馆、美术馆、公共图书馆、文化馆（站）等设施按规定免费或优惠开放，促进高等学校博物馆、美术馆等文化设施向社会开放。支持公共体育场馆免费或低收费开放。

社会服务。加强残疾人服务设施和综合服务能力建设，完善无障碍环境建设和维护政策体系，支持困难残疾人家庭无障碍设施改造，动态调整困难残疾人生活补贴和重度残疾人护理补贴标准。鼓励有条件的地方优先为经济困难的残疾学生提供免费的学前教育和高中教育，逐步实施残疾学生高中阶段免费教育。进一步推进儿童福利机构优化提质和创新转型高质量发展。加强县级、乡镇（街道）未成年人保护设施建设，重点支持留守儿童数量较多的欠发达地区相关设施建设。充分利用现有社会福利设施建设流浪乞讨人员救助设施或救助站，实现救助服务网络覆盖全部县（市、区）。加强精神卫生福利设施、公益性殡葬服务设施建设。加强退役军人服务中心（站）建设，提升优抚医院、光荣院等建设服务水平。提升基层社会救助经办服务能力，推动村级设立社会救助协理员，将走访、发现需要救助的困难群众列为村（社区）重要工作内容。加强公共法律服务平台建设，健全完善中西部边远地区法律援助机构设置，支持法治宣传阵地建设。加强法律援助专业人员培训，提升法律援助质量，优化法律援助人员资质。

第三节加快提升基本公共服务均等化水平

推动区域基本公共服务缩小差距。加大财政转移支付向特殊类型地区的倾斜力度，推进基本公共服务体系建设，完善地方基本公共服务支出保障机制，不断提高特殊类型地区基本公共服务供给水平。完善基本公共服务区域合作机制。鼓励具备条件的城市群、毗邻地区加强基本公共服务标准统筹，搭建区域内基本公共服务便利共享的制度安排。开展发达地区和欠发达地区基本公共服务在线对接，支持发展东西部线上对口帮扶、优质资源“1带N”、人才对口支援等方式，扩大优质服务资源辐射覆盖范围，缩小地区差距。完善城市群公共服务便利共享制度安排。加快城乡基本公共服务制度统筹。实施乡村建设行动，推进城乡基本公共服务标准统一、制度并轨，增加农村教育、医疗、

养老、文化等服务供给。结合户籍管理制度改革，健全以公民身份号码为标识、与居住年限相挂钩的非户籍人口基本公共服务提供机制，稳步实现基本公共服务由常住地供给、覆盖全部常住人口。落实农业转移人口市民化财政支持政策，完善异地结算、钱随人走等相关制度安排，保障符合条件的外来人口与本地居民平等享有基本公共服务。

优化基本公共服务对象认定制度。完善最低生活保障家庭、最低生活保障边缘家庭、特困人员认定办法。根据各地实际制定与当地经济社会发展水平相适应的最低生活保障家庭财产限定标准或条件，综合考虑居民人均消费支出或人均可支配收入等因素，结合财力状况动态调整。进一步完善和落实社会救助和保障标准与物价上涨挂钩的联动机制。将优抚对象优先纳入覆盖一般群众的救助、养老、医疗、住房以及残疾人保障等各项社会保障制度。进一步完善覆盖全学段的学生资助体系，加强教育、民政、乡村振兴等部门的数据比对和信息共享，按规定将符合条件的家庭经济困难学生纳入学生资助和社会救助范围，健全应助尽助机制，提升精准资助水平。推进公办养老机构入住综合评估制度，优先满足失能老年人的基本养老服务需求。研究做好老年人能力评估标准、长期护理保险失能等级评估标准、残疾军人和伤残民警残疾评定标准、职工工伤与职业病致残程度鉴定标准、国家残疾人残疾分类和分级标准等的衔接。

第四章扩大普惠性非基本公共服务供给

紧紧围绕供需矛盾突出的公共服务领域，发挥政府引导作用，鼓励支持社会力量重点加强养老、托育、教育、医疗等领域普惠性规范性服务供给，面向广大人民群众提供价格可负担、质量有保障的普惠性非基本公共服务。

第一节推动重点领域非基本公共服务扩容

发展普惠托育服务。着力构建多元化、多样化、覆盖城乡的婴幼儿照护服务体系，积极引导社会力量举办托育服务机构，将需要独立占地的婴幼儿照护服务设施和场地建设布局纳入相关规划，新建和改扩建一批服务设施，建成一批示范性服务机构。加强社区婴幼儿照护服务设施与社区服务中心（站）及社区卫生、文化、体育等设施的功能衔接，发挥综合效益。城镇婴幼儿照护服务建设要充分考虑农村进城务工人员随迁婴儿的照护服务需求。加大对农村和脱贫地区婴幼儿照护服务的支持力度，推广婴幼儿早期发展项目。推动儿童残疾筛查、诊断、康复救助有机衔接，提高残疾儿童早发现、早诊断、早干预、早康复能力和效果。构建覆盖城乡的家庭教育指导服务体系，为家庭提供更高质量更加精准的家庭教育指导，更好满足家长科学育儿的迫切需求。建立健全婴幼儿照护服务机构备案登记制度、信息公示制度和质量评估制度，对婴幼儿照护服务机构实施动态管理。依法逐步实行婴幼儿照护工作人员职业资格准入制度。推动学前教育普及普惠。健全普惠性学前教育保障机制，全面普及三年学前教育。继续实施第四期学前教育行动计划，补齐普惠性学前教育资源短板，提高幼儿园保教质量。根据适龄人口变化情况和城镇化发展趋势，完善县（市、区）普惠性幼儿园规划布局，及时修订和调整居住区人口配套学位标准，推动城市居住区、易地搬迁安置区配套建设与人口规模相适应的幼儿园。鼓励支持街道、村集体、国有企事业单位、普通高等学校举办公办园，扶持民办园提供普惠性服务，保障普惠性资源供给。完善农村学前教育资源布局，办好公办乡镇中心园，通过依托乡镇中心园举办分园、村独立或联合办园、巡回支教等方式满足农村适龄儿童入园需求。严格落实幼儿园教师持教师资格证上岗。大力加强幼儿园教师配备补充、工资待遇保障制度，提高教师待遇和社会地位。

加强县域普通高中建设。研究制定县域普通高中发展提升计划，全面加强县中建设，持续巩固提高高中阶段教育普及水平，促进高中阶段学校多样化有特色发展。全面化解普通高中大班额，加快消除大规模学校，积极改善办学薄弱环节，适应普通高中选课走班需要。健全教师补充激励机制，配齐配足教师，实施县中发展提升校长教师培训专项计划，提升县中教师能力素质。实施县中托管帮扶工程，加快提升县中整体办学水平。

积极发展普惠型养老服务。深入推进普惠养老专项行动，扩大普惠性养老服务供给。发展集中管理运营的社区养老服务网络，支持具备综合功能的社区服务设施建设，推动形成“15分钟”养老服务圈。引导专业化养老服务机构进社区、进家庭，提升家庭照护能力。大力发展政府扶得起、村里办得起、农民用得上、服务可持续的农村幸福院等互助养老设施。鼓励民间资本对企业厂房、商业设施及其他可利用的社会资源进行整合和改造后用于养老服务。推动培训疗养资源转型发展普惠养老服务。允许养老机构依法依规设立多个服务网点，实现规模化、连锁化、品牌化运营。全面保障外资举办养老服务机构享受国民待遇。探索具备条件的公办养老机构改制为国有养老服务企业。

均衡发展优质医疗服务。聚焦重点人群健康需求，提升全方位全生命周期健康服务与保障能力，促进医疗卫生服务公平可及、系统连续。依托综合医院、职业病专科医院，加强尘肺病、化学中毒等职业病诊断救治康复能力建设，增强职业健康服务可及性便利性。鼓励发展全科医疗服务，加快发展专业化服务，有序发展前沿医疗服务，推动医养结合等多业态融合服务发展。坚持中西医并重和优势互补，大力发展中医药服务，充分发挥中医药在疾病预防、治疗、康复中的独特作用。推进区域医疗中心建设和临床专科能力建设。在医疗资源不足地区，坚持“按重点病种选医院、按需求选地区，院地合作、省部共建”的思路进一步推进区域医疗中心试点建设，通过建设高水平医院分中心、分支机构和“一院多区”等方式，定向放大国家顶级优质医疗资源。支持省级区域医疗中心建设，积极发展医疗联合体，加强智慧医院建设，发展远程医疗服务，增强省级区域医疗中心的辐射服务能力。推动县级医院提标扩能，加强胸痛、卒中、创伤、危重孕产妇、危重新生儿和儿童等救治中心以及肿瘤综合治疗中心、慢性病管理中心建设。

积极推动改善住房条件。人口净流入的大城市要大力发展保障性租赁住房，主要解决符合条件的新市民、青年人等群体的住房困难问题，以建筑面积不超过70平方米的小户型为主，租金低于同地段同品质市场租赁住房租金。人口净流入的大城市因地制宜发展共有产权住房，以中小户型为主，供应范围以面向户籍人口为主，逐步扩大到常住人口。全面推进城镇老旧小区改造，重点改造完善小区配套和市政基础设施，提升社区养老、托育、医疗等公共服务水平，推动建设安全健康、设施完善、管理有序的完整居住社区。扩大住房公积金制度覆盖范围，多措并举促进单位依法缴存，鼓励灵活就业人员参加住房公积金制度。优化住房公积金使用政策，租购并举保障缴存人基本住房需求。

第二节推动非基本公共服务普惠化发展

降低服务成本。统筹用好规划、土地、投资、税收、金融等多种支持政策，盘活现有设施资源，低价或无偿提供给普惠性非基本公共服务供给主体，帮助降低服务成本、提升运营效率，扩大服务供给。

促进价格普惠。按照保本微利、优质优价、节约资源、公平负担的原则，加快理顺公共服务价格，引导非基本公共服务供给主体提供与当地城乡居民收入水平相适应的普惠性非基本公共服务，遏制过度逐利行为。依据成本变化、居民收入等情况，健全非基本公共服务价格调整机制，及时公开披露项目运行等信息。

加强质量监管。探索包容而有效的审慎监管方式，推动制修订相关法律法规和标准规范，加强服务质量监督监测，构建责任清晰、多元参与、依法监管的服务质量治理和促进体系，提高服务质量。强化政府事中事后监管能力，实行监督检查结果公开、质量安全事故强制报告、质量信用记录、严重失信服务主体强制退出等制度。健全公共服务机构评审评价体系，鼓励开展第三方服务认证，推行服务承诺和服务公约制度。发挥社会监督作用，拓宽公众参与监管的渠道，推广服务质量社会监督员制度，鼓励第三方服务质量调查。

第五章推动生活服务为公共服务提档升级拓展空间

适应人民群众需求增长和消费升级趋势，培育壮大市场主体，增加服务供给，强化服务标准，做大服务品牌，优先发展能够与公共服务密切配合、有序衔接的高品质多样化生活服务，推动生活服务与公共服务互嵌式、阶梯式发展，为公共服务提档升级探索方向、拓展空间、积蓄能量。

第一节推进重点行业创新融合发展

医疗卫生服务提质增效。鼓励发展专业性医院管理集团。支持发展医学检验等第三方医疗服务，推动检验检查结果互认，鼓励发展第三方医疗服务评价。推动精准医疗等新兴服务发展。鼓励支持医疗康复、健康管理、心理咨询、中医药养生保健等服务发展。以高端医疗、康复疗养、休闲养生为核心，丰富健康旅游产品。积极发展智慧医疗，鼓励医疗机构提升信息化、智能化水平，支持健康医疗大数据资源开发应用。丰富商业健康保险产品，大力发展医疗责任险、医疗意外险等执业保险。推动辅助器具产业发展。

养老服务高质量发展。促进养老服务与文化、旅游、体育、家政、健康等行业融合发展。培育满足老年人需求的健康产品专业化生产研发基地，促进养老企业连锁化、集团化发展，形成一批产业链长、覆盖领域广、经济社会效益显著的产业集群和集聚区。积极培育养老服务行业组织，支持行业协会增强服务能力，发挥推进养老服务业高质量发展的积极作用。文化旅游融合发展。坚持以文塑旅、以旅彰文，打造独具魅力的中华文化旅游体验。深入发展大众旅游、智慧旅游，创新旅游产品体系，改善旅游消费体验。加强区域旅游品牌和服务整合，建设一批富有文化底蕴的世界级旅游景区和度假区，打造一批文化特色鲜明的国家级旅游休闲城市和街区。推进红色旅游、文化遗产旅游、旅游演艺等创新发展，提升度假休闲、乡村旅游等服务品质，完善邮轮游艇、低空旅游等发展政策。健全旅游基础设施和集散体系，推进旅游厕所革命，强化智慧景区建设。加强旅游景区疫情防控，避免发生聚集性感染和疫情扩散。建设旅游服务质量评价体系，规范在线旅游经营服务。

智慧广电创新发展。壮大广播电视节目栏目、电视剧、动画片、纪录片、网络剧、短视频、网络电影等产业，打造高新视听产业基地，拓展衍生产品市场。加快电视频道高清化改造，提升超高清电视节目制播能力，推进互动视频、沉浸式视频、虚拟现实视频、云游戏等高清视频和云转播应用，改善内容消费体验。建设全媒体传播体系，推广互动式、服务式、场景式传播，丰富移动智能终端呈现形式，提升内容服务品质。推动县级融媒体中心建设。推进国家有线电视网络整合和5G一体化发展，提升内容服务和业务承载能力。依托广电5G网络发展5G广播电视，开通广播电视和公共安全应急服务，推动广播电视终端通、移动通、人人通。促进智慧广电参与数字社会、数字政府、数字乡村建设。完善视听全产业链发展格局，加快培育新型业态、新型消费模式。

体育服务加快发展。倡导全民健身，鼓励兴办多种形式的健身俱乐部和健身组织，加快发展健身休闲产业。繁荣发展足球、篮球、排球、冰雪、水上等运动，普及推广户外运动，推动体育竞赛表演产业发展，鼓励培育品牌赛事，丰富群众体育赛事活动，促进体育旅游、体育传媒、体育会展、体育经纪等发展。鼓励发展智能体育，培育体育消费新业态新模式。

家政服务提质扩容。加快建立供给充分、服务便捷、管理规范、惠及城乡的家政服务体系。实施家政服务业提质扩容“领跑者”行动，鼓励有条件的家政服务企业品牌化、连锁化发展，加快培育龙头企业，支持中小家政服务企业专业化、特色化发展。

提升家政服务规范化水平，加快建立家政服务人员持证上门制度，开展家政服务质量第三方认证。推进家政培训和就业服务。

第二节加强生活服务品牌化标准化建设

加强服务品牌培育。支持龙头企业做大做强，鼓励中小微企业创新发展，鼓励塑造代表性特色化服务品牌，保护传承“老字号”，开发打造“特字号”，培育壮大“新字号”。鼓励拥有优质资源的生活服务供给主体，通过合作、连锁经营等多种方式，跨地区设置服务网点、参与服务供给，共享先进服务技术和管理模式。鼓励银行业金融机构向企业提供以品牌为基础的商标权、专利权等质押贷款。支持服务企业拓展经营领域，促进跨界融合发展，鼓励发展体验服务、私人订制、共享服务、智慧服务等新业态新模式。

强化服务标准建设。建立政府主导制定的标准与市场自主制定的标准协同发展、协调配套的服务标准体系，加快完善生活服务国家标准，开展国家级服务业标准化试点示范，以标准化促进服务质量提升。支持社会组织制定团体标准。鼓励企业制定高于国家标准或行业标准的企业标准，增强企业市场竞争力。健全生活服务认证认可制度，推动生活服务职业化发展。

第六章系统提升公共服务效能

紧扣服务设施布局、生产供给、服务享有、要素保障等关键环节，科学谋划、改革创新，系统推动公共服务提质增效，不断增强公共服务体系对国家重大战略实施的支持能力。

第一节统筹规划公共服务设施布局

科学设定服务半径和服务人口。公共服务设施建设选址应贴近服务对象，与服务半径和服务对象数量、年龄结构等因素有机衔接。幼儿园和小学、社区养老托育设施、卫生站（室）等服务频次高、服务对象活动能力弱的设施，应适度控制设施规模、合理安排设施密度。人员居住相对分散的偏远农村地区，因地制宜、统筹布局固定服务设施和流动服务设施，流动服务应明确服务时间和地点并保持相对稳定，提高农村居民享受公共服务的便利性。

合理控制公共服务设施规模。公共服务设施建设坚持功能优先、经济适用的原则，不宜盲目追求大规模的综合性设施。对于高频次服务设施，应适度减小规模、增加布点，通过总分馆（院）、连锁等多种方式形成服务合力，共享优质资源。对于服务频次相对较低或多个服务事项具有较强相关性的设施，应统筹考虑服务链条，适度集中布局，推广“只跑一次”等已有成功经验，简化办理流程。在鼓励应用现代信息技术提供服务便利性的同时，为老年人、残疾人等特殊人群保留必要的现场服务窗口。加强毗邻地区设施共建共享。配合区域协调发展战略、国家新型城镇化战略等实施，加强跨地区统筹协调，鼓励毗邻地区打破行政区划限制，统筹公共服务标准，互联互通相关信息数据，充分发挥地区比较优势，共建共享公共服务设施，为城乡居民就近享有公共服务提供便利条件。引导京津冀地区的通州与北三县、长三角生态绿色一体化发展示范区等区域积极探索共建共享经验和做法。

第二节构建公共服务多元供给格局

深化事业单位改革。加快推进政事分开、事企分开、管办分离，优化布局结构，完善制度机制，强化公益属性，提高治理效能，促进新时代公益事业平衡充分高质量发展。聚焦普惠性、基础性、兜底性公共服务需求，引导事业资源参与公共服务供给。统筹盘活用好沉淀和低效配置的事业编制资源，加大对人口集中流入地区统筹调剂力度，解决义务教育、基本医疗、公共文化等编制急需。原则上能够通过政府购买等方式提供的公共服务，不再直接举办事业单位提供。

鼓励社会力量参与。完善相关政策，放开放宽准入限制，推进公平准入，鼓励社会力量通过公建民营、政府购买服务、政府和社会资本合作（PPP）等方式参与公共服务供给。在资格准入、职称评定、土地供给、财政支持、政府采购、监督管理等方面公平对待民办与公办机构，及时清理和废除妨碍公平竞争的各种规定和做法。深化“放管服”改革，全面清理整合涉及社会力量进入公共服务领域的行政审批事项，整合公共服务机构设置、执业许可、跨区域服务等审批环节，优化审批流程，提高审批效率。支持社会组织发展。大力培育发展面向社区居民提供各类公共服务的社区组织。支持社区社会组织承接社区公共服务，开展社区志愿服务。逐步扩大政府向社会组织购买服务的范围和规模，对民生保障、社会治理、行业管理、公益慈善等领域的公共服务项目，同等条件下优先向社会组织购买。大力发展慈善组织，广泛动员志愿服务组织和志愿者参与公共服务提供，鼓励企事业单位提供公益慈善服务。完善激励保障措施，落实慈善捐赠的相关优惠政策，共同营造社会力量参与公共服务的良好环境。发挥国有经济作用。进一步明确国有经济参与公共服务的领域和条件，推动国有资本在提供公共服务、应急能力建设和公益性服务等领域发挥更大作用。支持参与公共服务的国有企业壮大产业集团、做大做强品牌，重点培育发展一批实力雄厚、具有较强竞争力和影响力的大型社会服务企业和企业集团。鼓励和引导国有经济以兼并、收购、参股、合作、租赁、承包等多种形式参与公共服务，拓宽国有经济进入渠道。

第三节提高公共服务便利共享水平

推进新技术创新应用。推动数字化服务普惠应用，充分运用大数据、云计算、人工智能、物联网、区块链等新技术手段，鼓励支持新技术赋能，为人民群众提供更加智能、更加便捷、更加优质的公共服务。促进“互联网+公共服务”发展，推动线上线下融合互动，支持高水平公共服务机构对接基层、边远和欠发达地区。促进人工智能在公共服务领域推广应用，鼓励支持数字创意、智慧就业、智慧医疗、智慧住房公积金、智慧法律服务、智慧旅游、智慧文化、智慧广电、智能体育、智慧养老等新业态新模式发展。促进公共服务与互联网产业深度融合发展，大力培育跨行业跨领域综合性平台和行业垂直平台。探索“区块链+”在公共服务领域的运用。加快信息无障碍建设，切实解决老年人等特殊群体在运用智能技术方面遇到的突出困难，帮助老年人、残疾人等共享数字生活。充分发挥全国一体化政务服务平台一网通办枢纽作用，推动更多公共服务事项网上办、掌上办、一次办，持续提升公共服务数字化智能化水平。

推动服务数据互联互通。探索实施民生档案跨区查档服务项目，建立互认互通的档案专题数据标准体系。推进数字政府建设，强化教育、医疗卫生、社会保障、社会服务等重点领域数据信息交换共享，加快实现民生保障事项“一地受理、一次办理”。加强部门间信息共享和证明互认，通过完善信用监管、全面推进告知承诺制等方式，实施证明事项清单管理制度，减少不必要的证明事项。加强地区间的信息互联互通，积极推进残疾人“两项补贴”跨省通办、社会关系转移接续、流动人员人事档案信息化管理、异地就医结算等便利服务。逐步建立以社会保障卡为载体的居民服务“一卡通”。建立健全政府及公共服务机构数据开放共享规则，在加强公共服务数据安全保障和隐私保护的前提下，推动医疗卫生、养老等公共服务领域和政府部门数据有序开放。

推动服务重心向基层下沉。在明确服务标准规范的基础上，强化街道、乡镇和社区的基本公共服务职能，加强基层人财物保障力度，持续改善各类公共服务设施条件，推动基层综合公共服务平台统筹发展、共建共享。推进审批权限和公共服务事项向基层延伸，推动医疗卫生、就业社保、养老托育、扶残助残、家政服务、物流商超、治安执法、纠纷调处、心理援助等便民服务场景有机集成和精准对接。推动基本公共服务与社会治理深度融合，实现社区综合服务中心等基层公共服务供给站点与以社区网格员为主体搭建的社会治理网络有机结合。培养专业化专职化的城乡社区工作者队伍。

第四节健全公共服务要素保障体系

完善财力保障制度。落实公共服务领域中央与地方财政事权和支出责任划分改革要求，优化财政支出结构，加大中央和省级财政对基层政府提供基本公共服务的财力支持力度。将更多公共服务项目纳入政府购买服务指导性目录，完善财政、融资和土地等配套优惠政策。规范购买流程，按照政府采购法律制度规定确定承接主体，实行竞争择优、费随事转。进一步完善政府购买公共服务的绩效管理。加大金融支持力度，综合利用债券、保险、信贷等方式，为公共服务项目融资提供支持。强化人才队伍建设。进一步完善统一开放、竞争有序的人才资源市场，积极探索人才服务新模式，促进公共服务人才有序流动和合理配置。充分发挥高等学校、职业学校、科研院所作用，大力培养公共服务人才。健全公共服务从业人员教育培训制度，定期组织职业培训和业务轮训，提高公共服务专业化水平。探索公办与非公办公共服务机构在技术和人才等方面的合作机制，对非公办机构的人才培养、培训和进修等给予支持。加快农村公共服务和治理人才队伍建设。引导鼓励公共服务人才向中西部地区和基层流动。

保障设施用地需求。根据多层次多样化公共服务需求，优化土地供应调控机制，有效保障公共服务用地供给。将公共服务机构和设施用地纳入相关规划和年度用地计划并优先予以保障，农用地转用指标、新增建设用地指标分配要适当向公共服务机构和设施建设用地倾斜。在符合相关法律法规的前提下，鼓励利用低效土地、房屋建设公共服务机构和设施。符合条件的公共服务设施和机构建设用地，可采取划拨方式予以保障。探索实行长期租赁、先租后让、租让结合的弹性土地供应方式。优化资源配置机制。细化完善公共资源与常住人口挂钩、与服务半径挂钩的制度安排，提高公共服务的有效覆盖。逐步完善精准服务、主动响应的公共服务提供机制，实现从“人找服务”到“服务找人”的转变。逐项明晰公共服务标准及所需的软硬件标准规范，加强对公共服务供给水平和质量的有效评估监管，建立健全公共服务需求表达和反馈机制，并根据评估结果动态调整国家基本公共服务清单，不断完善公共服务资源配置。

第五节强化服务国家重大战略能力

促进人口长期均衡发展。提高优生优育服务水平，发展普惠托育服务体系，推进教育公平与优质教育资源供给，降低家庭教育开支。完善生育休假与生育保险制度，加强税收、住房等支持政策，保障女性就业合法权益。对全面两孩政策调整前的独生子女家庭和农村计划生育双女家庭，继续实行现行各项奖励扶助制度和优惠政策。建立健全计划生育特殊家庭全方位帮扶保障制度，完善政府主导、社会组织参与的扶助关怀工作机制，维护好计划生育家庭合法权益。

助力城乡区域协调发展。健全城市群公共服务便利共享制度安排和成本共担、利益共享机制，推动京津冀、长三角、粤港澳大湾区和成渝等主要城市群率先实现基本公共服务常住人口全覆盖，逐步实现区域内服务标准相互衔接、服务信息互联互通、服务事项异地享有。适应农村人口结构和经济社会形态的变化，强化农村公共服务供给县乡村统筹，推进县乡村公共服务一体化、均等化，鼓励社会力量兴办农村公益事业。在东、中、西部和东北地区选择一批县（市）开展县乡村公共服务一体化试点示范。加强边境地区公共服务设施建设，增加优质服务资源配置，发挥公共服务暖心留人、稳边固边的积极作用。

第七章加强规划实施保障

第一节加强党的领导

坚持党中央集中统一领导，把党的领导贯穿于公共服务发展各个阶段、各个领域、各个环节，把党的政治优势、组织优势转化为推进公共服务体系发展、促进共同富裕的强大动力。切实增强“四个意识”，坚定“四个自信”，做到“两个维护”，不断提高政治判断力、政治领悟力、政治执行力，坚持重大改革事项由党中央决定、整体工作进度由党中央掌握、政策实施情况及时向党中央报告，确保党中央关于公共服务体系建设的重大决策部署落地生效。

第二节凝聚实施合力

国家发展改革委要会同各有关部门完善基本公共服务标准体系建设部际联席会议机制，牵头制定本规划主要任务分工方案，加大对跨区域、跨领域、跨部门重大事项协调力度，研究推动重点任务、重大改革、重大项目等，着力解决堵点难点问题推动协调协商机制化常态化。各有关部门要按照职责分工，依据本规划细化提出可衡量、可考核的具体实施任务，明确工作责任和进度安排，深化政策解读，强化宣传引导，健全统计调查体系，定期分领域开展公共服务发展情况监测评估，确保本规划重点工作任务有效落实。各省、自治区、直辖市人民政府要把公共服务体系建设作为本地区“十四五”经济与社会发展重点任务，加强组织领导，明确责任分工，编制省级公共服务专项规划，细化落实举措，做好重大项目衔接统筹，确保本规划明确的重要任务和政策举措落实到位，实现公共服务能力和水平稳步提升。对新增公共服务事项、提升服务标准等要审慎研究论证，确保财力可承受、服务可持续。鼓励有条件的地区在公共服务体系统一规划、统筹建设、体制创新等方面开展试点，探索积累经验，分步推广实施。

第三节动态监测评估

国家发展改革委要会同有关部门完善规划实施监测评估机制，积极做好本规划实施年度监测、中期评估和总结评估工作，重大情况及时向党中央、国务院报告。各有关部门要定期开展分领域公共服务发展情况监测评估，跟踪督促各地区落实重点任务。
