# Supplementary material for: Mapping the evolution of fertility support policies in China: A content and instrumental analysis
Source: PLoS One. 2025 Oct 9;20(10):e0332137. doi: 10.1371/journal.pone.0332137 (PMC12510515; doi:10.1371/journal.pone.0332137)
Supplement: S1 Appendix — (ZIP) [file pone.0332137.s001.zip › S1 Appendix. 226 original policy documents/200-“十三五”全国计划生育事业发展规划.pdf]

“十三五”全国计划生育事业发展规划

本规划根据《中华人民共和国国民经济和社会发展第十三个五年规划纲要》、《中共中央 国务院关于实施全面两孩政策改革完善计划生育服务管理的决定》(中发〔2015〕40号，以下简称中央《决定》)、《国家人口发展规划(2016-2030年)》、《“健康中国”2030规划纲要》以及《“十三五”卫生与健康规划》编制，主要阐明“十三五”时期计划生育事业发展思路、目标和主要任务，是全面做好新时期计划生育工作的重要依据。

一、规划背景

“十二五”是我国计划生育事业改革发展承前启后的重要时期。党的十八大以来，以习近平同志为核心的党中央着眼人口与经济社会发展全局，强调坚持计划生育基本国策、逐步调整完善生育政策、促进人口长期均衡发展，作出一系列重大决策部署，开创了计划生育事业的新篇章。

5年来，计划生育主要目标任务顺利完成，人口自然增长率保持在5‰左右，出生人口性别比从117.94下降到113.51，2015年末全国总人口为13.75亿人。生育政策调整完善迈出历史性步伐，单独两孩政策平稳落地，全面两孩政策启动实施；卫生计生机构改革积极推进，资源整合初显成效；计划生育服务管理改革深入开展，依法行政水平明显提高；妇幼健康服务能力不断增强，出生缺陷综合防治体系逐

步形成；计划生育家庭奖励扶助政策全面落实，家庭发展能力稳步提升；流动人口计划生育全国“一盘棋”工作机制基本建立，均等化服务扎实推进；人口与发展领域的国际交流合作广泛开展，赢得国际社会积极评价。计划生育领域改革任务落实坚决、成效明显，发展态势良好，为“十三五”和今后一个时期事业发展奠定了坚实的基础。

专栏一 “十二五”时期主要指标进展情况

指标	规划目标	2015 年	2010 年
总人口数（亿人）	＜13.9	13.75	13.41
人口年均自然增长率（‰）	＜7.2	4.97	
出生人口性别比	＜115	113.51	117.94
计划生育优质服务县（市、区）比例（%）	80	87.4	72.7
免费计划生育基本技术服务覆盖率（%）	100	99.6	88
国家免费孕前优生健康检查项目目标人群覆盖率（%）	80	96.5	68.6
流动人口计划生育服务覆盖率（%）	85	89.2	76.5

“十三五”时期是我国全面建成小康社会的决胜阶段，也是实施好全面两孩政策、促进计划生育工作转型发展的关键时期。一方面，我国经济保持中高速增长，社会治理能力不断提高，民生领域投入继续加大，为计划生育事业发展提供了强大的物质基础和有利的外部环境。生育政策调整完善，卫生计生资源优化整合，为计划生育事业创新发展提供了更为广阔的平台。另一方面，人口总量压力仍然较大，结构矛盾更加突出，实现人口均衡发展需要付出长期艰苦的努力。

实施全面两孩政策的配套公共服务和相关经济社会政策还不完善，计划生育服务管理能力有待提高，计划生育特殊家庭扶助保障问题仍较突出，流动人口基本公共卫生计生服务均等化存在一定差距。做好新时期计划生育工作任务更重、要求更高、难度更大。

“十三五”时期，必须围绕全面建成小康社会这个目标，紧紧抓住贯彻落实中央《决定》这个重点，坚持计划生育基本国策，实施好全面两孩政策，创新计划生育服务管理的体制机制和方式方法，最大限度发挥人口对经济社会发展的能动作用，为全面建成小康社会创造良好的人口环境。

二、指导思想、基本原则和发展目标

（一）指导思想。高举中国特色社会主义伟大旗帜，全面贯彻党的十八大和十八届三中、四中、五中、六中全会精神，以马克思列宁主义、毛泽东思想、邓小平理论、“三个代表”重要思想、科学发展观为指导，深入贯彻习近平总书记系列重要讲话精神，紧紧围绕统筹推进“五位一体”总体布局和协调推进“四个全面”战略布局，坚持以人民为中心的发展思想，牢固树立和贯彻落实新发展理念，坚持正确的卫生与健康工作方针，全面落实中央《决定》，坚持计划生育基本国策，实施全面两孩政策，统筹推进计划生育服务管理制度、家庭发展支持体系和综合治理机制改革，引导群众负责任、有计划地生育，增进家庭和谐幸福，促进人口长期均衡发展，促进人口与经济社会、资源环境协调可持续发展。

（二）基本原则。

坚持以人为本。尊重家庭在计划生育中的主体地位，不断满足群众计划生育服务需求，积极构建鼓励按政策生育的制度环境，维护群众实行计划生育的合法权益。

坚持创新发展。大力推进人口和计划生育工作的理论创新、制度创新和文化创新，优化整合卫生计生资源，转变工作思路和方法，注重实效，全面推进计划生育治理体系和治理能力现代化建设。

坚持综合治理。加强顶层设计，注重改革措施的系统性、整体性、协同性，强化党政领导，进一步构建各相关部门齐抓共管的工作格局，同步推进生育政策、服务管理和配套措施完善。

坚持分类指导。充分考虑各地经济社会发展状况、人口发展阶段性特征以及计划生育工作实际，坚持问题导向和目标导向，把握重点，夯实基础，着力提升整体工作水平。

（三）发展目标。到 2020 年，全国总人口在 14.2 亿人左右，年均自然增长率在 6‰左右，出生人口性别比下降到 112 以下。计划生育家庭奖励扶助制度全面落实，家庭发展支持体系较为完善，新型人口文化广泛传播，鼓励按政策生育的制度体系和社会环境基本形成。妇幼健康服务体系更加健全，人人享有计划生育优质服务基本实现。计划生育目标管理责任制考核体系和运行机制进一步完善，计划生育多元共治新格局基本建立，计划生育治理能力全面提升。

专栏二　“十三五”时期主要指标

指标名称	2015 年	2020 年目标值	指标性质
总人口数（亿人）	13.7	14.2 左右	预期性

指标名称	2015 年	2020 年目标值	指标性质
人口年均自然增长率（‰）	4.97	6 左右	预期性
总和生育率	1.6　左	1.8 左右	预期性
出生人口性别比	113.51	112 以下	约束性
出生政策符合率（%）	85	90	预期性
出生人口信息准确率（%）	85	90	约束性
孕产妇系统管理率（%）	90	90	约束性
免费计划生育基本技术服务覆盖率（%）	99.6	100	预期性
免费孕前优生健康检查目标人群覆盖率（%）	80	80	预期性
农村部分计划生育家庭奖励扶助政策落实率（%）	100	100	预期性
计划生育特殊家庭联系人制度目标人群覆盖率	—	98	约束性
流动人口目标人群计划生育服务覆盖率（%）	87	90	预期性
生育登记服务目标人群覆盖率（%）	70	90	预期性

三、主要任务

（一）实施好全面两孩政策。

贯彻落实《人口与计划生育法》，推动完善相关法律法规和规章。准确把握目标人群情况，科学提出人口计划建议。加强出生人口监测，跟踪评估政策实施效果。做好奖励扶助和社会制约等政策的衔接，维护群众合法权益。

加强部门协同，合理配置妇幼保健、儿童照料、学前和中小学教育、社会保障等资源，满足新增公共服务需求。引导和鼓励社会力量举办非营利性妇女儿童医院、普惠性托儿所和幼儿园等服务机构。增强社区幼儿照料等服务功能，加快推进公共场所和用人单位母婴服务设施建设。

协调制定和完善鼓励按政策生育的经济社会政策。推动落实产假、哺乳假等制度，妥善解决延长生育假、配偶陪产

假等奖励假的待遇保障。倡导适龄生育，支持女性生育后重返工作岗位，鼓励用人单位制定有利于职工平衡工作与家庭关系的措施。鼓励生育水平长期偏低的地区采取综合措施，减轻家庭生养子女负担，引导群众按政策生育。

（二）强化计划生育基层基础工作。

优化整合卫生计生资源，巩固计划生育行政管理、技术服务、群众工作相结合的网络。健全乡级计生办或设立卫生计生办，按照常住人口比例配备专职工作人员，强化计划生育管理职能。建立健全卫生计生综合监督行政执法体系。加强村级专干队伍建设，协助落实计划生育政策、做好人口信息统计以及承担健康教育等相关公共卫生计生服务工作。妥善解决好村级专干报酬待遇、养老保障等问题。

坚持依法行政、文明执法，全面推进政务公开，规范社会抚养费征收管理。实行生育登记服务制度，优化特殊情形再生育办理流程，全面推行网上办事、一站式服务和承诺制等便民措施。将生育登记服务与孕前优生健康检查、母子健康手册发放等工作有机结合起来，及时为登记对象提供基本公共卫生计生服务。深入开展新一轮全国计划生育优质服务先进单位（以下简称新国优）创建活动。

专栏三　新国优创建活动

新国优创建活动是经批准保留的全国评比达标表彰项目。以实施好全面两孩政策为重点，以改革完善计划生育服务管理为动力，创建一批求真务实、成效明显、群众满意的新国优，以点带面，示范引领，推动实现计划生育治理体系和治理能力现代化。

创建内容包括落实党政责任、加强部门协作、做好宣传倡导、依法规范管理、提升服务水平、完善扶助保障、强化信息支撑、巩固基层网络等8个方面。

创建活动以3年为一评选周期，每一周期各省（区、市）评选出的新国优比例不超过应参评单位总数的20%。连续两个周期被评为新国优的，荣誉称号可顺延一个周期。2016年底国家卫生计生委命名第一批新国优。

加强县、乡、村级计划生育协会的组织和能力建设，在新经济组织、新社会组织、高等院校和流动人口集聚地普遍建立计划生育协会或开展协会工作。通过政策引导、项目运作、购买服务等方式，更多地把直接面向群众的计划生育宣传、指导和服务工作交由计划生育协会等社会组织承担。全面推进基层群众自治和诚信计生，引导群众自我管理、自我服务和自我教育。

（三）提升妇幼健康计划生育服务水平。

加强妇幼保健计划生育服务机构建设，加强孕产妇与新生儿危急重症救治能力建设。全面推行住院分娩补助制度，向孕产妇提供生育全过程的基本医疗保健服务。控制非医学指征剖宫产率，提高母乳喂养率。加强再生育技术服务保障，鼓励全面两孩政策目标人群较为集中的地区设置再生育或高危孕产妇门诊。

全面推行避孕方法自主知情选择，落实免费计划生育技术服务基本项目。完善免费避孕药具政府采购方式，构建药具服务管理标准体系，拓宽免费避孕药具发放范围，健全不

良反应监测网络。积极推广避孕节育知识普及、随访服务相结合的生殖健康促进模式。加强针对青少年及未婚人群、流动育龄人群的性与生殖健康教育。做好产后避孕和流产后避孕服务。大力开展计划生育和提高人口出生质量的科学研究，研发先进适用的技术和产品，提高计划生育技术服务能力。

专栏四　计划生育技术服务基本项目

1.为育龄人群免费提供避孕药具、避孕节育技术指导咨询与随访服务。<br>2.为育龄夫妇免费提供计划生育临床医疗服务，包括避孕和节育的医学检查、计划生育手术、计划生育手术并发症和计划生育药具不良反应的诊断、治疗。<br>3.为符合条件的育龄夫妇免费提供再生育技术服务。包括：生育力评价服务，取环、复通等恢复生育力手术，必要的辅助生殖技术服务。<br>4.为城乡居民免费提供避孕节育、优生优育、生殖健康、健康生活方式等科普宣传品。

坚持预防为主，综合防治出生缺陷。普及优生科学知识，推动国家免费孕前优生健康检查项目目标人群扩展到城市计划怀孕夫妇。继续开展贫困地区新生儿疾病筛查和地中海贫血防控试点项目，提高产前筛查和产前诊断人群覆盖率。逐步建立覆盖城乡居民，涵盖孕前、孕期、新生儿各阶段的出生缺陷防治免费服务项目。

促进儿童早期发展。以孕产期和0-3岁儿童为重点，逐步完善相关制度和标准，加强设施和人才队伍建设。继续推

进儿童早期发展示范基地创建工作，加强儿童营养与喂养、生长发育监测、心理行为发育评估、常见病防治等工作。依托妇幼健康服务等机构为托幼机构、社区和家庭提供早期发展指导服务。

（四）提高计划生育家庭发展能力。

加大对计划生育家庭扶助力度，对全面两孩政策实施前的独生子女家庭和农村计划生育双女家庭，继续实行现行各项奖励扶助政策，在社会保障、集体收益分配、就业创业、新农村建设等方面予以倾斜。完善计划生育家庭奖励扶助和特别扶助制度，实行扶助标准动态调整。继续实施“少生快富”工程，支持开展生育关怀等活动，推进计划生育与精准扶贫相结合。建立计划生育特殊家庭信息档案，全面落实联系人制度，协调完善经济扶助、养老保障、医疗保障、精神慰藉等扶助关怀政策，不断提高这些家庭的生活水平。

协调建立完善包括生育支持、幼儿养育、青少年发展等在内的家庭发展政策和服务体系。组织开展家庭发展追踪调查，加强家庭发展问题的实证性、前瞻性和政策性研究。全面开展创建幸福家庭活动，广泛实施新家庭计划项目。支持有条件的地方和单位开展托育服务，继续开展家庭科学育儿、青少年健康发展、养老照护等试点项目。推进医疗卫生与养老服务相结合。实施健康家庭行动。

（五）综合治理出生人口性别比偏高问题。

大力推进社会性别平等的宣传倡导。深入开展“关爱女孩行动”，以“圆梦女孩志愿行动”和“国际女童日”主题

宣传活动为载体，加强计划生育、妇女儿童权益保障相关法律法规的宣传普及。进一步完善计划生育女孩家庭扶助保障政策体系，解决计划生育女孩家庭养老保障、女孩成长成才等方面的困难和问题，努力提高女孩及女孩家庭发展能力。

建立全国集中整治“两非”专项行动、部门配合、区域协作长效机制，依法打击“两非”行为。加强执法监督，健全监管制度，建立“两非”违法机构和人员信息库，研究建立联合惩戒制度。健全考核评估办法，加大对重点省份的督查指导力度。继续推动完善相关法律法规。

（六）完善流动人口服务管理工作机制。

推动修订《流动人口计划生育工作条例》。巩固完善流动人口全国“一盘棋”工作机制，完善区域协作的组织形式和运行方式。加强大型集贸市场、产业园区、城市新建社区以及城乡结合部等流动人口集中场所卫生计生服务管理。完善流动人口卫生计生服务管理工作评估机制，发挥计划生育协会等群团组织的作用，落实流入、流出地服务管理责任和便民维权措施。加强对留守妇女、儿童和老人的帮扶。

大力推进流动人口基本公共卫生计生服务均等化，按照常住人口配置卫生计生服务资源，全面落实国家基本公共卫生服务项目。推动流动人口卫生计生服务管理政策及其与相关政策的相互衔接。深入开展流动人口健康教育和促进行动，培育流动人口健康促进场所和健康家庭。开展流动人口社会融合示范试点工作。深化流动人口信息化建设和应用，组织开展流动人口卫生计生动态监测调查，做好统计监测数据的

开放共享和分析应用。

专栏五 计划生育主要惠民政策

**农村部分计划生育家庭奖励扶助制度。**针对农村只有一个子女或两个女孩的计划生育家庭，父母年满 60 周岁以后，由中央和地方财政安排专项资金给予一定标准的奖励扶助金。<br>**“少生快富”工程。**在内蒙古、海南、四川、云南、甘肃、青海、宁夏、新疆 8 省（区）和新疆生产建设兵团允许生育三孩的地区，对自愿少生一个孩子并采取长效节育措施的夫妇给予一次性经济奖励，并引导和帮助这些家庭把奖励资金用于发展生产、勤劳致富。<br>**计划生育家庭特别扶助制度。**针对城镇和农村独生子女死亡或伤、病残（三级以上）后的夫妻，自女方年满 49 周岁以后，由中央和地方财政安排专项资金给予一定标准的扶助金。为三级以上计划生育手术并发症人员发放一定标准的特别扶助金。<br>**流动人口基本公共卫生计生服务均等化促进工程。**在全国流动人口占一定比例的区（县、市）培育和建设流动人口基本公共卫生计生服务均等化示范区（县、市），到 2020 年示范比例达到 50%左右。大力开展“新市民健康城市行”流动人口健康促进宣传活动，建设流动人口健康促进示范企业、学校和健康家庭，为流动人口青少年和未婚青年提供“青春健康”教育培训。<br>**国家免费孕前优生健康检查项目。**在全国建立免费孕前优生健康检查制度，为城乡符合生育政策并准备怀孕的夫妇提供免费孕前优生健康检查服务。<br>**“新家庭计划——家庭发展能力建设”项目。**围绕家庭保健、科学育儿、养老照护和家庭文化等内容，通过开展培训和推广试点经验

等方式，向广大计划生育家庭传播知识，培训技能，提高家庭发展能力。

（七）加强信息化建设和战略政策研究。

加快推进计划生育信息化，依托国家、省、地市、县四级人口健康信息平台，完善全员人口数据库，实现国家与省级人口和计划生育信息互联互通。建立健全计划生育业务信息系统，围绕群众需求推进“互联网+计划生育服务”，增强服务的及时性、精准性、便捷性。加强计划生育监督信息化建设，增强监督业务系统应用能力。强化信息化标准体系和安全保障体系建设，做好公民个人隐私保护。

建立健全出生人口信息管理机制，完善统计监测指标体系，巩固县乡村三级出生监测网络，推动计划生育监测点建设。加快推进生育登记、住院分娩、出生医学证明、儿童预防接种等信息交换比对，实现户籍管理、婚姻、教育、社会保障等信息共享。组织全国生育状况调查，加强人口形势分析。

专栏六 全员人口统筹管理信息系统工程

依托国家电子政务外网，建设全员人口数据库和计划生育业务信息系统，实现国家、省、地市、县、乡、村 6 级计划生育信息的互联互通。主要建设内容和目标:

全员人口个案信息数据库。包括全员人口基本信息、婚育信息、家庭信息等。在此基础上形成全员人口决策支持数据库，主要包括人口信息统计分析、家庭单元信息统计、分地区人口预测和人口迁移流动评估等业务数据。

计划生育业务信息系统。包括计划生育服务管理应用系统、人口变动信息校核与引导服务应用系统、人口决策支持应用系统。

主要目标。全员人口覆盖率达 95%，主要数据项准确率达 90%；计划生育公共服务管理事项网上办理覆盖率达 60%。

完善人口发展战略，加强计划生育政策研究。科学评估经济增长和社会发展对生育行为的影响，准确研判生育水平变动态势，科学预测中长期人口发展趋势。立足保持适度生育水平、促进人口长期均衡发展和经济社会持续健康发展，做好政策储备。充分发挥人口学会以及相关研究机构作用，加强智库建设，主动发布重要研究成果，扩大社会影响。

（八）持续深入开展宣传倡导。

依托各级党校、行政学院、党委（组）中心组、形势报告会和干部培训班等，加强党政领导干部人口理论学习，深入宣传解读中央《决定》精神，扎实推进人口国情、计划生育基本国策教育。深化人口理论研究，完善中国特色人口发展道路和人口长期均衡发展的理论体系。

深入开展婚育新风进万家活动，建设体现中华传统美德和时代精神的新型人口文化。运用传统和新兴媒体，利用世界人口日等重要纪念日，发挥各级卫生计生宣传教育机构、基层工作网络、人口学校、人口文化大院等载体的作用，广泛开展计划生育社会宣传，传播婚育新风尚。办好广播电视、报刊、网站有关人口与计划生育专栏和专题节目，继续开展中华人口文化奖评选活动，繁荣人口文化。加强舆情监测，主动回应社会关切。

（九）深化国际合作与交流。

主动参与人口与发展领域的战略与规则制定。充分利用联合国人口与发展委员会、联合国人口基金、世界卫生组织等平台，积极参与国际人口发展与计划生育领域的交流与合作。贯彻国际人口与发展大会行动纲领，参与推动联合国2030年可持续发展议程的落实。加强对外宣传工作，增进国际社会理解和支持。

积极开展国际对话交流与项目合作。加强与发达国家在低生育率应对策略、家庭发展政策方面的交流。深化与发展中国家的合作，依托人口与发展南南合作伙伴组织，加强在人口规划、政策研讨、能力建设和产品供应等方面的交流。继续推进金砖五国人口领域合作，开展"一带一路"沿线国家人口与发展领域交流与合作。

专栏七 计划生育基础建设工程

妇幼健康和计划生育服务体系建设。实施妇幼健康和计划生育服务保障工程，支持业务用房面积短缺的省、地市、县级妇幼保健计划生育服务机构建设。新增产科床位 8.9 万张，力争增加产科医生和助产士 14 万名。推进妇幼保健和计划生育技术服务资源整合，加强乡级妇幼健康计划生育服务能力，推动村卫生室和计划生育服务室共建共享。在人群密集场所和流动人口聚集社区配置避孕药具自助发放设备。

避孕药具不良反应监测体系建设。完善全国避孕药具不良反应监测网络，落实监测工作补助经费，加强监测队伍能力建设，提高监测质量和水平。

计划生育服务管理能力提升工程。通过调整转移支付项目支出结构、设立专项等方式，支持基层计划生育服务管理人员培训，为乡、村两级提供计划生育基本公共服务必要的补助，保障经常性工作所需经费。提高计划生育监督能力。支持做好贫困地区和民族地区计划生育工作。

人口与计划生育监测调查体系建设项目。组织全国生育状况调查。加强计划生育监测点建设。开展家庭发展追踪调查，完善家庭动态监测系统。实施流动人口动态监测项目，做好流动人口变动趋势及卫生计生服务管理状况分析。

人口形势分析及战略研究工程。建设高端智库，支持人口研究基地建设，做好年度人口形势分析，组织开展跨领域、前瞻性、战略性研究，加强政策储备，提出促进人口长期均衡发展的总体思路和重大政策建议。

新型人口文化建设项目。加强宣传教育阵地建设，大力推进婚育新风进万家、关爱女孩行动、创建幸福家庭活动、新家庭计划、生育关怀行动，传播和普及生殖健康知识，倡导积极科学的婚育文化。

四、保障措施

（一）加强组织领导。推动各地落实计划生育工作党政一把手亲自抓负总责，坚持计划生育工作领导小组制度，协调将计划生育工作纳入各级党委政府重大事项督查范围，作为党委政府综合工作考核评估重要内容。建立重大经济社会政策人口影响评估机制，推动人口及相关经济社会政策的有机衔接。加强政府与社会协同治理，充分发挥计划生育协会、人口学会、人口文化促进会、人口基金会等社会组织作用，

共同做好计划生育工作。

（二）健全投入机制。完善计划生育投入机制，加大扶助保障力度，支持改革完善计划生育服务管理，保障县级卫生计生部门履行管理和服务职能所必需的经费以及各项扶助保障政策、计划生育技术服务等支出，大力支持计划生育、优生优育、生殖健康领域的基础研究和科技创新。鼓励社会力量进入，建立多渠道筹资机制。

（三）完善目标管理。坚持和完善目标管理责任制，实行党委政府、责任部门、卫生计生部门三线考核，科学确定责任制的各项任务和考核指标。强化部门协作，明确部门职责，协调解决执法配合、信息共享、政策衔接等重大问题。加大重点工作定量考核权重，积极引进第三方评估和社会评价。严格兑现奖惩，落实“一票否决”制。

（四）强化督导落实。各地要根据国家规划，制订本地区计划生育事业发展规划及年度实施计划。加强规划实施监测评估，动态跟踪重大政策、工程和项目的实施进度，强化社会监督，做好中期、终期评估工作，确保规划实施效果。各省（区、市）卫生计生委要将规划贯彻落实情况及时向我委报告，我委将适时组织督促检查。
