# Supplementary material for: Mapping the evolution of fertility support policies in China: A content and instrumental analysis
Source: PLoS One. 2025 Oct 9;20(10):e0332137. doi: 10.1371/journal.pone.0332137 (PMC12510515; doi:10.1371/journal.pone.0332137)
Supplement: S1 Appendix — (ZIP) [file pone.0332137.s001.zip › S1 Appendix. 226 original policy documents/204-第十三届全国人民代表大会第五次会议关于2021年国民经济和社会发展计划执行情况与2022年国民经济和社会发展计划的决议(FBM-CLI-1-5114781).docx]

第十三届全国人民代表大会第五次会议关于2021年国民经济和社会发展计划执行情况与2022年国民经济和社会发展计划的决议

发布部门： 全国人民代表大会

发布日期：2022.03.11

实施日期：2022.03.11

时效性： 现行有效

效力级别： 工作文件

法规类别： 人大议事 营商环境优化

第十三届全国人民代表大会第五次会议关于2021年国民经济和社会发展计划执行情况与2022年国民经济和社会发展计划的决议

（2022年3月11日第十三届全国人民代表大会第五次会议通过）

第十三届全国人民代表大会第五次会议审查了国务院提出的《关于2021年国民经济和社会发展计划执行情况与2022年国民经济和社会发展计划草案的报告》及2022年国民经济和社会发展计划草案，同意全国人民代表大会财政经济委员会的审查结果报告。会议决定，批准《关于2021年国民经济和社会发展计划执行情况与2022年国民经济和社会发展计划草案的报告》，批准2022年国民经济和社会发展计划。

第十三届全国人民代表大会第五次会议关于2021年国民经济和社会发展计划

执行情况与2022年国民经济和社会发展计划草案的报告

--2022年3月5日在第十三届全国人民代表大会第五次会议上

各位代表：

受国务院委托，现将2021年国民经济和社会发展计划执行情况与2022年国民经济和社会发展计划草案提请十三届全国人大五次会议审查，并请全国政协各位委员提出意见。

一、2021年国民经济和社会发展计划执行情况

2021年是党和国家历史上具有里程碑意义的一年，也必将是载入史册的一年。我们隆重庆祝中国共产党成立一百周年，如期打赢脱贫攻坚战，正式宣布全面建成小康社会、实现第一个百年奋斗目标，开启全面建设社会主义现代化国家、向第二个百年奋斗目标进军新征程。我们召开党的十九届六中全会，总结党的百年奋斗重大成就和历史经验，通过百年党史上第三个历史决议。在以习近平同志为核心的党中央坚强领导下，各地区各部门坚持以习近平新时代中国特色社会主义思想为指导，全面贯彻党的十九大和十九届历次全会精神，弘扬伟大建党精神，按照党中央、国务院决策部署，认真执行十三届全国人大四次会议审议批准的《政府工作报告》和审查批准的《中华人民共和国国民经济和社会发展第十四个五年规划和2035年远景目标纲要》、2021年国民经济和社会发展计划，落实全国人大财政经济委员会审查意见，完整、准确、全面贯彻新发展理念，扎实做好“六稳”、“六保”工作，注重宏观政策跨周期和逆周期调节，有效应对多种风险挑战，构建新发展格局迈出新步伐，高质量发展取得新成效，统筹疫情防控和经济社会发展，全年主要目标任务较好完成，“十四五”实现良好开局，我国发展又取得新的重大成就。

（一）强化外防输入内防反弹，常态化疫情防控有力有序。坚持人民至上、生命至上，坚持全链条精准防控“动态清零”，保持全球疫情防控优势地位。

一是分区分级防控举措精准高效。严格落实人、物、环境同防措施，加强公共卫生防控救治能力建设，最大限度遏制疫情通过口岸传播，及时有效处置局部地区聚集性疫情，多措并举抓生产、增供应、强监测、畅物流，着力保障涉疫地区生活必需品供应充足、价格平稳，保障了人民生命安全和身体健康，维护了正常生产生活秩序。

二是疫苗接种和药物研发稳步推进。全力保障疫苗生产供应，截至2021年底，全国累计生产疫苗超过50亿剂，提升疫苗对变异毒株的适应性，分地区、分年龄段推进疫苗接种，累计报告接种新冠疫苗28.4亿剂次，完成全程接种人数超过12亿人，全程接种覆盖率超过85%。我国首个抗新冠病毒特效药获批上市。

三是抗疫国际合作成效突出。积极开展共享样本、共享数据、共享应对措施等。2021年，我国已向120多个国家和国际组织提供了超过20亿剂疫苗，成为世界对外提供新冠肺炎疫苗最多的国家。参与并支持新冠肺炎疫苗实施计划，提高疫苗在发展中国家的可及性和可负担性，发展中国家迄今获得的疫苗大多数来自我国。

（二）创新和完善宏观调控，经济基本盘进一步巩固。加快构建新发展格局，加强宏观政策跨周期调节，做好保供稳价工作，加大助企纾困力度，实现了较高增长和较低通胀的优化组合，经济保持较好发展态势。

一是经济增速稳居主要经济体前列。初步核算，2021年国内生产总值达到114.37万亿元，增长8.1%，是世界经济恢复发展的主要贡献国，占世界经济的比重进一步提升；人均国内生产总值超过1.2万美元，超过全球平均水平。全国城镇新增就业1269万人，全国城镇调查失业率平均为5.1%，居民消费价格指数上涨0.9%，国际收支保持基本平衡，年末外汇储备规模保持在3.2万亿美元以上。

二是保持宏观政策连续性针对性。宏观政策适应跨周期调节需要，保持对经济恢复必要支持力度，同时考虑为今年应对困难挑战预留政策空间。积极的财政政策提质增效、更可持续。保持必要的财政支出规模，地方政府专项债券发行使用管理进一步优化，建立常态化财政资金直达机制，将2.8万亿元中央财政资金纳入直达范围，及时为基层保基本民生、保工资、保运转提供财力支持。全年新增减税降费为市场主体减负超过1万亿元。对制造业中小微企业、煤电和供热企业实施阶段性缓缴税费。货币政策灵活精准、合理适度，灵活运用降准、再贷款、再贴现等多种政策工具，两次全面降准，流动性保持合理充裕，广义货币供应量（M2）余额和社会融资规模存量分别增长9%和10.3%。加大对实体经济、重点领域和薄弱环节的支持力度，企业综合融资成本稳中有降，全年新增人民币贷款19.95万亿元，比上年多增3150亿元，大型商业银行普惠小微企业贷款增幅超过40%，企业贷款平均利率为4.61%，比上年下降0.1个百分点。强化稳岗扩就业政策落实，扎实做好高校毕业生等重点群体就业工作，推进大众创业万众创新。

三是粮食能源等重要资源保供稳价工作有力有效。针对玉米、煤炭、铁矿石、铜铝锌等大宗商品价格异常波动，及时采取供需双向调节、期现货市场联动监管、预期引导等措施，打出保供稳价的政策组合拳，有力促进了价格回归合理区间。加强粮食市场调控，合理安排进口和政策性粮食库存销售，有效保障粮食市场稳定供应。加强期货市场监管和新棉收购引导，适时增加中央储备棉投放，促进棉花市场平稳有序运行。着力解决煤炭电力供应紧张问题，推动煤炭增产增供和价格回归合理水平，支持发电供热企业纾困解难，提升煤电支撑保障能力，改革完善煤电价格市场化形成机制，扩大市场交易价格上下浮动幅度，加强煤炭、液化天然气（LNG）等能源物资产运需衔接和运输保障，确保民生和公共用能需求，推进全社会节约用电。发挥跨部门协调工作机制作用，做好化肥市场保供稳价工作。制定实施原材料价格上涨应对方案以及分品种保供稳价工作方案。发挥国家储备市场调节作用，分批投放原油成品油和铜铝锌等国家储备。

四是防范化解重点领域风险扎实推进。压实地方属地责任、部门监管责任和企业主体责任，把握风险处置的力度和节奏，有序化解重点领域风险。坚持房子是用来住的、不是用来炒的定位，妥善处置高负债房地产企业风险，维护房地产市场平稳健康发展。反垄断、反不正当竞争、防止资本无序扩张取得重要成效。对严重违法违规的企业集团和一些地方的高风险金融机构及时“精准拆弹”。地方政府隐性债务风险防范化解工作扎实推进。

（三）推进高水平科技自立自强，产业链供应链安全稳定韧性持续提升。创新驱动发展战略深入实施，我国的全球创新指数排名提升至第12位，全产业链优势与国内外需求有效衔接。

一是国家战略科技力量加快壮大。首批国家实验室组建运行，北京、上海、粤港澳大湾区国际科技创新中心和怀柔、张江、大湾区、合肥综合性国家科学中心引领带动效应持续显现，成渝区域科技创新中心加快建设。生物育种等国家产业创新中心建设步伐加快，国家工程研究中心优化整合顺利完成。推动建立以企业为主体的科技创新体系，完善推广“揭榜挂帅”、“赛马”等机制。制定实施基础研究十年规划、科技体制改革三年攻坚方案、知识产权强国建设纲要（2021－2035年）。推进科技创新2030－重大项目。重大创新成果丰硕，“天问一号”探测器成功着陆火星，“羲和号”探日卫星成功发射，空间站天和核心舱和天舟货运飞船完成对接，中国人首次进入自己的空间站，自主第三代核电机组“华龙一号”投入商业运行，“深海一号”深水大气田成功投产，白鹤滩水电站全球单机容量最大功率百万千瓦水轮首批机组投产发电，国家重大科技基础设施航空遥感系统投入使用。

二是产业链供应链自主可控能力持续增强。推进保链稳链强链，推进实施基础软件、工业母机、新能源汽车和智能汽车、能源绿色低碳转型发展等领域关键核心技术攻关。延续实施研发费用加计扣除政策，将制造业企业研发费用加计扣除比例提高到100%。企业研发经费增长15.5%。推动原料药产业高质量发展，促进生物医药、高端仪器等重点领域产业链供应链安全稳定。推进铁矿石、铜等产供储加销体系建设，支持国内铁矿资源项目开发建设，战略性矿产资源安全保障能力进一步提升。

三是产业结构持续优化。振作工业经济运行，推动工业高质量发展，实施制造业核心竞争力提升五年行动计划和产业基础再造工程，制造业生产稳定、效益改善带动投资持续恢复。2021年制造业增加值占国内生产总值的比重达到27.4%，比上年提高1.1个百分点；带动制造业固定资产投资增速达到13.5%。先进制造业和现代服务业融合发展，加快推动制造服务业高质量发展，第二批先进制造业和现代服务业融合发展试点顺利开展。中国品牌日活动成功举办。

四是新产业新业态茁壮成长。战略性新兴产业集群建设稳步推进，新兴产业发展水平不断提升，高技术制造业增加值、高技术产业投资分别增长18.2%、17.1%。国产商用飞机持续创新发展，北斗产业化应用不断深化，国家民用空间基础设施建设加快推进。数字经济健康发展，实施“东数西算”工程，布局建设全国一体化大数据中心，完善数字经济监管制度，统筹线上线下一体化监管，推动平台经济规范健康持续发展。双创活动周系列活动成功举办，创业带动就业示范行动深入实施，全社会创新创业热情进一步激发释放。

（四）坚持扩大内需这个战略基点，国内大循环加快畅通。着力培育完整内需体系，充分激发国内需求潜力，消费和投资稳定恢复，供需循环进一步畅通。

一是消费在持续恢复中稳步升级。完善消费场所常态化疫情防控举措，挖掘消费潜力，全面促进消费，居民消费持续恢复。基本消费品产销稳定增长，升级类商品消费增势良好，新能源汽车等销量快速增长，餐饮、旅游、文化、体育、娱乐等服务消费逐步恢复。新型消费加快培育，线上线下消费融合持续推进。农村电商、物流覆盖面扩大，全国建制村“快递进村”比例超过80%，农村消费潜力进一步释放。社会消费品零售总额达到44.08万亿元，增长12.5%；网上零售额达到13.09万亿元，增长14.1%，其中实物商品网上零售额达到10.8万亿元，增长12%，占社会消费品零售总额的24.5%。

二是投资补短板力度持续加大。中央预算内投资注重聚焦大事、急事、难事，“十四五”规划《纲要》102项重大工程项目有序实施，“两新一重”和补短板项目建设积极推进。公共卫生、防灾减灾、重大科技和能源领域基础设施建设步伐加快，150项重大水利工程累计开工62项，新开工改造城镇老旧小区达5.6万个。规范推广政府和社会资本合作（PPP）模式，深入推进投融资体制机制改革。开展基础设施领域不动产投资信托基金（REITs）试点，有效盘活基础设施存量资产。固定资产投资（不含农户）增长4.9%，其中民间投资增长7.0%，社会领域投资增长10.7%。

三是基础设施发展质量效益不断提升。川藏铁路、西部陆海新通道、沿江高铁等重大项目建设进展顺利，乌东德水电站12台机组全部建成投产，雅鲁藏布江下游水电开发前期工作有序推进。全年铁路投资完成7489亿元，铁路营运总里程突破15万公里，其中高铁运营里程突破4万公里，“四纵四横”高铁网全面建成，“八纵八横”高铁网正在加密形成。修订国家公路网规划和全国港口与航道布局规划，公路水路建设稳步推进，建成自动化码头10座。革命老区、沿边抵边基础设施建设扎实推进。加快推进新型基础设施建设，建成全球最大的5G独立组网网络。交通低碳、智能发展加快推进。

四是现代流通体系加快建设。编制现代流通、现代物流、冷链物流、粮食物流等系列重大专项规划，物流基础设施建设稳步推进，全年新增国家物流枢纽25个，国家骨干冷链物流基地建设步伐加快，“通道+枢纽+网络”现代物流运行体系初步形成。统筹开展县域商业体系建设和城市商业提升行动。积极推动物流降本增效，社会物流成本水平稳中有降。

（五）全面实施乡村振兴战略，农业农村现代化扎实推进。乡村振兴战略规划深入实施，脱贫地区发展能力不断增强，农村一二三产业融合发展取得新进展。

一是巩固拓展脱贫攻坚成果同乡村振兴有效衔接。确定160个国家乡村振兴重点帮扶县。加大衔接推进乡村振兴补助资金投入力度，支持欠发达地区培育特色优势产业。加强对脱贫不稳定户、边缘易致贫户和突发严重困难户的监测帮扶，将240万人纳入低保或特困供养范围。加大以工代赈和易地扶贫搬迁后续扶持工作力度，开展搬迁安置区就业协作帮扶，基本实现有劳动力的搬迁家庭至少一人就业，脱贫劳动务工规模达3145万人。推动消费帮扶提档升级，市场化帮扶比例超过2/3。深化东西部协作和中央单位定点帮扶，开展“万企兴万村”行动，推动脱贫地区种养业高质量发展。

二是现代农业基础持续夯实。及时有力做好洪涝、台风、干旱等自然灾害应对，启动512处大中型灌区续建配套与现代化改造。对种粮农民一次性发放200亿元补贴，应对农资价格较快上涨等影响。提高稻谷、小麦最低收购价，确保口粮绝对安全。粮食总产量达到13657亿斤，实现高位增产，玉米年产量创历史最高水平，肉蛋奶产量达到1.6亿吨，蔬菜、水果等“菜篮子”产品量足价稳。农业生产力布局优化与结构调整加快推进，新建高标准农田1.05亿亩。启动实施国家黑土地保护工程，东北黑土地保护性耕作7200万亩。开展全国农业种质资源普查，核心种源技术攻关加快推进，种业振兴稳步开局。推进棉花、食糖产供储加销体系建设。

三是乡村建设有序推进。实施乡村建设行动和农村人居环境整治提升五年行动。实施农网巩固提升工程，进一步提升农村用电质量和水平。开展中小河流治理、大中型病险水库除险加固等水利薄弱环节工程建设，防汛基础设施短板进一步补齐。加快长江黄河流域农业面源污染治理项目建设，实施畜禽粪污资源化利用整县推进项目。因地制宜推进农村厕所革命，全国农村卫生厕所普及率超过70%。生活污水和垃圾治理力度加大，生活垃圾进行收运处理的自然村比例稳定保持在90%以上。现有行政村全面实现村村通宽带，农村自来水普及率达84%。深入推进农村产业融合发展，新创建一批农业现代化示范区、国家现代农业产业园，第三批国家农村产业融合发展示范园创建工作积极开展。

（六）有效推动区域协调发展和新型城镇化建设，区域经济布局调整优化。发挥比较优势、突出区域特色，着力促进城乡融合发展，深入实施区域重大战略和区域协调发展战略，地区经济运行稳中加固、稳中向好。

一是区域重大战略深入实施。北京非首都功能疏解取得新突破，国务院印发《关于支持北京城市副中心高质量发展的意见》，雄安新区进入大规模建设阶段，落实支持天津滨海新区高质量发展的意见，张家口首都水源涵养功能区和生态环境支撑区加快建设。长江经济带生态优先、绿色发展深入推进，生态环境突出问题整改和污染治理“4+1”工程稳步推进实施，生态环境系统保护修复成效明显，长江十年禁渔实现良好开局，水生生物资源逐步恢复，沿江综合交通运输体系加快构建，长江保护法正式实施。粤港澳大湾区建设稳步推进，中共中央、国务院印发《横琴粤澳深度合作区建设总体方案》、《全面深化前海深港现代服务业合作区改革开放方案》，基础设施和公共服务融合有序开展。长三角一体化发展深入推进，基础设施互联互通水平持续提升，一体化发展体制机制不断优化，中共中央、国务院印发《关于支持浦东新区高水平改革开放　打造社会主义现代化建设引领区的意见》，构建长三角世界级港口群形成一体化治理体系、共建虹桥国际开放枢纽等重大任务有力推进，生态绿色一体化发展示范区建设取得显著成效。“十四五”黄河流域生态保护和高质量发展实施方案印发，农业、工业、城镇生活和尾矿库污染“3+1”综合治理方案扎实推进，污染防治、生态保护修复、深度节水控水、水土保持等领域重大工程项目稳步实施，在黄河省区完成1.2万平方公里水土流失治理任务。

二是区域协调发展战略持续推进。执行新修订的《西部地区鼓励类产业目录》，优惠政策覆盖面进一步扩大，促进400毫米降水线西侧区域保护发展。东北地区国资国企改革持续推进，结构调整与布局优化力度加大，辽宁沿海经济带高质量发展规划印发实施。中共中央、国务院印发《关于新时代推动中部地区高质量发展的意见》。支持湖北经济社会发展一揽子政策任务全部完成，河南等地救灾与灾后恢复重建工作扎实推进。鼓励东部地区进一步提升创新能力，福建全方位推动高质量发展，济南新旧动能转换起步区加快建设，江苏沿海地区发展规划印发实施。有力推进重点领域重点平台建设，国家级新区和承接产业转移示范区、临空经济示范区等重点项目加快建设。以脱贫地区为重点的欠发达地区、革命老区、边境地区、生态退化地区、资源型地区和老工业城市等特殊类型地区振兴发展支持力度持续加大。加快发展海洋经济，推进建设海洋强国。

三是以人为核心的新型城镇化深入推进。坚持走中国特色新型城镇化道路，进一步完善新型城镇化战略，常住人口城镇化率达64.72%。城市落户限制有序放开放宽，城镇基本公共服务覆盖未落户常住人口工作持续推进，农业转移人口技能素质持续提升。城市群和都市圈承载能力不断提高，成渝地区双城经济圈建设“1+N”规划体系加快形成。县城补短板强弱项工作加快推进，120个县城建设示范地区带动作用有效发挥，边境地区城镇功能持续强化，特色小镇规范健康发展有序推进。国家城乡融合发展试验区加快改革探索。

（七）持续深化市场化改革，市场主体信心和活力不断增强。围绕重点领域和关键环节把改革推向深入，聚焦市场主体关切，更加精准地出台改革方案，各项改革举措加快落地见效。

一是要素市场化配置体制机制继续完善。完善市场体系基础制度，出台实施要素市场化配置综合改革试点总体方案，市场准入负面清单制度改革持续深化。建设高标准市场体系行动方案落地实施，全国统一大市场建设稳步进行。招标投标领域优化营商环境长效机制逐步健全，企业在招标投标、政府采购等方面存在的差别化待遇进一步消除，公共资源交易平台整合共享深入推进，交易平台服务行为进一步规范，交易平台区域一体化合作扎实开展。

二是各类市场主体活力进一步激发。深入实施国企改革三年行动方案，加快国有经济布局优化和结构调整，国有经济进一步聚焦战略安全、产业引领、国计民生、公共服务等功能。国有企业混合所有制改革积极稳妥推进，近100户试点企业完成主体改革任务。持续为民营企业改革发展营造良好环境，20多部涉产权法律法规立改废，5847件涉产权规章规范性文件被清理。民营企业进入油气进出口、铁路、核电等领域，首条民间资本控股的杭绍台高铁建成通车。总结8个地方72条支持民营企业改革发展的典型做法并向全国推广，充分发挥示范引领作用。充分发挥各级融资信用服务平台作用，加强信用信息共享应用促进中小微企业融资。

三是全国范围营商环境持续优化。“放管服”改革取得新进展。深入实施《优化营商环境条例》，营商环境评价扎实开展，开展营商环境创新试点。出台实施市场主体登记管理条例，统一市场主体登记制度。规范涉企收费。投资项目审批制度改革持续深化，投资管理法规制度体系基本形成，进一步压减涉企审批手续和办理时限，更多事项实现“一网通办”。强化反垄断和反不正当竞争执法，加大对平台企业监管力度。全年新设市场主体2887.2万户，市场主体总数超过1.5亿户，其中，个体工商户1.03亿户，市场主体活跃度保持在70%左右。

四是财税、金融、价格等重点领域改革稳步推进。出台关于进一步深化预算管理制度改革的意见，印花税法正式公布。深交所主板和中小板合并顺利实施，设立北京证券交易所和广州期货交易所，第三支柱养老保险规范发展。创新抽水蓄能价格机制，出台新能源平价上网政策，优化峰谷分时电价机制，创新天然气管道运价机制，完善城镇供水价格政策，非居民厨余垃圾计量收费和超定额累进加价机制逐步建立。电力体制改革多点并进，启动第二批电力现货交易试点，配售电竞争性业务有序放开，绿电交易试点正式启动。油气体制改革深入推进，建立起以国家石油天然气管网集团有限公司为纽带的油气生产运行新机制，调峰和应急保供能力进一步增强。

（八）多措并举稳定外贸外资，对外开放范围、领域和层次持续拓展。积极参与国际经济合作，对外开放水平不断提高。全年货物进出口总额达39.1万亿元，增长21.4%，实际使用外商直接投资金额1735亿美元，增长20.2%，对外非金融类直接投资额1136亿美元，增长3.2%。

一是稳外贸稳外资举措成效明显。加快发展外贸新业态新模式，外贸进出口质量进一步提升。进博会、广交会、服贸会、消博会、投洽会等重大展会成功举办。扩大跨境电商零售进口试点范围，布局建设一批海港陆港海外仓。全面深化服务贸易创新发展试点稳步推进。深入落实外商投资法，制定发布2021年版全国和自贸试验区外商投资准入负面清单，特别管理措施分别缩减至31条、27条。中国（上海）自贸试验区临港新片区建设发展再上新台阶。稳妥有序推进资本项目开放，跨境人民币结算政策进一步完善。健全全口径外债管理，持续优化外债结构，开展中长期外债监督检查和风险排查试点，防范企业外债风险。着力引导企业外债资金用于落实国家重大战略、促进重点领域发展。

二是共建“一带一路”高质量发展取得新进展。截至2021年底，我国已与145个国家、32个国际组织签署200多份共建“一带一路”合作文件。统筹推进境外项目疫情防控和生产建设，一批重大标志性项目取得积极进展，中老铁路建成通车，中巴、中蒙俄、中国－中南半岛等经济走廊以及雅万高铁、匈塞铁路等重点项目建设稳步推进，比雷埃夫斯港第二阶段股权顺利交割。扎实推进第三方市场合作。“一带一路”绿色能源合作加快推进。中欧班列安全稳定运行，全年开行1.5万列，运送货物146万标箱，分别增长22%、29%，重箱率98.1%，累计通达欧洲23个国家的180个城市。“一带一路”风险防控和安全保障体系建设不断完善，地方参与和融入共建“一带一路”水平不断提升。

三是对外开放平台建设稳步推进。海南自由贸易港法以及海南自由贸易港外商投资准入负面清单、跨境服务贸易负面清单颁布实施，对部分进口商品实施“零关税”政策，放宽市场准入特别措施、鼓励类产业目录和贸易自由化便利化措施出台实施。推进西部陆海新通道主通道建设，打造北部湾国际门户港，通道沿线与东盟的开放合作持续深化实化。北京国家服务业扩大开放综合示范区建设扎实推进，新增4个服务业扩大开放综合试点。

四是参与全球经济治理迈出新步伐。坚定维护以世界贸易组织（WTO）为核心的多边贸易体制，在二十国集团（G20）、亚太经合组织（APEC）、金砖国家等多边场合提出中国方案。推动《区域全面经济伙伴关系协定》（RCEP）正式生效，申请加入《全面与进步跨太平洋伙伴关系协定》（CPTPP）、《数字经济伙伴关系协定》（DEPA）。中柬（埔寨）自贸协定正式生效实施。中韩自贸协定第二阶段谈判、中新（加坡）自贸协定升级后续谈判等有序推进。

（九）扎实推进生态文明建设，全面绿色转型步伐稳健。深入打好污染防治攻坚战，污染防治成果巩固拓展，做好碳达峰碳中和工作，绿色生产生活方式加快形成。

一是生态安全屏障逐步筑牢。山水林田湖草沙一体化保护和系统治理扎实推进，国有林场和重点国有林区改革取得积极成效，青藏高原生态屏障区生态保护和修复等重大工程建设规划加快编制，天然林保护与营造林、退化草原修复、水土流失治理、荒漠化治理、湿地保护等重点任务有序落实，全国新增水土流失治理面积6.2万平方公里，新增国土绿化面积超1亿亩。以国家公园为主体的自然保护地体系加快建立。福建、江西、贵州、海南国家生态文明试验区建设取得新进展。生态保护补偿体制机制更加健全。进一步加强生物多样性保护，成功举办联合国《生物多样性公约》第十五次缔约方大会第一阶段会议。

二是碳达峰碳中和工作有序推进。出台实施《关于完整准确全面贯彻新发展理念做好碳达峰碳中和工作的意见》和《2030年前碳达峰行动方案》，加快构建碳达峰碳中和“1+N”政策体系。加快建立健全绿色低碳循环发展经济体系，大力推动产业绿色转型和能源结构调整，以甘肃、青海、内蒙古、宁夏、新疆等地的沙漠、戈壁、荒漠地区为重点，加快推进大型风电、光伏基地项目规划建设。加大对风电、光伏等可再生能源和煤炭清洁高效利用重点领域的金融支持力度。可再生能源发电装机规模突破10亿千瓦。积极建设性参与联合国气候变化格拉斯哥大会各项议题谈判磋商。全国碳排放权交易市场启动上线交易，第一个履约周期纳入发电行业重点排放单位2162家，碳减排支持工具落地生效。能源消费强度和总量双控制度进一步完善，明确重点领域能效标杆水平和基准水平，严格能效约束，坚决遏制“两高”低水平项目盲目发展。

三是污染防治攻坚战持续深化。深入打好污染防治攻坚战，推动构建现代环境治理体系。细颗粒物（PM2.5）和臭氧协同控制不断强化，城市黑臭水体和入河入海排污口及工业园排查整治力度加大，土壤污染管控和修复持续深化。推进污水资源化利用，大力推动城镇环境基础设施建设，深入推行清洁生产，塑料污染全链条治理有力推进，大宗固废综合利用取得积极进展，全面实现洋垃圾零进口。全国地级及以上城市空气质量优良天数比率达到87.5%，PM2.5平均浓度下降9.1%，地表水水质优良（Ⅰ－Ⅲ类）断面比例提高到84.9%，劣Ⅴ类水质断面比例降至1.2%。

（十）着力保障改善民生，人民生活水平稳步提高。不断做好普惠性、基础性、兜底性民生建设，提高公共服务水平，社会保障网进一步织密织牢。

一是促进共同富裕各项工作扎实推进。谋划促进共同富裕顶层设计，支持浙江高质量发展建设共同富裕示范区。收入分配制度改革持续深化，完善初次分配政策，加大再分配调节力度。城乡居民收入稳步增长，全国居民人均可支配收入实际增长8.1%。

二是就业优先政策继续强化。延续实施部分阶段性减负稳岗扩就业政策，创业带动就业示范行动、就业服务质量提升工程深入开展，推进劳务品牌建设。促进高校毕业生、退役军人、农民工等重点群体就业创业，加强新就业形态劳动者权益保障，促进灵活就业健康发展。2021年创业带动就业示范行动带动就业约200万人。大规模开展职业技能培训，支持建设一批公共实训基地。加强返乡入乡创业园建设，强化农民工等人员返乡入乡创业平台支撑。

三是教育强国建设步伐加快。教育强国推进工程启动实施，支撑基础教育补短板、职业教育树精品、高等教育创一流。九年义务教育巩固率、高中阶段教育毛入学率分别达到95.4%、91.4%，普通高等教育本专科招生和研究生招生超过1100万人。扎实推进义务教育“双减”工作，加强对学科类校外培训收费监管。提高农村义务教育学生营养膳食补助标准，深入推进义务教育薄弱环节建设与能力提升工作。持续增加普惠性学前教育资源供给，改善县域普通高中学校基本办学条件。集中力量支持脱贫县以义务教育为重点，统筹学前教育资源建设，扩大学位供给。超额完成高职扩招三年行动目标。加强经济社会发展重点领域急需学科专业建设和人才培养，加大对集成电路、人工智能等学科专业的支持。在集成电路、储能技术等关键领域布局建设一批产教融合创新平台。新认定63家国家产教融合型企业和21个国家产教融合试点城市。

四是健康中国建设稳步推进。深入开展爱国卫生运动，持续推进健康中国行动，实施国民营养计划。食品药品等产品质量安全监管不断加强。公立医院综合改革持续深化。推动优质医疗资源扩容和区域均衡布局，国家医学中心建设工作稳步推进，国家区域医疗中心建设项目试点范围有序扩大，分级诊疗和医联体建设加快推进。公共卫生防控救治能力建设加快推进，疾控中心建设步伐加快。促进中医药传承创新。推动药品集中采购常态化制度化，全国药品和医用耗材集中带量采购范围继续扩大，医保目录内部分药品价格大幅下降，重大疾病和特殊人群用药保障水平明显提高。优化生育政策，实施三孩生育政策及配套支持措施。

五是多层次社会保障体系不断健全。基本养老保险参保人数达10.29亿人，参保率超过91%。推进基本养老保险全国统筹，退休人员基本养老金稳步提高。职工基本医疗保险、城乡居民基本医疗保险和大病保险制度更趋完善，医疗保障待遇清单制度建立，基本医疗保险覆盖13.6亿人，参保率稳定在95%以上，城乡居民基本医疗保险参保居民政策范围内住院费用报销比例保持在70%，普通门诊费用跨省直接结算已覆盖所有统筹地区，住院费用跨省直接结算率达到60%，长期护理保险制度试点扩大。失业保险、工伤保险参保人数进一步增加，阶段性失业补助金政策延续实施，通过工伤保险为202万人次工伤职工及供养亲属提供待遇保障。加强困难群众基本生活保障，建立低收入人口动态监测机制，推进分层分类的社会救助体系建设，及时给予基本生活救助、专项救助或急难社会救助。加快推进低保制度城乡统筹，做到应保尽保。进一步健全社会救助和保障标准与物价上涨挂钩联动机制，扩大价格临时补贴发放范围。加快完善住房保障体系，支持人口净流入的大城市扩大保障性租赁住房供给。加强防灾减灾救灾工作，妥善安置受灾群众，支持河南郑州等地特大暴雨洪涝灾害灾后恢复重建，防范遏制重特大事故。

六是公共服务补短板强弱项提质量深入推进。出台实施《国家基本公共服务标准（2021年版）》，逐步完善行业标准规范，组织基层标准化试点，完善均等化推进机制。长城、大运河、长征、黄河等国家文化公园标志性项目建设有序推进。实施文化保护传承利用工程，加强重要文化遗产和重要自然遗产保护利用。推动公共文化服务高质量发展，实施全国智慧图书馆体系、公共文化云建设项目，促进城乡新型公共文化空间建设。研究制定国民旅游休闲发展纲要，推动落实带薪休假制度。支持红色旅游、乡村旅游高质量发展。制定关于构建更高水平的全民健身服务体系的意见，推进体育公园建设，人均体育场地面积达2.41平方米。深化促进家政服务业提质扩容“领跑者”行动，开展家政企业信用建设行动。加快推进老龄事业和老龄产业协同发展，居家社区机构相协调、医养康养相结合的养老服务体系初步建立，全社会养老床位数达813.5万张。老年人合法权益保障工作不断加强，城乡无障碍环境明显改善。发展普惠托育服务体系，推动建设一批方便可及、价格可承受、质量有保障的托育机构。健全残疾人两项补贴标准动态调整机制。做好妇女儿童权益保障工作，推进儿童友好城市建设，未成年人救助保护网络逐步健全，残疾人、孤儿等社会福利制度不断完善。

总的来看，2021年经济增长、就业、居民消费价格、国际收支等预期目标较好完成，科技创新、资源节约、环境保护、社会保障等领域指标持续改善，粮食能源生产稳步增长，全年经济社会发展主要目标任务完成情况良好。事非经过不知难。面对复杂严峻的国际国内形势，我们保持战略定力，坚定不移办好自己的事，奋力完成改革发展艰巨任务，顺利实现了全面建成小康社会的第一个百年奋斗目标，在全面建设社会主义现代化国家、向第二个百年奋斗目标进军的新征程上迈出了坚实一步。这些成绩的取得，是以习近平同志为核心的党中央坚强领导的结果，是习近平新时代中国特色社会主义思想科学指引的结果，是全党全国各族人民勠力同心、艰苦奋斗的结果。

同时也要看到，当前我国发展面临的问题和挑战明显增多。从国际形势看，全球疫情仍在持续，世界经济复苏动力不足，大宗商品价格高位波动，外部环境更趋复杂严峻和不确定。从国内形势看，经济发展面临多年未见的需求收缩、供给冲击、预期转弱三重压力，局部疫情时有发生，新的经济下行压力凸显，一些领域风险可能加快暴露，保持经济平稳运行难度明显加大。一是总需求收缩。市场环境总体趋紧，消费和投资恢复迟缓，有效需求依然不足。居民人均可支配收入两年平均增速低于疫情前水平，加上疫情散发的扰动，城市居民的消费能力、消费意愿受到抑制，线下接触性消费恢复仍受疫情影响。投资内生动力不强，制造业投资后续增长基础不牢，房地产土地购置面积下降、开发投资增速下滑，一些地方土地出让收入减少、资金用地等要素保障不足，影响扩大有效投资。同时，随着部分国家经济秩序恢复后供需缺口收窄，国际市场竞争更加激烈，稳出口难度增加。二是结构性短缺。企业生产仍面临要素短缺等问题，煤炭和电力供应保障仍然存在一些制约因素，石油天然气等大宗商品供应履约易受全球供求形势变化、地缘政治风险等影响，部分行业“卡脖子”问题突出，缺芯、缺柜、缺工等问题仍不同程度存在。高技术产业发展面临的国际环境更加严峻，科技创新能力还有待加强。资源环境压力也会对部分产品供给形成一定制约。三是成本和价格上扬。需求趋紧，供给受限，能源、矿产等大宗商品价格高位运行，企业主要生产经营指标不容乐观，一些中下游制造业企业特别是小微企业消化成本压力难度增大，用工贵、应收账款回收难等问题凸显。四是经济金融领域风险有所抬头。一些高负债、盲目扩张的企业特别是房地产企业风险暴露。部分中小银行资产质量下降，部分地区金融风险仍在积聚。一些地方经济恢复发展困难较多，基层财政收支矛盾仍较突出，部分地方政府偿债压力大，保基本民生、保工资、保运转难度仍然较大。生育、养育、教育、医疗、养老、环保等民生领域还有不少短板弱项。保障粮食安全和能源供应还面临不少挑战，资本无序扩张可能形成较大风险隐患。安全生产压力仍然较大，极端天气事件可能趋多趋强，防灾减灾任务艰巨。五是市场预期不稳。不少市场主体特别是小微企业和个体工商户生产经营困难，对市场前景存在担忧，稳就业任务更加艰巨。此外，多目标多政策的统筹协调能力有待提升，政府工作存在不足，形式主义、官僚主义仍然突出，脱离实际、违背群众意愿现象屡有发生，有的在政策执行中采取“一刀切”、运动式做法，有的地方对碳达峰碳中和目标的理解和认识还有偏差，有的地方对政策执行简单机械，有的存在局部合理政策叠加造成负面影响。

我们既要正视困难，也要坚定信心。尽管我国经济运行面临的困难和挑战明显增多，但经济持续恢复发展的良好态势没有改变，支撑高质量发展的生产要素条件没有改变，经济韧性强、长期向好的基本面没有改变，我国发展仍处于重要战略机遇期。在以习近平同志为核心的党中央坚强领导下，党的百年奋斗重大成就和历史经验汇聚强大精神力量，集中力量办大事的制度优势持续彰显，综合国力稳步增强和超大规模国内市场潜力形成坚强支撑，改革开放深入推进激发市场活力，区域城乡协调发展培育强大动力，特别是亿万人民有追求美好生活的强烈愿望、创业创新的巨大潜能、共克时艰的坚定意志，还积累了应对重大风险挑战的丰富经验，我们有基础、有条件、有信心、有能力保持经济平稳健康可持续发展。

二、2022年经济社会发展总体要求、主要目标和政策取向

2022年将召开中国共产党第二十次全国代表大会，是党和国家事业发展进程中十分重要的一年，做好经济工作，意义十分重大。

（一）总体要求。

做好2022年经济工作，要在以习近平同志为核心的党中央坚强领导下，以习近平新时代中国特色社会主义思想为指导，全面贯彻落实党的十九大和十九届历次全会精神，弘扬伟大建党精神，坚持稳中求进工作总基调，完整、准确、全面贯彻新发展理念，加快构建新发展格局，全面深化改革开放，坚持创新驱动发展，推动高质量发展，坚持以供给侧结构性改革为主线，统筹疫情防控和经济社会发展，统筹发展和安全，继续做好“六稳”、“六保”工作，持续改善民生，着力稳定宏观经济大盘，保持经济运行在合理区间，保持社会大局稳定，迎接党的二十大胜利召开。

在具体工作中，要正确把握社会主要矛盾和中心任务，重视战略策略问题，把战略的坚定性和策略的灵活性结合起来，深化对重大理论和实践问题的研究，正确认识和把握实现共同富裕的主要目标和实践途径，正确认识和把握资本的特性和行为规律，正确认识和把握初级产品供给保障，正确认识和把握防范化解重大风险，正确认识和把握碳达峰碳中和，在整体推进中实现重点突破，以重点突破带动经济社会发展水平整体跃升。要把稳增长放在更加突出的位置。经济工作是党治国理政的中心工作，发展是解决我国一切问题的基础和关键。必须坚持发展是第一要务，牢牢把握稳是主基调、稳是大局，努力解决经济运行中的两难、多难问题，促进平稳健康可持续发展。要统筹稳增长、调结构、推改革。加快转变发展方式，着力在提高供给体系质量、畅通经济循环上下更大功夫，深入实施创新驱动发展战略，推进高水平科技自立自强，以更大决心推动重点领域关键环节改革，着力打通各种卡点、堵点，强化要素保障支撑，增加高质量供给，带动消费升级、促进有效投资，形成强大国内市场。要坚持实事求是。立足社会主义初级阶段基本国情，尊重发展规律、客观实际和群众需求，因地制宜创造性地开展工作，把各方面干事创业积极性充分调动起来。要推动有效市场和有为政府更好结合。更加注重运用市场化手段，加快破除制约高质量发展的深层次体制机制障碍，建设更高水平开放型经济新体制，加快打造国际一流营商环境，更大激发市场活力和社会创造力。要更加注重统筹发展和安全。深刻把握世界百年未有之大变局演变态势，密切跟踪国际国内形势变化，加强重点领域的风险防控，提前做好应对各种复杂局面的工作预案，牢牢守住不发生系统性风险的底线。要坚持以人民为中心的发展思想。在发展中持续改善人民生活，在推动高质量发展中强化就业优先导向，坚持尽力而为、量力而行，完善公共服务政策制度体系，依靠共同奋斗，扎实推动共同富裕，保障好基本民生，不断增强人民群众的获得感、幸福感、安全感。

（二）主要预期目标。

按照上述总体要求，坚持立足当前、着眼长远，在综合平衡基础上兼顾需要与可能，提出2022年经济社会发展主要预期目标：

--国内生产总值增长5.5%左右。主要考虑：一是这一目标充分考虑了当前经济运行实际，体现了稳就业保民生防风险的需要，有利于引导发展预期、提振市场信心，调动各方面积极性。二是这一目标同近两年平均经济增速以及“十四五”规划目标要求相衔接。三是这一目标符合各方面对我国经济发展的预期，这是高基数上的中高速增长，体现了主动作为，需要付出艰苦努力才能实现。

--城镇新增就业1100万人以上，城镇调查失业率全年控制在5.5%以内。关于城镇新增就业：综合考虑高校毕业生等重点群体、产业升级带来的职工转岗等就业需求，实现1100万人以上的城镇新增就业是保就业的基本要求；同时，随着经济持续恢复特别是促进就业各项政策落地见效，实现1100万人以上的城镇新增就业有较好支撑。关于城镇调查失业率：主要考虑就业攸关民生和社会稳定，必须进一步突出就业优先的政策导向，充分体现稳就业的决心，压实各方特别是各地保就业责任，这一目标也是可以实现的。

--居民消费价格涨幅3%左右。主要考虑：综合输入性通胀压力可能延续、生产端成本上升可能逐步向消费端传导等因素，叠加翘尾影响，预计2022年居民消费价格上涨压力可能比2021年大，将居民消费价格涨幅设为3%左右，体现了做好生活必需品保供稳价、保持价格总水平基本稳定的工作要求，同时也适当留有余地，有利于稳定市场预期。

--居民收入增长与经济增长基本同步。主要考虑：这是坚持以人民为中心发展思想的必然要求，是扩大消费、稳住经济的重要基础。随着促进就业、提高就业质量、扩大中等收入群体、增加低收入群体收入、加大再分配调节力度等政策措施的推进，2022年居民收入增长有望继续与经济增长基本同步。

--进出口促稳提质，国际收支基本平衡。主要考虑：这是应对复杂严峻外部环境变化的需要，也是稳住外资外贸基本盘、保持经济平稳运行的重要举措。同时，我国全产业链优势继续显现、各项稳外贸稳外资政策落地见效、新业态新模式蓬勃发展、贸易高质量发展步伐加快，可以为进出口促稳提质、国际收支基本平衡提供有力支撑。

--粮食产量保持在1.3万亿斤以上。主要考虑：民以食为天，粮稳则天下安，确保粮食安全是必须守住的安全底线。虽然我国粮食连年丰收，但供需仍处于紧平衡状态，统筹考虑国内粮食消费需求、综合生产能力、全球粮食市场变化等因素，为保障市场供应和价格稳定，必须保持粮食产量在1.3万亿斤以上。

--生态环境质量持续改善，主要污染物排放量继续下降；能耗强度目标在“十四五”规划期内统筹考核，并留有适当弹性，新增可再生能源和原料用能不纳入能源消费总量控制。主要考虑：按照“十四五”序时进度设置生态环境年度目标，推进精准、科学、依法治污，深入打好污染防治攻坚战，加快实施各项重点减排工程。同时，综合当前经济发展实际、保障能源供给安全与实现节能降碳目标等因素，将能耗强度目标在“十四五”规划期内统筹考核，留有适当弹性，既为保障经济平稳运行留出合理用能空间，也能有效推动地方保持节能工作力度，兼顾发展和减排、当前和长远，在保障能源安全的前提下稳步推进绿色低碳发展，为实现“十四五”时期能耗强度降低目标提供支撑。

（三）主要宏观政策取向。

为实现上述目标，要坚持稳字当头、稳中求进。宏观政策要稳健有效，微观政策要持续激发市场主体活力，结构政策要着力畅通国民经济循环，科技政策要扎实落地，改革开放政策要激活发展动力，区域政策要增强发展的平衡性协调性，社会政策重在兜住兜牢民生底线。各方面要围绕贯彻这些重大政策和要求，细化实化具体举措。加强多元目标统筹，做好各项政策协调衔接，进一步发挥中央和地方两个积极性，政策发力适当靠前，及时动用储备政策工具，把握好政策时度效，加强经济监测预警和政策预研储备，对苗头性趋势性问题早发现早处置，在区间调控基础上加强定向调控、精准调控、相机调控，增强前瞻性、针对性、有效性，制定风险防范预案，提高政策执行效能，推出更多有利于提振有效需求、加强供给保障、稳定市场预期的实招硬招，努力以工作的确定性对冲外部环境的不确定性，提振各方面对我国发展的信心，确保经济平稳运行。

积极的财政政策要提升效能，更加注重精准、可持续。2022年赤字率拟按2.8%左右安排，比2021年有所下调，既保证财政支出强度，又增强财政可持续性、为应对可能出现的更为复杂局面预留充足空间。预计2022年财政收入继续增长，加之特定国有金融机构和专营机构上缴近年结存的利润、调入预算稳定调节基金等，可用财力明显增加，支出规模比2021年扩大2万亿元以上。新增财力要下沉基层，主要用于落实助企纾困和稳就业保基本民生政策，促进消费、扩大需求。强化对中小微企业、个体工商户、制造业、风险化解等的支持力度。实施新的组合式税费支持政策。优化支出重点和结构，加大对国家重大战略任务的财力保障，进一步支持保障和改善民生。中央对地方转移支付增长18%，比2021年大幅增加，将更多资金纳入直达范围，使基层落实惠企利民政策更有能力更有动力。党政机关坚持过紧日子，严肃财经纪律，坚决严控新建楼堂馆所，从严控制一般性支出。坚决遏制新增地方政府隐性债务。

稳健的货币政策要灵活适度，保持流动性合理充裕。发挥货币政策工具的总量和结构双重功能，为实体经济提供更有力支持。2022年保持广义货币供应量（M2）余额、社会融资规模存量增速与国内生产总值名义增速基本匹配，保持宏观杠杆率基本稳定。完善货币供应调控机制，健全市场化利率形成和传导机制，进一步降低实体经济综合融资成本。进一步疏通货币政策传导机制，促进货币信贷合理增长，优化信贷结构，用好再贷款、再贴现和定向直达工具，引导金融机构加大对实体经济特别是制造业、小微企业、科技创新、绿色发展的支持。利用多层次资本市场加大融资支持力度，加大股权融资比例，促进实现金融与科技、产业良性循环。完善外汇市场宏观审慎管理和微观监管，保持人民币汇率在合理均衡水平上的基本稳定。构建防范化解金融风险长效机制，牢牢守住不发生系统性金融风险的底线。

就业优先政策要提质加力。坚持实施就业优先战略，健全就业影响评估机制，在推动高质量发展中强化就业优先导向。大力拓宽就业渠道，注重通过稳市场主体来稳就业，增强创业带动就业作用。财政、金融等政策都要围绕就业优先实施，加大对企业稳岗扩岗的支持力度。调整完善部分减负稳岗扩就业政策，使用1000亿元失业保险基金支持稳岗和职业技能培训，持续推进创业带动就业，落实好创业担保贷款及贴息政策，深入实施提升就业服务质量工程。继续做好高校毕业生、退役军人、农民工等重点群体就业工作，加强脱贫易致贫劳动力就业帮扶，加大对就业困难人员、残疾人等就业援助力度，确保零就业家庭动态清零。建立促进多渠道灵活就业机制，支持和规范发展新就业形态，推进新就业形态就业人员职业伤害保障试点，维护新就业形态劳动者权益。深入实施技能中国行动，开展大规模多层次职业技能培训，共建共享一批公共实训基地，落实好扩大高校毕业生参加职业技能培训相关政策。健全全方位公共就业服务体系。加强就业监测和失业预警，切实防范化解规模性失业风险，努力实现更加充分更高质量就业。

同时，统筹高效做好煤电油气运调节。充分发挥煤电油气运保障工作部际协调机制作用，压实地方政府、部门、企业责任，坚持先立后破、民生优先，确保能源安全供应。增强国内资源生产保障能力，加快油气、矿产等资源勘探开发，巩固发展增储上产良好势头。保障电煤供应，加大重点产煤地区和运力紧张地区的电煤运输保障，完善煤炭市场价格形成机制，提升电力保障能力，确保稳定可靠充足供应，完善新能源上网电价机制，提高天然气气源保障水平。有序推进能源结构调整优化。实施全面节约战略，加强精细化用能管理，确保民生和公共用能需求，推动能效低于基准水平的重点行业企业有序实施改造升级，推动全社会加强节约用电用能。完善国家战略物资储备制度。

三、2022年国民经济和社会发展计划的主要任务

2022年，要按照党中央、国务院决策部署，突出重点、把握关键，着力做好十方面工作。

（一）加强和改善宏观调控，有效扩大国内需求。优化宏观政策组合，找准政策着力点，增强投资对优化供给结构的关键性作用，发挥消费对经济发展的基础性作用，释放内需潜力，激发国内超大规模市场优势，畅通国内大循环，为稳定宏观经济大盘提供坚实基础。

一是完善减负纾困等政策。坚持阶段性措施和制度性安排相结合，减税与退税并举。延续实施扶持制造业、小微企业和个体工商户的减税降费政策，并提高减免幅度、扩大适用范围，对小规模纳税人阶段性免征增值税，对小微企业年应纳税所得额100万元至300万元的部分，再减半征收企业所得税。对增值税留抵税额实行大规模退税，重点支持制造业，优先安排小微企业。预计全年减税退税约2.5万亿元，其中留抵退税约1.5万亿元。延续实施阶段性降低失业保险、工伤保险费率政策。开展涉企违规收费专项整治行动，坚决查处乱收费、乱罚款、乱摊派。进一步推动解决中小微企业融资难题，扩大普惠金融覆盖面，用好普惠小微贷款支持工具，继续增加支农支小再贷款，促进中小微企业融资增量、扩面、降价，推动普惠小微贷款余额明显增长，持续增加首贷和信用贷款发放。推动金融系统通过降低利率、减少收费等多种措施，向实体经济让利。引导金融机构优化信贷管理，继续对受疫情影响严重的行业企业给予融资支持，避免出现行业性盲目限贷、抽贷、断贷。推动降低企业生产经营成本，清理转供电环节不合理加价，支持地方对特殊困难行业用电实行阶段性优惠政策，加大拖欠中小企业账款清理力度，引导大型平台企业降低收费，减轻中小商户负担。

二是积极扩大有效投资。2022年拟安排地方政府专项债券3.65万亿元，安排中央预算内投资6400亿元。高质量建设“十四五”规划102项重大工程项目。坚持“项目跟着规划走，资金、要素跟着项目走”，加快推进在建工程，抓紧新开工一批成熟的项目，强化项目谋划储备，适度超前布局基础设施项目。做好土地、用能等要素保障，对国家重大项目实行能耗单列，加强项目调度和监管。扩大战略性新兴产业投资，支持企业扩大设备更新和技术改造投资。补齐农业农村、水利、市政工程、防灾减灾、应急保障等领域短板，推进城镇老旧小区改造，有序推进城市更新。推进城市燃气管道等老化更新改造，加强城市内涝治理，实施中西部中小城市基础网络完善工程。完善沿边抵边城镇体系和基础设施。深化投融资体制机制改革，鼓励和引导民间资本参与市政、交通、水利、生态环境、社会事业等补短板项目建设。规范有序推广政府和社会资本合作（PPP）模式，加快推进基础设施领域不动产投资信托基金（REITs）试点，推动盘活存量资产，形成投资良性循环。

三是促进消费持续恢复。进一步释放消费潜力，继续培育消费热点。稳定和扩大汽车等大宗消费，推动汽车等消费品由购买管理向使用管理转变，支持新能源汽车消费，持续开展新能源汽车下乡，加强城镇停车设施和充电桩、换电站等配套设施建设。发展服务消费，放宽服务领域市场准入。落实带薪休假制度。拓展消费新场景，培育壮大智慧零售、数字文化、智慧旅游等新型消费，促进网络消费，推动直播电商规范健康发展。推动实施冰雪旅游发展行动计划。鼓励有条件的地方开展绿色智能家电下乡和以旧换新，开展绿色建材下乡活动。倡导简约适度、绿色低碳的生活方式和消费模式。促进老字号创新发展。培育发展银发经济，开发适老化技术和产品。推进国际消费中心城市培育建设。推动打造区域消费中心。鼓励有条件的城市发展夜间经济。健全社区商业配套设施，打造城市便民生活圈，完善县域商业网络，加快贯通县乡村电子商务体系和快递物流配送体系。加大消费品质量安全监管力度，打击假冒伪劣，强化消费者权益保护，进一步改善消费环境。

四是适度超前开展重大基础设施建设。精准有效推进交通、水利、物流、能源、新型基础设施、民生等领域重大基础设施项目建设。推进川藏铁路、西部陆海新通道、沿江高铁、一批铁路专用线重点项目和川藏公路G318线提质改造等加快建设。整体推进京津冀、长三角、粤港澳大湾区城市群城际铁路、市域（郊）铁路建设。鼓励推广轨道交通+土地综合开发模式经验。做好中长期铁路网、国家公路网和全国港口与航道布局规划修编，强化国家公路、港口和高等级航道网络功能。推动三峡水运新通道研究论证。实施国家水网重大工程。加快发展公铁水多式联运。提升枢纽机场综合保障能力和服务水平，加快培育支线航空市场，提高国际航空货运能力。加快“支点城市+骨干走廊”现代流通网络建设。稳步推进国家物流枢纽、国家骨干冷链物流基地建设，强化冷链运输环节疫情防控措施，完善“321”冷链物流运行体系。加快物流业制造业融合创新发展，积极推动多式联运、智慧物流等新业态新模式发展。布局一批新型基础设施项目，加快推进5G、千兆光网、一体化大数据中心、北斗产业化重大工程、民用空间基础设施等建设，推动传统基础设施智能化改造。

（二）深入推进重点领域和关键环节改革，着力激发发展内生动力。突出增强发展动力、加快制度建设、促进共建共享、实现安全稳定，持续深化改革攻坚。

一是扎实推进高标准市场体系建设。加快建设全国统一大市场，持续完善产权保护、市场准入、公平竞争、社会信用等市场体系基础性制度，做好要素市场化配置综合改革试点。建立健全数据基础制度，加快培育数据要素市场，促进数据要素高效合规流通使用。落实深圳建设中国特色社会主义先行示范区放宽市场准入特别措施，支持浦东新区高水平改革开放、打造社会主义现代化建设引领区。坚持“两个毫不动摇”，依法保护各类市场主体产权和合法权益，一视同仁，平等对待，增强各类市场主体活力。推进国有资本布局优化和结构调整，积极稳妥深化国有企业混合所有制改革，全面完成国企改革三年行动。优化民营经济发展环境，支持民营企业融入和服务国家重大战略，保障民营企业依法平等使用资源要素、公开公平公正参与竞争、同等受到法律保护，及时有针对性地出台惠企纾困政策，持续营造有利于企业家成长的良好环境。打造大企业与中小微企业共生共荣的发展生态，加快构建扶持个体工商户发展的政策体系和长效机制。推动完善招标投标法律法规和制度规则，全面推行招标投标全流程电子化，编制电子招标投标技术标准和数据规范。坚持公共资源交易平台公共服务定位，持续深化交易平台整合共享和功能拓展，不断提升信息资源共享和应用水平。

二是深化重点领域体制改革。深化预算绩效管理改革，增强预算的约束力和透明度。推进省以下财政体制改革。推动增值税、消费税、关税等税收立法，稳妥推进后移部分品目消费税征收环节改革。完善税收征管制度，依法打击偷税骗税。持续深化资本市场改革，全面实行股票发行注册制。稳步推进垄断行业体制改革，深化能源、电信、公用事业等竞争性环节以及铁路行业等市场化改革，加快建设全国统一电力市场，推进省级管网以市场化方式融入国家管网，打造油气“全国一张网”。推进燃气发电、核电上网电价形成机制改革，完善风电、光伏发电新能源价格形成机制，完善高耗能行业阶梯电价政策，完成第三监管周期输配电价改革。扎实做好跨省天然气管道定价工作，深入推进农业水价综合改革，健全完善污水、垃圾处理收费机制。深化土地管理制度改革，增强对优势地区高质量发展保障能力。

三是打造市场化法治化国际化营商环境。持续推进“放管服”改革，对取消和下放审批事项要同步落实监管责任和措施。继续扩大市场准入。建立健全营商环境法规体系，持续抓好《优化营商环境条例》落实工作，建立损害营商环境案例归集通报制度。规范和用好营商环境评价机制，适时在全国组织开展营商环境评价。探索制定营商环境评价国家标准。推进长三角等重点区域优化营商环境一体化发展，复制推广优化营商环境典型经验和创新做法。全面实行行政许可事项清单管理。加强数字政府建设，推动政务数据共享，基本实现电子证照全国互认互通。强化政府监管责任，严格落实行业主管部门、相关部门监管责任和地方政府属地监管责任。加快建立健全全方位、多层次、立体化监管体系，实现事前事中事后全链条全领域监管，提高监管效能。强化反垄断和反不正当竞争，深入推进公平竞争政策实施。

（三）坚持高水平科技自立自强，强化科技创新支撑作用。深入实施创新驱动发展战略，依靠科技创新提升产业发展水平，培育壮大新动能。

一是加快关键核心技术攻关。实施基础研究十年规划，加强长期稳定支持。实施科技体制改革三年攻坚方案。完善关键核心技术攻关机制，加强基础研究和应用基础研究，加快布局生物医药、高端仪器、关键信息系统、能源绿色低碳转型发展、基础软件等基础和前沿技术研发，支持大型医疗设备、高端医用耗材研发，同步推进标准制定和实施，畅通源头创新、成果转化、市场应用链条。继续组织实施重大技术装备攻关工程。推进科研院所改革，完善重大科技项目立项和管理方式。完善人才发展体制机制，加大对青年科研人员支持力度。

二是加快打造高水平创新平台。加强国家实验室建设，稳步推进重组全国重点实验室。加大力度支持北京、上海、粤港澳大湾区国际科技创新中心建设，持续提升综合性国家科学中心创新能级，推动一批显示度高、带动性强、影响力大的国家重大科技基础设施项目尽快落地建设。突出产业需求引领，强化建设产业创新中心、工程研究中心、制造业创新中心、技术创新中心等创新平台，加大产业共性技术供给，大力发展工业软件，推动解决“卡脖子”问题，支撑和引领产业创新发展。

三是推进战略性新兴产业蓬勃发展。深入实施战略性新兴产业集群工程，系统推进生物、新材料、航空航天、海洋装备、新能源等新兴产业持续创新发展。大力推进北斗导航产业发展，促进光伏产业健康有序发展，稳慎推进氢能产业发展。积极前瞻布局未来产业，探索开展未来产业应用场景建设和示范迭代。

四是推动数字经济健康发展。统筹推动5G网络、人工智能、大数据等新型基础设施布局，谋划一批带动作用突出的重大基础设施和应用示范工程。协同推进数字产业化和产业数字化，打造数字经济新优势。维护网络安全。健全完善规则制度，提升监管能力和水平，推动平台经济规范健康持续发展。持续深入推进国家数字经济创新发展试验区建设，实施数字化转型试点。发展智慧城市、数字乡村。推动数字丝绸之路建设。加快构建全国一体化大数据中心体系，促进算力资源布局优化。

五是激发市场主体创新创造活力。加快构建龙头企业牵头、高校院所支撑、各创新主体相互协同的创新联合体。加大研发费用加计扣除政策实施力度，将科技型中小企业研发费用加计扣除比例从75%提高到100%，对企业投入基础研究实行税收优惠。完善科技创新的金融支持体系，继续加强知识产权保护和运用。推进知识产权质押融资等全链条服务。鼓励和支持技术入股。聚焦产业链供应链安全稳定，布局组建创新创业生态培育中心，推动大中小企业融通创新。深入推进大众创业万众创新，组织实施创业带动就业示范行动，扶持中小企业创业，带动重点群体就业。高质量办好双创活动周系列活动，增强双创平台服务能力。完善优质中小企业梯度培育体系，激发涌现一大批“专精特新”企业，在资金、人才、孵化平台搭建等方面给予大力支持。全面推动科技政策扎实落地，增强科技创新动力。大力推进全面创新改革试验，破解制约创新创业的体制机制障碍。

（四）促进产业链供应链循环畅通，巩固壮大实体经济根基。坚持把发展经济着力点放在实体经济上，促进工业经济平稳运行和提质升级。

一是保障产业链供应链安全稳定。建立完善产业链供应链苗头性问题预警机制，加强风险分析研判，积极应对突发情况，及时处置潜在风险。引导芯片制造企业有序扩大产能，稳定和畅通国内外供应渠道，推进供应链精准对接。做好需求少的高精尖技术精准供给。密切跟踪研判部分原材料供应和价格运行情况，引导供应链上下游稳定原材料供应和产销配套协作。强化大宗商品储备调节，加快推进国家大宗商品储运基地建设。加快构建现代国际物流供应链体系。聚焦新能源汽车、医疗装备等重点领域，实施重点领域“1+N”产业链供应链贯通工程，促进产业链供应链贯通发展。

二是着力振作工业经济运行。落实好振作工业经济运行、推动工业高质量发展实施方案和促进工业经济平稳增长的若干政策。加大中小微企业设备器具税前扣除力度。推动制造业中长期贷款继续保持较快增长。做好重要原材料和初级产品保供稳价，加强重大项目的用地、用能、环保等要素保障，精准打通产业链供应链堵点。加大对制造业企业特别是中小微企业的精准支持，释放重点领域消费潜力，推动企业技术改造，培育新业态新模式，促进工业经济行稳致远。

三是提升制造业核心竞争力。深入实施质量强国建设和产业基础再造工程，推动传统产业高端化、智能化、绿色化。推动重点工业领域节能降碳和绿色转型。实施先进制造业集群发展专项行动，支持国家新型工业化产业示范基地提升集群化发展水平，创建首批国家制造业高质量发展试验区。继续办好中国品牌日系列活动，高质量举办好中国品牌博览会和中国品牌发展国际论坛。

四是促进产业优化升级。修订《产业结构调整指导目录》。实施中国品牌新能源汽车发展行动。启动实施钢铁、有色、建材等重点领域企业节能降碳技术改造工程，持续优化石化产业布局。推动原料药产业绿色化、高端化发展。落实促进服务业领域困难行业恢复发展的若干政策，延续服务业增值税加计抵减政策，给予承租国有房屋租金减免，对餐饮、零售、旅游、公路水路铁路运输、民航等服务业实施财税金融等纾困扶持措施。加快发展现代服务业，支持制造服务业载体建设，抓好先进制造业和现代服务业融合发展试点，推动生活性服务业向高品质和多样化升级。

（五）坚持农业农村优先发展，落实落细乡村振兴各项举措。着力保障粮食安全，压茬推进乡村振兴战略，进一步巩固脱贫攻坚成果，持续推进农业农村现代化。

一是抓好粮食和重要农产品生产供应。实行粮食安全党政同责，严格落实地方粮食安全主体责任，坚决守住18亿亩耕地红线，划足划实永久基本农田，切实遏制耕地“非农化”、防止“非粮化”。确保全年粮食播种面积稳定在17.6亿亩以上，促进大豆和油料增产，巩固玉米产能恢复势头。加强中低产田改造，新建1亿亩高标准农田，加强高标准农田建设管理，确保到2022年底完成10亿亩高标准农田建设任务。因地制宜新建一批现代化灌区，全力做好水旱灾害防御。健全粮食产购储加销协同保障机制，加强粮食质量安全监管。加大农资保供稳价力度，给种粮农民再次发放农资补贴。适当提高稻谷、小麦最低收购价。深入推进优质粮食工程，推动粮食产业高质量发展。加强粮食应急保障体系建设。加强重点区域粮食接卸、仓储和集疏运体系建设。深入实施种业振兴行动，推进现代种业提升工程建设，加快种源关键核心技术攻关。加强农业科技攻关和推广应用，提高农机装备水平。加强农业社会化服务体系建设。深入实施国家黑土地保护工程，加大盐碱地综合利用力度。稳定生猪生产长效性支持政策，推进草原畜牧业转型升级，提高畜产品供给能力。做好粮油棉糖化肥总量平衡和市场调控，有效开展储备吞吐和进口调节。

二是全面巩固脱贫攻坚成果。强化易返贫致贫人口精准监测和及时帮扶，做好巩固脱贫成果后评估工作，确保不发生规模性返贫和新的致贫。加大易地扶贫搬迁后续扶持力度，推动大型搬迁安置区和进城安置群众融入新型城镇化建设。继续在农业农村基础设施建设领域大力推广以工代赈方式，提高劳务报酬发放比例。组织创建消费帮扶示范城市和产地示范区，健全消费帮扶助力乡村振兴长效机制。着力改善脱贫地区农业基础设施条件，发展优质高产高效农业、优势特色农业，提升农业质量效益和竞争力。支持脱贫地区创建国家农村产业融合发展示范园。加大对国家乡村振兴重点帮扶县的支持力度，在规划政策、资金投入等方面给予倾斜。加大对灾害多发、条件艰苦山区开展灾害防治、生态保育、乡村振兴协同融合发展的支持力度。

三是实施乡村建设行动。加强乡村规划建设管理，优化乡村生产生活生态空间。促进城乡融合发展，推动建立健全县域内城乡一体的就业、教育、医疗、养老等政策体系，在有条件的地方推进县乡村公共基础设施建设运营管护一体化。加强农村公路等基础设施和公共服务设施建设。继续实施农村供水保障和农村电网巩固提升工程。深入实施农村人居环境整治提升五年行动，统筹推进农村改厕和生活污水治理，开展农村生活垃圾分类与资源化利用，整治提升村容村貌。稳妥审慎开展农村宅基地制度改革试点，持续推进农村集体产权制度改革，推动农村集体经济组织立法，发展壮大新型农村集体经济。加强乡村振兴人才队伍建设。稳妥推进农村集体经营性建设用地入市。持续完善乡村治理体系。

（六）扎实推动区域协调发展和新型城镇化建设，完善区域协调发展新机制。坚持实施区域重大战略、区域协调发展战略、新型城镇化战略，加快构建高质量发展的动力系统。

一是深入实施区域重大战略。有力推动京津冀协同发展，高标准高质量建设雄安新区，稳妥有序推进率先启动的疏解项目在雄安新区落地，落实支持北京城市副中心高质量发展的意见，加快天津港北方国际航运枢纽建设。扎实推动长江经济带高质量发展，深入推进污染治理“4+1”工程，持续巩固长江禁捕和退捕渔民安置保障工作成果，积极构建生态产品价值实现机制政策制度体系，建立长江流域协调机制和水生态考核机制，完善综合交通运输体系，力争全线开工建设沿江高铁，大力发展多式联运。积极稳妥推进粤港澳大湾区建设，坚持基础设施“硬联通”和规则机制“软联通”并举，加快推进大湾区市场一体化进程。加快推进横琴粤澳深度合作区高质量发展，支持前海深港现代服务业合作区改革开放。深化通关模式改革，落实落细教育、医疗、养老、交通等领域政策，进一步便利港澳居民在大湾区内地学习工作生活。提升长三角一体化发展水平，深入推进基础设施公共服务等重点领域一体化发展，稳步推进长三角生态绿色一体化发展示范区、皖北承接产业转移集聚区等重点区域建设，推动科创产业深度融合，积极稳妥推动港航资源整合。扎实推进黄河流域生态保护和高质量发展，严控高耗水作物种植面积，大力推进沿黄河大中型灌区现代化改造，紧抓环境污染“3+1”综合治理，加快中游地区水土流失综合治理，加快补齐城镇污水收集管网和处理设施短板，推进尾矿库环境风险隐患排查整治，加强沿黄通道建设，推动建设宁夏黄河流域生态保护和高质量发展先行区。

二是提高区域协调发展水平。推动西部大开发形成新格局，发挥风、光、水电和矿产资源优势建设大型清洁能源基地。积极发展文化和旅游产业。实施重要生态系统保护和修复工程，加快重大项目规划建设。推进贵州大数据综合试验区建设。积极稳妥推进雅鲁藏布江下游水电开发等重大工程。推动东北振兴取得新突破，组织实施好东北全面振兴“十四五”实施方案，继续推进黑瞎子岛中俄国际合作示范区建设，积极推动大小兴安岭林区生态保护与经济转型，稳步推进长吉图开发开放先导区发展，推动《东北振兴重点项目三年滚动实施方案》重点项目加快建设。推动中部地区高质量发展，推动湘鄂赣协同高质量发展，支持晋陕豫黄河金三角承接产业转移示范区建设，支持山西进一步深化能源革命综合改革试点，推进淮海合作区协同发展，支持中国（武汉）光谷建设，编制出台洞庭湖生态经济区规划。鼓励东部加快高质量发展，支持福建高质量发展及平潭综合实验区建设，推动山东新旧动能转换综合试验区建设取得新进展。支持民族地区改善生产生活条件，加强各民族交流交往交融，铸牢中华民族共同体意识。推动革命老区、边境地区、生态退化地区、资源型地区、老工业城市等特殊类型地区振兴发展，推进国家级新区、临空经济示范区、承接产业转移示范区等功能平台建设，推动国家自主创新示范区和高新区高质量发展。支持海洋经济发展示范区、现代海洋城市等建设，提升海洋资源开发利用水平。

三是提升新型城镇化建设质量。持续推进农业转移人口市民化，促进在城镇稳定就业生活的农业转移人口举家进城落户。持续优化城镇化空间布局，更好发挥超大特大城市对周边市县辐射带动作用，培育形成现代化都市圈。持续推进成渝地区双城经济圈建设，印发推动长江中游、北部湾等城市群发展“十四五”实施方案。推进以县城为重要载体的城镇化建设，加强县城基础设施建设，增强综合承载能力，健全公共设施体系。健全特色小镇清单管理机制，统筹加强典型引路和规范纠偏。

（七）扩大高水平开放，推动外资外贸平稳发展。建设更高水平开放型经济新体制，坚持对内开放与对外开放相结合，全面提升开放平台能级，培育国际合作竞争新优势。

一是多措并举稳定外贸。加大对中小微外贸企业精准帮扶。大力发展跨境电商、市场采购等外贸新业态新模式。构建完善海外仓标准体系，不断完善覆盖全球的海外仓网络。视情扩大先进技术、重要设备、关键零部件、能源资源产品、紧缺农产品等进口，加大中国品牌海外推介力度，支持高技术、高质量、高附加值等产品出口。推动数字贸易创新发展。落实进口环节税收和出口退税政策。支持内陆开放试验区提升能级。建设国家服务贸易创新发展示范区和数字贸易示范区。高质量建设国家文化出口基地。在自贸试验区和全国推进实施跨境服务贸易负面清单，在自贸试验区和海南自由贸易港等试点对接国际高标准推进制度型开放。

二是提高利用外资水平。出台实施关于建设更高水平开放型经济新体制、促进构建新发展格局的意见。实施好新版外资准入负面清单，修订扩大《鼓励外商投资产业目录》，引导更多外资投向先进制造业、高技术等行业领域和中西部、东北地区。落实好外资企业国民待遇，推动重大外资项目加快落地。增设服务业扩大开放综合试点。优化企业外债分类管理，完善对房地产、地方融资平台、低信用企业的外债调控，持续防范外债风险。

三是提升境外投资效益。持续推动境外重大项目合作，探索产能与投资合作新方式。优化境外投资布局，深化双边和第三方市场合作。引导金融机构服务重点领域和项目。深入实施境外投资提质效防风险系列措施，提高重点主体境外投资水平，规范企业境外经营行为，支持行业协会商会建立境外投资自律机制。

四是推动共建“一带一路”高质量发展。扎实推进基础设施互联互通，加强重大项目合作，注重境外项目风险防控。深入推进地方参与共建“一带一路”高质量发展，推动新疆、福建“一带一路”核心区建设走深走实。推动“丝路海运”、“冰雪丝路”加快发展。加强中欧班列通道能力、枢纽节点、口岸扩能建设。完善“一带一路”风险防控和安全保障体系。稳妥开展健康、绿色、数字、创新等领域合作，推动境外煤电等“两高”项目绿色低碳转型发展。

五是推进海南自由贸易港建设。加快口岸和综合执法点的基础设施规划建设等，为实现全岛封关运作创造条件。支持口岸、路网等基础设施建设。大力发展外向型经济，不断夯实重大平台和产业发展基础，聚焦重点政策，依托重点项目，以更大力度探索贸易投资自由化便利化规则。进一步强化重大风险防控工作，加大高水平压力测试力度，建立健全风险预警、研判和处置联防联控机制。

六是推动多双边经贸合作。坚定维护多边贸易体制，积极参与世界贸易组织（WTO）改革。高质量实施《区域全面经济伙伴关系协定》（RCEP），积极推进加入《全面与进步跨太平洋伙伴关系协定》（CPTPP）和《数字经济伙伴关系协定》（DEPA）。推动与更多国家和地区商签高标准自贸协定，加强互利合作。推进落实全球发展倡议，推动国际发展合作。积极参与联合国、二十国集团（G20）、亚太经合组织（APEC）、金砖国家等多边机制合作。

（八）深入推进生态文明建设，扎实推动绿色低碳发展。建立健全绿色低碳循环发展经济体系，推动减污降碳协同增效，促进生态环境持续改善、资源利用效率不断提高。

一是加快推进经济绿色转型。完善绿色技术创新体系，建设国家绿色技术交易中心。建设绿色产业示范基地，修订绿色产业指导目录，大力发展节能环保产业。实施“十四五”清洁生产推行方案，在重点行业开展清洁生产改造。完善绿色金融标准体系和评价机制，发挥国家产融合作平台作用，引导金融资源向工业绿色低碳领域汇聚。深化环境信息依法披露制度改革，推动对企事业单位开展环保信用评价。持续实施国家节水行动，开展城市公共供水管网漏损治理工程，推动老城区、城乡结合部、县城、乡镇污水收集管网建设，加强京津冀、黄河流域、西北地区等重点区域、流域水资源节约集约利用，建设海水淡化示范城市。抓好全链条粮食节约减损。推进废旧物资循环利用体系建设和可循环快递包装规模化应用试点，组织开展大宗固体废弃物综合利用示范基地建设和骨干企业培育，推进废钢铁、废有色金属、废弃机动车、废旧家电、废塑料、厨余垃圾等城市废弃物分类利用和集中处置。

二是积极稳妥推进碳达峰碳中和。完善碳达峰碳中和“1+N”政策体系，实施“碳达峰十大行动”，坚持“全国一盘棋”。大力发展非化石能源，建设大型风电、光伏基地项目，积极发展分布式新能源，积极推动海上风电集群化开发，稳妥推进西南等地大型水电基地建设，大力发展抽水蓄能，在确保绝对安全的前提下积极有序发展核电，推进煤炭清洁高效利用。提升电网对可再生能源发电的消纳能力。做好全国碳排放权交易市场第二个履约周期管理。实施节能降碳改造升级。积极研究开发碳捕捉利用与封存技术。优化完善能耗双控政策，合理增加能耗总量弹性，新增可再生能源和原料用能不纳入能源消费总量控制，推动能耗“双控”向碳排放总量和强度“双控”转变。全面加强能源资源节约，强化工业、交通和建筑节能，不断降低单位产出能耗物耗和碳排放。对标能效标杆水平，推动能效低于基准水平的重点行业企业，有序实施改造升级，促进高耗能行业高质量发展。继续做好碳达峰碳中和及应对气候变化国际谈判与合作，主动参与全球绿色治理体系建设，加强绿色经贸、技术与金融合作。

三是深入打好污染防治攻坚战。加强PM2.5和臭氧协同控制，强化多污染物协同控制和区域协同治理。深入治理城乡黑臭水体，继续开展入河入海排污口排查整治，加强重点海域污染防治。持续推进土壤和地下水污染防治。稳步推进“无废城市”建设，因地制宜推进生活垃圾分类和减量化、资源化，持续抓好塑料污染全链条治理。建设污水资源化利用示范城市，建设节能低碳标杆污水处理厂。

四是持续强化生态系统治理。坚持山水林田湖草沙一体化保护和系统治理，实施重要生态系统保护和修复重大工程，保护生物多样性。继续开展大规模国土绿化行动，加强以国家公园为主体的自然保护地体系建设，把公益保护不断做深做实。深入推进国家生态文明试验区建设，复制推广福建、江西、贵州、海南的经验。研究出台《生态保护补偿条例》，加快建立健全生态产品价值实现机制，探索建立农林碳汇产品价值实现机制。

（九）有效防范化解重点领域风险，切实维护经济安全。统筹发展和安全，不断加强风险预警、防控机制和能力建设，守住不发生系统性风险的底线。

一是加强财政金融领域风险防控。持续推进防范化解地方政府隐性债务风险，坚决遏制隐性债务增量。稳妥处置一些地方的高风险金融机构，设立金融稳定保障基金，运用市场化、法治化方式化解风险隐患。稳妥有序缓释债券市场风险。加快不良贷款处置。妥善应对主要经济体货币政策转向的溢出风险。发挥资本作为生产要素的积极作用，同时有效控制其消极作用，为资本扩张设置“红绿灯”，支持和引导资本规范健康发展，依法加强对资本的有效监管，防止资本无序扩张、野蛮生长。

二是提升能源资源安全保障能力。深入推进煤电油气产供储销体系建设。推进石油储备重大工程建设，加快华北、西北等地下储气库新建和扩容达容建设。有序核增一批煤炭先进产能，加快提升煤炭储备能力。加强抽水蓄能等调峰电源建设，继续发挥传统能源特别是煤炭、煤电的兜底保供和调峰作用，积极推进现有煤电项目改造升级。推进白鹤滩、羊曲等重大水电工程建设。加大力度规划建设以大型风光电基地为基础、以其周边清洁高效先进节能的煤电为支撑、以稳定安全可靠的特高压输变电线路为载体的新能源供给消纳体系。加快推进国家储备建设，健全统一的战略和应急物资储备体系。

三是推动房地产市场平稳发展。坚持房子是用来住的、不是用来炒的定位，因城施策做好房地产调控，稳地价、稳房价、稳预期。扎实推进高风险房地产企业风险处置。加快发展长租房市场，推进人口净流入的大城市保障性租赁住房建设，加大住房公积金对租赁住房的支持力度，着力解决新市民、青年人等群体的住房困难问题。扩大灵活就业人员参加住房公积金制度试点。实施好差别化住房信贷政策，支持商品房市场更好满足购房者的合理住房需求。

（十）围绕切实保障和改善民生，扎实办好民生实事。正确认识和把握实现共同富裕的主要目标和实践途径，坚持尽力而为、量力而行，不断提升公共服务水平，着力解决人民群众普遍关心关注的民生问题。

一是强化就业优先导向。加快推动“十四五”就业促进规划重点任务落地落实。引导各地加大对返乡入乡创业园建设的支持力度。推动做好公共实训基地项目储备、建设和运营，推动县域公共职业技能培训资源共建共享，促进高质量产训结合。

二是着力发挥分配的功能和作用。完善工资合理增长机制，指导地方合理调整最低工资标准，拓宽城乡居民增收渠道，全面落实义务教育教师工资待遇。健全各类生产要素由市场决定报酬的机制，完善再分配机制。多措并举促进农民增收，依法治理拖欠农民工工资问题。

三是完善社会保障体系。稳步实施企业职工基本养老保险全国统筹制度，适当提高退休人员基本养老金和城乡居民基础养老金标准，确保按时足额发放。居民医保和基本公共卫生服务经费人均财政补助标准分别再提高30元和5元，推动基本医保省级统筹。进一步扩大药品、高值医用耗材带量采购范围，健全重大疾病医疗保险和救助制度，优化医保领域便民服务。推进失业保险省级统筹，统一农民工和城镇职工参保缴费办法。健全退役军人保障体系。深入开展新就业形态就业人员职业伤害保障试点。健全分层分类的社会救助体系，建立完善社会救助主动发现机制，加强低收入人口动态监测及常态化救助帮扶机制建设。加强困难群众基本生活保障，完善最低生活保障制度。保障妇女儿童合法权益，完善残疾人、孤儿等社会福利制度。

四是完善公共服务政策制度体系。分类推进基本公共服务均等化发展，推动服务重心向基层下沉，增加普惠性非基本公共服务供给。支持社会力量增加普惠性托育服务供给，进一步完善学前教育收费政策，推进普惠性学前教育资源扩容增效，推进儿童友好城市建设。推动义务教育优质均衡发展和城乡一体化，提升农村学校办学能力，依据常住人口规模增加城镇教育资源。继续做好义务教育阶段减负工作。加强县域普通高中建设。改善职业教育办学条件。推进高校“双一流”建设，加快经济社会发展重点领域急需学科专业建设、人才培养和科技攻关，完善产教融合政策措施。支持中西部高等教育发展，高校招生继续加大对中西部和农村地区倾斜力度。加强教师队伍建设，加大中西部地区教师培训力度。办好特殊教育、继续教育，规范民办教育发展。完善三孩生育政策配套措施，将3岁以下婴幼儿照护费用纳入个人所得税专项附加扣除，推动生育支持政策落地见效。贯彻落实积极应对人口老龄化国家战略，加强养老托育服务体系和健康支撑体系建设，维护好老年人合法权益。推动公立医院高质量发展，加快健全现代医院管理制度，深入推进国家医学中心、国家区域医疗中心建设项目和县级医院建设，加快完善分级诊疗体系，深化医疗服务价格、医保支付管理和公立医院薪酬制度改革。补齐妇幼儿科、精神卫生、老年医学等服务短板。持续深入开展爱国卫生运动、推进实施健康中国行动，倡导文明健康绿色环保生活方式。实施现代化疾控体系建设、国家重大传染病防治基地建设等工程，提升公共卫生防控救治能力。加快中医药特色发展。推动中华优秀传统文化创造性转化和创新性发展，促进出版、电影、广播电视、文物等事业高质量发展，推进公共文化数字化建设，提供更多公共文化产品。加强国家重大文化项目和国家文化公园建设，加强重要文化遗产和国家公园等重要自然遗产保护设施建设，全面提升革命文物保护利用水平，促进公共文化服务提质升级。推进国民旅游休闲加快发展，不断完善节假日制度。实施全民健身设施补短板工程，加强体育公园等全民健身场地设施建设。深化公共法律服务体系建设。

与此同时，继续做好常态化疫情防控，坚持外防输入、内防反弹，不断优化完善防控措施，科学精准高效处置局部突发疫情。一是积极做好疫苗接种和药物研发。加快推进新冠病毒疫苗研发，科学组织不同技术路线疫苗的序贯免疫研究，进一步提高疫苗加强针接种人群覆盖面，根据疫情防控需要进一步加快新冠病毒药物国内研发上市，统筹做好生产、储备、质量监管等工作。二是强化外防输入举措。加强口岸疫情防控能力建设，严格落实人防、物防、技防相关举措，强化口岸及集中隔离点疫情防控。做好对相关国家地区进口货物特别是冷链产品的检测消毒工作。对高风险岗位人员切实做到闭环管理，建立健全进口冻品集中监管制度。三是健全优化疫情防控体系。加强流调溯源、核酸筛查、隔离管控和重点场所防控，着力做好节假日等人员流动增多时期疫情防控，避免聚集性感染。完善落实农村地区疫情防控工作方案，强化医院感染防控。继续加强对散发病例和聚集性疫情的防范处置，最大限度减少对群众生产生活的影响。四是持续推进疫情防控国际合作。加快诊疗技术联合攻关，深入开展疫苗、药物等研发试验应用国际合作。力所能及开展疫苗援助和抗疫物资援助。加强跨境贸易合作，保障疫苗及原辅料贸易畅通。支持世界贸易组织就疫苗知识产权豁免早日作出决定，鼓励疫苗企业向发展中国家转让技术。

全面准确、坚定不移贯彻“一国两制”、“港人治港”、“澳人治澳”、高度自治的方针，维护国家主权、安全、发展利益和特别行政区社会大局稳定。支持香港、澳门发展经济、改善民生，更好融入国家发展大局，建设国际创新科技中心，打造共建“一带一路”功能平台，实现经济多元可持续发展，完善便利港澳居民在内地发展政策措施。深入贯彻落实新时代党解决台湾问题的总体方略，坚持一个中国原则和“九二共识”，坚决反对“***”分裂行径，坚决反对外部势力干涉，推进两岸关系和平发展和祖国统一，完善保障台湾同胞福祉和在大陆享受同等待遇的制度政策，加强两岸产业合作，打造两岸共同市场，壮大中华民族经济，共同弘扬中华文化。

2022年经济社会发展工作任务繁重，意义重大。我们要更加紧密地团结在以习近平同志为核心的党中央周围，高举中国特色社会主义伟大旗帜，以习近平新时代中国特色社会主义思想为指导，弘扬伟大建党精神，增强“四个意识”、坚定“四个自信”、做到“两个维护”，按照党中央、国务院决策部署，自觉接受全国人大的监督，认真听取全国政协的意见和建议，攻坚克难、砥砺奋进，努力完成全年目标任务，以实际行动迎接党的二十大胜利召开，为把我国建设成为富强民主文明和谐美丽的社会主义现代化强国、实现中华民族伟大复兴的中国梦不懈奋斗！

第十三届全国人民代表大会财政经济委员会关于2021年国民经济和社会发展

计划执行情况与2022年国民经济和社会发展计划草案的审查结果报告

（2022年3月8日第十三届全国人民代表大会第五次会议主席团第二次会议通过）

十三届全国人大五次会议主席团：

第十三届全国人民代表大会第五次会议审查了国务院提出的《关于2021年国民经济和社会发展计划执行情况与2022年国民经济和社会发展计划草案的报告》和2022年国民经济和社会发展计划草案。全国人民代表大会财政经济委员会在对计划报告和计划草案初步审查的基础上，根据各代表团和有关专门委员会的审查意见，作了进一步审查。国务院根据审查意见对计划报告作了修改。现将审查结果报告如下。

一、2021年计划执行情况总体良好

财政经济委员会认为，2021年国民经济和社会发展计划执行情况总体良好。面对复杂严峻的外部环境和国内经济下行压力进一步加大等困难、问题和挑战，在以习近平同志为核心的党中央坚强领导下，国务院和地方各级人民政府坚持以习近平新时代中国特色社会主义思想为指导，坚决贯彻落实党中央决策部署，认真执行十三届全国人大四次会议审查批准的2021年国民经济和社会发展计划，坚持稳中求进工作总基调，统筹疫情防控和经济社会发展，注重宏观政策跨周期调节，大力加强科技创新，稳定和优化产业链供应链，持续优化营商环境，强化和落实助企纾困政策，防范化解经济金融风险，巩固拓展脱贫攻坚成果，加强生态环境保护和污染防治，加大保民生补短板力度，推动经济运行保持在合理区间，奋力完成改革发展艰巨任务。经过不懈努力，年度计划的主要预期目标基本实现，全部61项计划指标完成情况较好，主要任务顺利完成，构建新发展格局迈出新步伐，推动高质量发展取得新成效，经济社会大局保持和谐稳定，实现了“十四五”良好开局。

同时也要看到，全球疫情仍在持续，地缘政治冲击国际经贸秩序，全球供应链趋紧，世界经济复苏放缓。国内经济下行压力凸显，宏观调控难度加大，实体经济困难较多，科技原始创新能力不足，重点领域风险隐患积聚，就业压力增大。计划执行中，重大政策综合影响评估有待加强，多重目标统筹协调不够，有的目标任务采用简单分解办法，有的在执行中存在传导不畅或层层加码、“一刀切”、运动式等情况。对这些困难、问题和挑战，要深入分析研判，加强系统协调，加大改革力度，不断增强应对的有效性。

二、2022年计划报告和计划草案总体可行

财政经济委员会认为，国务院提出的2022年计划报告和计划草案，以习近平新时代中国特色社会主义思想为指导，符合党的十九大和十九届历次全会精神，符合中央经济工作会议精神，符合“十四五”规划纲要目标要求，符合我国经济社会发展实际，对更趋复杂严峻的外部环境和国内需求收缩、供给冲击、预期转弱三重压力等作了认真分析，在主要任务中作了相应安排，国民经济和社会发展总体要求、主要预期目标、主要宏观政策取向、主要任务基本协调匹配，总体可行。

三、建议批准2022年计划报告和计划草案

财政经济委员会建议，第十三届全国人民代表大会第五次会议批准国务院提出的《关于2021年国民经济和社会发展计划执行情况与2022年国民经济和社会发展计划草案的报告》，批准2022年国民经济和社会发展计划草案。

四、做好2022年计划执行工作的建议

2022年将召开党的二十大，这是党和国家政治生活中的一件大事，做好计划执行工作意义重大。要在以习近平同志为核心的党中央坚强领导下，以习近平新时代中国特色社会主义思想为指导，全面贯彻落实党的十九大和十九届历次全会精神，贯彻中央经济工作会议精神，弘扬伟大建党精神，坚持稳中求进工作总基调，完整、准确、全面贯彻新发展理念，加快构建新发展格局，全面深化改革开放，坚持创新驱动发展，推动高质量发展，坚持以供给侧结构性改革为主线，统筹疫情防控和经济社会发展，统筹发展和安全，继续做好“六稳”、“六保”工作，持续改善民生，着力稳定宏观经济大盘，保持经济运行在合理区间，保持社会大局稳定，迎接党的二十大胜利召开。为此，财政经济委员会建议：

（一）把稳增长放在更加突出位置，更好把握调整政策和推进改革的时度效。各方面要坚持以经济建设为中心，坚持稳字当头、稳中求进，肯定成绩、正视困难、遵循规律、坚定信心，切实担负起稳定宏观经济的责任，积极推出有利于经济稳定的政策。推动有效市场和有为政府更好结合，最大限度调动各个方面稳增长的积极性，发挥好国家政策对稳预期、增信心的重要作用。着力做好上半年政策发力工作。建立政策措施统筹协调机制，把先立后破的原则落实到位，把握好各项政策的衔接。按照中央决策部署，做好碳达峰碳中和工作，科学推进绿色低碳发展。抓细抓实各项助企惠企措施的贯彻执行，确保政策实施顺畅通达、企业切实受惠。

（二）坚决实施好扩大内需战略，充分释放内需潜力。多措并举增加居民收入，进一步改善消费环境，促进新兴消费，推动绿色消费，拓展消费新场景。高质量实施“十四五”重大工程项目，强化项目要素保障，适度超前布局基础设施建设，提高专项债实际使用效果，积极扩大民间投资。促进区域协调发展，全面巩固拓展脱贫攻坚成果，接续推进乡村振兴，持续推进农业转移人口市民化，提升新型城镇化质量。

（三）深入推进改革扩大开放，提升经济增长潜能。进一步完善市场体系基础制度，着力推进要素市场化配置等改革。改革创新政府治理方式，加快打造市场化法治化国际化营商环境。落实好中央对科技工作的全面部署，强化科技创新支撑作用。深入实施公平竞争政策，依法精准开展反垄断和反不正当竞争工作，防止资本无序扩张。优化民营经济发展环境，依法保护各类市场主体产权和合法权益。扩大高水平对外开放，主动对标高标准国际经贸规则，积极应对国际市场变化，稳定外贸外资基本盘。

（四）防范化解重点领域风险，坚决守住不发生系统性风险的底线。坚决遏制新增地方政府隐性债务，稳妥处置高风险金融机构，做好跨市场跨行业风险处置工作。坚持“房住不炒”定位，促进房地产业良性循环和健康发展。提升重要能源资源保障能力，确保粮食等重要农产品供应安全。稳定和畅通产业链供应链，及时有效应对国际金融和大宗商品市场变动的影响。

（五）强化民生兜底保障，维护社会安全稳定。全力做好稳就业工作，高度重视高校毕业生、农民工、退役军人等重点群体及一些行业的就业问题。促进教育公平和质量提升，提高医疗卫生服务能力。认真实施并不断完善企业职工基本养老保险全国统筹制度实施方案，加快构建完善养老服务体系。统筹做好常态化疫情防控与经济社会发展，不断优化疫情防控措施。持续加强应急管理和防灾减灾，落实安全生产责任，保障社会公共安全。

以上报告，请审议。

第十三届全国人民代表大会财政经济委员会

2022年3月8日
