# Supplementary material for: Mapping the evolution of fertility support policies in China: A content and instrumental analysis
Source: PLoS One. 2025 Oct 9;20(10):e0332137. doi: 10.1371/journal.pone.0332137 (PMC12510515; doi:10.1371/journal.pone.0332137)
Supplement: S1 Appendix — (ZIP) [file pone.0332137.s001.zip › S1 Appendix. 226 original policy documents/205-第十三届全国人民代表大会第四次会议关于2020年国民经济和社会发展计划执行情况与2021年国民经济和社会发展计划的决议(FBM-CLI-1-353451).docx]

第十三届全国人民代表大会第四次会议关于2020年国民经济和社会发展计划执行情况与2021年国民经济和社会发展计划的决议

发布部门： 全国人民代表大会

发布日期：2021.03.11

实施日期：2021.03.11

时效性： 现行有效

效力级别： 工作文件

法规类别： 人大议事 营商环境优化

专题分类： 长江经济带发展

第十三届全国人民代表大会第四次会议关于2020年国民经济和社会发展计划执行情况与2021年国民经济和社会发展计划的决议

（2021年3月11日第十三届全国人民代表大会第四次会议通过）

第十三届全国人民代表大会第四次会议审查了国务院提出的《关于2020年国民经济和社会发展计划执行情况与2021年国民经济和社会发展计划草案的报告》及2021年国民经济和社会发展计划草案，同意全国人民代表大会财政经济委员会的审查结果报告。会议决定，批准《关于2020年国民经济和社会发展计划执行情况与2021年国民经济和社会发展计划草案的报告》，批准2021年国民经济和社会发展计划。

关于2020年国民经济和社会发展计划执行情况与2021年国民经济和社会发展计划草案的报告

--2021年3月5日在第十三届全国人民代表大会第四次会议上

国家发展和改革委员会

各位代表：

受国务院委托，现将2020年国民经济和社会发展计划执行情况与2021年国民经济和社会发展计划草案提请十三届全国人大四次会议审查，并请全国政协各位委员提出意见。

一、2020年国民经济和社会发展计划执行情况

2020年是新中国历史上极不平凡的一年。面对严峻复杂的国际形势、艰巨繁重的国内改革发展稳定任务特别是新冠肺炎疫情的严重冲击，以习近平同志为核心的党中央统揽全局，保持战略定力，准确判断形势，精心谋划部署，果断采取行动，付出艰苦努力，及时作出统筹疫情防控和经济社会发展的重大决策。各地区各部门坚持以习近平新时代中国特色社会主义思想为指导，全面贯彻党的十九大和十九届二中、三中、四中、五中全会精神，按照党中央、国务院决策部署，认真执行十三届全国人大三次会议审议批准的《政府工作报告》、2020年国民经济和社会发展计划，落实全国人大财政经济委员会审查意见，沉着冷静应对风险挑战，坚持高质量发展方向不动摇，统筹疫情防控和经济社会发展，扎实做好“六稳”工作，全面落实“六保”任务，我国经济运行逐季改善、逐步恢复常态，在全球主要经济体中唯一实现经济正增长，脱贫攻坚战取得全面胜利，决胜全面建成小康社会取得决定性成就，交出一份人民满意、世界瞩目、可以载入史册的答卷。

（一）坚持把人民生命安全和身体健康放在第一位，抗疫斗争取得重大战略成果。面对突如其来的新冠肺炎疫情带来的严峻考验，习近平总书记亲自指挥、亲自部署，团结带领全国各族人民迅速打响疫情防控的人民战争、总体战、阻击战，用1个多月的时间初步遏制疫情蔓延势头，用2个月左右的时间将本土每日新增病例控制在个位数以内，用3个月左右的时间取得武汉保卫战、湖北保卫战的决定性成果，此后又有效处置多起局部地区聚集性或散发疫情。

一是全力以赴做好疫情防控救治工作。按照坚定信心、同舟共济、科学防治、精准施策的总要求，坚持全国一盘棋，迅速成立中央应对疫情工作领导小组，向湖北派出中央指导组，充分发挥国务院联防联控机制作用，举全国之力开展武汉保卫战、湖北保卫战，快速阻断本土疫情传播。明确“四早”、“四集中”要求，费用全部由国家承担，着力提高收治率和治愈率、降低感染率和病亡率。开展联防联控和群防群控，各省（区、市）相继启动重大突发卫生事件一级响应，组织干部力量下沉抓好社区防控，引导各类社会组织、专业社会工作者和志愿服务力量依法有序参与疫情防控和社会服务。扎实做好医疗物资保障和生活必需品保供稳价工作，快速实现口罩等医疗防护物资、医疗救治设备、医治床位从严重短缺到基本满足疫情防控需要；千方百计协调解决重点物资生产核心岗位用工，保障粮油与肉禽蛋菜奶等食品的市场供应和价格基本稳定，多措并举确保能源供应安全稳定，有效保障医疗废物、废水安全处置。注重科研攻关和临床救治、防控实践相协同，第一时间研发出核酸检测试剂盒，加快有效药物筛选和疫苗研发，国产疫苗接种正式启动，充分发挥科技对疫情防控的支撑作用。针对境外疫情扩散蔓延，加强输入性风险防控，做好对境外我国公民关心关爱，开辟临时航班有序接回我国在外困难人员。

二是毫不放松抓好常态化疫情防控。适时将全国总体防控策略调整为“外防输入、内防反弹”，推动防控工作由应急性超常规防控向常态化防控转变，健全及时发现、快速处置、精准管控、有效救治的常态化防控机制。充分利用现代信息技术，广泛应用健康码识别，持续提升常态化疫情防控精准性，有效保障企业正常生产和居民正常生活。面对局部点状疫情反弹，坚持分区分级防控，有针对性调整区域疫情风险等级，及时开展流行病学调查和大数据追踪溯源。着力查补薄弱环节，持续提升新冠病毒核酸检测能力，盯紧冷链物流等重点行业加强防控。

三是深入推进疫情防控国际合作。本着公开、透明、负责任的态度，认真履行国际义务，最早向世界通报疫情，第一时间发布新冠病毒基因序列等信息，第一时间公布诊疗方案和防控方案，坚定支持世界卫生组织发挥领导作用。开设疫情防控网上知识中心并向所有国家开放，公开发布8版诊疗方案、7版防控方案，毫无保留同各方分享防控和救治经验。发起新中国成立以来规模最大的全球人道主义行动，向世界卫生组织和联合国全球人道主义应对计划提供支持，为有需要的34个国家派出36支医疗专家组，向150个国家和13个国际组织提供抗疫援助。发挥抗疫物资最大供应国优势，全年向200多个国家提供了超过2200亿只口罩、23亿件防护服、10亿人份检测试剂盒。积极推进药物、疫苗研发合作和国际联防联控，帮助发展中国家克服疫情带来的困难。

（二）围绕市场主体的急需制定和实施宏观政策，经济运行持续稳定恢复。面对历史罕见的冲击，我们在“六稳”工作基础上，明确提出“六保”任务，特别是保就业保民生保市场主体，以保促稳、稳中求进。立足国情实际，既及时果断又保持定力，坚持不搞“大水漫灌”，科学把握规模性政策的平衡点，加大宏观政策应对力度，经济发展的内生动力、平衡性和可持续性进一步增强。

一是主要指标好于预期。2020年，国内生产总值达101.6万亿元，增长2.3%。城镇新增就业1186万人，年末城镇调查失业率为5.2%。居民消费价格指数上涨2.5%。国际收支基本平衡，外汇储备保持在3万亿美元以上。

二是助企纾困政策有效实施。减税降费红利深度释放，实施阶段性大规模减税降费，阶段性减免小规模纳税人增值税，阶段性减免养老、失业、工伤三项社会保险单位缴费部分，减半征收职工医疗保险单位缴费部分，落实住房公积金阶段性支持政策，全年为市场主体减负超过2.6万亿元。创新宏观政策实施方式，中央财政对新增2万亿元资金建立直达机制，省级财政加大资金下沉力度，共同为市县基层落实惠企利民政策及时补充财力。通过降低存款准备金率、中期借贷便利、公开市场操作、再贷款再贴现、创新直达实体经济的货币政策工具等方式，共推出9万多亿元的货币支持措施。通过贷款市场报价利率（LPR）改革推动社会融资成本下降。大型商业银行普惠小微企业贷款增长50%以上，全年金融系统向实体经济让利1.5万亿元。阶段性对部分服务业小微企业和个体工商户减免缓收房屋租金。

三是市场主体预期稳定向好。迅速建立并不断完善全国疫情信息发布机制，实事求是、公开透明发布疫情权威信息，有效保障企业正常生产和居民正常生活。充分发挥宏观政策协调机制和重点省市“六稳”、“六保”会商机制作用，中央与地方之间、部门之间的政策联动协调不断增强，宏观经济治理体系更加完善，政策稳定性、可预期性和透明度进一步提升。解读形势和政策更加及时，回应社会关切更加积极，有效稳定市场主体发展信心。

（三）坚决打好三大攻坚战，主要目标任务如期完成。瞄准突出问题和薄弱环节狠抓政策落实，脱贫攻坚战取得了全面胜利，污染防治力度不断加大，重大风险得到有效防控。

一是脱贫攻坚战取得了全面胜利。实施挂牌督战，项目资金向“三区三州”等深度贫困地区倾斜。针对疫情、汛情对脱贫攻坚带来的不利影响，优先支持贫困劳动力务工就业，多渠道扩大以工代赈实施规模，加大产业扶贫和就业扶贫力度，强化产销对接和科技帮扶，开展消费扶贫行动，及时落实兜底保障等帮扶措施。出台易地扶贫搬迁后续扶持若干政策措施，“十三五”960多万人易地扶贫搬迁建设任务全面完成。着力巩固“三保障”成果，统筹运用基本医保、大病保险和医疗救助等制度保障，有效减轻贫困人口就医费用负担，脱贫攻坚农村危房改造扫尾工程按期完成，全面解决现行标准下的贫困人口饮水安全问题。开展国家脱贫攻坚普查，建立防止返贫监测和帮扶机制。现行标准下9899万农村贫困人口全部脱贫，全国832个贫困县全部摘帽，12.8万个贫困村全部出列，绝对贫困和区域性整体贫困得到解决。

二是污染防治攻坚战圆满完成阶段性目标。扎实推进节能减排，单位国内生产总值能耗和单位国内生产总值二氧化碳排放量继续下降，非化石能源占能源消费总量比重达15.9%。继续打好蓝天、碧水、净土保卫战，持续实施重点区域秋冬季大气污染综合治理，开展夏季臭氧（O）污染防治攻坚，积极稳妥推进北方地区冬季清洁取暖，有序推进钢铁行业超低排放改造，扎实推进柴油货车污染治理，积极推进“公转铁”、“公转水”，全国地级及以上城市空气质量优良天数比率达87%。长江、黄河等大江大河重点流域及渤海等重点海域环境质量加快改善，饮用水水源保护和城市黑臭水体治理力度加大，地表水质量达到或好于Ⅲ类水体比例达83.4%，劣Ⅴ类水体比例降至0.6%。深入实施国家节水行动，万元国内生产总值用水量预计下降1.9%。推动受污染耕地和污染地块安全利用。持续实施农业农村污染治理攻坚，全面开展“无废城市”建设试点，扎实推进污水资源化利用、塑料污染治理、医疗废物处置，推动资源循环利用基地和大宗固体废弃物综合利用基地建设，基本实现固体废物零进口目标。加快构建现代环境治理体系，实现全国固定污染源排污许可全覆盖。扎实推进国家生态文明试验区建设。强化生态保护修复，持续开展大规模国土绿化行动，深入推进三江源、祁连山等重点区域综合治理，森林草原防灭火、湿地保护等重要生态系统保护管理能力有效提升。深入推进生态保护红线监管工作。创建绿色产业示范基地，推广先进绿色技术。积极应对气候变化，提高国家自主贡献目标，参与和引领全球气候治理。开展全国碳排放权交易市场第一个履约周期配额分配。

三是防范化解重大风险取得良好成效。稳妥化解地方政府债务风险，持续推进结构性去杠杆，坚决遏制地方政府隐性债务增长。及时处置一批重大金融风险隐患，各类高风险金融机构得到有序处置，影子银行风险持续收敛，“精准拆弹”有力有效，互联网金融风险有效防控，逐步建立多元化的债券违约处置机制，初步建立系统重要性金融机构、金融控股公司、金融基础设施等统筹监管框架，防范化解重大金融风险攻坚战取得重要阶段性成果，金融市场运行平稳有序。提升重要材料、关键零部件、核心元器件和关键软件的稳定供应水平，着力保持产业链供应链稳定。

（四）深入推进创新驱动发展，科技实力进一步提升。创新在我国现代化建设全局中的核心地位不断增强，全国研究与试验发展经费投入强度为2.4%，科技进步贡献率提高至60%以上。

一是创新能力建设进一步加强。重大科技成果持续涌现，“嫦娥五号”任务首次实现我国地外天体采样返回，我国首次火星探测任务“天问一号”探测器成功发射，500米口径球面射电望远镜（FAST）正式开放运行，“北斗三号”全球卫星导航系统正式开通，量子计算原型系统“九章”成功研制，全海深载人潜水器“奋斗者”号完成万米深潜。国家实验室相继挂牌，新一代人工智能、量子通信与量子计算机、脑科学和类脑研究等“科技创新2030－重大项目”加快部署实施，深度参与热核聚变实验堆计划等国际大科学计划。创新平台建设加速推进，在新一代信息技术、生物医药、新能源等战略性领域高水平建设一批国家产业创新中心、工程研究中心、技术创新中心、制造业创新中心和企业技术中心，高能同步辐射光源、硬X射线自由电子激光、未来网络试验设施等国家重大科技基础设施加快建设。

二是关键核心技术攻关深入推进。实施“揭榜挂帅”等机制，积极探索完善社会主义市场经济条件下关键核心技术攻关新型举国体制，打好关键核心技术攻坚战，加快解决“卡脖子”问题。不断创新支持方式，强化企业创新主体地位，激励企业加大研发投入，支持企业联合科研院所和上下游企业开展技术研发。

三是新产业新业态逆势成长。国家战略性新兴产业集群发展工程深入实施，推动民用空间基础设施加快建设，5G、数据中心、工业互联网等新型基础设施建设稳步推进，集成电路产业有序发展。推动产业数字化智能化改造，推进国家数字经济创新发展试验区建设，开展数字化转型伙伴行动、中小企业数字化赋能专项行动、数字经济新业态培育行动，带动更多中小微企业“上云用数赋智”。传统产业数字化转型持续推进，电商扶贫力度不断加强。

四是重点区域创新高地建设加快推进。北京、上海、粤港澳大湾区国际科技创新中心建设整体格局初步形成，综合性国家科学中心建设成效显著，产业创新高地建设深入推进，国家自主创新示范区和国家高新技术产业开发区加快建设，生产力布局和创新力量布局实现进一步融合。

五是创新创业创造新生态持续构建。全国复制推广第三批20项全面创新改革经验。采取线上线下相结合的方式举办2020年全国双创活动周，布局建设第三批双创示范基地。聚焦创业带动就业，开展社会服务领域创业就业示范。加大创业担保贷款政策实施力度。全年日均净增市场主体4.1万户，其中企业1.3万户。

（五）坚定实施扩大内需战略，强大国内市场加快形成。着力畅通供需循环，深入挖掘和激发国内市场需求潜力，内需对经济增长的拉动力稳步提升。

一是消费基础作用进一步增强。积极支持以新业态新模式引领新型消费加快发展，加快培育建设国际消费中心城市，文化和旅游消费、信息消费试点示范有序推进，养老托育等服务消费扩容提质，电子商务进农村综合示范深入实施。稳定和扩大汽车等大宗消费，提振餐饮消费，农村消费潜力进一步释放。加快废旧家电回收体系建设，推动家电更新消费。全年社会消费品零售总额达39.2万亿元。全国网上零售额达11.8万亿元，增长10.9%，其中实物商品网上零售额增长14.8%，占社会消费品零售总额的24.9%。

二是投资关键作用进一步发挥。出台推动基础设施高质量发展的意见、推动都市圈市域（郊）铁路加快发展的意见，加大“两新一重”领域投资力度，开工建设川藏铁路等一批重大工程。加快下达中央预算内投资计划，及时调整优化结构，进一步集中力量办好国家层面的大事、难事、急事。重点支持公共卫生等疫情暴露的短板弱项和铁路、公路、水运、机场、重大水利、重大科技和能源基础设施、城镇老旧小区改造等领域建设。扩大地方政府专项债券使用范围，支持国家重大战略项目建设。用好向民间资本推介项目长效机制，支持民间投资参与重大工程建设。启动基础设施领域不动产投资信托基金（REITs）试点，盘活基础设施存量资产。深化投资审批制度改革，积极探索投资项目承诺制，多个审批环节统一受理、同步评估、并联审批有序推进。全年固定资产投资（不含农户）增长2.9%，对经济恢复增长发挥了重要作用。

三是现代流通体系加快建设。推动现代物流业高质量发展，新布局建设22个国家物流枢纽。优化发展环境促进生鲜农产品流通，面向特色农产品优势产地、集散地布局建设17个国家骨干冷链物流基地，“通道+枢纽+网络”的现代物流运行体系加快形成。创新物流服务模式，鼓励“互联网+”货运物流新业态健康规范发展，促进物流业制造业深度融合、创新发展。统筹降低流通领域制度性交易成本、技术性成本，积极推动物流降本增效。

（六）持续深化供给侧结构性改革，产业结构调整迈出新步伐。坚持把发展经济着力点放在实体经济上，经济质量效益和核心竞争力不断提高。

一是制造业高质量发展扎实推进。实施增强制造业核心竞争力工程。开展先进制造业集群培育试点示范，发挥好先进制造产业投资基金作用。深化新一代信息技术与制造业融合发展，进一步完善工业互联网平台赋能体系。引导企业开展智能化、绿色化、服务化改造，建成一批高水平智能制造示范工厂和绿色制造示范项目，促进传统产业安全、绿色、集聚、高效发展。持续巩固去产能成果，优化重大生产力布局。坚持“上大压小、增优汰劣”，优化煤炭产能结构，全年淘汰落后产能1亿吨以上。完善钢铁项目产能置换和备案办法，全面完成“十三五”期间压减粗钢产能目标任务，积极推进钢铁企业兼并重组。进一步优化石化产业布局，推动重大石化项目建设和城镇人口密集区危险化学品生产企业搬迁改造。促进稀土等战略性矿产资源产业高质量发展，规范资源开发秩序。推动智能汽车创新发展。成功举办云上2020年中国品牌日系列活动。

二是现代服务业发展迈出坚实步伐。积极构建优质高效、竞争力强的服务产业体系。出台进一步推进服务业改革开放发展的指导意见，组织首批先进制造业和现代服务业融合发展试点，积极推动产业共性技术研发、工业设计、总集成总承包、全生命周期管理、检验检测认证等生产性服务业发展。

三是粮食安全和农副产品市场供应得到有力保障。稳定粮食生产，粮食产量连续6年保持在1.3万亿斤以上，实现谷物基本自给、口粮绝对安全。加强粮食供需形势分析研判，进一步完善粮食储备调控体系。完善粮食“产购储加销”协同保障机制，优化储备品种结构和区域布局，建立政府储备规模动态调整机制，支持建设234个粮食仓储物流和应急项目，不断提升粮食仓储能力、提高粮食流通效率，多环节全链条系统化减少粮食产后损失。及时跟踪研判生猪市场形势变化，加强动物防疫基础设施建设，做好非洲猪瘟常态化防控，生猪生产加快恢复，聚焦重要时段投放中央冻猪肉储备，有效保障了猪肉等重要民生商品的供应。“菜篮子”、“果盘子”等产品数量充足，均衡供应能力明显增强。

四是能源安全保障能力持续提升。积极推进煤电油气产供储销体系建设，进一步增强紧缺矿产资源自主保障能力，推动国内油气增储上产，加强油气储备能力和重大电力工程建设。全国大电网基本实现联通，西电东送能力达到2.6亿千瓦。风电、太阳能、水电装机规模保持世界第一，非化石能源发电装机规模增长到9.8亿千瓦。持续开展电力系统灵活性改造，风电、光伏发电和水能利用率均提高到96%以上。

（七）深入实施乡村振兴战略，农业农村发展势头持续向好。统筹推进“三农”工作，不断优化现代农业产业体系、生产体系、经营体系，农业农村发展水平明显提高。

一是农业供给侧结构性改革进一步深化。实施藏粮于地、藏粮于技战略。农业基础设施条件不断改善，完成8000万亩高标准农田年度建设任务。农业科技支撑不断加强，农作物耕种收综合机械化率达到71%。动植物保护能力提升工程深入实施，农业防灾减灾能力不断增强。区域化布局、规模化经营、标准化生产、社会化服务、品牌化营销成为农业发展的新趋势，有效提高了土地生产率、劳动生产率。积极推进土地托管、代耕代种、生产服务外包等规模经营方式，全国农村承包耕地流转达到5.55亿亩。金融支农投入稳步增加，年末涉农贷款余额38.95万亿元，增长10.7%。

二是农村产业融合发展深入推进。已认定两批共200个国家农村产业融合发展示范园，探索创新融合发展的有效路径。农产品加工流通业加快发展，农业产业链、价值链不断延伸，农产品产加销一体化进程明显加快。农业农村多种功能得到释放，农村电商、休闲观光农业、乡村旅游等新业态蓬勃发展。

三是美丽宜居乡村建设全力推进。深入实施村庄清洁行动，制定完善农村厕所革命、生活污水和生活垃圾治理相关标准规范，农村人居环境整治三年行动方案目标任务顺利完成，农村卫生厕所普及率超过68%，农村生活垃圾进行收运处理的行政村比例超过90%。强化水电路等农村基础设施短板领域和薄弱环节建设，推动农村公共基础设施管护体制改革。完成新一轮农网改造，能源普遍服务水平大幅提升。

（八）加快构建区域协调发展新机制，区域发展格局进一步优化。更加注重发挥区域比较优势，着力促进城乡融合发展，区域优势互补、协调发展的格局持续优化，主体功能区战略稳步推进，“多规合一”的国土空间规划体系正在形成。

一是以人为核心的新型城镇化扎实推进。户籍制度改革深入推进，城区常住人口300万以下城市基本取消落户限制，1亿非户籍人口落户城镇目标顺利实现。城镇化空间格局持续优化，成渝地区双城经济圈建设规划纲要编制实施，关中平原、兰州－西宁等城市群建立协调推进机制，南京、广佛、长株潭等都市圈同城化水平持续提升。县城补短板强弱项工作稳步推进，国家新型城镇化综合试点顺利收官，一批有效经验在全国复制推广。特色小镇逐步走上规范健康发展轨道。国家城乡融合发展试验区全面启动探索试验。

二是区域重大战略深入实施。纵深推进京津冀协同发展，积极稳妥疏解北京非首都功能，高标准高质量推进雄安新区规划建设，加快北京城市副中心建设，持续实施协同发展重大项目，加大京津冀地区城际铁路规划建设力度。扎实推进长江经济带生态环境系统性保护修复，大力实施城镇污水垃圾、化工污染、农业面源污染、船舶污染以及尾矿库治理“4+1”工程，长江流域重点水域“十年禁渔”全面启动，深入开展绿色发展试点示范，长江保护法正式出台。稳步推进粤港澳大湾区建设，加大科技创新和产业协同力度，深入推进重大合作平台建设，实施粤港澳大湾区城际铁路建设规划，基础设施和规则机制互联互通取得新进展，《深圳建设中国特色社会主义先行示范区综合改革试点实施方案（2020－2025年）》出台实施。扎实推进长三角一体化发展，推进重大平台建设和跨区域合作，积极构建协同创新网络体系，持续深化生态环境共保联治，提升基础设施互联互通水平，加快公共服务便利共享。黄河流域生态保护和高质量发展规划纲要印发实施。

三是区域协调发展战略落细落实。持续推进西部大开发形成新格局，支持东北地区全面振兴，促进中部地区加快崛起，出台实施支持湖北省经济社会发展一揽子政策，继续推动东部地区率先发展。有力推进重点领域重点平台建设，支持国家级新区和承接产业转移示范区重点项目建设。革命老区、民族地区、边境地区、生态退化地区、资源型地区、老工业地区振兴发展迈出新步伐。坚持陆海统筹，稳步推进海洋经济发展。

（九）全面深化改革推向纵深，市场主体信心和活力进一步增强。社会主义市场经济体制加快完善，全面深化改革取得新的突破，发展活力和内生动力进一步增强。

一是要素市场化配置体制机制进一步完善。深化土地计划管理方式改革，赋予省级人民政府更多自主权，启动新一轮农村宅基地制度改革试点，调整完善土地出让收入使用范围，优先支持乡村振兴。加快完善技能人才评价制度，民营企业职称评审渠道逐渐打通，区域一体化人才资格互认机制稳步推进。深化科技成果使用权、处置权和收益权改革，开展赋予科研人员职务科技成果所有权或长期使用权试点。开展公共数据资源开发利用试点，推进政务数据有序共享。制定建设高标准市场体系行动方案。

二是国有企业改革深入推进。大力实施国企改革三年行动，剥离办社会职能和解决历史遗留问题主体任务基本完成，国有企业混合所有制改革积极稳妥深化，国有资产监管的系统性针对性有效性显著增强，市场化经营机制建设迈出新步伐。深化重点行业改革，推动电网企业剥离装备制造等竞争性业务。国家石油天然气管网集团有限公司完成油气管网资产、人员、业务交接，实现并网运行，推动油气管网向社会资本、市场主体公平开放。

三是民营企业发展环境持续改善。在放宽市场准入、加强金融支持、营造公平竞争环境、保护企业和企业家合法权益等方面出台一系列政策举措。民营企业进入油气勘探开采的准入限制逐步放开，支持民营企业参与交通基础设施建设发展。商业银行对民营企业“敢贷、愿贷、能贷”的长效机制加快建立，清理拖欠民营企业中小企业账款行动取得重要成果，妨碍统一市场和公平竞争的政策措施清理工作深入推进，涉政府产权纠纷问题专项治理取得积极成效。

四是营商环境不断优化。“放管服”改革深入推进，全面实施优化营商环境条例，在部分城市开展营商环境评价，《中国营商环境报告2020》正式发布。深入开展工程建设项目审批制度改革，基本建成全国统一的审批和管理体系。招标投标和政府采购领域营商环境持续优化，公共资源全流程电子化交易全面推行。修订印发《市场准入负面清单（2020年版）》，清单事项由131项压减至123项，市场准入限制持续放宽。

五是财税、金融、价格等重点领域改革步伐加快。生态环境、公共文化、自然资源、应急救援等领域中央与地方财政事权和支出责任划分改革方案印发实施。公开发行企业债券、公司债券全面实施注册制，资本市场韧性增强并延续总体平稳态势，金融业的适应性、竞争力和普惠性稳步提升。修订《中央定价目录》，定价项目缩减近30%，电力、油气、公用事业、农产品等重点领域价格改革不断深化。服务业改革开放发展力度进一步加大，电力、油气体制改革持续深化。

（十）持续拓展对外开放范围、领域和层次，开放型经济新体制加快构建。有力克服新冠肺炎疫情和外部环境诸多不确定性，坚持实施更大范围、更宽领域、更深层次对外开放。

一是稳外贸稳外资力度加大。全年货物进出口额达32.2万亿元，吸引外资1444亿美元。扩大出口信用保险覆盖范围，增加出口信贷投放，完善出口退税政策，稳定加工贸易，支持边境贸易创新发展。贸易新业态新模式加快发展，增设46个跨境电子商务综合试验区，跨境电商零售进口试点扩大至86个城市和海南全岛，新增17个市场采购贸易方式试点，积极探索保税维修、离岸贸易等新业务。第三届中国国际进口博览会、中国国际服务贸易交易会成功举办，在网上举办第127、128届广交会。共同落实中美第一阶段经贸协议工作稳步推进。全国和自贸试验区外资准入负面清单分别缩减至33条和30条，进一步放宽服务业、制造业、农业领域外资准入。出台2020年版鼓励外商投资产业目录，扩大鼓励外商投资范围。制定海南自由贸易港外资准入负面清单，海南投资自由化便利化水平进一步提高。北京新一轮服务业扩大开放综合试点继续深化。

二是共建“一带一路”稳步推进。统筹推进疫情防控和“一带一路”境外项目建设，境外投资保持总体平稳。与非洲联盟签署共建“一带一路”合作规划。国际产能合作和第三方市场合作不断深化。中巴经济走廊成功统筹防疫与生产，成为“一带一路”重大项目“两手抓”、“两不误”的标杆。缅甸皎漂经济特区、中阿（联酋）产能合作示范园等取得积极进展，雅万高铁和匈塞、中老、中泰铁路等互联互通项目扎实推进。健康丝绸之路、绿色丝绸之路、数字丝绸之路建设深入推进。中欧班列保持安全稳定畅通运行，开行数量超1.2万列，逆势增长50%，综合重箱率达98.4%。

三是对外开放高地建设取得新成效。海南自由贸易港建设总体方案出台实施，政策制度框架加快建立。增设北京等3个自贸试验区，推动浙江自贸试验区扩区。向全国复制推广自贸试验区37项制度创新成果，累计复制推广260项。新设12个综合保税区，将7个其他形式的海关特殊监管区整合优化为综合保税区。设立广西百色、新疆塔城重点开发开放试验区。

四是参与全球经济治理迈出新步伐。在联合国、二十国集团、亚太经合组织、金砖国家等重要多边平台积极提出中国主张和中国方案。推动区域全面经济伙伴关系协定、中柬自贸协定、中欧地理标志协定等正式签署，中国－毛里求斯自贸协定按时生效实施。中欧投资协定谈判如期完成，中国－挪威、中国－摩尔多瓦自贸协定谈判加快推进。提出《全球数据安全倡议》，推动数字经济国际合作与交流。积极发展全球伙伴关系，推进大国协调合作，深化同周边国家关系，加强同发展中国家团结合作。

（十一）强化民生兜底，人民群众基本生活得到切实保障。针对疫情带来的民生问题，通过加大投入、落实政策、织密扎牢社会保障网，不断提升人民群众获得感幸福感安全感。全国居民人均可支配收入实际增长2.1%。

一是就业优先政策落细落实。就业是最大的民生，保市场主体也是为稳就业保民生。加大减负、稳岗、扩就业支持力度，帮扶受疫情影响的重点行业、中小微企业和个体工商户等市场主体纾困，扩大有效投资增加就业。多渠道做好重点群体就业工作，支持大众创业万众创新带动就业，在家政服务、养老托育、乡村旅游、家电回收等社会服务领域开展双创带动就业示范行动，推进返乡入乡创业高质量发展。支持多渠道灵活就业，扩大个体经营、国有企事业单位招聘、基层项目招聘、升学入伍、就业见习等吸纳就业规模，促进高校毕业生市场化社会化就业。统筹做好退役军人、农民工等重点群体就业工作。支持建设53个区域性公共实训基地，提升重点群体就业技能。

二是健康中国建设扎实推进。健康中国行动启动实施，深入开展爱国卫生运动，综合防控儿童青少年近视，积极推进“一老一小”健康服务，居民健康素养水平明显提升。区域医疗中心建设启动，医联体建设和县域综合医改稳步推进，84%的县级医院达到二级及以上医院水平。每千人口医疗卫生机构床位数预计6.5张。现代医院管理制度建设持续推进，公立医院耗材加成全面取消。医教协同不断深化，医师区域注册制度建立完善，每千人口执业（助理）医师数预计2.9人，每万人口全科医生数预计2.83人。药品、医用耗材集中采购和使用改革协同推进，短缺药品保供稳价扎实推动，基本药物数量由520种增加到685种。

三是社会保障体系进一步完善。参加城镇职工基本养老保险和城乡居民基本养老保险人数达9.99亿人。企业职工基本养老保险基金中央调剂比例从3.5%提高到4.0%，实现省级统收统支，退休人员基本养老金稳步提高。职工基本医疗保险、城乡居民基本医疗保险和大病保险制度更趋完善，医保扶贫成效显著，医保药品目录动态调整有序推进。通过工伤保险为185万工伤职工及供养亲属提供待遇保障。失业保险保障范围进一步扩大，阶段性实施失业补助资金政策、阶段性扩大失业农民工保障范围，全年共有1337万人领取到不同项目的失业保险。全年向608万户企业发放失业保险稳岗返还1042亿元，惠及职工1.56亿人。加强困难群众兜底保障，适度扩大低保覆盖范围，做到“应保尽保”；出台救助“扩围”政策，及时启动社会救助和保障标准与物价上涨挂钩联动机制，对因疫致困、未参保失业人员加大救助帮扶，实现“应救尽救”，因疫情新纳入低保、特困供养近600万人，实施临时救助超过800万人次。城镇老旧小区改造全面推进，已开工改造城镇老旧小区4.03万个，涉及居民736万户。保障性安居工程建设持续推进，棚户区改造新开工209万套；大中城市公租房继续发展，城镇困难群众住房保障不断加强。有力有序做好防汛抢险救援工作。

四是公共服务补短板强弱项提质量深入推进。推动出台国家基本公共服务标准。学前教育毛入学率、九年义务教育巩固率、高中阶段教育毛入学率分别达到85.2%、95.2%、91.2%，普通高等教育本专科招生967.5万人，研究生招生110.7万人。基本实现每个县办好1－2所县级公立医院，每个乡镇有1所乡镇卫生院，每个行政村有1所村卫生室。长城、大运河、长征等国家文化公园标志性项目建设统筹推进。预计人均体育场地面积2.2平方米，增长4.8%。城乡社区综合服务设施覆盖率分别达到96.4%和83.7%，每百户居民拥有城乡社区综合服务设施达34.7平方米。继续实施残疾人两项补贴制度，惠及1153万困难残疾人和1433万重度残疾人，为残疾人提供服务设施数预计4403个。家政服务提质扩容深入推进。修订未成年人保护法，妇女儿童权益保障工作不断加强。支持特困人员供养服务设施建设。促进养老托育服务健康发展的意见、建立健全养老服务综合监管制度促进养老服务高质量发展的意见出台，普惠养老专项行动继续实施，各类养老床位数达到823.8万张。普惠托育服务设施建设持续推进。

综合来看，2020年经济实现正增长，就业物价预期目标较好完成，国际收支保持基本平衡，创新驱动、资源节约、环境保护、民生保障等领域指标继续改善，全年经济社会发展主要目标任务较好完成。

经过五年持续奋斗，“十三五”规划目标任务胜利完成，经济发展方式实现重大转型，经济总量越过100万亿元大关，居民收入基本同步增长。人均国内生产总值超过1万美元，165项重大工程项目基本完成，国家发展物质基础更加雄厚。覆盖城乡居民的社会保障体系基本建成，教育公平和质量较大提升，生态文明建设取得重大进展。我国经济实力、科技实力、综合国力和人民生活水平又跃上新的大台阶，全面建成小康社会取得伟大历史性成就，中华民族伟大复兴向前迈出了新的一大步。这是以习近平同志为核心的党中央坚强领导的结果，是习近平新时代中国特色社会主义思想科学指引的结果，是各地区各部门贯彻落实党中央决策部署、勇于担当、善于作为的结果，是广大人民群众团结奋进、开拓进取的结果。

同时也要看到，2021年国内外环境面临深刻复杂变化。世界百年未有之大变局进入加速演变期，新冠肺炎疫情又增添了新的变数；长期存在的结构性矛盾依然凸显，在外部冲击下又出现了一些新问题。一是疫情走势不确定性对经济进一步恢复构成掣肘。新冠肺炎疫情仍在全球蔓延，疫情反弹和长期持续的风险客观存在，国际上推动复工复产和防止疫情扩散面临“两难”。国内疫情防控仍有薄弱环节，我国外防输入和内防反弹的压力始终存在。二是外部环境复杂严峻可能影响我国内经济平稳运行。世界经济有望出现恢复性增长，但复苏不稳定不平衡，主要经济体量化宽松等宏观政策造成外溢效应，全球产业链供应链区域化、本地化特征更趋明显，单边主义、保护主义持续演化，影响我国经济稳定恢复的外部变数依然较多。三是经济恢复基础尚不牢固。国内外市场有效需求仍显疲弱，居民消费仍受制约，投资增长后劲不足，出口持续回稳基础不牢。中小微企业和个体工商户困难较多。不少行业企业还处在疫后恢复期，生产经营还面临不少压力，影响供需良性循环。四是经济动能接续转换面临不少障碍。推动经济转型升级和创新发展仍受到一些制约，保持产业链供应链稳定运行面临挑战，“卡脖子”问题依然突出，传统行业转型升级面临挑战，国内统一大市场仍需完善，要素资源配置、生产力布局等仍需优化。五是重点领域安全风险不容忽视。稳就业压力仍然较大，一些地方财政收支矛盾突出，基层保基本民生、保工资、保运转支出和地方政府债务还本付息压力较大，防范化解金融等领域风险任务依然艰巨，企业债务违约压力加大，保障粮食能源安全面临新的挑战，生态环境质量改善成效并不稳固，教育、医疗、养老、托育等民生领域还有不少薄弱环节。同时，我们在工作中也还存在一些不足，比如，推动经济高质量发展、构建新发展格局的能力和水平还需进一步提升，政策间的协调配合还有待加强，有的政策实施效果还有待提高。

总的看，虽然挑战前所未有，更具有复杂性、全局性，但机遇也前所未有，更具有战略性、可塑性，机遇大于挑战，我国有中国共产党领导和中国特色社会主义制度的显著优势，经济长期向好的趋势没有改变，我国发展仍处于重要战略机遇期。我们有信心、有底气、有能力危中寻机、化危为机，不断开创我国经济社会发展的新局面。

二、2021年经济社会发展总体要求、主要目标和政策取向

2021年是我国现代化建设进程中具有特殊重要性的一年，“十四五”开局，全面建设社会主义现代化国家新征程开启，做好经济工作意义重大。

（一）总体要求。

做好2021年经济社会发展工作，要在以习近平同志为核心的党中央坚强领导下，以习近平新时代中国特色社会主义思想为指导，全面贯彻党的十九大和十九届二中、三中、四中、五中全会精神，坚持稳中求进工作总基调，立足新发展阶段，贯彻新发展理念，构建新发展格局，以推动高质量发展为主题，以深化供给侧结构性改革为主线，以改革创新为根本动力，以满足人民日益增长的美好生活需要为根本目的，坚持系统观念，巩固拓展疫情防控和经济社会发展成果，更好统筹发展和安全，扎实做好“六稳”工作、全面落实“六保”任务，科学精准实施宏观政策，努力保持经济运行在合理区间，坚持扩大内需战略，强化科技战略支撑，扩大高水平对外开放，保持社会和谐稳定，确保“十四五”开好局起好步，以优异成绩庆祝中国共产党成立100周年。

在具体工作中，要把握好五个重点方面。一是坚持稳中求进。保持战略定力，保持经济运行在合理区间，为保障民生、增强人民群众获得感幸福感安全感提供支撑，为“十四五”开好局创造良好条件。二是坚持做好新冠肺炎疫情防控工作。确保不出现规模性输入和反弹，适应在常态化疫情防控中谋划经济社会发展，巩固拓展疫情防控成果。三是坚持新发展理念构建新发展格局。以编制实施“十四五”规划纲要为抓手，把新发展理念贯穿发展全过程和各领域。把实施扩大内需战略同深化供给侧结构性改革有机结合起来，强化科技自立自强，坚定推进结构调整和产业优化升级，坚持推动经济社会发展全面绿色转型，加快构建以国内大循环为主体、国内国际双循环相互促进的新发展格局。四是坚持深化改革开放。大力破除体制机制障碍，坚定实施高水平对外开放。五是坚持系统观念。统筹国内国际两个大局、办好发展安全两件大事，注重防范化解重大风险挑战，实现发展质量、结构、规模、速度、效益、安全相统一。

（二）主要预期目标。

按照上述总体要求，坚持立足当前、着眼长远，尽力而为、量力而行，在综合平衡基础上兼顾需要与可能，提出2021年经济社会发展主要预期目标：

--国内生产总值增长6%以上。主要考虑：一是按照“十四五”时期经济发展的目标要求，并考虑到2020年基数较低的影响，坚持底线思维，稳定市场预期，有利于实现平稳可持续增长。二是考虑到2021年是“十四五”开局之年，为应对疫情冲击采取的各项政策措施逐步回归常态，将经济增速设为6%以上，有利于引导各方面集中精力推进改革创新、推动高质量发展。三是考虑了经济运行恢复情况，随着经济运行逐步恢复常态、保持在合理区间，实现这一目标有条件有支撑。

--城镇新增就业1100万人以上，城镇调查失业率5.5%左右。关于城镇新增就业总量：随着经济规模扩大、服务业发展，经济增长对就业的吸纳能力不断增强，特别是优化营商环境、支持灵活就业等政策落地见效，实现1100万人以上的城镇新增就业有较好支撑。关于城镇调查失业率：比2020年预期目标下调0.5个百分点，主要是更好体现稳就业的决心以及实施就业优先、强化就业保障的政策导向。同时，在经济运行持续恢复的条件下，随着稳就业工作的深入开展，2021年城镇调查失业率可以控制在5.5%左右。从这两年的实践看，城镇调查失业率逐步运行成熟，能够准确反映就业形势，实现与城镇登记失业率的平稳过渡，2021年不再把城镇登记失业率作为国家宏观调控目标，地方是否保留可根据实际情况自主确定。

--居民消费价格涨幅3%左右。主要考虑：综合翘尾因素和新涨价因素，预计2021年居民消费价格上涨压力总体小于2020年；同时，考虑到国际粮食和能源等大宗商品价格存在上涨可能性，从稳定市场预期等角度出发，将居民消费价格涨幅设为3%左右。

--居民收入稳步增长。主要考虑：这是逐步实现共同富裕的必然要求，有利于更好满足人民日益增长的美好生活需要，更好体现以人民为中心的发展思想。随着企业经营效益逐步改善、促进低收入群体增收、再分配调节不断加力等政策措施持续推进，2021年居民收入增长具有较好支撑。

--进出口量稳质升，国际收支基本平衡。主要考虑：促进外贸进出口量稳质升、保持国际收支基本平衡是应对错综复杂国际形势变化的需要，是推动构建新发展格局、实现高水平对外开放的需要。我国产业链供应链稳步恢复，出口产品综合竞争优势不断增强，跨境电商等新业态蓬勃发展，这都为外贸量稳质升提供了有力支撑。

--单位国内生产总值能耗降低3%左右，主要污染物排放量继续下降。主要考虑：为确保实现2030年前二氧化碳排放达到峰值，结合需要与可能，并与“十四五”规划目标相衔接，将2021年能耗强度目标设为下降3%左右。

--粮食产量保持在1.3万亿斤以上。主要考虑：粮食安全事关国计民生，事关发展全局。为稳定市场预期，夯实粮食安全基础，统筹考虑国内粮食消费需求、综合生产能力、全球粮食市场变化和粮食自给率目标等因素，2021年新增粮食产量指标，将预期目标设为保持在1.3万亿斤以上，同时保持粮食播种面积稳定并努力提高单产。

（三）主要宏观政策取向和重点。

为实现上述目标，要保持宏观政策连续性稳定性可持续性，促进经济运行在合理区间。在区间调控基础上加强定向调控、相机调控、精准调控。宏观政策要继续为市场主体纾困，保持必要支持力度，不急转弯，根据形势变化适时调整完善，进一步巩固经济基本盘，继续实施积极的财政政策、稳健的货币政策和就业优先政策，用好改革政策，根据实际情况不断完善宏观政策组合，把握好政策时度效，以高质量发展为“十四五”开好局。

--积极的财政政策要提质增效、更可持续。考虑到疫情得到有效控制和经济逐步恢复，今年赤字率拟按3.2%左右安排、比去年有所下调，不再发行抗疫特别国债。因财政收入恢复性增长，财政支出总规模比去年增加，重点仍是加大对保就业保民生保市场主体的支持力度。中央本级支出继续安排负增长，进一步大幅压减非急需非刚性支出，对地方一般性转移支付增长7.8%、增幅明显高于去年，其中均衡性转移支付、县级基本财力保障机制奖补资金等增幅均超过10%。建立常态化财政资金直达机制并扩大范围，将2.8万亿元中央财政资金纳入直达机制，为基层提供更加及时有力的财力支持。要节用为民、坚持过紧日子，确保基本民生支出只增不减，助力市场主体焕发生机。优化和落实减税政策，继续执行制度性减税政策，延长小规模纳税人增值税优惠等部分阶段性政策执行期限，实施新的结构性减税举措，对冲部分政策调整带来的影响。将小规模纳税人增值税起征点从月销售额10万元提高到15万元。对小微企业和个体工商户年应纳税所得额不到100万元的部分，在现行优惠政策基础上，再减半征收所得税。

--稳健的货币政策要灵活精准、合理适度。把服务实体经济放到更加突出的位置，处理好恢复经济与防范风险的关系。货币供应量和社会融资规模增速与名义经济增速基本匹配，保持流动性合理充裕，保持宏观杠杆率基本稳定。保持人民币汇率在合理均衡水平上的基本稳定。进一步解决小微企业融资难题。延续普惠小微企业贷款延期还本付息政策，加大再贷款再贴现支持普惠金融力度。延长小微企业融资担保降费奖补政策，完善贷款风险分担补偿机制。加快信用信息共享步伐。完善金融机构考核、评价和尽职免责制度。引导银行扩大信用贷款、持续增加首贷户，推广随借随还贷款，使资金更多流向科技创新、绿色发展，更多流向小微企业、个体工商户、新型农业经营主体，对受疫情持续影响行业企业给予定向支持。大型商业银行普惠小微企业贷款增长30%以上。创新供应链金融服务模式。适当降低小微企业支付手续费。优化存款利率监管，推动实际贷款利率进一步降低，继续引导金融系统向实体经济让利。今年务必做到小微企业融资更便利、综合融资成本稳中有降。

--就业优先政策要继续强化、聚力增效。着力稳定现有岗位，对不裁员少裁员的企业，继续给予必要的财税、金融等政策支持。继续降低失业和工伤保险费率，扩大失业保险返还等阶段性稳岗政策惠及范围，延长以工代训政策实施期限。拓宽市场化就业渠道，促进创业带动就业。推动降低就业门槛，动态优化国家职业资格目录，降低或取消部分准入类职业资格考试工作年限要求。支持和规范发展新就业形态，加快推进职业伤害保障试点。继续对灵活就业人员给予社保补贴，放开在就业地参保的户籍限制。做好高校毕业生、退役军人、农民工等重点群体就业工作，完善残疾人、零就业家庭成员等困难人员就业帮扶政策，促进失业人员再就业。拓宽职业技能培训资金使用范围，开展大规模、多层次职业技能培训，完成职业技能提升和高职扩招“三年行动”目标，建设一批高技能人才培训基地。实施提升就业服务质量工程。运用就业补助资金等，支持各类劳动力市场、人才市场、零工市场建设，广开就业门路，为有意愿有能力的人创造更多公平就业机会。

同时，进一步加强宏观政策协同配合，促进财政、货币、就业、产业、投资、消费、环保、区域、改革等政策形成系统集成效应，着力发挥有效投资的关键作用，强化预期管理，增强市场信心，汇聚起集中力量办大事、难事、急事的合力。改革政策要着力激发市场活力，聚焦实现有效市场和有为政府更好结合，加快推进有利于提高资源配置效率、有利于调动各方积极性、有利于提高发展质量和效益的改革，坚持问题导向，围绕增强创新能力、推动平衡发展、改善生态环境、提高开放水平、促进共享发展等重点领域和关键环节，继续把改革推向深入。

三、2021年国民经济和社会发展计划的主要任务

2021年，要全面贯彻党中央确定的“十四五”时期经济社会发展指导方针和决策部署，深入落实中央经济工作会议精神和《政府工作报告》任务要求，准确把握新发展阶段，深入贯彻新发展理念，加快构建新发展格局，统筹发展和安全，着力做好以下方面工作。

（一）抓紧抓实“外防输入、内防反弹”，科学有效做好常态化疫情防控。坚持常态化精准防控与局部应急处置有机结合，提高应对突发情况的能力。

一是持续优化常态化疫情防控举措。将“外防输入”作为重中之重，严格实施远端防控措施，健全口岸公共卫生体系，对入境人员境外核酸检测全覆盖。加强冷链食品等进口商品预防性全面消毒，加大对重点场所人员、环境、物品等检测排查力度，完善全国进口冷链食品追溯管理平台和省级平台。加强核酸检测能力建设，保障常态化疫情防控需求。科学推进疫苗研制生产、接种使用工作，确保安全有效。落实“四早”要求，进一步改进救治工作。及时主动向社会发布疫情信息，确保公开透明。

二是加快健全国家公共卫生应急管理体系。落实健全公共卫生应急物资保障体系实施方案、公共卫生防控救治能力建设方案，加强防控物资和技术储备。加强公共卫生设施建设，健全突发公共卫生事件应对预案体系。改革完善疾病预防控制体系运行机制，完善传染病疫情和突发公共卫生事件监测系统。优化医疗资源和救治力量布局，构建分层次、分区域的重大疫情医疗救治体系，着力提高重大突发公共卫生事件应对能力。支持医疗废物处置设施建设。

三是积极推进疫情国际联防联控。支持世界卫生组织协调整合资源，公平有效分配疫苗。继续向应对疫情能力薄弱的国家和地区提供帮助，发挥全球抗疫物资最大供应国作用，推动构建人类卫生健康共同体。

（二）加快科技自立自强，推进产业基础高级化、产业链现代化。强化国家战略科技力量，继续加强基础研究、应用研究和科技成果转移转化，进一步提高我国产业核心竞争力。全社会研发经费投入增长7%以上。

一是大力提升科技创新能力。加快推动国家实验室建设和国家重点实验室体系重组，抓紧制定实施基础研究十年行动方案，重点布局一批基础学科研究中心。加强关键核心技术攻关，深入谋划推进“科技创新2030－重大项目”，改革科技重大专项实施方式，推广“揭榜挂帅”等机制。制定实施国家中长期科学和技术发展规划（2021－2035年）。组织实施新型基础设施建设，加快推进人工智能、量子信息、脑科学、生物育种等科技创新。加快北京、上海、粤港澳大湾区国际科技创新中心建设，大力推进怀柔、张江、合肥、粤港澳大湾区综合性国家科学中心建设，高质量谋划推进成渝地区具有全国影响力的科技创新中心建设，增强国家自主创新示范区、高新技术产业开发区、经济技术开发区等创新发展能力。优化国家产业创新中心、技术创新中心、制造业创新中心、工程研究中心、企业技术中心布局。组织实施融通创新示范工程，鼓励大企业向中小企业开放资源、开放场景、开放应用、开放创新需求，打造基于产业链供应链的创新创业生态。用税收优惠机制激励企业加大研发投入，延续执行企业研发费用加计扣除75%政策，将制造业企业加计扣除比例进一步提高到100%。持续推进大众创业万众创新，支持双创示范基地建设，办好全国双创活动周、双创主题日。倡导敬业、精益、专注、宽容失败的创新创业文化，完善试错容错纠错机制。深入推进科技体制改革，加强项目、基地、人才、资金一体化配置，强化国家科普能力建设，完善科技人才激励机制和科技评价机制，支持青年人才加快成为科研主力军。深入推进全面创新改革。健全基础研究稳定支持机制，大幅增加投入，中央本级基础研究支出增长10.6%。完善金融支持创新体系，引导更多资金进入基础研究、自主研发、成果转化等领域。

二是着力振兴和发展实体经济。实施新一轮制造业核心竞争力提升五年行动计划，开展先进制造业集群发展专项行动，创建国家制造业高质量发展试验区。对先进制造业企业按月全额退还增值税增量留抵税额，提高制造业贷款比重，扩大制造业设备更新和技术改造投资。加快重点行业结构调整和绿色化改造，持续推进国家战略性新兴产业集群发展工程，强化技术创新和公共服务。落实新时期促进集成电路产业和软件产业高质量发展的若干政策。加强产业管理，促进新能源汽车健康有序发展，加快构建智能汽车基础设施、产业生态等支撑体系。加快壮大新能源等产业，提升新能源消纳和存储能力。培育壮大生物经济，推动生物技术融合发展。加快临床急需用药和高端医疗装备开发及产业化。推动北斗大规模应用及产业化发展。稳步提升航空产业链现代化和国际化水平。继续推动先进制造业和现代服务业融合试点。依托工业互联网、工业软件等新一代信息技术，加快制造服务业发展。进一步完善废旧家电回收处理体系，畅通家电生产、消费、回收、处理全链条。实施中华老字号保护发展工程。办好2021年中国品牌日活动。

三是加力推动数字化发展。发展数字经济，推进数字产业化和产业数字化，打造具有国际竞争力的数字产业集群。培育数据要素市场，发挥好数据要素关键作用。推动数字政府建设，持续推进实施国家政务信息化重大工程，加快打造数字化一流营商环境。深入推进全国一体化大数据中心体系建设，做好数据中心建设布局，促进数据资源有序流通和创新应用。积极参与数字领域国际规则和标准制定。

四是增强产业链供应链自主可控能力。推进产学研用深度融合，支持企业牵头组建创新联合体，承担国家重大科技项目。统筹推进补齐短板和锻造长板，开展制造业强链补链行动，针对产业薄弱环节，实施好关键核心技术攻关工程，尽快解决一批“卡脖子”问题。实施好产业基础再造工程，提升关键基础材料、基础零部件、先进基础工艺、产业技术基础、工业基础软件等方面自主创新能力。实施产业链合作伙伴提升计划。加强顶层设计、应用牵引、整机带动，强化共性技术供给，深入实施质量提升行动。加强国际产业安全合作。推动搭建国际产业链供应链合作平台，促进全球产业链稳定性和多元化。

（三）坚定实施扩大内需战略，加快形成强大国内市场。紧紧扭住供给侧结构性改革这条主线，注重需求侧管理，形成需求牵引供给、供给创造需求的更高水平动态平衡。

一是激发国内消费潜力。健全工资合理增长机制，提高居民消费能力。有序取消一些行政性限制消费购买的规定，推动汽车等消费品由购买管理向使用管理转变，取消对二手车交易不合理限制，扩大新能源汽车消费占比，增加停车场、充电桩、换电站等设施，加快建设动力电池回收利用体系，鼓励开展汽车下乡和汽车、家电、家具以旧换新，促进家装消费。发展健康、文化、旅游、体育等服务消费，推动互联网诊疗、在线教学、体外诊断、远程办公、通用航空、智能体育等消费新业态新模式加快发展，促进线上线下消费融合发展。引导平台企业合理降低商户服务费。充分挖掘县乡消费潜力，大力提升电商快递进农村综合水平和农产品流通现代化水平，着力畅通工业品下乡、农产品进城双向流通渠道，补齐农产品冷链物流设施短板，开展农商互联农产品供应链建设，推动农村产品和服务品牌化、标准化、数字化、产业化改造，吸引城市居民下乡消费。完善节能环保产品推广机制，提高绿色产品市场占有率。培育国际消费中心城市，建设一批区域消费中心。高质量推进步行街改造提升。以信用体系建设为抓手，强化消费者权益保护，营造良好消费环境。

二是增强投资增长后劲。坚持“项目跟着规划走，资金要素跟着项目走”，加快国家重大战略项目实施步伐，加强重大项目资金、用地等要素保障。发挥中央预算内投资在外溢性强、社会效益高领域的引导和撬动作用，安排中央预算内投资6100亿元，比上年增加100亿元。新增地方政府专项债券规模为3.65万亿元，优先支持在建工程，合理扩大使用范围。激发全社会投资活力，形成市场主导的投资内生增长机制。协同推进投资审批制度改革、开展投资领域专项执法检查，着力深化在线平台建设应用。加大新型基础设施投资力度，谋划布局5G、数据中心、基础软件、空间基础设施等建设。支持城市防洪排涝设施建设，加强城市内涝治理。稳步推进川藏铁路、西部陆海新通道等重大项目建设，完善机场布局，加快推进三峡枢纽水运新通道建设前期工作，实施国家水网、航道网工程，有序推进油气和电力等重大能源工程。稳妥推进基础设施领域不动产投资信托基金试点，推动盘活存量资产，形成投资良性循环。规范推广政府和社会资本合作模式，吸引民间资本参与。

三是加快构建现代流通体系。加快推进流通体系软硬件建设，优化提升流通基础设施网络，发展流通新技术新业态新模式，完善流通领域规范和标准，推动形成全国统一大市场。持续加强国家物流枢纽、国家骨干冷链物流基地等重大物流基础设施建设，开展商贸物流高质量发展专项行动，建设现代物流体系。巩固物流减税降费成果，完善铁路、水运干线物流通道，优化货物运输结构，积极发展多式联运、智慧物流，加快推进“快递进村”工程。培育壮大现代流通企业，加强国际航空货运能力建设，拓展全球流通服务网络，构建安全可靠的现代国际物流供应链体系，建设覆盖线上线下的重要产品追溯体系。

（四）深化重点领域和关键环节改革，推动有效市场和有为政府更好结合。紧盯解决突出问题，提高改革的战略性、前瞻性、针对性，使改革更好对接发展所需、基层所盼、民心所向。

一是加快建设高标准市场体系。实施建设高标准市场体系行动方案。修订印发《市场准入负面清单（2021年版）》，巩固维护“全国一张清单”管理模式，开展市场准入效能评估，加快推进放宽市场准入试点，制定出台海南、深圳等放宽市场准入特别措施。完善竞争政策框架，健全公平竞争审查机制，加强反垄断和反不正当竞争执法，防止资本无序扩张，着力营造一视同仁、公平竞争的公正市场环境。开展要素市场化配置综合改革试点，推进要素市场制度建设。开展招标投标领域改革创新试点，加大地方招标投标制度规则清理整合力度。深化土地管理制度改革，积极探索实施农村集体经营性建设用地入市制度，推动不同产业用地类型合理转换，建立健全省级人民政府用地审批工作评价机制。畅通劳动力和人才社会性流动渠道，深化人才评价、职业资格职称评定等人事人才管理制度改革。健全多层次资本市场体系。强化产权和知识产权保护，健全职务科技成果产权制度，深化科技成果使用权、处置权、收益权改革，持续做好赋予科技人员职务科技成果所有权或长期使用权试点工作，设立知识产权和科技成果产权交易机构。制定出台加快培育数据要素市场的意见，研究制定《网络数据处理安全规范》等国家标准，推动出台《数据安全法》和《个人信息保护法》。

二是进一步转变政府职能。持续优化市场化法治化国际化营商环境。将行政许可事项全部纳入清单管理。深化“证照分离”改革，大力推进涉企审批减条件、减环节、减材料、减时限。完善中小微企业简易注销制度。实施工业产品准入制度改革，推进汽车、电子电器等行业生产准入和流通管理全流程改革。推动工程建设项目全流程审批制度化，进一步精减规范工程建设项目审批事项和条件。深化行业协会、商会改革。完善以信用为基础的新型监管机制，进一步健全守信联合激励和失信联合惩戒机制。建立公共信用信息同金融信息共享整合机制，完善全国“信易贷”平台，扩大信用贷款规模，全面推行信用承诺制度，提升社会信用体系建设法治化水平。推进政务服务标准化、规范化、便利化，建立健全政务数据共享协调机制，推动电子证照扩大应用领域和全国互通互认，实现更多政务服务事项网上办、掌上办、一次办。压缩企业开办时间，支持省级层面统筹开展住所与经营场所分离登记试点。全面实现公共资源全流程电子化交易。进一步完善中国营商环境评价机制，开展营商环境评价和动态监测，编制发布中国营商环境报告（2021年版）。

三是用改革办法降低企业生产经营成本。推进能源、交通、电信等基础性行业改革，提高服务效率，降低收费水平。允许所有制造业企业参与电力市场化交易，进一步清理用电不合理加价，继续推动降低一般工商业电价。中小企业宽带和专线平均资费再降10%。推行高速公路货车分时段分路段差异化收费。取消港口建设费，将民航发展基金航空公司征收标准再降低20%。鼓励受疫情影响较大的地方，对承租国有房屋的服务业小微企业和个体工商户减免租金。推动各类中介机构公开服务条件、流程、时限和收费标准。严控非税收入不合理增长，严厉整治乱收费、乱罚款、乱摊派。

四是着力激发市场主体活力。深入实施国企改革三年行动，加快推进国有经济布局优化和结构调整，积极稳妥深化国有企业混合所有制改革，健全管资本为主的国有资产监管体制。构建亲清政商关系，依法平等保护民营企业产权和企业家权益，弘扬企业家精神，加快建设世界一流企业，实施中小企业创新能力和专业化水平提升工程，培育一批“专精特新”中小企业。加强小微企业普惠金融服务，落实保障中小企业款项支付条例，切实维护中小微企业合法权益。推进能源、铁路、电信等行业竞争性环节市场化改革，深入推进自然垄断业务监管体制改革，加快构建全国统一电力市场，开展铁路市场化改革综合试点。

五是深化财税、金融、价格等体制改革。完善预算管理制度，强化预算约束和绩效管理，加大预算公开力度，精简享受税费优惠政策的办理流程和手续。落实中央与地方政府事权和支出责任划分改革方案。加快推进后移消费税征收环节并稳步下划地方。建设现代中央银行制度，完善货币供应调控机制，稳妥推进数字货币研发。健全金融机构公司治理，深化国有商业银行改革，支持中小银行和农村信用社持续健康发展，改革优化政策性金融。提高直接融资比重，稳步推进主板（中小板）、新三板注册制改革，完善常态化退市机制，提高上市公司质量，促进资本市场健康发展。纵深推进能源、资源等重点领域价格改革，加强和完善垄断环节成本监审，持续深化电价改革，建立健全油气管网运输价格机制，积极推进水资源价格改革。

（五）深入推进高水平对外开放，建设更高水平开放型经济新体制。推动贸易和投资自由化便利化，依托我国大市场优势，促进国际合作，实现互利共赢。

一是稳住外贸外资基本盘。加强外贸政策支持保障，稳住贸易规模和市场主体。持续推进贸易创新发展，优化国内国际市场布局、商品结构、贸易方式，提升出口质量，增加优质产品进口。全面落实外商投资法及其实施条例，保护外商投资合法权益，健全准入前国民待遇加负面清单管理制度和外商投资国家安全审查制度。进一步缩减外商投资准入负面清单，落实2020年版鼓励外商投资产业目录，积极引导外资投向先进制造、高新技术、节能环保等领域和中西部地区。完善对人民币使用的支持体系，继续推动金融市场高质量双向开放。

二是推动共建“一带一路”高质量发展。坚持共商共建共享原则，秉持绿色、开放、廉洁理念，深化务实合作。严格做好境外项目疫情防控，大力推进健康、绿色、数字丝绸之路建设。提升境外投资质量效益，促进对外投融资基金健康发展。深入推进与重点国家和地区规划对接，加强产能合作。有序推进境外经贸合作区建设。推动铁路、港口、能源等互联互通重大项目取得积极进展，高标准推进中巴经济走廊建设，稳妥有序推动中蒙俄经济走廊等重大项目建设。积极推进中国与欧盟、新加坡、韩国、日本等第三方市场合作，推动与相关国家签署第三方市场合作重点项目清单。继续深化与国际组织共建“一带一路”合作。积极做好风险防范和处置应对，有效维护境外投资经营合法权益。拓展“丝路电商”合作。加强中欧班列通道能力、枢纽节点、口岸扩能及海外仓建设，推进中欧班列集结中心示范工程建设和政府间合作机制建设。商签中新共建国际陆海贸易新通道合作规划，推动国际陆海贸易新通道与澜湄合作对接发展。突出特色做好新疆、福建“一带一路”核心区建设，推进长江经济带发展等区域重大战略与共建“一带一路”深度融合。讲好共建“一带一路”故事。

三是加快对外开放高地建设。赋予自贸试验区更大改革自主权，狠抓改革试点任务落实，推动形成更多可复制可推广的制度创新成果。推动海关特殊监管区域与自贸试验区统筹发展。稳步推进海南自由贸易港建设，推动各项早期政策安排落地实施，推动出台海南自由贸易港法，积极推进国际旅游消费中心建设。提高沿边开发开放水平，支持沿边重点开发开放试验区建设。全面深化服务贸易创新发展试点，新增一批服务外包示范城市。

四是积极参与全球经济治理。坚定维护多边贸易体制，推动二十国集团发挥国际经济合作功能，积极参与世界贸易组织改革，推动国际金融体系改革。实施自由贸易区提升战略，构建面向全球的高标准自由贸易区网络，推动区域全面经济伙伴关系协定尽早生效实施，签署落实中欧投资协定，加快中日韩等自贸区谈判进程，加大力度支持非洲自贸区建设。积极考虑加入全面与进步跨太平洋伙伴关系协定。

（六）全面推进乡村振兴，稳步推进农业农村现代化。坚决巩固脱贫攻坚成果，坚持农业农村优先发展方针，实行农业现代化与农村现代化一体设计、一并推进。

一是做好巩固拓展脱贫攻坚成果同乡村振兴有效衔接。对脱贫县从脱贫之日起设立5年过渡期，过渡期内保持现有主要帮扶政策总体稳定。健全防止返贫动态监测和帮扶机制，对易返贫致贫人口及时发现、及时帮扶。扎实做好易地搬迁后续帮扶工作，继续完善易地扶贫搬迁集中安置区配套基础设施，持续加大就业和产业扶持力度。加大对脱贫县乡村振兴支持力度，实施脱贫地区特色种养业提升行动，深化拓展消费帮扶。在农业农村基础设施建设领域大力推广以工代赈方式，吸纳更多脱贫人口和低收入人口就地就近就业。坚持和完善东西部协作和对口支援、社会力量参与帮扶等机制。加强农村低收入人口常态化帮扶。

二是深入实施藏粮于地、藏粮于技战略。落实新一轮高标准农田建设规划，充分考虑水资源条件和水资源刚性约束，建设1亿亩旱涝保收、高产稳产高标准农田。实施国家黑土地保护工程，推广保护性耕作模式。加强农业种质资源保护开发利用，实施现代种业提升工程，有序推进生物育种产业化应用，强化制种基地和良种繁育体系建设。实施农业关键核心技术攻关工程，深入开展乡村振兴科技支撑行动，支持高端智能、丘陵山区农机装备研发制造，大力推进农业机械化。强化动物防疫和农作物病虫害防治体系建设。继续支持大中型灌区续建配套和现代化改造，推进病险水库除险加固。

三是加快发展乡村产业。依托乡村特色优势资源，打造农业全产业链，把产业链主体留在县域，建设农村一二三产业融合发展示范园、科技示范园区、现代农业产业园、农业产业强镇、优势特色产业集群，完善利益联结机制，让农民更多分享产业增值收益。完善农村产权制度和要素市场化配置机制，鼓励发展多种形式适度规模经营。支持农村产业化龙头企业创新发展、做大做强。加大对农产品分拣、加工、包装、预冷等一体化集配设施建设支持力度。加快完善县乡村三级节点农村物流体系，实施农产品仓储保鲜冷链物流设施建设工程，推进公益性农产品市场和农产品流通骨干网络建设。发展乡村旅游等多元业态，培育农产品电商品牌。完善农村生活性服务业支持政策。

四是实施乡村建设行动。大力提升农房设计和建设质量，提高农房现代化水平。加强畜禽粪污资源化利用，全面实施秸秆综合利用、农膜、农药包装物回收行动。因地制宜扎实推进农村厕所革命，加快推进农村生活污水治理，健全农村生活垃圾收运处置体系，推进农村生活垃圾分类减量与处理利用，开展美丽宜居村庄创建行动。统筹县域城镇和村庄规划建设，完善乡村水电路气、通信、广播电视、邮政等基础设施。实施农村供水保障工程，加强中小型水库等稳定水源工程建设和水源保护。实施农村水系综合治理。实施农村道路畅通、乡村清洁能源建设和数字乡村建设发展等工程，全面巩固提升农村电力保障水平。提升农村基本公共服务水平，建立城乡公共资源均衡配置机制。加快县域内城乡融合发展，实现县乡村功能衔接互补。

（七）大力推进区域协调发展，优化国土空间布局和支撑体系。纵深推进新型城镇化战略，坚持实施区域重大战略、区域协调发展战略、主体功能区战略，健全区域协调发展体制机制，着力打造带动全国高质量发展的新动力源。

一是着力推动新型城镇化高质量发展。研究出台《国家新型城镇化规划（2021－2035年）》。深化户籍制度改革，完善财政转移支付和城镇新增建设用地规模与农业转移人口市民化挂钩政策，依法坚决维护进城落户农民农村权益，提高农业转移人口享有的保障性住房、职业技能培训、子女义务教育等城镇基本公共服务水平。建立健全城市群规划实施机制和一体化发展机制。支持重点都市圈加快轨道交通网络建设。更好发挥中心城市对城市群和都市圈建设的带动作用，加强超大特大城市治理中的风险防控，提升大中城市功能品质，推进县城补短板强弱项，强化县城综合服务能力。促进特色小镇规范健康发展。因地制宜实施城市更新行动，推进老旧小区、老旧厂区、老旧街区、城中村等改造，新开工改造城镇老旧小区5.3万个。开展城市现代化试点示范，推动城市结构优化、功能完善和品质提升，加快建设宜居、绿色、韧性、智慧、人文城市。推进城市生态恢复、功能完善工程，强化历史文化保护，塑造城市风貌，强化社区管理服务功能。支持有条件的城市推进海绵城市建设。推动城市治理科学化精细化智能化。健全城乡融合发展体制机制，支持国家城乡融合发展试验区率先改革突破。

二是深入实施区域重大战略。以“十四五”规划实施方案为引领，统筹支持区域重大战略实施。持续推进京津冀协同发展，完善北京非首都功能疏解政策体系，推进雄安新区高标准高质量建设，推动北京城市副中心高质量发展，落实支持天津滨海新区高质量发展的意见，继续实施一批协同发展重大项目、重大改革、重大政策。推进长江经济带高质量发展，加强生态环境综合治理、系统治理、源头治理，深入推进污染治理“4+1”工程，持续巩固长江禁捕退捕成效，开展长江生物多样性保护工程。打造若干生态产品价值实现机制示范基地，加快完善长江经济带综合运输体系。深入推进粤港澳大湾区建设，完善国际科技创新中心“两廊”、“两点”空间布局，促进要素高效便捷流动，深入推进深圳建设中国特色社会主义先行示范区综合改革试点，推进前海深港现代服务业合作区、横琴粤澳深度合作区和河套深港科技创新合作区发展。大力推进长三角一体化发展，出台支持浦东新区高水平改革开放打造社会主义现代化建设引领区的意见，支持浦东探索开展综合性改革试点，鼓励长三角地区推进新型基础设施建设，总结推广长三角生态绿色一体化发展示范区制度创新经验，统筹推进上海自贸试验区临港新片区规划建设和发展。稳步推进黄河流域生态保护和高质量发展，加快制定形成“1+N+X”规划和政策体系。

三是扎实落实区域协调发展战略。推动构建更加有效的区域协调发展新机制。研究出台西部大开发“十四五”实施方案和重点地区规划，继续实施西部大开发企业所得税优惠政策，执行新修订的《西部地区鼓励类产业目录》，推进成渝地区双城经济圈建设，打造带动全国高质量发展的重要增长极和新的动力源，提升关中平原城市群建设水平，促进西北地区与西南地区合作互动，积极推动西部大开发形成新格局。研究出台东北振兴“十四五”实施方案，着力推动东北地区产业结构调整优化，支持东北地区加大开放合作，推动东北振兴取得新突破。研究出台《关于新时代推动中部地区高质量发展的指导意见》，继续支持湖北省经济社会发展，促进中部地区加快崛起。大力推进山东新旧动能转换，支持福建高质量发展及平潭综合实验区建设，有力促进环渤海地区合作加快发展，深入推进淮河生态经济带建设，鼓励东部地区加快推进现代化。支持革命老区加快发展，贯彻落实《关于新时代支持革命老区振兴发展的意见》，促进民族地区经济社会发展，加强边境地区建设，加快推进资源型地区转型升级，支持老工业基地转型发展、承接产业转移。支持国家级新区、经济开发区、高新区创新发展。完善推进海洋经济高质量发展的政策体系，培育壮大海洋战略性新兴产业。

（八）加快推动绿色低碳发展，持续加强生态文明建设。建立完善生态文明领域统筹协调机制，紧盯目标任务，持续精准发力，进一步改善生态环境质量，促进经济社会发展全面绿色转型。

一是扎实开展碳达峰、碳中和工作。加强顶层设计和统筹谋划，研究出台做好碳达峰碳中和工作指导意见。坚持推进节能减排，完善能源消费总量和强度双控制度，实施“十四五”节能减排综合工作方案，加强重点领域节能，加快建设全国用能权交易市场。坚决遏制高耗能高排放项目盲目发展。实施积极应对气候变化国家战略，制定2030年前碳排放达峰行动方案和应对气候变化中长期目标保障措施。建设并运行全国碳排放权注册登记结算系统和交易系统，完善温室气体自愿减排交易机制。深化应对气候变化试点示范，启动气候投融资地方试点。积极推进应对气候变化多边进程和国际合作，推进气候变化南南合作。研究制定中长期能源发展规划纲要，出台构建清洁能源增长消纳和储能协调有序发展体制机制的指导意见。加强西部能源基地外送电力通道建设，科学有序推进水电发展，积极有效推进风电、光伏发电、垃圾发电及氢能等能源发展，在确保安全的前提下积极有序发展核电，提高非化石能源消费占比，加大抽水蓄能和储能发展支撑力度，大力提升电力系统调节能力。

二是深入打好污染防治攻坚战。巩固蓝天、碧水、净土保卫战成果，加强细颗粒物（PM2.5）、臭氧（O3）等多污染物协同控制，北方地区清洁取暖率达到70%，加快淘汰报废老旧柴油货车，强化区域大气污染防治协作，聚焦长江、黄河、粤港澳大湾区等重点流域和海湾，推进美丽河湖、美丽海湾保护和建设，持续推动城市和农村黑臭水体治理。深入开展土壤污染防治行动，严格农用地安全利用和建设用地风险管控，加强农业面源污染防治，推进化肥农药减量化。落实“精准治污、科学治污、依法治污”要求，制定实施构建现代环境治理体系三年工作方案，推进构建以排污许可制为核心的固定污染源监管制度体系。深入推进塑料污染全链条治理，统筹开展快递包装绿色转型，加快城镇环境基础设施发展，持续提升危险废物处理处置能力，构建污水资源化利用“1+N”政策体系，推进污水资源化利用重点工程建设，补齐城镇生活污水处理设施短板弱项，加快推进长江经济带、黄河流域污水处理和资源化利用。加强噪声污染综合防治。持续推动货物运输“公转铁”。

三是加快推动形成绿色生产生活方式。深入推进国家生态文明试验区建设。大力发展循环经济，加快构建废旧物资循环利用体系。扩大环境保护、节能节水等企业所得税优惠目录范围，促进新型节能环保技术、装备和产品研发应用，培育壮大节能环保产业。大力发展绿色金融。推进重点行业和重要领域绿色化改造，开展清洁生产评价与认证，提升重点行业和园区清洁生产水平。推动绿色建筑发展。大力促进资源节约集约高效利用，推动大宗固体废弃物资源综合利用，持续推进危险废弃物环境风险排查整治，强化环境风险评估与管控。推进垃圾分类和减量化、资源化，稳步推开“无废城市”建设。推动节水型城市建设，推进海水淡化规模化利用示范。开展绿色社区创建行动，提倡“厉行节约、反对浪费”的社会风尚。

四是提升生态系统质量。制定生态保护红线监管办法，出台生态保护补偿条例，统筹推进生态环境损害赔偿制度改革。优化国家生态安全屏障体系，坚持山水林田湖草系统治理，实施全国重要生态系统保护和修复重大工程，统筹开展大规模国土绿化行动，科学推进荒漠化、石漠化、水土流失综合治理，加强重大战略区域重要湿地保护，强化海岸带生态保护和修复。调整优化国家公园布局，加快构建以国家公园为主体的自然保护地体系，持续加强自然保护地生态环境监管。加强生物多样性保护工作，举办《生物多样性公约》第十五次缔约方大会。

（九）进一步增进民生福祉，增强人民群众获得感、幸福感、安全感。把实现好、维护好、发展好最广大人民根本利益作为发展的出发点和落脚点，促进人的全面发展，扎实推动共同富裕。

一是持续提高居民收入水平。通过提升就业质量、强化人力资本、畅通社会流动、激发要素活力等方式，使各类社会群体依靠自身努力和智慧提高收入、创造财富。统筹推进收入分配重点领域改革，进一步健全工资指导线和企业薪酬调查制度，加大再分配调节力度和精准性。把促进全体人民共同富裕摆在更加重要的位置，精心谋划共同富裕顶层设计，研究开展共同富裕示范区建设，研究制定“十四五”时期扩大中等收入群体实施方案。

二是加快发展教育事业。多措并举扩大普惠性学前教育资源供给，完善普惠性学前教育保障机制。推动义务教育优质均衡发展和城乡一体化，提升高中阶段教育普及水平。分类推进“双一流”建设和应用型本科高校发展，全面振兴本科教育，推进新工科、新农科、新医科、新文科建设。加强教师队伍建设，加大中西部地区教师培训力度。健全教师工资保障长效机制，改善乡村教师待遇。保障进城务工人员随迁子女平等接受义务教育，进一步扩大城镇义务教育容量。纵深推进产教融合改革，推出首批国家产教融合型企业和试点城市。推动职业教育提质培优，完善高职院校“分类招考”制度，健全普职融通制度。开展多样化的在职培训和继续教育。

三是加大社会保障力度。提高退休人员基本养老金、优抚对象抚恤和生活补助标准。完善全国统一的社会保险公共服务平台。推进基本养老保险全国统筹。发展多层次、多支柱养老保险体系，支持第三支柱养老保险发展。推动失业保险、工伤保险省级统筹，全面做实基本医疗保险市地级统筹，鼓励有条件的省份推进省级统筹。居民医保和基本公共卫生服务经费人均财政补助标准分别再增加30元和5元。健全重大疾病医疗保险和救助制度，落实异地就医结算，稳步推进长期护理保险制度试点，积极发展商业医疗保险。建立健全门诊共济保障机制，逐步将门诊费用纳入统筹基金报销。建立健全医疗保障待遇清单制度，完善医保药品目录动态调整机制，推动药品集中采购常态化制度化。健全灵活就业人员社保制度，推进新就业形态人员职业伤害保障试点。健全退役军人工作体系和保障制度。健全分层分类的社会救助体系，建立完善社会救助主动发现机制，加大城镇困难群众脱困解困力度。保障妇女儿童合法权益，健全市、县、乡未成年人救助保护网络，完善残疾人、孤儿等社会福利制度。

四是保障好群众住房需求。坚持房子是用来住的、不是用来炒的定位，稳地价、稳房价、稳预期。解决好大城市住房突出问题，通过增加土地供应、安排专项资金、集中建设等办法，切实增加保障性租赁住房和共有产权住房供给，重视解决好新市民、青年人等住房困难群体的住房问题。加快完善长租房政策，逐步使租购住房在享受公共服务上具有同等权利，规范发展长租房市场，降低租赁住房税费负担。加大住房租赁市场金融支持，支持专业化、规模化住房租赁企业发展。

五是加强社会公共服务体系建设。推动出台“十四五”公共服务发展规划，完善均等化推进机制，推动国家基本公共服务标准落地落实。持续推进健康中国行动。启动实施优质高效医疗卫生服务体系建设工程，推进疾病预防控制体系现代化，加强心理健康服务体系建设和规范化管理，发展全方位全生命周期健康服务。推进国家医学中心建设，深入推进区域医疗中心建设试点，加快优质医疗资源扩容和均衡布局。启动实施医疗保障服务示范工程，推动医疗保障公共服务标准化、规范化、精细化。建立健全农村医疗卫生服务网络和城市社区卫生服务网络，提高医疗保障水平。坚持中西医并重，实施中医药振兴发展重大工程。实施积极应对人口老龄化国家战略，增强生育政策包容性，着力降低生育、养育、教育成本。发展普惠型养老服务和互助性养老，构建居家社区机构相协调、医养康养相结合的养老服务体系，健全养老服务综合监管制度，加快推进养老产业发展，统筹推进农村养老服务发展。发展普惠托育服务体系。深化家政服务业“领跑者”行动，培育员工制家政企业，推动家政进社区。推进长城、大运河、长征、黄河等国家文化公园建设，着力打造中华文化重要标志。实施文化保护传承利用工程，加强重要文化遗产和国家公园等重要自然遗产保护利用，推进故宫博物院北院区、国家美术馆等重大文化设施项目建设。繁荣发展哲学社会科学。促进文化和旅游融合发展，培育世界级旅游景区和度假区、国家级休闲城市和街区。出台实施国民休闲纲要（2021－2035年），推动落实带薪休假制度。深入推进北京冬奥会、冬残奥会筹办工作。制定构建更高水平的全民健身服务体系的意见，打造贴近自然、便捷高效的群众健身场地。开展社会服务设施兜底线工程，支持儿童福利设施、残疾人康复和托养设施建设，进一步补上退役军人等领域基础设施短板，提升兜底保障能力。加强和创新社会治理，健全城乡社区治理和服务体系。深化公共法律服务体系建设。完善和落实安全生产责任制，加强安全生产监管执法。强化生物安全保护，加强国家生物数据中心体系和高级别生物安全实验室建设，提高食品药品安全保障水平，加强网络食品安全监督管理，启动建设食品安全风险评估与标准研制重点实验室。加强应急救援力量建设，提高防灾减灾抗灾救灾能力，切实做好洪涝干旱、森林草原火灾、地质灾害、地震等防御和气象服务。

（十）统筹发展和安全，防范化解重点领域风险。坚持总体国家安全观，不断加强经济安全风险预警、防控机制和能力建设，实现关键领域安全可控，守住不发生系统性风险底线。

一是提升粮食和重要农产品供给保障能力。加快推动粮食安全保障立法。深入实施重要农产品保障战略，严格落实粮食安全省长责任制和“菜篮子”市长负责制，确保粮棉油糖肉等供给安全。落实最严格的耕地保护制度，支持利用撂荒地发展粮食生产，稳定粮食播种面积、提高单产水平。强化粮食“产购储加销”协同保障机制。完善粮食主产区利益补偿机制，加强粮食生产功能区和重要农产品生产保护区建设，建设国家粮食安全产业带。深入推进优质粮食工程，加快粮食产业高质量发展。适度提高稻谷、小麦最低收购价，完善玉米、大豆生产者补贴政策，完善产粮大县奖励政策，保护农民种粮积极性。深入推进农业结构调整，扩大玉米种植面积，稳定大豆生产，多措并举发展油菜、花生等油料作物，稳定棉花、糖料种植面积，集成推广绿色高质高效技术模式。持续抓好生猪生产恢复，积极发展牛羊产业，继续实施奶业振兴行动。健全粮食、生猪产业监测预警体系，充分发挥储备调节作用，提升收储调控和应急保障能力，推进粮食流通现代化。实施重要农产品进口多元化战略。加快培育大型跨国农业企业，加强粮食和重要农产品境外供应链建设。开展粮食节约行动，坚持不懈制止餐饮浪费。

二是保障能源资源安全。继续推进能源产供储销体系建设，加强网络布局、规划建设、运行管理、调配调度等方面有机衔接。优化煤炭生产开发布局和产能结构，系统提升煤炭供应保障能力。提升电力运行水平，推进风光水火核储一体化发展，充分发挥煤电调峰、应急、支持和兜底保供作用。大力提升油气勘探开发力度，实施天然气管网互联互通工程，强化油气供应保障基础。积极推进能源通道建设，增强能源储备能力，提升运输保障能力。完善应急保障预案，提升风险应对和应急调节能力，增强能源安全韧性。继续开展找矿突破战略行动，实施矿产地储备工程，全面推进矿业权竞争性出让，积极推进“净矿出让”，调动各类市场主体积极性。加强矿产资源开发管理和保护，提升战略性矿产资源储备安全保障能力。积极参与全球矿业治理，持续开展国际矿业合作。

三是维护金融等领域安全。严格落实地方政府债务风险防控相关规定，坚决遏制隐性债务增量、积极稳妥化解隐性债务存量。完善现代金融监管体系，提升金融监管能力，加强对金融创新的审慎监管。稳妥处置重点领域风险，健全金融风险预防、预警、处置、问责制度体系。完善存款保险制度，充分发挥早介入、早预警、早处置的作用。全面加强网络安全保障体系和能力建设。

全面准确贯彻“一国两制”、“港人治港”、“澳人治澳”、高度自治的方针，维护国家主权、安全、发展利益和特别行政区社会大局稳定。支持香港、澳门更好融入国家发展大局，建设国际创新科技中心，打造“一带一路”功能平台，实现经济多元可持续发展，完善便利港澳居民在内地发展政策措施。坚持一个中国原则和“九二共识”，坚决遏制“***”分裂活动，推动两岸关系和平发展、融合发展，加强两岸产业合作，打造两岸共同市场，壮大中华民族经济，共同弘扬中华文化。

2021年经济社会发展工作任务繁重，意义重大。我们要更加紧密地团结在以习近平同志为核心的党中央周围，高举中国特色社会主义伟大旗帜，以习近平新时代中国特色社会主义思想为指导，增强“四个意识”、坚定“四个自信”、做到“两个维护”，按照党中央、国务院决策部署，自觉接受全国人大的监督，认真听取全国政协的意见和建议，不忘初心、牢记使命，齐心协力、开拓进取，以钉钉子精神做实做细各项工作，努力在危机中育先机，于变局中开新局，确保全面建设社会主义现代化国家新征程开好局、起好步，以优异成绩庆祝中国共产党成立100周年。

第十三届全国人民代表大会财政经济委员会关于2020年国民经济和社会发展

计划执行情况与2021年国民经济和社会发展计划草案的审查结果报告

（2021年3月9日第十三届全国人民代表大会第四次会议主席团第二次会议通过）

十三届全国人大四次会议主席团：

第十三届全国人民代表大会第四次会议审查了国务院提出的《关于2020年国民经济和社会发展计划执行情况与2021年国民经济和社会发展计划草案的报告》和2021年国民经济和社会发展计划草案。全国人民代表大会财政经济委员会在对计划报告和计划草案初步审查的基础上，根据各代表团和有关专门委员会的审查意见，又作了进一步审查。国务院根据审查意见对计划报告作了修改。现将审查结果报告如下。

一、2020年计划执行情况总体良好

财政经济委员会认为，2020年国民经济和社会发展计划执行情况总体良好。面对严峻复杂的国际形势、艰巨繁重的国内改革发展稳定任务特别是新冠肺炎疫情的严重冲击，以习近平同志为核心的党中央统筹疫情防控和经济社会发展，疫情防控取得重大战略成果，在全球主要经济体中唯一实现经济正增长，脱贫攻坚战取得全面胜利，决胜全面建成小康社会取得决定性成就，交出一份人民满意、世界瞩目、可以载入史册的答卷。国务院和地方各级人民政府以习近平新时代中国特色社会主义思想为指导，全面贯彻党的十九大和十九届二中、三中、四中、五中全会以及中央经济工作会议精神，按照党中央决策部署和十三届全国人大三次会议各项决议要求，扎实做好“六稳”工作，全面落实“六保”任务，着力推动高质量发展，经济运行逐步恢复常态，经济总量迈上100万亿元大台阶。十三届全国人大三次会议批准的2020年国民经济和社会发展计划确定的主要目标任务较好完成，我国改革开放和社会主义现代化建设又取得新的重大进展，为开启全面建设社会主义现代化国家新征程奠定了基础。

同时也要看到，当前国内外环境面临深刻复杂变化。国际形势中不稳定不确定因素增多，新冠肺炎疫情仍在全球蔓延，世界经济复苏乏力。我国经济恢复基础尚不牢固，新冠肺炎疫情“外防输入、内防反弹”任务艰巨，有效需求仍显疲弱，实体经济特别是民营和小微企业仍较困难，重点领域安全风险不容忽视，关键核心技术“卡脖子”问题仍然突出，民生领域还有薄弱环节。对于这些困难和问题，要按照中央经济工作会议要求，进一步坚持系统观念，加强全局谋划和统筹协调，尽快加以解决。

二、2021年计划报告和计划草案总体可行

财政经济委员会认为，国务院提出的2021年计划报告和计划草案，符合《中共中央关于制定国民经济和社会发展第十四个五年规划和二〇三五年远景目标的建议》和中央经济工作会议精神，符合我国经济社会发展实际，主要指标和工作安排总体可行。建议第十三届全国人民代表大会第四次会议批准国务院提出的《关于2020年国民经济和社会发展计划执行情况与2021年国民经济和社会发展计划草案的报告》，批准2021年国民经济和社会发展计划草案。

三、做好2021年计划执行工作的建议

2021年是中国共产党成立100周年，是实施“十四五”规划、开启全面建设社会主义现代化国家新征程的第一年，在党和国家事业发展进程中具有特殊重要性，做好经济社会发展工作意义重大。要在以习近平同志为核心的党中央坚强领导下，以习近平新时代中国特色社会主义思想为指导，全面贯彻党的十九大和十九届二中、三中、四中、五中全会以及中央经济工作会议精神，增强“四个意识”、坚定“四个自信”、做到“两个维护”，坚持稳中求进工作总基调，立足新发展阶段，贯彻新发展理念，构建新发展格局，以推动高质量发展为主题，以深化供给侧结构性改革为主线，以改革创新为根本动力，以满足人民日益增长的美好生活需要为根本目的，坚持系统观念，巩固拓展疫情防控和经济社会发展成果，更好统筹发展和安全，扎实做好“六稳”工作、全面落实“六保”任务，科学精准实施宏观政策，努力保持经济运行在合理区间，坚持扩大内需战略，强化科技战略支撑，扩大高水平对外开放，保持社会和谐稳定，确保“十四五”开好局起好步，以优异成绩庆祝中国共产党成立100周年。为此，财政经济委员会提出以下建议：

（一）更好统筹疫情防控和经济社会发展，稳定预期提振信心。切实抓紧抓实“外防输入、内防反弹”，科学推进疫苗研制生产、接种使用工作。持续优化常态化疫情防控举措，积极构建统筹疫情防控和经济社会发展的中长期协调机制。保持宏观政策连续性、稳定性、可持续性，不急转弯，根据形势变化适时调整优化。继续为市场主体纾困，保持必要支持力度，进一步巩固经济基本盘。加强宏观政策协调配合，形成政策合力，稳定市场主体预期，提振信心。

（二）加快科技自立自强，强化国家战略科技力量。把科技自立自强作为国家发展的战略支撑，发挥新型举国体制优势，推进国家实验室建设，强化企业创新主体地位，促进产学研用深度融合，推动科技力量优化配置和资源共享。提高基础研究经费投入占研发经费投入比重，完善稳定支持机制。加快推进关键核心技术攻关。加大知识产权保护力度，完善创新激励机制和科技评价机制，提高科技成果转化率。加强重点产业链、供应链安全监测评估，统筹推进补短板和锻长板，增强产业链、供应链自主可控能力。

（三）坚持扩大内需这个战略基点，加快形成强大国内市场。加快培育完整内需体系，建设现代流通体系和全国统一大市场。适应居民消费多元化和结构升级趋势，推动线上线下融合发展，健全社会信用体系，营造良好消费环境，全面促进消费。依托强大国内市场，吸引全球资源要素，促进内外贸一体化。发挥政府投资引领作用，激发全社会投资活力，鼓励民间资本参与重大工程项目，推动重大项目落地实施，增强投资增长后劲。持续深化供给侧结构性改革，推动制造业高质量发展，统筹产业布局，避免重复建设，鼓励企业加快设备更新和技术改造。大力发展数字经济，加强顶层设计和法律规范，推动数字经济健康发展。深入推进以人为核心的新型城镇化，促进城乡融合发展、区域协调发展。

（四）持续激发市场主体活力，促进实体经济健康发展。帮助市场主体特别是中小微企业和个体工商户解决生产经营中遇到的难题。继续执行制度性减税政策，实施新的结构性减税举措，坚决制止涉企乱收费。认真落实《保障中小企业款项支付条例》，加大清理拖欠企业账款力度。保持流动性合理充裕，强化普惠金融服务，促进中小微企业融资更加便利，降低融资成本。支持各类企业包括平台企业创新规范发展，预防和规制平台经济垄断和不正当竞争行为，引导互联网经济健康发展。

（五）防范化解重点领域风险，统筹发展和安全。坚决遏制地方政府隐性债务增量、积极化解隐性债务存量，切实缓解基层“三保”困难。完善现代金融监管体系，保持宏观杠杆率基本稳定，完善债券市场基础制度，前瞻性研判金融风险，提升系统性金融风险防范化解能力。深入实施重要农产品保障战略，提高粮食和重要农产品供给保障能力，加强种质资源保护利用和技术攻关。深入开展节约粮食行动，坚持不懈制止餐饮浪费。保障能源资源安全，提升风险应对和应急调控能力。维护新型领域安全，加强网络安全能力建设。

（六）坚定不移深化改革扩大开放，持续增强发展内生动力。加快建设高标准市场体系，完善公平竞争制度，推进要素资源市场化配置。继续深化“放管服”改革，营造市场化、法治化、国际化营商环境。推进国有经济布局优化和结构调整，进一步放宽市场准入，依法平等保护民营企业产权和企业家权益。完善多层次、广覆盖、差异化金融机构体系，健全金融机构治理，促进资本市场健康发展，提升直接融资比重。多措并举稳定外贸外资基本盘，有序扩大服务业对外开放。发挥好自贸区（港）等各类开放平台作用，推动共建“一带一路”高质量发展。

（七）着力保障改善民生，促进社会事业全面发展。把促进共同富裕放在更加重要的位置，逐步健全基本公共服务体系。落实就业优先政策，扩大就业容量，提高劳动者技能素质和就业质量。持续提高居民收入水平，健全再分配机制。促进教育发展更加公平更高质量。建立健全巩固拓展脱贫攻坚成果同乡村振兴有效衔接机制，坚决防止规模性返贫。聚焦解决农民群众“急难愁盼”问题，大力实施乡村建设行动，推进农业农村现代化。坚持绿水青山就是金山银山理念，深入打好污染防治攻坚战，持续改善环境质量。繁荣发展文化事业和文化产业。健全多层次社会保障体系，加快实现基本养老保险全国统筹，推动基本医疗保险、失业保险、工伤保险省级统筹。加强医疗卫生机构建设，提高公共卫生治理能力。积极应对人口老龄化，加快完善养老服务体系。解决好大城市住房突出问题。完善和落实安全生产责任制，提高防灾、减灾、抗灾、救灾能力。

以上报告，请审议。

第十三届全国人民代表大会财政经济委员会

2021年3月9日
