# Supplementary material for: Mapping the evolution of fertility support policies in China: A content and instrumental analysis
Source: PLoS One. 2025 Oct 9;20(10):e0332137. doi: 10.1371/journal.pone.0332137 (PMC12510515; doi:10.1371/journal.pone.0332137)
Supplement: S1 Appendix — (ZIP) [file pone.0332137.s001.zip › S1 Appendix. 226 original policy documents/206-第十三届全国人民代表大会第三次会议关于2019年国民经济和社会发展计划执行情况与2020年国民经济和社会发展计划的决议(FBM-CLI-1-342612).docx]

第十三届全国人民代表大会第三次会议关于2019年国民经济和社会发展计划执行情况与2020年国民经济和社会发展计划的决议

发布部门： 全国人民代表大会

发布日期：2020.05.28

实施日期：2020.05.28

时效性： 现行有效

效力级别： 工作文件

法规类别： 人大议事 营商环境优化

专题分类： 京津冀协同发展 长江经济带发展 黄河流域生态保护和高质量发展

第十三届全国人民代表大会第三次会议关于2019年国民经济和社会发展计划执行情况与2020年国民经济和社会发展计划的决议

（2020年5月28日第十三届全国人民代表大会第三次会议通过）

第十三届全国人民代表大会第三次会议审查了国务院提出的《关于2019年国民经济和社会发展计划执行情况与2020年国民经济和社会发展计划草案的报告》及2020年国民经济和社会发展计划草案，同意全国人民代表大会财政经济委员会的审查结果报告。会议决定，批准《关于2019年国民经济和社会发展计划执行情况与2020年国民经济和社会发展计划草案的报告》，批准2020年国民经济和社会发展计划。

关于2019年国民经济和社会发展计划执行情况与2020年国民经济和社会发展计划草案的报告

—— 2020年5月22日在第十三届全国人民代表大会第三次会议上

国家发展和改革委员会

各位代表：

受国务院委托，现将2019年国民经济和社会发展计划执行情况与2020年国民经济和社会发展计划草案提请十三届全国人大三次会议审议，并请全国政协各位委员提出意见。

一、2019年国民经济和社会发展计划执行情况

2019年，在以习近平同志为核心的党中央坚强领导下，各地区各部门以习近平新时代中国特色社会主义思想为指导，深入贯彻党的十九大和十九届二中、三中、四中全会精神，增强“四个意识”、坚定“四个自信”、做到“两个维护”，全面贯彻落实党中央、国务院决策部署，认真执行十三届全国人大二次会议审议批准的《政府工作报告》、2019年国民经济和社会发展计划，落实全国人大财政经济委员会审查意见，坚持稳中求进工作总基调，深入贯彻新发展理念，坚持以供给侧结构性改革为主线，推动高质量发展，扎实做好“六稳”工作，统筹推进稳增长、促改革、调结构、惠民生、防风险、保稳定，全年经济社会发展主要目标任务较好完成，“十三五”规划主要指标完成进度符合预期，为全面建成小康社会打下决定性基础。

（一）科学实施宏观调控，经济运行保持在合理区间。加强区间调控、定向调控、相机调控、精准调控，加强政策协调配合，强化预期管理，促进经济平稳健康发展。

一是主要宏观指标完成情况良好。国内生产总值达到99.1万亿元，增长6.1%，符合预期目标。城镇新增就业1352万人，年末城镇调查失业率、城镇登记失业率分别为5.2%和3.62%。居民消费价格指数上涨2.9%。国际收支基本平衡，外汇储备保持在3万亿美元以上。

图表：关于2019年国民经济和社会发展计划执行情况与2020年国民经济和社会发展计划草案的报告（图1） 新华社发

二是宏观政策逆周期调节有力有效。以减税降费为重点，积极的财政政策加力提效，财政支出结构持续优化，民生等重点领域资金需求得到有力保障。全国一般公共预算收入19.04万亿元，增长3.8%；全国一般公共预算支出23.89万亿元，增长8.1%；财政赤字2.76万亿元，与预算持平。全年减税降费2.36万亿元。合理扩大专项债券使用范围，加快专项债券发行使用。稳健的货币政策松紧适度，逆周期调节效果持续显现，信贷结构不断优化，对实体经济特别是小微企业、民营企业信贷投放力度进一步加大。年末广义货币（M2）余额增长8.7%，社会融资规模存量增长10.7%。更大力度实施就业优先政策，援企稳岗力度进一步加大，失业保险基金稳岗返还政策全面落实，高校毕业生、农民工、退役军人等重点群体就业总体保持稳定。

（二）深入推进供给侧结构性改革，促进形成强大国内市场。坚持“巩固、增强、提升、畅通”八字方针，持续推进产业结构调整，着力畅通供需循环。

一是农业供给侧结构性改革深入推进。毫不放松抓好粮食生产，粮食总产量连续第5年保持在1.3万亿斤以上。启动重要农产品保障战略，实施大豆振兴计划。粮食生产功能区、重要农产品生产保护区基本划定。持续加强农田水利建设，完成8000万亩高标准农田和2000万亩高效节水灌溉任务。加强非洲猪瘟防控，加快恢复生猪生产，进一步完善蔬菜产供储销体系。完善粮食最低收购价政策和棉花目标价格政策，重要农产品收储制度和重要农资储备制度改革深入推进。农产品冷链物流仓储设施及冷链运输较快发展。农村产业融合发展持续推进，累计创建107个现代农业产业园、210个农村产业融合发展示范园。新型农业支持保护政策体系加快建立健全。

二是制造业转型升级步伐加快。出台推动制造业高质量发展政策措施，发布产业结构调整指导目录（2019年本）。运用市场化、法治化办法又淘汰煤炭落后产能1亿吨左右，稳妥推进钢铁企业兼并重组，推动重大石化项目建设。实施新一轮技术改造工程，推动中国标准地铁A型车等一批国产首台（套）技术装备示范应用。

三是服务业高质量发展扎实推进。出台支持服务业高质量发展、传统服务行业改造升级等政策措施，大力培育新业态新模式，推动先进制造业和现代服务业深度融合发展，支持共性技术研发、工业设计、工业互联网等平台建设。

图表：关于2019年国民经济和社会发展计划执行情况与2020年国民经济和社会发展计划草案的报告（图2） 新华社发

四是支持实体经济降成本力度加大。制造业等行业增值税税率从16%降至13%，交通运输业、建筑业等行业从10%降至9%，实施小微企业普惠性税收减免，小规模纳税人增值税起征点由月销售额3万元提高到10万元。企业职工基本养老保险单位缴费比例高于16%的省份已全部降至16%，阶段性降低失业、工伤保险费率政策延续一年。深化利率市场化改革，社会综合融资成本明显降低。清理政府部门和国有企业拖欠民营企业、中小企业账款6647亿元。进一步压减政府定价经营服务性收费目录，减免部分行政事业收费并降低收费标准。一般工商业平均电价再降10%，全年降低企业用电成本846亿元。扩大电力直接交易规模，降低企业购电成本约790亿元。降低成品油、天然气门站价格和跨省管道运输价格，减轻用户负担约650亿元。取消和降低铁路、港口、民用机场部分收费，减轻企业负担100多亿元。

五是消费惠民新增长点不断拓展。出台加快发展流通促进商业消费、促进家政服务业提质扩容、激发文化和旅游消费潜力、促进全民健身和体育消费、促进“互联网+社会服务”发展等政策措施，加大对夜间消费的支持力度，鼓励汽车、家电、电子产品更新消费。电子商务进农村综合示范深入实施，农村地区快递网点超过3万个，乡镇覆盖率达96.6%。全年社会消费品零售总额突破40万亿元，增长8.0%。全国网上零售额达10.6万亿元，增长16.5%，其中实物商品网上零售额增长19.5%，占社会消费品零售总额的20.7%。成功举办2019年中国品牌日系列活动。

图表：关于2019年国民经济和社会发展计划执行情况与2020年国民经济和社会发展计划草案的报告（专栏1） 新华社发

六是重点领域有效投资合理扩大。发布实施《政府投资条例》，适当降低重点领域项目资本金比例。健全重大项目储备机制，积极推进专项债券项目建设。规范有序推进政府和社会资本合作（PPP），鼓励民间资本参与补短板重点领域建设。172项重大水利工程已累计开工144项。印发实施交通强国建设纲要。23个国家物流枢纽建设稳步推进。川藏铁路前期工作扎实推进，北京大兴国际机场建成投运，乌东德、白鹤滩等大型水电站加快建设。2019年底，铁路营业里程达13.9万公里，其中高速铁路3.5万公里，民用运输机场达235个，新增220千伏及以上电网里程3.4万公里，油气干线里程0.4万公里。全年固定资产投资（不含农户）增长5.4%，其中民间投资增长4.7%；投资结构持续优化，高技术产业投资和社会领域投资分别增长17.3%和13.2%。

（三）全力抓重点补短板解难题，三大攻坚战取得重大进展。精准脱贫成效显著，生态环境质量总体改善，金融风险有效防控。

一是脱贫攻坚工作扎实推进。强化产业、就业、消费等扶贫，集中力量攻坚“三区三州”等深度贫困地区“两不愁三保障”突出问题。累计支持733万户建档立卡贫困户实施农村危房改造。累计建设易地扶贫搬迁安置区3.5万个、住房260余万套，可安置947万建档立卡易地扶贫搬迁人口，提前一年基本完成“十三五”规划建设任务。加大后续产业扶持和就业帮扶力度，定点帮扶等工作有力推进。全年农村贫困人口减少1109万，贫困县摘帽344个，贫困发生率降至0.6%。截至2019年底，97%现行标准的贫困人口实现脱贫，94%的贫困县实现摘帽，区域性整体贫困基本得到解决。

二是生态环境保护和污染防治有力推进。坚决打好蓝天、碧水、净土保卫战，细颗粒物（PM2.5）未达标地级及以上城市年均浓度下降2.4%，地表水质量达到或好于Ⅲ类水体比例为74.9%。非化石能源占能源消费比重达15.3%，提前一年完成“十三五”规划目标。启动第二轮中央生态环境保护例行督察。落实河长制湖长制。县级水源地生态环境问题整治基本完成，地级及以上城市黑臭水体消除近87%。坚定不移推进禁止洋垃圾入境，全国固体废物进口量减少40.4%。启动“无废城市”建设试点。加快实施排污许可制度。发布绿色产业指导目录（2019年版）。开展能源消耗总量和强度“双控”行动、国家节水行动、绿色生活创建行动。单位国内生产总值能耗下降2.6%，万元国内生产总值用水量下降6.1%。完善天然林保护制度，扩大退耕还林还草，实施荒漠化、石漠化综合治理。启动生态综合补偿试点。单位国内生产总值二氧化碳排放量降低4.1%。

三是金融等领域重大风险得到有效防控。地方政府隐性债务和企业债务风险处置稳妥推进，宏观杠杆率过快上升势头得到遏制。影子银行无序发展得到有效治理，部分高风险金融机构特别是中小银行“精准拆弹”取得阶段性成果，互联网金融等涉众风险得到治理。金融市场运行平稳有序，外汇市场和人民币汇率总体稳定。金融监管制度进一步完善。

（四）深入实施创新驱动发展战略，科技创新能力进一步提升。不断深化科技体制改革，大力支持基础研究和应用基础研究，全国研究与试验发展经费投入强度达2.19%，科技进步贡献率提高到59.5%。

一是自主创新步伐加快。重大科技成果持续涌现，嫦娥四号成功在月球背面着陆，北斗三号全球系统核心星座部署全面完成，5G商用加速推出，长征五号遥三运载火箭成功发射，首艘国产航母“山东舰”正式列装。科技创新2030－重大项目和国家科技重大专项深入实施，高能同步辐射光源等一批国家重大科技基础设施开工建设。全面创新改革试验稳步推进，169项先行先试改革举措基本完成。北京、上海科技创新中心建设取得重要进展，粤港澳大湾区国际科技创新中心建设顺利起步。北京怀柔、上海张江、安徽合肥等综合性国家科学中心建设全面加速，大湾区综合性国家科学中心加快谋划建设。

图表：关于2019年国民经济和社会发展计划执行情况与2020年国民经济和社会发展计划草案的报告（图3） 新华社发

二是新动能加速培育。启动国家数字经济创新发展试验区建设。统筹推进重大信息化工程建设，实施一批“十三五”重大政务信息化工程。战略性新兴产业集群发展工程深入实施。

三是创新创业创造活力持续增强。开展科研项目经费使用“包干制”和“绿色通道”改革试点。成功举办2019年全国双创活动周。截至2019年底，全国高新技术企业超过22.5万家，科技型中小企业超过15.1万家，分别增长约24%和15%。我国创新指数世界排名提升至第14位，企业数量日均净增1万户以上。

（五）落实落细重大战略，城乡区域发展协调性不断增强。推动乡村振兴和区域发展重大战略落地见效，着力提升新型城镇化质量，努力缩小城乡区域发展差距。

一是乡村振兴战略加快实施。乡村振兴战略规划确定的重大工程、重大计划和重大行动启动实施。乡村旅游、休闲农业等新业态不断涌现。持续推进农药化肥减量增效，加快推进农作物秸秆、畜禽粪污资源化利用。农村水电路等条件显著改善，农业农村污染治理攻坚战全面展开，农村人居环境整治加快推进。乡村文化建设和乡村治理深入推进。

二是新型城镇化质量稳步提高。建立健全城乡融合发展体制机制的政策措施印发实施。1000多万农业转移人口落户城镇，1亿非户籍人口在城市落户工作取得重大进展，全国常住人口城镇化率达60.60%，户籍人口城镇化率达44.38%。中心城市和城市群人口集聚能力逐步提升，都市圈建设有序推进，特大镇设市取得突破，特色小镇发展进一步规范。

三是区域协调发展新机制加快构建。支持西部大开发、东北振兴、中部崛起、东部率先的政策体系更加完善。京津冀协同发展有力有序推进，雄安新区转入施工建设阶段。长江经济带生态环境突出问题整改和生态环境污染治理成效显著。粤港澳大湾区建设规划政策体系进一步完善。长三角区域一体化发展规划纲要印发实施，生态绿色一体化发展示范区启动建设。黄河流域生态保护和高质量发展规划纲要启动编制。老少边贫等特殊类型地区加快振兴发展，对口支援有力推进。海洋经济发展示范区建设全面启动。

图表：关于2019年国民经济和社会发展计划执行情况与2020年国民经济和社会发展计划草案的报告（图4） 新华社发

图表：关于2019年国民经济和社会发展计划执行情况与2020年国民经济和社会发展计划草案的报告（专栏2） 新华社发

（六）全面推进市场化改革，市场主体活力进一步激发。坚持和完善社会主义基本经济制度，重点领域、关键环节改革全面推进。

一是营商环境持续优化。“放管服”改革向纵深推进，《优化营商环境条例》颁布，构建中国营商环境评价体系，在全国41个城市开展营商环境评价。印发市场准入负面清单（2019年版），清单事项压减至131项。规范投资审批行为，投资审批“一网通办”水平不断提高。工程建设项目审批环节继续精简。部门联合“双随机、一公开”监管逐步推行，“互联网+监管”事项清单加快制定，全国信用信息共享平台功能和服务不断完善，以信用为基础的新型监管机制加快构建。全国一体化政务服务平台上线试运行，金融信用信息数据库服务网络覆盖全国。

二是混合所有制改革、产权保护、激发保护企业家精神等改革联动推进。第四批160户混合所有制改革试点启动实施。加强产权和知识产权保护，甄别纠正涉产权冤错案件取得重要突破，涉政府产权纠纷问题专项治理行动有序开展。出台支持民营企业改革发展、促进中小企业健康发展的政策措施，建立健全企业家参与涉企政策制定机制。

图表：关于2019年国民经济和社会发展计划执行情况与2020年国民经济和社会发展计划草案的报告（专栏3） 新华社发

三是要素市场化配置改革加快落地。制定促进劳动力和人才社会性流动体制机制改革意见。修订证券法，设立科创板并试点注册制，改革完善贷款市场报价利率（LPR）形成机制。扩大高校和科研院所科研相关自主权，开展赋予科研人员职务科技成果所有权或长期使用权试点，构建技术转移服务体系。修订土地管理法、城市房地产管理法，建设用地使用权转让、出租、抵押二级市场进一步完善，工业用地多方式出让和市场供应体系更加健全。

四是重点领域改革深入推进。国家石油天然气管网集团有限公司组建成立。全面放开石油天然气上游勘查开采准入。出台教育、科技、交通运输等领域中央与地方财政事权和支出责任划分改革方案。电力交易市场化程度进一步提高，增量配电业务改革和电力现货市场建设试点稳步推进。深化燃煤发电上网电价形成机制改革，风电和光伏上网电价改革、农业水价综合改革、交通运输价格改革有序推进。改制成立中国国家铁路集团有限公司和中国邮政集团有限公司。行业协会商会与行政机关脱钩改革全面推开。

图表：关于2019年国民经济和社会发展计划执行情况与2020年国民经济和社会发展计划草案的报告（专栏4） 新华社发

（七）持续深化高水平开放，开放型经济新格局加快构建。对外开放向更大范围、更宽领域、更深层次全面推进，国际经济合作和竞争新优势加快培育。

一是共建“一带一路”扎实推进。第二届“一带一路”国际合作高峰论坛成功举办。累计与138个国家和30个国际组织签署200份共建“一带一路”合作文件。中巴经济走廊等建设进展顺利。雅万高铁、中老铁路、瓜达尔港等重大项目取得积极进展，中俄东线天然气管道投产通气。与法国、英国、日本等14个国家建立第三方市场合作机制。“一带一路”绿色发展国际联盟正式启动，“数字丝绸之路”“丝路电商”建设合作有序推进。中欧班列累计开行突破2万列，通达欧洲18个国家的57个城市，综合重箱率达94%。西部陆海新通道总体规划印发实施。

二是外贸发展稳中提质。推进贸易高质量发展的指导意见印发实施。第二届中国国际进口博览会成功举办。区域全面经济伙伴关系协定（RCEP）谈判取得重大进展。对欧盟、东盟以及“一带一路”沿线国家进出口保持较快增长，集成电路等高附加值产品及知识密集型服务出口保持快速增长。新设24个跨境电商综合试验区，市场采购贸易方式试点、服务贸易创新发展试点扎实推进，综合保税区等海关特殊监管区域发展质量进一步提升。

三是利用外资水平持续提升。外商投资法及实施条例出台实施。进一步扩大鼓励外商投资范围，鼓励外资投向制造业和生产性服务业，支持中西部地区承接外资产业转移。全国和自贸试验区外资准入负面清单分别缩减至40条、37条。放宽在华外资金融机构持股比例和业务范围限制，扩大金融市场双向开放。推进国家级经济技术开发区创新提升。完善企业外债备案登记管理，加强重点行业外债风险防范。全年实际使用外商直接投资1381亿美元。

四是自贸试验区建设取得积极成效。增设上海自贸试验区临港新片区，新设山东等6个自贸试验区，实现沿海省份全覆盖，并首次在沿边地区布局。向全国复制推广自贸试验区49项制度创新成果，累计复制推广223项。海南自由贸易港建设加快推进。

五是境外投资平稳发展。实施企业境外经营合规管理指引，中国装备、技术、标准和服务稳步“走出去”，全年非金融类境外直接投资1106亿美元。人民币跨境融资渠道逐步拓宽，人民币国际化有序推进。

（八）着力保障和改善民生，人民群众获得感幸福感安全感不断提升。坚持以人民为中心的发展思想，保障人民群众尤其是困难群众的基本生活，让各项惠民举措落到实处。

一是稳就业促增收力度加大。落实就业优先政策，出台实施进一步做好稳就业工作意见。强化重点群体和就业困难人员帮扶，全方位完善公共就业服务，拓展劳动者流动就业空间。全年使用失业保险基金向115万户企业发放稳岗返还552亿元，惠及职工7290万人；向126万人次失业保险参保职工发放失业保险技能提升补贴20亿元，从失业保险基金结余中拿出逾1000亿元支持职业技能提升行动，超额完成补贴职业技能培训1500万人次以上的目标任务。深入推行终身职业技能培训制度，职业技能公共实训基地建设持续加强。多措并举促进居民增收，努力增加劳动者特别是一线劳动者劳动报酬，居民人均可支配收入超过3万元。

二是社会保障体系进一步完善。基本养老保险覆盖人数达9.68亿人，企业职工基本养老保险基金中央调剂比例从3%提高到3.5%，退休人员基本养老金稳步提高。划转部分国有资本充实社保基金工作全面推开。城乡居民医疗保险和大病保险制度更趋完善，医疗保障扶贫、医保药品目录调整等工作扎实推进。通过工伤保险为194万工伤职工及供养亲属提供待遇保障。全年有461.2万失业人员领取到不同期限的失业保险金，平均每人每月1393元，保障水平稳步提高。稳步推进低保制度城乡统筹，健全低保标准动态调整机制，全面实施特困人员救助供养制度。推进城镇老旧小区改造，城镇棚户区改造开工316万套，大中城市住房租赁市场加快培育。

三是公共服务补短板强弱项提质量深入推进。国家财政性教育经费支出占国内生产总值比例继续超过4%，对困难地区和薄弱环节教育投入力度不断加大，九年义务教育巩固率达94.8%，高中阶段教育毛入学率达89.5%，高等教育毛入学率超过50%，高职院校扩招100万人目标顺利完成，分层分类开展国家产教融合建设试点。健康中国行动启动实施，区域医疗中心建设试点稳步开展，药品集中采购和使用试点积极推进，促进中医药传承创新发展的意见印发实施。推进养老服务发展的政策体系更加完善。妇女儿童权益保障工作不断加强，规范托育机构设置和管理，促进3岁以下婴幼儿照护服务发展。食品、药品等重点领域监管不断加强。基本公共文化服务均等化水平不断提高。长城、大运河、长征国家文化公园建设统筹推进。加大对全民健身中心等项目建设支持力度，冬奥会场馆建设有序推进。国家积极应对人口老龄化中长期规划印发实施。年末总人口达14.0005亿人，人口自然增长率3.34‰。

图表：关于2019年国民经济和社会发展计划执行情况与2020年国民经济和社会发展计划草案的报告（专栏5） 新华社发

从2019年经济社会发展情况看，经济增长、就业、物价、国际收支等主要指标以及创新驱动、资源节约、环境保护、民生保障等领域指标完成情况良好。约束性指标中，受钢铁、建材、有色、化工等行业生产以及服务业保持较快增长等多种因素影响，单位国内生产总值能耗降低指标实际下降2.6%，低于3%左右的计划目标，但该指标2016－2019年的累计降幅已完成“十三五”规划目标任务的87.1%，符合进度要求。预期性指标中，第一产业增加值、社会消费品零售总额、一般公共预算收入和城镇居民人均可支配收入指标的实际运行值与预期值存在一定差距。第一产业增加值预期目标为“增长3.5%左右”，实际增长3.1%，主要原因是猪肉等一些农产品产量出现较大幅度下降，猪肉产量下降21.3%。社会消费品零售总额预期目标为“增长9.0%左右”，实际增长8.0%，主要原因是汽车、家电等传统消费增速放缓，其他消费新增长点尚在培育中。一般公共预算收入预期目标为“增长5.0%”，实际增长3.8%，主要原因是为支持实体经济发展，减税降费规模超过预期。城镇居民人均可支配收入增速预期目标为“与经济增长基本同步”，实际增长5.0%，低于国内生产总值6.1%的增速，主要原因是受城镇居民经营净收入和财产净收入增速有所放缓、物价涨幅高于上年等因素影响。

各位代表：

今年以来，突如其来的新冠肺炎疫情对我国经济社会发展带来巨大冲击。这次疫情是新中国成立以来我国遭遇的传播速度最快、感染范围最广、防控难度最大的公共卫生事件。面对疫情带来的严峻考验，习近平总书记亲自指挥、亲自部署，迅速成立中央应对疫情工作领导小组，向湖北派出中央指导组，充分发挥国务院联防联控机制作用，始终把人民群众生命安全和身体健康放在第一位。在以习近平同志为核心的党中央坚强领导下，各地区、各部门认真贯彻党中央、国务院决策部署，全国上下众志成城、守望相助，按照坚定信心、同舟共济、科学防治、精准施策的总要求，迅速打响了疫情防控的人民战争、总体战、阻击战。

全力以赴做好疫情防控救治工作。举全国之力支援湖北省、武汉市主战场，组织4万多名医护人员驰援，迅速建成火神山、雷神山等集中收治医院和方舱医院，着力提高收治率和治愈率、降低感染率和病亡率。开展联防联控和群防群控，各省（区、市）相继启动重大突发卫生事件一级响应，组织干部力量下沉抓好社区防控，引导各类社会组织、专业社会工作者和志愿服务力量依法有序参与疫情防控和社会服务。扎实做好医疗物资保障和生活必需品保供稳价工作，快速实现口罩等医疗防护物资、医疗救治设备、医治床位从严重短缺到基本满足疫情防控需要；千方百计保障粮油与肉禽蛋菜奶等食品的市场供应和价格基本稳定。强化疫情防控科技攻关，短时间内研发快速检测试剂并大规模应用，坚持中西医结合，坚持“四集中”，尽最大可能挽救更多患者生命。针对境外疫情扩散蔓延，加强输入性风险防控，做好对境外我国公民关心关爱，开辟临时航线有序接回我国在外困难人员，积极推进疫情防控国际合作，加强与世界卫生组织合作，向出现疫情扩散的国家和地区提供力所能及的帮助。

统筹推进疫情防控和经济社会发展工作。分区分级有序推动复工复产复市复业。在有效防控疫情的前提下，积极帮助解决企业用工、物流、资金和原材料、零部件供应问题，全力保障城乡道路、公共交通畅通，加强对中小微企业和个体工商户的应急纾困服务，推动产业链上下游、大中小企业协同复工复产。截至4月底，规模以上工业企业复工率、员工到岗率分别达到99.4%和94.3%。加强投资项目远程审批服务，做好用工、土地、资金、用能、环评等方面保障，及时帮助解决重大项目复工遇到的堵点难点问题。及时推出一系列对冲纾困政策。在去年已提前下达2020年地方政府专项债券额度1.29万亿元的基础上，进一步增加提前下达限额1万亿元。加大减税降费力度，阶段性减免小规模纳税人增值税，阶段性减免企业社保费、缓缴住房公积金，降低企业用电用气成本等，一季度减税降费规模超过7000亿元。实施3次普遍降准、定向降准，新增1.8万亿元再贷款再贴现额度，进一步强化对中小微企业的普惠性金融支持措施。对中小微企业贷款实施延期还本付息。出台促进消费扩容提质加快形成强大国内市场的政策措施，延长新能源汽车购置补贴和免征车辆购置税政策年限。同时，及时解读形势和政策，积极回应社会关切，稳定市场主体发展信心。抓好春季农业生产。保障种子、化肥、农药、农机等生产供应，春耕春播进展顺利，早稻播种面积预计达7100万亩，冬小麦、冬油菜长势良好，着力解决畜牧水产养殖饲料供应和部分农产品滞销等困难。凝心聚力推进脱贫攻坚。加快推进易地扶贫搬迁安置区配套设施、扶贫车间、农村饮水安全、以工代赈等脱贫攻坚项目开工复工，积极挖掘公益岗位潜力，促进贫困人口就地就近就业，优先组织贫困劳动力返城返岗和外出务工，对因疫情返贫致贫人员及时落实帮扶措施。强化保居民就业和民生兜底。充分发挥失业保险基金援企稳岗作用，扩大国有企业招收、硕士研究生招生、专升本、大学生应征入伍数量规模，通过“点对点”运输等方式引导农民工有序返岗复工。组织全国大中小学有序开展线上教学。对受疫情影响的困难群众实施救助帮扶，及时启动社会救助和保障标准与物价上涨挂钩联动机制，阶段性提高补贴标准、扩大覆盖范围。

经过全党全国上下艰苦卓绝的努力，武汉保卫战、湖北保卫战取得决定性成果，疫情防控阻击战取得重大战略成果，统筹推进疫情防控和经济社会发展工作取得积极成效。实践再次证明，中国共产党领导和我国社会主义制度、我国国家治理体系具有强大生命力和显著优越性，能够战胜任何艰难险阻，能够为人类文明进步作出重大贡献。

同时，我们也清醒认识到，尽管我国疫情得到有效控制，但境外疫情快速扩散蔓延，世界经济出现严重衰退，不确定不稳定因素显著增多，我国经济运行面临巨大挑战。从国际看，疫情对全球产业链供应链形成较大冲击，国际金融市场剧烈震荡，单边主义、贸易保护主义抬头，国际经贸规则面临挑战，地缘政治风险仍然较高，这些都会加大我国发展的外部风险。从国内看，境外疫情输入压力加大，经济发展特别是产业链供应链恢复面临新的挑战。一是内外需求下降导致经济循环受阻。居民非必需品消费等因疫情冲击受到严重抑制，汽车等大宗商品消费大幅下滑，消费增长受到制约。由于企业经营困难加大、订单减少，加之地方投资能力受限，投资增长面临较大困难。受外需持续疲弱和全球供应链中断影响，稳外贸稳外资压力明显加大。二是行业企业运行困难较多。企业利润大幅下降，特别是生活性服务业受疫情冲击最大，不少中小微企业出现资金链断裂、停产关闭等情况，存在局部地区、部分行业中小微企业破产增多的可能。三是公共卫生和应急体系短板凸显。疾病预防控制管理体系不完善，新发传染病监测预警和应对能力不足，县镇村公共卫生基础设施相对薄弱，重要物资国家储备体系尚不健全。四是科技创新能力短板仍较突出。研发投入强度与建设创新型国家的要求相比尚需提高，有利于科技创新的深层次制度障碍还没有破除，关键核心技术受制于人的状况尚未根本改观。五是重点领域改革仍需加力。由市场决定要素价格机制仍有待健全。需持续在国企国资、财税金融、营商环境、民营经济、扩大内需、城乡融合等重点改革领域攻坚克难。六是重点领域风险有所集聚。外部输入性风险上升，信用违约风险可能加大，部分中小金融机构风险较高。受疫情影响，各级财政防控支出增加、税收收入减少，一些地方基层财政运转困难。七是社会民生领域面临较大挑战。稳岗稳就业难度加大，高校毕业生等重点群体就业压力明显攀升，一些地方失业再就业难度加大。部分劳动者工资收入降低，居民增收难度加大。剩余贫困人口脱贫任务艰巨，深度贫困地区实现“三保障”面临不少新的挑战。生态环境治理仍然存在短板和薄弱环节。养老、托育、教育等公共服务体系与人民群众期待相比还存在明显差距。同时，我们在工作中也还存在一些不足，比如，疫情防控政策协调协同还不够，有的政策存在碎片化、条块化问题，有的政策措施落实还不够到位，有的领域治理手段和治理能力有待优化和加强，工作中还存在形式主义、官僚主义等问题。

在看到困难和挑战的同时，我们更要看到，疫情的冲击和影响都是阶段性的、总体可控的，改变不了我国发展潜力大、韧性强、经济长期向好的基本面。危与机同生并存，克服了危即是机。在以习近平同志为核心的党中央坚强领导下，有中国特色社会主义制度的显著优势，有强大的动员能力和雄厚的综合实力，有全党全军全国各族人民的团结奋斗，我们一定能够战胜这场疫情，也一定能够保持我国经济社会良好发展势头，确保完成决战决胜脱贫攻坚的目标任务，全面建成小康社会。

二、2020年经济社会发展总体要求、主要目标和政策取向

2020年是全面建成小康社会和“十三五”规划收官之年，要实现第一个百年奋斗目标，为“十四五”发展和实现第二个百年奋斗目标打好基础，做好经济社会发展工作十分重要。

（一）总体要求。

要在以习近平同志为核心的党中央坚强领导下，以习近平新时代中国特色社会主义思想为指导，全面贯彻党的十九大和十九届二中、三中、四中全会精神，坚决贯彻党的基本理论、基本路线、基本方略，增强“四个意识”、坚定“四个自信”、做到“两个维护”，紧扣全面建成小康社会目标任务，统筹推进疫情防控和经济社会发展工作，在疫情防控常态化前提下，坚持稳中求进工作总基调，坚持新发展理念，坚持以供给侧结构性改革为主线，坚持以改革开放为动力推动高质量发展，坚决打好三大攻坚战，加大“六稳”工作力度，保居民就业、保基本民生、保市场主体、保粮食能源安全、保产业链供应链稳定、保基层运转，坚定实施扩大内需战略，维护经济发展和社会稳定大局，确保完成决战决胜脱贫攻坚目标任务，全面建成小康社会。

具体工作中，必须增强底线思维，做好应对各种复杂局面的思想准备和工作准备，深刻认识“六稳”和“六保”是内在统一的，“六保”是今年“六稳”工作的着力点，守住“保”的底线，稳住经济基本盘，进而以保促稳、稳中求进，就能为全面建成小康社会夯实基础。要围绕“六保”加大宏观政策实施力度，落实好已出台应急纾困政策，根据形势变化及时推出新的对冲政策，兜住民生底线。同时，必须紧盯主要目标，讲究策略方法，做到应对外部风险和克服内部挑战相结合、解决当前问题和化解长期矛盾相结合、有效市场和有为政府相结合、尽力而为和量力而行相结合，善于化危为机，确保党中央、国务院决策部署落到实处、见到实效。

（二）主要预期目标。

按照上述总体要求和工作思路，本着尊重经济规律和实事求是的原则，综合考虑国际与国内、当前与长远、需要与可能，提出2020年经济社会发展主要预期目标：

——优先稳就业保民生，坚决打赢脱贫攻坚战，努力实现全面建成小康社会目标任务。主要考虑：全球疫情和经贸形势不确定性很大，我国发展面临一些难以预料的影响因素，不对经济增长目标作量化要求，有利于引导各方面继续认真贯彻落实党中央、国务院决策部署，针对当前面临的主要矛盾、困难、问题，将工作重心放在落实“六保”任务和推动经济高质量发展上。同时，今年不提经济增速量化指标，并不意味着不要经济增长，保居民就业、保基本民生，完成脱贫攻坚既定目标任务、实现全面建成小康社会，以及防范化解各类风险，都要有经济增长作为支撑。

——城镇新增就业900万人以上，全国城镇调查失业率和城镇登记失业率分别为6%左右和5.5%左右。主要考虑：关于城镇新增就业总量，受疫情冲击和经济下行压力加大影响，城镇新增就业压力明显攀升，但考虑到新成长劳动力在城镇就业的需求量，保持相当规模的就业增量是必要的、也是必须的，也体现就业优先政策导向。关于失业率，尽管疫情对就业影响还会持续一段时间，但我们有信心有能力防范化解大规模失业风险，做好稳就业工作。

——居民消费价格涨幅为3.5%左右。主要考虑：2019年居民消费价格上涨的翘尾影响较大，受疫情影响，2020年还有一些新的涨价因素，价格总体上涨压力仍然较大；同时，3.5%左右的预期目标也兼顾了稳定市场预期的需要。

——居民收入增长与经济增长基本同步。主要考虑：与全面建成小康社会的目标要求相衔接，更好满足人民日益增长的美好生活需要，更好体现以人民为中心的发展思想。同时，随着乡村振兴战略深入实施、促进居民增收的政策措施逐步落地，2020年实现居民收入增长预期目标是有支撑的。

——进出口促稳提质，国际收支基本平衡。主要考虑：2020年外贸增长难度较大，既要努力促进外贸稳定发展、稳定市场预期和企业信心，也要加快培育外贸竞争新优势，推动外贸高质量发展。

——单位国内生产总值能耗和主要污染物排放量继续下降，努力完成“十三五”规划目标任务。主要考虑：虽然到2019年底已完成“十三五”规划确定的单位国内生产总值能耗降低15%目标任务的87.1%，但今年任务仍然十分繁重，特别是疫情对经济增速的影响可能大于对能源消费总量增速的影响，完成规划目标任务需要付出艰苦努力。

（三）主要政策取向。

为实现上述目标，需要充分估计疫情对世界经济的冲击影响，在精准施策的前提下，加大宏观政策调节和实施力度，加强宏观政策协调配合，着力稳经济保民生，防止短期冲击演变成趋势性变化。

——积极的财政政策要更加积极有为。2020年财政赤字率拟按3.6%以上安排，财政赤字规模比去年增加1万亿元；中央财政发行抗疫特别国债1万亿元，不计入财政赤字。上述两项措施新增的资金全部给地方，主要用于保居民就业、保基本民生、保市场主体以及支持减税降费、减租降息、扩大消费和投资等。大力优化财政支出结构，坚决压减一般性支出，严禁新建楼堂馆所，严禁铺张浪费，同时保持扶贫、义务教育、基本养老、基本医疗、城乡低保等民生支出只增不减。统筹用好中央财政以前年度结转资金，用于支持完成全面建成小康社会任务、克服疫情对经济的影响、“十四五”规划重点项目等。对于财政受疫情影响较大的地方，加大转移支付力度，保基本民生、保工资、保运转。

——稳健的货币政策要更加灵活适度。引导广义货币供应量（M2）和社会融资规模存量增速明显高于去年。综合运用公开市场操作、降息、降准、再贷款、再贴现等工具，保持流动性合理充裕，引导贷款利率进一步下行，带动企业综合融资成本明显下降。延长中小微企业贷款延期还本付息政策，对普惠小微贷款努力做到应延尽延，对部分困难较大的大中型企业协商延期。创新直达实体经济的货币政策工具，提供应急融资支持。引导金融机构加大对中小微企业的信贷投放力度，鼓励发放普惠小微信用贷款。保持人民币汇率在合理均衡水平上的基本稳定，保持外汇储备规模合理适度。做好金融稳定和风险处置工作，守住不发生系统性风险的底线。

——就业优先政策要全面强化。将保居民就业摆在突出位置，根据就业形势变化调整政策力度，稳定就业总量，改善就业结构，提升就业质量。加大援企稳岗力度，因地因企因人分类帮扶，着力防范化解规模失业风险。更好发挥大众创业万众创新对保居民就业的支撑作用，探索相关支持政策鼓励高校毕业生创新创业。引导灵活就业、新就业形态健康发展。强化对高校毕业生、农民工、退役军人等重点群体帮扶，实施部分职业资格“先上岗、再考证”阶段性措施，用好职业技能提升行动专账资金，加强对就业困难人员特别是贫困劳动力的就业援助，确保零就业家庭动态清零。

——加强宏观政策的统筹协调。加强政策预研储备，促进财政、货币、就业政策与消费、投资、产业、区域等政策协同发力、形成合力。坚定实施扩大内需战略，充分挖掘超大规模市场潜力，发挥消费的基础作用和投资的关键作用，引导资金投向供需共同受益、具有乘数效应的领域，推动国内需求稳步扩大。产业政策要围绕推动制造业高质量发展，坚持政府引导和市场机制相结合，坚持独立自主和开放合作相促进，着力促进产业基础高级化、产业链现代化。强化竞争政策的基础性地位，创造公平竞争制度环境，进一步激发市场活力。区域政策要充分发挥各地区比较优势，以重大项目、重大政策为牵引推动重大区域战略落地，加快形成带动全国高质量发展的新动力源。

三、2020年国民经济和社会发展计划的主要任务

2020年，要按照党中央、国务院决策部署，统筹推进疫情防控和经济社会发展，着力做好九方面工作。

（一）继续巩固和拓展疫情防控阶段性成效，坚决做好疫情常态化防控。全面落实“外防输入、内防反弹”的总体防控策略，及时采取有针对性和实效性的防控措施，继续抓紧抓实抓细各项防控工作。

一是坚决巩固国内防控成果。健全及时发现、快速处置、精准管控、有效救治的常态化防控机制，完善并及时启动相关防控预案，科学防范，堵住漏洞。全面筑牢社区防控网底，加强信息化、智能化防控能力。继续全力做好疑似和确诊患者的救治工作，继续做好康复患者、隔离群众和抗疫一线医务人员的心理疏导。加快推进疫苗研发。

二是加强和完善外防输入举措。继续加强对境外我国公民的关心关爱。坚决守住守好口岸城市防线，优化医疗资源和救治力量布局，支持边境地区公共卫生设施建设，加强检疫检测能力建设，快速精准识别和管控风险源风险点。加强集中隔离人员安全服务保障，做好健康监测和人文关怀。

三是深入推进疫情防控国际合作。同世界卫生组织深化交流合作，探索建立区域公共卫生应急联络机制，继续向有关国家和地区提供力所能及的帮助，推进健康丝绸之路建设，以多种方式为国际防疫合作贡献力量。严把防疫物资出口质量关。加强科研攻关国际合作，争取在特效药物研发和疫苗研制等方面尽早取得突破。

（二）坚决打好三大攻坚战，确保如期完成各项目标任务。确保脱贫攻坚任务如期全面完成，确保实现污染防治攻坚战阶段性目标，确保不发生系统性金融风险。

一是决战决胜脱贫攻坚。落实好中央统筹、省负总责、市县抓落实的工作机制，继续聚焦“三区三州”等深度贫困地区，对未摘帽贫困县和贫困村实施挂牌督战，确保现行标准下农村贫困人口全部脱贫、贫困县全部摘帽。加大就业扶贫力度，优先组织贫困劳动力返岗务工。加快脱贫攻坚项目开工复工进度，鼓励企业更多招用贫困地区特别是建档立卡贫困家庭人员，支持扶贫龙头企业、扶贫车间吸纳当地就业。深入开展消费扶贫，组织好产销对接，解决疫情等造成的贫困地区农产品卖难问题。加大产业扶贫力度，用好产业帮扶资金和扶贫小额信贷政策，允许地方今年酌情调整优化财政专项扶贫资金政策，加大产业扶贫项目奖补力度。加快易地扶贫搬迁、农村饮水安全脱贫攻坚“扫尾”工程开复工和建设，确保上半年基本完成所有扫尾工作，细化落实易地扶贫搬迁后续扶持措施，补齐大型安置区配套教育、医疗设施等领域的短板。加强贫困地区公共服务基础设施建设。深入推进教育扶贫攻坚，巩固深化控辍保学工作成果。持续推进农村危房改造，完成贫困户住房安全有保障的目标任务。扩大以工代赈建设领域、实施范围和受益对象。做好因疫致贫返贫人口的帮扶，确保基本生活不受影响。对没有劳动能力的特殊贫困人口要强化社会保障兜底，实现应保尽保。深化定点扶贫。支持革命老区脱贫攻坚和振兴发展。继续执行对摘帽县的主要扶持政策。研究建立解决相对贫困的长效机制。

二是持续加强污染防治。坚持方向不变、力度不减，抓好源头防控，推动生态环境质量持续好转，加快构建现代环境治理体系。巩固蓝天、碧水、净土保卫战成果。有序推进北方地区冬季清洁取暖长效机制建设，扩大钢铁行业超低排放改造规模，加强柴油货车污染治理，加大铁路专用线建设力度，持续推进货运“公转铁”。地级及以上城市空气质量优良天数比率确保完成“十三五”规划目标。进一步加强城镇污水处理、城市黑臭水体治理、渤海综合治理、农业农村污染治理、水源地保护，推动实施一批长江、黄河生态环境保护重大工程。扎实推进土壤污染防治，推进受污染耕地和污染地块安全利用。编制全国重要生态系统保护和修复重大工程专项建设规划，完善河长制湖长制，加快推动落实林长制，持续推动山水林田湖草系统治理，推进地下水超采综合治理。万元国内生产总值用水量下降1.2%。强化生态保护监管，推动健全生态保护红线监管标准体系和配套政策。健全生态补偿机制，起草生态保护补偿条例，推进生态综合补偿试点。深化国家生态文明试验区建设。积极应对全球气候变化，加快全国碳市场建设。发展绿色产业，发布绿色技术推广目录，支持园区环境污染第三方治理。加快补齐城镇污水垃圾处置和医疗废物、危险废物收集处理等环境基础设施短板。全面推进垃圾分类，研究治理快递包装污染及商品过度包装，提高城市生活垃圾无害化处理率。

三是守住不发生系统性风险的底线。密切关注全球金融市场和大宗商品市场震荡可能带来的输入性风险，完善跨境资金流动的预警响应机制。跟踪监测杠杆率较高、盈利能力较弱的行业企业的流动性风险，防止资金链断裂引发连锁反应。继续有序处置高风险金融机构风险。积极维护债券市场稳定，加快健全市场化、法治化的债券违约处置机制，建立早识别、早预警、早发现、早处置风险防控体系。落实银行不良贷款核销政策，鼓励市场主体参与不良贷款处置。完善金融机构恢复与处置机制，支持银行多渠道补充资本。稳妥化解地方政府存量隐性债务。坚持“房子是用来住的、不是用来炒的”定位，稳步建立健全因城施策的促进房地产市场平稳健康发展的长效机制，着力稳地价、稳房价、稳预期。

（三）坚定不移实施扩大内需战略，着力促进形成强大国内市场。聚焦疫情带来的冲击和影响，深入挖掘和激发国内市场供给与需求潜力，增强内需对经济增长的拉动力。

一是释放消费潜力。在防控措施到位前提下，有序推动各类商场、市场复市复业，促进线下消费加速回补。培育壮大线上消费，推进互联网和各类消费业态深度融合。促进教育、医疗、养老、家政、文旅、体育等服务消费线上线下融合发展，进一步支持依托互联网的外卖配送、网约车等新业态。发展大健康产业。扩大电商、快递进农村覆盖面，畅通工业品下乡、农产品进城渠道。加速5G网络建设和场景应用，完善新型基础设施布局，推动超高清视频、虚拟现实等新兴消费。积极扩大汽车消费，允许进口尾气排放实测结果达到国六标准的平行进口汽车。对京津冀等重点地区淘汰国三及以下排放标准柴油货车给予适当奖补。加大汽车、绿色智能家电等消费金融支持。鼓励有条件的地区实施家电以旧换新补贴政策。加快废旧家电回收体系建设。扩大智能产品、定制化产品和绿色产品供给，促进绿色消费发展。培育建设国际消费中心城市，因地制宜形成若干区域消费中心。积极发展“智慧商圈”，加快建设新型文旅商业消费聚集区。加快恢复乡村旅游和休闲农业。鼓励夜间经济发展。有序放宽低空空域限制，加快通用航空网络建设，推动通用航空发展。加强国家物流枢纽、国家骨干冷链物流基地和商品流通网络建设，推动冷链物流、智慧物流、国际物流、供应链发展。推进电子商务与快递物流协同发展。深入推进步行街改造提升。强化消费信用体系建设，大力整治伪劣商品、侵权仿冒等问题，加大农村商品流通市场秩序监管力度，健全消费维权法律制度和政策体系，营造安全放心消费环境。

二是积极扩大有效投资。安排中央预算内投资6000亿元，比上年增加224亿元。全年安排地方政府专项债券3.75万亿元，比上年增加1.6万亿元。按照“资金跟着项目走”要求，安排好地方政府专项债券项目，集中支持有一定收益的基础设施和公共服务项目。加强公共卫生、生物安全、应急物资保障、物资和能源储备、物流设施、农林水利、城乡基础设施等领域补短板。出台推动新型基础设施建设的相关政策文件，推进5G、物联网、车联网、工业互联网、人工智能、一体化大数据中心等新型基础设施投资。做好城镇老旧小区改造，重点改造提升小区水电气路信等配套便民设施，有条件的加装电梯、配建停车设施。推动城市更新，加大城市停车场和充电设施、城乡污水垃圾处理设施、采煤沉陷区综合治理、独立工矿区和城区老工业区搬迁改造等建设力度。稳步推进棚户区改造。实施好“十三五”规划165项重大工程项目和国家重大区域战略重大项目。加大沿江高铁、沿海高铁项目建设力度。积极推动川藏铁路开工，加快重点城市群、都市圈城际和市域（郊）铁路规划建设。加快推进在建重大水利工程建设进度，新开工一批重大水利工程。加快推进枢纽机场新建、迁建和改扩建项目建设，有序推进支线机场和通用机场建设。加快推进国家高速公路、普通国省干线、农村公路和内河水运等项目建设。坚持“要素跟着项目走”，加强项目用地用海用能等要素保障，对重大投资项目开设绿色通道。继续加大金融支持，发挥政府资金的导向作用，规范创新政府和社会资本合作（PPP）模式，支持民间资本参与补短板和新型基础设施建设。稳妥推进基础设施领域不动产投资信托基金（REITs）试点，充分调动包括民间投资在内的各类社会资本积极性，盘活存量资产。

三是着力稳定社会预期。完善疫情信息发布机制，继续做到依法、公开、透明、及时、准确。加大对传染病防治法的宣传教育，多层次、高密度发布权威信息，更好维护社会大局稳定。提高政策稳定性和可预期性，健全政策目标管理和重大决策公开机制，不断提高政策透明度。促进部门之间、中央与地方间的政策联动，加强对政策方向、目标、时序、边界的协调。进一步加强预期管理，创新宣传手段，做好信息发布和政策解读，及时回应社会关切，稳定市场主体信心。

（四）深入推进供给侧结构性改革，稳定提升产业链供应链水平。大力振兴实体经济，持续提升产业基础能力和产业链现代化水平，保持我国产业链供应链的稳定性和竞争力。

一是加大对各类企业特别是中小微企业和个体工商户支持力度。简化各项助企纾困政策手续，政府涉企事项尽可能网上办理。加大中介服务收费清理整顿力度，坚决制止各种涉企违规收费。推动降低企业用能、用网、物流、房租等成本，将除高耗能以外的大工业和一般工商业电价降低5%政策延长到今年年底，宽带和专线平均资费降低15%。减免国有房产租金，鼓励各类业主减免或缓收房租。限期完成清偿政府机构、国有企业拖欠民营企业中小企业账款的任务。引导银行向实体经济让利，鼓励商业银行对受疫情冲击较大的地区企业和批发零售、住宿餐饮、物流运输、文化娱乐旅游和外贸等行业企业阶段性减免一定比例贷款利息。优化创业担保贷款贴息政策，提高小微企业信用贷款、续贷业务发放比例，加大首次贷款支持，大型商业银行普惠型小微企业贷款增速要高于40%。支持金融机构发行3000亿元小微金融债券以发放小微贷款。引导公司信用类债券净融资比上年多增1万亿元。促进小微企业全年应收账款融资8000亿元。鼓励资本市场加大对应对疫情科技型中小企业的融资支持。鼓励大企业运用获得的融资，以预付款形式向上下游中小微企业支付现金。出台支持制造业民营企业稳增长和转型升级的意见，鼓励地方以市场化手段对陷入困境的优质民营企业进行纾困救助。

二是推动制造业高质量发展。加大制造业中长期贷款支持力度，重点支持高端装备制造、传统产业改造提升、电子信息制造等重点领域。实施好制造业核心竞争力提升工程，完善技术改造服务体系。实施智能制造工程，研究出台推进制造业智能化改造的指导意见。积极推进钢铁企业“推重组、促转型”，持续优化石化产业布局，继续实施人口密集区危化品生产企业搬迁改造。实施绿色制造工程。推广钢结构装配式建筑，推动智能制造与建筑工业化融合发展。支持老工业城市和资源型城市产业转型升级示范区建设。开展先进制造业集群培育试点示范。

三是保产业链供应链稳定。着力完善和畅通物流运输网络，支持企业增加关键物料备货。实施产业基础再造工程。大力培育产业生态主导型企业，培育一批有国际竞争力的先进制造业集群，增强产业链的集聚效应。培育一批“专精特新”中小企业。研究开展供应链安全风险评估，优化生产力布局，增加应急储备，进一步增强供应链韧性和稳定性。深入实施工业互联网创新发展工程，打造工业互联网平台体系，强化工业互联网平台间标准对接。推动先进制造业和现代服务业融合发展，大力发展服务型制造。加快推动生产性服务业公共服务平台建设，发展研发设计、现代物流、检验检测认证等生产性服务业。

四是着力培育壮大新动能。深入推进国家战略性新兴产业集群发展工程，加强创新和公共服务综合体建设。创新战略性新兴产业金融产品和服务供给。深入推进“上云用数赋智”，实施数字化转型伙伴行动、中小企业数字化赋能专项行动和数字经济新业态培育行动，深入推进数字经济创新发展试验区建设，推动制造、商贸流通等经济社会重点领域数字化转型，发展数字商务，支撑建设数字供应链。深入实施国家大数据战略、“互联网+”行动，推动新型智慧城市建设，推进5G深度应用。大力发展生物经济，加强生物资源开发与利用，培育若干生物集聚发展高地。加快智能制造、无人配送、在线消费、医疗健康、机器人等新兴产业发展。支持商业航天发展，延伸航天产业链条，扩展通信、导航、遥感等卫星应用。制定国家氢能产业发展战略规划。支持新能源汽车、储能产业发展，推动智能汽车创新发展战略实施。加快深远海捕捞养殖装备创新发展，建设现代化海洋牧场。

图表：关于2019年国民经济和社会发展计划执行情况与2020年国民经济和社会发展计划草案的报告（专栏6） 新华社发

五是着力保障粮食安全和农副产品市场供应。深化农业供给侧结构性改革，压实粮食安全省长责任制、“菜篮子”市长负责制。编制新一轮国家粮食安全中长期规划纲要、新时期农业生产力布局与结构调整规划，制定落实全球疫情影响下确保我国粮食安全的应对方案，加快推动出台粮食安全保障法，深入推进优质粮食工程，继续实施大豆振兴计划，增加绿色优质粮油产品供给，促进农民增收、企业增效、消费者得实惠。扎实开展高标准农田建设，实施东北黑土地保护性耕作行动计划，实施现代种业和动植物保护能力提升工程，加强病虫害监测和防控。完善农机补贴政策。深入推进农村科技创新，加强农业科技人才培养，推动各类农业科研院所和高校联合协作加快培养高层次农业人才，强化粮食安全科技支撑。创新完善粮食“产购储加销”协同联动体系，加强粮食市场监测预警和监管，完善粮食储备安全管理体制，积极破解夏粮仓容紧张矛盾。抓好农业保险保费补贴政策落实。落实小麦、稻谷最低收购价政策，完善玉米、大豆市场化收购加补贴机制，落实棉花目标价格政策。持续抓好生猪生产，持续加强非洲猪瘟、高致病性禽流感等重大动物疫病监测和防控，促进畜牧水产养殖业提质发展，加快推进奶业振兴。实施农产品仓储保鲜冷链物流设施建设工程，支持农村电商发展。推进重要农产品进口多元布局，做好居民生活必需品保供调度，保障粮油肉蛋果蔬等重要农产品质量安全、稳定供应。

六是着力保障能源安全。稳步推进煤炭、石油、天然气和电力产供储销体系和石油储备基地建设，加强煤电油气运行调节。健全国内外供需形势发生重大变化的应对预案，确保供需总体平衡和市场平稳运行。继续做好油气勘探开发工作，加快推进油气矿业权区块竞争出让。加快油气管网和储备工程建设，健全油气管网运营机制，推动管网设施公平开放。着力推动煤电改造升级，积极稳妥发展水电，安全发展先进核电，保持风电光伏发电合理发展，推动非化石能源成为增量主体。健全可再生能源电力消纳利用长效机制，积极推进就地就近消纳新模式。有序建设跨省跨区输电通道，提升能源系统输送和调节能力。持续推进电力应急体系建设，提高快速响应能力。推动电力交易机构独立规范运行，建设全国统一电力市场，全面放开经营性电力用户发用电计划，推动增量配电改革试点落地见效。深化电网企业装备制造、设计施工等竞争性业务改革。

（五）坚定实施创新驱动发展战略，强化科技创新对经济发展支撑作用。充分发挥创新第一动力、人才第一资源作用，大力弘扬科学精神和工匠精神，营造劳动光荣的社会风尚和精益求精的敬业风气，加快建设创新型国家。

一是加快关键核心技术攻关。构建社会主义市场经济条件下关键核心技术攻关新型举国体制。开展科技重大专项梯次接续有关工作，加快组织实施科技创新2030－重大项目。支持构建面向全行业国产化应用的基础支撑平台。健全鼓励支持基础研究、原始创新的体制机制，发展市场化的新型研发机构，鼓励企业等社会力量增加投入。加快提升企业技术创新能力，研究将国有企业技术创新情况纳入考核机制。加快推进首台（套）重大技术装备、紧缺新材料研制和关键共性技术平台建设。完善支持创新产品应用的政府采购政策。综合多学科力量开展科研攻关，加强新冠肺炎传染源、传播致病机理等研究，加大药品、疫苗研发力度和应急生产能力建设。夯实生物安全基础设施建设，统筹做好生物安全科技攻关工作。加快完善国家技术安全管理清单制度。

二是加强创新能力建设。编制新一轮国家中长期科技发展规划和国家重大科技基础设施建设规划。加强基础研究和原始创新、颠覆性技术创新，加快组建国家实验室，重组国家重点实验室体系，加快建设跨学科、大协作、高强度的协同创新基础平台。推动科技创新中心和综合性国家科学中心建设，科学布局建设重大科技基础设施、科教基础设施和国家产业创新中心、工程研究中心、技术创新中心、制造业创新中心、供应链科创中心。实行重点项目攻关“揭榜挂帅”。改革科技成果转化机制，畅通创新链，营造鼓励创新宽容失败的科研环境。制定支持重大专项成果产业化配套政策，完善创新人才培养支持机制和政策，健全人才分类评价体系。提升国家自主创新示范区和高新区创新发展水平。

三是推进创新创业创造高质量发展。实施新一轮全面创新改革试验。建立健全以企业为主体、市场为导向、产学研深度融合的技术创新体系，支持大中小企业和各类主体融通创新。新建一批双创示范基地，实施一批双创支撑平台项目。实施示范基地“校企行”专项行动。加快推进创业投资领域的体制机制改革。研究加大对孵化器、众创空间等创新创业服务机构的政策支持力度。加大对受疫情影响企业帮扶力度，扩大创业担保贷款对象范围，对优质项目免除反担保要求，加大对重点群体创业场地支持力度。

（六）持续推进市场化改革，进一步激发经济发展活力动力。抓紧出台进一步深化改革举措，着力破解制约发展的体制机制障碍，坚持和完善社会主义基本经济制度。

一是深化“放管服”改革。全面落实《优化营商环境条例》，总结推广疫情防控期间各地高效服务企业的经验做法。完善营商环境评价指标体系和评价方法，在全国部分地级及以上城市、国家级新区定期开展中国营商环境评价。推动各地开通企业开办“一网通办”平台，进一步简化审批和登记手续，实现企业开办全程网上办理。放宽小微企业、个体工商户登记经营场所限制，便利各类创业者注册经营，及时享受扶持政策。深入推进“证照分离”改革。进一步压减中央和地方层面设定的行政许可以及备案、登记、年检、认定等事项。深入开展投资审批制度改革，建立健全投资审批清单制度，完善投资项目统一代码制度。推行并联审批，谋划承诺制改革，进一步提高项目落地效率。以“多规合一”为基础，深化规划用地“多审合一”“多证合一”改革，推进“多测合一”“多验合一”。加快推进政府采购法和招标投标法修订工作。在市场监管领域实现相关部门“双随机、一公开”监管全覆盖。促进公共资源交易市场健康有序发展，全面实施电子化交易。深入推进“互联网+政务服务”，加快打造全国政务服务“一张网”，进一步压减办税事项、纳税时间，整合各类动产登记和权利担保登记系统，不断提升政务服务效能。大力推行信用承诺制度，深入开展“信易贷”工作，提升社会信用体系建设法治化、规范化水平。基本完成行业协会商会与行政机关脱钩改革。

二是建设高标准市场体系。实施新时代加快完善社会主义市场经济体制的意见。制定实施高标准市场体系建设行动方案。构建更加完善的要素市场化配置体制机制，开展要素市场化配置综合改革试点。全面实施市场准入负面清单制度，以服务业为重点开展放宽市场准入试点，探索实施市场准入负面清单许可事项审批在线监管和综合评估。推动地方建立市场准入隐性壁垒台账，重点破除民营企业特别是中小微企业准入障碍。研究制定竞争政策的指导意见，修订完善公平竞争审查制度实施细则。强化对各类产权平等保护的法律规定。健全支持民营经济发展的法治环境，保护民营企业、民营企业家合法财产权和经营权。加强和改进反垄断和反不正当竞争执法，强化企业商业秘密保护。建立健全知识产权侵权惩罚性赔偿制度，加大知识产权执法力度，加强专利转化和应用。

图表：关于2019年国民经济和社会发展计划执行情况与2020年国民经济和社会发展计划草案的报告（专栏7） 新华社发

三是加快国资国企和重点行业改革。制定实施国企改革三年行动方案，加快推动国有经济布局优化和结构调整，健全现代企业制度，着力形成以管资本为主的国有资产监管体制。积极深化混合所有制改革，做深做实四批试点，制定出台深化国有企业混合所有制改革的实施意见。推动市场化债转股增量扩面提质，引导金融资产投资公司参与推进传统产业市场化重组。加快推进国有企业退休人员社会化管理，基本完成剥离办社会职能和解决历史遗留问题。

四是深化财税、金融、价格体制改革。扎实推进中央与地方财政事权和支出责任划分改革，健全地方税体系，逐步后移消费税征收环节并稳步下划地方。探索运用零基预算理念，打破支出固化僵化格局。进一步提高国有资本经营预算调入一般公共预算比例。深化金融供给侧结构性改革，优化融资结构和金融机构体系、市场体系、产品体系，有序推进存量浮动利率贷款定价基准转换。出台创业板改革并试点注册制总体实施方案，继续提高直接融资特别是股权融资比例。完成第二监管周期输配电价改革，深化公用事业价格改革，清理规范城镇供水、供电、供气、供暖行业收费，落实燃煤发电价格形成机制改革，鼓励有条件的地方探索推行城镇生活垃圾处理差别化收费政策，继续深化电价、水价改革。完善油气管网价格形成机制。

（七）统筹推进城乡区域协调发展，培育壮大高质量发展动力源。发挥各地比较优势，推动乡村振兴战略和新型城镇化战略落地见效。

一是落实落细重大区域发展战略。落实区域协调发展新机制，持续推进西部大开发形成新格局，支持东北地区全面振兴全方位振兴，健全中部地区崛起政策体系和工作机制，继续推动东部地区率先发展。纵深推进京津冀协同发展，加快实施雄安新区重点建设项目，完善北京非首都功能疏解政策体系。大力推进长江经济带生态环境突出问题整改和系统性保护修复，加快构建综合交通运输体系，促进内河船舶改造。推动粤港澳大湾区基础设施互联互通，有序开展国际科技创新中心建设，加快横琴、河套等重大合作平台建设。高标准建设长三角生态绿色一体化示范区，推动长三角交通一体化更高质量发展，建设虹桥国际开放枢纽。抓紧做好黄河流域生态保护和高质量发展规划纲要编制和出台实施工作。大力支持成渝地区双城经济圈建设，打造全国高质量发展的重要增长极。积极推进西部陆海新通道建设。深化对口支援。支持少数民族和民族地区加快发展。推动国家级新区、开发区等功能平台创新提升发展，加快推进资源型地区经济转型和老工业城市更新改造。推动山西开展能源革命综合改革试点。继续推进承接产业转移示范区建设，在具备条件的内陆城市打造一批高端制造业基地。制定发展海洋经济加快建设海洋强国的意见。完善主体功能区战略和制度，编制《全国国土空间规划纲要（2021－2035）》。

图表：关于2019年国民经济和社会发展计划执行情况与2020年国民经济和社会发展计划草案的报告（专栏8） 新华社发

实施好支持湖北省经济社会发展的一揽子政策，支持保就业、保民生、保运转和产业链供应链稳定运行，促进湖北省经济社会运行正常秩序全面恢复。

二是推进以人为核心的新型城镇化建设。提高农业转移人口市民化质量，进一步完善配套政策，推动完成1亿非户籍人口在城市落户目标。引导超特大城市提升高端要素配置能力和国际竞争力，加快向外疏解非核心功能。推动省会和地级市等大中城市优化城市功能，补短板强弱项，改善公共服务品质，提升宜居宜业水平。加快推进县城新型城镇化建设，弥补环境卫生、市政公用、公共服务、产业配套等领域短板弱项，以适应农民日益增加的到县城就业安家需求。推动长江中游、中原、关中平原、山东半岛、北部湾等城市群重点领域发展，启动一批重点都市圈交通设施互联互通项目，支持城市群和中心城市提高综合承载和资源优化配置能力。提升城市治理现代化水平，增强城市韧性，推进新型智慧城市建设，打造城市数据大脑。推动城乡融合发展体制机制和政策体系落实落地，引导国家城乡融合发展试验区改革探索。

三是压茬推进乡村振兴战略巩固脱贫成效。深入推进农村一二三产业融合发展，支持农产品加工园区建设，加快建设现代农业产业园，建设一批农村产业融合发展示范园、农业产业强镇和中国特色农产品优势区，培育一批乡村旅游重点村，创新发展具有民族和地域特色的乡村休闲旅游业、手工业等特色产业，加快发展乡村新型服务业，做大做强农业品牌。完善乡村产业发展用地保障政策。增强集体经济实力。加强现代农业设施建设，如期完成大中型灌区续建配套与节水改造。继续推进农村电网升级改造和农村道路建设，加快农村地区宽带网络和移动通信网络覆盖。以疫情防治为切入点，深入开展普遍性村庄清洁行动。因地制宜推进农村厕所革命，强化生活垃圾和污水处理，完成农村人居环境整治三年行动任务。探索建立农业绿色发展支撑体系，加强农业面源污染监测和治理。做好县域村庄空间布局规划，加快编制多规合一的实用性村庄规划。启动农村水系综合整治试点县建设。推动农村能源发展，加快推进农业废弃物能源化资源化利用。深入开展乡村治理体系建设试点示范。

（八）加快建设更高水平开放型经济新体制，推动对外开放取得新进展。积极应对疫情全球蔓延的不利影响，高质量推进共建“一带一路”，稳住外贸外资基本盘。

一是保持进出口基本稳定。大力开拓多元化市场，支持企业通过网上洽谈、网上办展等方式，促合作、拓市场。支持和鼓励出口企业积极开拓国内市场，积极对冲外需萎缩影响。支持跨境电商贸易、市场采购贸易等外贸新业态新模式加快发展。进一步推动综合保税区高水平开放高质量发展。推动新一轮服务贸易创新发展试点。稳定加工贸易发展。在风险可控前提下，支持扩大短期出口信用保险业务并降低费率，设立中长期政策性出口信用保险专项安排。积极筹办第三届中国国际进口博览会。积极参与国际物流供应链建设，保障国际货运畅通。

二是积极利用外资。全面实施外商投资法及配套法规。大幅缩减外资准入负面清单，深化服务业、金融业、制造业、农业对外开放。做好招商、安商、稳商工作，扩大鼓励外商投资产业目录范围，继续推动一批重大外资项目落地。推广服务业扩大开放试点经验。加强外资企业服务，确保内外资企业同等享受助企纾困政策。加大我投资环境与政策宣介，吸引国际资源要素有序流入。进一步发挥国外贷款、中长期外债等低成本资金作用，防范外债风险。

三是推动共建“一带一路”高质量发展。落实好第二届“一带一路”国际合作高峰论坛成果。深入推进与重点国家及国际组织务实合作，提高风险防控和处置能力。扎实推进与周边国家互联互通，稳步推进中蒙俄、中巴、中国－中南半岛经济走廊等建设。持续推动中欧班列高质量发展，深化数字丝绸之路建设合作。鼓励开展人文交流，夯实共建“一带一路”民意基础。进一步强化国际次区域合作。

四是加快对外开放高地建设。深化经济特区改革开放。加快落实自贸试验区改革试点任务，赋予自贸试验区更大改革自主权，形成更多高质量的可复制可推广制度创新成果。发布实施海南自由贸易港建设总体方案，切实抓好早期政策安排落地。出台海南自由贸易港跨境服务贸易负面清单。在中西部地区增设自贸试验区、综合保税区。提高沿边开发开放水平，支持沿边重点开发开放试验区建设。

五是推动境外投资平稳健康发展。坚持企业主体，完善分类指导，优化境外投资结构。稳步开展国际产能合作，开拓多元化市场，带动装备、技术、服务、品牌、标准“走出去”。健全境外投资政策和服务体系，为设计、咨询、会计、认证、法律等生产性服务业加快国际化发展营造良好环境，便利企业因地制宜建设海外仓。积极拓展第三方市场合作。引导企业健全境外合规管理体系，规范企业境外经营行为，加强境外风险防范。

六是积极参与国际经济治理。坚定维护多边贸易体制，积极参与世界贸易组织（WTO）等多边机构改革。推动实施更高水平自贸区战略，争取区域全面经济伙伴关系协定（RCEP）如期签署，加快推进中日韩等自贸区谈判。

（九）切实做好民生兜底工作，保障人民群众基本生活需要。针对疫情带来的民生问题，通过加大投入、落实政策保障予以有效解决。

一是千方百计稳定就业。加大对受疫情影响的中小企业的帮扶力度，支持企业稳定岗位，规范企业裁员行为。推进公共就业服务城乡常住人口全覆盖，畅通失业登记渠道，支持建立共享用工、就业保障等服务平台，充分发挥经营性人力资源机构等市场化服务力量。加快落实已出台各项招生、入伍扩招计划，国有企业今明两年连续扩大高校毕业生招聘规模。鼓励大学毕业生到基层就业，扩大“三支一扶”计划等基层服务项目和见习实施规模。发挥创业带动就业倍增效应，实施农村创新创业带头人培育行动，支持农民工、高校毕业生和退役军人等人员返乡入乡创业，促进多渠道灵活就业，合理设定流动摊贩经营场所。在公共基础设施建设中，尽可能吸纳农村劳动者就地就近就业。推进公共职业技能培训基础平台共建共享，加大职业技能培训支持力度，对未继续升学的应届初、高中毕业生和青年登记失业人员，动员参加劳动预备制培训。今明两年职业技能培训3500万人次以上，完成三年培训5000万人次目标任务。高职院校扩招200万人。

二是加快补齐公共卫生短板。健全公共卫生服务体系，强化公共卫生法制保障，适当提高基本公共卫生服务经费财政补助标准，加强公共卫生人才队伍建设和基层防控能力建设。改革疾病预防控制体制。完善重大疫情防控体制机制，健全国家公共卫生应急管理体系，全面加强疾控中心能力建设，力争各省都有达到生物安全三级水平的实验室，构建以传染病专科医院和综合医院感染科、呼吸科等为骨干、其他医疗机构为预备队的动员机制，规划布局国家重大疫情救治基地。深化公立医院综合改革。发展“互联网+医疗健康”。提高城乡社区医疗服务能力。推进分级诊疗。构建和谐医患关系。促进中医药振兴发展，完善中医医疗服务体系。进一步推进全民预防保健。深入实施健康中国行动。

三是加快社会保障体系建设。稳步提高基本医保基金统筹层次，推进做实医保地市级统筹，鼓励有条件的地方探索推进省级统筹。建立国家医保药品目录动态调整机制。提高医保基金使用效率，完善总额预算管理，推行符合不同医疗服务特点的医保支付方式，加强定点医药机构绩效考核，深入开展异地就医直接结算。居民医保人均财政补助标准提高30元。完善短缺药品供应保障，进一步深化医疗、医保、医药联动改革。有序扩大国家组织药品集中采购和使用药品品种范围，开展国家组织高值医用耗材集中采购试点。推进失业保险省级统筹，全面实现工伤保险省级统筹。加快推进养老保险全国统筹，实现企业职工基本养老保险基金省级统收统支，适度提高城乡居民基础养老金最低标准。落实好各项社会保险阶段性减免、缓缴和降低费率政策，确保社保待遇按时足额发放。加快划转部分国有资本充实社保基金。加快多层次社会保障体系建设，大力发展企业（职业）年金、补充医疗保险，建立并推动养老保险第三支柱发展。扩大失业保险保障范围，将参保不足1年的农民工等失业人员都纳入常住地保障。扩大低保保障范围，对城乡困难家庭应保尽保，将符合条件的城镇失业和返乡人员及时纳入低保等救助范围。做好因公殉职人员抚恤工作。保障妇女、儿童、老年人、残疾人合法权益，健全农村留守儿童和妇女、老年人关爱服务体系。推进保障性安居工程建设，大力培育和发展租赁住房，加大城市困难群众住房保障工作力度。

四是稳定居民收入增长保障重点群体基本生活。进一步稳定居民财产性收入增长预期，适度扩大国债、地方政府债券面向个人投资者的发行额度。鼓励有条件的地区进一步提高居民转移性收入。统筹推进收入分配重点领域改革，健全工资指导线和企业薪酬调查制度。保障外出农民工在常住地享有与城镇户籍人口同等的就业创业政策，依法保障农民工劳动报酬权益，从源头上防止发生新的农民工工资拖欠，推动进城农民工更好地融入城市。做好重点农产品、重点时段价格调控工作，落实好社会救助和保障标准与物价上涨挂钩联动机制，强化对困难群众的兜底保障。进一步统筹完善城乡社会救助制度，稳步提高城乡低保标准，继续推进落实低保等救助政策与扶贫政策衔接。

五是持续提高公共服务水平。推动国家基本公共服务标准出台，推进基层服务机构标准化试点。稳定教育投入，优化投入结构，缩小城乡、区域、校际差距。巩固城乡义务教育经费保障机制，积极化解城镇义务教育学校大班额，加强乡镇寄宿制学校和乡村小规模学校、县城学校建设，完善随迁子女义务教育入学政策。办好特殊教育、继续教育。多渠道扩大普惠性学前教育资源，支持深度贫困地区优质普惠学前教育资源扩容建设，帮助民办幼儿园纾困。加快实施高中阶段教育普及攻坚，持续改善贫困地区学校基本办学条件。稳步发展普通本科教育和研究生教育。支持中西部高校发展。扩大高校面向农村和贫困地区招生规模。普通高等教育本专科招生920万人，研究生招生111.4万人。大力推进国家产教融合建设试点。加强教师队伍建设。推进教育信息化。积极应对人口老龄化，支持社会力量发展普惠养老、普惠托育服务。繁荣发展哲学社会科学。加强文物保护利用和非物质文化遗产传承。继续实施文化旅游提升工程，积极推动黄河文化保护传承弘扬，加快推进重大文化设施项目建设，推动全域旅游、乡村旅游和红色旅游发展。积极推进海南国际旅游消费中心、横琴国际休闲旅游岛、平潭国际旅游岛等建设。加快推进“互联网+旅游”发展。深入开展“领跑者”行动，全面推进家政服务业提质扩容。推进冬奥会场馆建设。实施应急管理能力标准化建设，加快推动战略应急物资储备安全管理体制机制改革。加强自然灾害监测预警，加快自然灾害防治重大工程实施，做好防灾减灾和救灾工作。加强和创新社会治理，健全社区管理和服务机制，提升基层应急能力。深化公共法律服务体系建设。加强和改进食品、药品安全监管。严格落实安全生产责任制，做好安全生产和隐患排查，坚决防止各类重特大事故灾难发生。

全面准确贯彻“一国两制”、“港人治港”、“澳人治澳”、高度自治的方针，始终坚定支持和推动香港、澳门融入国家发展大局。立足香港作为国际金融、航运、贸易中心的优势，落实好内地与香港建立更紧密经贸关系的安排（CEPA）系列协定。支持澳门推进世界旅游休闲中心、中国与葡语国家商贸合作服务平台等建设。扩大两岸经济文化交流合作，为台胞台企提供更多发展机遇、同等待遇。

今年还有一项重要任务，就是要按照党中央、国务院决策部署，在全面评估“十三五”规划实施情况基础上，广泛征求意见，集聚民智，编制国民经济和社会发展第十四个五年规划《纲要》，统筹推进“十四五”国家级专项规划、区域规划、空间规划、地方规划编制工作，做好“十四五”规划《纲要草案》提交十三届全国人大四次会议审查的准备。

各位代表：

统筹做好2020年疫情防控和经济社会发展工作意义重大，任务艰巨。我们要更加紧密地团结在以习近平同志为核心的党中央周围，高举中国特色社会主义伟大旗帜，以习近平新时代中国特色社会主义思想为指导，增强“四个意识”、坚定“四个自信”、做到“两个维护”，按照党中央、国务院决策部署，自觉接受全国人大的监督，认真听取全国政协的意见和建议，迎难而上、求真务实，主动担当、攻坚克难，在常态化疫情防控中着力抓经济、促生产、拓需求，努力完成全年经济社会发展目标任务，全面建成小康社会，为开启全面建设社会主义现代化国家新征程打下坚实基础。

第十三届全国人民代表大会财政经济委员会关于

2019年国民经济和社会发展计划执行情况与2020年国民经济和社会发展计划草案的审查结果报告

（2020年5月26日第十三届全国人民代表大会第三次会议主席团第二次会议通过）

十三届全国人大三次会议主席团：

第十三届全国人民代表大会第三次会议审查了国务院提出的《关于2019年国民经济和社会发展计划执行情况与2020年国民经济和社会发展计划草案的报告》和2020年国民经济和社会发展计划草案。全国人民代表大会财政经济委员会在对计划报告和计划草案初步审查的基础上，根据各代表团和有关专门委员会的审查意见，又作了进一步审查。国务院根据审查意见对计划报告作了修改。现将审查结果报告如下。

一、2019年计划执行情况总体良好

财政经济委员会认为，2019年国民经济和社会发展计划执行情况总体良好。过去的一年，在以习近平同志为核心的党中央坚强领导下，国务院和地方各级人民政府坚持以习近平新时代中国特色社会主义思想为指导，全面贯彻党的十九大和十九届二中、三中、四中全会精神，按照党中央决策部署和十三届全国人大二次会议各项决议要求，坚持稳中求进工作总基调，深入贯彻新发展理念，坚持以供给侧结构性改革为主线，推动高质量发展，扎实做好“六稳”工作，经济运行总体平稳，发展质量稳步提升。十三届全国人大二次会议批准的2019年国民经济和社会发展计划中绝大多数约束性指标和预期性指标完成情况符合或好于预期。各项重点任务扎实推进，供给侧结构性改革继续深化，三大攻坚战取得关键进展，改革开放迈出新步伐，民生和社会事业不断发展，为全面建成小康社会打下决定性基础。

今年以来，面对新冠肺炎疫情带来的严峻考验，习近平总书记亲自指挥、亲自部署，坚持把人民群众生命安全和身体健康放在第一位。在以习近平同志为核心的党中央坚强领导下，全党全军全国各族人民万众一心、众志成城，迅速打响疫情防控的人民战争、总体战、阻击战。经过艰苦卓绝的努力，武汉保卫战、湖北保卫战取得决定性成果，疫情防控阻击战取得重大战略成果；多项应急纾困政策及时推出，复工复产复市复业复学有序推进；统筹推进疫情防控和经济社会发展工作取得积极成效。

同时也要看到，当前全球疫情和经济形势严峻复杂，我国发展面临风险挑战前所未有。世界经济严重衰退，国际贸易投资萎缩，单边主义、贸易保护主义抬头，全球产业链供应链循环受阻，不确定性加大。国内经济特别是产业链供应链受到冲击，消费、投资、出口下滑，企业特别是中小微企业困难凸显，就业压力明显加大，部分领域风险隐患有所积聚，重点领域改革仍需加力，公共卫生和应急体系短板凸显。同时，计划执行中还存在一些不足，有的政策存在碎片化、条块化问题，有的政策措施落实还不到位，个别指标没有完成年度计划目标。要切实落实党中央决策部署，采取更加积极有效的措施加以解决。

二、2020年计划报告和计划草案总体可行

财政经济委员会认为，国务院提出的2020年计划报告和计划草案以习近平新时代中国特色社会主义思想为指导，符合中央经济工作会议精神和“十三五”规划纲要要求，充分考虑全球疫情和经贸形势的不确定性，本着尊重经济社会发展规律的原则，综合考虑国际与国内、当前与长远、需要与可能，进一步聚焦决战决胜脱贫攻坚和全面建成小康社会目标任务，体现了统筹推进疫情防控和经济社会发展工作要求，主要预期目标和政策取向符合实际、总体可行，有利于引导各方面把思想和行动统一到落实党中央的决策部署上来，奋力实现第一个百年奋斗目标。建议第十三届全国人民代表大会第三次会议批准国务院提出的《关于2019年国民经济和社会发展计划执行情况与2020年国民经济和社会发展计划草案的报告》，批准2020年国民经济和社会发展计划草案。

三、做好2020年计划执行工作的建议

2020年是全面建成小康社会和“十三五”规划收官之年，要实现第一个百年奋斗目标，为“十四五”发展和实现第二个百年奋斗目标打好基础，做好经济社会发展工作意义重大。要在以习近平同志为核心的党中央坚强领导下，以习近平新时代中国特色社会主义思想为指导，全面贯彻党的十九大和十九届二中、三中、四中全会精神，坚决贯彻党的基本理论、基本路线、基本方略，增强“四个意识”、坚定“四个自信”、做到“两个维护”，紧扣全面建成小康社会目标任务，统筹推进疫情防控和经济社会发展工作，在疫情防控常态化前提下，坚持稳中求进工作总基调，坚持新发展理念，坚持以供给侧结构性改革为主线，坚持以改革开放为动力推动高质量发展，坚决打好三大攻坚战，加大“六稳”工作力度，保居民就业、保基本民生、保市场主体、保粮食能源安全、保产业链供应链稳定、保基层运转，坚定实施扩大内需战略，维护经济发展和社会稳定大局，确保完成决战决胜脱贫攻坚目标任务，全面建成小康社会。为此，财政经济委员会提出以下建议：

（一）着力巩固和拓展疫情防控成果，统筹推进疫情防控和经济社会发展。坚决贯彻党中央决策部署，按照“外防输入、内防反弹”的总体防控策略，坚持做好输入性疫情防控；毫不放松常态化疫情防控，坚决防止疫情反弹。围绕疫情暴露出的问题补短板、堵漏洞、强弱项，加强公共卫生基础设施建设，完善疾病预防控制体系，健全重大疫情、公共卫生应急管理和救治体系，加快推进相关疫苗和药品研发，强化公共卫生法治保障，切实提高应对处置突发重大公共卫生事件的能力和水平。推进市场主体有序复工复产复市复业，实施好支持湖北省经济社会发展的一揽子政策，努力将疫情造成的损失降到最低。

（二）坚决打赢三大攻坚战，助力全面建成小康社会。脱贫攻坚要进一步加大对“三区三州”等深度贫困地区扶持力度，确保现行标准下农村贫困人口全部脱贫、贫困县全部摘帽，做好因疫情致贫或返贫帮扶工作。建立完善反贫困长效机制。污染防治要坚持方向不变、力度不减，突出依法、科学、精准治污，抓好源头防控和结构性减排，推动生态环境质量持续好转，加快构建现代环境治理体系。防范化解重大风险要密切关注输入性风险，稳妥化解地方政府隐性债务风险，有序处置高风险金融机构风险，促进房地产市场平稳健康发展，坚决守住不发生系统性风险的底线。

（三）坚定实施扩大内需战略，保持经济平稳健康发展。加强宏观政策的统筹协调、精准操作和实施力度，积极的财政政策要更加积极有为，稳健的货币政策要更加灵活适度，就业优先政策要全面强化。积极引导和稳定市场主体预期。充分发掘超大规模市场潜力，发挥消费的基础作用和投资的关键作用，促进产业和消费“双升级”。稳定居民收入增长，培育壮大新兴消费增长点，促进消费业态融合，提高居民消费意愿和能力。积极扩大有效投资，加强补短板项目建设，发挥好政府资金的引导作用，激发民间投资活力。

（四）持续深化供给侧结构性改革，着力提升产业链供应链稳定性和竞争力。夯实农业基础，保障粮食安全和农副产品市场供应，促进农民增收。实施产业基础再造和产业链提升工程，巩固传统产业优势，强化优势产业领先地位，提升产业基础高级化和产业链现代化水平。推动生产性服务业向专业化、高品质和多样化升级。畅通产业循环、市场循环、经济社会循环，加强国际协调合作，共同维护产业链供应链安全稳定，提升其竞争力。加强基础研究和科技创新，增加全社会研发投入，加强知识产权保护，强化关键环节、关键领域、关键产品保障能力。

（五）加大对各类企业特别是中小微企业支持力度，千方百计稳定就业。用好用足各种援企稳岗政策，加大对企业特别是中小微企业和个体工商户的扶持力度，帮助渡过难关。切实落实减免或延缴税费和基金、财政补贴、加大政府采购力度等惠企政策，巩固和拓展减税降费成效，降低运营成本。增加信贷投放，提高企业融资便利性，降低融资成本。继续做好清理拖欠企业账款工作。千方百计稳定就业，支持企业稳定岗位，规范裁员行为，突出抓好重点群体就业工作，多措并举扩大各类用人需求，促进多渠道就业，努力提高就业质量。

（六）加快落实区域发展战略，完善区域政策和空间布局。推动西部大开发形成新格局，支持东北地区全面振兴全方位振兴，推动中部地区崛起，支持东部地区率先发展。深入推进京津冀协同发展、长三角一体化发展、粤港澳大湾区建设，构建引领全国高质量发展的动力源。落实长江经济带共抓大保护措施，推动黄河流域生态保护和高质量发展，大力支持成渝地区双城经济圈建设。推动中西部地区和东北地区有序承接国内外产业转移，有力推进产业优化升级。协调推进乡村振兴和新型城镇化战略，促进城乡融合发展。发挥各地区比较优势，形成主体功能明显、优势互补、高质量发展的区域经济布局。

（七）进一步全面深化改革，扩大高水平对外开放。着力破除制约高质量发展的体制机制障碍，增强市场主体信心和活力。完善要素市场化配置体制机制，切实发挥市场在资源配置中的决定性作用。继续深化“放管服”改革，不断优化营商环境。深化国资国企改革，优化国有经济布局。对各类产权平等保护，健全支持民营经济发展的法治环境。深化金融供给侧结构性改革，提高金融服务实体经济能力和水平。推进海南自由贸易港建设，加快建设更高水平开放型经济新体制，开拓多元化进出口市场。高质量共建“一带一路”。积极参与全球经济治理。

（八）强化民生兜底保障，推动社会事业全面发展。进一步落实以人民为中心的发展思想，加大基本民生保障力度。强化困难群体基本生活保障，扩大失业保险保障范围，落实好社会保险阶段性减免等政策，并保障待遇按时足额发放。加快养老保险全国统筹步伐，稳步提高基本医保基金统筹层次。构建多层次社会保障体系，覆盖各类困难群体。推动教育公平发展和质量提升。积极发展养老、托幼服务。加强和完善食品、药品安全监管，做好防灾减灾救灾和安全生产工作，保障人民群众生命财产安全。

今年，在努力实现“十三五”规划目标任务的基础上，要按照党中央决策部署，做好“十四五”规划《纲要》编制工作。

以上报告，请予审议。

第十三届全国人民代表大会财政经济委员会

2020年5月26日
