# Supplementary material for: Mapping the evolution of fertility support policies in China: A content and instrumental analysis
Source: PLoS One. 2025 Oct 9;20(10):e0332137. doi: 10.1371/journal.pone.0332137 (PMC12510515; doi:10.1371/journal.pone.0332137)
Supplement: S1 Appendix — (ZIP) [file pone.0332137.s001.zip › S1 Appendix. 226 original policy documents/39-国家卫生健康委办公厅关于印发托育综合服务中心建设指南(试行)的通知.docx]

**国家卫生健康委办公厅关于印发托育综合服务中心建设指南（试行）的通知**

各省、自治区、直辖市及新疆生产建设兵团卫生健康委：

为贯彻落实《国家发展改革委民政部国家卫生健康委关于印发〈“十四五”积极应对人口老龄化工程和托育建设实施方案〉的通知》（发改社会〔2021〕895号），指导地方做好公办托育服务能力建设项目申报工作，我委组织制定了《托育综合服务中心建设指南（试行）》（可从国家卫生健康委网站下载）。现印发给你们，请遵照执行。

附件：《托育综合服务中心建设指南（试行）》

国家卫生健康委办公厅

2021年12月30日

（信息公开形式：主动公开）

**附件**

托育综合服务中心建设指南

（试行）

目 录

编制说明 1
第一章  总则 2
第二章  项目构成与建设规模 3
第三章  选址与规划布局 6
第四章  建筑与建筑设备 8
第五章  相关指标 13

**编制说明**

根据《国务院办公厅关于促进3岁以下婴幼儿照护服务发展的指导意见》（国办发〔2019〕15号）《国家发展改革委民政部国家卫生健康委关于印发〈“十四五”积极应对人口老龄化工程和托育建设实施方案〉的通知》（发改社会〔2021〕895号）等有关文件要求，依据国家卫生健康委《托育机构设置标准（试行）》《托育机构管理规范（试行）》及相关法律法规标准规范，编制本指南。

本指南主要包括总则、项目构成与建设规模、选址与规划布局、建筑与建筑设备、相关指标等，为托育综合服务中心的建设提供技术指导。

本指南适用于托育综合服务中心的新建、改建和扩建工程项目。

**第一章 总则**

一、统筹规划，科学布局

托育综合服务中心的建设，应当综合考虑城乡区域发展特点，根据经济社会发展水平、本地工作基础和3岁以下婴幼儿家庭需求，优化资源配置，统筹设施数量、规模和布局。

二、规范建设，示范引领

托育综合服务中心的建设，应发挥示范引领、带动辐射作用，为托育服务机构高质量建设提供技术支撑及样板标杆，努力做到规模适度、功能完善、环境安全、装备适宜、经济合理。

三、创新机制，多方协作

托育综合服务中心的建设，积极争取地市级及以上政府发挥支持引导作用，充分调动社会力量积极参与，建立健全项目立项、建设、运营等机制，为托育服务健康发展提供综合保障。

**第二章 项目构成与建设规模**

一、托育综合服务中心建设项目由场地、房屋建筑和建筑设备组成。

（一）场地包括建筑占地、道路、室外活动场地、绿地等；

（二）房屋建筑包括托育服务用房、托育从业人员培训用房、托育产品研发和标准设计用房、婴幼儿早期发展用房、监督管理用房和设备辅助用房等；

（三）建筑设备包括给排水系统、暖通空调系统、电气系统、智能化系统及电梯等。

二、托育综合服务中心建筑面积宜为3000m^2^以上。根据项目建设的实际情况和具体要求，可调整相应建筑面积。

三、托育综合服务中心的托位数可根据当地实际需要设置相应的托位，原则上建设托位规模在150个以内为宜，可相应设置乳儿班（6～12个月，10人以下）、托小班（12～24个月，15人以下）、托大班（24～36个月，20人以下）三种班型。18个月以上的婴幼儿可混合编班，每个班不超过18人。

四、托育服务用房主要包括婴幼儿活动用房、服务管理用房和附属用房等，每托位建筑面积不应少于12m^2^。托育服务用房参照此部分用房相关标准和规范执行。

在托育机构建设标准正式发布前，公办托育服务机构可参照此部分用房进行建设。

各类用房主要包括以下内容：

（一）婴幼儿活动用房包括但不限于班级活动单元和综合活动室；班级活动单元包括睡眠区、活动区、配餐区、清洁区、卫生间、储藏区等；

（二）服务管理用房包括但不限于晨检接待厅、保健观察室、隔离室、母婴室、警卫室、办公室、财务室、会议室、储藏室等；

（三）附属用房包括但不限于设备机房、开水间、餐食准备区、卫生间、清洁间、车库等。

五、托育从业人员培训用房可包括实训室、培训室、教师办公室等，并可根据需要设置绘画室、手工室、辅食制作室、讨论室、报告厅、教研室、远程示教室等。托育从业人员培训用房总建筑面积宜为1000m^2^～2000m^2^，可按10m^2^/学员（同期学员数量）计算。

六、托育产品研发和标准设计用房可根据研发业务需要设置，可包括研发室、标准设计室、教具制作室、从业人员培训教材编写室、绘本创作室、影音制作室、模拟体验室、产品展示厅等，建筑面积宜为600m^2^～800m^2^。鼓励相邻城市或区域共建共享，集中进行产品研发和标准设计。

七、婴幼儿早期发展用房可包括养育照护指导室、早期发展指导室、营养膳食指导室、婴幼儿情景体验区、互联网家长课堂、工作人员办公室等，建筑面积宜为1000m^2^～1200m^2^。

八、监督管理用房可根据协助监管相关业务需要设置，可包括监控管理室、信息机房、资料存储室、办公室等，建筑面积宜为400m^2^～600m^2^。

九、设备辅助用房包括变配电室、空调机房、进排风机房、消防水泵房、给水泵房、智能化系统机房、车库等。车库建筑面积应根据所在地区的相关要求确定并另行增加。

**第三章 选址与规划布局**

一、托育综合服务中心的选址应符合城乡总体发展规划要求，结合人口发展、群众需求等因素，合理布点，保障安全。

二、托育综合服务中心的选址应满足以下要求：

（一）宜交通便利、环境安静、符合卫生和环保要求；

（二）宜远离对婴幼儿成长有危害的建筑、设施及污染源；

（三）应具有较好的工程地质条件和水文地质条件；

（四）周边应有便利的供水、供电、排水、通信及市政道路等公用基础设施；

（五）宜有良好的自然通风和采光条件。

三、托育综合服务中心宜独立设置。当与其它建筑合并设置时，宜设置在低层区域，自成一区，并应设置独立的出入口。

四、托育综合服务中心主入口不宜直接设在城市主干道或过境公路干道一侧，机构外宜设置人流缓冲区和安全警示标志，独立园区周围宜设置围墙。

五、托育综合服务中心的规划布局应功能分区明确、方便管理、节约用地。

六、托育综合服务中心应设置婴幼儿室外活动场地。室外活动场地面积每托位宜为2m^2^～5m^2^，宜有良好的日照和通风条件，并应设置安全防护设施。当与其他设施共用活动场地时，应考虑共用时的安全防护措施，并方便照护。

七、托育综合服务中心停车宜符合当地有关规定。场地内设汽车库（场）时，应与婴幼儿室外活动场地分开，并宜设置家长接送临时停车区域。

八、托育综合服务中心绿化用地宜符合当地有关规定。绿化用地面积每托位不宜低于1.5m^2^～3m^2^，绿地中严禁种植有毒、有刺、有飞絮、病虫害多、有刺激性的植物。

**第四章 建筑与建筑设备**

一、托育综合服务中心的建设，应贯彻安全、适用、经济、节能、环保的原则，应功能完善、分区明确，托育服务用房应适合婴幼儿身心健康发展。

二、托育服务用房应为独立区域，宜有良好朝向；托育从业人员培训用房、托育产品研发和标准设计用房、婴幼儿早期发展用房及监督管理用房宜自成一区。

三、托育服务用房应设置在二层及以下部分，应设独立出入口，婴幼儿活动用房不应设在地下室、半地下室，应满足婴幼儿生活、活动等功能需要。

四、托育服务用房的室内装修和设施应符合下列规定：

（一）入口晨检接待厅应宽敞明亮，有利于人流集散通行，宜设置家长等候区、婴儿车存放区。

（二）每个婴幼儿应有一张床位，不应设双层床，床侧不宜紧靠外墙布置；睡眠和活动区合并设置的，应设置床位的收纳空间。

（三）婴幼儿活动区域的室内房间高度和走廊宽度应符合婴幼儿活动和照护的要求，楼梯扶手、栏杆、踏步高度和宽度应满足婴幼儿使用、保护婴幼儿安全的要求。

（四）婴幼儿卫生间宜临近活动区或睡眠区设置，宜分间或分隔设置；卫生间不宜设置台阶，应设婴儿护理台和婴儿冲洗设施；托小班和托大班宜设适合幼儿使用的卫生器具，每班宜设2～4个大便器、2～3个小便器、3～5个适合幼儿使用的洗手池或盥洗台水龙头，便器之间宜设隔断；可结合适合需求设置成人卫生间。

（五）母婴室宜临近婴幼儿生活空间，宜设尿布台、洗手池等设施。

（六）隔离室宜设置独立卫生间，具有良好通风。

（七）餐食准备区宜相对独立，与婴幼儿活动用房宜有一定距离。

五、婴幼儿活动区域应满足以下要求：

（一）宜设双扇平开门，不应设置弹簧门、推拉门、旋转门，不宜设置门槛，宜设置门扇固定装置。门应设置观察窗，采用安全玻璃。

（二）婴幼儿活动区域宜采用柔性、易清洁的楼地面材料；有水房间地面应采用防滑材料；墙面宜选用环保、耐久、易清洁和美观的材料；宜选用吸声降噪材料，并适合婴幼儿心理特点的色彩；内墙阳角、柱子及窗台宜做成小圆角。

（三）婴幼儿活动区域窗台距楼地面不宜高于0.6m，当窗台面距楼地面高度低于0.9m时，应采取防护措施，防护高度应从可踏部位顶面起算，不应低于0.9m。

（四）婴幼儿活动区家具宜适合婴幼儿尺度、防蹬踏，边缘宜做成小圆角，桌椅和玩具柜等家具表面及婴幼儿手指可触及的隐蔽处，均不得有锐利的棱角、毛刺及小五金部件的锐利尖端。

（五）婴幼儿活动用房应有直接天然采光，并应满足相应的日照要求。卫生间、未设外窗的房间等宜设置通风设施。

六、托育从业人员培训用房应满足以下要求：

（一）实训室应按照睡眠、活动、饮食、如厕等婴幼儿活动内容分设不同的区域，在每个区域配置不同的家具和相应设施。

（二）主要培训用房室内采光宜均匀明亮，采光应符合建筑采光设计标准的要求，严禁使用有色玻璃，并应防止眩光。

七、托育产品研发和标准设计用房应满足以下要求：

（一）影音制作用房应有相应的隔声措施，满足影音制作要求，并避免对周边用房的干扰；

（二）标准设计用房应有标准化教具、器材展示、存放的空间；

（三）产生噪声的教具制作用房应相对独立，并有良好的隔声措施。

八、婴幼儿早期发展用房应满足以下要求：

（一）宜设置相对独立的出入口和等候区、婴儿车存放区等。

（二）应按照活动类别，动静分区：咨询室、评估室、指导室、工作人员办公室宜设置在“静区”，婴幼儿情景体验区、多功能活动室、多媒体教室、亲子课堂宜设置在“动区”。

（三）宜结合成人卫生间设置婴幼儿卫生设施，或设置独立的婴幼儿卫生间。

（四）应设置母婴室，使用面积不应低于10m^2^；母婴室应设置洗手盆、婴儿尿布台及桌椅等必要的家具。可与托育服务用房母婴室合并使用。

（五）灯具的选择和照度应满足各区域活动要求，并应防止眩光。

九、监督管理用房应满足以下要求：

（一）监督管理用房应相对独立、自成一区，宜设置在相对安静的区域，机房不应布置在用水区域的正下方，宜避免设置在顶层。

（二）监控管理室、信息机房的设备布置应满足机房管理、人员操作和安全、设备散热、安装维护要求；宜采用防静电架空地面；新建项目净高应根据机柜高度及通风要求确定。

（三）监控管理室、信息机房的温度、相对湿度应满足电子信息设备的使用要求。

十、托育综合服务中心的抗震、消防应符合现行国家相关标准的规定。

十一、托育综合服务中心的给水应符合现行国家标准《生活饮用水卫生标准》（GB5749）的规定。婴幼儿活动区域的电热水器等应有防止幼儿接触的保护措施。

十二、设置集中采暖系统的婴幼儿活动用房，散热器宜暗装。采用电采暖，必须有可靠的安全防护措施。

十三、托育综合服务中心的供电设施应安全可靠。室内照明宜采用带保护罩的节能灯具，应安装应急照明灯。婴幼儿活动用房应采用安全型插座。

十四、托育综合服务中心应根据使用特点和需求，设置相适应的智能化及信息系统，充分利用互联网、大数据、物联网、人工智能等技术，推动线上实景教学、线下线上融合，加强安全监督和防控。

**第五章 相关指标**

一、托育综合服务中心的投资估算应按国家现行有关规定编制，并根据工程实际内容及价格变化的情况，按照动态管理的原则进行调整。

二、新建独立托育综合服务中心应根据使用要求、区域特点，合理确定投资。

三、托育综合服务中心的经济评价和后评估应按照国家现行有关建设项目经济评价方法与参数的规定执行。
