# Supplementary material for: Mapping the evolution of fertility support policies in China: A content and instrumental analysis
Source: PLoS One. 2025 Oct 9;20(10):e0332137. doi: 10.1371/journal.pone.0332137 (PMC12510515; doi:10.1371/journal.pone.0332137)
Supplement: S1 Appendix — (ZIP) [file pone.0332137.s001.zip › S1 Appendix. 226 original policy documents/56-国务院办公厅关于印发“十四五”国民健康规划的通知(FBM-CLI-2-5117062).docx]

国务院办公厅关于印发“十四五”国民健康规划的通知

发布部门： 国务院办公厅 机构沿革

发文字号：国办发〔2022〕11号

发布日期：2022.04.27

实施日期：2022.04.27

时效性： 现行有效

效力级别： 国务院规范性文件

法规类别： 卫生综合规定 营商环境优化

国务院办公厅关于印发“十四五”国民健康规划的通知

（国办发〔2022〕11号）

各省、自治区、直辖市人民政府，国务院各部委、各直属机构：

《“十四五”国民健康规划》已经国务院同意，现印发给你们，请认真贯彻执行。

国务院办公厅

2022年4月27日

“十四五”国民健康规划

为全面推进健康中国建设，根据《中华人民共和国国民经济和社会发展第十四个五年规划和2035年远景目标纲要》、《“健康中国2030”规划纲要》，编制本规划。

一、规划背景

“十三五”时期，以习近平同志为核心的党中央把保障人民健康放在优先发展的战略位置，作出实施健康中国战略的决策部署。党中央、国务院召开全国卫生与健康大会，印发《“健康中国2030”规划纲要》。国务院印发《关于实施健康中国行动的意见》。各地各有关部门认真贯彻落实，扎实推进健康中国建设，启动实施健康中国行动，深入开展爱国卫生运动，持续完善国民健康政策。重大疾病防治成效显著，居民健康素养水平从10.25%提高到23.15%，人均基本公共卫生服务经费补助标准提高到74元，多数疫苗可预防传染病发病率降至历史最低水平，重大慢性病过早死亡率呈现下降趋势。重点人群健康服务不断完善，危重孕产妇和新生儿救治转运体系基本建立，儿童青少年近视监测和干预持续加强，老年健康与医养结合服务列入基本公共卫生服务。医药卫生体制改革深入推进，公立医院综合改革全面推开，药品和医用耗材加成全部取消，二级以上公立医院绩效考核全面实施；职工基本医疗保险、城乡居民基本医疗保险政策范围内住院费用支付比例分别稳定在80%和70%左右；基本药物数量从520种增加到685种，药品集中带量采购改革形成常态化机制，国家集中采购中选药品价格平均下降53%；医疗卫生服务体系不断完善，分级诊疗制度建设有序推进；社会办医稳步发展，健康产业规模显著扩大。健康扶贫任务全面完成，832个脱贫县县级医院服务能力全面提升，远程医疗服务覆盖全部脱贫县并向乡镇卫生院延伸，历史性消除脱贫地区乡村医疗卫生机构和人员“空白点”；大病专项救治病种扩大到30种，高血压等4种慢性病患者优先纳入家庭医生签约服务，2000多万贫困患者得到分类救治，近1000万因病致贫返贫户成功脱贫，基本医疗有保障全面实现。中医药服务体系持续完善，独特优势日益彰显。

经过努力，人民健康水平不断提高。2015年至2020年，人均预期寿命从76.34岁提高到77.93岁，婴儿死亡率从8.1‰降至5.4‰，5岁以下儿童死亡率从10.7‰降至7.5‰，孕产妇死亡率从20.1/10万降至16.9/10万，主要健康指标居于中高收入国家前列，个人卫生支出占卫生总费用的比重下降到27.7%。同时也应看到，我国仍面临多重疾病威胁并存、多种健康影响因素交织的复杂局面。全球新冠肺炎疫情仍处于大流行状态，新发突发传染病风险持续存在，一些已经控制或消除的传染病面临再流行风险。慢性病发病率上升且呈年轻化趋势，患有常见精神障碍和心理行为问题人数逐年增多，食品安全、环境卫生、职业健康等问题仍较突出。同时，人口老龄化进程加快，康复、护理等需求迅速增长。优生优育、婴幼儿照护服务供给亟待加强。需要加快完善国民健康政策，持续推进健康中国建设，不断满足人民群众日益增长的健康需求。

二、总体要求

（一）指导思想。坚持以习近平新时代中国特色社会主义思想为指导，全面贯彻党的十九大和十九届历次全会精神，统筹推进“五位一体”总体布局，协调推进“四个全面”战略布局，认真落实党中央、国务院决策部署，坚持稳中求进工作总基调，立足新发展阶段，完整、准确、全面贯彻新发展理念，构建新发展格局，把人民群众生命安全和身体健康放在第一位，贯彻新时代党的卫生健康工作方针，全面推进健康中国建设，实施积极应对人口老龄化国家战略，加快实施健康中国行动，深化医药卫生体制改革，持续推动发展方式从以治病为中心转变为以人民健康为中心，为群众提供全方位全周期健康服务，不断提高人民健康水平。

（二）基本原则。

健康优先，共建共享。加快构建保障人民健康优先发展的制度体系，推动把健康融入所有政策，形成有利于健康的生活方式、生产方式，完善政府、社会、个人共同行动的体制机制，形成共建共治共享格局。

预防为主，强化基层。把预防摆在更加突出的位置，聚焦重大疾病、主要健康危险因素和重点人群健康，强化防治结合和医防融合。坚持以基层为重点，推动资源下沉，密切上下协作，提高基层防病治病和健康管理能力。

提高质量，促进均衡。把提高卫生健康服务供给质量作为重点，加快优质医疗卫生资源扩容和区域均衡布局，不断提升基本医疗卫生服务公平性和可及性，缩小城乡、区域、人群之间资源配置、服务能力和健康水平差异。

改革创新，系统整合。坚持基本医疗卫生事业公益性，破除重点领域关键环节体制机制障碍。统筹发展和安全，提高重大风险防范处置能力。统筹预防、诊疗、康复，优化生命全周期、健康全过程服务。发挥中医药独特优势，促进中西医相互补充、协调发展。

（三）发展目标。到2025年，卫生健康体系更加完善，中国特色基本医疗卫生制度逐步健全，重大疫情和突发公共卫生事件防控应对能力显著提升，中医药独特优势进一步发挥，健康科技创新能力明显增强，人均预期寿命在2020年基础上继续提高1岁左右，人均健康预期寿命同比例提高。

——公共卫生服务能力显著增强。基本建成能有效应对重大疫情和突发公共卫生事件、适应国家公共卫生安全形势需要的强大公共卫生体系，早期监测、智能预警、快速反应、高效处置、综合救治能力显著提升。

——一批重大疾病危害得到控制和消除。艾滋病疫情继续控制在低流行水平，结核病发病率进一步降低，寄生虫病、重点地方病和人畜共患病危害持续得到控制和消除，重大慢性病发病率上升趋势得到遏制，心理相关疾病发生的上升趋势减缓，严重精神障碍、职业病得到有效控制。

——医疗卫生服务质量持续改善。基层医疗卫生服务能力不断提升，全方位全周期健康服务体系逐步健全，分级诊疗格局逐步构建，中医药特色优势进一步彰显。

——医疗卫生相关支撑能力和健康产业发展水平不断提升。适应行业特点的医学教育和人才培养体系逐步健全，卫生健康科技创新能力进一步增强，卫生健康信息化建设加快推进，健康服务、医药制造等健康产业持续发展。

——国民健康政策体系进一步健全。卫生健康法律法规体系更加完善，医药卫生体制改革持续深化，保障人民健康优先发展的制度体系和健康影响评价评估制度逐步建立，卫生健康治理能力和治理水平进一步提升。

主要发展指标

展望2035年，建立与基本实现社会主义现代化相适应的卫生健康体系，中国特色基本医疗卫生制度更加完善，人均预期寿命达到80岁以上，人均健康预期寿命逐步提高。

三、织牢公共卫生防护网

（一）提高疾病预防控制能力。明确各级疾病预防控制机构职责定位，强化疾病预防控制体系军民融合、防治结合、全社会协同，强化上级疾病预防控制机构对下级机构的业务领导和工作协同，强化医疗机构公共卫生责任。落实城乡基层医疗卫生机构疾病预防控制、公共卫生管理服务职责，完善疾病预防控制部门与城乡社区联动机制，夯实联防联控、群防群控的基础。创新医防协同机制，加强疾病预防控制机构对医疗机构疾病预防控制工作的技术指导和监督考核，建立完善人员通、信息通、资源通和监督监管相互制约的机制。探索推进疾病预防控制机构专业人员参与医疗联合体工作，推动县级疾病预防控制机构与县域医共体协同发展。持续完善国家基本公共卫生服务项目和重大传染病防控等项目，优化服务内涵，提高服务质量，实行科学动态调整，做到有进有出，提高防治结合和健康管理服务水平，推进基本公共卫生服务均等化。

（二）完善监测预警机制。完善传染病疫情和突发公共卫生事件监测系统，改进不明原因疾病和异常健康事件监测机制，强化公共卫生信息系统与医疗机构信息系统对接协同。充分发挥国家监测预警信息平台作用，探索建立跨区域疫情监测站点，实现不明原因传染病疫情和突发公共卫生事件实时分析、集中研判、及时报告。研究建立完善新发未知传染病多点触发预警机制，依托公共卫生、动物疫病、口岸检疫、食品安全、生态环境等系统拓展信息报告渠道，打通科研院所和第三方检测机构报告渠道，开通社会公众主动报告渠道。压实信息报告责任，明确传染病疫情和突发公共卫生事件的报告内容、程序、方式和时限等具体要求。健全风险评估方法和制度，提高监测分析、综合评价和潜在隐患早期识别能力。

（三）健全应急响应和处置机制。发挥集中统一高效的应急指挥体系作用，完善体制机制，实现监测预警、发现报告、风险评估、信息发布、应急处置和医疗救治等环节职责清晰、无缝对接，确保指令清晰、系统有序、条块畅达、执行有力。构建分层分类、高效实用的应急预案体系。完善传染病疫情和突发公共卫生事件分级应急响应机制，规范决策主体和处置原则，明确相关部门及机构的职责分工和工作机制。提升医务人员早期识别和应急处置水平，完善首诊负责、联合会诊等制度和处置流程，提高各级各类医疗卫生机构规范化处置能力。完善重大疫情医疗废物应急处置机制。依托大型综合医院，建立健全分级分类的卫生应急队伍，提高紧急医学救援能力。建立重大传染病疫情和突发事件国家救援力量整体调动与支援机制。

（四）提高重大疫情救治能力。全面提高二级以上综合医院（含中医医院，下同）感染性疾病科和发热门诊、留观室服务能力，全面提升急诊、重症、呼吸、检验、麻醉、消化、心血管、护理、康复等专科服务能力。提高医疗卫生机构实验室检测能力。依托高水平医疗卫生机构，发挥国家重大传染病防治基地作用，提高辐射带动能力。提高中医疫病防治能力。进一步完善地市级传染病救治网络，提高县级医院传染病检测和诊治能力。强化基层医疗卫生机构传染病防控能力。提升边境地区执法执勤力量科学应对重大疫情能力。加强医疗机构应急物资配置，鼓励企业、机关单位和居民参与储备，建立健全应急物资调配协同联动机制。

四、全方位干预健康问题和影响因素

（一）普及健康生活方式。

加强健康促进与教育。完善国家健康科普专家库和资源库，构建全媒体健康科普知识发布和传播机制，鼓励医疗机构和医务人员开展健康促进与健康教育。深入开展健康知识宣传普及，提升居民健康素养。开展健康县区建设，国家和省级健康县区比例不低于40%。进一步推进健康促进医院建设，二级以上医院中健康促进医院比例不低于50%。持续推进中小学健康促进专项行动，深化学校健康教育改革，切实保证学校健康教育时间，提升健康教育教学效果。

推行健康生活方式。全面实施全民健康生活方式行动，推进“三减三健”（减盐、减油、减糖，健康口腔、健康体重、健康骨骼）等专项行动。实施国民营养计划和合理膳食行动，倡导树立珍惜食物的意识和养成平衡膳食的习惯，推进食品营养标准体系建设，健全居民营养监测制度，强化重点区域、重点人群营养干预。开展控烟行动，大力推进无烟环境建设，持续推进控烟立法，综合运用价格、税收、法律等手段提高控烟成效，强化戒烟服务。加强限酒健康教育，控制酒精过度使用，减少酗酒。

开展全民健身运动。深化体卫融合，举办全民健身主题示范活动，倡导主动健康理念，普及运动促进健康知识。构建更高水平的全民健身公共服务体系，推进公共体育场馆和学校体育场馆开放共享，提高健身步道等便民健身场所覆盖面。保障学校体育课和课外锻炼时间。落实国民体质监测制度，推动国民体质监测站点与医疗卫生机构合作，在有条件的社区医疗卫生机构设立科学健身门诊。针对特殊人群开展体育健身指导，加强非医疗健康干预，建立完善运动处方库，推进处方应用。

（二）加强传染病、寄生虫病和地方病防控。

做好重点传染病防控。做好新冠肺炎疫情防控，完善落实常态化防控措施，巩固疫情防控成果。坚持多病共防，进一步加强流感、登革热等重点传染病监测和分析研判，统筹做好人感染禽流感、埃博拉出血热等新发突发传染病防控，有效防控霍乱、手足口病、麻疹等重点传染病疫情。强化鼠疫自然疫源地、重点地区和疫源不明地区动物间鼠疫的监测、疫源性调查、风险评估和及时处置，加强区域鼠疫联防联控。继续将艾滋病疫情控制在低流行水平，突出重点地区、重点人群和重点环节，有效落实宣传教育、综合干预、检测咨询、治疗随访、综合治理等防治措施。全面实施病毒性肝炎防治措施，开展消除丙肝公共卫生危害行动。全面落实结核病防治策略，加强肺结核患者发现和规范化诊疗，实施耐药高危人群筛查，强化基层医疗卫生机构结核病患者健康管理，加大肺结核患者保障力度。实施以传染源控制为主的狂犬病、布病等人畜共患病综合治理，加大动物源头防控力度。

强化疫苗预防接种。加强疫苗可预防传染病监测。稳妥有序做好新冠病毒疫苗接种工作，加强全流程管理，确保接种安全，逐步提高人群接种率。做好流感疫苗供应保障，推动重点人群流感疫苗接种。根据需要适时调整国家免疫规划疫苗种类。加强免疫规划冷链系统管理，提升追溯能力。加大疑似预防接种异常反应监测力度。

巩固重点寄生虫病、地方病防治成果。在血吸虫病流行区坚持以控制传染源为主的综合防治策略，加强黑热病等虫媒传染病防控，实施包虫病综合防治策略，持续保持消除疟疾状态。完善地方病防控策略，确保持续消除碘缺乏危害，保持基本消除燃煤污染型氟砷中毒、大骨节病和克山病危害，有效控制饮水型氟砷中毒、饮茶型地氟病和水源性高碘危害。

（三）强化慢性病综合防控和伤害预防干预。

实施慢性病综合防控策略。加强国家慢性病综合防控示范区建设，到2025年覆盖率达到20%。提高心脑血管疾病、癌症、慢性呼吸系统疾病、糖尿病等重大慢性病综合防治能力，强化预防、早期筛查和综合干预，逐步将符合条件的慢性病早诊早治适宜技术按规定纳入诊疗常规。针对35岁以上门诊首诊患者，积极推进二级以下医院和基层医疗卫生机构开展血压普查工作。在医院就诊人群中开展心脑血管疾病机会性筛查。推进机关、企事业单位、公共场所设置免费自助血压检测点，引导群众定期检测。推进“三高”（高血压、高血糖、高血脂）共管，高血压、Ⅱ型糖尿病患者基层规范管理服务率达到65%以上。将肺功能检查纳入40岁以上人群常规体检，推行高危人群首诊测量肺功能，提升呼吸系统疾病早期筛查和干预能力。多渠道扩大癌症早诊早治覆盖范围，指导各地结合实际普遍开展重点癌症机会性筛查。以龋病、牙周病等口腔常见病防治为重点，加强口腔健康工作，12岁儿童龋患率控制在30%以内。强化死因监测、肿瘤随访登记和慢性病与营养监测体系建设，探索建立健康危险因素监测评估制度。逐步建立完善慢性病健康管理制度和管理体系，推动防、治、康、管整体融合发展。

加强伤害预防干预。完善全国伤害监测体系，拓展儿童伤害监测，开发重点伤害干预技术标准和指南。实施交通安全生命防护工程，减少交通伤害事件的发生。加强儿童和老年人伤害预防和干预，减少儿童溺水和老年人意外跌倒。完善产品伤害监测体系，建立健全消费品质量安全事故强制报告制度，加强召回管理，减少消费品安全伤害。

（四）完善心理健康和精神卫生服务。

促进心理健康。健全社会心理健康服务体系，加强心理援助热线的建设与宣传，为公众提供公益服务。加强抑郁症、焦虑障碍、睡眠障碍、儿童心理行为发育异常、老年痴呆等常见精神障碍和心理行为问题干预。完善心理危机干预机制，将心理危机干预和心理援助纳入突发事件应急预案。

提高精神卫生服务能力。推广精神卫生综合管理机制，完善严重精神障碍患者多渠道管理服务。按规定做好严重精神障碍患者等重点人群救治救助综合保障。提高常见精神障碍规范化诊疗能力，鼓励上级精神卫生专业机构为县（市、区、旗）、乡镇（街道）开展远程服务。建立精神卫生医疗机构、社区康复机构及社会组织、家庭相衔接的精神障碍社区康复服务模式。

（五）维护环境健康与食品药品安全。

加强环境健康管理。深入开展污染防治行动，基本消除重污染天气，完善水污染防治流域协同机制，基本消除劣Ⅴ类国控断面和城市黑臭水体。加强噪声污染治理，全国声环境功能区夜间达标率达到85%。加强噪声对心脑血管、心理等疾病的健康风险研究。加强餐饮油烟治理。持续推进北方地区城市清洁取暖，加强农村生活和冬季取暖散煤替代。开展新污染物健康危害识别和风险评估。强化公共场所及室内环境健康风险评价。完善环境健康风险评估技术方法、监测体系和标准体系，逐步建立国家环境与健康监测、调查和风险评估制度。探索建立重大工程、重大项目健康影响评估技术体系。开展药品环境风险评估制度研究。加强医疗机构内部废弃物源头分类和管理，加快建设地级及以上城市医疗废弃物集中处置设施。加强排放物中粪大肠菌群、肠道病毒等指标监测。提升居民环境与健康素养，构建各方积极参与、协作共建健康环境的格局。

强化食品安全标准与风险监测评估。完善食品安全风险监测与评估工作体系和食品安全技术支持体系，提高食品安全标准和风险监测评估能力。实施风险评估和标准制定专项行动，加快制修订食品安全国家标准，基本建成涵盖从农田到餐桌全过程的最严谨食品安全标准体系，提高食品污染物风险识别能力。全面提升食源性疾病调查溯源能力。

保障药品质量安全。完善国家药品标准体系，推进仿制药质量和疗效一致性评价。建立符合中药特点的质量和疗效评价体系。构建药品和疫苗全生命周期质量管理机制，推动信息化追溯体系建设，实现重点类别来源可溯、去向可追。稳步实施医疗器械唯一标识制度。

（六）深入开展爱国卫生运动。

全面推进卫生城镇和健康城镇建设。深入推进国家卫生城镇创建，优化评审流程，引导推进全域创建和城乡均衡发展。总结推广健康城市试点的有效经验，打造一批健康城市样板，创造健康支持性环境。广泛开展健康县区、健康乡镇和健康细胞（健康村、健康社区、健康企业、健康机关、健康学校、健康促进医院、健康家庭等）建设，培育一批健康细胞建设特色样板。

改善城乡环境卫生。完善城乡环境卫生治理长效机制，提高基础设施现代化水平，统筹推进城乡环境卫生整治。加强城市垃圾和污水处理设施建设，推进城市生活垃圾分类和资源回收利用。推行县域生活垃圾和污水统筹治理，持续开展村庄清洁行动，建立健全农村村庄保洁机制和垃圾收运处置体系，选择符合农村实际的生活污水处理技术，推进农村有机废弃物资源化利用。加快研发干旱寒冷地区卫生厕所适用技术和产品，加强中西部地区农村户用厕所改造，加强厕所粪污无害化处理和资源化利用，务实推进农村厕所革命。实施农村供水保障工程。推进农贸市场标准化建设。强化以环境治理为主、以专业防制为辅的病媒生物防制工作。

创新社会动员机制。推动爱国卫生运动与传染病、慢性病防控等紧密结合，通过爱国卫生月等活动，加大科普力度，倡导文明健康、绿色环保的生活方式。制止餐饮浪费行为，坚决革除滥食野生动物等陋习，推广分餐公筷、垃圾分类投放等生活习惯。促进爱国卫生与基层治理工作相融合，发挥村规民约、居民公约的积极作用，推广居民健康管理互助小组、周末大扫除、卫生清洁日、环境卫生红黑榜、积分兑换等经验，完善社会力量参与机制，培育相关领域社会组织和专业社工、志愿者队伍，推动爱国卫生运动融入群众日常生活。

五、全周期保障人群健康

（一）完善生育和婴幼儿照护服务。

优化生育服务与保障。实施三孩生育政策，完善相关配套支持措施。继续做好生育保险对参保女职工生育医疗费用、生育津贴待遇等的保障，做好城乡居民医保参保人生育医疗费用保障，减轻生育医疗费用负担。做好生育咨询指导服务。推进“出生一件事”联办。完善国家生命登记管理制度，建立人口长期均衡发展指标体系，健全覆盖全人群、全生命周期的人口监测体系和预测预警制度。发挥计生协会组织作用，深入开展家庭健康促进行动。对全面两孩政策实施前的独生子女家庭和农村计划生育双女家庭，继续实行现行各项奖励扶助制度和优惠政策。动态调整扶助标准，建立健全计划生育特殊家庭全方位帮扶保障制度。支持有资质的社会组织接受计划生育特殊家庭委托，开展生活照料、精神慰藉等服务，依法代办入住养老机构、就医陪护等事务。

促进婴幼儿健康成长。完善托育服务机构设置标准和管理规范，建立健全备案登记、信息公示和质量评估等制度，加快推进托育服务专业化、标准化、规范化。研究制定托育从业人员学历教育和相关职业标准，提高保育保教质量和水平。鼓励和引导社会力量提供普惠托育服务，发展集中管理运营的社区托育服务网络，完善社区婴幼儿活动场所和设施。支持有条件的用人单位单独或联合相关单位在工作场所为职工提供托育服务。加强对家庭的婴幼儿早期发展指导，研究出台家庭托育点管理办法，支持隔代照料、家庭互助等照护模式，鼓励专业机构和社会组织提供家庭育儿指导服务。支持“互联网＋托育服务”发展，打造一批关键共性技术网络平台及直播教室，支持优质机构、行业协会开发公益课程，增强家庭的科学育儿能力。加强婴幼儿照护服务机构的卫生保健工作，预防控制传染病，降低常见病的发病率，保障婴幼儿的身心健康。

（二）保护妇女和儿童健康。

改善优生优育全程服务。实施母婴安全行动提升计划，全面落实妊娠风险筛查与评估、高危孕产妇专案管理、危急重症救治、孕产妇死亡个案报告和约谈通报等母婴安全五项制度，提供优质生育全程医疗保健服务。实施出生缺陷综合防治能力提升计划，构建覆盖城乡居民，涵盖婚前、孕前、孕期、新生儿和儿童各阶段的出生缺陷防治体系。加强婚前保健，推广婚姻登记、婚育健康宣传教育、生育指导“一站式”服务，为拟生育家庭提供科学备孕指导、孕前优生健康检查和增补叶酸指导服务，加强产前筛查和产前诊断。到2025年，孕前优生健康检查目标人群覆盖率不低于80%，产前筛查率不低于75%，新生儿遗传代谢性疾病筛查率达到98%以上。强化先天性心脏病、听力障碍、苯丙酮尿症、地中海贫血等重点疾病防治，推动围孕期、产前产后一体化管理服务和多学科诊疗协作。医疗卫生机构开展孕育能力提升专项攻关，规范人类辅助生殖技术应用，做好不孕不育诊治服务。支持妇幼保健机构整合预防保健和临床医疗服务。

加强妇女健康服务。发展妇女保健特色专科，提高服务能力，针对青春期、育龄期、孕产期、更年期和老年期妇女的健康需求，提供女性内分泌调节、心理、营养等预防保健服务以及妇女常见疾病治疗等涵盖生理、心理和社会适应的整合型医疗保健服务。促进生殖健康服务，推进妇女宫颈癌、乳腺癌防治，进一步提高筛查率和筛查质量。

促进儿童和青少年健康。实施母乳喂养促进行动，开展婴幼儿养育专业指导，加强婴幼儿辅食添加指导，实施学龄前儿童营养改善计划，降低儿童贫血患病率和生长迟缓率。实施健康儿童行动提升计划，完善儿童健康服务网络，建设儿童友好医院，加强儿科建设，推动儿童保健门诊标准化、规范化建设，加强儿童保健和医疗服务。加强对儿童青少年贫血、视力不良、肥胖、龋齿、心理行为发育异常、听力障碍、脊柱侧弯等风险因素和疾病的筛查、诊断和干预。指导学校和家长对学生实施防控综合干预，抓好儿童青少年近视防控。加强儿童心理健康教育和服务，强化儿童孤独症筛查和干预。推广青春健康教育工作，开展青少年性与生殖健康教育。统筹推进各级疾病预防控制机构学校卫生队伍和能力建设，加强对辖区学校卫生工作的指导。开展儿童健康综合发展示范县（市、区、旗）创建活动。

（三）促进老年人健康。

强化老年预防保健。开发老年健康教育科普教材，开展老年人健康素养促进项目，做好老年健康教育。加强老年期重点疾病的早期筛查和健康管理，到2025年，65岁及以上老年人城乡社区规范健康管理服务率达到65%以上。实施老年人失能预防与干预、老年人心理关爱、老年口腔健康、老年营养改善和老年痴呆防治等行动，延缓功能衰退。

提升老年医疗和康复护理服务水平。推动开展老年人健康综合评估和老年综合征诊治，促进老年医疗服务从单病种向多病共治转变。到2025年，二级以上综合医院设立老年医学科的比例达到60%以上。完善从居家、社区到专业机构的长期照护服务模式。提升基层医疗卫生机构康复护理服务能力，开展老年医疗照护、家庭病床、居家护理等服务，推动医疗卫生服务向社区、家庭延伸。支持有条件的医疗机构与残疾人康复机构等开展合作。稳步扩大安宁疗护试点。

提升医养结合发展水平。健全医疗卫生机构和养老服务机构合作机制，为老年人提供治疗期住院、康复期护理、稳定期生活照料、安宁疗护一体化的服务。进一步增加居家、社区、机构等医养结合服务供给。鼓励农村地区通过托管运营、毗邻建设、签约合作等多种方式实现医养资源共享。开展医养结合示范项目，提升服务质量和水平。

（四）加强职业健康保护。

强化职业健康危害源头防控和风险管控。建立健全职业病和职业病危害因素监测评估制度，扩大主动监测范围，到2025年，工作场所职业病危害因素监测合格率达到85%以上。开展尘肺病筛查和新兴行业及工作相关疾病等职业健康损害监测。完善用人单位职业健康信息及风险评估基础数据库，构建职业病危害风险分类分级、预测预警和监管机制，对职业病危害高风险企业实施重点监管。强化重点行业职业病危害专项治理。鼓励企业完善职业病防护设施，改善工作场所劳动条件。

完善职业病诊断和救治保障。健全职业病诊断与鉴定制度，优化诊断鉴定程序。强化尘肺病等职业病救治保障，实施分类救治救助，对未参加工伤保险且用人单位不存在或无法确定劳动关系的尘肺病患者，按规定落实基本医疗保障和基本生活救助政策。

加强职业健康促进。推动用人单位开展职工健康管理，加强职业健康管理队伍建设，提升职业健康管理能力。全面提高劳动者职业健康素养，倡导健康工作方式，显著提升工作相关的肌肉骨骼疾病、精神和心理疾病等防治知识普及率。推动健康企业建设，培育一批健康企业特色样板。深入开展争做“职业健康达人”活动。

（五）保障相关重点人群健康服务。

巩固拓展健康扶贫成果同乡村振兴有效衔接。过渡期内保持现有健康帮扶政策总体稳定，调整优化支持政策，健全因病返贫致贫动态监测机制，建立农村低收入人口常态化精准健康帮扶机制。加大对脱贫地区、“三区三州”、原中央苏区、易地扶贫搬迁安置地区等县级医院支持力度，鼓励开展对口帮扶、合作共建医疗联合体，重点提高传染病疫情和突发公共卫生事件监测预警、应急处置和医疗救治能力。加强脱贫地区乡村医疗卫生服务体系达标提质建设，支持采用巡诊派驻等方式保障乡村医疗卫生服务覆盖面，确保乡村医疗卫生机构和人员“空白点”持续实现动态清零。结合脱贫地区实际，推广大病专项救治模式，巩固并逐步提高重点人群家庭医生签约服务覆盖面和服务质量。

维护残疾人健康。加强残疾人健康管理，全面推进残疾人家庭医生签约服务。加强和改善残疾人医疗服务，完善医疗机构无障碍设施，强化残疾人服务设施和综合服务能力建设。建成康复大学，加快培养高素质、专业化康复人才。加强残疾人康复服务，提升康复医疗、康复训练、辅助器具适配等服务质量。建立儿童残疾筛查、诊断、康复救助衔接机制，确保残疾儿童得到及时有效的康复服务。加强残疾人心理健康工作，做好残疾人健康状况评估。贯彻实施《国家残疾预防行动计划（2021—2025年）》。继续开展防盲治盲，推动实施全面眼健康行动。继续推进防聋治聋，提升耳与听力健康水平。

六、提高医疗卫生服务质量

（一）优化医疗服务模式。

推行预约诊疗和日间服务。建立健全预约诊疗制度，全面推行分时段预约诊疗和检查检验集中预约服务，有序推进检查检验结果互认。推动三级医院日间手术等服务常态化、制度化，逐步扩大日间手术病种范围，稳步提高日间手术占择期手术的比例。鼓励有条件的医院设置日间病房、日间治疗中心等，为患者提供日间化疗、日间照射治疗等服务。

推广多学科诊疗。针对肿瘤、多系统多器官疾病、疑难复杂疾病等，推动建立多学科诊疗制度。鼓励将麻醉、医学检验、医学影像、病理、药学等专业技术人员纳入多学科诊疗团队，提升综合诊治水平。鼓励医疗机构采取多种方式设置服务协调员，在患者诊疗过程中予以指导协助和跟踪管理。

创新急诊急救服务。优化院前医疗急救网络。继续推进胸痛、卒中、创伤、危重孕产妇救治、危重新生儿和儿童救治等中心建设，为患者提供医疗救治绿色通道和一体化综合救治服务，提升重大急性疾病医疗救治质量和效率。完善智能化调度系统，推动院前医疗急救网络与院内急诊有效衔接，实现患者信息院前院内共享，构建快速、高效、全覆盖的急危重症医疗救治体系。

强化医防融合。依托国家基本公共卫生服务项目，以高血压和Ⅱ型糖尿病为切入点，实施城乡社区慢病医防融合能力提升工程，为每个乡镇卫生院和社区卫生服务中心培养1—2名具备医防管等能力的复合型骨干人员，探索建立以基层医生团队为绩效考核单元、以健康结果和居民满意度为导向的考核体系。推动预防、治疗、护理、康复有机衔接，形成“病前主动防，病后科学管，跟踪服务不间断”的一体化健康管理服务。

（二）加强医疗质量管理。

完善医疗质量管理与控制体系。强化医疗质量安全核心制度，健全国家、省、市三级质控组织体系，完善覆盖主要专业和重点病种的质控指标。完善国家、省、医疗机构三级感染监测体系，逐步将基层医疗卫生机构纳入监测。完善诊疗规范和技术指南，全面实施临床路径管理。可以在有条件的医疗联合体内探索建立一体化临床路径，为患者提供顺畅转诊和连续诊疗服务。

优化护理服务。健全护理服务体系，增加护士配备。强化基础护理，实施以病人为中心的责任制整体护理，开展延续护理服务。进一步扩大优质护理服务覆盖面，逐步实现二级以上医院全覆盖。通过培训、指导、远程等方式，在医疗联合体内将优质护理、康复护理、安宁疗护等延伸至基层医疗卫生机构。

提高合理用药水平。完善覆盖全国二级以上医院的合理用药监测系统，逐步将基层医疗卫生机构纳入监测。加强医疗机构药事管理，以抗菌药物、抗肿瘤药物、其他重点监控药物等为重点，加强用药监测和合理用药考核，抗菌药物使用强度符合规定要求。以临床需求为导向，推进药品使用监测和药品临床综合评价体系建设。加强药品不良反应监测。发挥临床药师作用，开设合理用药咨询或药物治疗管理门诊，开展精准用药服务。推动医疗联合体内药学服务下沉，临床药师指导基层医疗卫生机构提高合理用药水平，重点为签约服务的慢性病患者提供用药指导。

加强平安医院建设。严格落实医院安保主体责任，健全涉医矛盾纠纷多元化解机制，构建系统、科学、智慧的医院安全防范体系。建立完善医警数据共享和联动处置机制，依法严厉打击涉医违法犯罪特别是伤害医务人员的暴力犯罪行为。加强医疗服务人文关怀，大力推行医务社工、志愿者服务，构建和谐医患关系。

（三）加快补齐服务短板。

巩固提升基层服务网络。把乡村医疗卫生服务体系纳入乡村振兴战略全局统筹推进，提高县域医疗卫生服务整体水平。采取派驻、邻村延伸服务、流动巡诊等方式，保障乡、村两级医疗卫生服务全覆盖。开展基层卫生健康综合试验区建设。

提升血液供应保障能力。完善采供血网络布局。巩固血液核酸检测全覆盖成果。建立血液应急保障指挥平台，健全巩固常态化全国血液库存监测制度和血液联动保障机制，提高血液应急保障能力。加大无偿献血宣传动员力度，提升献血率。

七、促进中医药传承创新发展

（一）充分发挥中医药在健康服务中的作用。实施中医药振兴发展重大工程。实施中医药健康促进行动，推进中医治未病健康工程升级。提升地市级以上中医医院优势专科和县级中医医院特色专科服务能力，力争全部县级中医医院达到医疗服务能力基本标准。丰富中医馆服务内涵，促进中医适宜技术推广应用。探索有利于发挥中医药优势的康复服务模式。建立和完善国家重大疑难疾病中西医协作工作机制与模式。推进中医药博物馆事业发展，实施中医药文化传播行动，推动中医药文化进校园。发展中医药健康旅游。

（二）夯实中医药高质量发展基础。开展中医药活态传承、古籍文献资源保护与利用。提升中医循证能力。促进中医药科技创新。加快古代经典名方制剂研发。加强中药质量保障，建设药材质量标准体系、监测体系、可追溯体系。推动教育教学改革，构建符合中医药特点的人才培养模式。健全中医医师规范化培训制度和全科医生、乡村医生中医药知识培训机制。

八、做优做强健康产业

（一）推动医药工业创新发展。鼓励新药研发创新和使用，加快临床急需重大疾病治疗药物的研发和产业化，支持优质仿制药研发。加快构建药品快速应急研发生产体系，针对新发突发传染病以及其他涉及国家公共卫生安全的应急需求，加强对防控所需药品和医疗器械应急研发、检验检测、体系核查、审评审批、监测评价等工作的统一指挥与协调。建立国家参考品原料样本和病患信息应急调用机制，完善药品紧急研发攻关机制。深化药品医疗器械审评审批制度改革，对符合要求的创新药、临床急需的短缺药品和医疗器械、罕见病治疗药品等，加快审评审批。强化对经济实惠的精神疾病药物和长效针剂的研发攻坚。

（二）促进高端医疗装备和健康用品制造生产。优化创新医疗装备注册评审流程。开展原创性技术攻关，推出一批融合人工智能等新技术的高质量医疗装备。鼓励有条件的地方建设医疗装备应用推广基地，打造链条完善、特色鲜明的医疗装备产业集群。完善养老托育等相关用品标准体系，支持前沿技术和产品研发应用。围绕健康促进、慢病管理、养老服务等需求，重点发展健康管理、智能康复辅助器具、科学健身、中医药养生保健等新型健康产品，推动符合条件的人工智能产品进入临床试验。推进智能服务机器人发展，实施康复辅助器具、智慧老龄化技术推广应用工程。

（三）促进社会办医持续规范发展。鼓励社会力量在医疗资源薄弱区域和康复、护理、精神卫生等短缺领域举办非营利性医疗机构。引导促进医学检验中心、医学影像中心等独立设置机构规范发展，鼓励有经验的执业医师开办诊所。增加规范化健康管理服务供给，发展高危人群健康体检、健康风险评估、健康咨询和健康干预等服务。落实行业监管职责，促进社会办医规范发展。

（四）增加商业健康保险供给。鼓励围绕特需医疗、前沿医疗技术、创新药、高端医疗器械应用以及疾病风险评估、疾病预防、中医治未病、运动健身等服务，增加新型健康保险产品供给。鼓励保险机构开展管理式医疗试点，建立健康管理组织，提供健康保险、健康管理、医疗服务、长期照护等服务。在基本签约服务包基础上，鼓励社会力量提供差异化、定制化的健康管理服务包，探索将商业健康保险作为筹资或合作渠道。进一步完善商业长期护理保险支持政策。搭建高水平公立医院及其特需医疗部分与保险机构的对接平台，促进医、险定点合作。加快发展医疗责任险、医疗意外保险，鼓励保险机构开发托育机构责任险和运营相关保险。

（五）推进健康相关业态融合发展。促进健康与养老、旅游、互联网、健身休闲、食品等产业融合发展，壮大健康新业态、新模式。支持面向老年人的健康管理、预防干预、养生保健、健身休闲、文化娱乐、旅居养老等业态深度融合，创新发展健康咨询、紧急救护、慢性病管理、生活照护等智慧健康养老服务。强化国有经济在健康养老领域有效供给。推动健康旅游发展，加快健康旅游基地建设。选择教学科研资源丰富、医疗服务能力强、产业实力雄厚的城市或区域，以高水平医院为基础，完善综合协同政策，打造健康产业集群。

九、强化国民健康支撑与保障

（一）深化医药卫生体制改革。

加快建设分级诊疗体系。加强城市医疗集团网格化布局管理，整合医疗机构和专业公共卫生机构，为网格内居民提供一体化、连续性医疗卫生服务。加快推动县域综合医改，推进紧密型县域医共体建设，推进专科联盟和远程医疗协作网发展。稳步扩大家庭医生签约服务覆盖范围，加强基本公共卫生服务与家庭医生签约服务的衔接，提高签约服务质量。明确各级医疗卫生机构在相关疾病诊疗中的职责分工、转诊标准和转诊程序，形成连续通畅的双向转诊服务路径。推动三级医院提高疑难危重症和复杂手术占比，缩短平均住院日。

推动公立医院高质量发展。健全现代医院管理制度，充分发挥公立医院党委把方向、管大局、作决策、促改革、保落实的领导作用，健全全面预算管理、成本管理、预算绩效管理、内部审计和信息公开机制，推动医院管理科学化、精细化、规范化。全面开展公立医院绩效考核，持续优化绩效考核指标体系和方法。大力弘扬伟大抗疫精神和崇高职业精神，在全社会营造尊医重卫的良好氛围。推进优抚医院改革发展。提高监管场所医疗机构专业化水平。

深化相关领域联动改革。发挥好福建省三明市作为全国医改经验推广基地的作用，加大经验推广力度，按照“腾空间、调结构、保衔接”的路径，加快推进综合改革。健全全民医保制度，开展按疾病诊断相关分组、按病种分值付费，对于精神病、安宁疗护和医疗康复等需要长期住院治疗且日均费用较稳定的疾病推进按床日付费，将符合条件的互联网医疗服务按程序纳入医保支付范围。稳步建立长期护理保险制度。完善药品供应保障体系，扩大药品和高值医用耗材集中采购范围，落实集中采购医保资金结余留用政策，完善短缺药品监测网络和信息直报制度，保障儿童等特殊人群用药。深化医疗服务价格改革，规范管理医疗服务价格项目，建立灵敏有度的价格动态调整机制，优化中医医疗服务价格政策。深化人事薪酬制度改革，落实医疗卫生机构内部分配自主权，建立主要体现岗位职责和知识价值的薪酬体系。

健全医疗卫生综合监管制度。建立健全机构自治、行业自律、政府监管、社会监督相结合的医疗卫生综合监督管理体系，加强对服务要素准入、质量安全、公共卫生、机构运行、医疗保障基金、健康养老、托育服务和健康产业等的监管。积极培育医疗卫生行业组织，在制定行业管理规范和技术标准、规范执业行为、维护行业信誉、调解处理服务纠纷等方面更好发挥作用。提升卫生健康监督执法能力。构建更为严密的医疗卫生机构安全生产责任体系，加强医疗卫生机构危险化学品使用管理，落实医疗卫生机构消防安全管理责任，深入开展从业人员消防安全教育培训。

（二）强化卫生健康人才队伍建设。强化医教协同，推进以胜任力为导向的教育教学改革，优化医学专业结构。完善毕业后医学教育制度，支持新进医疗岗位的本科及以上学历临床医师均接受住院医师规范化培训。健全继续医学教育制度。强化基层人才队伍建设，加强全科医生临床培养培训，深入实施全科医生特岗计划、农村订单定向医学生免费培养和助理全科医生培训，有条件的地区探索实施“县聘乡用、乡聘村用”。开发退休医务人员人力资源，支持城市二级以上医院在职或退休医师到乡村医疗卫生机构多点执业或开办诊所。加强乡村卫生人才在岗培训和继续教育。加强疾控骨干人才队伍建设，提升现场流行病学调查等核心能力。完善公共卫生人员准入、使用和考核评价等机制。加强职业卫生复合型人才培养。加强药师队伍建设和配备使用。改革完善医务人员评价机制，坚持分层分类评价，突出品德能力业绩导向，增加临床工作数量和质量指标，探索试行成果代表作制度，淡化论文数量要求。

（三）加快卫生健康科技创新。推进医学科技创新体系的核心基地建设。新布局一批国家临床医学研究中心，形成覆盖全国的协同研究网络。加强疾病防控和公共卫生科研攻关体系与能力建设，汇聚力量协同开展重大传染病防控全链条研究。面向人民生命健康，开展卫生健康领域科技体制改革试点，启动卫生健康领域科技创新2030—重大项目、“十四五”重点研发计划等国家科技计划，实施“脑科学与类脑研究”等重大项目以及“常见多发病防治研究”、“生育健康及妇女儿童健康保障”等重点专项。健全涉及人的医学研究管理制度，规范生物医学新技术临床研究与转化应用管理。加快推广应用适合基层和边远地区的适宜医疗卫生技术。完善审批程序，加强实验室生物安全管理，强化运行评估和监管。完善高级别病原微生物实验室运行评价和保障体系，完善国家病原微生物菌（毒）种和实验细胞等可培养物保藏体系。

（四）促进全民健康信息联通应用。落实医疗卫生机构信息化建设标准与规范。依托实体医疗机构建设互联网医院，为签约服务重点人群和重点随访患者提供远程监测和远程治疗，推动构建覆盖诊前、诊中、诊后的线上线下一体化医疗服务模式。支持医疗联合体运用互联网技术便捷开展预约诊疗、双向转诊、远程医疗等服务。优化“互联网＋”签约服务，全面对接居民电子健康档案、电子病历，逐步接入更广泛的健康数据，为签约居民在线提供健康咨询、预约转诊、慢性病随访、健康管理、延伸处方等服务。推动“互联网＋慢性病（糖尿病、高血压）管理”，实现慢性病在线复诊、处方流转、医保结算和药品配送。推广应用人工智能、大数据、第五代移动通信（5G）、区块链、物联网等新兴信息技术，实现智能医疗服务、个人健康实时监测与评估、疾病预警、慢病筛查等。指导医疗机构合理保留传统服务方式，着力解决老年人等群体运用智能技术困难的问题。构建权威统一、互联互通的全民健康信息平台，完善全民健康信息核心数据库，推进各级各类医疗卫生机构统一接入和数据共享。探索建立卫生健康、医疗保障、药监等部门信息共享机制，通过全国一体化政务服务平台，实现跨地区、跨部门数据共享。研究制定数据开放清单，开展政府医疗健康数据授权运营试点。严格规范公民健康信息管理使用，强化数据资源全生命周期安全保护。

（五）完善卫生健康法治体系。贯彻落实基本医疗卫生与健康促进法，加快推动传染病防治法、突发公共卫生事件应对法、职业病防治法、中医药传统知识保护条例等法律法规的制修订工作，构建系统完备的卫生健康法律体系。加快完善医疗卫生技术标准体系，针对“互联网＋医疗健康”等新业态加快标准制修订。加强普法宣传。持续深化卫生健康领域“放管服”改革。

（六）加强交流合作。全方位推进卫生健康领域国际合作，推动构建人类卫生健康共同体。完善政策对话与协作机制，深入参与相关国际标准、规范、指南等的研究、谈判与制定。健全跨境卫生应急沟通协调机制。完善我国参与国际重特大突发公共卫生事件应对机制。深化中医药领域国际交流合作。促进“一带一路”卫生健康合作，推进健康丝绸之路建设。创新卫生发展援助与合作模式。深化与港澳台地区卫生健康交流合作。

十、强化组织实施

（一）加强组织领导。加强党对卫生健康工作的领导，强化政府责任，健全部门协作机制，及时细化完善政策措施，完善国民健康政策，推动各项任务落实。加快建立健康影响评价评估制度，推动经济社会发展规划中突出健康目标指标、公共政策制定实施中向健康倾斜、公共资源配置上优先满足健康发展需要。

（二）动员各方参与。强化跨部门协作，发挥工会、共青团、妇联、残联、计生协会等群团组织以及其他社会组织的作用，调动各企（事）业单位、学校、村（社区）积极性和创造性，鼓励相关行业学会、协会等充分发挥专业优势，将卫生健康工作纳入基层治理，引导群众主动落实健康主体责任、践行健康生活方式。

（三）做好宣传引导。发挥基层首创精神，鼓励地方结合实际积极探索创新。及时总结推广地方好的经验和做法，发挥示范引领作用。积极宣传推进健康中国建设相关政策措施，做好信息发布，加强正面宣传和典型报道。加强舆论引导，及时回应社会关切。

（四）强化监测评价。健全卫生健康规划体系，加强不同层级规划衔接。各有关部门要加强对地方的指导。建立健全规划实施监测评价机制，加强监测评估能力建设，对规划实施进行年度监测和中期、末期评估，及时发现和统筹研究解决实施中的问题。
