# Supplementary material for: Mapping the evolution of fertility support policies in China: A content and instrumental analysis
Source: PLoS One. 2025 Oct 9;20(10):e0332137. doi: 10.1371/journal.pone.0332137 (PMC12510515; doi:10.1371/journal.pone.0332137)
Supplement: S1 Appendix — (ZIP) [file pone.0332137.s001.zip › S1 Appendix. 226 original policy documents/57-国务院办公厅关于印发“十四五”全民医疗保障规划的通知(FBM-CLI-2-5077320).docx]

国务院办公厅关于印发“十四五”全民医疗保障规划的通知

发布部门： 国务院办公厅 机构沿革

发文字号：国办发〔2021〕36号

发布日期：2021.09.23

实施日期：2021.09.23

时效性： 现行有效

效力级别： 国务院规范性文件

法规类别： 医疗保健 营商环境优化

国务院办公厅关于印发“十四五”全民医疗保障规划的通知

（国办发〔2021〕36号）

各省、自治区、直辖市人民政府，国务院各部委、各直属机构：

《“十四五”全民医疗保障规划》已经国务院同意，现印发给你们，请认真贯彻执行。

国务院办公厅

2021年9月23日

“十四五”全民医疗保障规划

医疗保障是减轻群众就医负担、增进民生福祉、维护社会和谐稳定的重大制度安排。习近平总书记指出，要加快建立覆盖全民、城乡统筹、权责清晰、保障适度、可持续的多层次医疗保障体系。新一轮医改以来，贯彻党中央、国务院决策部署，我国已建成全世界最大、覆盖全民的基本医疗保障网，为全面建成小康社会、实现第一个百年奋斗目标作出了积极贡献。为进一步推进医疗保障高质量发展，保障人民健康，促进共同富裕，依据《中华人民共和国国民经济和社会发展第十四个五年规划和2035年远景目标纲要》和《中共中央 国务院关于深化医疗保障制度改革的意见》，制定本规划。

一、发展基础

党中央、国务院高度重视医疗保障工作，“十三五”期间，加强全民医疗保障制度顶层设计，推动医疗保障事业改革发展取得突破性进展，为缓解群众看病难、看病贵问题发挥了重要作用。

制度体系更加完善。以基本医疗保险为主体，医疗救助为托底，补充医疗保险、商业健康保险、慈善捐赠、医疗互助等共同发展的多层次医疗保障制度框架基本形成，更好满足了人民群众多元化医疗保障需求。统一的城乡居民基本医疗保险和大病保险制度全面建成。基本医疗保险统筹层次稳步提高。生育保险与职工基本医疗保险合并实施。长期护理保险制度试点顺利推进。

体制机制日益健全。整合医疗保险、生育保险、药品和医疗服务价格管理、医疗救助等职责，初步建立起集中统一的医疗保障管理体制。医保基金战略性购买作用初步显现，支付方式改革进一步深化，医保药品目录动态调整机制基本建立，定点医药机构协议管理更加规范，对医药体系良性发展的引导和调控作用明显增强。城乡居民高血压、糖尿病（以下统称“两病”）门诊用药保障机制普遍建立。

重点改革成效显著。药品集中带量采购工作实现常态化。高值医用耗材集中带量采购改革破冰。医疗服务价格合理调整机制初步形成。基金监管制度体系改革持续推进，飞行检查形成震慑，举报奖励机制初步建立，打击欺诈骗保专项治理成效显著，综合监管格局基本形成。“互联网＋医疗健康”等新模式蓬勃发展，医疗保障支持“互联网＋医疗健康”发展的机制初步成型。

基础支撑不断夯实。医疗保障信息化、标准化建设取得突破，医疗保障信息国家平台建成并投入使用，医保信息业务编码标准和医保电子凭证推广应用。制定《医疗保障基金使用监督管理条例》，医疗保障法治基础持续夯实。医疗保障经办管理服务体系初步理顺，政务服务事项实施清单管理，服务智能化、适老化程度显著提高。基金预算和绩效管理持续加强。

疫情应对及时有效。新冠肺炎疫情发生后，第一时间出台应对措施，确保患者不因费用问题影响就医，确保收治医院不因支付政策影响救治。加大医保基金预拨力度，及时结算医疗费用，支持医疗机构平稳运行。积极推行“不见面办”、“及时办”、“便民办”、“延期办”、“放心办”，确保疫情期间群众医保服务不断线。合理降低新冠病毒核酸检测价格，新冠病毒疫苗接种实行全民免费，有力支持疫情防控。

群众获得感持续增强。基本医疗保险覆盖13.6亿人，覆盖率稳定在95%以上，职工和城乡居民基本医疗保险政策范围内住院费用基金支付比例分别稳定在80%左右和70%左右，国家组织药品和高值医用耗材集中带量采购价格平均降幅50%以上。跨省异地就医住院费用直接结算全面推开，门诊费用跨省直接结算稳步试点，异地就医备案服务更加便捷。高质量打赢医疗保障脱贫攻坚战，助力近千万户因病致贫家庭精准脱贫，基本医疗有保障目标全面实现。基本医疗保险（含生育保险）五年累计支出8.7万亿元，2020年个人卫生支出占卫生总费用比例下降到27.7%。

当前，我国社会主要矛盾发生变化，城镇化、人口老龄化、就业方式多样化加快发展，疾病谱变化影响更加复杂，基金运行风险不容忽视，对完善医疗保障制度政策提出更高要求。同时，我国医疗保障发展仍不平衡不充分，多层次医疗保障体系尚不健全，重特大疾病保障能力还有不足，医保、医疗、医药改革协同性需进一步增强，医保服务与群众需求存在差距。但也要看到，我国制度优势显著，治理效能提升，经济长期向好，医疗保障制度框架基本形成，管理服务日趋精细，医疗保障改革共识不断凝聚，推动医疗保障高质量发展具有多方面的优势和条件。

二、总体要求

（一）指导思想。

以习近平新时代中国特色社会主义思想为指导，深入贯彻党的十九大和十九届二中、三中、四中、五中全会精神，按照党中央、国务院关于医疗保障工作的决策部署，立足新发展阶段，完整、准确、全面贯彻新发展理念，构建新发展格局，坚持以人民为中心的发展思想，深入实施健康中国战略，深化医药卫生体制改革，以推动中国特色医疗保障制度更加成熟定型为主线，以体制机制创新为动力，发挥医保基金战略性购买作用，坚持医疗保障需求侧管理和医药服务供给侧改革并重，加快建设覆盖全民、统筹城乡、公平统一、可持续的多层次医疗保障体系，努力为人民群众提供全方位全周期的医疗保障，不断提升人民群众的获得感、幸福感、安全感。

（二）基本原则。

--坚持党的全面领导。始终坚持党对医疗保障工作的领导，完善中国特色医疗保障制度，坚持制度的统一性和规范性，强化顶层设计，增强制度的刚性约束，为医疗保障制度更加成熟定型提供根本保证。

--坚持以人民健康为中心。把维护人民生命安全和身体健康放在首位，提供更加公平、更加充分、更高质量的医疗保障，使改革发展成果更多惠及全体人民，增进民生福祉，促进社会公平，推进共同富裕。

--坚持保障基本、更可持续。坚持实事求是，尽力而为、量力而行，把保基本理念贯穿始终，科学合理确定保障范围和标准，纠正过度保障和保障不足问题，提高基金统筹共济能力，防范和化解基金运行风险。

--坚持系统集成、协同高效。准确把握医疗保障各方面之间、医疗保障领域和相关领域之间改革的联系，建立基本医疗体系、基本医保制度相互适应的机制，统筹谋划，协调推进，汇聚改革合力，推动医疗保障改革取得更大突破。

--坚持精细管理、优质服务。深入推进医保领域“放管服”改革，加强管理服务能力建设，优化定点医药机构管理，健全基金监管长效体制机制。坚持传统服务方式和智能化应用创新并行，为群众提供更贴心、更暖心的服务。

--坚持共享共治、多方参与。促进多层次医疗保障有序衔接、共同发展，形成政府、市场、社会协同保障的格局。强化多主体协商共治，调动各方面积极性，凝聚改革发展共识，提高医疗保障治理水平。

（三）发展目标。

到2025年，医疗保障制度更加成熟定型，基本完成待遇保障、筹资运行、医保支付、基金监管等重要机制和医药服务供给、医保管理服务等关键领域的改革任务，医疗保障政策规范化、管理精细化、服务便捷化、改革协同化程度明显提升。

--建设公平医保。基本医疗保障更加公平普惠，各方责任更加均衡，保障范围和标准与经济社会发展水平更加适应，公共服务更加可及，制度间、人群间、区域间差距逐步缩小，医疗保障再分配功能持续强化。

--建设法治医保。医疗保障制度法定化程度明显提升，定点医药机构管理更加透明高效，基金监管制度体系更加完善，行政执法更加规范，全社会医保法治观念明显增强。

--建设安全医保。基金运行更加安全稳健，信息安全管理持续强化，防范和化解因病致贫返贫长效机制基本建立，医疗保障安全网更加密实。

--建设智慧医保。医疗保障信息化水平显著提升，全国统一的医疗保障信息平台全面建成，“互联网＋医疗健康”医保服务不断完善，医保大数据和智能监控全面应用，医保电子凭证普遍推广，就医结算更加便捷。

--建设协同医保。医疗保障和医药服务高质量协同发展，医保支付机制更加管用高效，以市场为主导的医药价格和采购机制更加完善，医疗服务价格调整更加灵敏有度。

注：①指“十四五”期间基本医疗保险参保率每年保持在95%以上。

②指到2025年各省（自治区、直辖市）国家和省级药品集中带量采购品种达500个以上。

③指到2025年各省（自治区、直辖市）国家和省级高值医用耗材集中带量采购品种达5类以上。

④指住院费用跨省直接结算人次占全部住院跨省异地就医人次的比例。

展望2035年，基本医疗保障制度更加规范统一，多层次医疗保障体系更加完善，医疗保障公共服务体系更加健全，医保、医疗、医药协同治理格局总体形成，中国特色医疗保障制度优越性充分显现，全民医疗保障向全民健康保障积极迈进。

三、健全多层次医疗保障制度体系

坚持公平适度、稳健运行，持续完善基本医疗保障制度。鼓励支持商业健康保险、慈善捐赠、医疗互助等协调发展。

（四）提升基本医疗保险参保质量。

依法依规分类参保。职工基本医疗保险覆盖用人单位及其职工，城乡居民基本医疗保险覆盖除职工基本医疗保险应参保人员以外的其他所有城乡居民。灵活就业人员可根据自身实际，以合适方式参加基本医疗保险。完善灵活就业人员参保缴费方式，放开对灵活就业人员参保的户籍限制。落实困难群众分类资助参保政策。

实施精准参保扩面。建立健全医疗保障部门与教育、公安、民政、人力资源社会保障、卫生健康、税务、市场监管、乡村振兴、残联等部门和单位的数据共享机制，加强数据比对，完善覆盖全民的参保数据库，实现参保信息实时动态查询。落实全民参保计划，积极推动职工和城乡居民在常住地、就业地参保，避免重复参保，巩固提高参保覆盖率。

优化参保缴费服务。深化医疗保险费征收体制改革，提高征缴效率。优化城乡居民参保缴费服务，积极发挥乡镇（街道）在参保征缴中的作用，加强医疗保障、税务部门和商业银行等“线上＋线下”合作，丰富参保缴费便民渠道。做好跨统筹地区参保人员基本医疗保险关系转移接续工作。

（五）完善基本医疗保障待遇保障机制。

促进基本医疗保险公平统一。完善职工基本医疗保险与城乡居民基本医疗保险分类保障机制，基金分别建账、分账核算。巩固提高基本医疗保险统筹层次，基本统一全国基本医疗保险用药范围，规范医保支付政策确定办法。坚持保基本定位，建立健全医疗保障待遇清单制度，确定基本保障内涵，厘清待遇支付边界，明确政策调整权限，规范政策制定流程。

合理确定待遇保障水平。根据经济社会发展水平和基金承受能力，稳定基本医疗保险住院待遇，稳步提高门诊待遇，做好门诊待遇和住院待遇的统筹衔接。健全职工基本医疗保险门诊共济保障机制，改革职工基本医疗保险个人账户。完善城乡居民基本医疗保险门诊保障政策，逐步提高保障水平。完善城乡居民“两病”门诊用药保障机制，推进“两病”早诊早治、医防融合。

规范补充医疗保险。完善和规范城乡居民大病保险制度，加强与基本医疗保险和医疗救助的衔接，提高保障能力和精准度。逐步规范职工大额医疗费用补助、企业补充医疗保险等制度。

统一规范医疗救助制度。建立救助对象及时精准识别机制。实施分层分类救助，规范救助费用范围，合理确定救助标准。建立健全防范和化解因病致贫返贫长效机制，协同实施大病专项救治，积极引导慈善等社会力量参与救助保障，强化互联网个人大病求助平台监管，促进医疗救助与其他社会救助制度的衔接。完善疾病应急救助管理运行机制，确保需急救的急重危伤病患者不因费用问题影响及时救治。

有效衔接乡村振兴战略。巩固拓展医保脱贫攻坚成果，实现由集中资源支持脱贫攻坚向基本医疗保险、大病保险、医疗救助三重制度常态化保障平稳过渡。分类优化医疗保障综合帮扶政策，坚决治理过度保障，将脱贫攻坚期地方自行开展的其他医疗保障扶贫措施资金逐步统一并入医疗救助基金。综合施策降低农村低收入人口看病就医成本，引导合理诊疗，促进有序就医，整体提升农村医疗保障和健康管理水平。

健全重大疫情医疗保障机制。在突发疫情等紧急情况时，确保医疗机构先救治、后收费，确保患者不因费用问题影响就医。探索建立重大疫情特殊群体、特定疾病医药费豁免制度，有针对性免除医保目录、支付限额、用药量等限制性条款，减轻困难群众就医就诊后顾之忧。统筹医保基金和公共卫生服务资金使用，对基层医疗机构实施差别化支付政策，实现公共卫生服务和医疗服务有效衔接。

完善生育保险政策措施。继续做好生育保险对参保女职工生育医疗费用、生育津贴等待遇的保障，规范生育医疗费用支付管理，推进生育医疗费用支付方式改革，住院分娩按病种支付，产前检查按人头支付，控制生育医疗费用不合理增长，降低生育成本，提高生育保险与职工基本医疗保险合并实施成效。继续做好城乡居民基本医疗保险参保人员生育医疗费用待遇保障。

（六）优化基本医疗保障筹资机制。

完善责任均衡的多元筹资机制。均衡个人、用人单位和政府三方筹资责任。建立基准费率制度，合理确定费率，研究规范缴费基数。提高统筹基金在职工基本医疗保险基金中的比重。完善城乡居民基本医疗保险筹资政策，研究建立缴费与经济社会发展水平和居民人均可支配收入挂钩的机制，优化个人缴费和政府补助结构。拓宽医疗救助筹资渠道，鼓励社会捐赠等多渠道筹资。加强财政对医疗救助的投入。

提高基金统筹层次。按照制度政策统一、基金统收统支、管理服务一体的标准，全面做实基本医疗保险市地级统筹。按照政策统一规范、基金调剂平衡、完善分级管理、强化预算考核、提升管理服务的方向，推动省级统筹。完善提高统筹层次的配套政策，夯实分级管理责任，强化就医管理和医疗服务监管。推动医疗救助统筹层次与基本医疗保险统筹层次相协调。建立健全与医疗保障统筹层次相适应的管理体系，探索推进市地级以下医疗保障部门垂直管理。

提升基金预算管理水平。科学编制医疗保障基金收支预算。加强预算执行监督，全面实施预算绩效管理，强化绩效监控、评价和结果运用。加强基金精算管理，构建收支平衡机制，建立健全基金运行风险评估预警机制，促进基金中长期可持续。探索开展跨区域基金预算试点。

（七）鼓励商业健康保险发展。

鼓励产品创新。鼓励商业保险机构提供医疗、疾病、康复、照护、生育等多领域的综合性健康保险产品和服务，逐步将医疗新技术、新药品、新器械应用纳入商业健康保险保障范围。支持商业保险机构与中医药机构合作开展健康管理服务，开发中医治未病等保险产品。更加注重发挥商业医疗保险作用，引导商业保险机构创新完善保障内容，提高保障水平和服务能力。

完善支持政策。厘清基本医疗保险责任边界，支持商业保险机构开发与基本医疗保险相衔接的商业健康保险产品，更好覆盖基本医保不予支付的费用。按规定探索推进医疗保障信息平台与商业健康保险信息平台信息共享。

加强监督管理。规范商业保险机构承办大病保险业务，建立并完善参与基本医疗保险经办的商业保险机构绩效评价机制。落实行业监管部门责任，加强市场行为监管，突出商业健康保险产品设计、销售、赔付等关键环节监管。

（八）支持医疗互助有序发展。

更好发挥医疗互助低成本、低缴费、广覆盖、广受益的优势，加强制度建设，强化监督管理，规范医疗互助发展。加强医疗互助与职工基本医疗保险的衔接，依托全国统一的医疗保障信息平台，推动医疗保障与医疗互助信息共享，充分发挥医疗保险和医疗互助的协同效应。坚持职工医疗互助的互济性和非营利性，推动科学设计、规范运营，更好减轻职工医疗费用负担，提高服务保障能力。

（九）稳步建立长期护理保险制度。

适应我国经济社会发展水平和老龄化发展趋势，构建长期护理保险制度政策框架，协同促进长期照护服务体系建设。从职工基本医疗保险参保人群起步，重点解决重度失能人员基本护理保障需求。探索建立互助共济、责任共担的多渠道筹资机制，参加长期护理保险的职工筹资以单位和个人缴费为主，形成与经济社会发展和保障水平相适应的筹资动态调整机制。建立公平适度的待遇保障机制，合理确定待遇保障范围和基金支付水平。制定全国统一的长期护理保险失能等级评估标准，建立并完善长期护理保险需求认定、等级评定等标准体系和管理办法，明确长期护理保险基本保障项目。做好与经济困难的高龄、失能老年人补贴以及重度残疾人护理补贴等政策的衔接。健全长期护理保险经办服务体系。完善管理服务机制，引入社会力量参与长期护理保险经办服务。鼓励商业保险机构开发商业长期护理保险产品。

四、优化医疗保障协同治理体系

发挥医保支付、价格管理、基金监管综合功能，促进医疗保障与医疗服务体系良性互动，使人民群众享有高质量、有效率、能负担的医药服务和更加优质便捷的医疗保障。

（十）持续优化医疗保障支付机制。

完善医保药品目录调整机制。立足基金承受能力，适应群众基本医疗需求、临床技术进步需要，建立并完善医保药品目录调整规则及指标体系，动态调整优化医保药品目录，及时将临床价值高、患者获益明显、经济性评价优良的药品按程序纳入医保支付范围。将符合条件的中药按规定纳入医保支付范围。健全医保药品评价机制，加强医保药品目录落地情况监测和创新药评价，支持药品创新，提高谈判药品可及性。2022年实现全国基本医保用药范围基本统一。建立健全医保药品支付标准，从谈判药品、集中带量采购药品和“两病”患者用药支付标准切入，逐步衔接医保药品目录管理和支付标准。

加强医保医用耗材管理。建立医保医用耗材准入制度，制定医保医用耗材目录。探索制定医用耗材医保支付标准，引导规范医疗服务行为，促进医用耗材合理使用。

提升医疗服务项目管理水平。完善医保医疗服务项目范围管理，明确医疗服务项目医保准入、支付、监管政策，规范医疗服务行为。在规范明细、统一内涵的基础上，逐步建立科学、公正、透明的医疗服务项目准入和动态调整机制，促进医疗服务新技术有序发展。支持将符合条件的中医医疗服务项目按规定纳入医保支付范围。

持续深化医保支付方式改革。在全国范围内普遍实施按病种付费为主的多元复合式医保支付方式，推进区域医保基金总额预算点数法改革，引导医疗机构合理诊疗，提高医保资金使用效能。制定医保基金总额预算管理、按床日付费、按人头付费等技术规范。完善紧密型医疗联合体医保支付政策。深化门诊支付方式改革，规范门诊付费基本单元，逐步形成以服务能力、服务项目、服务量为基础的支付方式。引导合理就医，促进基层首诊。探索符合中医药特点的医保支付方式，发布中医优势病种，鼓励实行中西医同病同效同价，引导基层医疗机构提供适宜的中医药服务。制定完善不同支付方式经办规程。探索医疗服务与药品分开支付。

健全对定点医药机构的预算分配机制。坚持“以收定支、收支平衡、略有结余”的总额预算编制原则，统筹考虑住院与门诊保障、药品（医用耗材）与医疗服务支付、地区内就医与转外就医等情况，完善分项分类预算管理办法，健全预算和结算管理机制。支持有条件的地区医保经办机构按协议约定向医疗机构预付部分医保资金，提高医保基金使用绩效。

加强医保定点管理。全面实施医疗保障定点医疗机构、医疗保障定点零售药店管理办法。优化定点管理流程，扩大定点覆盖面，将更多符合条件的基层医疗机构纳入医保定点范围。加强考核监督，完善定点医药机构绩效考核，制定针对不同支付方式的医疗服务行为监督管理办法，推动定点管理与医疗质量、协议履行相挂钩。

（十一）改革完善医药价格形成机制。

深化药品和医用耗材集中带量采购制度改革。常态化制度化实施国家组织药品集中带量采购，持续扩大国家组织高值医用耗材集中带量采购范围。强化对集中采购机构的统一指导，规范地方开展集中带量采购，形成国家、省级、跨地区联盟采购相互配合、协同推进的工作格局。建立以医保支付为基础，招标、采购、交易、结算、监督一体化的省级集中采购平台。推进并规范医保基金与医药企业直接结算，完善医保支付标准与集中采购价格协同机制。完善与集中带量采购相配套的激励约束机制，落实医保资金结余留用政策，推动集中带量采购成为公立医疗机构医药采购的主导模式，鼓励社会办医疗机构、定点零售药店参与集中带量采购。

完善药品和医用耗材价格治理机制。全面建立公立医疗机构药品和医用耗材采购价格信息监测机制、交易价格信息共享机制，提升对药品和医用耗材价格异常变动的分析预警应对能力。强化药品和医用耗材价格常态化监管，实施全国医药价格监测工程，全面落实医药价格和招采信用评价制度，灵活运用成本调查、函询约谈、信用评价、信息披露、价格指数、挂网规则等管理工具，遏制药品和医用耗材价格虚高，兼顾企业合理利润，促进医药行业高质量发展。

稳妥有序试点医疗服务价格改革。加强医疗服务价格宏观管理，完善定调价规则，改革优化定调价程序，探索适应经济社会发展、更好发挥政府作用、医疗机构充分参与、体现技术劳务价值的医疗服务价格形成机制。开展深化医疗服务价格改革试点，形成可复制的改革经验并有序推广。制定完善医疗服务价格项目编制规范，分类整合现行价格项目，健全医疗服务价格项目进入和退出机制，简化新增医疗服务价格申报流程，加快受理审核，促进医疗技术创新发展和临床应用。探索完善药学类医疗服务价格项目。健全上门提供医疗服务的价格政策。完善公立医疗机构价格监测，编制医疗服务价格指数，探索建立灵敏有度的动态调整机制，发挥价格合理补偿功能，稳定调价预期。加强总量调控、分类管理、考核激励、综合配套，提高医疗服务价格治理的社会化、标准化、智能化水平。

（十二）加快健全基金监管体制机制。

建立健全监督检查制度。建立并完善日常巡查、专项检查、飞行检查、重点检查、专家审查等相结合的多形式检查制度，健全“双随机、一公开”检查机制，规范不同检查形式的对象、内容、工作要求和流程，明确各方权利义务，确保公开、公平、公正。完善部门联动机制，开展联合检查，形成监管合力。引入信息技术服务机构、会计师事务所、商业保险机构等第三方力量参与医保基金监管，提升监管的专业性、精准性、效益性。

全面建立智能监控制度。提升医保智能监管能力，积极探索将按疾病诊断相关分组付费、按病种分值付费等新型支付方式、“互联网＋医疗健康”等新模式、长期护理保险等纳入智能监控范围，实现智能审核全覆盖，加强对定点医疗机构临床诊疗行为的引导和审核，实现基金监管从人工抽单审核向大数据全方位、全流程、全环节智能监控转变。

建立医疗保障信用管理体系。完善医疗保障信用管理制度，形成信用承诺、信用评价、信息共享、结果公开、结果应用、信用修复等全链条闭环式信用监管，推动实施分级分类监管。在充分掌握信用信息、综合研判信用状况基础上，根据信用等级高低，对监管对象采取差异化监管措施。以相关处理结果为依据，按程序将性质恶劣、情节严重、社会危害大的医疗保障违法失信行为的责任主体纳入严重失信主体名单，依法依规开展失信联合惩戒。建立药品和医用耗材生产流通企业等信用承诺制度，鼓励行业协会开展自律建设，促进行业规范发展。

健全综合监管制度。适应医疗保障管理服务特点，建立并完善部门间相互配合、协同监管的综合监管制度。大力推进部门联合执法、信息共享和互联互通，促进监管结果协同运用。对查实的欺诈骗保行为，各相关部门按照职责权限对有关单位和个人依规依纪依法严肃处理。加强基金监管行政执法与刑事司法有效衔接，按程序向公安机关移送涉嫌犯罪案件。

完善社会监督制度。广泛动员社会各界参与医疗保障基金监管，协同构建基金安全防线，促进形成社会监督的良好态势，实现政府治理和社会监督、舆论监督良性互动。健全欺诈骗保行为举报投诉奖励机制，完善奖励政策和奖励标准。健全完善要情报告制度，用好基金监管曝光台，做好医保基金监管典型案例的收集遴选和公开通报。医疗保障经办机构定期向社会公布参加基本医疗保险情况以及基金收入、支出、结余和收益情况，接受社会监督。

（十三）协同建设高效的医药服务供给体系。

优化提升医疗卫生服务体系。完善区域卫生规划和医疗机构设置规划，健全城市三级医院、县级医院和基层医疗卫生机构分工协作的现代医疗卫生服务体系，支持整合型医疗卫生服务体系建设，加强分级诊疗体系建设，推进基层医疗卫生机构发展，促进基层医疗卫生服务有效利用和患者有序就医。促进定点医药机构行业行为规范、成本控制和行业自律。支持中医药传承创新发展，强化中医药在疾病预防治疗中的作用，推广中医治未病干预方案。支持儿科、老年医学科、精神心理科和康复、护理等紧缺医疗服务发展。鼓励日间手术、多学科诊疗、无痛诊疗等医疗服务发展。完善检查检验政策，推进医疗机构检查检验结果互认。支持远程医疗服务、互联网诊疗服务、互联网药品配送、上门护理服务等医疗卫生服务新模式新业态有序发展，促进人工智能等新技术的合理运用。

提高医药产品供应和安全保障能力。深化审评审批制度改革，鼓励药品创新发展，加快新药好药上市，促进群众急需的新药和医疗器械研发使用。稳步推进仿制药质量和疗效一致性评价。分步实施医疗器械唯一标识制度，拓展医疗器械唯一标识在卫生健康、医疗保障等领域的衔接应用。严格药品监管，有序推进药品追溯体系建设。健全短缺药品监测预警和分级应对体系，加大对原料药垄断等违法行为的执法力度，进一步做好短缺药品保供稳价。逐步建立中标生产企业应急储备、库存和产能报告制度，保障集中采购药品供应。支持药店连锁化、专业化、数字化发展，更好发挥药店独特优势和药师作用。依托全国统一的医疗保障信息平台，支持电子处方流转。

强化协商共治机制。健全医疗保障部门、参保人代表、医院协会、医师协会、药师协会、护理学会、药品上市许可持有人、药品生产流通企业等参加的协商机制，构建多方利益协调的新格局，推动政策制定更加精准高效。

五、构筑坚实的医疗保障服务支撑体系

聚焦群众就医和医保需求，深入推进“放管服”改革，补短板、堵漏洞、强弱项，着力健全经办管理服务体系，提升医疗保障基础支撑能力，不断增强服务效能。

（十四）健全医疗保障公共服务体系。

加强经办管理服务体系建设。建立统一规范的医疗保障公共服务和稽核监管标准体系。统一经办规程，规范服务标识、窗口设置、服务事项、服务流程、服务时限，推进标准化窗口和示范点建设。建立覆盖省、市、县、乡镇（街道）、村（社区）的医疗保障服务网络。依托乡镇（街道）政务服务中心、村（社区）综合服务中心，加强医疗保障经办力量，大力推进服务下沉。在经办力量配置不足地区，可通过政府购买服务等方式，补齐基层医疗保障公共管理服务能力配置短板。加强医疗保障经办管理服务机构内控机制建设，落实协议管理、费用监控、稽查审核责任。建立绩效评价、考核激励、风险防范机制，提高经办管理服务能力和效率。

提升服务质量。坚持传统服务方式和新型服务方式“两条腿”走路，为参保群众提供优质服务，推进政务服务事项网上办理，健全多种形式的医疗保障公共管理服务。实现医疗保障热线服务与“12345政务服务便民热线”相衔接，探索实施“视频办”。建立健全跨区域医疗保障管理服务协作机制，推进高频医疗保障政务服务事项“跨省通办”落地实施。健全政务服务“好差评”制度，制定与医疗保障发展相适应的政务服务评价标准体系和评价结果应用管理办法。

完善异地就医直接结算服务。加强国家异地就医结算能力建设，实现全国统一的异地就医备案，扩大异地就医直接结算范围，逐步实现住院、门诊费用线上线下一体化的异地就医结算服务。

健全完善医保协议管理。简化优化定点医药机构专业评估、协商谈判程序，制定并定期修订医疗保障服务协议范本，加强事中事后监管。建立健全跨区域就医协议管理机制。合理确定统筹地区定点医药服务资源配置。

探索经办治理机制创新。推进经办管理服务与各地政务服务、网上政务服务平台衔接，鼓励商业保险机构等社会力量参与经办管理服务。加强定点医疗机构医保职能部门建设，发挥其联结医保服务与医院管理的纽带作用，加强定点医疗机构医保精细化管理，提升医疗卫生服务与医疗保障服务的关联度和协调性。

更好服务重大区域发展战略及高水平对外开放。推动重点区域医疗保障合作，提升区域医疗保障一体化发展水平。开展医疗保障领域对外交流合作，积极宣传医疗保障中国方案，为推动构建人类卫生健康共同体贡献中国智慧。

（十五）强化法治支撑。

建立健全法律法规体系。推进医疗保障法立法工作，夯实医疗保障事业改革和发展的法治基础。深入实施《医疗保障基金使用监督管理条例》。制定药品价格管理办法等规章，做好相关释义及解释。

规范医疗保障行政执法。完善权责清单、执法事项清单、服务清单，制定全国统一的行政处罚程序规定，规范执法文书样式、行政执法指引，约束行政执法自由裁量权。规范执法行为，改进执法方式，加强执法监督，建立健全医疗保障行政执法公示、执法全过程记录、重大执法决定法制审核等制度。健全行政复议案件处理工作机制。

（十六）推动安全发展。

强化基金管理。全面实施基金运行监控，提高基金管理水平，防范系统性风险，促进基金运行区域平衡。全面开展统筹地区基金运行评价，压实统筹地区管理责任。

确保数据安全。落实数据分级分类管理要求，制定医疗保障数据安全管理办法，规范数据管理和应用，依法保护参保人员基本信息和数据安全。强化医疗保障信息基础设施建设，维护信息平台运行安全。

加强内部控制。梳理医疗保障内部管理和职权运行风险点，建立健全流程控制、风险评估、运行监控、内部监督等内部控制工作机制，及时发现并有效防范化解安全隐患，确保不发生重大安全问题。强化责任追究，促进内控机制有效运行。

（十七）加快医保信息化建设。

全面建成全国统一的医疗保障信息平台。持续优化运行维护体系和安全管理体系，完善平台功能。依托全国统一的医疗保障信息平台，建立救助患者医疗费用信息共享机制。有效发挥国家智慧医保实验室作用。通过全国一体化政务服务平台，实现跨地区、跨部门数据共享，做好医疗保障数据分级分类管理，探索建立医疗保障部门与卫生健康、药监等部门信息共享机制。

完善“互联网＋医疗健康”医保管理服务。完善“互联网＋医疗健康”医保服务定点协议管理，健全“互联网＋”医疗服务价格和医保支付政策，将医保管理服务延伸到“互联网＋医疗健康”医疗行为，形成比较完善的“互联网＋医疗健康”医保政策体系、服务体系和评价体系。

提升医疗保障大数据综合治理能力。发挥全国统一的医疗保障信息平台优势，加强对医疗保障基础信息数据、结算数据、定点医药机构管理数据的采集、存储、清洗、使用，完善部门数据协同共享机制，探索多维度数据校验，提升精细化治理水平，提高医药资源配置效率。

（十八）健全标准化体系。

完善标准化工作基础。建立上下联动、部门合作、职责分明的标准化工作机制，推进医疗保障部门与人力资源社会保障、卫生健康、银保监、药监等部门的工作衔接。推动医疗保障标准在规范执业行为和促进行业自律等方面更好发挥作用。强化标准实施与监督。向定点医药机构提供标准服务。

加强重点领域标准化工作。统一医疗保障业务标准和技术标准，制定基础共性标准清单、管理工作标准清单、公共服务标准清单、评价监督标准清单，组建各类标准咨询专家团队。

健全标准化工作体制机制。组建全国医疗保障标准化技术委员会，建设高水平医疗保障标准化智库。强化医疗保障标准日常管理维护，完善落地应用长效机制。健全医保信息业务编码信息维护、审核、公示、发布的常态化工作机制。

六、做好规划实施

各地区、各有关部门要始终在思想上政治上行动上同以习近平同志为核心的党中央保持高度一致，增强“四个意识”、坚定“四个自信”、做到“两个维护”，确保医疗保障工作始终坚持正确政治方向，确保本规划各项任务落实到位。

（十九）健全实施机制。

建立健全国家和省两级医疗保障规划体系，加强两级规划衔接，确保全国医疗保障规划“一盘棋”。建立规划实施机制，做好规划重点任务分解，明确责任单位、实施时间表和路线图，提升规划实施效能。组织开展规划实施评估，监测重点任务进展、主要指标完成情况，及时完善优化政策。

（二十）强化能力建设。

加强医疗保障人才队伍建设，培养高素质专业化人才，鼓励高等院校、科研院所等与医疗保障部门开展合作，加强智库建设和人才支撑。实施医疗保障干部全员培训，开展多种形式挂职交流。建立体现医疗保障领域特点的人才评价机制，加大对先进单位和个人的表彰力度。

（二十一）营造良好氛围。

做好政府信息公开和新闻发布，及时准确发布权威信息，引导社会舆论，增进各方共识。开展多种形式的医保普法宣传活动，增强全社会医保法治意识，提高政策知晓度，营造医保、医疗、医药协同改革的良好氛围，为深化医疗保障制度改革创造良好舆论环境。
