# Supplementary material for: Mapping the evolution of fertility support policies in China: A content and instrumental analysis
Source: PLoS One. 2025 Oct 9;20(10):e0332137. doi: 10.1371/journal.pone.0332137 (PMC12510515; doi:10.1371/journal.pone.0332137)
Supplement: S1 Appendix — (ZIP) [file pone.0332137.s001.zip › S1 Appendix. 226 original policy documents/58-国务院办公厅关于印发国家残疾预防行动计划(2021—2025年)的通知(FBM-CLI-2-5113169).docx]

国务院办公厅关于印发国家残疾预防行动计划(2021—2025年)的通知

发布部门： 国务院办公厅 机构沿革

发文字号：国办发〔2021〕50号

发布日期：2021.12.14

实施日期：2021.12.14

时效性： 现行有效

效力级别： 国务院规范性文件

法规类别： 老少妇幼残保护

国务院办公厅关于印发国家残疾预防行动计划（2021-2025年）的通知

（国办发〔2021〕50号）

各省、自治区、直辖市人民政府，国务院各部委、各直属机构：

《国家残疾预防行动计划（2021-2025年）》已经国务院同意，现印发给你们，请认真贯彻执行。

国务院办公厅

2021年12月14日

国家残疾预防行动计划（2021-2025年）

残疾严重损害个人健康、家庭幸福，影响经济社会健康发展，做好残疾预防对于保障人民群众生命安全和身体健康、提高全民族健康素质、促进经济社会高质量发展具有重大意义。“十三五”期间，通过制定实施《国家残疾预防行动计划（2016-2020年）》，残疾预防工作取得显著成效。政府主导、多部门协调联动、社会共同参与的残疾预防工作格局初步形成，残疾预防法规政策更加完善，遗传和发育、疾病、伤害致残防控及残疾康复服务各项任务有效落实、工作目标如期实现。

当前，我国发展已进入新阶段，为贯彻落实党中央、国务院关于健康中国建设和新时代残疾人工作的决策部署，进一步加强残疾预防，有效减少和控制残疾发生、发展，依据《残疾预防和残疾人康复条例》等法规、政策，制定本行动计划。

一、总体要求

（一）指导思想。

以习近平新时代中国特色社会主义思想为指导，全面贯彻党的十九大和十九届历次全会精神，认真落实党中央、国务院决策部署，坚持以人民为中心的发展思想，贯彻预防为主的方针，以基层为重点，以改革创新为动力，将残疾预防融入经济社会发展各领域，全民动员、科学施策、依法推进，提高全社会残疾风险综合防控能力，有力保障人民群众生命安全和身体健康。

（二）基本原则。

政府主导，联防联控。进一步完善政府主导、多部门协调联动、社会共同参与的残疾预防工作格局。强化政府责任，加强跨部门协作，完善防治策略、制度安排和保障政策。落实单位、个人责任，调动全社会积极性，形成政府、社会、个人协同推进残疾预防的合力。

人人尽责，共建共享。倡导每个人是自己健康第一责任人的理念，把增强公民个人残疾预防意识和能力作为残疾预防的基础工程抓紧、抓实，广泛开展残疾预防宣传教育，让残疾预防知识、行为和技能成为全民普遍具备的素养和能力。

系统推进，早期干预。全面实施覆盖全人群全生命周期的残疾预防三级防控策略，着力推进关口前移、早期干预。针对各阶段主要致残因素采取综合干预措施，推进健康教育、健康促进，提供系统连续的筛查、诊断、治疗、康复一体化服务。

（三）工作目标。

到2025年，覆盖经济社会发展各领域的残疾预防政策体系进一步完善，全人群全生命周期残疾预防服务网络更加健全，全民残疾预防素养明显提升，遗传和发育、疾病、伤害等主要致残因素得到有效防控，残疾康复服务状况持续改善，残疾预防主要指标处于中高收入国家前列。

（四）主要指标。

二、主要行动

（一）残疾预防知识普及行动。

建立完善残疾预防科普知识资源库。出版、遴选、推介一批残疾预防科普读物，针对重点人群、主要致残因素定期更新、发布残疾预防核心知识。推动将残疾预防和出生缺陷防治核心知识纳入全科医生、专科医生、妇幼保健人员、社会工作人员、残疾人工作者等职业培训课程和教材内容，形成残疾预防知识科普骨干队伍，确保残疾预防知识规范、有效传播。（中国残联、国家卫生健康委牵头，中央宣传部、中央网信办、教育部、司法部、生态环境部、交通运输部、应急部、广电总局、国家疾控局、全国总工会、共青团中央按职责分工负责）

加强重点人群残疾预防知识普及。面向儿童、青少年、新婚夫妇、孕产妇、婴幼儿家长、老年人、高危职业从业者等重点人群开展针对性宣传教育，主动提供残疾预防和出生缺陷防治科普知识，普及遗传和发育、疾病、伤害等致残防控的科学知识、方法；面向伤病者、残疾人，加强康复知识宣传普及，着力提升康复意识、能力。（中国残联、国家卫生健康委牵头，教育部、民政部、司法部、生态环境部、交通运输部、应急部、国家疾控局、全国总工会、共青团中央、全国妇联按职责分工负责）

组织实施重点宣传教育行动。持续开展残疾预防日宣传教育活动，同时利用爱耳日、爱眼日、世界噪音日、防治碘缺乏病日、预防出生缺陷日、精神卫生日、防灾减灾日、全国消防日、全国交通安全日等宣传节点，加强残疾预防知识专题宣传，充分利用群众喜闻乐见的活动形式、传播方式，提升各类宣传教育活动的影响力、实效性。（中国残联、国家卫生健康委、中央宣传部牵头，中央网信办、教育部、工业和信息化部、公安部、民政部、司法部、人力资源社会保障部、生态环境部、交通运输部、应急部、广电总局、国家疾控局、全国总工会、共青团中央、全国妇联按职责分工负责）

（二）出生缺陷和发育障碍致残防控行动。

加强婚前、孕前保健。推进婚前保健，加强对遗传性疾病、指定传染病、严重精神障碍的检查并提出医学意见，指导婚前医学检查服务机构科学优化婚前医学检查场所布局及服务流程，加强婚姻登记场所婚姻家庭健康咨询室建设，加大健康婚育指导力度。深入实施孕前优生健康检查、增补叶酸等基本公共卫生服务，指导科学备孕，为计划怀孕夫妇提供健康教育、咨询指导、筛查评估、综合干预等孕前优生服务，推进补服叶酸预防神经管缺陷。（国家卫生健康委牵头，民政部、全国妇联按职责分工负责）

做好产前筛查、诊断。提供生育全程基本医疗保健服务，广泛开展产前筛查，加强对常见胎儿染色体病、严重胎儿结构畸形、单基因遗传病等重大出生缺陷的产前筛查和诊断。推进高龄孕产妇等重点人群的分类管理和服务，落实妊娠风险筛查与评估、高危孕产妇专案管理等制度，强化县、乡、村三级妇幼卫生服务网络建设，完善基层网底和转诊网络。（国家卫生健康委负责）

加强儿童早期筛查和早期干预。全面开展新生儿苯丙酮尿症、先天性甲状腺功能减低症等遗传代谢性疾病和听力筛查，逐步扩大致残性疾病筛查病种范围，推进早筛、早诊、早治。规范婴幼儿早期发展服务，加强对家庭和托幼机构儿童早期发展服务的指导，深入实施中央专项彩票公益金出生缺陷干预救助项目。做实0-6岁儿童健康管理工作，大力推进0-6岁儿童致残性疾病筛查，建立筛查、诊断、康复救助衔接机制，不断提升儿童致残性疾病早发现、早诊断、早干预、早康复能力和效果。（国家卫生健康委、中国残联牵头，教育部、全国妇联按职责分工负责）

（三）疾病致残防控行动。

加强慢性病致残防控。推广健康生活方式，提倡戒烟限酒、合理膳食、均衡营养、科学运动，减少每日食用油、盐、糖摄入量。开展全民健身行动，发挥好体育健身在主动健康干预、慢性病防治、康复中的作用。加强高血压、糖尿病等慢性病患者规范管理，做好并发症筛查和干预。丰富家庭医生签约服务内容，提高服务质量，推进基层慢性病医防融合管理。持续开展脑卒中等高危人群筛查与干预项目。着力做好防盲治盲、防聋治聋工作。（国家卫生健康委、国家疾控局牵头，教育部、体育总局、中国残联按职责分工负责）

加强社会心理服务和精神疾病防治。构建社会心理健康服务体系，强化重点人群心理健康服务、社会工作服务和个体危机干预，加强群体危机管理，将心理援助纳入突发事件应急预案，为遭遇突发公共事件群体提供心理援助服务。加强对精神分裂症、阿尔茨海默症、抑郁症、孤独症等主要致残性精神疾病的筛查识别和治疗。做好严重精神障碍患者规范管理，落实监管责任，加强救治救助。（中央政法委、公安部、民政部、国家卫生健康委、应急部、国家中医药局、国家疾控局、全国妇联、中国残联按职责分工负责）

加强传染病及地方病致残防控。全面实施国家免疫规划，继续将脊髓灰质炎、流行性乙型脑炎等致残性传染病的疫苗接种率维持在高水平。落实《中华人民共和国疫苗管理法》，保证疫苗使用安全。加强传染病防控，做好传染病报告及患者医疗救治。针对地方病流行状况，实行重点地方病监测全覆盖，持续消除碘缺乏病、大骨节病、氟骨症等重大地方病致残。（国家卫生健康委、国家疾控局牵头，各省级人民政府负责）

加强职业病致残防控。加强职业健康监管体系建设，做好重点行业职业健康管理，督促用人单位落实职业病防治主体责任，提升职业健康工作水平。落实防尘、防毒、防噪声、防辐射等重点措施，减少工作场所职业危害因素。加强重点人群劳动保护，避免接触有毒有害因素。加强严重致残职业病患者救治，预防尘肺病、职业中毒、噪声等致残。（国家卫生健康委牵头，国家发展改革委、人力资源社会保障部、应急部、全国总工会按职责分工负责）

（四）伤害致残防控行动。

加强安全生产和消防安全监督管理。加大安全生产监管执法力度，排查治理重点行业领域重大事故隐患，持续改善工矿行业劳动条件。大力推进工伤预防工作，减少因工伤致残。加强消防安全治理，排查治理客运车站、码头、医院、学校、幼儿园、养老院、儿童福利机构、未成年人救助保护机构及劳动密集型企业等人员密集场所的消防安全隐患，完善消防安全设施，提高防范火灾能力。（应急部牵头，教育部、民政部、人力资源社会保障部、住房城乡建设部、交通运输部、国家卫生健康委、全国总工会按职责分工负责）

加强道路交通和运输安全管理。加强交通安全系统治理、依法治理、综合治理、源头治理，深化隐患排查治理，提升道路设施安全保障水平，加大严重交通违法行为查处力度。加强道路运输指挥调度、动态监测、应急处置。加强旅游包车、班线客车、危险货物运输车、货车等重点车辆安全管理，推动落实政府领导责任、行业部门监管责任和企业安全主体责任。加强机动车生产、改装、登记、检验等环节监管。加强道路交通事故伤者救援渠道和救治网络建设，减少交通事故致残。（公安部、交通运输部牵头，工业和信息化部、文化和旅游部、国家卫生健康委、应急部、市场监管总局按职责分工负责）

加强儿童伤害和老年人跌倒致残防控。开展学校、幼儿园、社区、家庭儿童伤害综合干预，推广“四安全”儿童伤害综合干预模式，积极开展针对儿童溺水、道路交通伤害、跌落、烧烫伤、中毒、暴力等风险的安全教育，健全儿童用品强制性国家标准体系，加强对玩具、电子产品的监督和管理。推广使用儿童安全座椅。加强老年友好环境建设，鼓励家居环境适老化改造，改造易致跌倒的危险环境。开展老年人跌倒干预和健康指导，提高老年人及其照料者预防跌倒的意识和能力。提高对儿童伤害和老年人跌倒的救援、救治水平。（教育部、公安部、民政部、国家卫生健康委、市场监管总局、国家疾控局、全国妇联按职责分工负责）

增强防灾减灾能力。加强灾害风险隐患排查及群众性应急演练。做好灾害监测预警、应急准备、应急救援、生活救助、恢复重建等工作，加强社区、学校、幼儿园、医院、车站、工厂等人员密集场所灾害防御，依托现有资源，推动建设全国应急救援医疗平台，提高突发灾害现场应急处置能力和水平。完善应急医疗技能实战训练、救援人员康复治疗技能培训、移动医院和医疗救援装备储备等。（应急部牵头，教育部、民政部、自然资源部、交通运输部、水利部、国家卫生健康委、中国气象局按职责分工负责）

加强农产品和食品药品安全监管。聚焦突出问题，防范化解农产品质量安全风险隐患，推进农产品质量安全治理现代化。完善食品生产安全风险防控体系和分级管理制度，加强食品安全风险动态排查，定期开展风险评估研判，加强生产经营过程监管，加大抽检力度，严惩重处违法行为，压实企业主体责任。严厉打击制售假劣药品、无证医疗器械违法行为，持续加强药品不良反应和医疗器械不良事件监测。（市场监管总局牵头，农业农村部、国家卫生健康委、国家药监局按职责分工负责）

保障饮用水安全和加强空气、噪声污染治理。全面开展城乡饮用水卫生监测，及时掌握全国饮用水水质基本状况，确保达到生活饮用水卫生标准。加强水源保护和水质保障，推动城市供水设施建设改造，保障城市供水安全，推进农村饮水安全向农村供水保障转变。持续开展大气污染防治行动，强化工业企业无组织排放管控，推进工业污染源全面达标排放，加大超标处罚和联合惩戒力度。大力推进企业清洁生产，推动重点行业污染治理升级改造，积极推进钢铁等行业超低排放改造，深入推进柴油货车污染治理，实施清洁取暖等措施，加强环境空气质量监测，做好重污染天气应急响应。加强噪声污染治理，推动地级及以上城市全面实现功能区声环境质量自动监测。强化生态环境与健康管理，减少饮用水、空气、噪声等环境污染致残。（生态环境部牵头，自然资源部、住房城乡建设部、水利部、国家卫生健康委按职责分工负责）

（五）康复服务促进行动。

加强康复医疗服务。贯彻落实国家卫生健康委等八部门印发的《关于加快推进康复医疗工作发展的意见》，提高康复医疗服务能力，完善康复医疗服务指南和技术规范，积极发展中医特色康复服务。加强康复医疗人才教育培养，加快建设康复大学，鼓励有条件的院校设置康复治疗、康复工程等相关学科和专业。积极发展社区和居家康复医疗，鼓励有条件的医疗机构将机构内康复医疗服务延伸至社区和家庭。（国家发展改革委、教育部、民政部、国家卫生健康委、国家中医药局、中国残联按职责分工负责）

保障残疾人基本康复服务。落实政府基本公共服务责任，开展残疾人基本需求与服务状况调查，持续组织实施残疾人精准康复服务行动，为残疾人提供康复医疗、康复训练、康复辅助器具配置等基本康复服务。加强残疾人康复机构规范化建设，着力推进精神障碍、智力残疾等社区康复服务。健全基本康复服务、康复辅助器具适配服务标准规范，持续提升残疾康复服务质量。落实残疾儿童康复救助制度，合理确定救助标准，增加康复服务供给，确保残疾儿童得到及时有效的康复服务。有条件的地方可对城乡困难残疾人、重度残疾人基本型辅助器具适配给予补贴。（中国残联牵头，教育部、民政部、国家卫生健康委按职责分工负责）

加强长期照护服务。完善居家、社区、机构相衔接的专业化长期照护服务体系，改善失能老年人照护服务质量，努力延缓残疾发生、发展。落实经济困难的失能老年人补贴制度，加强与残疾人两项补贴政策衔接。稳步推进长期护理保险制度试点，推动形成符合我国国情的长期护理保险制度。鼓励发展商业性长期护理保险产品，为参保人提供个性化长期照护服务。（民政部、国家卫生健康委、市场监管总局、国家医保局、银保监会按职责分工负责）

提升无障碍设施建设水平。修订完善无障碍环境建设标准，组织创建全国无障碍建设城市，持续推动城市道路、公共交通、居住社区、公共服务设施和残疾人服务设施等加快无障碍建设和改造。实施困难重度残疾人家庭无障碍改造，提高残疾人家庭无障碍改造水平。探索传统无障碍设施设备数字化、智能化升级。加快发展信息无障碍，加快普及互联网网站、移动互联网应用程序和自助公共服务设备无障碍。（住房城乡建设部牵头，中央网信办、工业和信息化部、交通运输部、广电总局、中国残联按职责分工负责）

三、保障措施

（一）加强组织领导。国务院残疾人工作委员会负责组织实施本行动计划，指导各地、各有关部门及单位落实相关工作任务，定期召开会议，听取汇报，通报情况，开展调度，研究解决重大问题。各地要结合实际研究制定本地残疾预防行动计划，健全工作推进机制，保障工作条件，加强统筹调度，确保实现各项任务目标。各有关部门要按照职责分工，将所承担的残疾预防工作任务纳入重点工作安排，逐项抓好落实。（各级残疾人工作委员会及其成员单位、有关单位按职责分工负责）

（二）健全技术支撑体系。完善国家残疾预防专家咨询委员会，建立健全各省（自治区、直辖市）残疾预防专家咨询委员会，承担咨询、评估、宣教等任务，为本行动计划实施提供技术支持。加强残疾预防科技攻关、示范应用，针对残疾预防重点难点，结合中央财政科技计划（专项、基金等）以及地方科技发展专项等给予支持。强化残疾预防信息支撑，推动残疾预防信息跨部门跨区域共享。确定残疾预防重点联系地区，加强监测，探索经验，开展残疾预防新技术示范应用。（教育部、科技部、公安部、民政部、财政部、人力资源社会保障部、生态环境部、住房城乡建设部、交通运输部、国家卫生健康委、应急部、市场监管总局、国家疾控局、中国残联按职责分工负责）

（三）开展监测评估。国务院残疾人工作委员会成员单位和有关单位按职责分工做好相关任务指标年度监测，及时收集、分析反映相关任务落实情况的数据和信息。国务院残疾人工作委员会组织开展中期及终期评估，通过评估了解掌握本行动计划实施进展情况，系统分析评价目标任务完成情况，总结经验做法，找出突出问题，提出对策建议。地方各级残疾人工作委员会负责组织有关单位，做好本地残疾预防行动计划实施情况监测评估。对进度滞后、工作不力的地区、部门和单位，及时督促整改。（各级残疾人工作委员会及其成员单位、有关单位按职责分工负责）

（四）做好宣传引导。采取多种方式，强化舆论宣传，编写发布解读材料，宣传介绍实施本行动计划的重大意义、目标任务和主要举措，帮助社会各界了解掌握核心内容，鼓励引导社会广泛参与、支持实施。及时宣传报道实施进展、阶段性成效，做好经验交流分享，为推进实施营造良好氛围。（中国残联、国家卫生健康委牵头，中央宣传部、中央网信办、教育部、工业和信息化部、公安部、民政部、司法部、生态环境部、住房城乡建设部、交通运输部、应急部、市场监管总局、广电总局、国家疾控局、全国总工会、共青团中央、全国妇联按职责分工负责）
